# Asymmetric C–H Functionalization of *N*‑Boc-2,5-dihydro‑1*H*‑pyrrole and Its Application as a Key Step in the Synthesis of (−)-Dragocin D

**DOI:** 10.1021/jacs.5c08080

**Published:** 2025-07-28

**Authors:** Terrence-Thang H. Nguyen, Kristin Shimabukuro, Djamaladdin G. Musaev, John Bacsa, Antonio Navarro, Huw M. L. Davies

**Affiliations:** † Department of Chemistry, 1371 Emory University, Atlanta, Georgia 30322, United States; ‡ Cherry L. Emerson Center for Scientific Computation, Emory University, 1521 Dickey Drive, Atlanta, Georgia 30322, United States; § Lilly Research Laboratories, Eli Lilly and Company, Indianapolis, Indiana 46285, United States

## Abstract

Dirhodium tetracarboxylate-catalyzed reaction of aryldiazoacetates with *N*-Boc-2,5-dihydro-1*H*-pyrrole results in a highly enantio- and diastereoselective C–H functionalization exclusively at the α-*N* C2 position. This result is a sharp contrast to the reaction with ethyl diazoacetate, which results in cyclopropanation of the olefinic site. Rh_2_(*S*- or *R*-PTAD)_4_ is the optimal chiral catalyst and is capable of generating the C–H functionalization products in up to 87% yield with high levels of diastereoselectivity (>20:1 d.r.) and enantioselectivity (97% ee) with a low catalyst loading (0.05 mol %). Computational studies were conducted to rationalize the reactivity difference between donor/acceptor carbenes and acceptor carbenes. The utility of the C–H functionalization chemistry was illustrated by its application to the synthesis of (−)-dragocin D and a variety of pharmaceutically relevant pyrrolidines.

## Graphical abstract

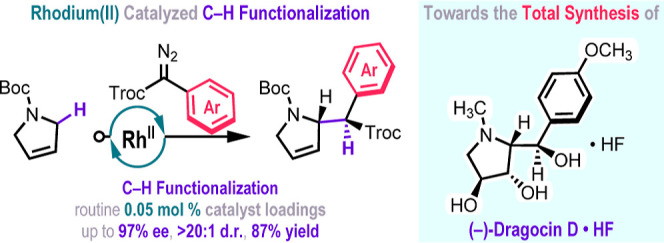

## Introduction

Dirhodium-catalyzed reactions of diazo compounds to generate transient rhodium carbene intermediates have been broadly applied in organic synthesis.^1^ Most of the earlier work in this field was conducted with acceptor and acceptor/acceptor carbene intermediates functionalized with one or two acceptor groups. In the 1980s, the Davies group demonstrated that donor/acceptor carbenes in which the donor group is typically vinyl, aryl, or heteroaryl have a very different reactivity profile to the acceptor and acceptor/acceptor carbenes.^2^ The donor group attenuates the reactivity of the carbene, causing it to exhibit a greater selectivity profile compared to the traditional carbenes functionalized with just acceptor groups.^3^ Furthermore, many chiral dirhodium tetracarboxylates are capable of highly enantioselective reactions with this class of carbenes.^4^ These rhodium carbenes have been applied to a variety of enantioselective intermolecular transformations such as cyclopropanation,^5^ cyclopropenation,^6^ and ylide formation^7^ and have been shown to be especially effective at catalyst-controlled C–H functionalization.^1f,3b,4b,8^ Consequently, they have been applied in strategic reactions for total synthesis and the synthesis of pharmaceutically relevant targets.^1b,9^

The impetus for this project came from our studies on the development of a practical cyclopropanation of *N*-Boc-2,5-dihydro-1*H*-pyrrole (**1**) with ethyl diazoacetate (**2**) under low catalysts loading (0.005 mol %) to access stereoselectively either diastereomer of 3-azabicyclo[3.1.0]­hexane-6-carboxylate, *exo*-**4** or *endo*-**4** (Scheme 1A),^10^ which are prevalent in a variety of drugs and drug candidates.^11^

**Scheme 1:**
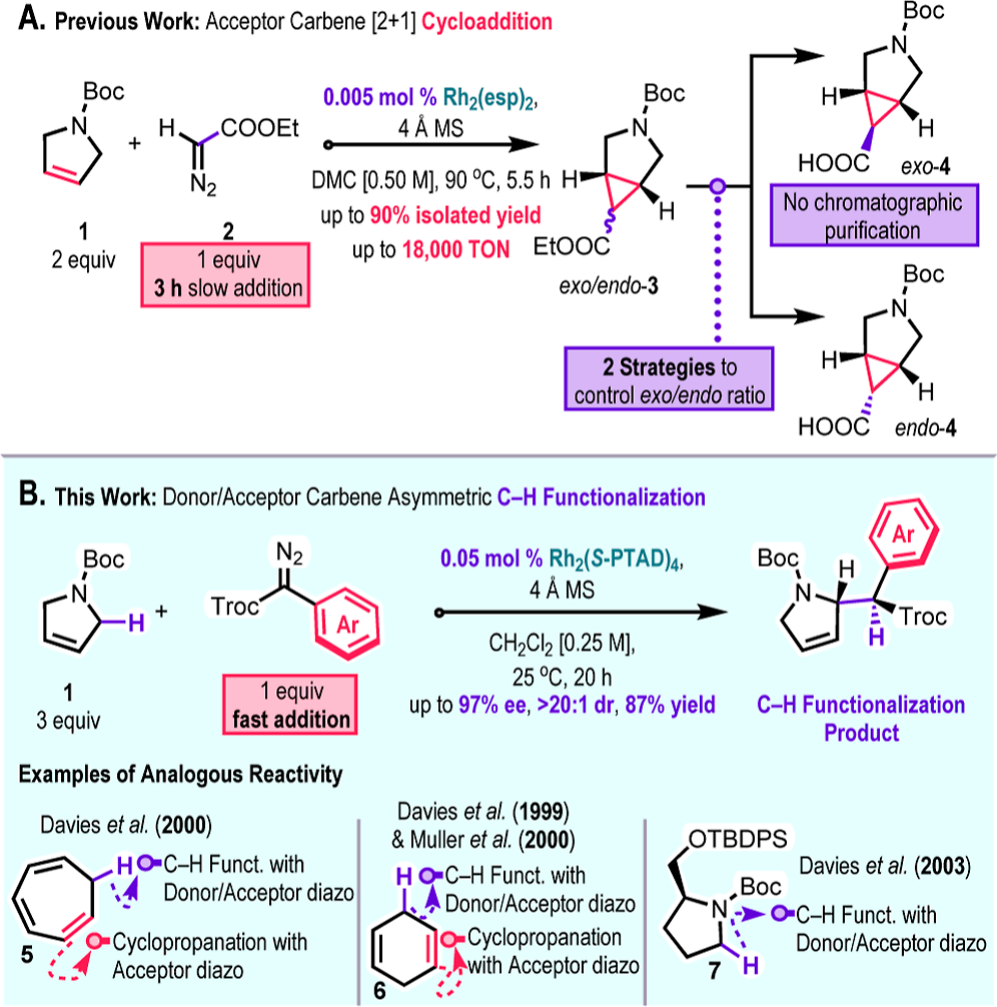
Background and Overview

Having established the cyclopropanation with an acceptor carbene, we became intrigued to explore the reaction of the dihydropyrroles with donor/acceptor carbenes derived from aryldiazoacetates and unexpectedly found it gave an entirely different outcome: The C–H functionalization product was exclusively formed instead of cyclopropanation (Scheme 1B). Even though it is well-established that the reactivity profile of donor/acceptor carbenes is different from acceptor carbenes, especially with regards to enantioselective reactions,^3^ examples of a switch from cyclopropanation to C–H functionalization are rare, having only been observed in hydrocarbons with highly activated C–H bonds such as cycloheptatriene (**5**)^12^ and 1,4-cyclohexadiene (**6**)^13^ (Scheme 1B). Therefore, we decided to conduct an extensive study to explore the scope of the C–H functionalization reaction on dihydropyrroles. This project complements earlier studies that were conducted on saturated pyrrolidines **7**,^14^ especially as the unsaturated functionality greatly increases the synthetic utility of the resulting C–H functionalization products.

The C–H functionalization described herein introduces an aryl acetate functionality in the place of the C–H bond, as illustrated in the core scaffold **8** as shown in Figure 1. The direct introduction of this functionality in a diastereoselective and enantioselective manner is attractive from a pharmaceutical perspective because several lead compounds and biologically active chiral molecules have related structural features as illustrated in **9**–**13**.^11a–11e^ Therefore, we anticipate that the C–H functionalization described herein will be a generally useful method for the stereoselective synthesis of highly functionalized pyrrolidines of pharmaceutical interest.

**Figure 1:**
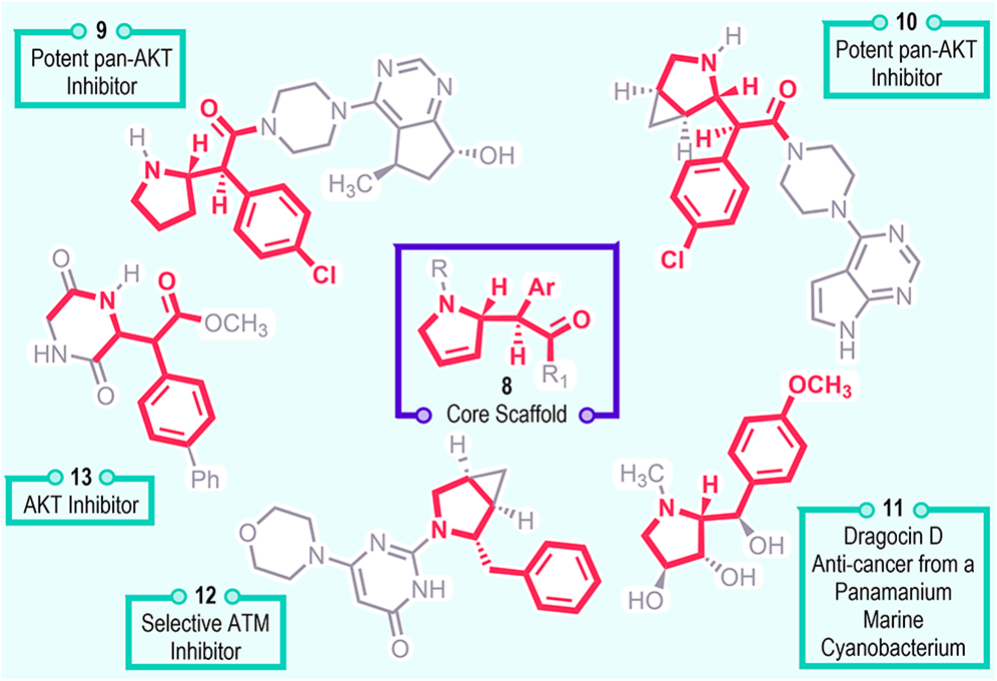
Relevant bioactive compounds with the core scaffold.

## Results and Discussion

The initial evaluation was conducted using dihydropyrrole **1** and trichloroethyl *p*-methoxyphenyldiazoacetate (**14**) in dichloromethane as a solvent in the presence of 4 Å molecular sieves (Table 1). A catalyst screen quickly revealed that the C–H functionalization to form **15** was the preferred reaction because none of the cyclopropanation product was observed in any of the reactions. However, a catalyst screen with 5 equiv of the dihydropyrrole and slow addition of aryldiazoacetate **14** revealed that the stereoselectivity was highly dependent on the catalyst. Many of the chiral catalysts that had previously given high enantioselectivity in C–H functionalization reactions with aryldiazoacetates performed poorly in this system.^4^ The baseline reaction with dirhodium­(II) acetate gave a 10:1 mixture of diastereomers (entry 1). An improved diastereomeric ratio of 19:1 was obtained with Rh_2_(*S*-DOSP)_4_, but the enantioselectivity was very poor (–10% ee, entry 2). This is not surprising because high enantioselectivity with Rh_2_(*S*-DOSP)_4_ is obtained only when the acceptor group is a methyl ester and hydrocarbons are used as solvent.^15^ Previously, Rh_2_(*S*-DOSP)_4_ had resulted in highly diastereoselective reactions with *N*-Boc-pyrrolidine but poor diastereoselectivity with *N*-Boc-piperidine.^14^ Far less expected was the poor asymmetric induction (1–58% ee) exhibited by the chiral catalysts Rh_2_(*S*-*p*-PhTPCP)_4_,^5^ Rh_2_(*S*-*p*-BrTPCP)_4_,^5^ Rh_2_(*S*-2-Cl,5-BrPhTPCP)_4_,^16^ and Rh_2_(*S*-TPPTTL)_4_ ^17^ (entries 3–6), all of which have been shown to be capable of highly enantioselective transformations with aryldiazoacetates. Furthermore, the catalysts had variable effects on the diastereoselectivity of the reaction, from 1:1 d.r. with Rh_2_(*S*-2-Cl,5-BrPhTPCP)_4_ to >20:1 d.r. with Rh_2_(*S*-*p*-PhTPCP)_4_ and Rh_2_(*S*-TPPTTL)_4_. These four catalysts are considered to adopt a fairly rigid structure, and so we reasoned that a more flexible catalyst may be needed to accommodate the C–H functionalization of the dihydropyrrole in a stereodefined way (see Supporting Information Figures S2 and S3). Therefore, we conducted a reaction with Rh_2_(*R*-PTAD)_4_,^18^ a bowl-shaped catalyst that is likely to be more flexible.^19^ Indeed, this catalyst was extremely effective in this reaction, generating **15** in 72% yield, >20:1 d.r., and –96% ee (entry 7). Of course, the opposite enantiomer of **15** is readily obtained by conducting the reaction with Rh_2_(*S*-PTAD)_4_ (entry 8). The initial evaluation was conducted with 5 equiv of trapping agent **1** and slow addition of aryldiazoacetate **14** in order to maximize the yield of the reaction. Typically, an excess of trap generally results in a cleaner reaction, and in this case, the dihydropyrrole **1** can be readily recovered by a short-path distillation prior to chromatographic purification. Slow addition of the diazo compound is often used because it minimizes the generation of side products from carbene dimerization. In this case, however, such stringent reaction conditions were unnecessary because similar yields and enantioselectivities were obtained when just 3 equiv of trapping agent **1** was used and the aryldiazoacetate **14** was added in one portion (entry 9). The standard reaction scale was conducted with 1 mol % of catalyst, but much lower catalyst loading can be used if desired. For example, a similar yield and stereoselectivity was obtained with 0.05 mol % of Rh_2_(*S*-PTAD)_4_ and running the reaction for about 20 h (overnight) instead of 5.5 h (entry 11).

**Table 1:**
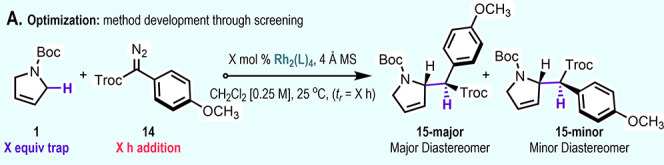

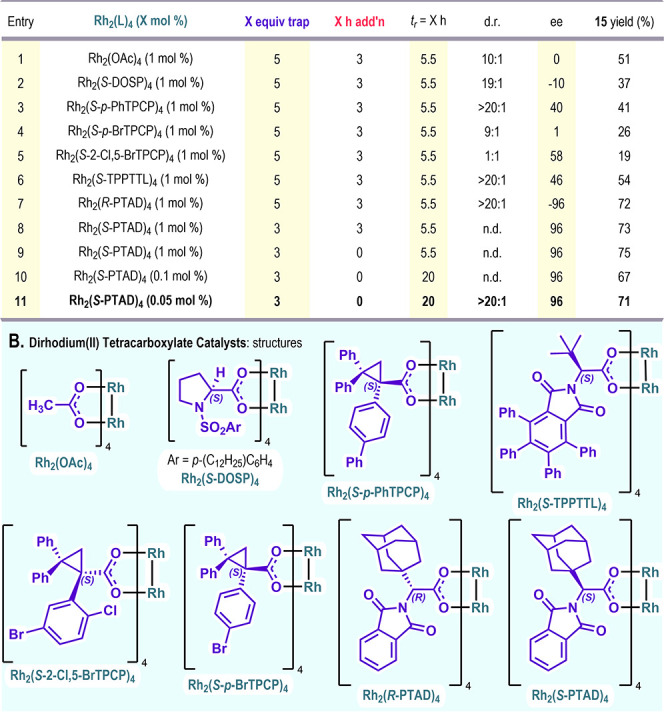
Reaction Optimization of Dirhodium­(II)-Catalyzed C–H Functionalization^a^

a (A) Catalyst screen of different generations of dirhodium­(II) tetracarboxylate catalysts at the 0.500 mmol scale. (B) Structure of dirhodium­(II) tetracarboxylate catalysts utilized in the screen.

The Rh_2_(*S*-PTAD)_4_-catalyzed reaction can be extended to a range of aryldiazoacetates, as illustrated in Scheme 2. In all instances, the products **15**–**26** are formed with high levels of diastereoselectivity (>20:1 d.r.) and, except for one substrate (**24**), the enantioselectivity is ≥90% ee. A wide range of aryldiazoacetates substituted at the meta and para positions worked well, but the reactions were not effective with ortho-substituted aryldiazoacetates. Notable examples are the boronate derivatives **19** and **22**, which are well-suited for further derivatization. Additionally, the use of a diaryl diazoketone as the carbene precursor^17b^ showed comparable enantioselectivity and diastereoselectivity to the aryldiazoacetates, generating **26** in 90% ee and >20:1 d.r. The stereochemical analysis in these reactions is challenging because of the hindered rotation of the *N*-Boc group. The enantioselectivity was determined by SFC, whereas the diastereoselectivity was typically determined on the *N*-Boc-deprotected amines (see Supporting Information Figure S4) or variable-temperature ^1^H NMR studies. X-ray crystallographic data were obtained for the tosylate salts of the amines derived from **15** and **17**, which enabled determination of their relative and absolute configurations. The stereochemical assignments of the other C–H functionalization products are tentatively assigned by analogy.

**Scheme 2:**
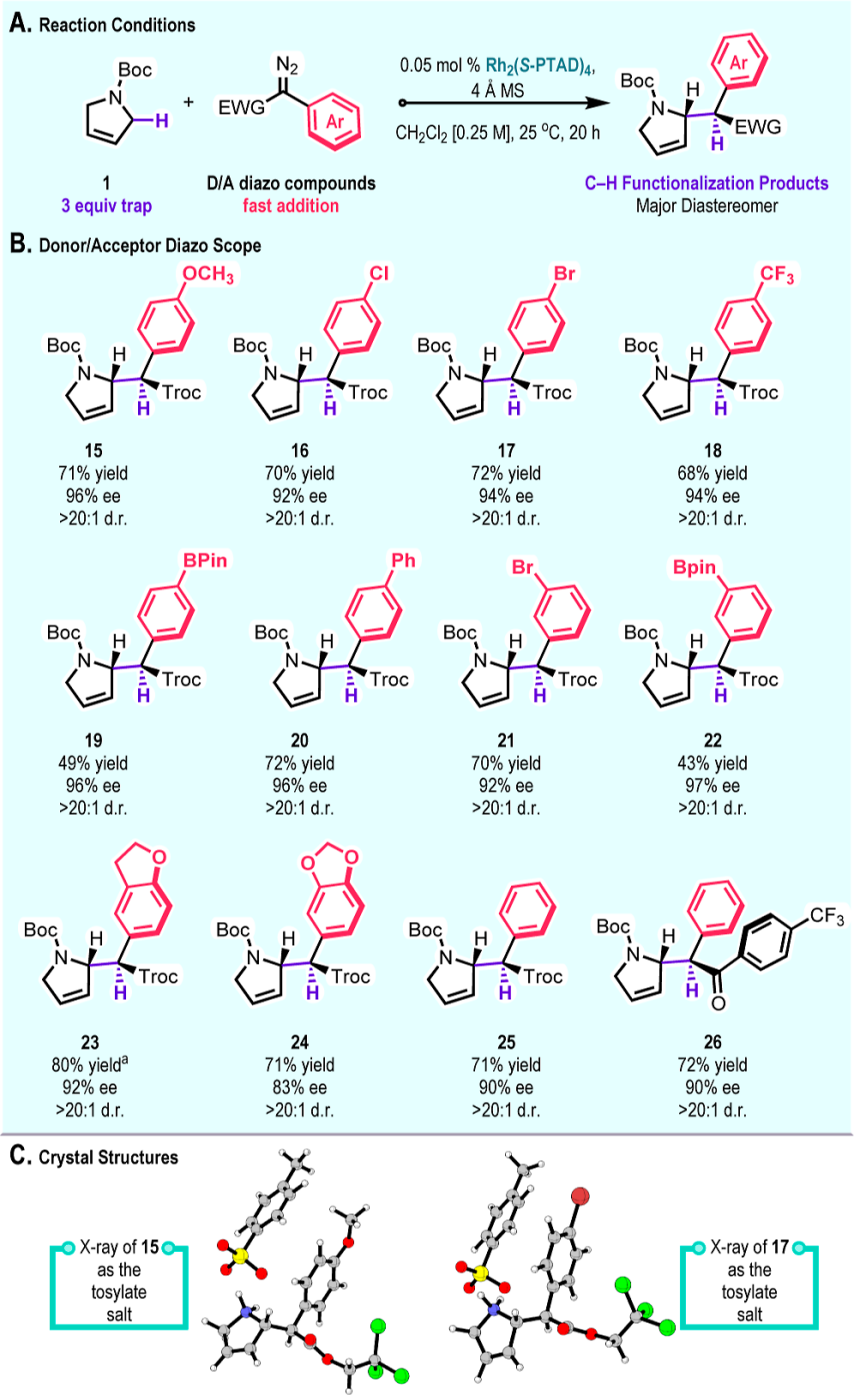
Exploration of a Variety of Donor/Acceptor Carbene Precursors^a^

a Scope of donor/acceptor compounds ran at the 0.5 mmol scale. (A) C–H Functionalization conditions. (B) Scope of diazo compounds. ^a^Required 3 h slow addition instead. C) Crystal structures of tosylate salts derived from (**15**) and (**17**).

To further expand the utility of the C–H functionalization on dihydropyrrole **1**, a double C–H functionalization was performed by switching the stoichiometry of **1** and aryldiazoacetate **27** (Scheme 3). A slow addition of an excess of diazo compound **27** (3 equiv) to a solution of dihydropyrrole **1** as the limiting reagent furnished the *C*_2_-symmetric product **28** with high levels of enantioselectivity and diastereoselectivity (>99% ee, >20:1 d.r.). Differentiated functionality at C2 and C5 can be obtained by conducting a second C–H functionalization on a preformed mono C–H functionalization product with a different aryldiazoacetate as illustrated in the conversion of **17** (94% ee, >20:1 d.r.) with aryldiazoacetate **29** to form **30** (98% ee, >20:1 d.r.). The second C–H functionalization occurs from the opposite face of the dihydropyrrole to the first C–H functionalization and both C–H functionalizations are highly enantioselective. The enantioenrichment during the second C–H functionalization is indicative of a matched/mismatched behavior with the chiral catalyst and the major enantiomer of **17** matched for the second C–H functionalization.

**Scheme 3:**
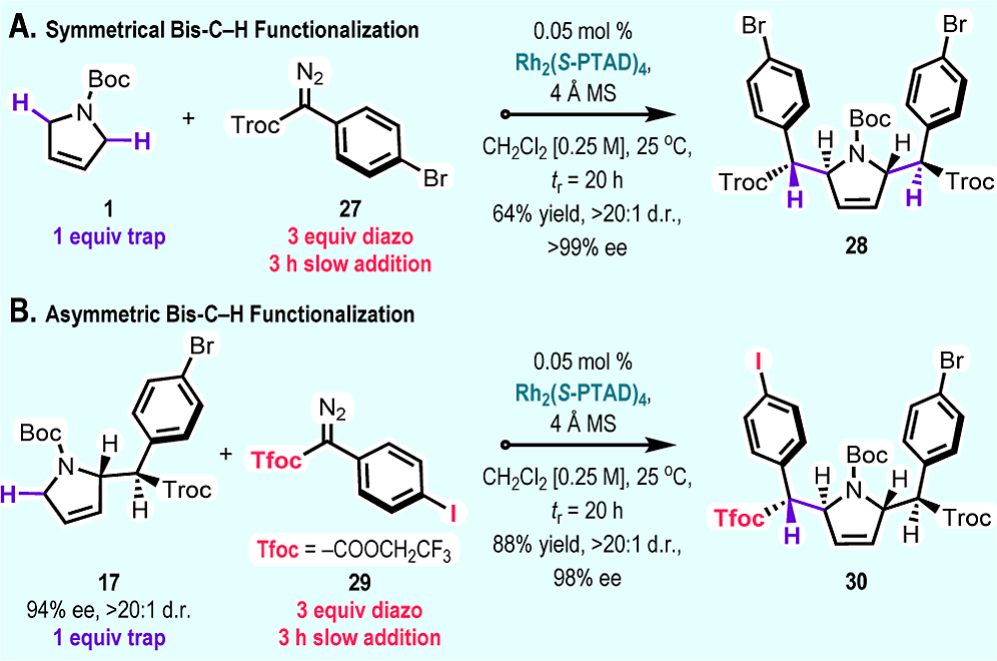
Bis-C–H Functionalization of *N*-Boc-2,5-dihydro-1*H*-pyrrole

The orthogonal functionality in bis-C–H functionalization product **30** is useful for further selective transformations (Scheme 4). This versatility is illustrated by the selective hydrolysis of the trichloroethyl ester (Troc) over the trifluoroethyl ester (Tfoc) using a zinc-mediated reductive elimination of the Troc group to form acid **31** in 54% yield with no epimerization (Scheme 4). A selective Suzuki–Miyaura Coupling (SMC) of the aryl iodide over the aryl bromide is also feasible to form **32** in 55% yield, again with no epimerization. Further functionalization of the carboxylic acid in **31** and the aryl bromide in **32** should also be feasible for further downstream modifications of the scaffold. This scheme is a good illustration of the potential of the carbene-induced C–H functionalization strategy to access complex pharmaceutically relevant scaffolds that would be very difficult to obtain by any other means.

**Scheme 4:**
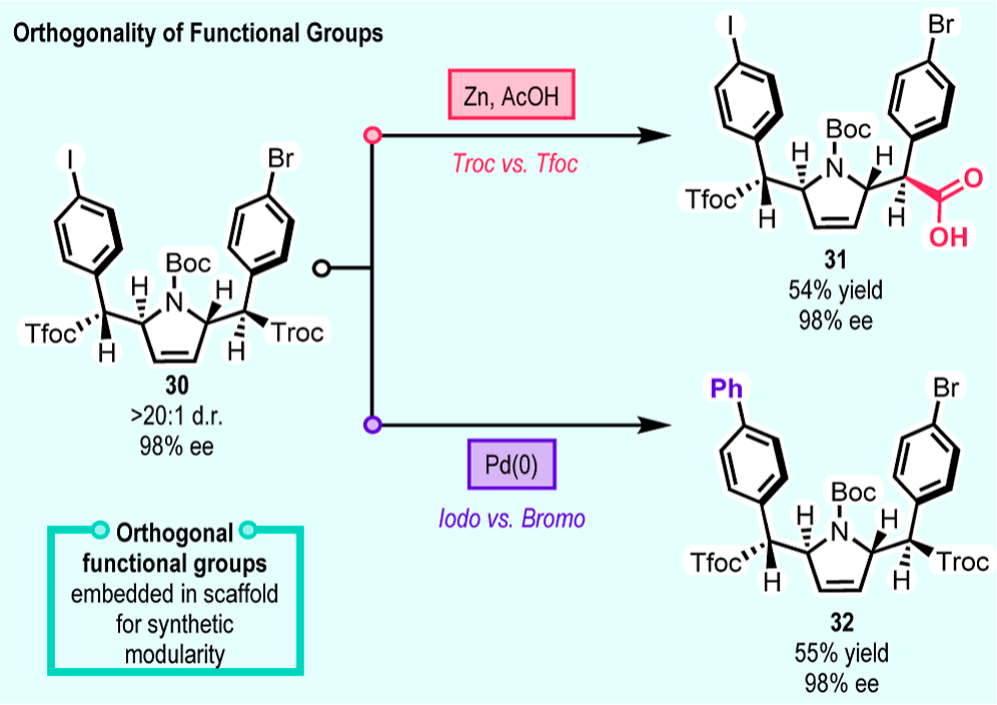
Demonstration of Transformations of Orthogonal Functional Groups^a^

a (A) Demonstration of selective functionalization of orthogonal functional groups from asymmetric bis-C–H functionalization product **30**. Troc deprotection conditions: Zn (10 equiv; <10 μm, dust), AcOH (0.1 M), 25 °C, 4 h. SMC conditions: PhB­(OH)_2_ (1.1 equiv), Cs_2_CO_3_ (2.2 equiv), Pd­(PPh_3_)_4_ (10 mol %), Tol/H_2_O (10:1 v/v), 100 °C, 2 to 4 h.

Using the optimized conditions, we expanded the scope of the C–H functionalization to substituted chiral *N*-Boc-2,5-dihydro-1*H*-pyrroles (Scheme 5). We found that the (*S*)-enantiomer of the trap reacts cleanly and diastereoselectively with the (*S*)-enantiomer of the catalyst. For example, **33** was obtained in 92% yield, >20:1 d.r., and 98% ee in a Rh_2_(*S*-PTAD)_4_-catalyzed reaction with the (*S*)-enantiomer of the trap. Similarly, the opposite enantiomer **34** was obtained using the (*R*)-enantiomer of the trap with Rh_2_(*R*-PTAD)_4_. An effective reaction was also obtained with (*R*)-TIPS-protected alcohol using Rh_2_(*R*-PTAD)_4_ as the catalyst to form **35** in 67% yield, >20:1 d.r., and >99% ee. When the mismatched reactions were attempted, a complex mixture of products and unreacted trap were obtained. A model for the stereochemical outcome is shown in Scheme 5C.^14^ Rh_2_(*S*-PTAD)_4_ shows a strong preference for functionalization at the pro-*S* C–H bond. When the C2 position has an *S*-configuration, the pro-*S* C–H bond at the C5 position is trans to the substituent at C2 and is readily functionalized. Rh_2_(*R*-PTAD)_4_ would preferentially react with the pro-*R* C–H bond, but this is located *cis* to the C2 substituent and would be sterically more challenging.

**Scheme 5:**
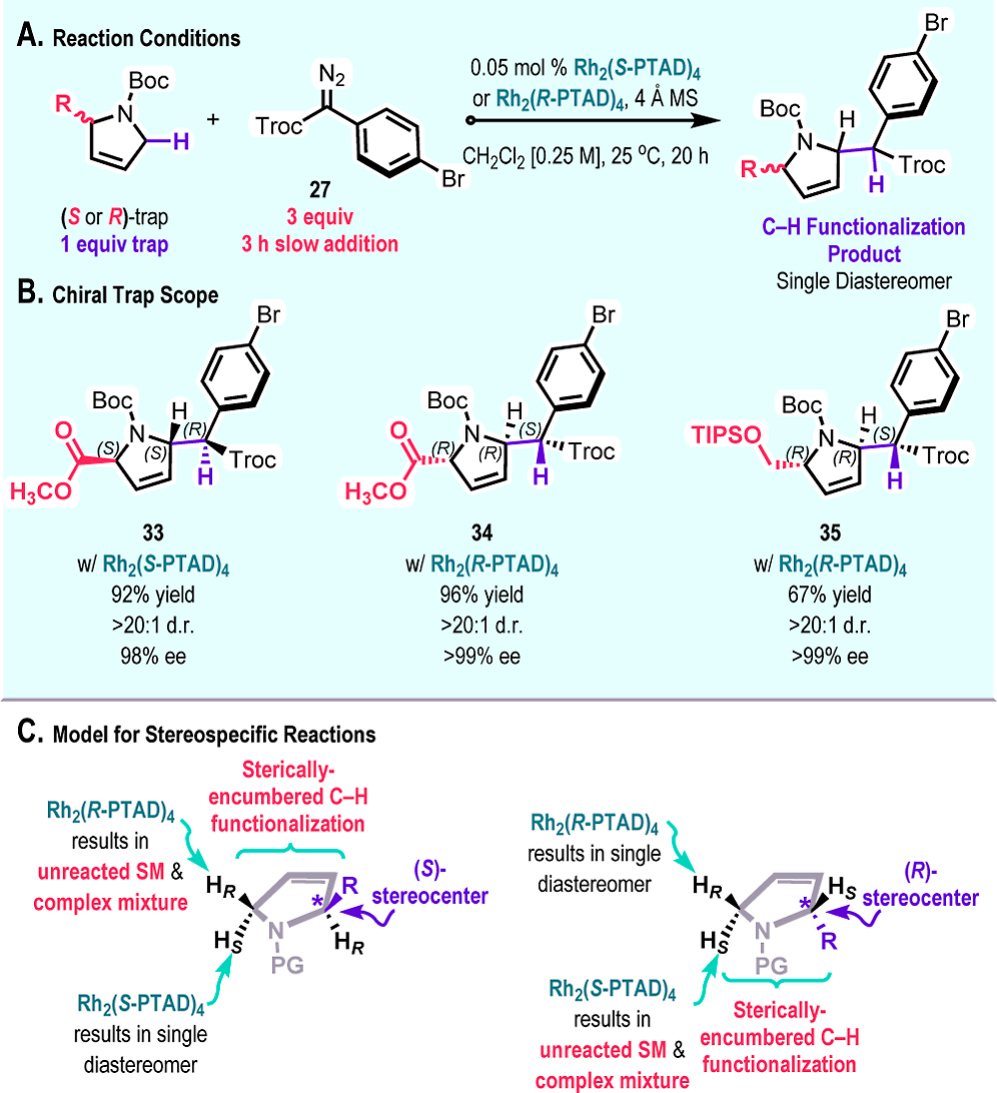
Stereospecific Reactions with Chiral Traps^a^

a Stereospecific reactions with chiral traps ran at the 0.5 mmol scale. (A) Conditions for C–H functionalization. (B) Chiral trap scope. (C) Model for stereospecific reactions.

Having established previously that ethyl diazoacetate undergoes cyclopropanation of the unsubstituted *N*-Boc-2,5-dihydro-1*H*-pyrrole (**1**) to generate meso products,^10^ we were intrigued to determine whether it would still cyclopropanate the mono-C–H functionalization products. This would make it possible to generate 3-azabicyclo[3.1.0]­hexane-6-carboxylates with five stereogenic centers by means of two sequential carbene reactions (Scheme 6). In order to fully consume the C–H functionalization product **17**, 5 equiv of ethyl diazoacetate (**2**) and 1 mol % of the catalyst Rh_2_(esp)_2_ ^20^ were required. Under these conditions, a 75% yield was obtained of cyclopropanes *exo*-**36** and *endo*-**36** as a ∼1:1 mixture. The cyclopropanation occurs from the opposite face of the C2-substituent and the *exo*:*endo* isomers are readily separated. The low diastereoselectivity is common for cyclopropanations with acceptor carbenes. The *exo*-isomer (*exo*-**36**) and *endo*-isomer (*endo*-**36**) were both obtained in 94% ee with retention of stereochemistry.

**Scheme 6:**
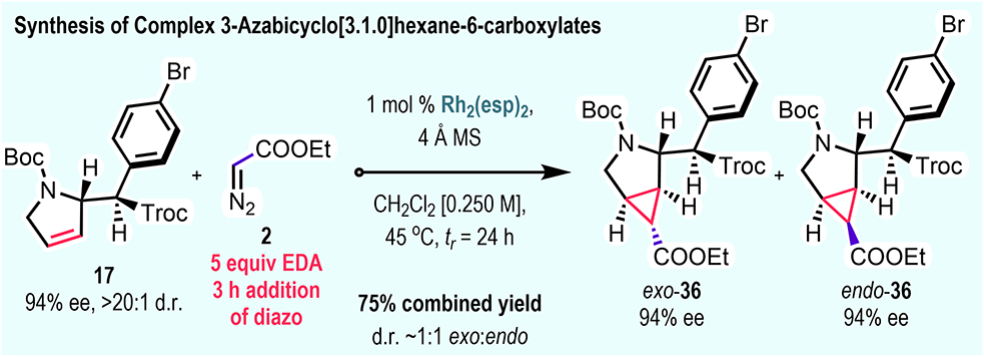
Synthesis of Complex 3-Azabicyclo[3.1.0]­hexane-6-carboxylates via Rh­(II)-Catalyzed [2 + 1] Cycloaddition

In order to further demonstrate the utility of this chemistry, the C–H functionalization was conducted on a larger scale and the further derivatization of the product was illustrated (Scheme 7). The reaction to form **17**, conducted at a 30 mmol scale, gave virtually identical results to those of the small-scale reaction. *N*-Boc deprotection of **17** using traditional acidic conditions (TFA or *p*TSA) followed by neutralization led to decomposition because the amine is prone to do a retro-Mannich reaction, driven by the rearomatization of the iminium intermediate to a pyrrole (see Supporting Information Figures S5 and S6). However, the amine can be isolated as its tosylate salt, and X-ray crystallographic data can be obtained, as described in Scheme 2C. Nitrogen deprotection can be readily conducted if the ester group is first reduced (Scheme 7). Reduction of the Troc group on **17** with lithium borohydride generated the alcohol **37** in 82% yield which, on *N*-Boc deprotection, furnished the stable free amine **38** in 81% yield. Furthermore, we demonstrated the functionalization of the olefin moiety by osmium tetroxide-catalyzed dihydroxylation to generate diol **39** as a single diastereomer in a 77% yield. Reduction of **39** with lithium borohydride generated triol **40** in 86% yield. All of these reactions proceeded without any epimerization.

**Scheme 7:**
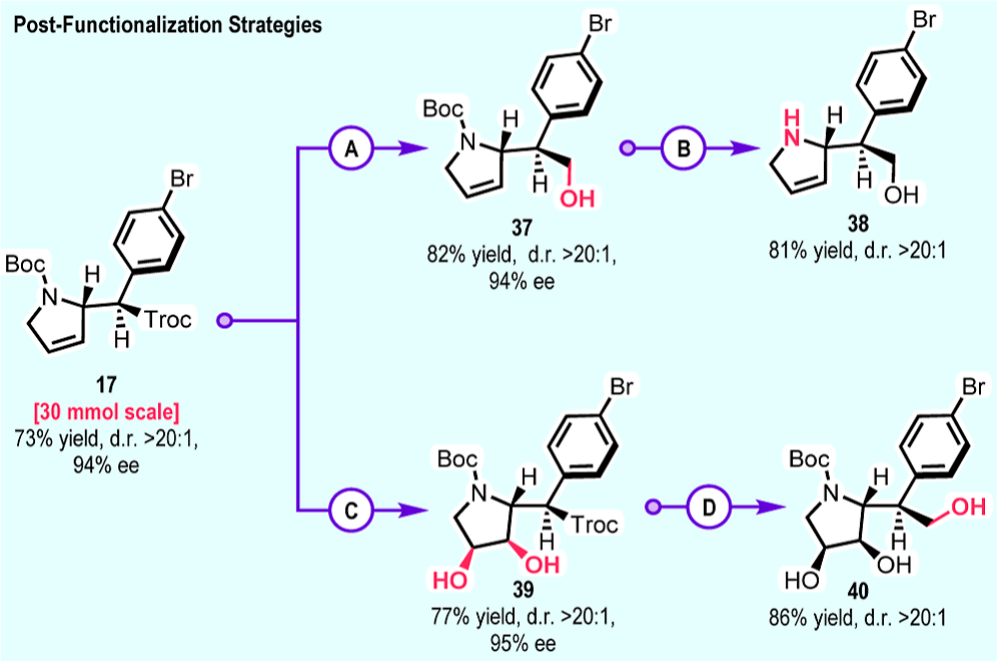
Postfunctionalization of Mono-C–H Functionalization Products^a^

a Synthetic transformations of **17**. (A) LiBH_4_ (2 equiv), MeOH (0.5 equiv), THF, 25 °C, 2.5 h; (B) TFA (10 equiv), CH_2_Cl_2_, 25 °C, 20 h; (C) OsO_4_, NMO, THF, 25 °C, 20 h; (D) LiBH_4_ (6 equiv), MeOH (0.5 equiv), THF, 25 °C, 2.5 h.

Having established the C–H functionalization reactions and illustrated the possibilities for further functionalization of the products, we decided to demonstrate the general utility of this methodology by its application to the stereoselective synthesis of (−)-dragocin D (**11**). (−)-Dragocin D (**11**) is a recently isolated dihydroxylated pyrrolidine natural product from a marine cyanobacterium.^11c^ It displays anticancer activity, but this could not be fully evaluated because of limited supply from the natural source. It has not been previously synthesized, but related members of this family have been synthesized.^21^ A major unsolved challenge in the previous syntheses is the effective control of the relative stereochemistry in natural products.

The synthetic approach to (−)-dragocin D (**11**) is illustrated in Scheme 8. The key C–H functionalization of the dihydropyrrole **1** with aryldiazoacetate **14** was conducted on a 100 mmol scale using Rh_2_(*S*-PTAD)_4_ (0.05 mol %) to form **15** in 87% yield, >20:1 d.r., and 96% ee as the first key C–C bond formation. Higher yields are typically obtained when these reactions are conducted on a larger scale (see Supporting Information page S38) presumably because the evolved nitrogen protects the systems from side reactions caused by either oxygen or water.^10^ The C–H functionalization product **15** was then epoxidized with *m*CPBA followed by zinc-induced Troc deprotection and epoxide ring opening to form γ-butyrolactone **41** in 73% yield over two steps. The γ-lactonization is highly diastereoselective because the epoxidation occurs from the opposite face to the C2-substituent in **15** and the epoxide ring opening is in an *anti*-relationship. The hydrolysis of the lactone in **41** followed by silylation of the alcohol to form the acid **44** was not favorable (see Supporting Information Figure S11), and so, an indirect method had to be developed to form **44**. First, lithium borohydride reduction of the γ-butyrolactone **41** followed by global silylation with TBDMSCl generated the trisilylated derivative **42** in 61% yield over 2 steps. Selective hydrolysis of the primary siloxy group^22^ in **42** generated the alcohol **43** in 78% yield, which was then oxidized to the acid **44** in 91% yield by a sequential TEMPO and Pinnick oxidations. A photoinduced decarboxylative carbamylation on **44** generated the bicyclic derivative **45** with excellent diastereocontrol (20:1 d.r.). We initially expected the reaction to perform an intermolecular acetoxylation^23^ in the presence of the excess Cu­(OAc)_2_, but since the substrate contains a *N*-Boc group, the intermediate carbocation is intramolecularly trapped by the *N*-Boc group to form the carbamate **45**. We attribute the high diastereoselectivity to the bulky 2° siloxy group at C3 that is in the same concave face of the carbamate, resulting in the *p*-methoxyphenyl group orienting itself in the convex face of the bicyclic system. DIBAL-H reduction of carbamate **45** generated *N*-methyl alcohol **46**. Desilylation with tetrabutylammonium fluoride of **46** was effective, but the removal of tetrabutyl ammonium salts from the desired product proved to be challenging (see Supporting Information Figure S11). However, an alternative approach developed for desilylation of substrates containing acid-sensitive and nitrogen functionalities using ammonium fluoride in methanol^24^ successfully generated (−)-6-*epi*-dragocin D•HF (**49**) as the hydrofluoride salt in 77% isolated yield (90% qNMR yield from crude). In order to obtain (−)-dragocin D•HF (**48**), we needed to invert the configuration of the benzylic carbon of alcohol **46**. This was achieved by oxidation of the alcohol to the ketone followed by sodium borohydride reduction (15:1 dr) to selectively form the epimeric derivative **47** in 60% yield for the two steps. Desilylation with ammonium fluoride generated the hydrofluoride salt of (−)-dragocin D (**48**) in 72% isolated yield (86% qNMR yield from crude). Attempts to generate the neutral amine of **48** and **49** proved to be difficult, but the compounds are stable as the hydrofluoride salt.

**Scheme 8:**
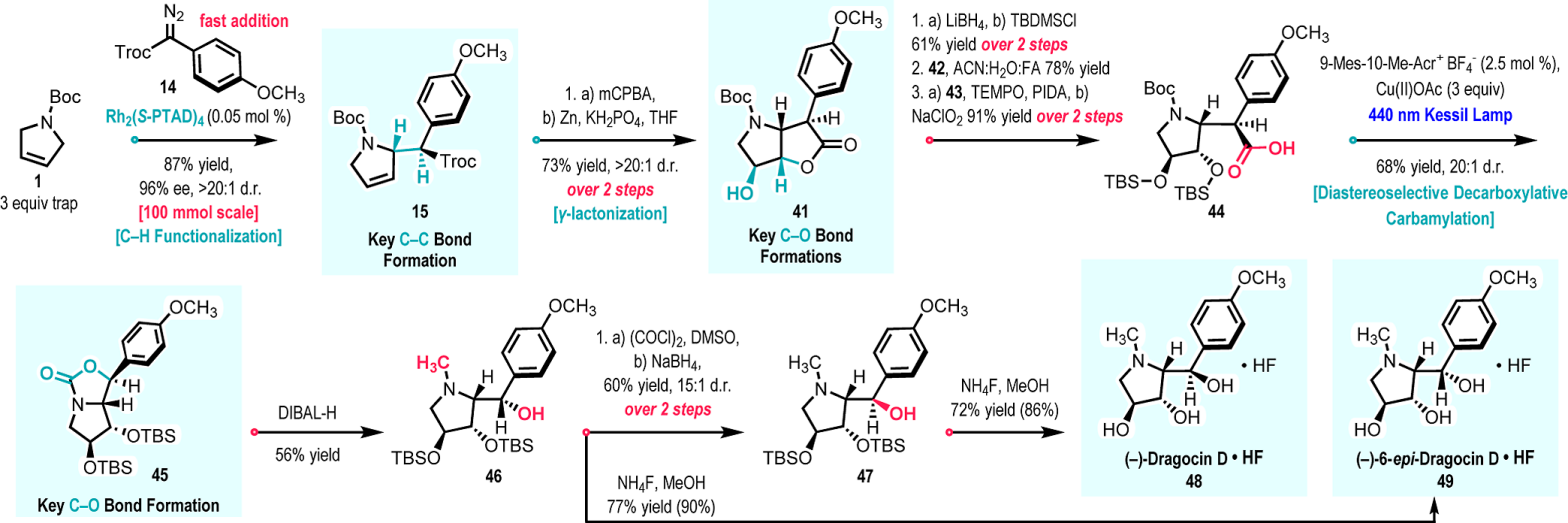
Total Synthesis of (−)-Dragocin D•HF (**48**) and (−)-6-*epi*-Dragocin D•HF (**49**)

Density functional theory (DFT) studies were conducted to help understand why the acceptor carbene prefers cyclopropanation of the dihydropyrrole, whereas the donor/acceptor carbene prefers C–H functionalization. As these outcomes are catalyst-independent, the calculations were carried out using rhodium acetate as the catalyst. Previous computational studies have shown that the rhodium-bound donor/acceptor carbene is far more stable than the rhodium-bound acceptor carbene, even though it is still a high energy intermediate and displays far greater selectivity than the rhodium-bound acceptor carbene.^25^ The calculations with trichloroethyl phenyldiazoacetate revealed that the donor/acceptor carbene has a very favorable concerted asynchronous pathway for C–H functionalization at C2 of the dihydropyrrole **1** with a free energy barrier of 1.3 kcal/mol (**TS1**) (Figure 2A). The free energy barrier for cyclopropanation is considerably higher (4.2 kcal/mol, **TS2**), and thus, the observed C–H functionalization is indeed the expected product.

**Figure 2:**
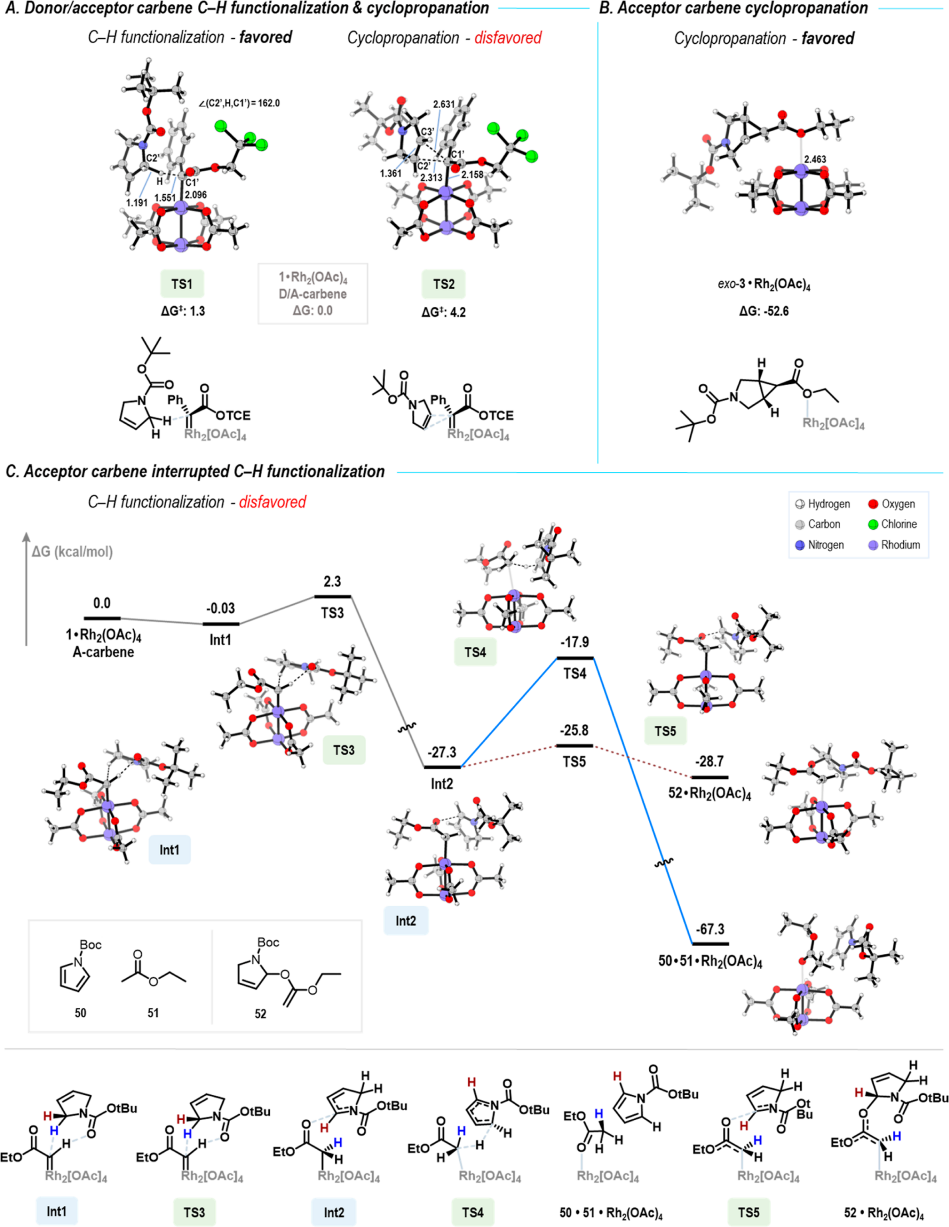
Computational studies on the reaction of rhodium carbenes with *N*-Boc-2,5-dihydro-1*H*-pyrrole. (A) Energetically favorable transition states for the C–H functionalization and cyclopropanation of the donor/acceptor carbene. Additional transition state conformations are available in Supporting Information Section 11. (B) Cyclopropane product from the reaction with an acceptor carbene. (C) Reaction pathways of interrupted C–H functionalization reactions beginning with a hydride transfer to the acceptor carbene. All Gibbs free energies are set relative to the respective substrate•rhodium carbene complexes (kcal/mol).

The computational analysis of the reaction of dihydropyrrole **1** with ethyl diazoacetate (**2**) was more nuanced than we had originally expected. The cyclopropanation with ethyl diazoacetate (**2**) is essentially barrierless, and thus, we consistently could not locate any transition state and instead arrived directly at the product *exo*-**3•Rh**_**2**_ **(OAc)**_**4**_, a reaction which is exergonic by −52.6 kcal/mol (Figure 2B). These results are consistent with previous computational studies of cyclopropanation with ethyl diazoacetate (**2**), which showed the conversion from the carbene to the cyclopropane was essentially barrierless.^25,26^

In contrast, our computational studies revealed that C–H functionalization with ethyl diazoacetate (**2**) is not a viable pathway (Figure 2C). It begins with weakly bound complex **Int1** (involving interactions between the CO of the Boc group and the C–H bond of the carbene), which undergoes a hydride atom transfer (with a 2.3 kcal/mol free energy barrier, **TS3**) to form **Int2**. **Int2** involves a resonance-stabilized carbocation and a rhodium-bound enolate as a consequence of the hydride atom transfer. Interestingly, the two fragments of this intermediate do not have a favorable pathway to combine to form the C–H functionalization product. Instead, the rhodium-bound enolate and the carbocation undergo proton transfer via **TS4** to form *N*-Boc-pyrrole (**50**) and ethyl acetate (**51**). This process requires overcoming a 9.4 kcal/mol free energy barrier and is exergonic by −40.0 kcal/mol. Considerable efforts were made to find a C–H functionalization pathway, but this could not be achieved. It always proceeded through the hydride abstraction pathway.

Alternatively, the rhodium-bound enolate **Int2** may proceed via **TS5** to form (**52**), a process that has a low barrier (1.5 kcal/mol) and is reversible. Thus, in contrast to the donor/acceptor carbenes, cyclopropanation is preferred for the reaction with ethyl diazoacetate (**2**), and the energetics are less favorable for the C–H functionalization pathways. These calculations provide an explanation for why acceptor carbenes undergo C–H functionalization reactions with a very limited range of substrates compared to the donor/acceptor carbenes, even though acceptor carbenes are inherently more reactive. The donor/acceptor carbenes undergo a concerted asynchronous C–H functionalization with a variety of substrates, but the C–H functionalization reactions with acceptor carbenes are capable of a hydride transfer when the C–H bond is able to stabilize a positive charge. The resulting cationic and anionic fragments can engage in other transformations rather than just C–H functionalization (Figure 2C).

## Conclusion

In conclusion, these studies demonstrate that the Rh_2_(*S*- or *R*-PTAD)_4_-catalyzed C–H functionalization of dihydropyrroles with aryldiazoacetates is a versatile reaction, proceeding in high yield and with excellent diastereo- and enantioselectivity. The resulting products have potential as versatile synthetic intermediates and can be readily converted to pharmaceutically relevant pyrrolidine derivatives. This potential was illustrated by its application toward the first and most efficient stereoselective synthesis to date of (−)-dragocin D•HF (**48**), the hydrofluoride salt of (−)-dragocin D (**11**), and (−)-6-*epi*-dragocin D•HF (**49**). These studies also illustrate the considerable advantages of using donor/acceptor carbenes versus acceptor carbenes in C–H functionalization reactions. Computational studies revealed that donor/acceptor carbenes are capable of smoothly undergoing a concerted asynchronous C–H functionalization with dihydropyrrole **1**, whereas the acceptor carbene preferentially undergoes cyclopropanation. However, in the acceptor carbene C–H functionalization-related pathway, hydride transfer dominates which then proceeds to side product formation rather than a productive C–H functionalization.

## Supplementary Material



## Notes

Complete experimental procedures, materials, computational details, and compound characterizations and crystal structure for compounds **15** and **17** (PDF)

## Abbreviations

d.r.: diastereomeric ratio
ee: enantiomeric excess
SFC: supercritical fluid chromatography
Tfoc: trifluoroethoxycarbonyl
Troc: trichloroethoxycarbonyl
n.d.: not determined
EDA: ethyl diazoacetate
DFT: density functional theory
*N*-Boc: *N*-*tert*-butyloxycarbonyl
SMC: Suzuki–Miyaura Coupling
TIPS: triisopropylsilyl
TFA: trifluoroacetic acid
*p*TSA: *p*-toluenesulfonic acid
SM: starting material
