# Supplementary material for: Asymmetric C–H Functionalization of N‑Boc-2,5-dihydro‑1H‑pyrrole and Its Application as a Key Step in the Synthesis of (−)-Dragocin D
Source: J Am Chem Soc. 2025 Jul 28;147(31):28098–106. doi: 10.1021/jacs.5c08080 (PMC12333343; doi:10.1021/jacs.5c08080)
Supplement: Supplementary file 1 [file ja5c08080_si_001.pdf]

Asymmetric C–H Functionalization of *N*-Boc-2,5-Dihydro-1*H*-Pyrrole and its Application as a Key Step in the Synthesis of (–)-Dragocin D

Terrence-Thang H. Nguyen[1], Kristin Shimabukuro[1], Djamaladdin G. Musaev[2], John Bacsa[1], Antonio Navarro[3], Huw M. L. Davies[1]*

[1]Department of Chemistry, Emory University, Atlanta, Georgia 30322, United States
[2]Cherry L. Emerson Center for Scientific Computation, Emory University, 1521 Dickey Drive, Atlanta, Georgia 30322, United States
[3]Lilly Research Laboratories, Eli Lilly and Company, Indianapolis, Indiana 46285, United States

Corresponding author email: hmdavie@emory.edu

Supporting Information

Complete experimental procedures, materials, computational details, and compound characterizations

Table of Contents

1. General Considerations 1
2. Reagent Acquisition 4
   i. Reagents Acquired Through Vendors: 4
   ii. Reagents Acquired Through Known Procedures: 6
3. Catalyst Structures and Structure Activity Relationship (SAR) Study 6
4. Determination of d.r. by *N*-Boc deprotection and/or Variable Temperature ^{1}H NMR 10
5. Observation of Retro-Mannich Reaction Through *N*-Boc Deprotection Followed by Free-Base 11
6. Characterization of Catalysts and Starting Materials 12
7. Characterization of C–H Functionalization Products 38
8. Route Scouting for the Synthesis of (–)-6-*epi*-Dragocin D • HF and (–)-Dragocin D • HF 163
9. Comparison of NMR data between Natural (–)-Dragocin D and Synthetic (–)-Dragocin D • HF 164
10. Crystal Structure and Experimental 165
11. DFT Calculations of C–H Functionalization & Cyclopropanation 185
12. References 214

1. General Considerations

Warning: This project involves the use of diazo compounds. Diazo compounds are known to have thermal stability issues and are explosive hazards; work with diazo compounds should be performed in a well-ventilated hood, require the use of PPE, and careful handling of the reagents.[1] Any excess diazo or azide reagent was quenched by treating it with O_3 using an ozone generator.

Absolute Configurations

The absolute configuration is determined based on X-ray crystal structures. The stereochemical assignment of C–H functionalization products that do not have an X-ray crystal structure is tentatively assigned by analogy of X-ray crystal structures **15** and **17**.

Reaction Setup, Workup, and Purification

Schlenk-line setup was connected to an Edwards RV12 rotary vane vacuum pump and used to manipulate samples to perform inert atmosphere techniques. Reactions were carried out under nitrogen in flame-dried vessels unless otherwise specified. Dichloromethane, diethyl ether, tetrahydrofuran, and toluene were purified using a *Glass Contour Solvent Purification System* (SPS). Dichloromethane used for C–H functionalization reactions was distilled from calcium hydride onto activated 4Å molecular sieves (beads), the distilled solution sparged with nitrogen for at least 15 minutes and stored under nitrogen overnight prior to use. 4Å molecular sieves (beads) were activated by placing the molecular sieves into a flame-dried round-bottom flask attached with a vacuum adapter, the vessel containing the molecular sieves were heated to 260 - 300 ^{o}C overnight under vacuum. Once dried and activated, the molecular sieves were stored into an oven set at around 150 ^{o}C and used as is. Generally, for C–H functionalization reactions, the hot activated 4Å MS is quickly weighed (1 mmol of limiting reagent / 1 g of activated 4Å MS) into the vial charged with a stir-bar and flame-dried under vacuum. Concentrating *in vacuo* at a set temperature refers to using a Heidolph Hei-Vap rotary evaporator to evaporate off solvent. Flash column chromatography was performed on Biotage Isolera systems with Biotage Sfär DLV empty columns that are filled with Silicycle SiliaFlash P60 silica gel (60 Å pore size, 40–63 µm particle size, 230–400 mesh) and ACS reagent grade solvents. Biotage Isolera settings are set as SNAP KP-Sil 100g unless otherwise specified. A "Running-Column" refers to the column that contains pure SiO_2, undergoes equilibration by the instrument, and is where the reaction crude undergoes the chromatographic separation. A "Loading-Column" refers to the column that contains the SiO_2 dry-load that has the crude reaction material adsorbed. The "Loading-Column" is positioned on top of the "Running-Column" after equilibration**.** General Biotage runs are set up as step-gradients unless otherwise specified. Reactions were monitored by thin layer chromatography (TLC) carried out on with aluminum-sheet or glass-backed silica gel plates, visualizing with UV light, and staining with various stains. Room temperature is approximately 25 ^{o}C. Syringe pumps were obtained from KD Scientific. Syringe needles were obtained from Air-Tite or Silver-Point. Generally, for C–H functionalization reactions requiring slow addition of diazo compounds, the total volume is partitioned in half: half volume used to dissolve catalyst with trap, and the other half used to dissolve diazo compounds.

Resolving Rotamers

Rotamers are resolved by either *N*-Boc deprotection (*p*TSA or TFA) and analyzed as the ammonium salt or variable temperature ^{1}H NMR of the intact *N*-Boc product to determine d.r.. The deprotected products were not further characterized other than as a means of determining d.r.. Variable temperature NMR was performed on Varian-400; the instrument temperature setting can only go up to 75 – 80 ^{o}C in our facilities.

NMR Spectroscopy and Processing

All ^{1}H NMR spectra were recorded at either 400 MHz, 500 MHz, 600 MHz, or 800 MHz on Varian/Bruker-400, Varian-500, or Varian/Bruker-600/800 spectrometers. ^{13}C NMR spectra were recorded at either 101 MHz, 126 MHz, 151 MHz, or 201 MHz on Varian/Bruker-400, Varian-500, Bruker 600/800 spectrometers. ^{19}F NMR spectra were recorded at 376, 565, or 753 MHz on Varian/Bruker-400 or Varian/Bruker-600/800 spectrometer and pulse sequences are either ^{1}H-decoupled ^{19}F NMR or ^{1}H-coupled ^{19}F NMR. 2-D NMR spectra were recorded on 400 MHz, 600 MHz, or 800 MHz on Varian/Bruker-400 or Varian/Bruker-600/800 spectrometers. Deuterated chloroform ($CDCl_3$ or Chloroform-*d*) was neutralized with barium carbonate and filtered before use when acquiring NMR spectra. NMR spectra were obtained from solutions of deuterated chloroform ($CDCl_3$ or Chloroform-*d*) with or without 0.03% TMS with residual solvent serving as internal standard (7.26 ppm for ^{1}H or 0.00 ppm and 77.16 ppm for ^{13}C) or deuterated

DMSO (DMSO-d_6) (2.50 ppm for ^{1}H and 39.52 ppm for ^{13}C) or deuterated methanol (MeOD or Methanol-d_4) (3.30 ppm for ^{1}H and 49.0 ppm for ^{13}C). NMR shifts were reported in parts per million (δ ppm). Abbreviations for signal multiplicity are as follows: s = singlet, d = doublet, t = triplet, q = quartet, m = multiplet, brs = broad singlet, dd = doublet of doublet, etc. Coupling constants (J values) were calculated directly from the spectra. NMR processing was performed using MestReNova and all spectra were subjected to "Auto Phase Correction" using the "Automatic" setting and "Auto Baseline Correction" using the "Baseline Correction" followed by Polynomial Fit and Autodetect Filter enabled. All reagents were purchased commercially and used without further purification unless otherwise noted.

Chiral Resolution and Instrumentation

Racemic standards were generated by performing reactions with Rh_2(R/S-PTAD)$_4$, which was generated by dissolving an equimolar mixture of the *R*- and *S*-catalyst in a minimal amount of dichloromethane and concentrating *in vacuo*. The enantiomeric excess (ee) was determined by 1) Supercritical Fluid Chromatography using Waters Acquity UPC^2 SFC system and the data outlined below is processed through Waters MassLynx software or 2) High Performance Liquid Chromatography using an Agilent 1100 HPLC system and the data outlined below is processed through Agilent's software.

SFC traces are reported based on the racemic retention times. The SFC system utilized supercritical fluid CO_2 with cosolvents of either HPLC-grade methanol, or acetonitrile, or ethanol, or isopropanol, or 1:1 MeOH:IPA with 0.2% formic acid, or 1:1 Ethanol:IPA with 0.2% formic acid, or 1:1 Ethanol:ACN with 0.2% formic acid, or 1:1:1 Ethanol:IPA:ACN with 20 mM ammonium formate with SFC columns: Trefoil AMY1 Column (2.5 µm, 3.0 mm X 150 mm), Trefoil CEL1 Column (2.5 µm, 3.0 mm X 150 mm), Trefoil CEL2 Column (2.5 µm, 3.0 mm X 150 mm), Regis (S,S) Whelk-O 1 Kromasil (3.5 µm, 3.0 mm X 150 mm), ChiralPak AD-3 (3.0 µm, 3.0 mm X 150 mm SFC), ChiralCel OZ-3 (3.0 µm, 3.0 mm X 150 mm), ChiralCel OD-3 (3.0 µm, 3.0 mm X 150 mm SFC), ChiralCel OX-3 (3.0 µm, 3.0 mm X 150 mm SFC); ChiralCel OJ-3 (3.0 µm, 3.0 mm X 150 mm SFC); ChiralPak AS-3 (3.0 µm, 3.0 mm X 150 mm SFC). The default pressure for SFC runs is 2000 psi unless otherwise specified.

HPLC traces are reported based on the racemic retention times. The Agilent 1100 HPLC system used isocratic IPA/*n*-hexane solvent system and commercial ChiralPak AD-H (5 µm particle size, 4.6 mm vs. 250 mm), ChiralCel OD-H (5 µm particle size, 4.6 mm vs. 250 mm), and Regis (S,S) Whelk O1 5/100 Kromasil.

Optical Rotation

Optical rotations were measured on Jasco P-2000 polarimeters and on Rudolph Research Analytical Automatic Polarimeter APIV-1W using the Scale: Specific Rotation setting (wavelength 589nm, cell length 100mm, Quartz temperature correction, and multiples of 5s).

Mass Spectroscopy

Mass spectra were taken on a Thermo Finnigan LTQ-FTMS spectrometer with APCI, ESI or NSI.

Abbreviations

RBF = round-bottom flask
o-NBSA = *o*-nitrobenzenesulfonyl azide
DBU = 1,8-Diazabicyclo[5.4.0]undec-7-ene
DCC = N,N'-Dicyclohexylcarbodiimide
TLC = Thin-Layer Chromatography
SM = Starting Material
POI = Product of Interest
SPS = Solvent Purification System
Ether = diethyl ether
Rf = retention factor

DI = deionized
MS = molecular sieves
CV = Column Volumes
Trap = substrate that reacted with the rhodium carbene (i.e. tert-Butyl 2,5-dihydro-1H-pyrrole-1-carboxylate (*N*-Boc-2,5-dihyropyrrole or *N*-Boc-3-pyrroline))
d.r. = diastereomeric ratio
ee = enantiomeric excess
IS = internal standard
Troc = trichloroethoxy carbonyl (the trichloroethyl ester group)
[2]Tfoc = trifluoroethoxy carbonyl (the trifluoroethyl ester group)
FA = formic acid
[O] = oxidation
EtOAc = ethyl acetate
DCM = dichloromethane
MeOH = methanol
qNMR = quantitative Nuclear Magnetic Resonance (using standard operating procedures from Sigma Aldrich: https://www.sigmaaldrich.com/deepweb/assets/sigmaaldrich/marketing/global/documents/101/854/qnmr-brochure-rjo.pdf)
IPA = isopropanol

2. Reagent Acquisition

i. Reagents Acquired Through Vendors:

Reagent	CAS #	Vendor(s)	Notes
tert-Butyl 2,5-dihydro-1H-pyrrole-1-carboxylate (*N*-Boc-2,5-dihyropyrrole or *N*-Boc-3-pyrroline) (**1**)	73286-70-1	Ambeed, TCI Chemicals, Combi-Blocks	**Impurities** (mainly *N*-Boc pyrrole) **present** in all samples; **repurified before use**. Physical characteristic as a white crystalline solid. See previous study for reagent purification.[3]
CH_2Cl_2, suitable for HPLC, >99.8%, contains amylene as stabilizer	75-09-2	Sigma-Aldrich	Distilled from calcium hydride onto activated 4Å molecular sieves (beads), the distilled solution sparged with nitrogen for at least 15 minutes and stored under nitrogen overnight prior to use.
$Rh_2(OAc)_4$	15956-28-2	Umicore	
$Rh_2(esp)_2$	819050-89-0	Combi-Blocks	
N,N'-Dicyclohexylcarbodiimide	538-75-0	Oakwood Chemicals	
4-(Dimethylamino)pyridine	1122-58-3	Oakwood Chemicals	
2,2,2-trichloroethan-1-ol	115-20-8	Sigma-Aldrich	
2,2,2-Trifluoroethanol	75-89-8	Sigma-Aldrich, Oakwood Chemicals	
Osmium Tetroxide, 4% solution	20816-12-0	Polysciences	**Caution:** Glassware and utensils that came into contact with reagent was quenched with saturated sodium bisulfite solution.

Sodium Azide	26628-22-8	Chem-Impex Int'l Inc.	**Caution:** manipulation of reagent was conducted using only plastic utensils; avoid metal utensils
2-Nitrobenzenesulfonyl chloride	1694-92-4	Oakwood Chemicals, Combi-Blocks	
Ethyl diazoacetate contains ≥ 13 wt. % CH_2Cl_2	623-73-4	Sigma-Aldrich	^1H NMR analysis was performed before use to determine actual wt. % of EDA in CH_2Cl_2; determined to be 74% wt. of EDA in CH_2Cl_2
(S)-2-(Adamantan-1-yl)-2-(((R)-2-hydroxy-1-phenylethyl)amino)acetic acid hydrochloride	361441-96-5	Synthonix, Ambeed	Protected amino acid precursor to chiral dirhodium(II) adamantyl ligands. Ambeed Optical Rotation from COA is -103.387; Purity (NMR) is 97%.
1,8-Diazabicyclo[5.4.0]undec-7-ene	6674-22-2	Oakwood, Ambeed	
4-Acetamidobenzensesulfonyl azide (*p*-ABSA)	2158-14-7	Synthonix	**Caution:** manipulation of reagent was conducted using only plastic utensils; avoid metal utensils
Tetrakis(triphenylphosphine)palladium(0)	14221-01-3	Strem	The reagent is stored at -20 °C under nitrogen after opening. After usage, the reagent bottle is immediately sealed with a septum, backfilled with nitrogen three times, then the punctured septum sealed with melted parafilm and the reagent bottle stored in a -20 °C freezer. The color of the catalyst remains a bright yellow color.
(R)-1-tert-butyl 2-methyl 1H-pyrrole-1,2(2H,5H)-dicarboxylate; 1-(tert-butyl) 2-methyl (R)-2,5-dihydro-1H-pyrrole-1,2-dicarboxylate	220652-51-7	Ambeed	**Contains TBC**; **repurified before use** in carbene reactions.
(S)-1-tert-Butyl 2-methyl 1H-pyrrole-1,2(2H,5H)-dicarboxylate; 1-(tert-butyl) 2-methyl (S)-2,5-dihydro-1H-pyrrole-1,2-dicarboxylate	74844-93-2	Ambeed	**Contains TBC**; **repurified before use** in carbene reactions.
DIBAL-H solution, 1.0 M in CH_2Cl_2	1191-15-7	Sigma-Aldrich	
Zinc dust (<10 μm particle size)	7440-66-6	Sigma-Aldrich	
Acetic Acid (glacial, ACS reagent)	64-19-7	Sigma-Aldrich	
Tifluoroacetic Acid	76-05-1	Oakwood Chemicals	
Lithium borohydride solution, 2.0 M in THF	16949-15-8	Sigma-Aldrich	
4-Methylmorpholine N-oxide monohydrate	70187-32-5	Sigma-Aldrich	
2-(3-bromophenyl)acetic acid	1878-67-7	Ambeed	
2,3-Dihydrobenzofuranyl-5-acetic acid	69999-16-2	Ambeed	
2-(benzo[d][1,3]dioxol-5-yl)acetic acid	2861-28-1	AccelaChem	
2-(4-iodophenyl)acetic acid	1798-06-7	Oakwood, Combi-Bocks	
Chloroform-*d*	865-49-6	Sigma-Aldrich	Before use, the deuterated solvent was neutralized by adding in an excess of $BaCO_3$ and $MgSO_4$, filtered, and the filtrate used for NMR analysis.

tert-butylchlorodimethylsilane	18162-48-6	Oakwood Chemicals	Use fresh for alcohol protection; we observed diminished yields when we used old-quality of reagent
CD_3OD (MeOD)	811-98-3	Sigma-Aldrich	
Sodium Chlorite	7758-19-2	Sigma-Aldrich	Technical grade, 80%
9-Mesityl-10-methylacridinium tetrafluoroborate	1442433-71-7	Sigma-Aldrich	
2-Methyl-2-butene	513-35-9	TCI America	
3-Chloroperbenzoic acid	937-14-4	Oakwood Chemicals	
Imidazole	288-32-4	Oakwood Chemicals	

ii. Reagents Acquired Through Known Procedures:

Reagent	CAS #	Reference(s)	Notes
2,2,2-trichloroethyl 2-diazoacetate	60719-24-6	Davies, H. M. L. *et al.* **2017**[4]	
2,2,2-trichloroethyl 2-(4-bromophenyl)acetate	946825-15-6	Sigman, M. S. *et al.* **2015**[5]	
2-diazo-2-phenyl-1-(4-(trifluoromethyl)phenyl)ethan-1-one	2765408-70-4	Davies, H. M. L. *et al.* **2024**[6]	
2,2,2-trichloroethyl 2-([1,1'-biphenyl]-4-yl)-2-diazoacetate	2271117-09-8	Davies, H. M. L. *et al.* **2020**[7]	
2,2,2-trichloroethyl 2-diazo-2-(4-(trifluoromethyl)phenyl)acetate	1641528-28-0	Reiser, O. *et al.* **2023**[8]	
2,2,2-trichloroethyl 2-diazo-2-phenylacetate	2072109-81-8	Reiser, O. *et al.* **2023**[8]	
(S)-((3S,5S,7S)-adamantan-1-yl)(carboxy)methanaminium chloride	102502-64-7	Poh, J.-S. *et al.* **2017**[9]	Synthesized from (S)-2-(Adamantan-1-yl)-2-(((R)-2-hydroxy-1-phenylethyl)amino)acetic acid hydrochloride. $[\alpha]^{20}_D$: +28.8° (c = 1.930 g/100 mL, MeOH).

3. Catalyst Structures and Structure Activity Relationship (SAR) Study

The following catalysts were used in this study:

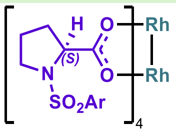 Ar = *p*-$(C_{12}H_{25})C_6H_4$<br>**Rh_2(*S*-DOSP)$_4$**	**Generation:** Prolinato<br>**Name:** Rh_2(*S*-DOSP)$_4$[10]

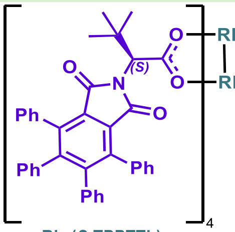 Rh_2(*S*-TPPTTL)$_4$	**Generation:** Phthalimido<br>**Name:** Rh_2(*S*-TPPTTL)$_4$[11]
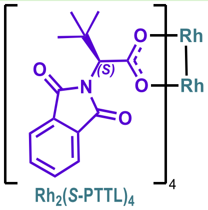 Rh_2(*S*-PTTL)$_4$	**Generation:** Phthalimido<br>**Name:** Rh_2(*S*-PTTL)$_4$[12]
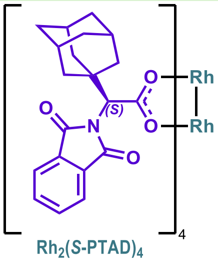 Rh_2(*S*-PTAD)$_4$	**Generation:** Phthalimido<br>**Name:** Rh_2(*S*-PTAD)$_4$[13]
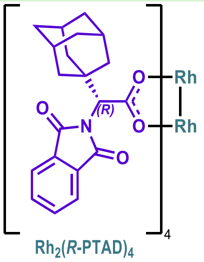 Rh_2(*R*-PTAD)$_4$	**Generation:** Phthalimido<br>**Name:** Rh_2(*R*-PTAD)$_4$[13]
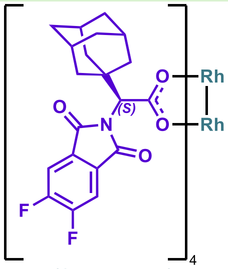 Rh_2(*S*-4,5-DFPTAD)$_4$	**Generation:** Phthalimido<br>**Name:** Rh_2(*S*-4,5-DFPTAD)$_4$
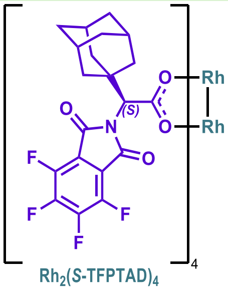 Rh_2(*S*-TFPTAD)$_4$	**Generation:** Phthalimido<br>**Name:** Rh_2(*S*-TFPTAD)$_4$[14]

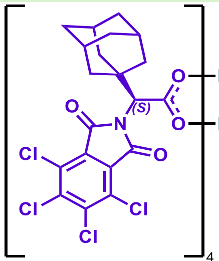 Rh_2(*S*-TCPTAD)$_4$	**Generation:** Phthalimido **Name:** Rh_2(*S*-TCPTAD)$_4$[15]
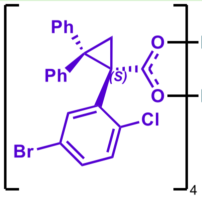 Rh_2(*S*-2-Cl,5-BrTPCP)$_4$	**Generation:** TPCP **Name:** Rh_2(*S*-2Cl,5Br-TPCP)$_4$[16]
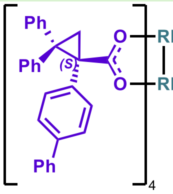 Rh_2(*S*-*p*-PhTPCP)$_4$	**Generation:** TPCP **Name:** Rh_2(*S*-*p*-PhTPCP)$_4$[17]

Figure S1. Catalyst structures used in catalyst screen.

Flexible Complexes

(C_4, C_1)

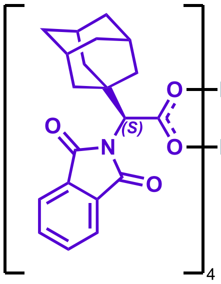

Rh_2(*S*-PTAD)$_4$
Symmetry: C_4, C_1
ee: 96%

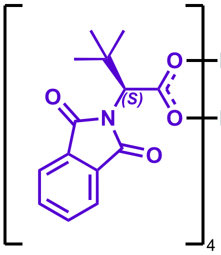

Rh_2(*S*-PTTL)$_4$
Symmetry: C_4, C_1
ee: 94%

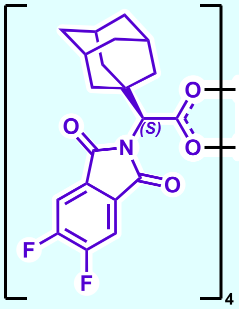

Rh_2(*S*-4,5-DFPTAD)$_4$
Symmetry: C_4, C_1
ee: **92%**

Rigid Complexes

(C_4)

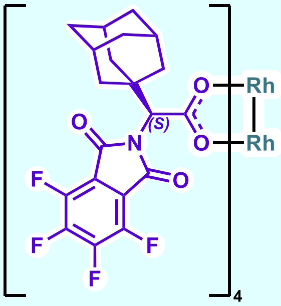

Rh_2(*S*-TFPTAD)$_4$
Symmetry: C_4
ee: **54%**

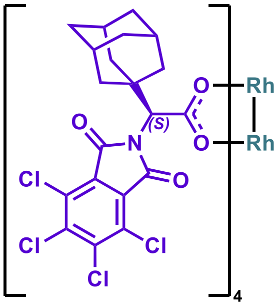

Rh_2(*S*-TCPTAD)$_4$
Symmetry: C_4
ee: 52%

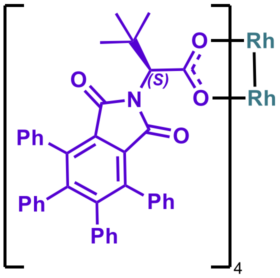

Rh_2(*S*-TPPTTL)$_4$
Symmetry: C_4
ee: 46%

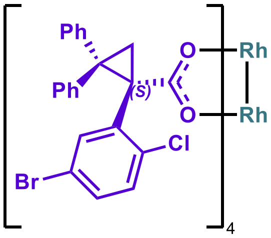

Rh_2(*R*-2-Cl-5-BrTPCP)$_4$
Symmetry: C_4
ee: 58%

Figure S2. Enantiomeric excess (ee) values from C–H functionalization of 2,5-dihydro-1*H*-pyrrole to furnish **15**. Flexible C_4-symmetric catalysts give up to 96% ee. Rigid C_4-symmetric catalysts give up to 58% ee. Understanding the molecular dynamics in the asymmetric induction of chiral dirhodium(II) tetracarboxylate C–H functionalization is one still one of the grand challenges of this chemistry. To have an initial understanding of the high enantioinduction by Rh_2(*S*- or *R*-PTAD)$_4$, we studied the structure activity relationship assessing the enantioselectivity of our toolbox of chiral dirhodium tetracarboxylate catalysts using our optimal conditions. We observed that rigid C_4-symmetric complexes furnished ee's between 46 – 58%. With flexible C_4-symmetric complexes, the ee's were significantly higher, ranging from 92 to 96% ee. This suggest that a crucial component for the high enantioinduction is the ability of the ligand to flip.

Observations

1. Match-pair analysis shows that having a bulkier sidechain such as an adamantyl group versus a *tert*-butyl group slightly improves ee (Rh_2(*S*-PTAD)$_4$ versus Rh_2(*S*-PTTL)$_4$).
2. Match-pair analysis shows that having a halogen substituted at the 3,6-position of the benzene ring significantly erodes ee (Rh_2(*S*-TFPTAD)$_4$ versus Rh_2(*S*-4,5-DFPTAD)$_4$)). Halogens at the 3,6-position of the benzene ring participate in halogen-bonding with the adjacent carbonyl in the rhodium complex, which rigidifies the complex;[18] rigid complexes display moderate ee's.

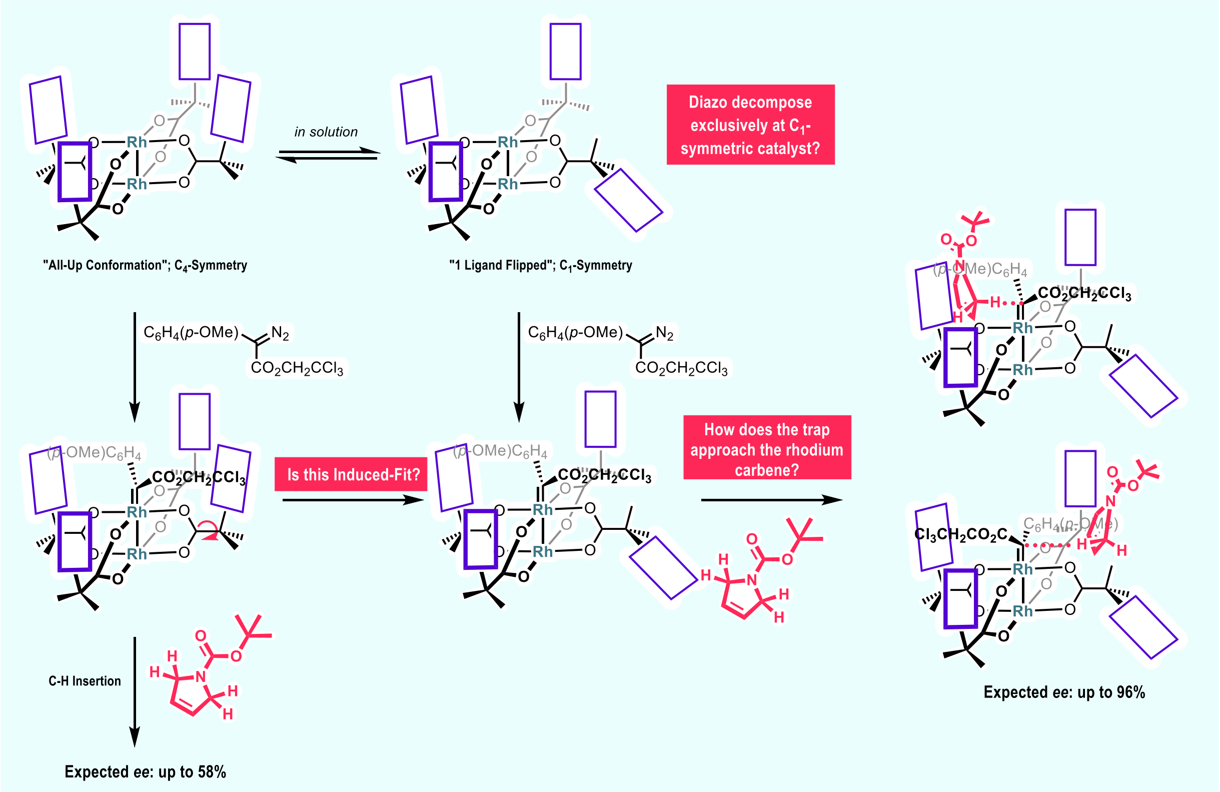

Figure S3. Proposed hypothesis for high enantioinduction by flexible chiral C_4-symmetric dirhodium tetracarboxylate catalysts to furnish **15**. As observed by the Charette Group[18], we proposed that in solution, the flexible C_4-symmetric conformer (all-up conformation) is in equilibrium with the C_1-symmetric conformer (1-ligand flipped), in which the C_1-symmetric conformer is the complex to give the high enantioselectivity. If the diazo is decomposed by the C_4-symmetric conformer, we would expect either 1) the trap reacting with the carbene in an all-up conformation with a relatively difficult facial selectivity, similar to the rigid C_4-symmetric catalysts or 2) one ligand is flipped due to the induced-fit of the diazo decomposition, setting up a C_1-symmetric complex, and the trap interacts with this carbene complex with a high facial preference resulting in high ee. The latter is plausible since one face of the carbene is more open than the other,

allowing for the trap to favor the more opened face of the olefin. Further computation simulation techniques will be necessary to understand the behavior of these catalysts.

4. Determination of d.r. by *N*-Boc deprotection and/or Variable Temperature ^{1}H NMR

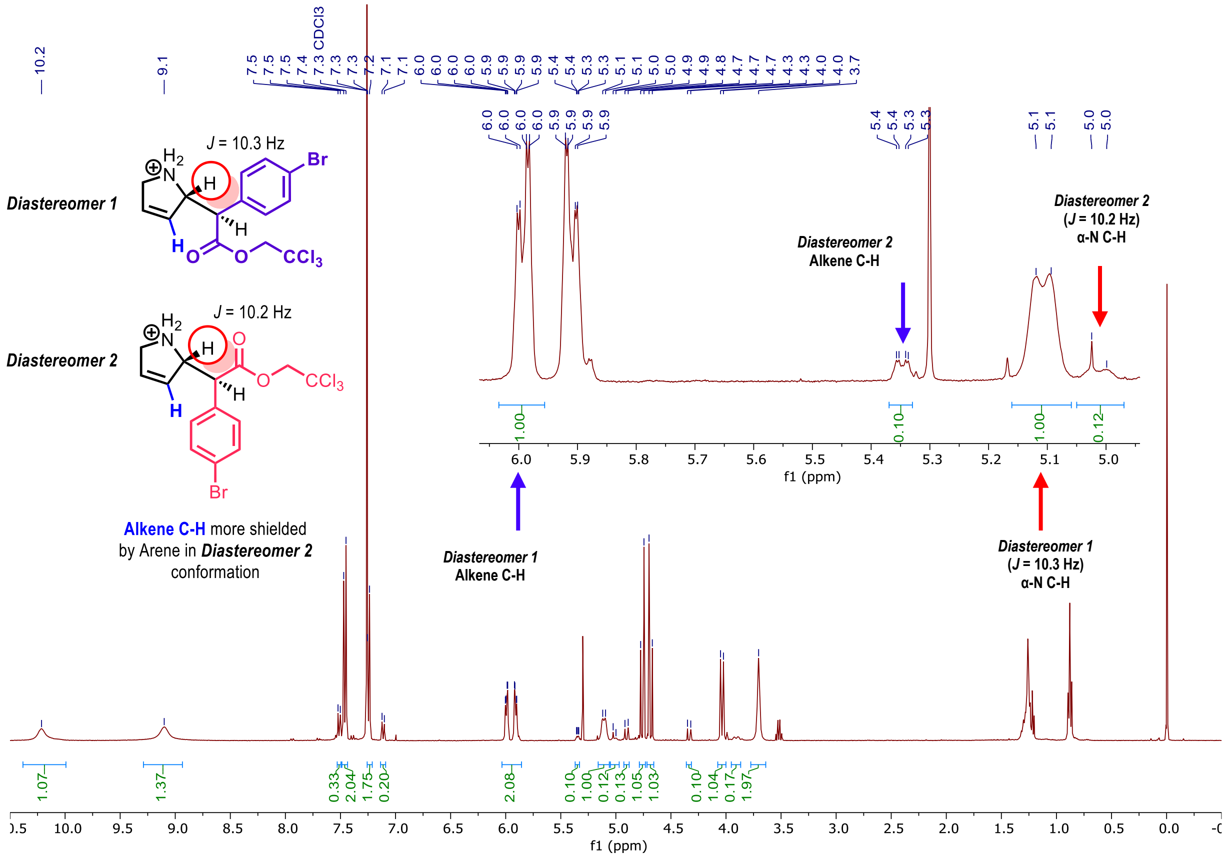

Figure S4. $Rh_2(OAc)_4$ C–H functionalization followed by TFA to form **17-deboc** as the TFA salt (d.r. 10:1). Performed on 400 MHz ^{1}H NMR in $CDCl_3$. Analysis performed to determine diagnostic peaks for both diastereomers. Conformational and stereochemical analysis adapted from Davies, H. M. L. *et al.* **2001**.[19]

5. Observation of Retro-Mannich Reaction Through *N*-Boc Deprotection Followed by Free-Base

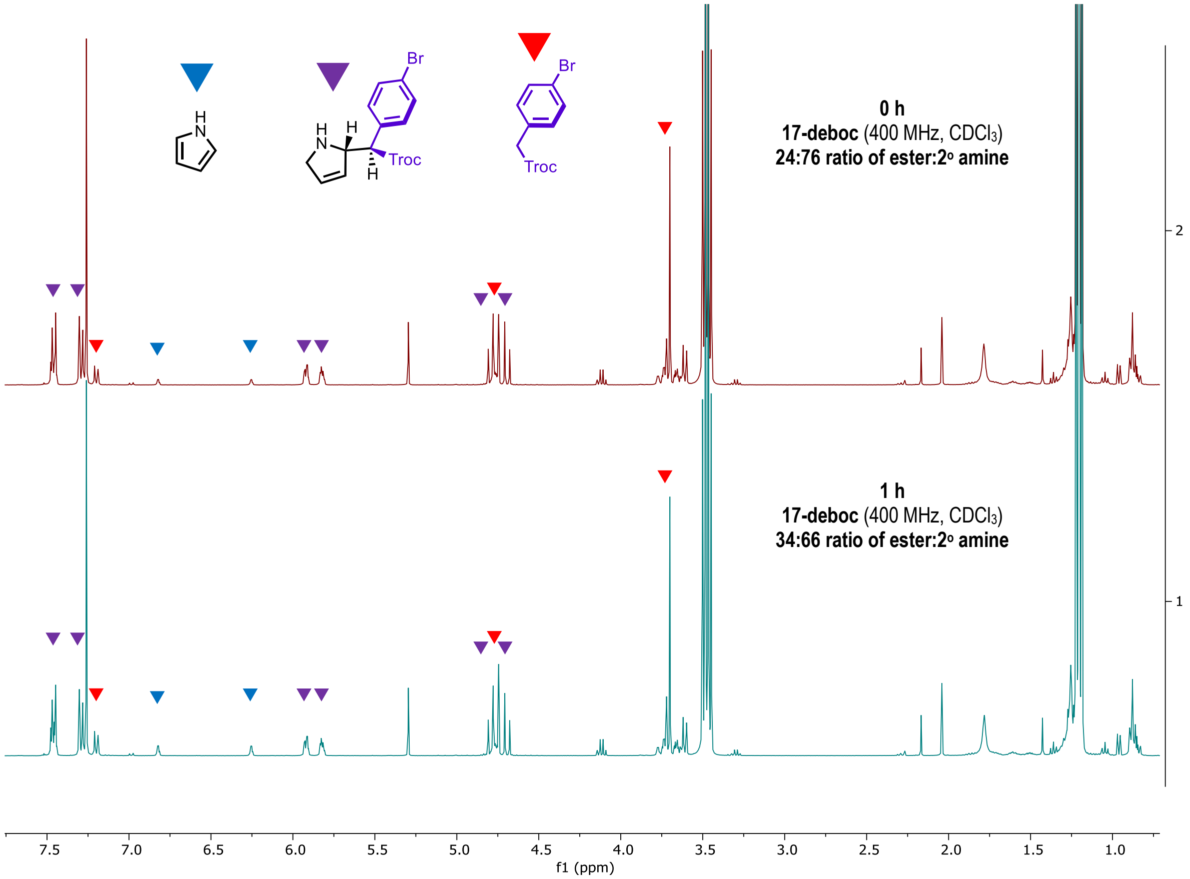

Figure S5. *p*TSA deprotection of **17** followed by DI water neutralization and extraction of organic material to form **17-deboc**. The top spectrum is the crude ^{1}H NMR right after neutralization. The bottom spectrum is the same crude ^{1}H NMR in $CDCl_3$ after 1 hour. The neutral amine seems to inherently decompose through the retro-Mannich to form pyrrole and 2,2,2-trichloroethyl 2-(4-bromophenyl)acetate over time. Neutralization with saturated sodium bicarbonate, 5% sodium bicarbonate solution, or triethylamine (1.1 equiv) in ether gave similar results to the DI water neutralization – decomposition of neutral amine observed through retro-Mannich.

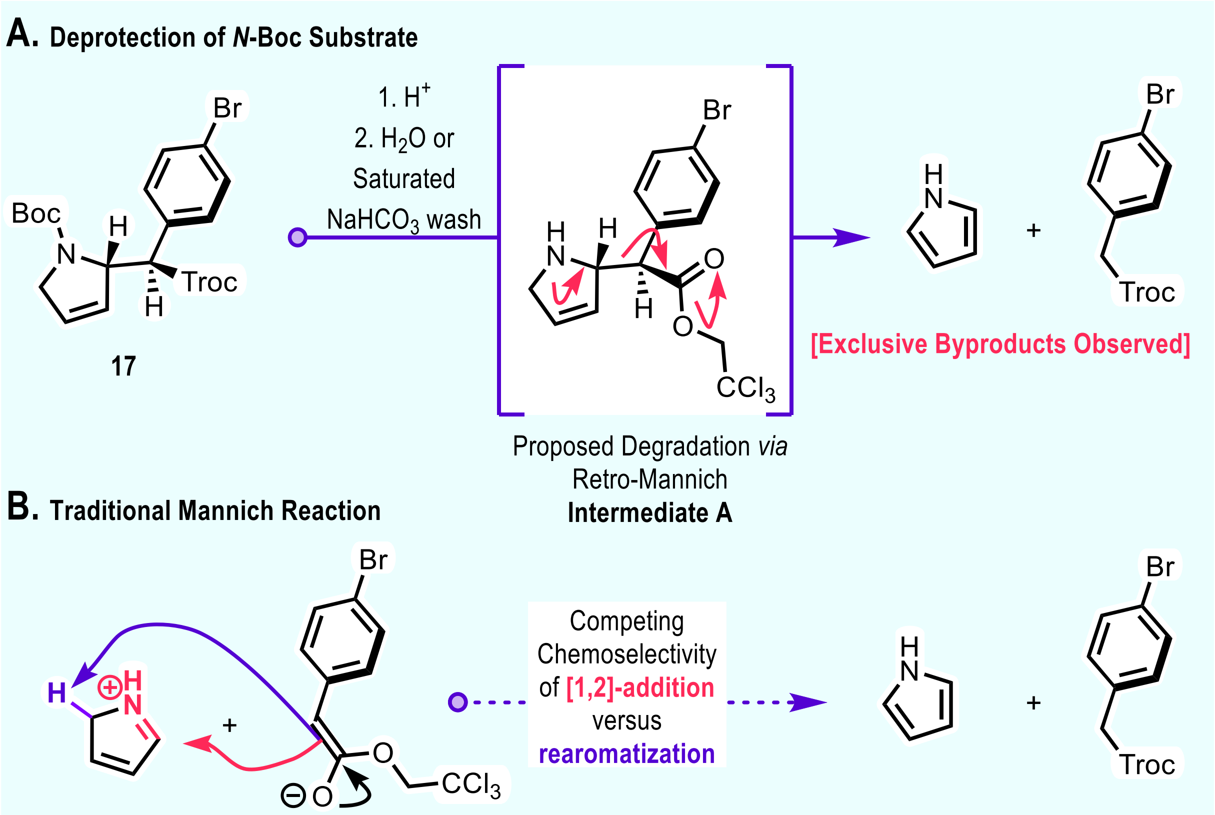

Figure S6. A) After deprotection of **17** with H+ (*p*TSA, TFA, HCl, etc.) followed by free-base with H_2O or saturated bicarbonate solution, the neutral amine undergoes a retro-Mannich to furnish pyrrole and 2,2,2-trichloroethyl 2-(4-bromophenyl)acetate. B) Competing chemoselectivity of the enolate to the iminium ion. The unexpected decomposition observed upon neutralization of the salt after the cleavage of the protecting group in *N*-Boc-dihydropyrrole moiety is a limitation to synthesize these scaffolds through a traditional Mannich reaction. This chemistry would go through the iminium intermediate, which makes the enolate behave as a base deprotonating the acidic C_{sp^3}-H α-*N* proton to rearomatize to the pyrrole rather than a nucleophile to the iminium center to generate intermediate A, which in turn can undergo the retro-Mannich to reform the iminium and enolate. Alternatively with the C–H functionalization pathway, this process does not generate a fully zwitterionic intermediate but goes through an asynchronous concerted mechanism with partial positive charge build up on the α-*N* carbon and partial negative charge build up on the carbene center. This results in a productive reaction to asymmetrically (under chiral catalyst control) functionalize the α-*N* C_{sp^3}-H bond of *N*-Boc-2,5-dihydro-1*H*-pyrrole.[20]

6. Characterization of Catalysts and Starting Materials

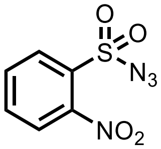

o-Nitrobenzenesulfonyl Azide; *o*-NBSA (S1)

Caution: manipulation of reagent was conducted using only plastic utensils; avoid metal utensils. Avoid the use of any halogenated solvents (such as CH_2Cl_2). Additionally, blast-shields should be placed in front of the azide material when performing manipulations as a precaution.

With a blast-shield in front, to a 5 L erlenmeyer flask charged with a stir-bar was added 2-nitrobenzenesulfonyl chloride (148 g, 1 equiv, 668 mmol) and acetone (698 g, 884 mL, 18 equiv, 12.0 mol) and water (265 g, 0.26 L, 22 equiv, 14.7 mol). To the reaction vessels were added sodium azide (47.8 g,

1.1 equiv, 735 mmol) via a plastic spatula, rinsing any excess/residual azide into the reaction vessel with DI water. The resulting solution is an opaque and orange solution. To the reaction mixture was stirred at 25 °C for 24 hours open to air with a stream of gentle air to slowly evaporate the acetone as the reaction proceeds. After the elapsed time, the reaction mixture is nearly dried out with white solids. With a blast-shield in front, the reaction mixture was resuspended in excess DI water and ethanol and vacuum filtered through a Büchner funnel with a paper filter. The filter cake was washed thoroughly with DI water and ethanol and then vacuum dried to afford *o*-NBSA (151 g, 662 mmol, 99.1% yield) as an off-white amorphous powder. The material was transferred to a plastic container via a plastic utensil and placed in a -20 °C fridge for storage. ^{1}H NMR spectrum is in good agreement with literature precedent.[21]

^{1}H NMR (400 MHz, Chloroform-*d*) δ 8.20 (dd, *J* = 7.8, 1.5 Hz, 1H), 8.03 – 7.74 (m, 3H).

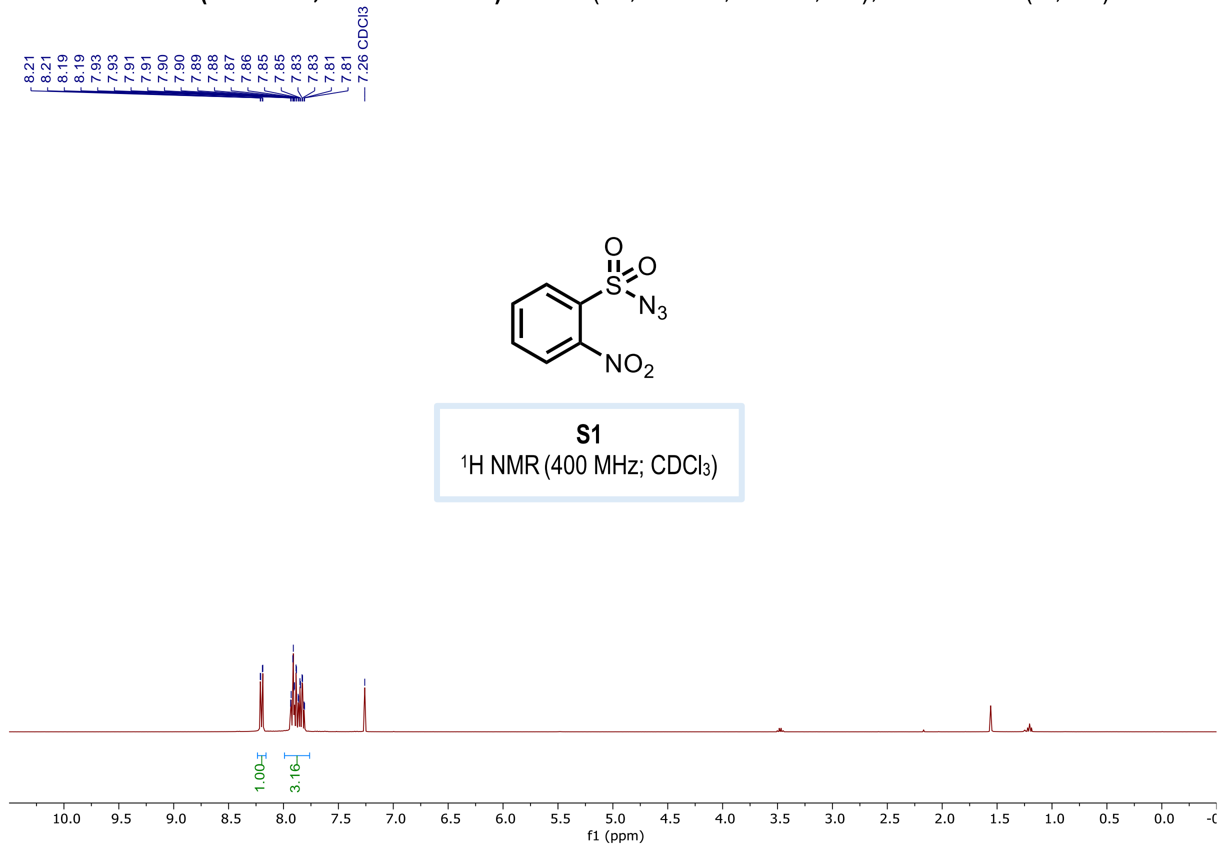

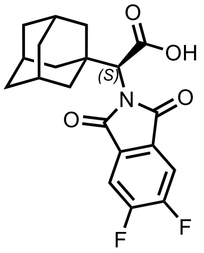

(S)-2-((3S,5S,7S)-adamantan-1-yl)-2-(5,6-difluoro-1,3-dioxoisoindolin-2-yl)acetic acid (S2)

To a flame-dried RBF charged with a stir-bar was added (S)-((3S,5S,7S)-adamantan-1-yl)(carboxy)methanaminium chloride (3.686 g, 1 equiv, 15.00 mmol), 5,6-difluoroisobenzofuran-1,3-dione (3.314 g, 1.2 equiv, 18.00 mmol) and then acetic acid (900.7 mg, 75.00 mL, 0.20 molar, 1 equiv, 15.00 mmol). The reaction vessel was topped with a findenser and then the reaction mixture was stirred at 140 °C for 48 hours. After the elapsed time, the reaction mixture was concentrated *in vacuo* at 80 °C to remove excess AcOH. The crude was dry-loaded onto SiO_2 and subjected to SiO_2 flash chromatography using

pentane/EtOAc (Biotage Sfär DLV 100 g column as the running-column and Biotage Sfär DLV 50 g column for the dry-load; 0% 6CV → 5% 6CV →10% 6CV → 15% 6CV → 20% 6CV; 100 mL/min flow-rate; 254/280 nm detector; POI elutes at 20%; **Note:** *make sure to remove excess AcOH or product will drag throughout the column*). The collected fractions were concentrated *in vacuo* at 80 °C to afford (S)-2-((3S,5S,7S)-adamantan-1-yl)-2-(5,6-difluoro-1,3-dioxoisoindolin-2-yl)acetic acid (2.3 g, 6.1 mmol, 41% yield) as a white amorphous powder.

^{1}H NMR (800 MHz, Chloroform-*d*) δ 7.68 (dd, *J* = 6.9 Hz, 6.9 Hz, 2H), 4.54 (s, 1H), 1.96 (brd, *J* = 7.6 Hz, 6H), 1.70 (brd, *J* = 11.5 Hz, 3H), 1.64 (brq, *J* = 12.1 Hz, 6H).

^{13}C NMR (201 MHz, Chloroform-*d*) δ 173.4, 166.1, 154.6 (dd, J_{C-F} = 261.2, 15.0 Hz), 128.5 (br), 113.6 (d, J_{C-F} = 20.7 Hz), 61.1, 39.3, 37.7, 36.5, 28.4.

^{19}F NMR (753 MHz, Chloroform-*d*) δ -125.1.

HRMS (APCI +) calc. mass for $C_{20}H_{20}O_4NF_2$ [M + H]$^+$ 376.1355; obs. mass for $C_{20}H_{20}O_4NF_2$ [M + H]$^+$ 376.1351.

$[\alpha]^{20}_D$: -50.9° (c = 3.175 g/100 mL, EtOAc).

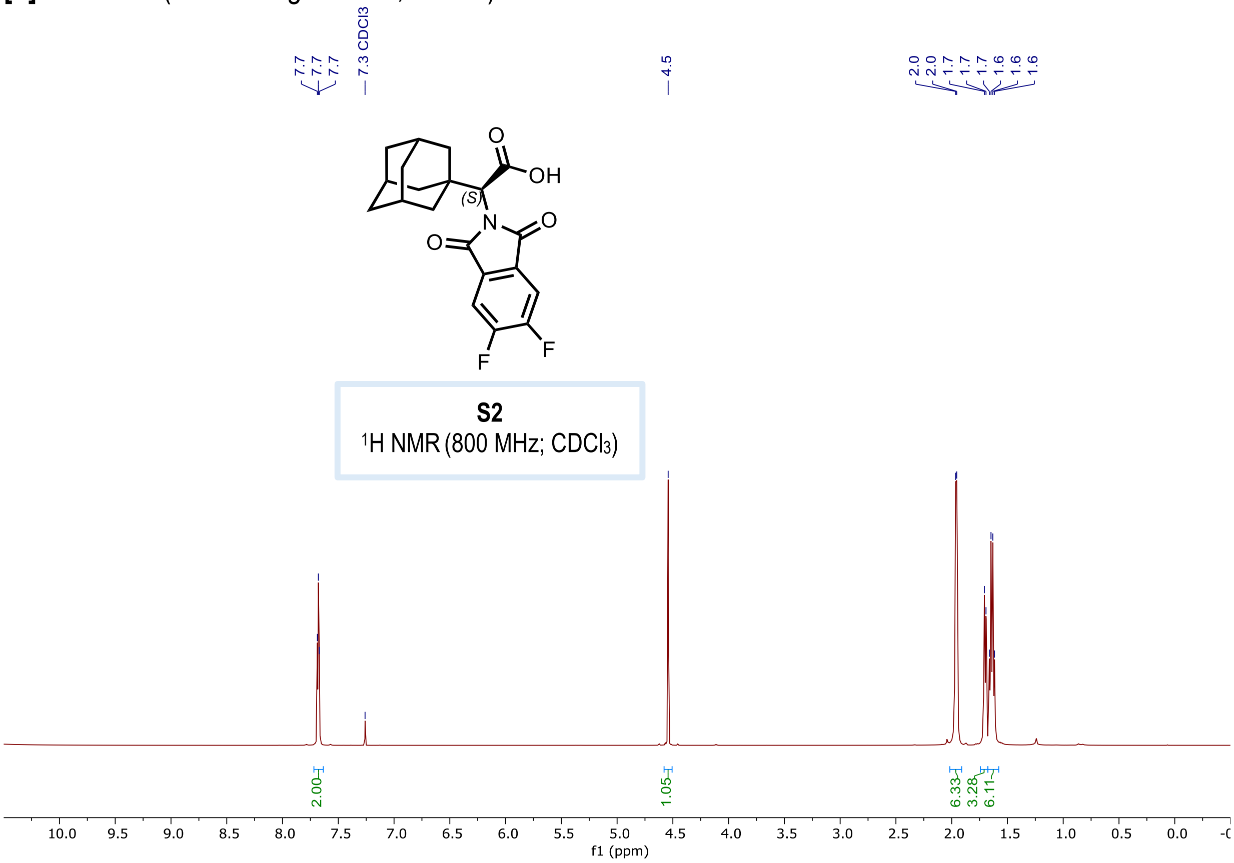

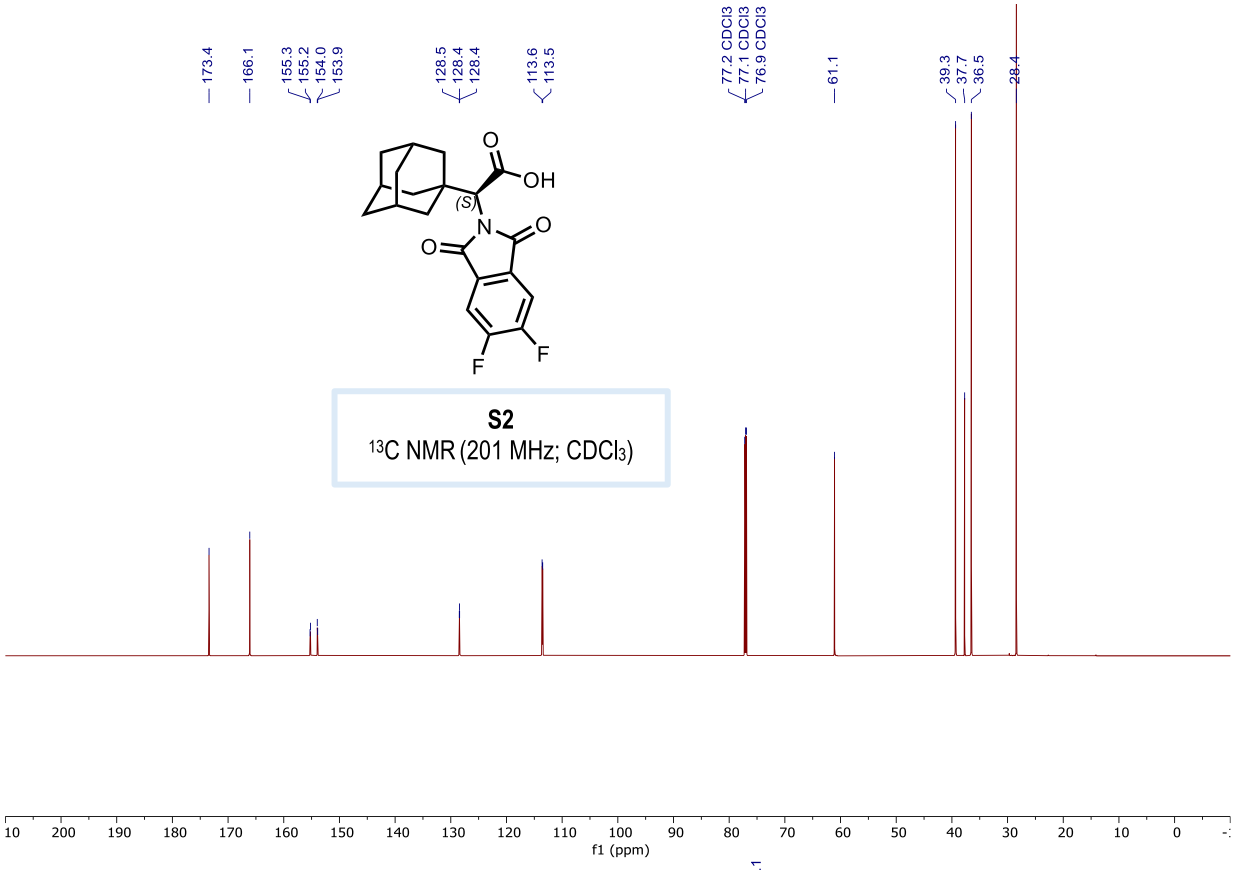
173.4
166.1
155.3
155.2
154.0
153.9
128.5
128.4
128.4
113.6
113.5
77.2 CDCl3
77.1 CDCl3
76.9 CDCl3
61.1
39.3
37.7
36.5
28.4
O
OH
(S)
N
O
O
F
F
S2
^{13}C NMR (201 MHz; $CDCl_3$)
f1 (ppm)

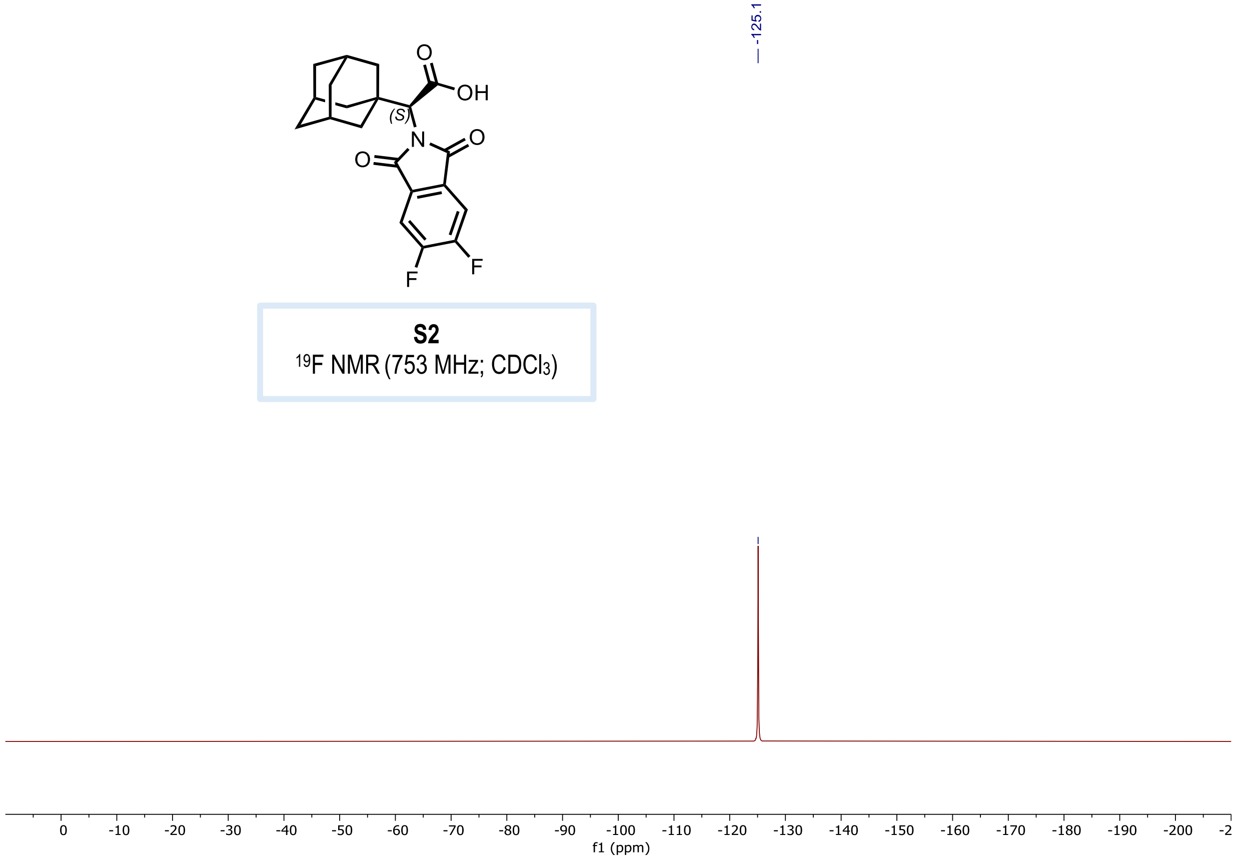
-125.1
O
OH
(S)
N
O
O
F
F
S2
^{19}F NMR (753 MHz; $CDCl_3$)
f1 (ppm)

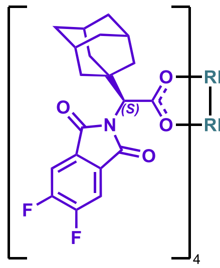

Rh_2(*S*-4,5-DFPTAD)$_4$ (S3)

To a flame-dried RBF charged with a stir-bar was equipped with a soxhlet apparatus charged with 1:1 mixture of sodium carbonate:sand. To the RBF was added (S)-2-((3S,5S,7S)-adamantan-1-yl)-2-(5,6-difluoro-1,3-dioxoisoindolin-2-yl)acetic acid (1.80 g, 6 equiv, 4.80 mmol), rhodium(II) acetate dimer (354 mg, 1 equiv, 0.800 mmol) and chlorobenzene (90.0 mg, 32.0 mL, .025 molar, 1 equiv, 800 μmol). The reaction solution was heated to 185 °C and stirred for 18 hours. The reaction mixture was cooled and concentrated *in vacuo* at 65 °C. The material was resuspended in diethyl ether and passed through a plug of neutral alumina. To the filtrate was added SiO_2 and concentrated *in vacuo* at 45 °C. The SiO_2 dry-load was subjected to SiO_2 flash chromatography using pentane/ether (Biotage Sfär DLV 100 g column as the running-column and Biotage Sfär DLV 50 g column for the dry-load; 0% 6CV → 1% 6CV → 5% 6CV → 7% 6CV → 10% 6CV → 12% 6CV → 15% 6CV; 100 mL/min flow-rate; 210/254 nm detector; POI eluted at 12% ether). The collected fractions were concentrated *in vacuo* at 65 °C. The dried material was dissolved in diethyl ether and vacuum filtered with neutral alumina to get rid of residual ligands. The filtrate was concentrated *in vacuo* at 65 °C and then further dried under vacuum at 85 °C overnight to afford Rh_2(*S*-4,5-DFPTAD)$_4$ (259 mg, 152 μmol, 19.0% yield) as a green amorphous powder.

^{1}H NMR (600 MHz, Chloroform-*d*) δ 7.63 (brs, 8H), 4.65 (s, 4H), 2.0 – 1.86 (m, 22H), 1.72 – 1.62 (m, 38H).

^{13}C NMR (151 MHz, Chloroform-*d*; mixture of conformers) δ 186.7, 166.4 & 165.6 (each br, 2 conformers of Ar–C–H), 154.4 (d, J_{C-F} = 251.5 Hz), 128.6 (dd, J_{C-F} = 7.6, 3.1 Hz), 113.3 (m), 66.3, 62.5, 39.3, 37.6, 36.9, 28.6, 15.0.

^{19}F NMR (565 MHz, Chloroform-*d*; mixture of conformers) δ -125.5, -125.7, -126.1, -126.2.

HRMS (ESI +) calc. mass for $C_{80}H_{72}O_{16}N_4F_8{}^{103}Rh_2$ [M]$^+$ 1702.2920; obs. mass for $C_{80}H_{72}O_{16}N_4F_8{}^{103}Rh_2$ [M]$^+$ 1702.2789.

$[\alpha]^{20}_D$: +66.3° (c = 0.130 g/100 mL, EtOAc).

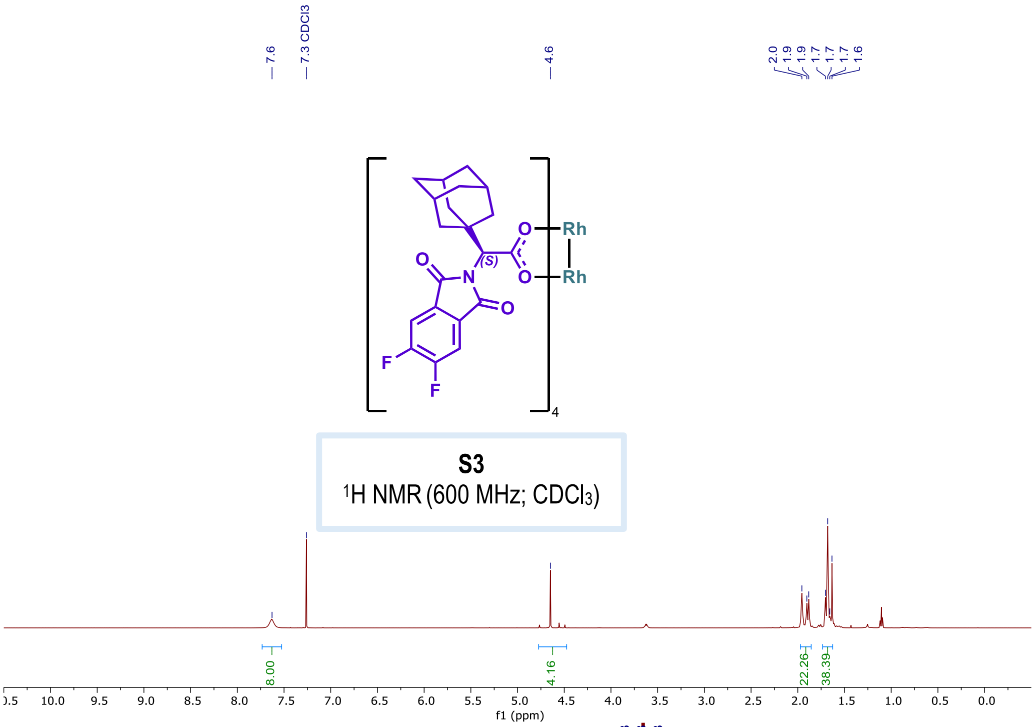
S3
1H NMR (600 MHz; CDCl3)
7.6
7.3 CDCl3
4.6
2.0
1.9
1.9
1.7
1.7
1.7
1.6
8.00
4.16
22.26
38.39
f1 (ppm)

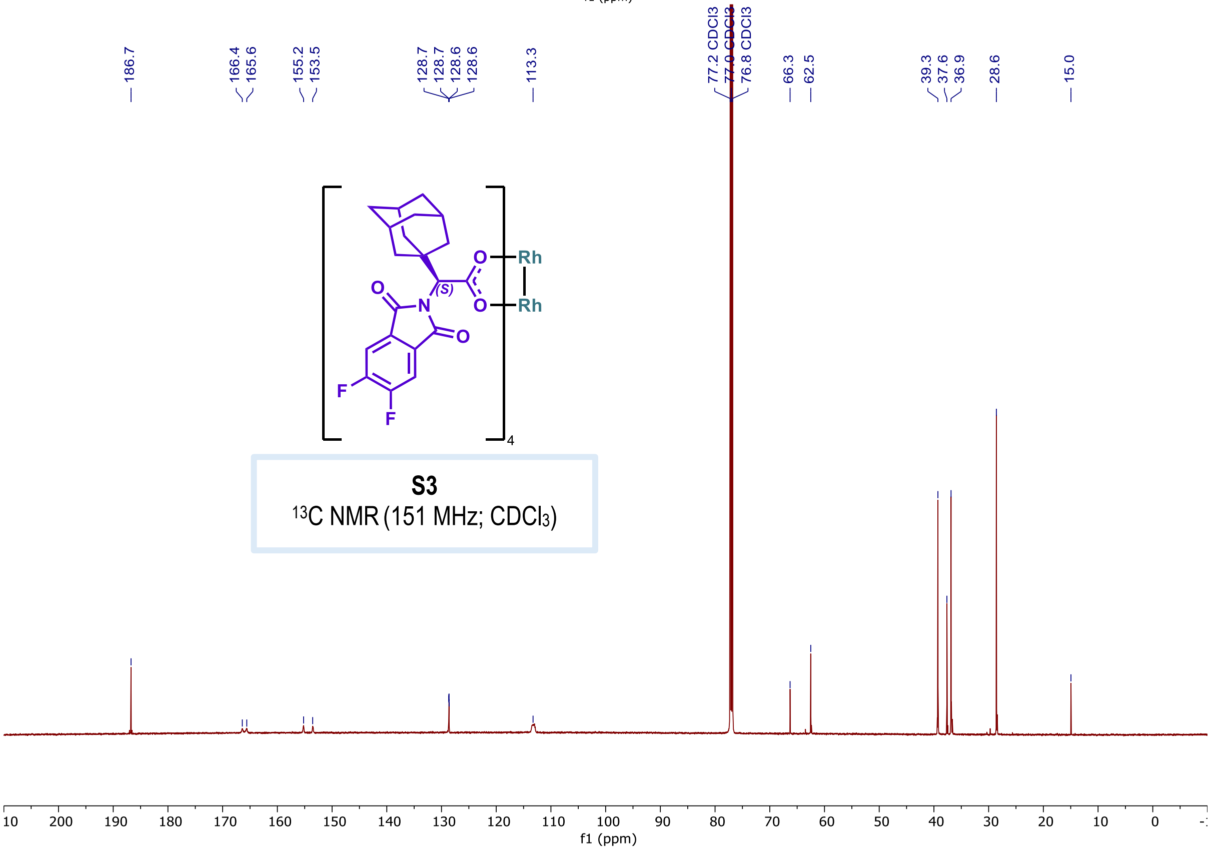
S3
13C NMR (151 MHz; CDCl3)
186.7
166.4
165.6
155.2
153.5
128.7
128.7
128.6
128.6
113.3
77.2 CDCl3
76.8 CDCl3
66.3
62.5
39.3
37.6
36.9
28.6
15.0
f1 (ppm)

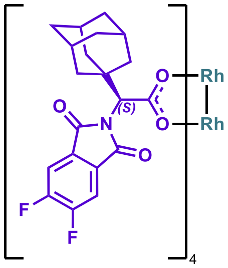

S3
^{19}F NMR (151 MHz; $CDCl_3$)

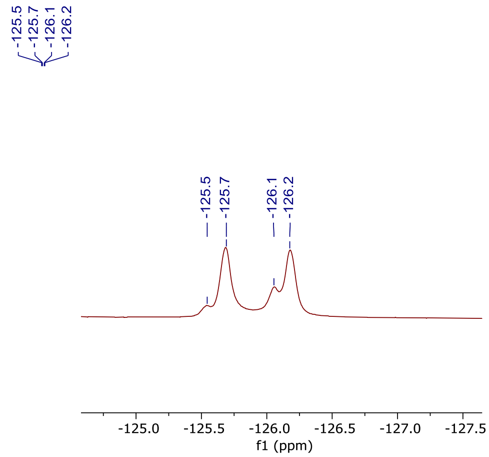

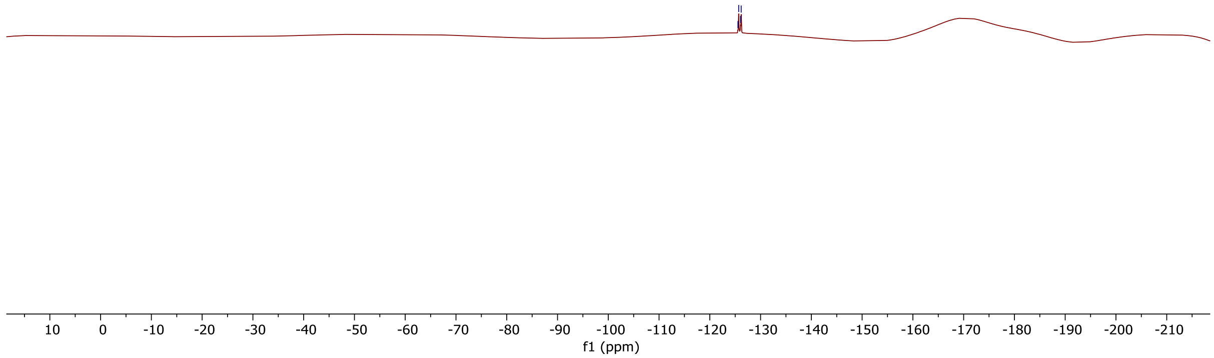

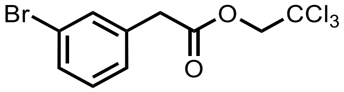

2,2,2-trichloroethyl 2-(3-bromophenyl)acetate (S4)

To an erlenmeyer flask charged with a stir-bar was added: 2-(3-bromophenyl)acetic acid (4.301 g, 1 equiv, 20.00 mmol), 2,2,2-trichloroethan-1-ol (3.586 g, 2.30 mL, 1.2 equiv, 24.00 mmol), and N,N-dimethylpyridin-4-amine (244.3 mg, 0.1 equiv, 2.000 mmol) and dichloromethane (1.699 g, 66.67 mL, 0.3 molar, 1 equiv, 20.00 mmol). To the stirred reaction mixture was added DCC (4.539 g, 3.44 mL, 1.1 equiv, 22.00 mmol) and was allowed to stir at room temperature overnight. The reaction mixture was vacuum filtered through a celite plug and the filtrate concentrated *in vacuo*. The crude material was dry-loaded onto SiO_2 and subjected to SiO_2 flash chromatography hexanes/diethyl ether (Biotage Sfär DLV 100 g column; step-gradient 0% 3CV → 1% 3CV → 25% 2CV → 25% - 50% 2CV → 50% 2CV; 200 mL/min flow rate; 210/254 nm detector). The collected fractions were concentrated *in vacuo* to afford 2,2,2-trichloroethyl 2-(3-bromophenyl)acetate (5.87 g, 16.9 mmol, 84.7% yield) as a clear colorless oil. ^{1}H NMR spectrum is in good agreement with literature precedent.[22]

^{1}H NMR (400 MHz, Chloroform-*d*) δ 7.50 (t, *J* = 1.8 Hz, 1H), 7.43 (dt, *J* = 7.6, 1.8 Hz, 1H), 7.29 – 7.15 (m, 2H), 4.76 (s, 2H), 3.74 (s, 2H).

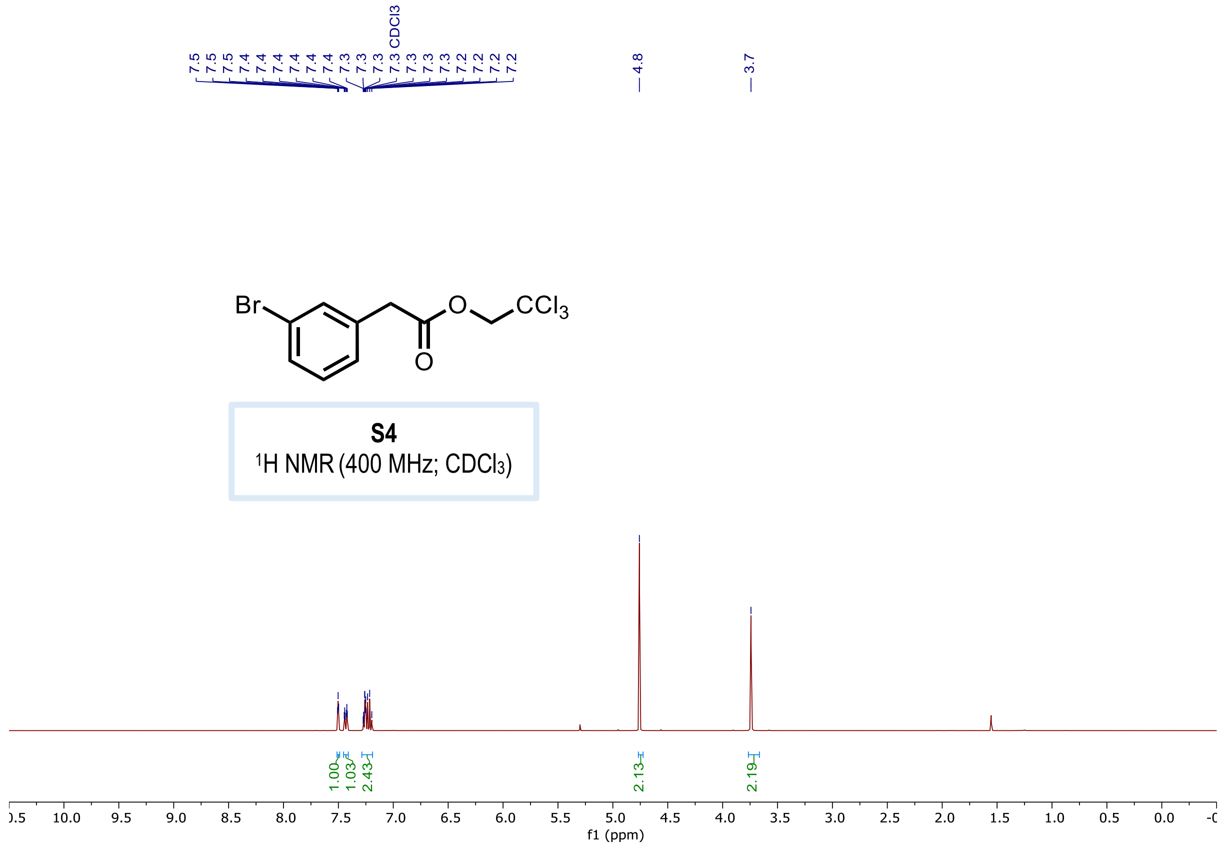

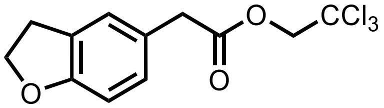

2,2,2-trichloroethyl 2-(2,3-dihydrobenzofuran-5-yl)acetate (S5)

To a RBF charged with a stir-bar was added: 2-(2,3-dihydrobenzofuran-5-yl)acetic acid (8.9 g, 1 equiv, 50 mmol), 2,2,2-trichloroethan-1-ol (9.0 g, 5.7 mL, 1.2 equiv, 60 mmol), and N,N-dimethylpyridin-4-amine (0.61 g, 0.1 equiv, 5.0 mmol) and dichloromethane (4.2 g, 0.17 L, 0.3 molar, 1 equiv, 50 mmol). To the stirred reaction mixture was added DCC (11 g, 8.6 mL, 1.1 equiv, 55 mmol) and was allowed to stir at room temperature overnight. The reaction mixture was vacuum filtered through a celite/alumina (celite on top, alumina bottom) plug, washing the filter cake with CH_2Cl_2, and the filtrate concentrated *in vacuo* at 75 °C. The dried crude was redissolved in diethyl ether, filtered through a celite/alumina (celite on top, alumina bottom) plug, washing the filter cake with diethyl ether, and the filtrate concentrated *in vacuo* at 75 °C. The crude mixture was dry-loaded onto SiO_2 and subjected to SiO_2 flash chromatography hexanes/ether (Biotage Sfär DLV 100 g column; 0% 3CV → 0 to 100% 10CV → 100% 6CV; 100 mL/min flow-rate; 210/230 nm detector). The collected fractions were concentrated *in vacuo* at 45 °C to afford 2,2,2-trichloroethyl 2-(2,3-dihydrobenzofuran-5-yl)acetate (13.5 g, 43.6 mmol, 87% yield) as a white amorphous powder.

^{1}H NMR (800 MHz, Chloroform-*d*) δ 7.16 (brs, 1H), 7.04 (dd, *J* = 8.2, 1.7 Hz, 1H), 6.74 (d, *J* = 8.2 Hz, 1H), 4.75 (s, 2H), 4.57 (t, *J* = 8.7 Hz, 2H), 3.69 (s, 2H), 3.20 (t, *J* = 8.7 Hz, 2H).

^{13}C NMR (201 MHz, Chloroform-*d*) δ 170.5, 159.5, 129.1, 127.5, 126.0, 124.7, 109.3, 94.9, 74.2, 71.3, 40.3, 29.7.

HRMS (APCI +) calc. mass for $C_{12}H_{11}O_3{}^{35}Cl_3$ $[M]^+$ 307.9768; obs. mass for $C_{12}H_{11}O_3{}^{35}Cl_3$ $[M]^+$ 307.9766.

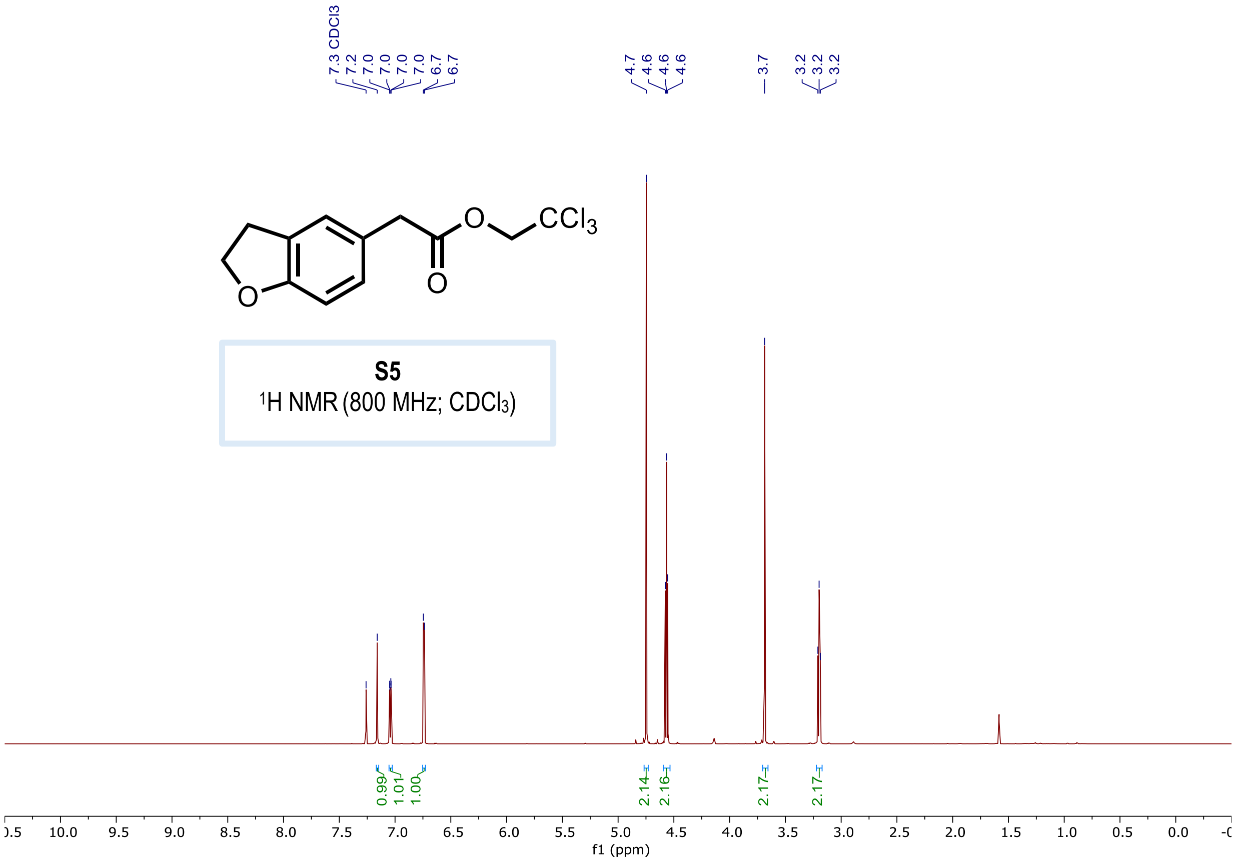
7.3 CDCl3
7.2
7.0
7.0
7.0
7.0
6.7
6.7
4.7
4.6
4.6
4.6
3.7
3.2
3.2
3.2
CCl3
O
O
O
S5
1H NMR (800 MHz; CDCl3)
0.99
1.01
1.00
2.14
2.16
2.17
2.17
f1 (ppm)

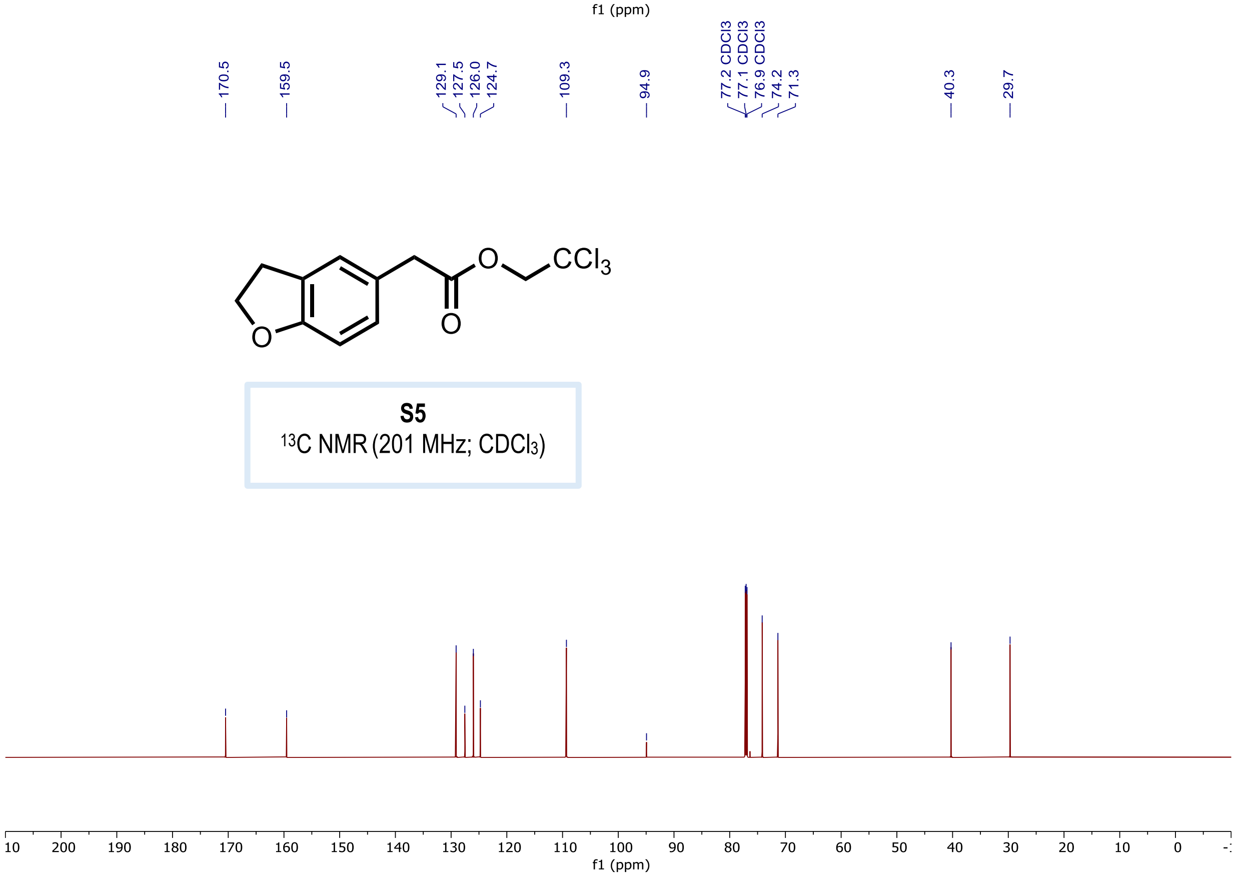
170.5
159.5
129.1
127.5
126.0
124.7
109.3
94.9
77.2 CDCl3
77.1 CDCl3
76.9 CDCl3
74.2
71.3
40.3
29.7
CCl3
O
O
O
S5
13C NMR (201 MHz; CDCl3)
f1 (ppm)

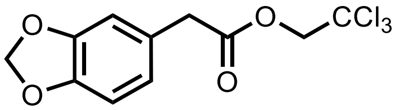

2,2,2-trichloroethyl 2-(benzo[d][1,3]dioxol-5-yl)acetate (S6)

To an RBF charged with a stir-bar was added: 2-(benzo[d][1,3]dioxol-5-yl)acetic acid (9.0 g, 1 equiv, 50 mmol), 2,2,2-trichloroethan-1-ol (9.0 g, 5.7 mL, 1.2 equiv, 60 mmol), and N,N-dimethylpyridin-4-amine (0.61 g, 0.1 equiv, 5.0 mmol) and dichloromethane (4.2 g, 0.17 L, 0.3 molar, 1 equiv, 50 mmol). To the stirred reaction mixture was added DCC (11 g, 8.6 mL, 1.1 equiv, 55 mmol) and was allowed to stir at room temperature overnight. The reaction mixture was vacuum filtered through a celite/alumina (celite on top, alumina bottom) plug, washing the filter cake with CH_2Cl_2, and the filtrate concentrated *in vacuo* at 75 °C. The dried crude was redissolved in diethyl ether, filtered through a celite/alumina (celite on top, alumina bottom) plug, washing the filter cake with diethyl ether, and the filtrate concentrated *in vacuo* at 75 °C. The crude mixture was dry-loaded onto SiO_2 and subjected to SiO_2 flash chromatography hexanes/diethyl ether (Biotage Sfär DLV 100 g column; 5% 3CV → 5 to 100% 10CV → 100% 6CV; 100 mL/min flow-rate; 210/230 nm detector). The collected fractions were concentrated *in vacuo* at 45 °C to afford 2,2,2-trichloroethyl 2-(benzo[d][1,3]dioxol-5-yl)acetate (14.7 g, 47.2 mmol, 94% yield) as a white amorphous powder.

^{1}H NMR (400 MHz, Chloroform-*d*) δ 6.82 (brd, *J* = 1.5 Hz, 1H), 6.79 – 6.73 (m, 2H), 5.95 (s, 2H), 4.75 (s, 2H), 3.67 (s, 2H).

^{13}C NMR (201 MHz, Chloroform-*d*) δ 170.1, 147.9, 147.0, 126.4, 122.7, 109.8, 108.4, 101.1, 94.9, 76.3, 74.2, 40.5.

HRMS (APCI +) calc. mass for $C_{11}H_9O_4{}^{35}Cl_3$ $[M]^+$ 309.9561; obs. mass for $C_{11}H_9O_4{}^{35}Cl_3$ $[M]^+$ 309.9559.

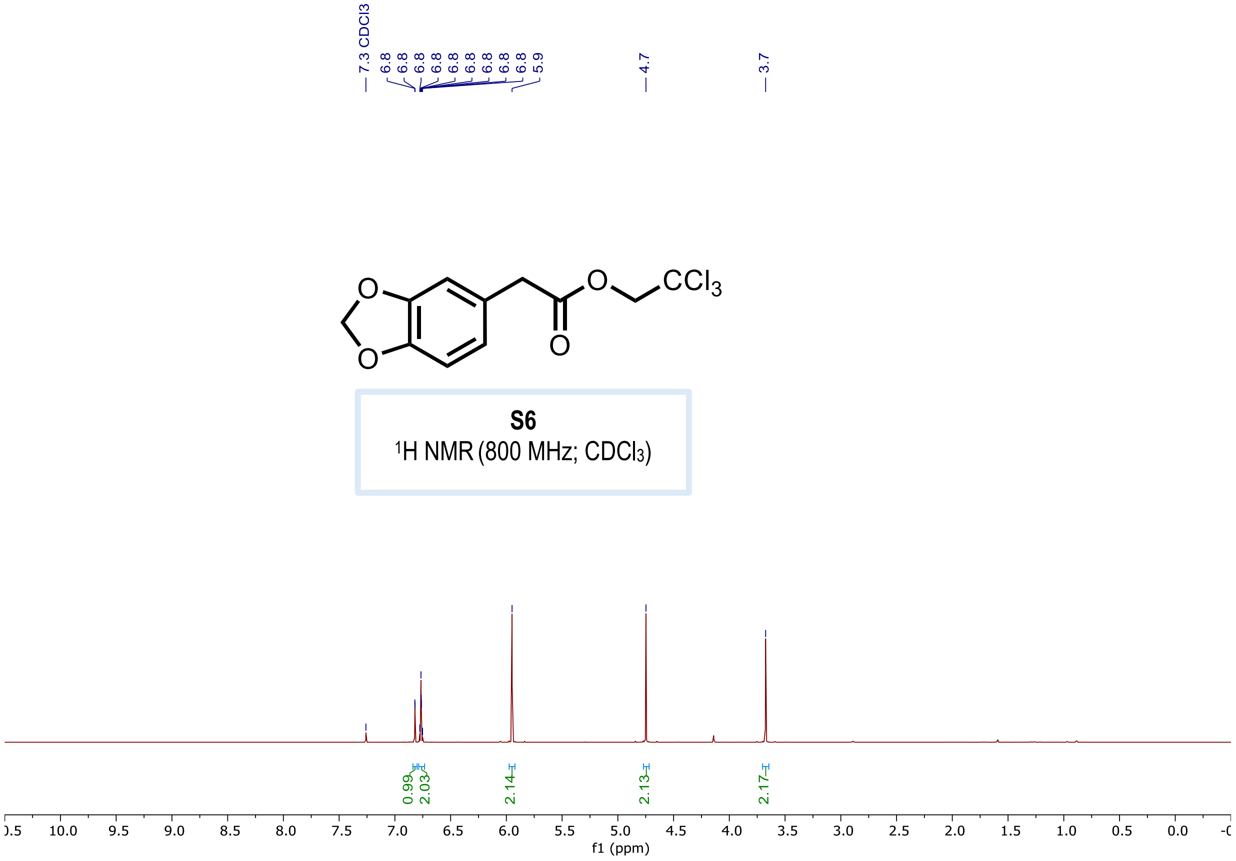

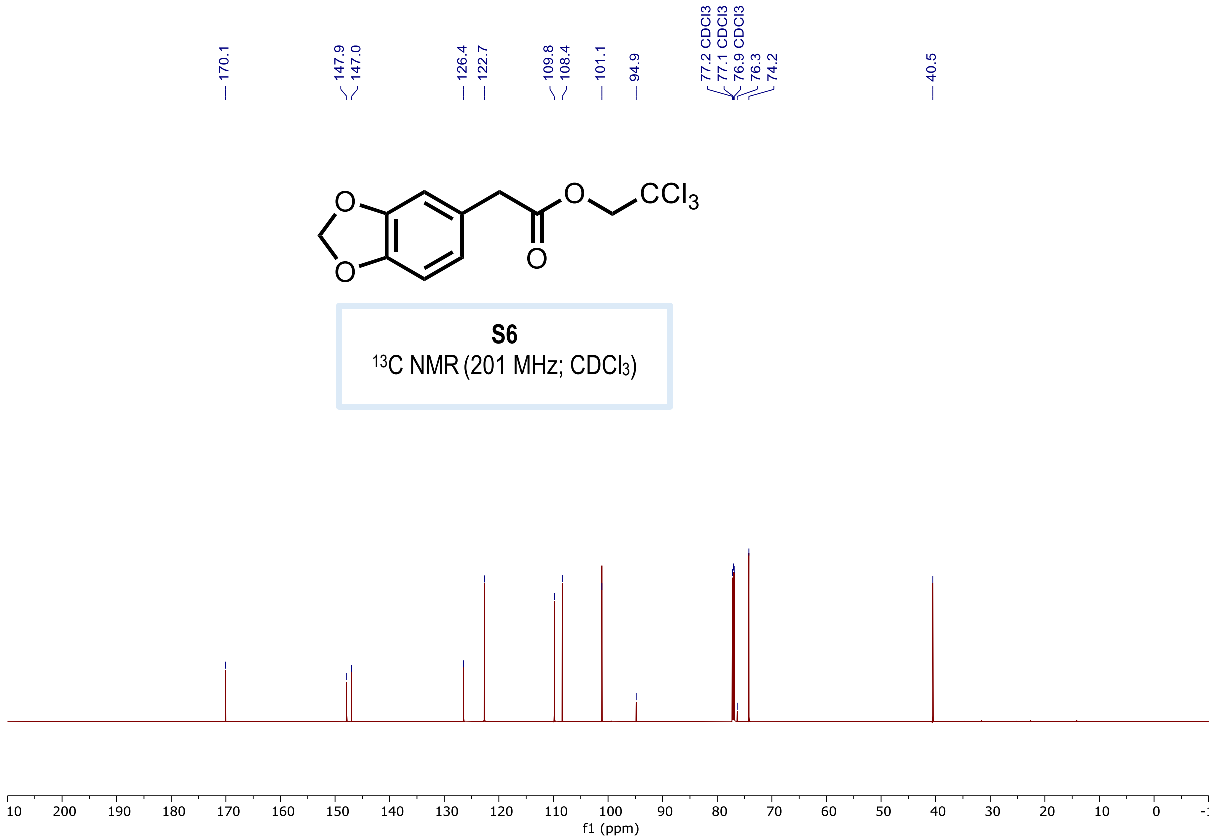

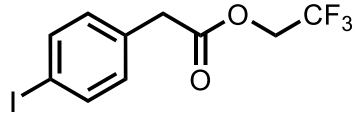

2,2,2-trifluoroethyl 2-(4-iodophenyl)acetate (S7)

To an erlenmeyer flask charged with a stir-bar was added: 2-(4-iodophenyl)acetic acid (13.10 g, 1 equiv, 50.00 mmol), 2,2,2-trifluoroethan-1-ol (6.002 g, 4.32 mL, 1.2 equiv, 60.00 mmol), and N,N-dimethylpyridin-4-amine (610.9 mg, 0.1 equiv, 5.000 mmol) and dichloromethane (4.247 g, 166.7 mL, 0.3 molar, 1 equiv, 50.00 mmol). To the stirred reaction mixture was added DCC (11.35 g, 8.565 mL, 1.1 equiv, 55.00 mmol) and was allowed to stir at room temperature overnight. The reaction mixture was vacuum filtered through a celite plug and the filtrate contracted *in vacuo*. The dried crude was redissolved in diethyl ether, filtered through a celite/alumina (celite on top, alumina bottom) plug, washing the filter cake with diethyl ether, and the filtrate concentrated *in vacuo* at 75 °C. The crude mixture was dry-loaded onto SiO_2 and subjected to SiO_2 flash chromatogaphy hexanes/diethyl ether (Biotage Sfär DLV 100 g column; 0% 3CV → 0 to 100% 10CV → 100% 6CV; 100 mL/min flow-rate; 210/230 nm detector). The collected fractions were concentrated *in vacuo* at 45 °C to afford 2,2,2-trifluoroethyl 2-(4-iodophenyl)acetate (15.2 g, 44.2 mmol, 88.4% yield) as a white amorphous powder. ^{1}H NMR spectrum is in good agreement with literature precedent.[23]

^{1}H NMR (400 MHz, Chloroform-*d*) δ 7.67 (d, *J* = 8.3 Hz, 2H), 7.04 (d, *J* = 8.3 Hz, 2H), 4.48 (q, *J* = 8.4 Hz, 2H), 3.67 (s, 2H).

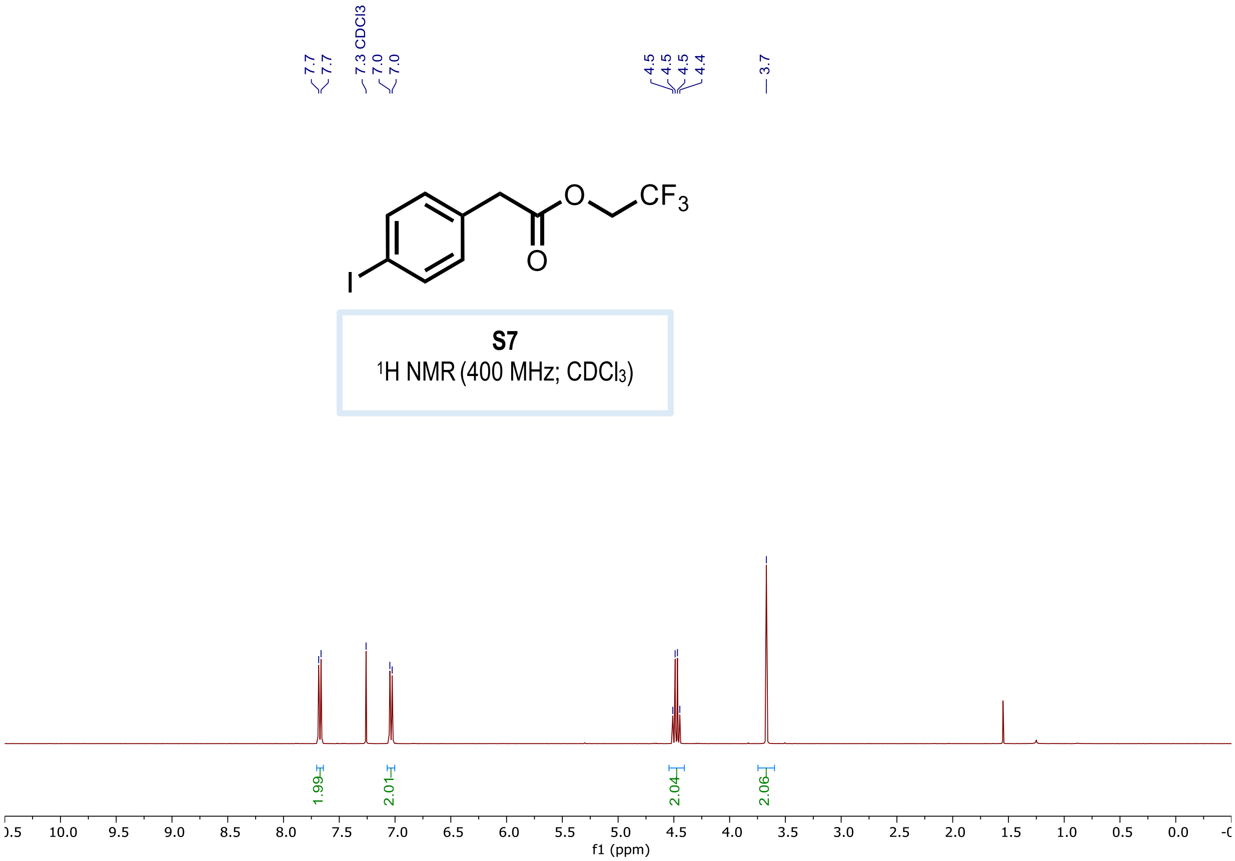

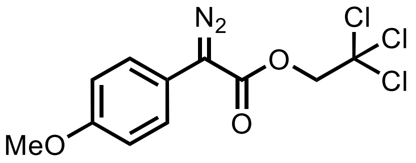

2,2,2-trichloroethyl 2-diazo-2-(4-methoxyphenyl)acetate (14)

[35.00 mmol scale with pure ester]: To a flame-dried RBF charged with a stir-bar was added: 2,2,2-trichloroethyl 2-(4-methoxyphenyl)acetate (10.41 g, 1 equiv, 35.00 mmol), *o*-NBSA (11.98 g, 1.5 equiv, 52.50 mmol), and acetonitrile (1.437 g, 116.7 mL, .30 molar, 1 equiv, 35.00 mmol). The reaction mixture was then sealed and topped with an air balloon and cooled to 0 ºC via saltwater ice bath. To the cooled reaction vessel was added DBU (21.31 g, 21.1 mL, 4 equiv, 140.0 mmol) slowly to the stirred reaction mixture at 0 ºC. The vessel was allowed to stir for 70 min at 0 °C. After the elapsed time, the reaction solution was a black-red solution. 9:1 hexanes/ether TLC showed that the SM had been fully consumed; diazo Rf is ~0.6 (an orange spot) and ester Rf is ~0.5. To the orange solution was added SiO_2 and concentrated *in vacuo* at 45 ºC to afford a SiO_2 dry-load. The SiO_2 dry-load was then subjected to SiO_2 flash chromatography isocratic hexanes (Biotage Sfär DLV 200 g column running column & 200 g loading column; 100g KP-Sil setting; 100% hexanes for 30 CV; 150 mL/min flow-rate; 210/230 nm detector; POI elutes at around 30 CVs). The collected fractions were concentrated *in vacuo* at 45 ºC to afford 2,2,2-trichloroethyl 2-diazo-2-(4-methoxyphenyl)acetate (7.3 g, 23 mmol, 64% yield) as a fluffy orange solid that has a cotton-like texture.

[150 mmol scale with crude ester]: To an erlenmeyer charged with a stir-bar was added: 2-(4-methoxyphenyl)acetic acid (49.9 g, 1 equiv, 300 mmol), N,N-dimethylpyridin-4-amine (3.67 g, 0.1 equiv, 30.0 mmol), dichloromethane (25.5 g, 1.00 L, 0.3 molar, 1 equiv, 300 mmol), 2,2,2-trichloroethan-1-ol (53.8 g, 34.5 mL, 1.2 equiv, 360 mmol). The mixture was stirred open to air at room temperature. To the stirring solution was added slowly [*careful - exothermic!*] DCC (68.1 g, 51.6 mL, 1.1 equiv, 330 mmol). After

complete addition, the sides of the erlenmeyer were rinsed with dichloromethane to get any residual material into solution. The reaction was allowed to stir overnight at room temperature.

The suspension was vacuum filtered through a plug consisting of 5 layers (from bottom to top; approx. 2 inches each layer): 1) sand, 2) neutral alumina, 3) sand, 4) celite, and 5) sand. The filtrate was then washed with saturated bicarbonate solution three times and then water three times, and the organic layer dried with $MgSO_4$, vacuum filtered through a celite plug, and the filtrate concentrated *in vacuo* at 80 ^oC.

The neat crude was cooled to room temperature and then resuspended in diethyl ether, the suspension vacuum filtered through a plug consisting of 5 layers (from bottom to top; approx. 2 inches each layer): 1) sand, 2) neutral alumina, 3) sand, 4) celite, and 5) sand, the filtrate concentrated *in vacuo* at 85 ^oC for at least 2 hours to rotovap off excess trichloroethanol resulting in 2,2,2-trichloroethyl 2-(4-methoxyphenyl)acetate (89 g, 0.27 mol, 91% yield, 91.6% purity) as a tan oil. The material was not further purified and pushed forwards to the next step.

To a flame-dried 2L RBF charged with a stir-bar was added: 2,2,2-trichloroethyl 2-(4-methoxyphenyl)acetate (48.7 g, 91.6% Wt, 1 equiv, 150 mmol), *o*-NBSA (51.3 g, 1.5 equiv, 225 mmol), and acetonitrile (6.16 g, 500 mL, .30 molar, 1 equiv, 150 mmol) and a temperature probe. The reaction mixture was then cooled below 0 ^oC (approx. -6 ^oC) via saltwater ice bath open to air. No precaution was taken to make the reaction under inert atmosphere.

To the cooled reaction vessel was added DBU (91.3 g, 90.4 mL, 4 equiv, 600 mmol) was slowly added to the stirred reaction mixture (1/4 portion of DBU was added at -6.2 ^oC which increased temp to 0 ^oC after addition; the next 1/4 portions was added after cooling the reaction mixture below -6.2 ^oC; the addition procedure was repeated until all DBU was added), maintaining the reaction below 0 ^oC via the temperature probe. After complete addition, the vessel was allowed to stir for 70 min below 0 °C. After the elapsed time, the reaction solution was a black-red solution. 9:1 hexanes/ether TLC showed that the SM had been fully consumed; diazo Rf is ~0.6 (an orange spot) and ester Rf is ~0.5.

To the orange solution was added SiO_2 and concentrated *in vacuo* at 55 ^oC to afford a SiO_2 dry-load. The SiO_2 dry-load was then subjected to SiO_2 flash chromatography hexanes/ether (Biotage Sfär DLV 350g running column & 350g loading column; 720g KP-Sil setting; 100% hexanes for 10 CV then 90% hexanes for 10 CV; 200 mL/min flow-rate; 210/230 nm detector; diazo elutes at around 6 to 10 CVs [approx. at 6000 mL of hexanes]).

The collected fractions were concentrated *in vacuo* at 55 ^oC to afford 2,2,2-trichloroethyl 2-diazo-2-(4-methoxyphenyl)acetate (26.9 g, 83.1 mmol, 55.4% yield) as a fluffy orange solid that has a cotton-like texture (cotton-like texture if material was dissolved in hexanes; when the material was dissolved in dichloromethane then concentrated, the solid material was more crystalline). ^1H NMR spectrum is in good agreement with literature precedent.[8]

^1H NMR (400 MHz, Chloroform-*d*) δ 7.40 (d, *J* = 9.0 Hz, 2H), 6.97 (d, *J* = 9.0 Hz, 2H), 4.90 (s, 2H), 3.82 (s, 3H).

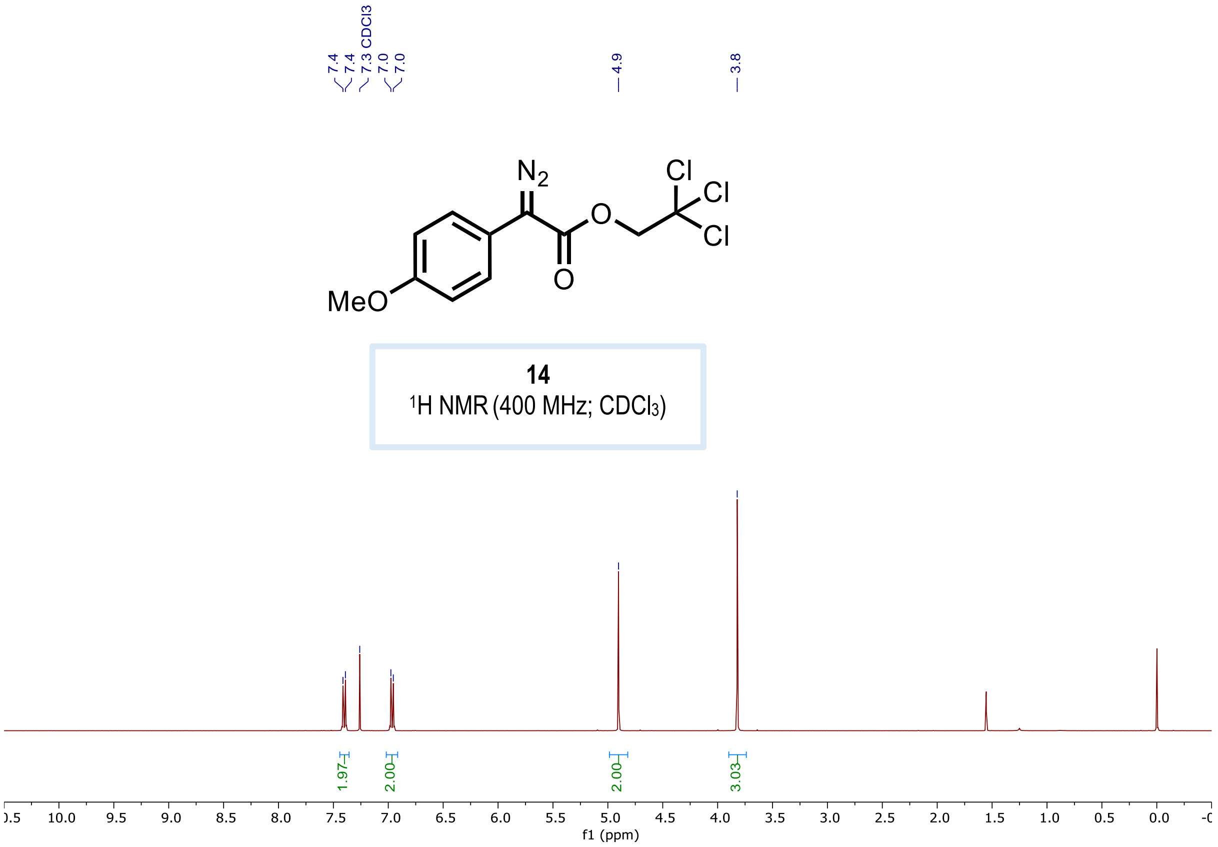

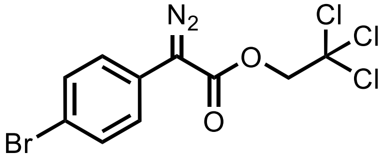

2,2,2-trichloroethyl 2-(4-bromophenyl)-2-diazoacetate (27)

To a flame-dried RBF charged with a stir-bar was added: 2,2,2-trichloroethyl 2-(4-bromophenyl)acetate (31 g, 1 equiv, 89 mmol), *o*-NBSA (31 g, 1.5 equiv, 0.13 mol), and acetonitrile (3.7 g, 0.30 L, .30 molar, 1 equiv, 89 mmol). The reaction mixture was then sealed and topped with an air balloon and cooled to 0 °C via saltwater ice bath. To the cooled reaction vessel was added DBU (54 g, 54 mL, 4 equiv, 0.36 mol) slowly to the stirred reaction mixture at 0 °C. The vessel was allowed to stir for 80 min at 0 °C. After the elapsed time, the reaction solution was a black-red solution. 9:1 hexanes/ether TLC showed that the SM had been fully consumed; product Rf (orange-yellow spot) is 0.7 and SM Rf is 0.6. To the orange solution was added SiO_2 and concentrated *in vacuo* at 45 °C to afford a SiO_2 dry-load. The SiO_2 dry-load was then subjected to SiO_2 flash chromatography isocratic hexanes (Biotage Sfär DLV 350 g column running column & 200 g loading column; SNAP Ultra 340 g setting; 100% hexanes for 30 CV; 200 mL/min flow-rate; 210/230 nm detector; POI elutes at around 30 CVs). The collected fractions were concentrated *in vacuo* at 45 °C to afford 2,2,2-trichloroethyl 2-(4-bromophenyl)-2-diazoacetate (23 g, 62 mmol, 69% yield) as an orange amorphous powder. ^1H NMR spectrum is in good agreement with literature precedent.[8]

^1H NMR (400 MHz, Chloroform-*d*) δ 7.53 (d, *J* = 8.8 Hz, 2H), 7.38 (d, *J* = 8.8, 2H), 4.91 (s, 2H).

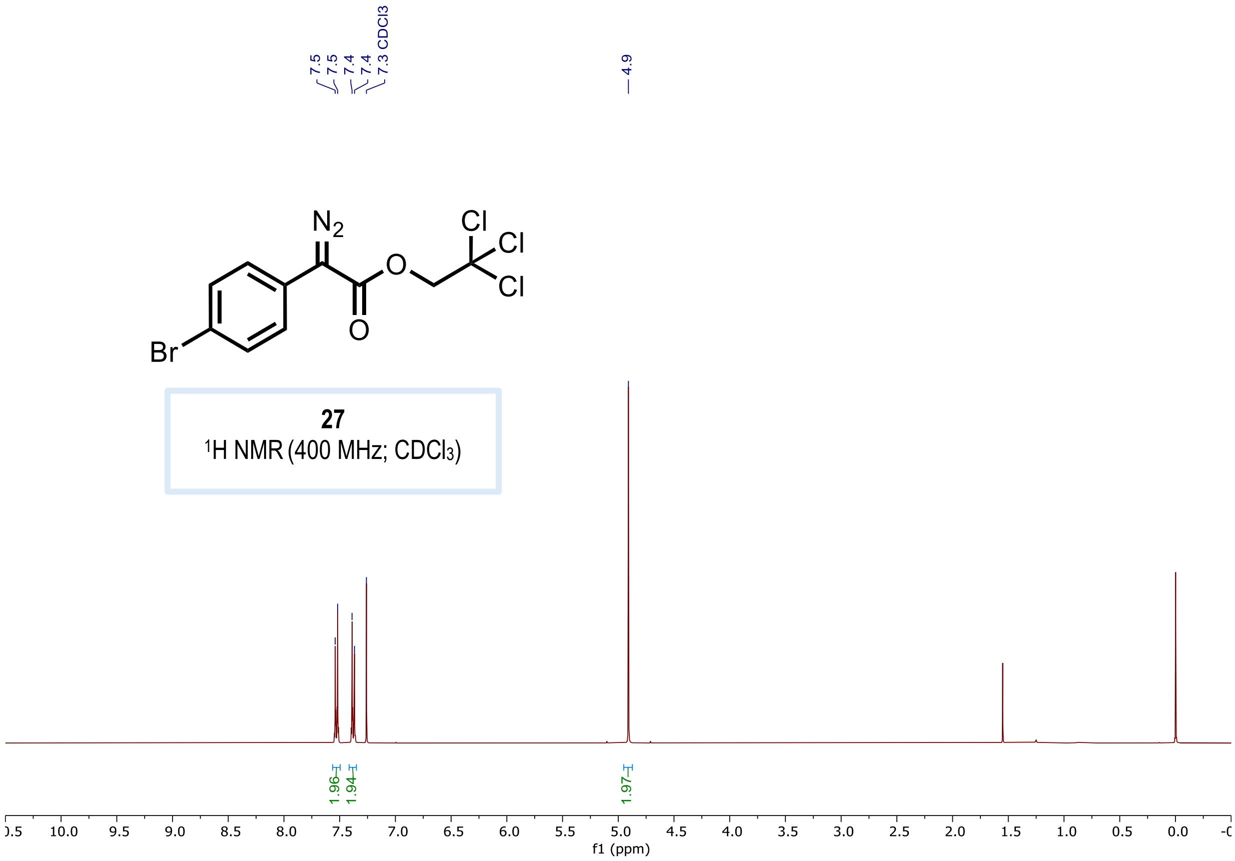

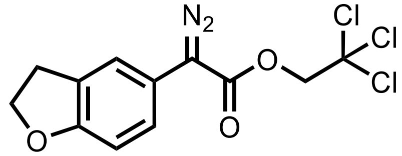

2,2,2-trichloroethyl 2-diazo-2-(2,3-dihydrobenzofuran-5-yl)acetate (S8)

To a flame-dried RBF charged with a stir-bar was added: 2,2,2-trichloroethyl 2-(2,3-dihydrobenzofuran-5-yl)acetate (6.2 g, 1 equiv, 20 mmol), *o*-NBSA (6.8 g, 1.5 equiv, 30 mmol), and acetonitrile (0.82 g, 67 mL, .30 molar, 1 equiv, 20 mmol). The reaction mixture was then sealed and topped with an air balloon and cooled to 0 °C via saltwater ice bath. To the cooled reaction vessel was added DBU (12 g, 12 mL, 4 equiv, 80 mmol) slowly to the stirred reaction mixture at 0 °C. The vessel was allowed to stir for 1 hour at 0 °C. To the orange solution was added SiO_2 and concentrated *in vacuo* at 40 °C to afford a SiO_2 dry-load. The SiO_2 dry-load was then subjected to SiO_2 flash chromatography hexanes isocratic (Biotage Sfär DLV 100 g column; 100 g KP-Sil setting;100% hexanes; 125 mL/min flow-rate; 210/254 nm detector). The collected fractions were concentrated *in vacuo* at 40 °C to afford 2,2,2-trichloroethyl 2-diazo-2-(2,3-dihydrobenzofuran-5-yl)acetate (2.8 g, 8.3 mmol, 42% yield) as an orange fluffy powder.

^{1}H NMR (800 MHz, Chloroform-*d*) δ 7.37 (brs, 1H), 7.15 (dd, *J* = 8.4, 2.0 Hz, 1H), 6.83 (d, *J* = 8.4 Hz, 1H), 4.90 (s, 2H), 4.60 (t, *J* = 8.7 Hz, 2H), 3.23 (t, *J* = 8.7 Hz, 2H).

^{13}C NMR (201 MHz, Chloroform-*d*) δ 164.1, 159.3, 128.4, 125.1, 122.2, 115.6, 109.9, 95.2, 73.8, 71.5, 29.7.

HRMS (APCI +) calc. mass for $C_{12}H_{10}O_3{}^{35}Cl_3$ $[M + H – N_2]^+$ 306.9690; obs. mass for $C_{12}H_{10}O_3{}^{35}Cl_3$ $[M + H – N_2]^+$ 306.9692.

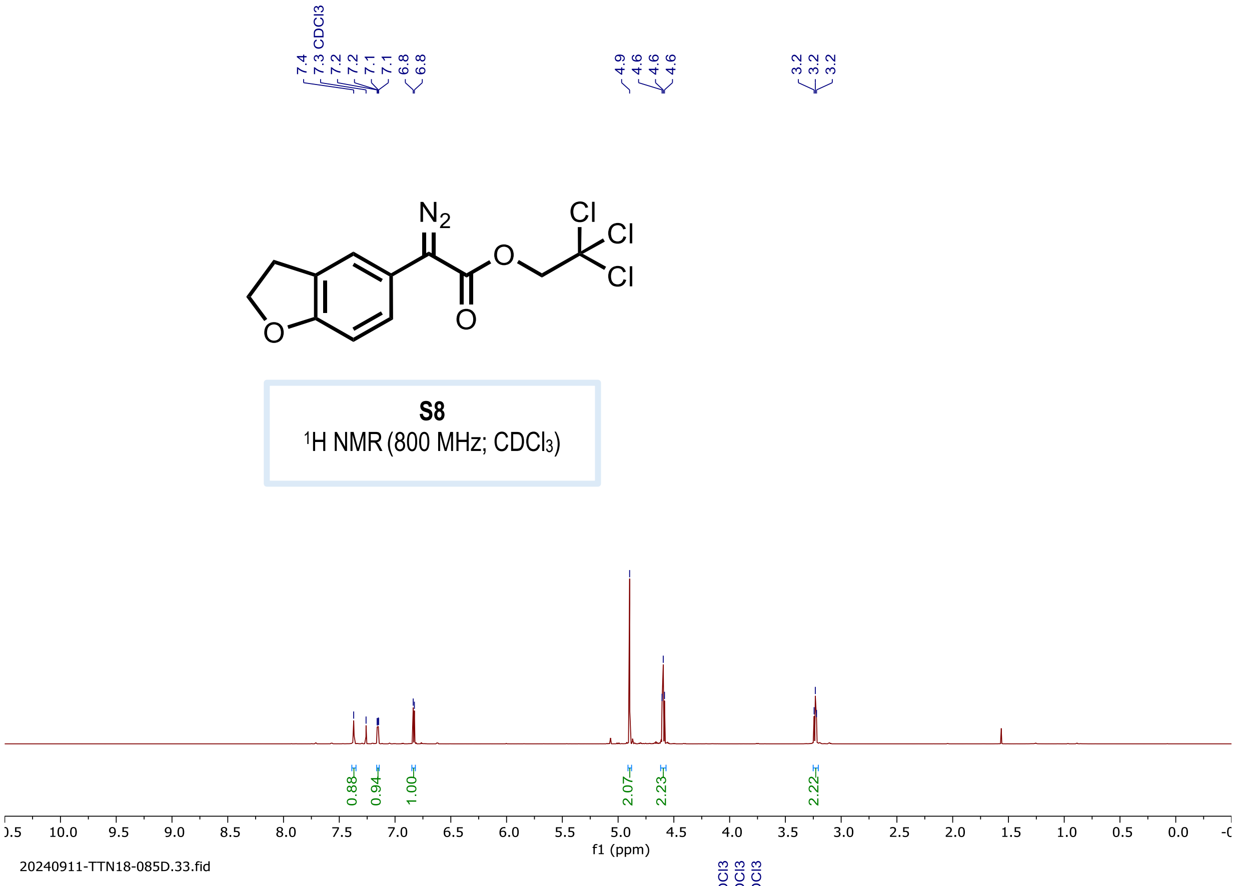

S8
^{1}H NMR (800 MHz; $CDCl_3$)

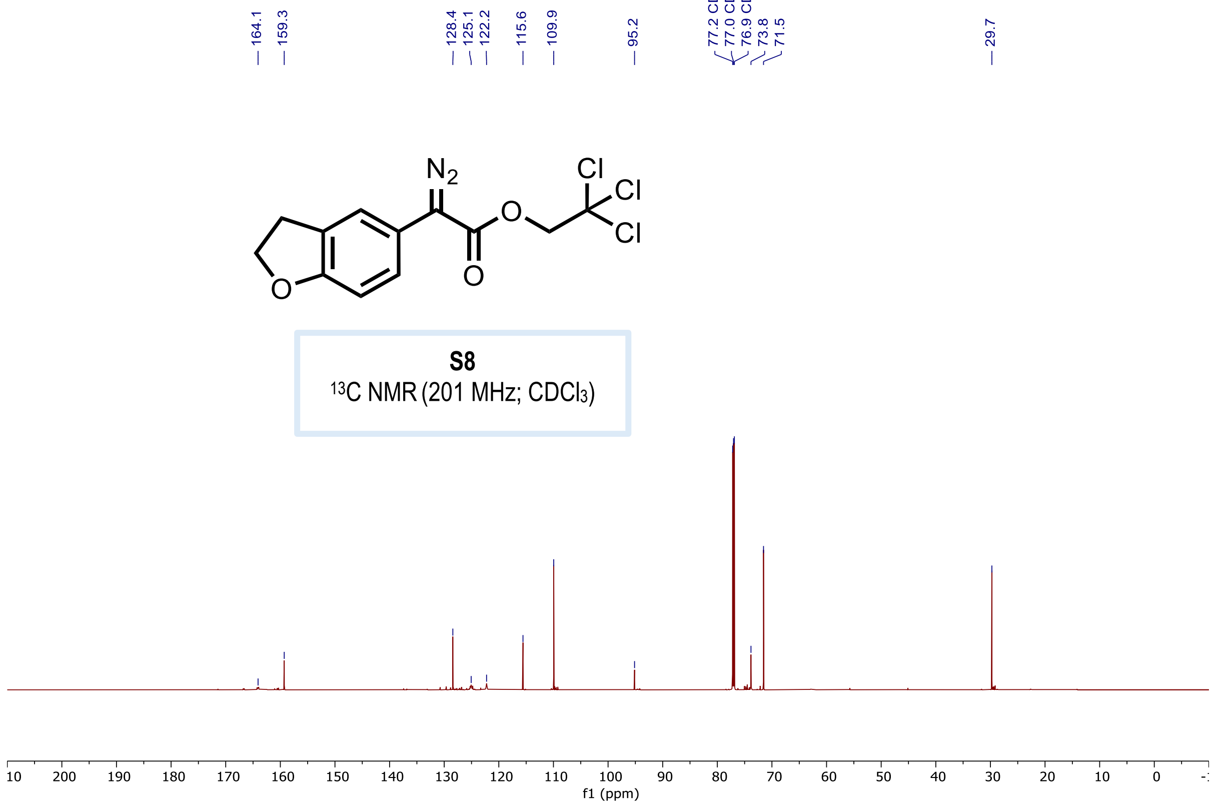

S8
^{13}C NMR (201 MHz; $CDCl_3$)

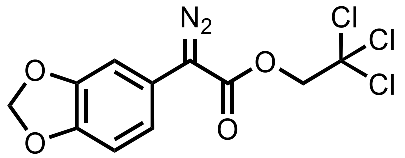

2,2,2-trichloroethyl 2-(benzo[d][1,3]dioxol-5-yl)-2-diazoacetate (S9)

To a flame-dried RBF charged with a stir-bar was added: 2,2,2-trichloroethyl 2-(benzo[d][1,3]dioxol-5-yl)acetate (6.2 g, 1 equiv, 20 mmol), *o*-NBSA (6.8 g, 1.5 equiv, 30 mmol), and acetonitrile (0.82 g, 67 mL, .30 molar, 1 equiv, 20 mmol). The reaction mixture was then sealed and topped with an air balloon and cooled to 0 ^{o}C via saltwater ice bath. To the cooled reaction vessel was added DBU (12 g, 12 mL, 4 equiv, 80 mmol) slowly to the stirred reaction mixture at 0 ^{o}C. The vessel was allowed to stir for 1 hour at 0 °C. To the orange solution was added SiO_2 and concentrated *in vacuo* at 40 ^{o}C to afford a SiO_2 dry-load. The SiO_2 dry-load was then subjected to SiO_2 flash chromatography hexanes isocratic (Biotage Sfär DLV 100 g column; 100 g KP-Sil setting; 100% hexanes; 125 mL/min flow-rate; 210/254 nm detector). The collected fractions were concentrated *in vacuo* at 40 ^{o}C to afford 2,2,2-trichloroethyl 2-(benzo[d][1,3]dioxol-5-yl)-2-diazoacetate (4.7 g, 14 mmol, 70% yield) as an orange amorphous powder.

^{1}H NMR (^{1}H NMR (800 MHz, Chloroform-*d*) δ 7.06 (d, *J* = 1.9 Hz, 1H), 6.88 (dd, *J* = 8.2, 1.9 Hz, 1H), 6.85 (d, *J* = 8.2 Hz, 1H), 5.98 (s, 2H), 4.90 (s, 2H).

^{13}C NMR (201 MHz, Chloroform-*d*) δ 163.7, 148.5, 146.5, 118.1, 117.7, 108.9, 105.9, 101.4, 95.1, 73.9.

HRMS (APCI +) calc. mass for $C_{11}H_8O_4{}^{35}Cl_3$ $[M + H – N_2]^+$ 308.9483; obs. mass for $C_{11}H_8O_4{}^{35}Cl_3$ [M + H – N_2]+ 308.9482.

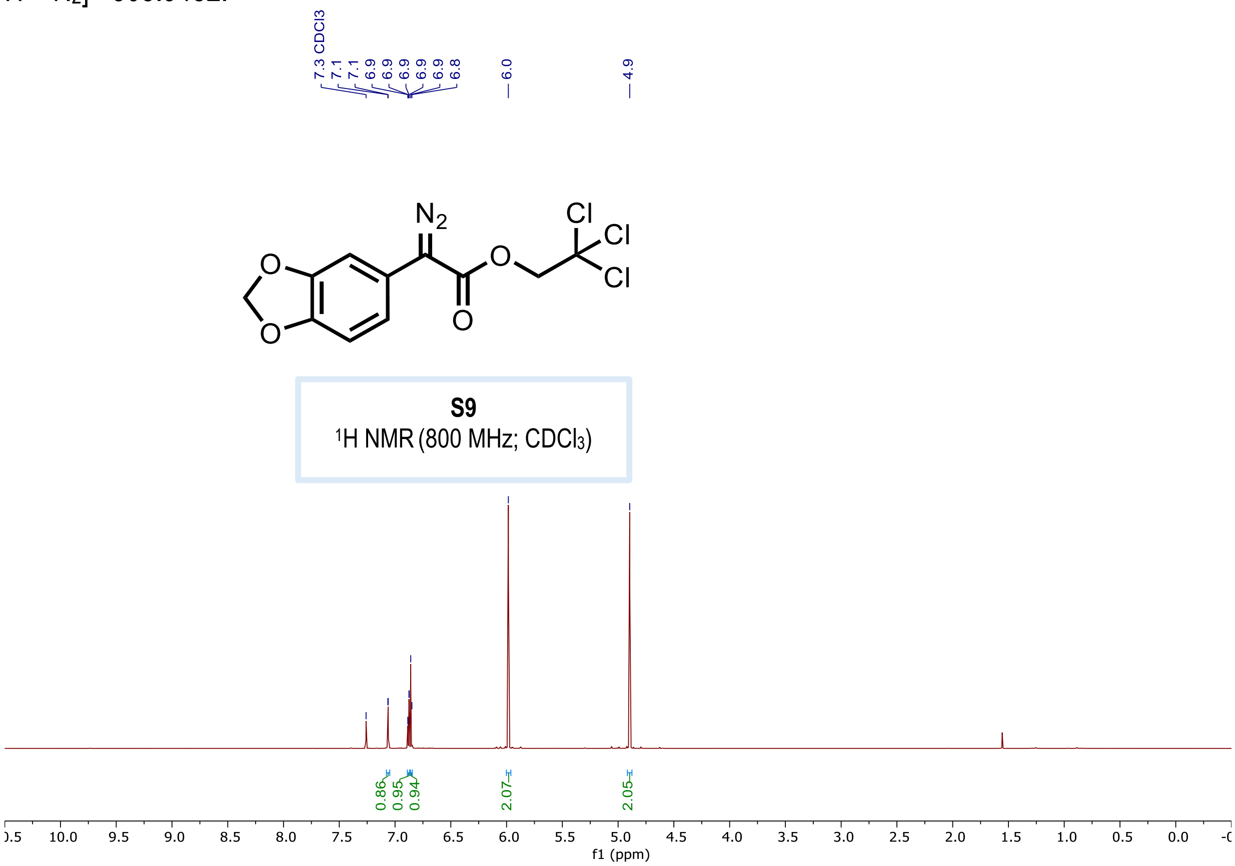

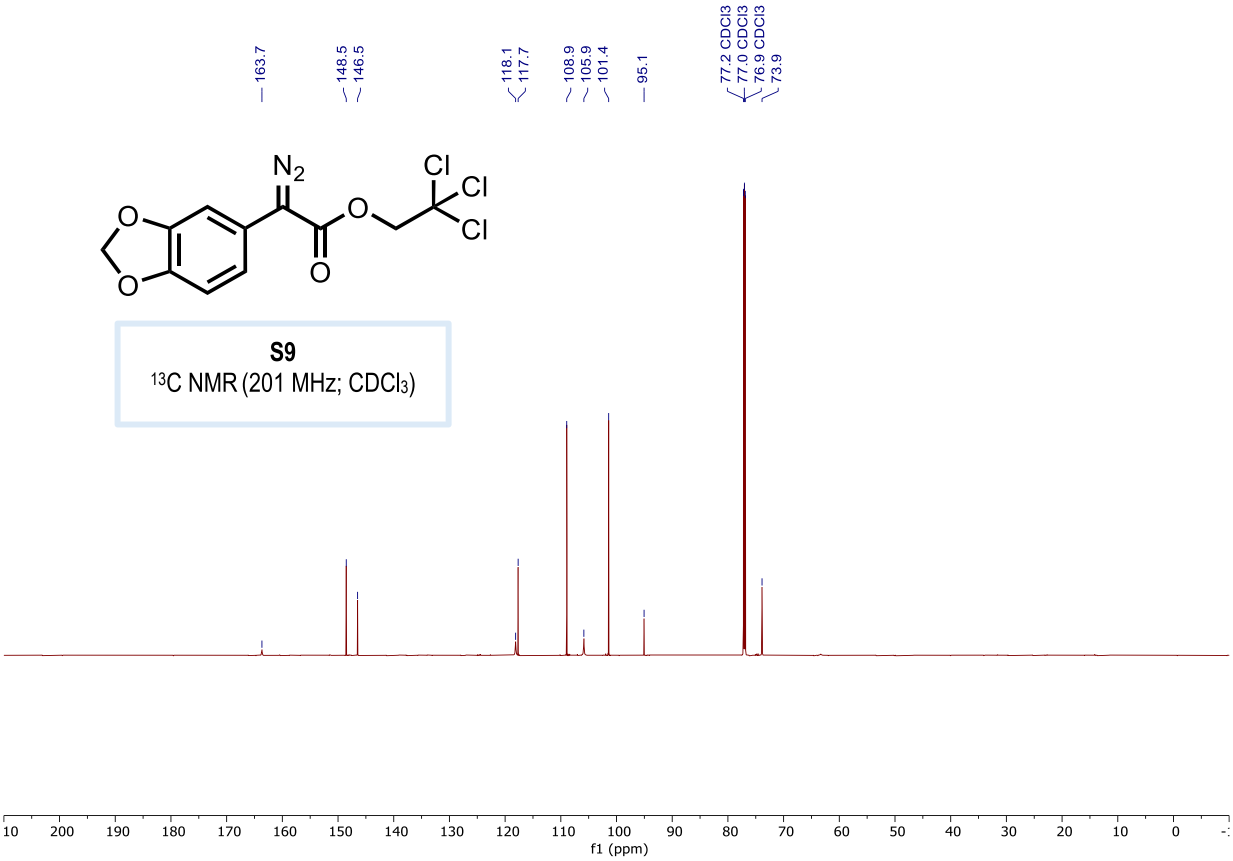

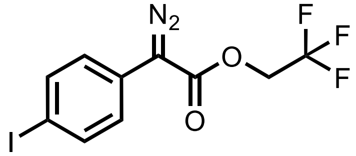

2,2,2-trifluoroethyl 2-diazo-2-(4-iodophenyl)acetate (29)

To a flame-dried RBF charged with a stir-bar was added: 2,2,2-trifluoroethyl 2-(4-iodophenyl)acetate (14 g, 1 equiv, 40 mmol), *o*-NBSA (14 g, 1.5 equiv, 60 mmol), and acetonitrile (1.6 g, 0.13 L, .30 molar, 1 equiv, 40 mmol). The reaction mixture was then sealed and topped with an air balloon and cool to 0 ^{o}C via saltwater ice bath. To the cooled reaction vessel was added DBU (24 g, 24 mL, 4 equiv, 0.16 mol) slowly to the stirred reaction mixture at 0 ^{o}C. The vessel was allowed to stir for 1 hour at 0 °C. To the orange solution was added SiO_2 and concentrated *in vacuo* at 40 ^{o}C to afford a SiO_2 dry-load. The SiO_2 dry-load was then subjected to SiO_2 flash chromatography hexanes isocratic (Biotage Sfär DLV 200 g column; 350 g KP-Sil setting; 100% hexanes 15 CV; 150 mL/min flow-rate; 210/254 nm detector). The collected fractions were concentrated *in vacuo* at 40 ^{o}C to afford 2,2,2-trifluoroethyl 2-diazo-2-(4-iodophenyl)acetate (12.3 g, 33.2 mmol, 83% yield) as a yellow-orange fluffy powder. ^{1}H NMR spectrum is in good agreement with literature precedent.[23]

^{1}H NMR (400 MHz, Chloroform-*d*) δ 7.72 (d, *J* = 8.7 Hz, 2H), 7.22 (d, *J* = 8.7 Hz, 2H), 4.65 (q, *J* = 8.3 Hz, 2H).

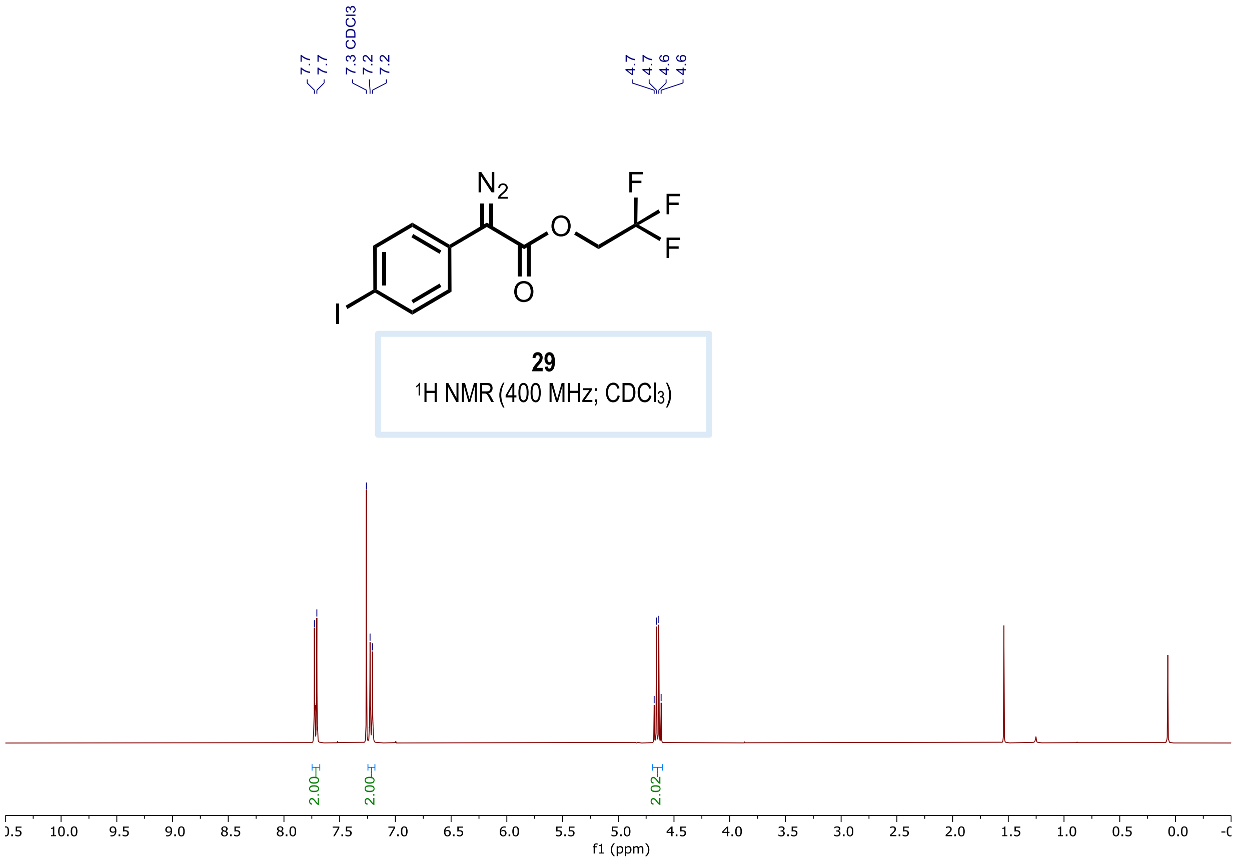

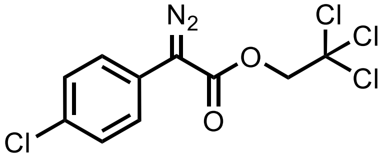

2,2,2-trichloroethyl 2-(4-chlorophenyl)-2-diazoacetate (S10)

To an erlenmeyer flask charged with a stir-bar was added: 2-(4-chlorophenyl)acetic acid (8.5 g, 1 equiv, 50 mmol), 2,2,2-trichloroethan-1-ol (9.0 g, 5.7 mL, 1.2 equiv, 60 mmol), and N,N-dimethylpyridin-4-amine (0.61 g, 0.1 equiv, 5.0 mmol) and dichloromethane (4.2 g, 0.17 L, 0.3 molar, 1 equiv, 50 mmol). To the stirred reaction mixture was added DCC (11 g, 8.6 mL, 1.1 equiv, 55 mmol) and was allowed to stir at room temperature overnight. The reaction mixture was vacuum filtered through a celite plug and the filtrate concentrated *in vacuo*. The neat material was resuspended in hexanes and filtered through a celite plug and then the filtrate concentrated *in vacuo* at 65 ^oC to afford 2,2,2-trichloroethyl 2-(4-chlorophenyl)acetate as a yellow oil. The material was pushed to the next step without further purification.

To a flame-dried RBF charged with a stir-bar was added: 2,2,2-trichloroethyl 2-(4-chlorophenyl)acetate (15 g, 1 equiv, 50 mmol), *o*-NBSA (17 g, 1.5 equiv, 75 mmol), and acetonitrile (2.1 g, 0.17 L, .30 molar, 1 equiv, 50 mmol). The reaction mixture was then sealed and topped with an air balloon and cool to 0 ^oC via saltwater ice bath. To the cooled reaction vessel was added DBU (30 g, 30 mL, 4 equiv, 0.20 mol) slowly to the stirred reaction mixture at 0 ^oC. The vessel was allowed to stir for 80 min at 0 °C. To the orange solution was added SiO_2 and concentrated *in vacuo* at 45 ^oC to afford a SiO_2 dry-load. The SiO_2 dry-load was then subjected to SiO_2 flash chromatography isocratic hexanes (Biotage Sfär DLV 200 g column running column & 200 g loading column; 100 g KP-Sil setting; 100% hexanes for 30 CV; 150 mL/min flow-rate; 210/230 nm detector; POI elutes at around 30 CVs). The collected fractions were concentrated *in vacuo* at 45 ^oC to afford 2,2,2-trichloroethyl 2-(4-chlorophenyl)-2-diazoacetate (13 g, 40

mmol, 79% yield) as an orange amorphous powder. ^{1}H NMR spectrum is in good agreement with literature precedent.[4]
^{1}H NMR (400 MHz, Chloroform-*d*) δ 7.44 (d, *J* = 8.8 Hz, 2H), 7.38 (d, *J* = 8.8 Hz, 2H), 4.91 (s, 2H).

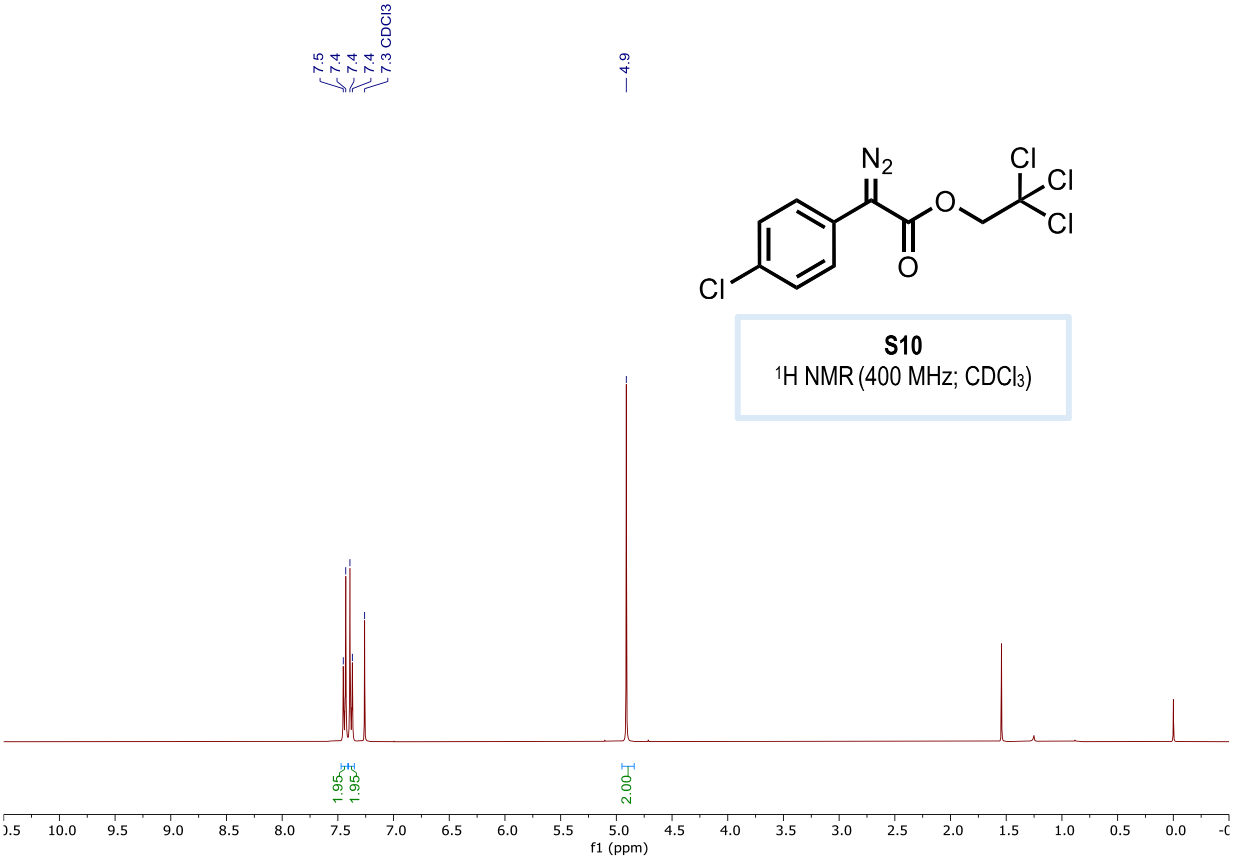

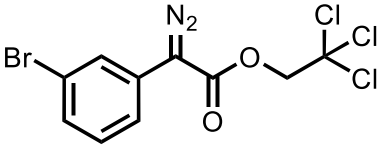

2,2,2-trichloroethyl 2-(3-bromophenyl)-2-diazoacetate (S11)

To a flame-dried RBF charged with a stir-bar was added: 2,2,2-trichloroethyl 2-(3-bromophenyl)acetate (3.464 g, 1 equiv, 10.00 mmol), 4-acetamidobenzenesulfonyl azide (2.883 g, 1.2 equiv, 12.00 mmol), and acetonitrile (410.5 mg, 33.33 mL, 0.30 molar, 1 equiv, 10.00 mmol). The reaction mixture was sparged with N_2 for at least 10 minutes and then was sealed with a N_2 balloon and cool to 0 ^{o}C via salt-ice water bath. The cooled mixture was stirred at this temp for at least 10 minutes. DBU (1.827 g, 1.81 mL, 1.2 equiv, 12.00 mmol) was slowly added to the stirred reaction mixture at 0 ^{o}C. The reaction was allowed to react at 0 ^{o}C for 1.5 hours. The reaction mixture is then quenched with NH_4Cl solution, diluted with diethyl ether, extracted with diethyl ether three times, the organic layer dried with $MgSO_4$, filtered, and then the filtrate concentrated *in vacuo* at 40 ^{o}C. The crude was dry-loaded onto SiO_2 and was subjected to SiO_2 flash chromatography isocratic hexanes (Biotage Sfär DLV 25 g column; 100 g SNAP KP-Sil setting; 100 mL/min flow-rate; 210/230 nm detector). The collected fractions were concentrated *in vacuo* at 40 ^{o}C to afford 2,2,2-trichloroethyl 2-(3-bromophenyl)-2-diazoacetate (1.18 g, 3.17 mmol, 31.7% yield) as an orange oil. ^{1}H NMR spectrum is in good agreement with literature precedent.[4]
^{1}H NMR (400 MHz, Chloroform-*d*) δ 7.72 (t, *J* = 1.5 Hz, 1H), 7.41 (dt, *J* = 7.9, 1.5 Hz, 1H), 7.36 (dt, *J* = 7.9, 1.5 Hz, 1H), 7.31 – 7.26 (m, 1H), 4.93 (s, 2H).

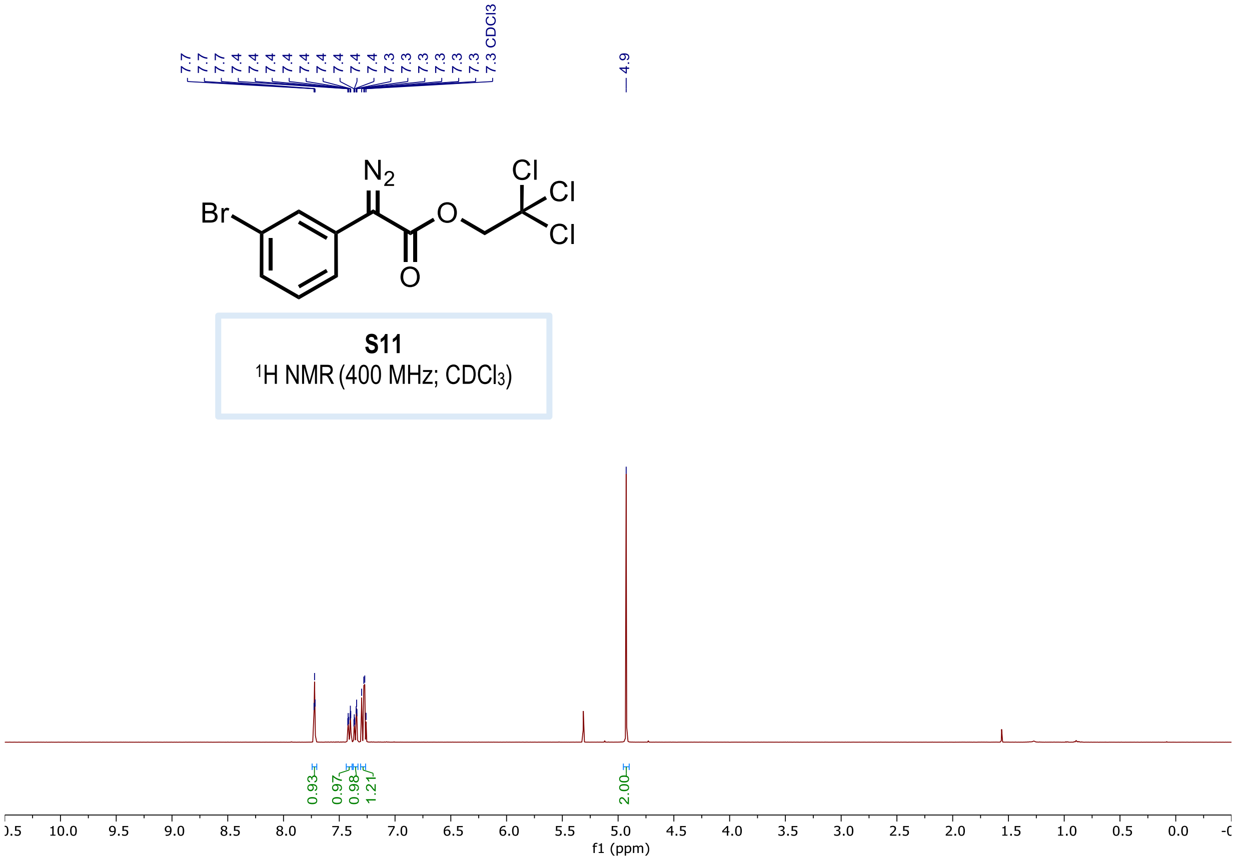

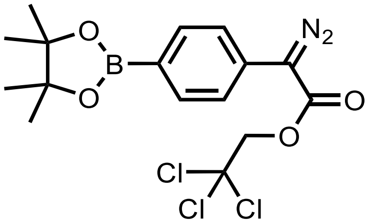

2,2,2-trichloroethyl 2-diazo-2-(4-(4,4,5,5-tetramethyl-1,3,2-dioxaborolan-2-yl)phenyl)acetate (S12)

To a flame-dried RBF charged with a stir-bar was added in the following order: triphenylphosphine (131 mg, 0.10 equiv, 500 µmol), silver carbonate (689 mg, 0.50 equiv, 2.50 mmol), 2-(4-iodophenyl)-4,4,5,5-tetramethyl-1,3,2-dioxaborolane (2.14 g, 1.3 equiv, 6.50 mmol), and then lastly, tetrakis(triphenylphosphine)palladium(0) (289 mg, 0.05 equiv, 250 µmol). The reaction vessel was sealed and backfilled with N_2 three times. Subsequently, SPS-grade argon sparged toluene (461 mg, 20.0 mL, 0.25 molar, 1 equiv, 5.00 mmol) was added to the reaction mixture. Subsequently, to the reaction mixture was added 2,2,2-trichloroethyl 2-diazoacetate (1.09 g, 742 µL, 1 equiv, 5.00 mmol) and then triethylamine (658 mg, 906 µL, 1.3 equiv, 6.50 mmol). The reaction mixture was stirred vigorously at 25 °C for 18 hours. After the elapsed time, the reaction mixture was filtered through a celite plug, to the filtrate was added SiO_2, and the filtrate concentrated *in vacuo* at 45 °C to afford the crude mixture dry-loaded onto SiO_2. The dry-load was subjected to SiO_2 flash chromatography hexanes/diethyl ether (Biotage Sfär DLV 100 g column; 0% 5CV →1% 5CV → 2% 5CV → 3% 5CV → 4% 5CV → 5% 15CV; 150 mL/min flow-rate; 210/254 nm detector; the POI eluted at 4 - 5% diethyl ether). The collected fractions were concentrated *in vacuo* at 45 °C to afford 2,2,2-trichloroethyl 2-diazo-2-(4-(4,4,5,5-tetramethyl-1,3,2-dioxaborolan-2-yl)phenyl)acetate (1.27 g, 3.03 mmol, 60.5% yield) as an orange solid. ^{1}H NMR spectrum is in good agreement with literature precedent.[4]

^{1}H NMR (400 MHz, Chloroform-*d*) δ 7.83 (d, *J* = 8.5 Hz, 2H), 7.50 (d, *J* = 8.5 Hz, 2H), 4.91 (s, 2H), 1.35 (s, 12H).

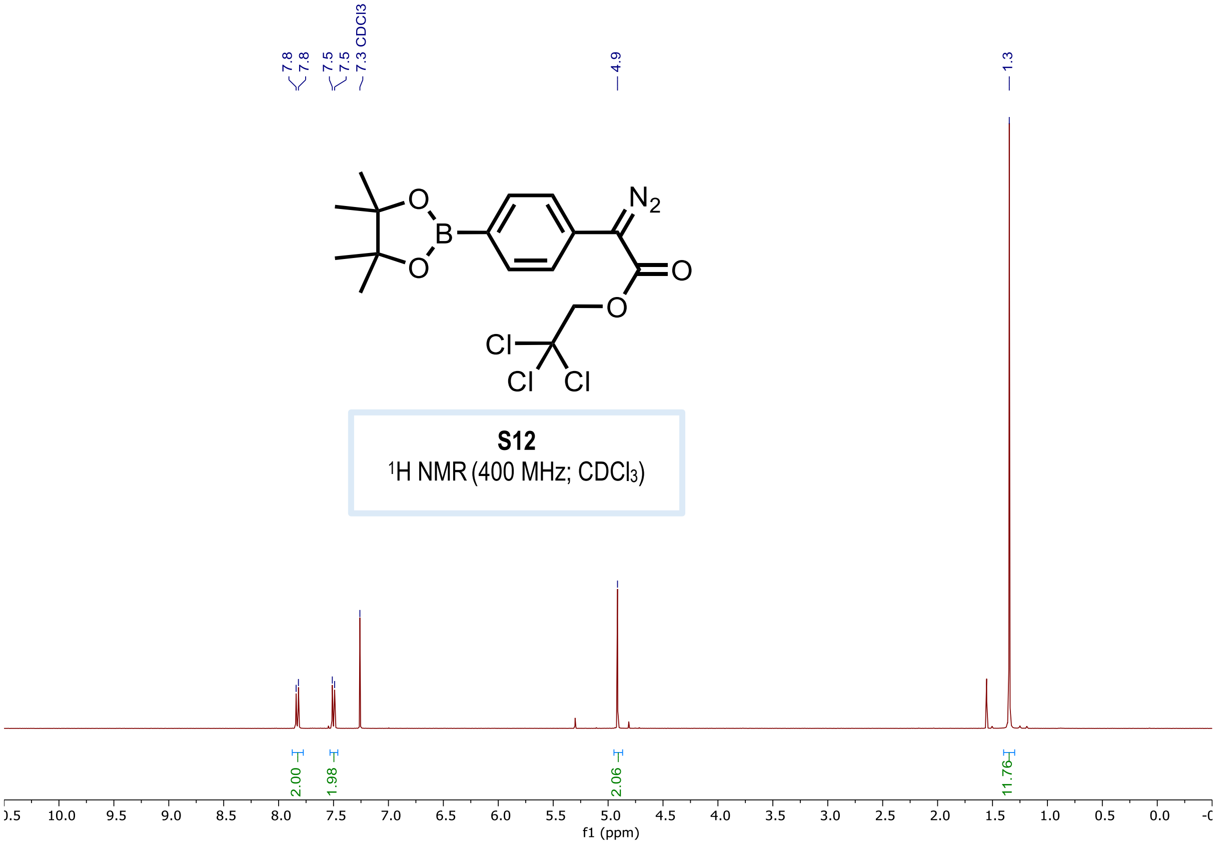

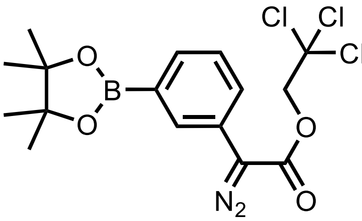

2,2,2-trichloroethyl 2-diazo-2-(3-(4,4,5,5-tetramethyl-1,3,2-dioxaborolan-2-yl)phenyl)acetate (S13)

To a flame-dried RBF charged with a stir-bar was added in the following order: triphenylphosphine (131 mg, 0.10 equiv, 500 µmol), silver carbonate (689 mg, 0.50 equiv, 2.50 mmol), 2-(3-iodophenyl)-4,4,5,5-tetramethyl-1,3,2-dioxaborolane (2.14 g, 1.3 equiv, 6.50 mmol), and then lastly, tetrakis(triphenylphosphine)palladium(0) (289 mg, 0.05 equiv, 250 µmol). The reaction vessel was sealed and backfilled with N_2 three times. Subsequently, SPS-grade argon sparged toluene (461 mg, 20.0 mL, 0.25 molar, 1 equiv, 5.00 mmol) was added to the reaction mixture. Subsequently, to the reaction mixture was added 2,2,2-trichloroethyl 2-diazoacetate (1.09 g, 742 µL, 1 equiv, 5.00 mmol) and then triethylamine (658 mg, 906 µL, 1.3 equiv, 6.50 mmol). The reaction mixture was stirred vigorously at 25 °C for 18 hours. After the elapsed time, the reaction mixture was filtered through a celite plug, to the filtrate was added SiO_2, and the filtrate concentrated *in vacuo* at 45 °C to afford the crude mixture dry-loaded onto SiO_2. The dry-load was subjected to SiO_2 flash chromatography hexanes/diethyl ether (Biotage Sfär DLV 100 g column; 0% 5CV → 1% 5CV → 2% 5CV → 3% 5CV → 4% 5CV → 5% 15CV; 150 mL/min flow-rate; 210/254 nm detector; the POI eluted at 4 - 5% diethyl ether). The collected fractions were concentrated *in vacuo* at 45 °C to afford 2,2,2-trichloroethyl 2-diazo-2-(3-(4,4,5,5-tetramethyl-1,3,2-dioxaborolan-2-yl)phenyl)acetate (1.2 g, 2.9 mmol, 57% yield) as an orange solid.

^{1}H NMR (400 MHz, Chloroform-*d*) δ 7.82 (brm, 1H), 7.69 (brd, *J* = 7.6 Hz, 1H), 7.66 (dt, *J* = 7.6, 1.1 Hz, 2H), 7.42 (t, *J* = 7.6 Hz, 1H), 4.92 (s, 2H), 1.34 (s, 12H).
^{13}C NMR (201 MHz, Chloroform-*d*) δ 132.8, 128.5, 127.4, 124.1, 95.1, 84.0, 73.9, 24.9.
HRMS (APCI +) calc. mass for $C_{16}H_{18}O_{4}{}^{10}B^{35}Cl_{3}$ $[M - N_2]^+$ 389.0395; obs. mass for $C_{16}H_{18}O_{4}{}^{10}B^{35}Cl_{3}$ $[M - N_2]^+$ 389.0395.

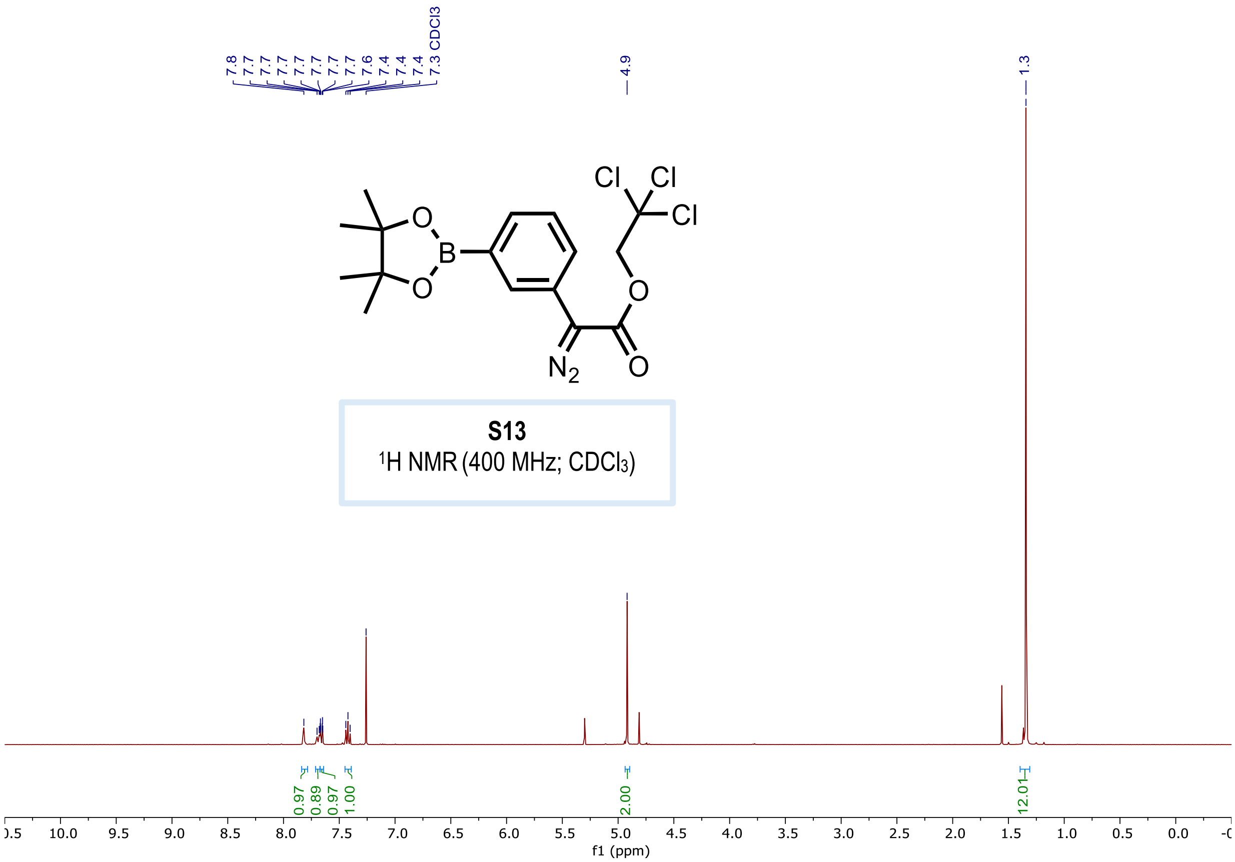

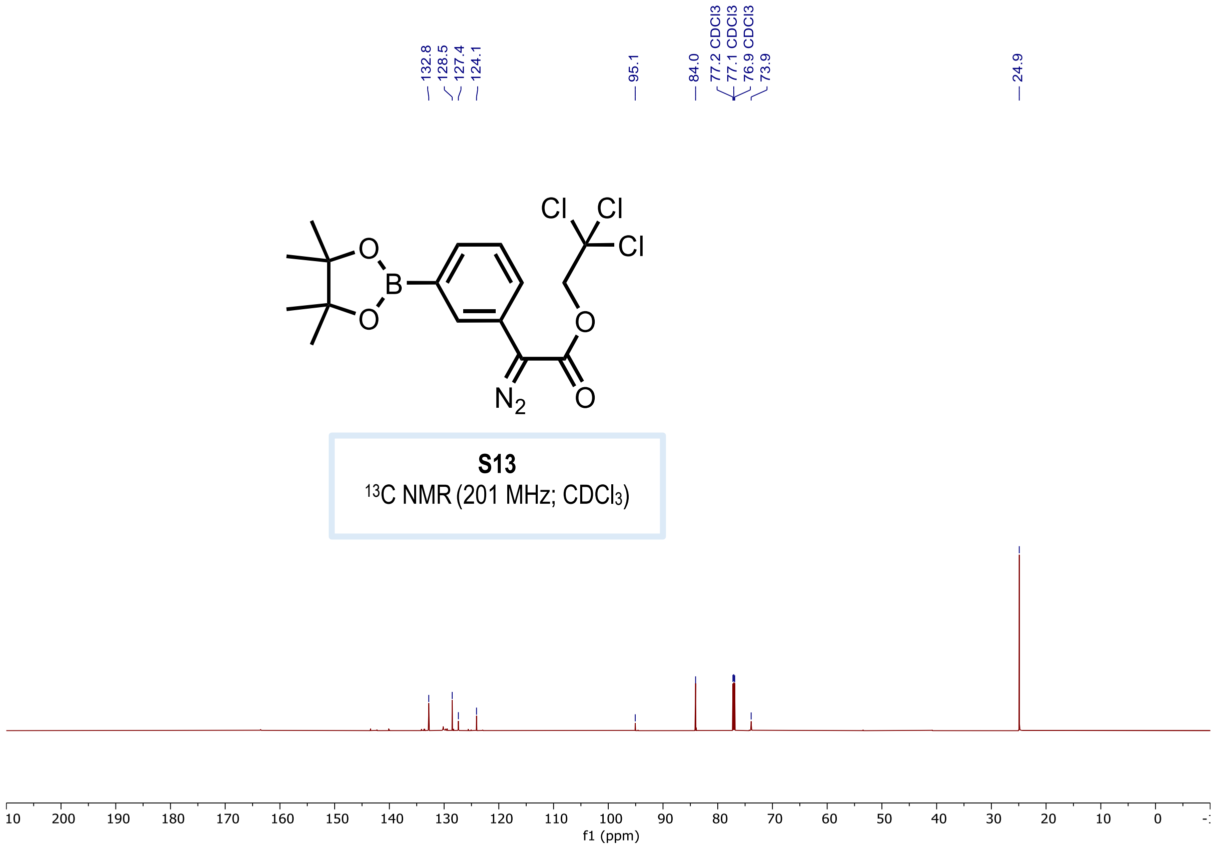

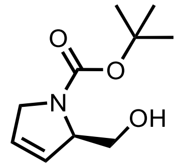

tert-butyl (R)-2-(hydroxymethyl)-2,5-dihydro-1H-pyrrole-1-carboxylate (S14)

To a flame-dried 250 mL RBF charged with a stir-bar was added 1-(tert-butyl) 2-methyl (R)-2,5-dihydro-1H-pyrrole-1,2-dicarboxylate (2.5655 g, 1 equiv, 11.289 mmol) in THF (814.0 mg, 33.900 mL, 0.333 molar, 1 equiv, 11.289 mmol). The reaction solution was then cooled to -78 °C via dry-ice/acetone bath and allowed to stir for at least 10 minutes. To the cooled solution was added DIBAL-H in CH_2Cl_2 (4.8165 g, 33.866 mL, 1 molar, 3 equiv, 33.866 mmol) drop-wise. After complete addition, the reaction mixture was allowed to stir at -78 °C for 15 min. Subsequently, the reaction mixture was warmed to room temperature and allowed to stir for an additional 3 h. After the elapsed time, the reaction mixture was quenched with saturated sodium potassium tartrate solution and diluted with CH_2Cl_2. The bilayer was allowed to stir vigorously overnight until the aqueous layer was not opaque. The aqueous layer was decanted and the organic layer washed with DI water three times, the organic layer dried with $MgSO_4$, filtered, and the filtrate concentrated *in vacuo* to afford tert-butyl (R)-2-(hydroxymethyl)-2,5-dihydro-1H-pyrrole-1-carboxylate (1.6 g, 8.0 mmol, 71% yield) as a golden-yellow oil. The crude was subjected without further purification and characterization to the next step. ^{1}H NMR spectrum is in good agreement with literature precedent.[24]
^{1}H NMR (400 MHz, Chloroform-*d*; mixture of rotamers) δ 5.84 – 5.77 (m, 1H), 5.64 – 5.57 (m, 1H), 4.74 (ddq, *J* = 7.7, 5.5, 2.1 Hz, 1H), 4.18 (dd, *J* = 15.7, 2.1 Hz, 1H), 4.07 (ddt, *J* = 15.7, 5.5, 2.1 Hz, 1H), 3.77 (brt, *J* = 11.4 Hz, 1H), 3.56 (dd, *J* = 11.4, 7.7 Hz, 1H), 1.48 (s, 9H).

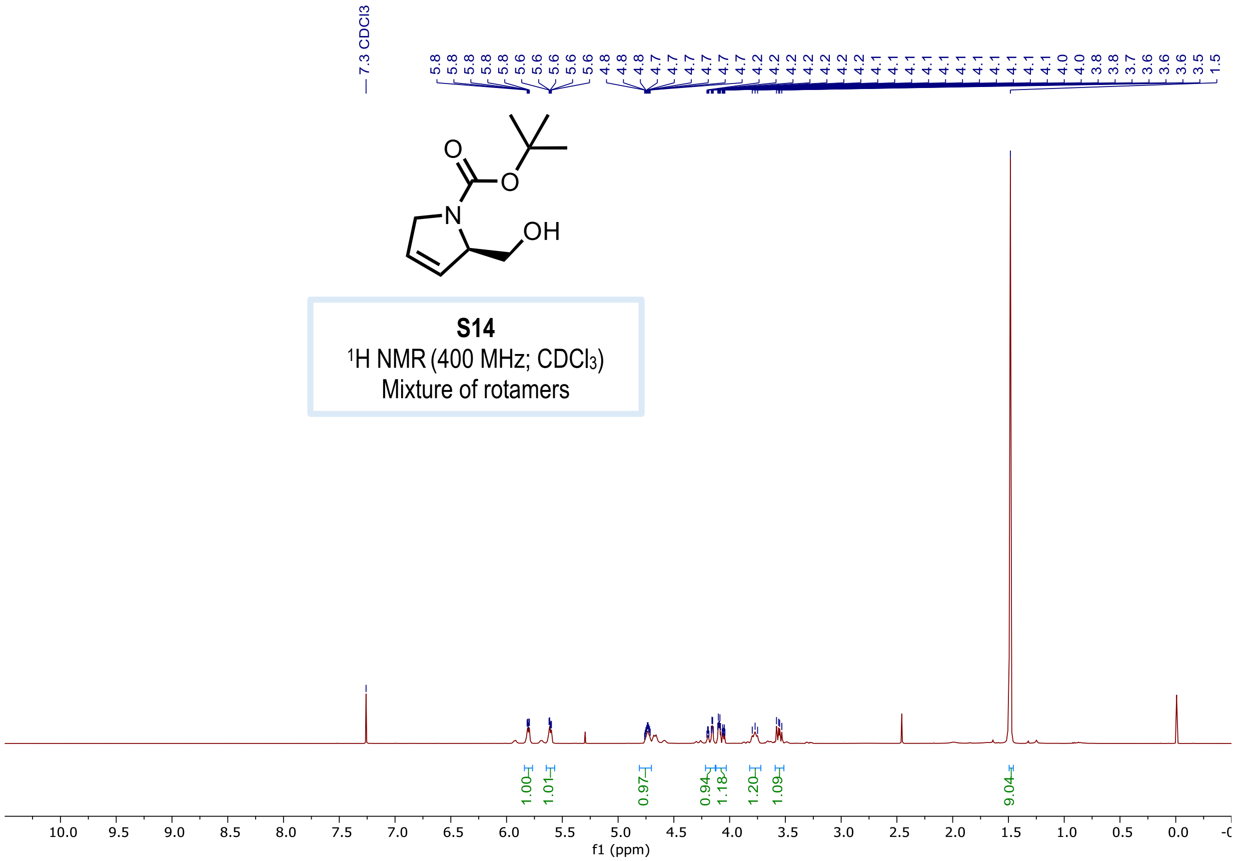

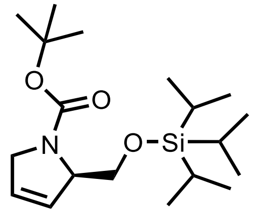

tert-butyl (R)-2-(((triisopropylsilyl)oxy)methyl)-2,5-dihydro-1H-pyrrole-1-carboxylate (S15)

To a flame-dried 100 mL RBF charged with a stir-bar was added tert-butyl (R)-2-(hydroxymethyl)-2,5-dihydro-1H-pyrrole-1-carboxylate (1.6 g, 1 equiv, 8.0 mmol), DMAP (0.20 g, 0.2 equiv, 1.6 mmol), CH_2Cl_2 (16 mL) and then triethylamine (0.98 g, 1.3 mL, 1.2 equiv, 9.6 mmol) and chloro-triisopropyl-silane (1.9 g, 2.1 mL, 1.2 equiv, 9.6 mmol). The reaction mixture was sealed and the solution was stirred at 25 °C for 18 hours. The resulting solution turned from a transparent yellow solution to an opaque orange solution. To the solution was diluted with CH_2Cl_2 and then SiO_2. The suspension was concentrated *in vacuo* to afford the crude dry-loaded material. The SiO_2 dry-load was subjected to SiO_2 flash chromatography hexanes/diethyl ether (Biotage Sfär DLV 100 g column; 100 mL/min flow-rate; 210/230 nm detector; hexanes/diethyl ether step-gradient; 0% 6CV → 1% 6CV → 5% 6CV → 10% 6CV → 15% 6CV → 20% 6CV → 40% 6CV; POI elutes at 10% diethyl ether). The collected fractions were concentrated *in vacuo* to afford tert-butyl (R)-2-(((triisopropylsilyl)oxy)methyl)-2,5-dihydro-1H-pyrrole-1-carboxylate (2.4 g, 6.7 mmol, 84% yield) as a pale-yellow oil.

^{1}H NMR (800 MHz, Chloroform-*d*; mixture of rotamers) δ 5.90 – 5.73 (m, 2H), 4.56 (brm, 0.4H), 4.46 (brm, 0.6H), 4.22 (brd, *J* = 15.3 Hz, 0.6H), 4.12 (brd, *J* = 15.3 Hz, 0.4H), 4.04 – 3.97 (brm, 1.6H), 3.92 – 3.86 (brm, 0.9H), 3.59 (brt, *J* = 7.2 Hz, 0.5H), 1.49 – 1.44 (brm, 9H), 1.10 – 0.99 (brm, 21H).

^{13}C NMR (201 MHz, Chloroform-*d*; mixture of rotamers) δ 154.2, 154.1, 129.2, 128.9, 125.9, 125.8, 79.5, 79.1, 65.8, 65.5, 65.1, 63.6, 54.2, 53.9, 28.6, 28.5, 18.0, 17.9, 12.0, 11.9.

HRMS (ESI +) calc. mass for $C_{19}H_{37}O_3N^{23}Na^{28}Si$ $[M + Na]^+$ 378.2435; obs. mass for $C_{19}H_{37}O_3N^{23}Na^{28}Si$ $[M + Na]^+$ 378.2434.
$[\alpha]^{20}_D$: +162.6° (c = 1.260 g/100 mL, EtOAc).

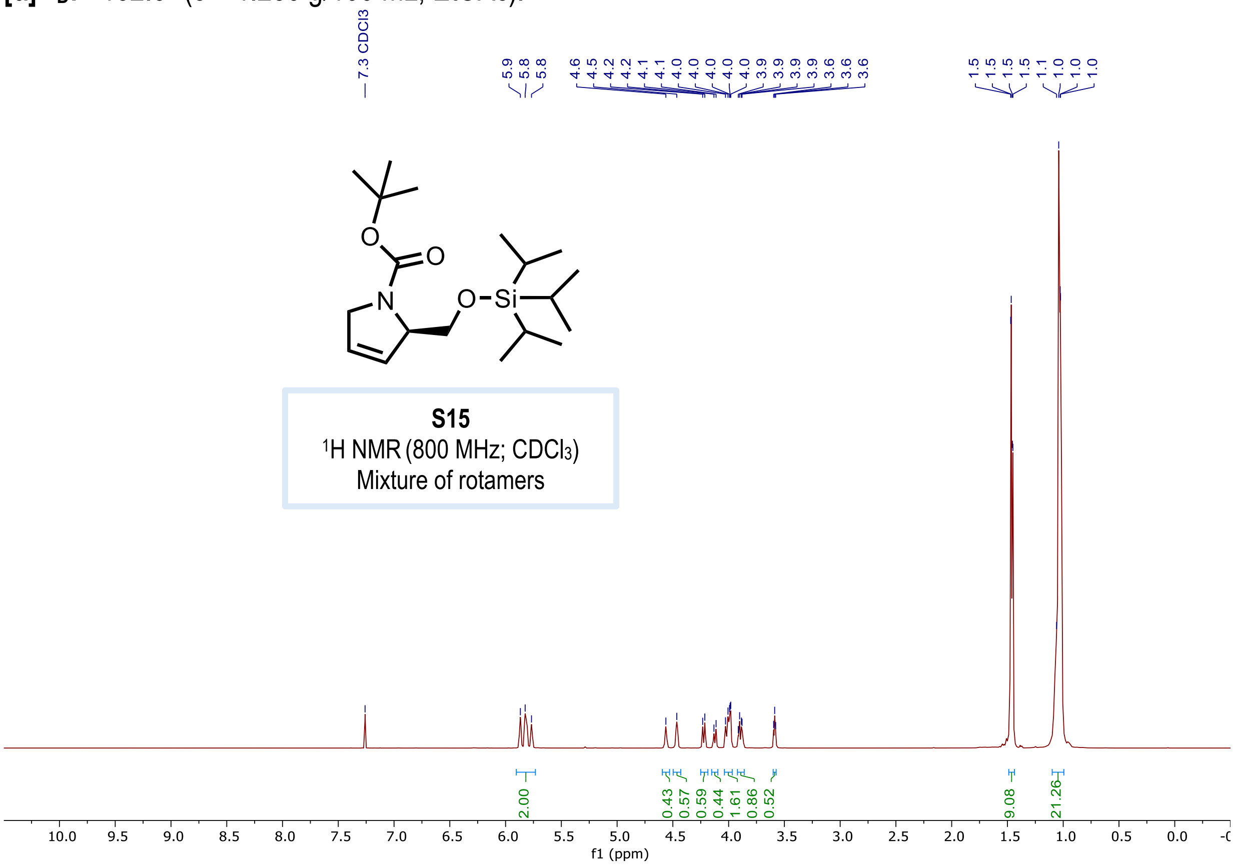

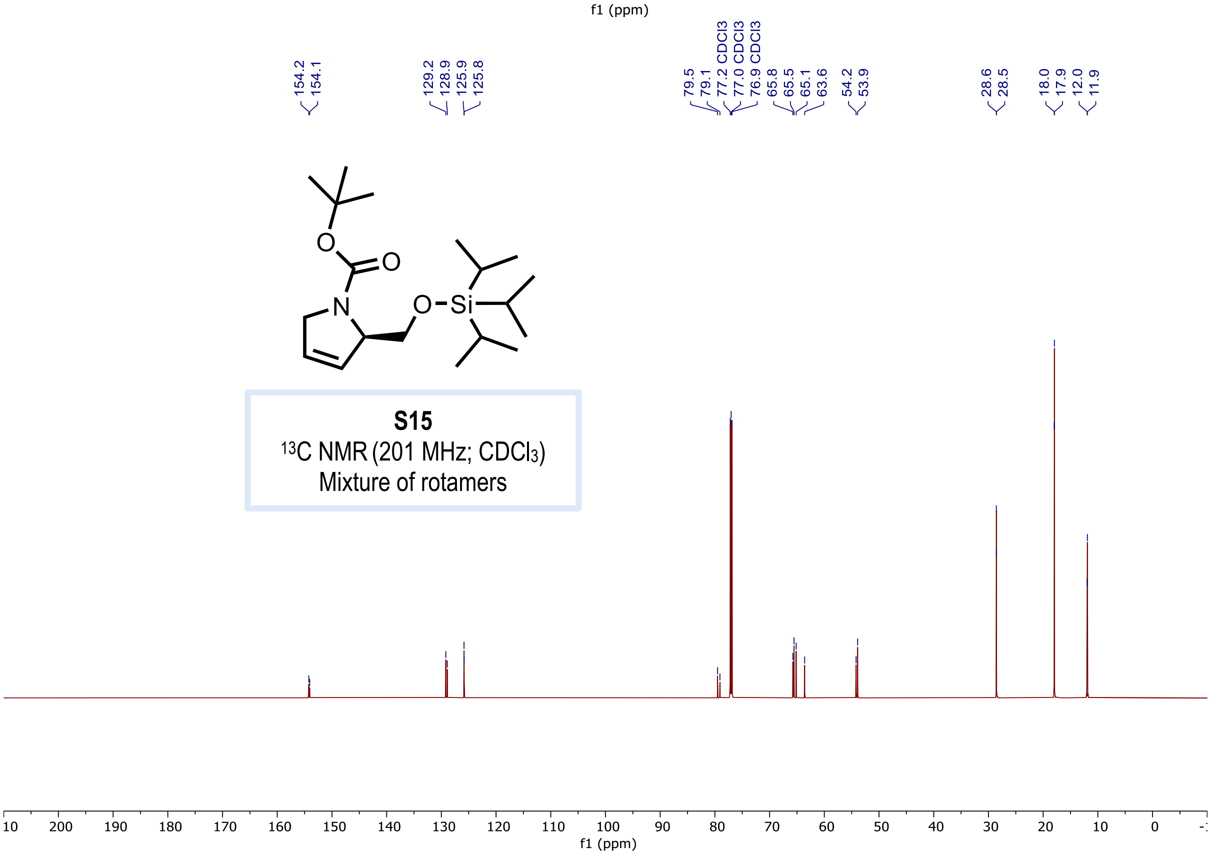

7. Characterization of C–H Functionalization Products

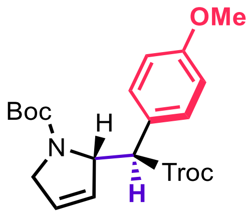

tert-butyl (S)-2-((R)-1-(4-methoxyphenyl)-2-oxo-2-(2,2,2-trichloroethoxy)ethyl)-2,5-dihydro-1H-pyrrole-1-carboxylate (15)

[0.500 mmol Scale]: To a 16 mL vial charged with a stir-bar and 0.5 g of activated 4Å MS was added tert-butyl 2,5-dihydro-1H-pyrrole-1-carboxylate (254 mg, 3 equiv, 1.50 mmol) and was backfilled with nitrogen three times and then capped with a nitrogen balloon. CH_2Cl_2 (500 µL) followed by a stock solution of Rh_2(*S*-PTAD)$_4$ (390 µg, 500 µL CH_2Cl_2, 0.0005 molar, 0.0005 equiv, 0.250 µmol) was added to the vessel. The reaction solution containing the olefin and catalyst was stirred at 25 °C and for at least 10 minutes. After the elapsed time, to the solution was added 2,2,2-trichloroethyl 2-diazo-2-(4-methoxyphenyl)acetate (162 mg, 1.00 mL of CH_2Cl_2, 0.5 molar, 1 equiv, 0.500 mmol) to the reaction vessel dropwise over approximately 30 seconds. After complete addition and an elapsed time of 20 hours, the resulting solution was filtered over a celite plug and the filtrate concentrated *in vacuo* at 50 °C. The crude material was dry-loaded onto SiO_2 and subjected to SiO_2 flash chromatography with pentane/diethyl ether (Biotage Sfär DLV 50 g column as the running-column and a Biotage Sfär DLV 50 g column for the dry-load; 100 mL/min flow-rate; 210/230 nm detector; 0% 6CV → 1% 6CV → 3% 6CV → 5% 6CV → 10% 8CV → 15% 12CV; SM elutes first and then the POI elutes after at 10% - 15% diethyl ether). The collected fractions were concentrated *in vacuo* at 45 °C to afford tert-butyl (S)-2-((R)-1-(4-methoxyphenyl)-2-oxo-2-(2,2,2-trichloroethoxy)ethyl)-2,5-dihydro-1H-pyrrole-1-carboxylate (165 mg, 355 µmol, 71.0% yield, 96% ee, >20:1 d.r.) as a yellow oil.

[100 mmol Scale]: To a flame-dried 1000 mL 3-neck RBF charged with a stir-bar and 100 g activated 4Å MS was added tert-butyl 2,5-dihydro-1H-pyrrole-1-carboxylate (50.766 g, 3 equiv, 300.00 mmol) and 2,2,2-trichloroethyl 2-diazo-2-(4-methoxyphenyl)acetate (32.355 g, 1 equiv, 100.00 mmol) was added to the vessel. The vessel was backfilled with nitrogen three times from the Schlenk-line via a vacuum adapter attached in 1 neck and then topped with two nitrogen balloons in the other 2 necks. To the reaction vessel was added CH_2Cl_2 (200.00 mL).

The red reaction vessel containing trap, diazo, & 4Å MS was stirred at 25 °C and for at least 10 minutes. After the elapsed time, the vacuum adapter attached to the Schlenk-line was opened to the bubbler allowing for the nitrogen balloons to deflate and then to the red reaction solution was added Rh_2(*S*-PTAD)$_4$ (77.967 mg, 200.00 mL of CH_2Cl_2, 0.00025 molar, 0.0005 equiv, 50.000 µmol) to the reaction vessel over approximately 5 minutes (4 x 60 mL syringes). [**Note:** *the reaction does not extrude nitrogen violently compared to the para-Br-phenyl trichloroethyl diazoacetate when used*]. After complete addition, an exotherm of 34.3 °C was recorded as the maximum temperature of the reaction. The bubbling stopped after a couple of minutes. The reaction mixture was allowed to stir overnight.

After an elapsed time of 20 hours, the yellow-orange reaction solution was vacuum filtered over a plug of celite and concentrated *in vacuo over* 55 °C. The material was filtered through a celite plug into a flask for Kugelrohr distillation at 110 °C with 0.6 to 0.7 torr vacuum for at least 1 hour to get rid of the majority of unreacted trap. The resulting pot residue contains predominantly the desired product as a clear orange-yellow oil. The distillate containing the unreacted trap was saved and repurified for future reactions.

The crude material was dry-loaded onto SiO_2 and subjected to SiO_2 flash chromatography with hexanes/ether (350 g Biotage Sfär DLV column as the running-column and 200 g Biotage Sfär DLV column for the dry-load; 350 g KP-Sil setting; 200 mL/min flow rate; 210/230 nm detector; hexanes/ether step-gradient; 0% 10CV → 1% 5CV → 3% 3CV → 5% 3CV → 10% 3CV → 15% 3CV → 30% 3CV → 50%

3CV → 75% 3CV → 100% 10CV; TLC conditions 1:1 hexanes/ether POI Rf is ~0.6 rf and stains with $KMnO_4$ with heat; Trap Rf is ~0.5 and stains with $KMnO_4$ immediately even without heat; POI elutes at around 15% to 50% ether).

The collected fractions were concentrated *in vacuo* at 55 °C to afford tert-butyl (S)-2-((R)-1-(4-methoxyphenyl)-2-oxo-2-(2,2,2-trichloroethoxy)ethyl)-2,5-dihydro-1H-pyrrole-1-carboxylate (40.3 g, 86.7 mmol, 86.7% yield; 96% ee, >20:1 d.r.) as a yellow oil.

Yields of other reaction scales: [15.00 mmol scale] – 6.3 g, 14 mmol, **90 % yield**, 96% ee, >20:1 d.r.; **[40.00 mmol scale]** – 15.5 g, 33.4 mmol, **83.4% yield**, 96% ee, >20:1 d.r.; **[92.7 mmol scale]** – 38.9 g, 83.7 mmol, **90.3% yield**, 96% ee, >20:1 d.r.

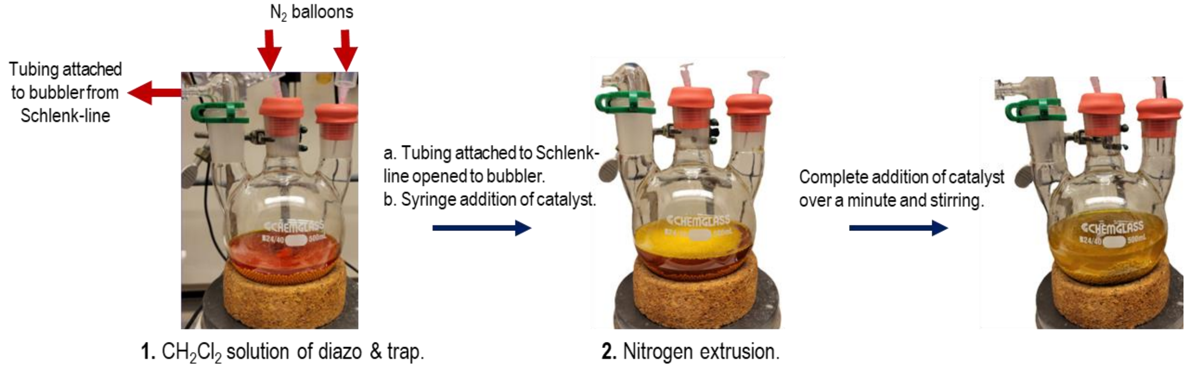

Figure S7. Example of a setup for large scale synthesis of **15**. **Word of caution**: Make sure that bubbler is working properly by opening Schlenk line port to allow the nitrogen balloons to deflate. If the bubbler is not bubbling, make sure to fix before adding catalyst. If the bubbler is working properly, then you can begin to add the catalyst solution.

^{1}H NMR (600 MHz, Chloroform-*d*; as a 0.55:0.45 mixture of rotamers) δ 7.36 (Ar–H d, *J* = 8.3 Hz, 1.1H, major rotamer), 7.31 (Ar–H d, *J* = 8.3 Hz, 0.9H, minor rotamer), 6.89 (Ar–H d, *J* = 8.3 Hz, 0.9H, minor rotamer), 6.86 (Ar–H d, *J* = 8.3 Hz, 1.1H, major rotamer), 5.95 – 5.86 (=C–H brm, 0.45H, minor rotamer), 5.84 – 5.72 (=C–H brm, 1.55H, mixture of rotamers [0.45H minor rotamer + 1.1H major rotamer = 1.55H]), 4.95 – 4.89 (C–$H_{\alpha\text{-}N,C\text{-}2}$ brm, 0.55H, major rotamer), 4.83 (C–$H_{methylene}$ d, *J* = 12.0 Hz, 0.55H, major rotamer), 4.77 (C–$H_{methylene}$ & C–$H_{\alpha\text{-}N,C\text{-}2}$ d & brm, *J* = 12.0 Hz, 0.9H, minor rotamers [0.45H minor rotamer + 0.45H minor rotamer = 0.9H]), 4.64 (C–$H_{methylene}$ d, *J* = 12.0 Hz, 1H, mixture of rotamers [0.45H minor rotamer + 0.55H major rotamer = 1H]), 4.62 (C–$H_{benzylic}$ d, *J* = 4.8 Hz, 0.55H, major rotamer), 4.46 (C–$H_{benzylic}$ d, *J* = 4.8 Hz, 0.45H, minor rotamer), 4.25 (C–$H_{\alpha\text{-}N,C\text{-}5}$ d, *J* = 15.4 Hz, 0.45H, minor rotamer), 4.11 (C–$H_{\alpha\text{-}N,C\text{-}5}$ d, *J* = 15.4 Hz, 0.55H, major rotamer), 3.99 (C–$H_{\alpha\text{-}N,C\text{-}5}$ dd, *J* = 15.4, 6.0 Hz, 0.45H, minor rotamer), 3.92 (C–$H_{\alpha\text{-}N,C\text{-}5}$ dd, *J* = 15.4, 6.0 Hz, 0.55H, major rotamer), 3.80 (–OCH_3 s, 3H, mixture of rotamers), 1.53 (–$C(CH_3)_3$ s, 4.05H, minor rotamer), 1.48 (–$C(CH_3)_3$ s, 4.95H, major rotamer).
^{13}C NMR (151 MHz, $CDCl_3$; as a mixture of rotamers) δ 170.2, 169.8, 159.2, 159.0, 154.4, 153.9, 130.3, 129.9, 127.6, 127.2, 126.9, 126.7, 114.1, 113.8, 95.0, 94.8, 80.3, 79.9, 74.2, 74.2, 74.0, 68.3, 68.2, 55.3, 55.3, 54.3, 54.1, 53.3, 52.1, 28.5.
HRMS (ESI +) calc. mass for $C_{20}H_{25}O_5N^{35}Cl_3$ $[M + H]^+$ 464.0793; obs. mass for $C_{20}H_{25}O_5N^{35}Cl_3$ $[M + H]^+$ 464.0798.
$[\alpha]^{20}_D$: -116.7° (c = 0.270 g/100 mL, EtOAc).
SFC (ChiralCel OJ-3 column; sample dissolved in *n*-Heptane; 1% cosolvent of 1:1 MeOH:IPA with 0.2% formic acid; 2.5 mL min^{-1} flow-rate; 10 min; UV 210 nm): retention times of 1.64 min (major) and 1.93 min (major); 96% ee with Rh_2(*S*-PTAD)$_4$.
Determination of d.r.: *N*-Boc deprotection (1.5 equiv of *p*-Toluenesulfonic acid monohydrate in EtOAc [0.1 M] at 60 °C overnight); observed >20:1 d.r.

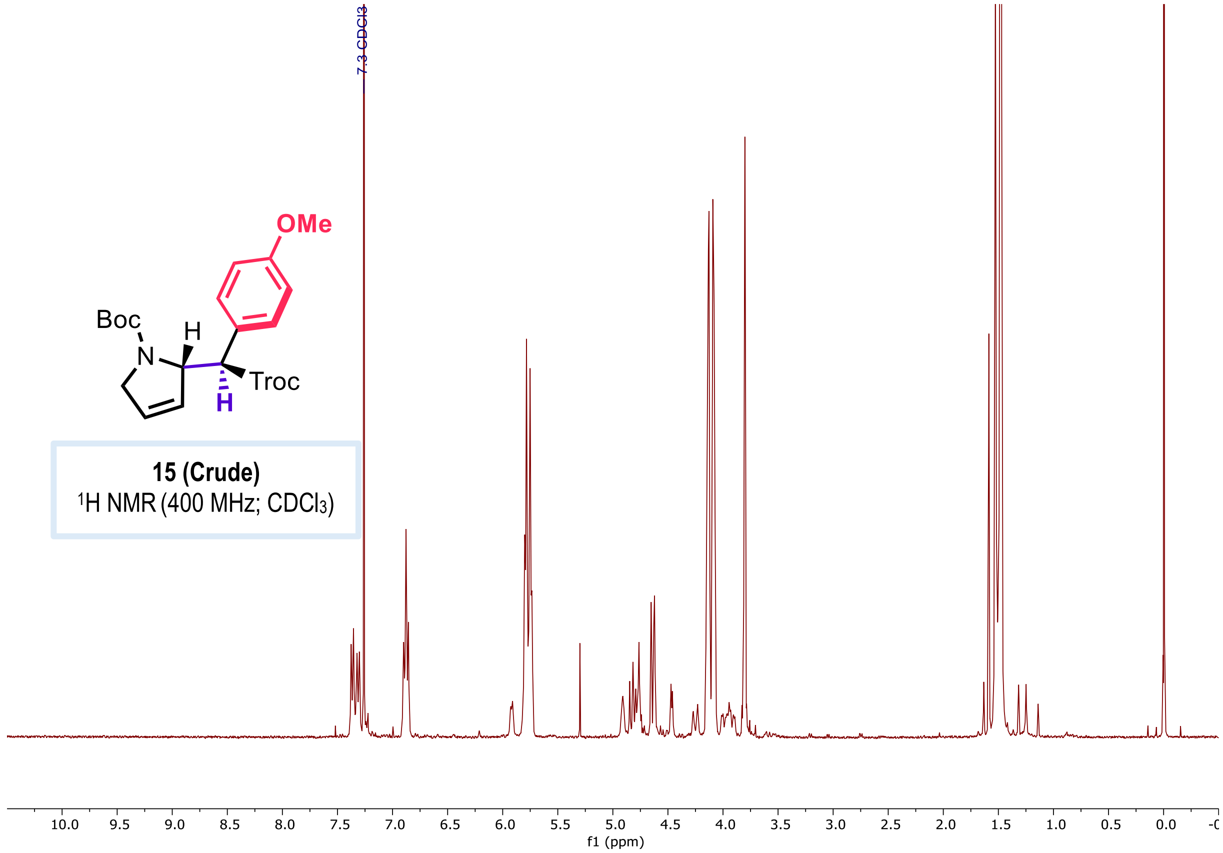
OMe
Boc
N
H
Troc
H
15 (Crude)
1H NMR (400 MHz; CDCl3)
7.3 CDCl3
10.0 9.5 9.0 8.5 8.0 7.5 7.0 6.5 6.0 5.5 5.0 4.5 4.0 3.5 3.0 2.5 2.0 1.5 1.0 0.5 0.0
f1 (ppm)

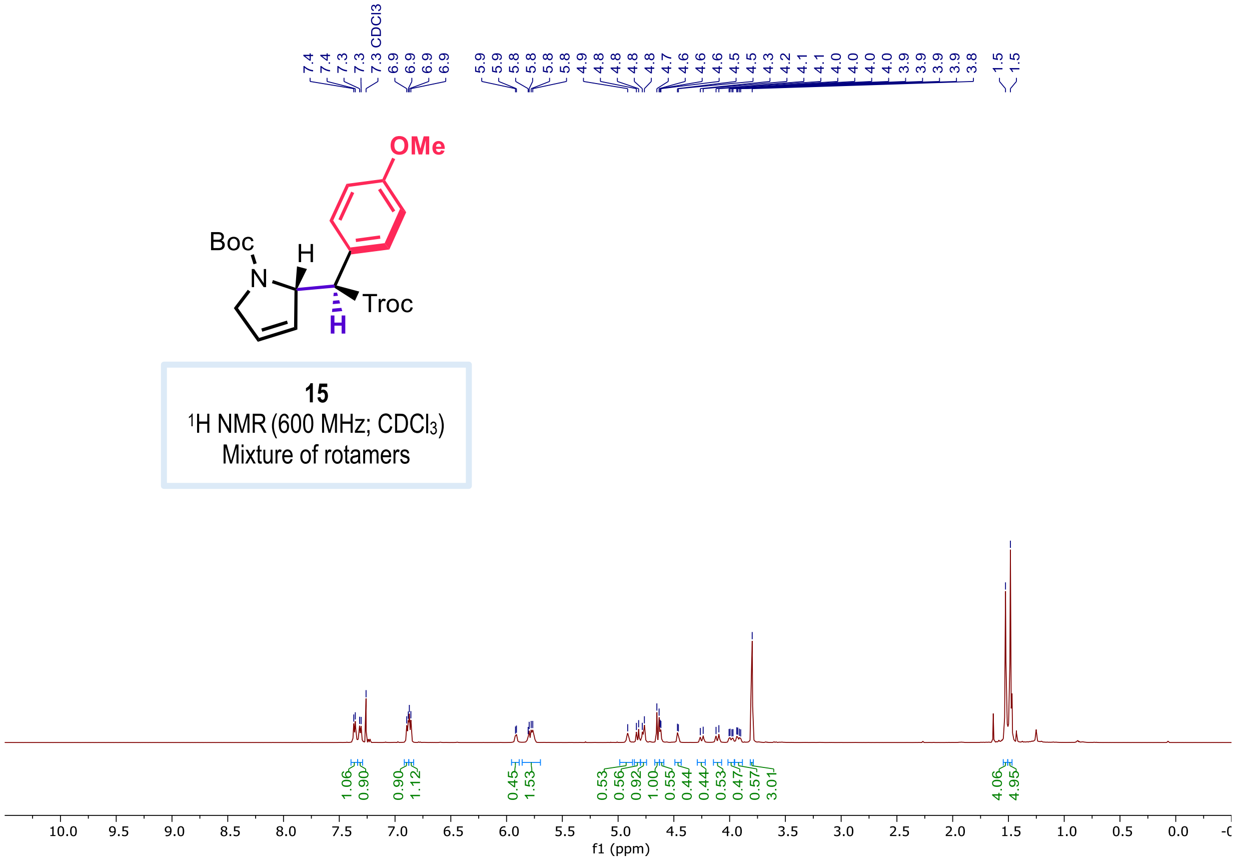
7.4 7.4 7.3 7.3 7.3 CDCl3 6.9 6.9 6.9 6.9
5.9 5.9 5.8 5.8 5.8 5.8 4.9 4.8 4.8 4.8 4.8 4.7 4.6 4.6 4.6 4.5 4.5 4.3 4.2 4.1 4.1 4.0 4.0 4.0 4.0 3.9 3.9 3.9 3.9 3.8 1.5 1.5
OMe
Boc
N
H
Troc
H
15
1H NMR (600 MHz; CDCl3)
Mixture of rotamers
1.06 0.90 0.90 1.12 0.45 1.53 0.53 0.56 0.92 1.00 0.55 0.44 0.44 0.53 0.47 0.57 3.01 4.06 4.95
10.0 9.5 9.0 8.5 8.0 7.5 7.0 6.5 6.0 5.5 5.0 4.5 4.0 3.5 3.0 2.5 2.0 1.5 1.0 0.5 0.0
f1 (ppm)

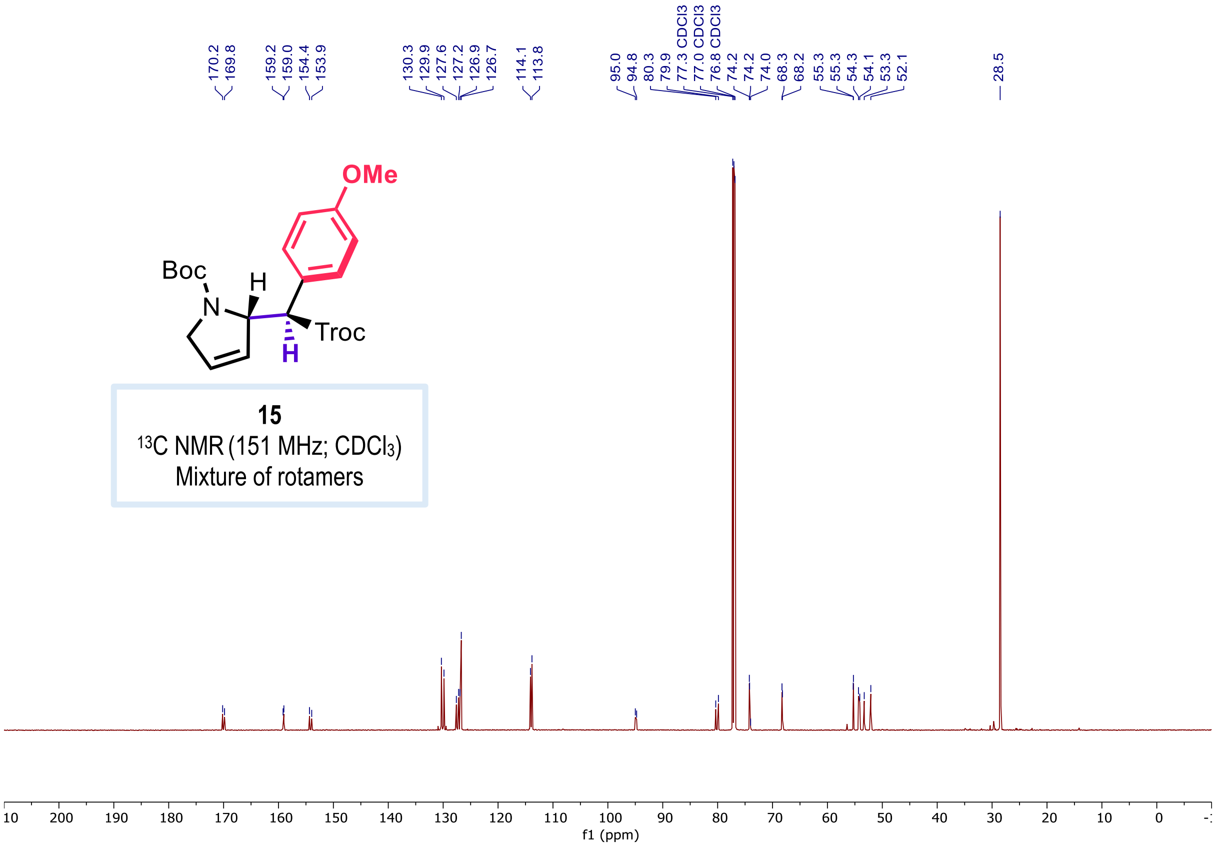
170.2
169.8
159.2
159.0
154.4
153.9
130.3
129.9
127.6
127.2
126.9
126.7
114.1
113.8
95.0
94.8
80.3
79.9
77.3 CDCl3
77.0 CDCl3
76.8 CDCl3
74.2
74.2
74.0
68.3
68.2
55.3
55.3
54.3
54.1
53.3
52.1
28.5
OMe
Boc
H
N
Troc
H
15
13C NMR (151 MHz; CDCl3)
Mixture of rotamers
f1 (ppm)

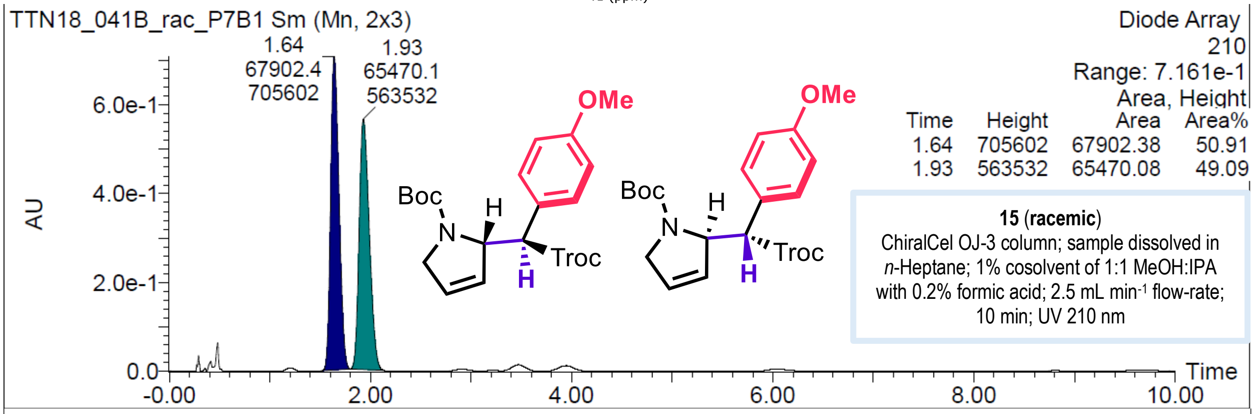
TTN18_041B_rac_P7B1 Sm (Mn, 2x3)
Diode Array
210
Range: 7.161e-1
Area, Height
1.64
67902.4
705602
1.93
65470.1
563532
AU
Time
Time	Height	Area	Area%
1.64	705602	67902.38	50.91
1.93	563532	65470.08	49.09
15 (racemic)
ChiralCel OJ-3 column; sample dissolved in n-Heptane; 1% cosolvent of 1:1 MeOH:IPA with 0.2% formic acid; 2.5 mL min-1 flow-rate; 10 min; UV 210 nm
OMe
Boc
H
N
Troc
H

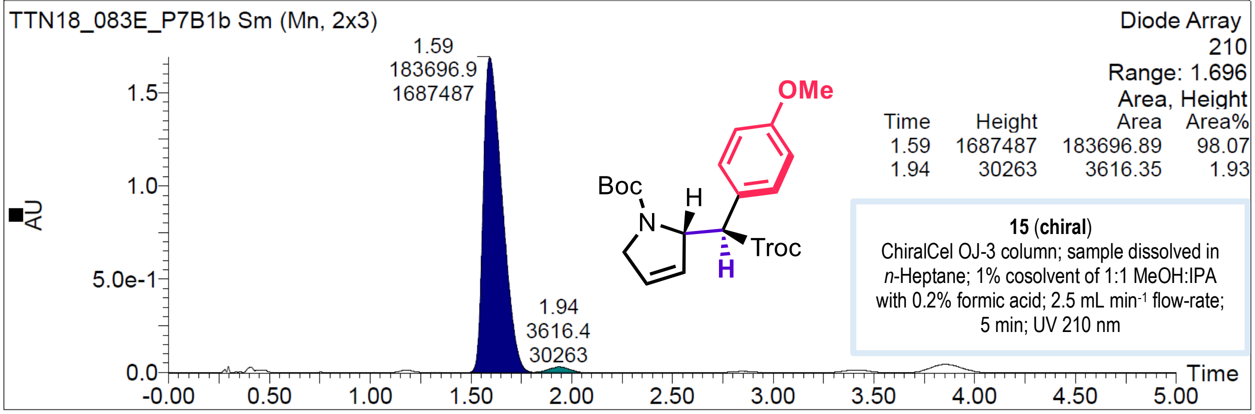
TTN18_083E_P7B1b Sm (Mn, 2x3)
Diode Array
210
Range: 1.696
Area, Height
1.59
183696.9
1687487
1.94
3616.4
30263
AU
Time
Time	Height	Area	Area%
1.59	1687487	183696.89	98.07
1.94	30263	3616.35	1.93
15 (chiral)
ChiralCel OJ-3 column; sample dissolved in n-Heptane; 1% cosolvent of 1:1 MeOH:IPA with 0.2% formic acid; 2.5 mL min-1 flow-rate; 5 min; UV 210 nm
OMe
Boc
H
N
Troc
H

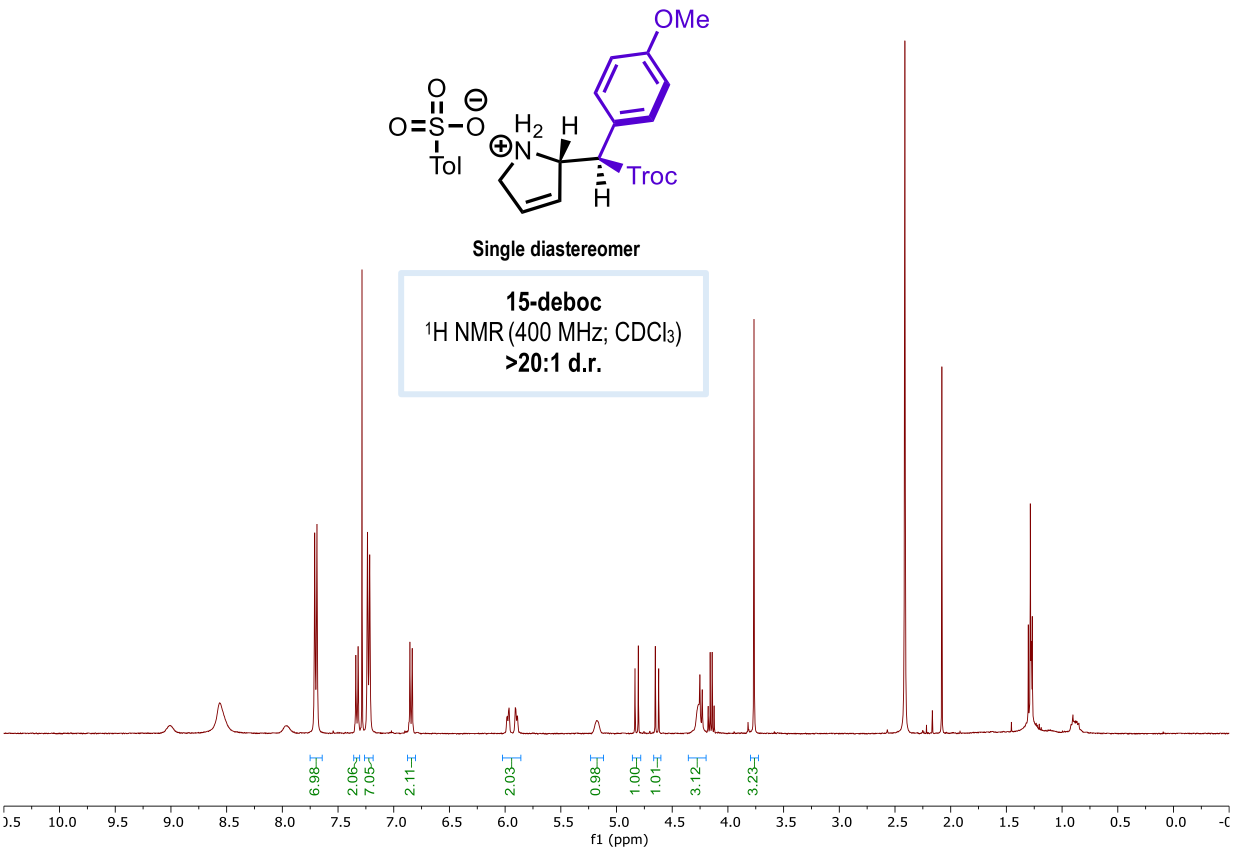

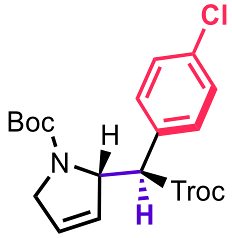

tert-butyl (S)-2-((R)-1-(4-chlorophenyl)-2-oxo-2-(2,2,2-trichloroethoxy)ethyl)-2,5-dihydro-1H-pyrrole-1-carboxylate (16)

To an 8 mL vial charged with a stir-bar and 0.5 g of activated 4Å MS was added tert-butyl 2,5-dihydro-1H-pyrrole-1-carboxylate (254 mg, 3 equiv, 1.50 mmol) and 2,2,2-trichloroethyl 2-(4-chlorophenyl)-2-diazoacetate (164 mg, 1 equiv, 0.500 mmol). The reaction vial was sealed, backfilled with nitrogen three times, and the vial topped with a nitrogen balloon. To the vial containing the diazo and olefin was added CH_2Cl_2 (1.5 mL). The solution was stirred at room temperature for at least 10 minutes. A stock solution of Rh_2(*S*-PTAD)$_4$ (390 µg, 500 µL CH_2Cl_2, 0.0005 molar, 0.0005 equiv, 0.250 µmol) was added into the reaction solution containing diazo and olefin drop-wise over 15 seconds. The reaction mixture was then allowed to stir for 20 hours at room temperature. After the elapsed time, the reaction mixture was filtered through a celite plug and the filtrate concentrated *in vacuo* at 45 °C. The crude was dry-loaded onto SiO_2 and subjected to SiO_2 flash chromatography pentane/diethyl ether (Biotage Sfär DLV 50 g column as the running-column and Biotage Sfär DLV 50 g column for the dry-load; 100 mL/min flow-rate; 210/230 nm detector; 0% 6CV → 1% 6CV → 3% 6CV → 5% 6CV → 7% 6CV → 10% 6CV → 13% 6CV → 16% 12CV; POI elutes at 13% diethyl ether). The collected fractions were concentrated *in vacuo* at 45 °C to afford tert-butyl (S)-2-((R)-1-(4-chlorophenyl)-2-oxo-2-(2,2,2-trichloroethoxy)ethyl)-2,5-dihydro-1H-pyrrole-1-carboxylate (163 mg, 347 µmol, 69.5% yield, 92% ee, >20:1 d.r.) as a clear pale-yellow oil.

^{1}H NMR (400 MHz, Chloroform-*d*; as a 0.6:0.4 mixture of rotamers) δ 7.42 – 7.28 (Ar–H m, 4H, mixture of rotamers), 6.00 – 5.89 (=C–H brm, 0.4H, minor rotamer), 5.85 – 5.68 (=C–H brm, 1.6H, mixture of rotamers [0.4H minor rotamer + 1.2H major rotamer = 1.6H]), 5.01 – 4.91 (C–$H_{\alpha\text{-}N,C\text{-}2}$ brm, 0.6H, major rotamer), 4.79 (C–$H_{methylene}$ & C–$H_{\alpha\text{-}N,C\text{-}2}$ d & brm, *J* = 11.8 Hz, 1.4H, mixture of rotamers [0.4H minor rotamer + 0.4H minor rotamer + 0.6H major rotamer = 1.4H]), 4.69 (C–$H_{methylene}$ d, *J* = 11.8 Hz, 1H, mixture of rotamers [0.4H minor rotamer + 0.6H major rotamer = 1H]), 4.58 (C–$H_{benzylic}$ d, *J* = 4.7 Hz, 0.6H, major rotamer), 4.44 (C–$H_{benzylic}$ d, *J* = 4.7 Hz, 0.4H, minor rotamer), 4.26 (C–$H_{\alpha\text{-}N,C\text{-}5}$ d, *J* = 15.7 Hz, 0.4H, minor rotamer), 4.11 (C–$H_{\alpha\text{-}N,C\text{-}5}$ d, *J* = 15.7 Hz, 0.6H, major rotamer), 3.97 (C–$H_{\alpha\text{-}N,C\text{-}5}$ dd, *J* = 15.7, 5.3 Hz, 0.4H, minor rotamer), 3.88 (C–$H_{\alpha\text{-}N,C\text{-}5}$ dd, *J* = 15.7, 5.3 Hz, 0.6H, major rotamer), 1.51 (–$C(CH_3)_3$ s, 3.6H, minor rotamer), 1.48 (–$C(CH_3)_3$ s, 5.4H, major rotamer).
^{13}C NMR (151 MHz, Chloroform-*d*; as a mixture of rotamers) δ 169.7, 169.3, 154.4, 153.9, 133.8, 133.6, 133.1, 130.7, 130.1, 128.9, 128.6, 128.1, 127.7, 126.6, 126.3, 94.8, 94.6, 80.6, 80.1, 74.4, 74.3, 68.0, 67.9, 54.3, 54.1, 53.7, 52.5, 28.5.
HRMS (ESI +) calc. mass for $C_{19}H_{22}O_4N^{35}Cl_4$ [M + H]$^+$ 468.0298; obs. mass for $C_{19}H_{22}O_4N^{35}Cl_4$ [M + H]$^+$ 468.0301.
[α]$^{20}_{D}$: -23.1° (c = 0.475 g/100 mL, EtOAc).
SFC (Regis (S,S) Whelk-O 1 Kromasil column; sample dissolved in *n*-Heptane; 1% cosolvent of 1:1 MeOH:IPA with 0.2% formic acid; 2.5 mL min^{-1} flow-rate; 10 min; UV 230 nm): retention times of 5.74 min (major) and 6.28 (minor); 92% ee with Rh_2(*S*-PTAD)$_4$.
Determination of d.r.: *N*-Boc deprotection (1.5 equiv of *p*-Toluenesulfonic acid monohydrate in EtOAc [0.1 M] at 60 °C, 4 hours); observed >20:1 d.r.

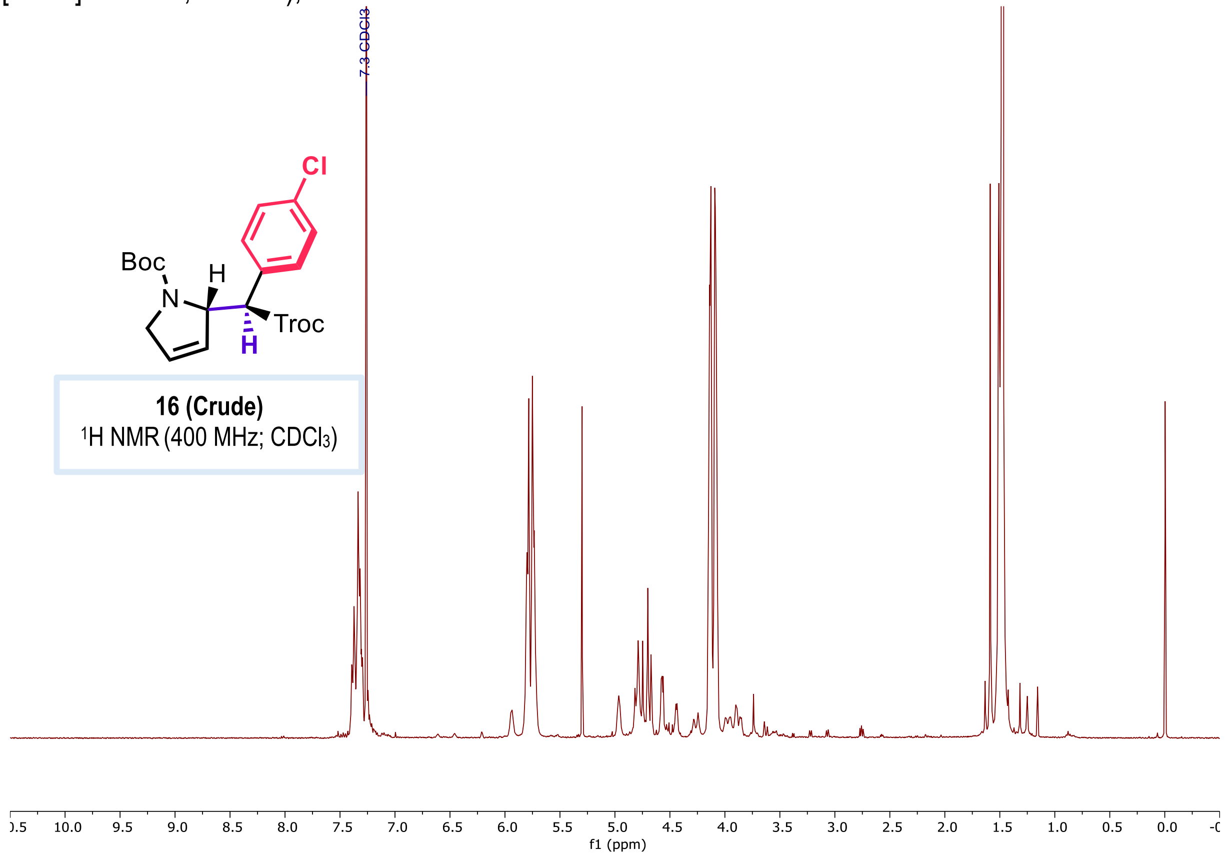

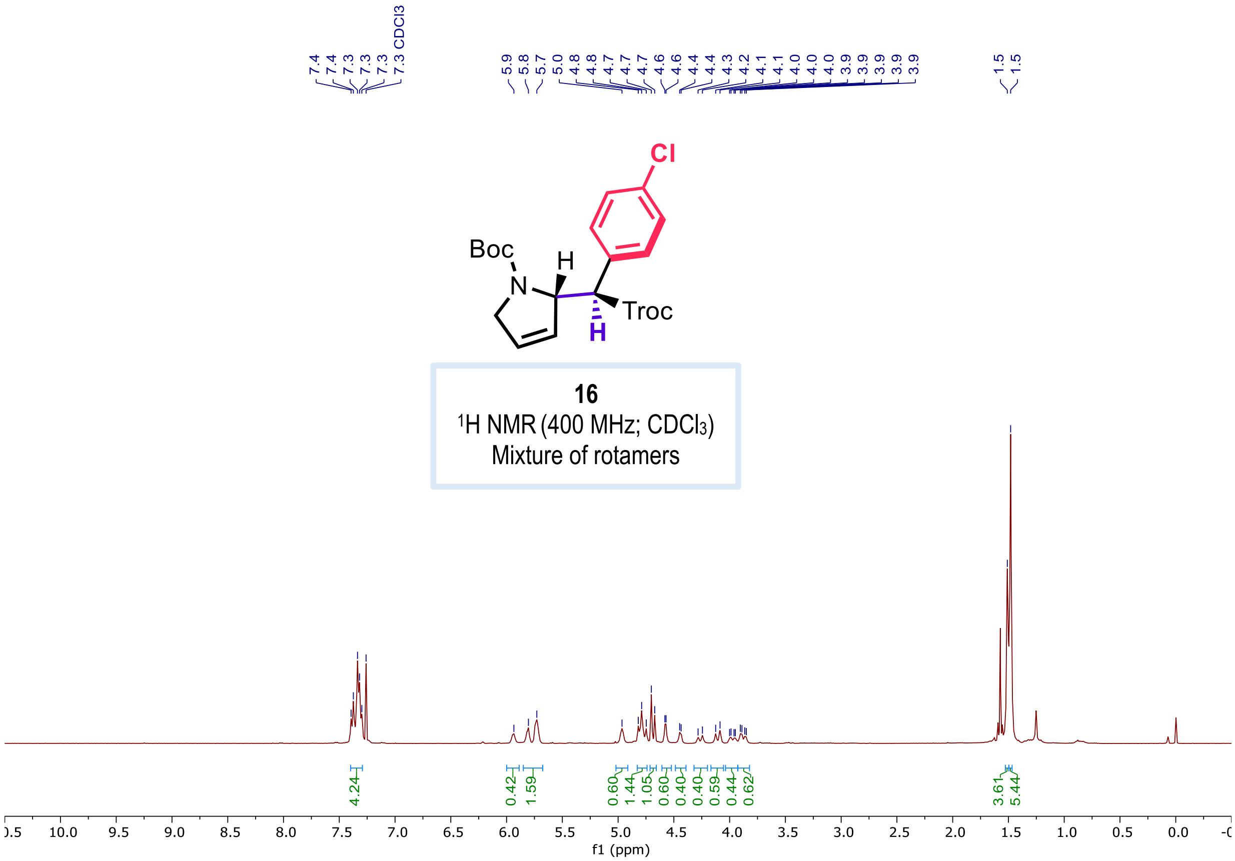
7.4
7.4
7.3
7.3
7.3
7.3 CDCl3
5.9
5.8
5.7
5.0
4.8
4.8
4.7
4.7
4.7
4.6
4.6
4.4
4.4
4.3
4.2
4.1
4.1
4.0
4.0
3.9
3.9
3.9
3.9
3.9
1.5
1.5
Cl
Boc
N
H
Troc
H
16
1H NMR (400 MHz; CDCl3)
Mixture of rotamers
4.24
0.42
1.59
0.60
1.44
1.05
0.60
0.40
0.40
0.59
0.44
0.62
3.61
5.44
f1 (ppm)

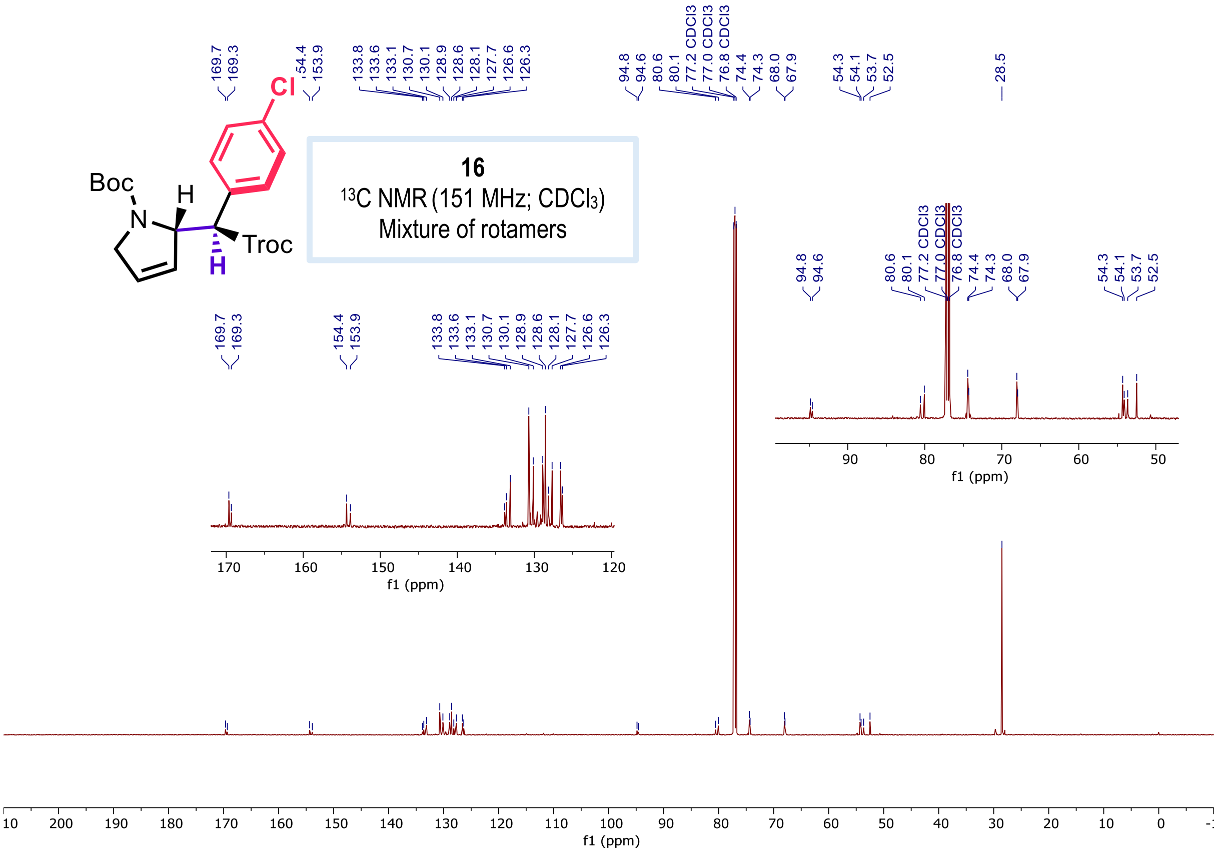
169.7
169.3
154.4
153.9
133.8
133.6
133.1
130.7
130.1
128.9
128.6
128.1
127.7
126.6
126.3
94.8
94.6
80.6
80.1
77.2 CDCl3
77.0 CDCl3
76.8 CDCl3
74.4
74.3
68.0
67.9
54.3
54.1
53.7
52.5
28.5
Cl
Boc
N
H
Troc
H
16
13C NMR (151 MHz; CDCl3)
Mixture of rotamers
f1 (ppm)

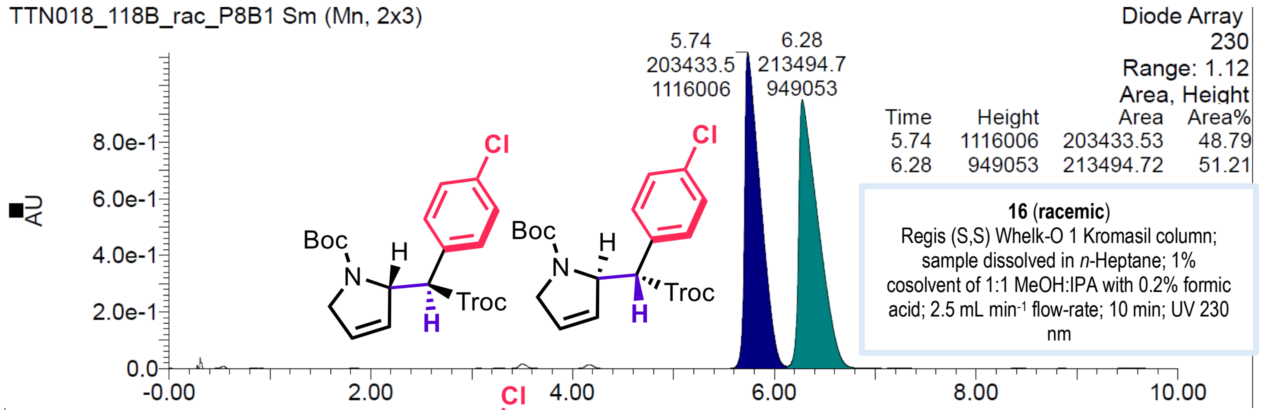
TTN018_118B_rac_P8B1 Sm (Mn, 2x3)
Diode Array
230
Range: 1.12
Area, Height
5.74
203433.5
1116006
6.28
213494.7
949053
Time Height Area Area%
5.74 1116006 203433.53 48.79
6.28 949053 213494.72 51.21
16 (racemic)
Regis (S,S) Whelk-O 1 Kromasil column; sample dissolved in n-Heptane; 1% cosolvent of 1:1 MeOH:IPA with 0.2% formic acid; 2.5 mL min-1 flow-rate; 10 min; UV 230 nm
Boc
N
H
Troc
Cl
AU
8.0e-1
6.0e-1
4.0e-1
2.0e-1
0.0
-0.00
2.00
4.00
6.00
8.00
10.00

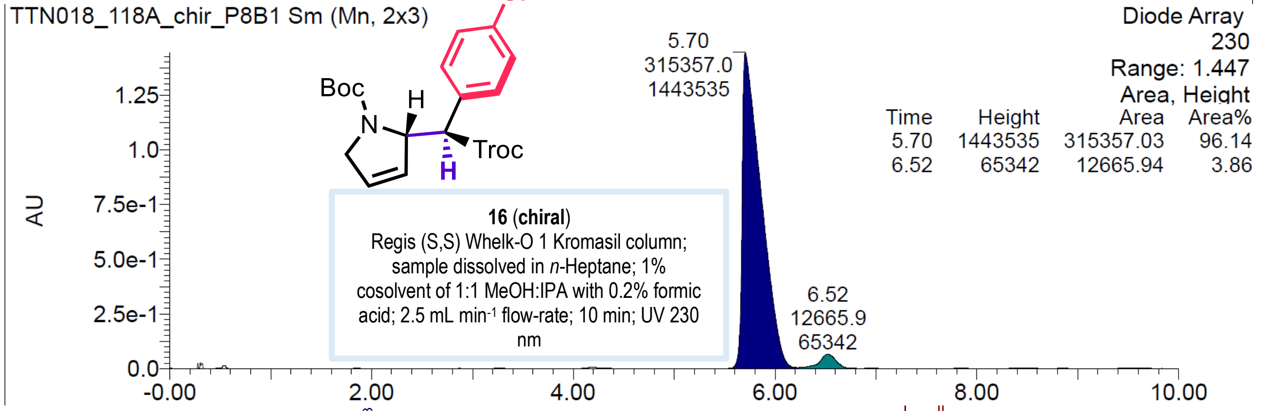
TTN018_118A_chir_P8B1 Sm (Mn, 2x3)
Diode Array
230
Range: 1.447
Area, Height
5.70
315357.0
1443535
6.52
12665.9
65342
Time Height Area Area%
5.70 1443535 315357.03 96.14
6.52 65342 12665.94 3.86
16 (chiral)
Regis (S,S) Whelk-O 1 Kromasil column; sample dissolved in n-Heptane; 1% cosolvent of 1:1 MeOH:IPA with 0.2% formic acid; 2.5 mL min-1 flow-rate; 10 min; UV 230 nm
Boc
N
H
Troc
Cl
AU
1.25
1.0
7.5e-1
5.0e-1
2.5e-1
0.0
-0.00
2.00
4.00
6.00
8.00
10.00

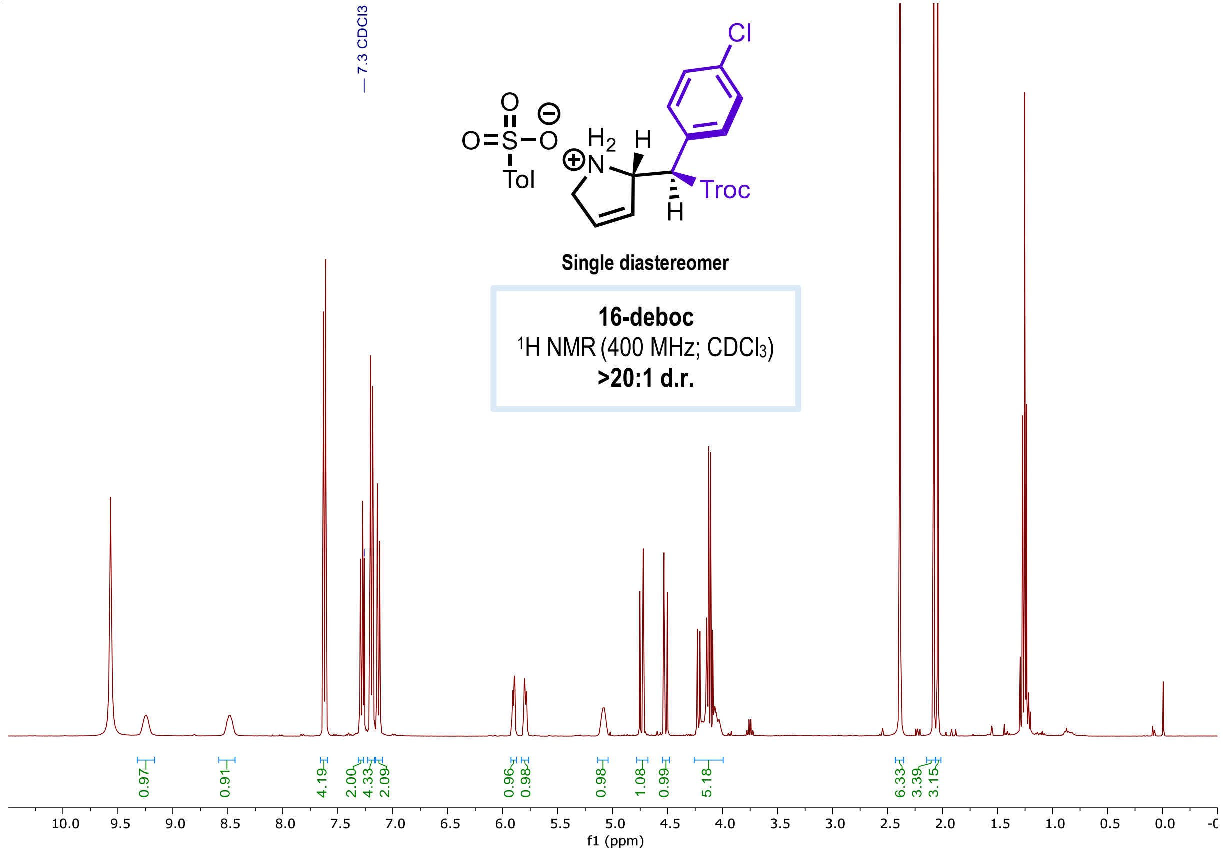
7.3 CDCl3
Cl
O
O=S-O
Tol
H2
N
H
Troc
H
Single diastereomer
16-deboc
1H NMR (400 MHz; CDCl3)
>20:1 d.r.
0.97
0.91
4.19
2.00
4.33
2.09
0.96
0.98
0.98
1.08
0.99
5.18
6.33
3.39
3.15
10.0
9.5
9.0
8.5
8.0
7.5
7.0
6.5
6.0
5.5
5.0
4.5
4.0
3.5
3.0
2.5
2.0
1.5
1.0
0.5
0.0
f1 (ppm)

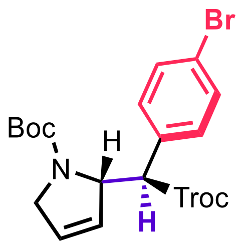

tert-butyl (S)-2-((R)-1-(4-bromophenyl)-2-oxo-2-(2,2,2-trichloroethoxy)ethyl)-2,5-dihydro-1H-pyrrole-1-carboxylate (17)

To a 500 mL RBF charged with a stir-bar and 30 g of activated 4Å MS was added tert-butyl 2,5-dihydro-1H-pyrrole-1-carboxylate (15.23 g, 3 equiv, 90.00 mmol) and 2,2,2-trichloroethyl 2-(4-bromophenyl)-2-diazoacetate (11.17 g, 1 equiv, 30.00 mmol) and was backfilled with nitrogen three times and then sealed with a septum and an argon balloon. CH_2Cl_2 (90.09 mL) was added to the vessel. The reaction vessel containing diazo and olefin was stirred at 25 °C for at least 10 minutes. After the elapsed time, to the solution was added a stock solution of Rh_2(*S*-PTAD)$_4$ (23.39 mg, 30.00 mL CH_2Cl_2, 0.0005 molar, 0.0005 equiv, 15.00 µmol) to the reaction vessel slowly dropwise over approximately 30 seconds. After complete addition, the resulting solution was reacted for 20 h and then filtered over a celite plug and concentrated *in vacuo* at 50 °C. The crude material was dry-loaded onto SiO_2 and subjected to SiO_2 flash chromatography pentane/diethyl ether (Biotage Sfär DLV 100 g column as the running-column and Biotage Sfär DLV 100 g column for the dry-load; 150 mL/min flow-rate; 210/230 nm detector; 0% 6CV → 1% 6CV → 3% 6CV → 5% 6CV → 7% 6CV → 10% 6CV → 12% 6CV → 14% 6CV → 16% 6CV →18% 6CV → 20% 6CV; SM and POI coelutes after at 12% - 16% ether). The collected fractions were concentrated *in vacuo* at 45 °C to afford the mixture of product and trap as a pale-yellow oil. The mixture was filtered through a plug of celite into a RBF for Kugelrohr distillation, concentrated *in vacuo* at 45 °C and then subjected to Kugelrohr distillation with a 110 °C air bath under vacuum (7 x 10^{-1} torr) for at least 2 hours. The pot residue was collected as tert-butyl (S)-2-((R)-1-(4-bromophenyl)-2-oxo-2-(2,2,2-trichloroethoxy)ethyl)-2,5-dihydro-1H-pyrrole-1-carboxylate (11.3 g, 22.0 mmol, 73.3% yield, 94% ee, >20:1 d.r.) as a viscous purple viscous gel after leaving it under vacuum for 2 days.

Yields of other reaction scales: [0.500 mmol scale] – 0.18 g, 0.36 mmol, **72% yield**, 94% ee, >20:1 d.r..

^{1}H NMR (400 MHz, Chloroform-*d*, as a 0.6:0.4 mixture of rotamers) δ 7.57 – 7.41 (Ar-H m, 2.2H, mixture of rotamers), 7.37 – 7.26 (Ar-H m, 1.8H, mixture of rotamers), 5.99 – 5.88 (=C–H brm, 0.4H, minor rotamer), 5.87 – 5.67 (=C–H brm, 1.6H, mixture of rotamers [0.4H minor rotamer + 1.2H major rotamer = 1.6H]), 5.01 – 4.90 (C–$H_{\alpha\text{-}N,C\text{-}2}$ brm, 0.6H, major rotamer), 4.78 (C–$H_{methylene}$ & C–$H_{\alpha\text{-}N,C\text{-}2}$ d & brm, *J* = 11.9 Hz, 1.4H, mixture of rotamers [0.4H minor rotamer + 0.4H minor rotamer + 0.6H major rotamer = 1.4H]), 4.69 (C–$H_{methylene}$ d, *J* = 11.9 Hz, 1H, mixture of rotamers [0.4H minor rotamer + 0.6H major rotamer = 1H]), 4.56 (C–$H_{benzylic}$ d, *J* = 4.3 Hz, 0.6H, major rotamer), 4.42 (C–$H_{benzylic}$ d, *J* = 4.3 Hz, 0.4H, minor rotamer), 4.26 (C–$H_{\alpha\text{-}N,C\text{-}5}$ d, *J* = 15.7 Hz, 0.4H, minor rotamer), 4.10 (C–$H_{\alpha\text{-}N,C\text{-}5}$ d, *J* = 15.7 Hz, 0.6H, major rotamer), 3.97 (C–$H_{\alpha\text{-}N,C\text{-}5}$ dd, *J* = 15.7, 5.6 Hz, 0.4H, minor rotamer), 3.88 (C–$H_{\alpha\text{-}N,C\text{-}5}$ dd, *J* = 15.7, 5.6 Hz, 0.6H, major rotamer), 1.51 (–C(CH_3)$_3$ s, 3.6H, minor rotamer), 1.48 (–C(CH_3)$_3$ s, 5.4H, major rotamer).

^{13}C NMR (151 MHz, Chloroform-*d*, as a mixture of rotamers) δ 169.6, 169.2, 154.3, 153.8, 133.7, 133.6, 131.8, 131.5, 131.1, 130.5, 128.2, 127.7, 126.6, 126.3, 121.9, 121.8, 94.8, 94.6, 80.6, 80.0, 74.4, 74.3, 68.0, 67.9, 54.3, 54.1, 53.7, 53.4, 52.5, 28.5, 28.4.

HRMS (ESI +) calc. mass for $C_{19}H_{22}O_4N^{79}Br^{35}Cl_3$ [M + H]$^+$ 511.9792; obs. mass for $C_{19}H_{22}O_4N^{79}Br^{35}Cl_3$ [M + H]$^+$ 511.9798.

[α]$^{20}_D$: -85.1° (c = 1.050 g/100 mL, EtOAc).

SFC (Regis (S,S) Whelk-O 1 Kromasil column; sample dissolved in *n*-Heptane; 1% cosolvent of 1:1 MeOH:IPA with 0.2% formic acid; 2.5 mL min^{-1} flow-rate; 10 min; UV 210 nm): retention times of 7.24 min (major) and 7.98 (minor); 94% ee with Rh_2(*S*-PTAD)$_4$.
Determination of d.r.: *N*-Boc deprotection (1.1 equiv of *p*-Toluenesulfonic acid monohydrate in EtOAc [0.1 M] at 60 °C overnight); observed >20:1 d.r.

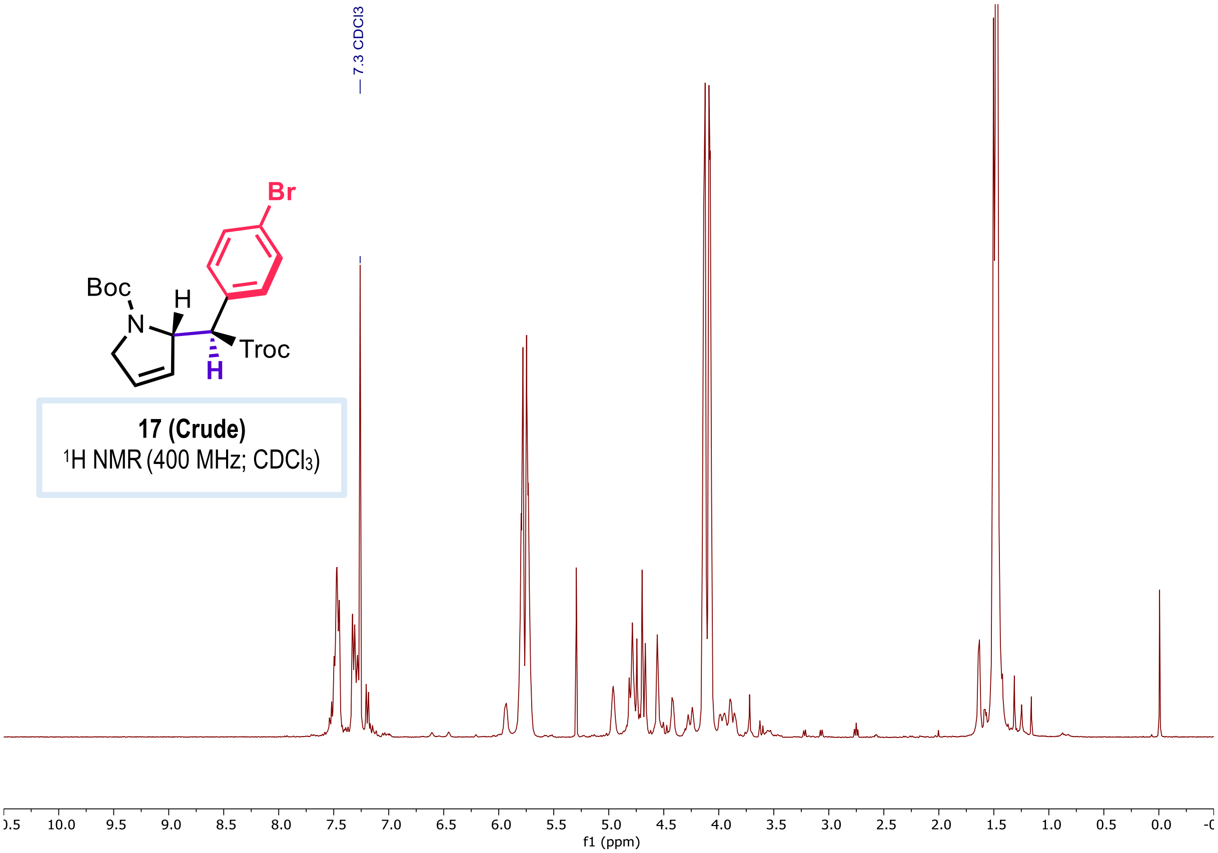

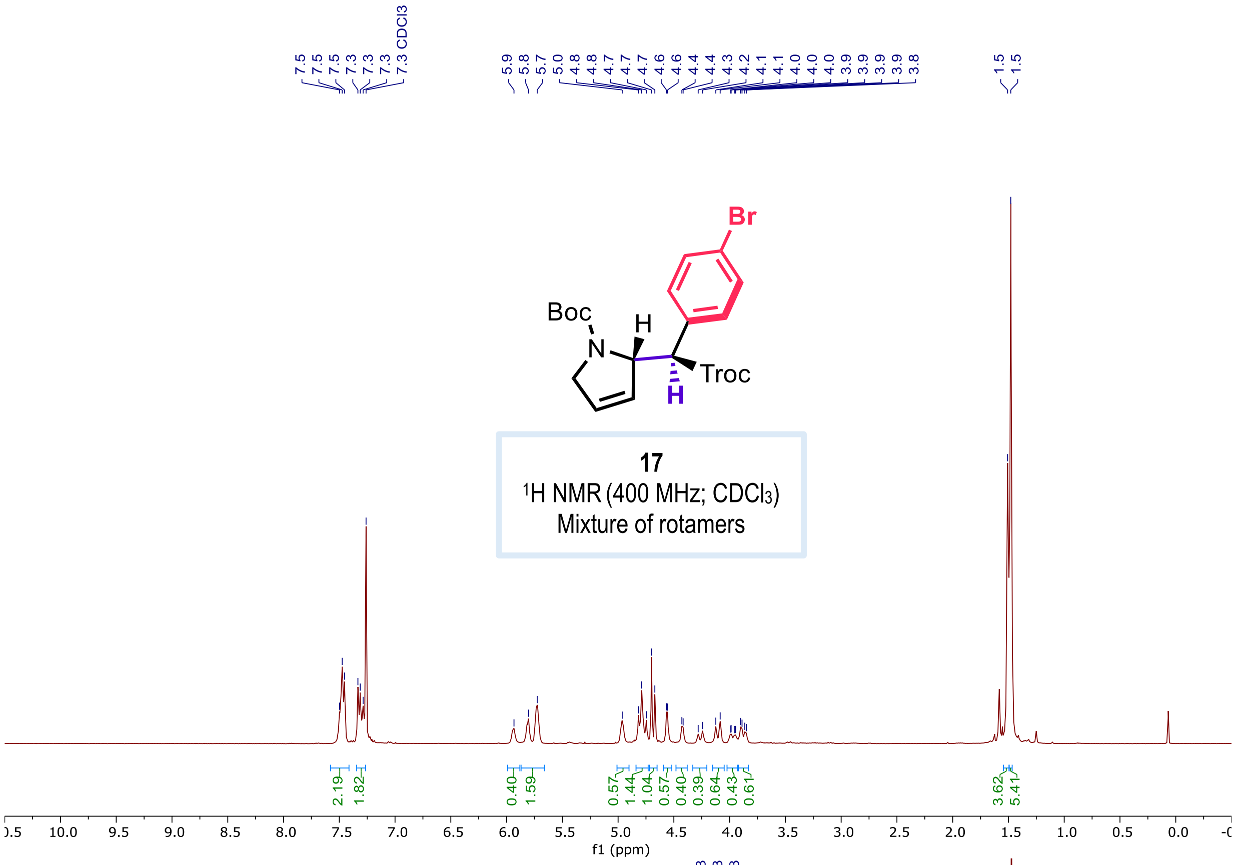
7.5
7.5
7.5
7.3
7.3
7.3
7.3 CDCl3
5.9
5.8
5.7
5.0
4.8
4.8
4.7
4.7
4.7
4.6
4.6
4.4
4.4
4.3
4.2
4.1
4.1
4.0
4.0
3.9
3.9
3.9
3.9
3.8
1.5
1.5
Br
Boc
N
H
Troc
H
17
1H NMR (400 MHz; CDCl3)
Mixture of rotamers
2.19
1.82
0.40
1.59
0.57
1.44
1.04
0.57
0.40
0.39
0.64
0.43
0.61
3.62
5.41
f1 (ppm)

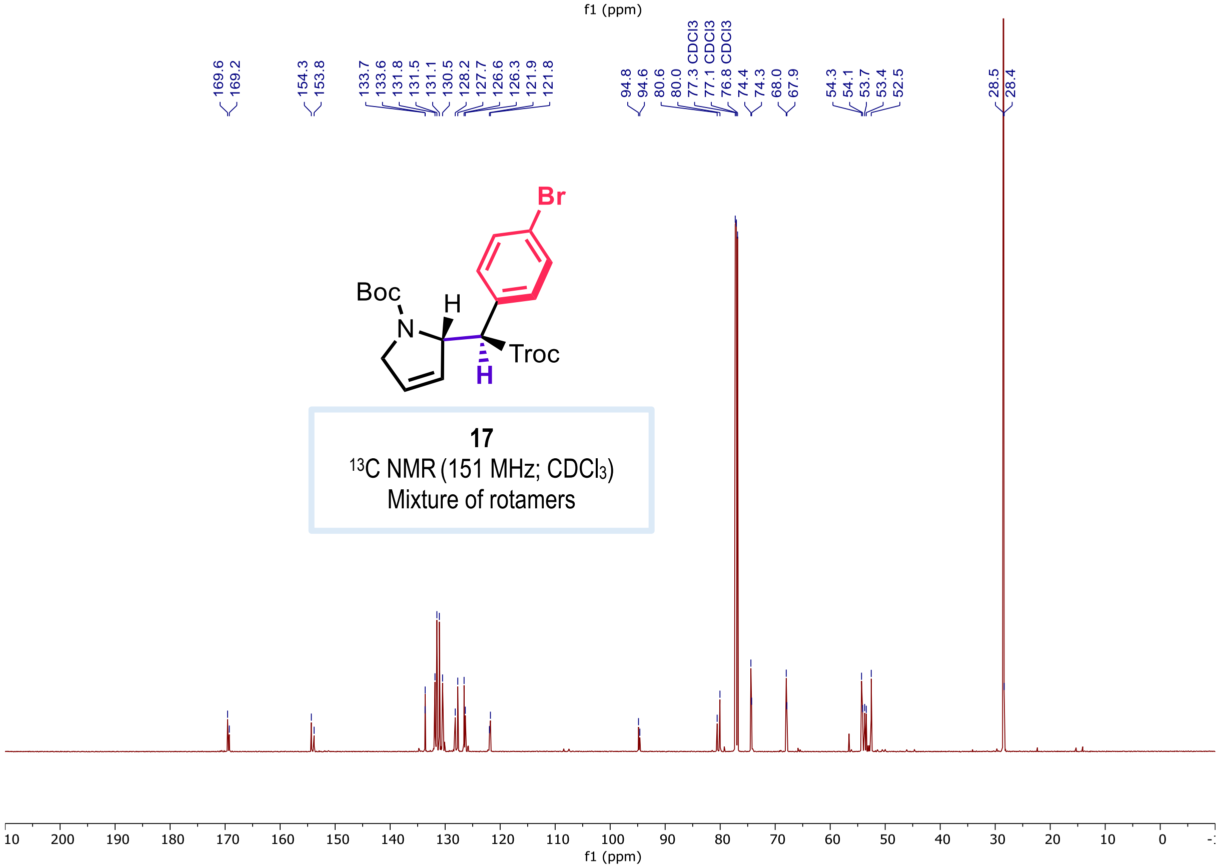
169.6
169.2
154.3
153.8
133.7
133.6
131.8
131.5
131.1
130.5
128.2
127.7
126.6
126.3
121.9
121.8
94.8
94.6
80.6
80.0
77.3 CDCl3
77.1 CDCl3
76.8 CDCl3
74.4
74.3
68.0
67.9
54.3
54.1
53.7
53.4
52.5
28.5
28.4
Br
Boc
N
H
Troc
H
17
13C NMR (151 MHz; CDCl3)
Mixture of rotamers
f1 (ppm)

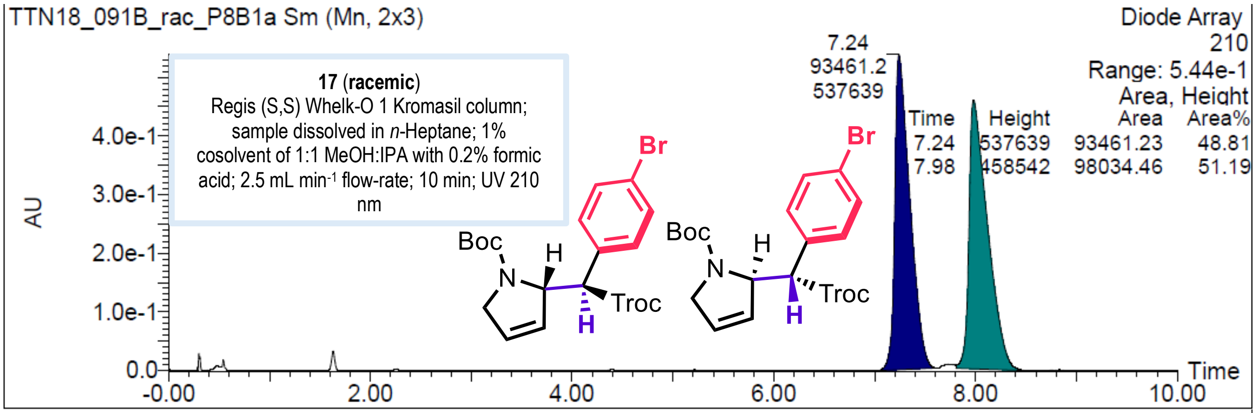
TTN18_091B_rac_P8B1a Sm (Mn, 2x3)
Diode Array
210
Range: 5.44e-1
Area, Height
17 (**racemic**)
Regis (S,S) Whelk-O 1 Kromasil column; sample dissolved in *n*-Heptane; 1% cosolvent of 1:1 MeOH:IPA with 0.2% formic acid; 2.5 mL min-1 flow-rate; 10 min; UV 210 nm
7.24
93461.2
537639
Time Height Area Area%
7.24 537639 93461.23 48.81
7.98 458542 98034.46 51.19
Boc
N
H
H
Troc
Br
AU
Time

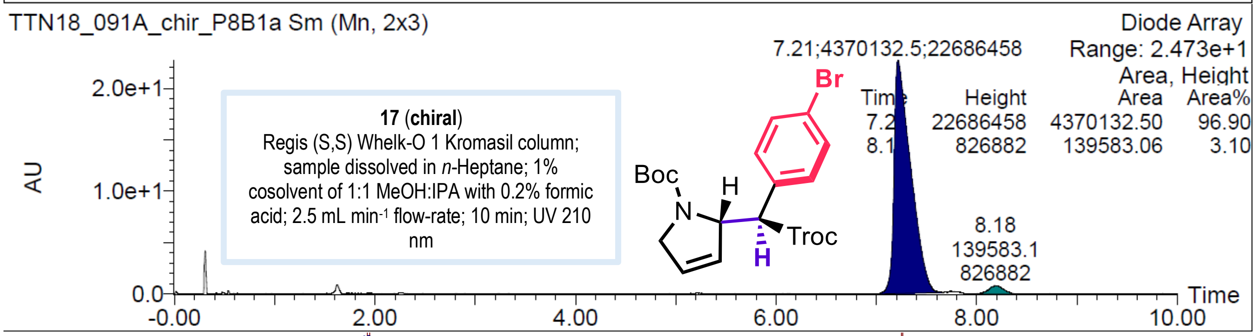
TTN18_091A_chir_P8B1a Sm (Mn, 2x3)
Diode Array
Range: 2.473e+1
Area, Height
7.21;4370132.5;22686458
17 (**chiral**)
Regis (S,S) Whelk-O 1 Kromasil column; sample dissolved in *n*-Heptane; 1% cosolvent of 1:1 MeOH:IPA with 0.2% formic acid; 2.5 mL min-1 flow-rate; 10 min; UV 210 nm
Height Area Area%
22686458 4370132.50 96.90
826882 139583.06 3.10
8.18
139583.1
826882
Boc
N
H
H
Troc
Br
AU
Time

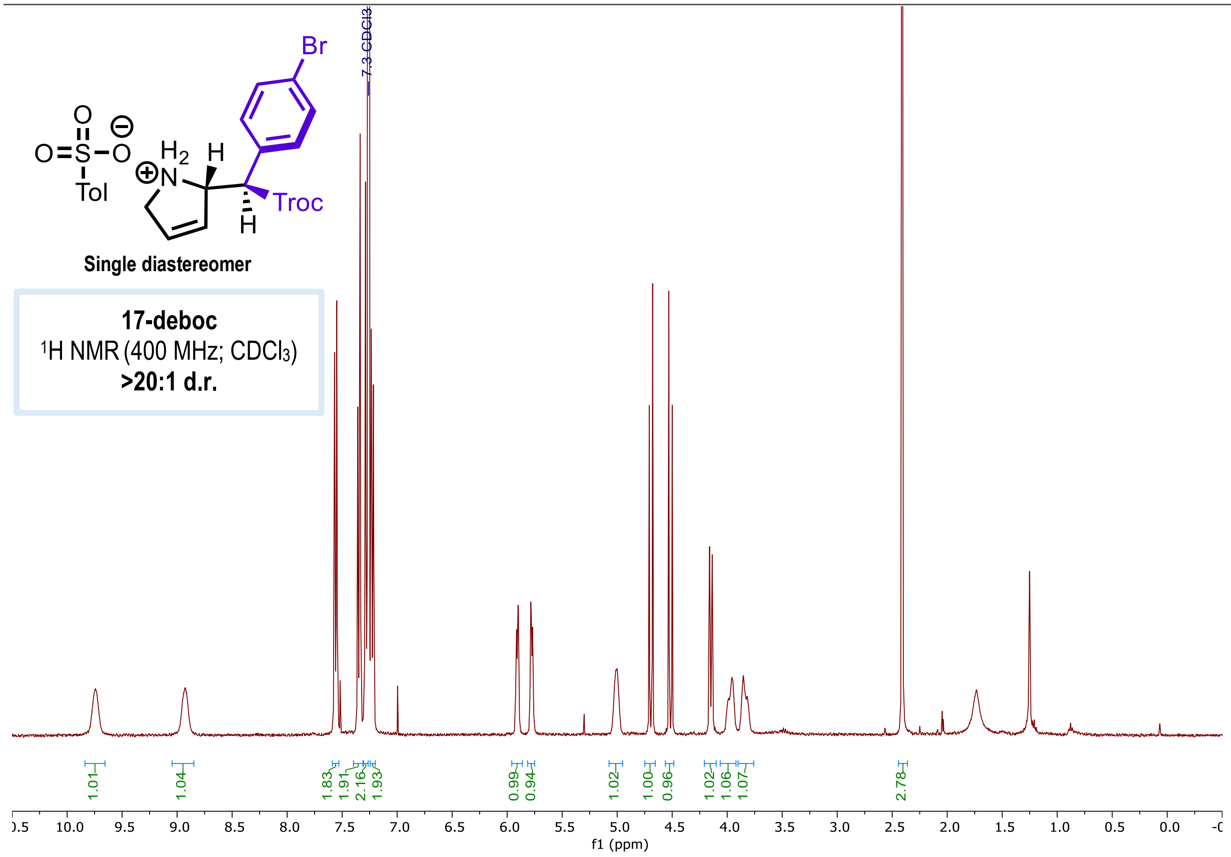
7.3 CDCl3
Br
Tol
Troc
Single diastereomer
17-deboc
1H NMR (400 MHz; CDCl3)
>20:1 d.r.
1.01
1.04
1.83
1.91
2.16
1.93
0.99
0.94
1.02
1.00
0.96
1.02
1.06
1.07
2.78
f1 (ppm)

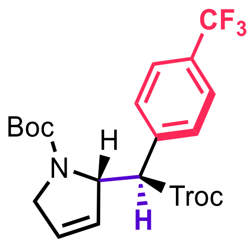

tert-butyl (S)-2-((R)-2-oxo-2-(2,2,2-trichloroethoxy)-1-(4-(trifluoromethyl)phenyl)ethyl)-2,5-dihydro-1H-pyrrole-1-carboxylate (18)

To an 8 mL vial charged with a stir-bar and 0.5 g of activated 4Å MS was added 2,2,2-trichloroethyl 2-diazo-2-(4-(trifluoromethyl)phenyl)acetate (181 mg, 1 equiv, 0.500 mmol) and tert-butyl 2,5-dihydro-1H-pyrrole-1-carboxylate (254 mg, 3 equiv, 1.50 mmol). The reaction vial was sealed, backfilled with nitrogen three times, and the vial topped with a nitrogen balloon. To the vial containing the diazo and olefin was added CH_2Cl_2 (1.5 mL). The solution was stirred at room temperature for at least 10 minutes. A stock solution of Rh_2(*S*-PTAD)$_4$ (390 µg, 500 µL, 0.0005 molar, 0.0005 equiv, 0.250 µmol) was added into the reaction solution containing diazo and olefin drop-wise over 15 seconds. The reaction mixture was then allowed to stir for 20 hours at room temperature. After the elapsed time, the reaction mixture was filtered through a celite plug and the filtrate concentrated *in vacuo* at 45 °C. The crude was dry-loaded onto SiO_2 and subjected to SiO_2 flash chromatography pentane/diethyl ether (Biotage Sfär DLV 50 g column as the running-column and Biotage Sfär DLV 50 g column for the dry-load; 100 mL/min flow-rate; 210/230 nm detector; 0% 6CV → 1% 6CV → 3% 6CV → 5% 6CV → 7% 6CV → 10% 6CV → 13% 6CV → 16% 12CV; POI elutes at 10% diethyl ether with trap). The collected fractions were concentrated *in vacuo* at 45 °C to afford a mixture of product and trap as a clear oil. The material was then dissolved in CH_2Cl_2, the solution passed through a celite plug into a 20 mL vial, and concentrated *in vacuo* at 45 °C. The vial containing the neat material was then subjected to Kugelrohr distillation at 8.0 x 10^{-1} torr vacuum and 95 °C air bath for at least 20 minutes to distill off the trap. The material was cooled to afford tert-butyl (S)-2-((R)-2-oxo-2-(2,2,2-trichloroethoxy)-1-(4-(trifluoromethyl)phenyl)ethyl)-2,5-dihydro-1H-pyrrole-1-carboxylate (171 mg, 340 µmol, 68.0% yield, 94% ee, >20:1 d.r.) as a pale-yellow oil.

^{1}H NMR (600 MHz, Chloroform-*d*; as a 0.6:0.4 mixture of rotamers) δ 7.66 – 7.51 (Ar-H m, 4H, mixture of rotamers), 6.00 – 5.90 (=C–H brm, 0.4H, minor rotamer), 5.89 – 5.66 (=C–H brm, 1.6H, mixture of rotamers [0.4H minor rotamer + 1.2H major rotamer = 1.6H]), 5.07 – 4.97 (C–$H_{\alpha\text{-}N,C\text{-}2}$ brm, 0.6H, major rotamer), 4.89 – 4.83 (C–$H_{\alpha\text{-}N,C\text{-}2}$ brm, 0.4H, minor rotamer), 4.81 (C–$H_{methylene}$ d, *J* = 12.2 Hz, 0.6H, major rotamer), 4.78 (C–$H_{methylene}$ d, *J* = 12.2 Hz, 0.4H, minor rotamer), 4.71 (C–$H_{methylene}$ brd, *J* = 12.2 Hz, 1H, mixture of rotamers [0.4H minor rotamer + 0.6H major rotamer = 1H]), 4.64 (C–$H_{benzylic}$ d, *J* = 3.9 Hz, 0.6H, major rotamer), 4.50 (C–$H_{benzylic}$ d, *J* = 3.9 Hz, 0.4H, minor rotamer), 4.28 (C–$H_{\alpha\text{-}N,C\text{-}5}$ d, *J* = 15.6 Hz, 0.4H, minor rotamer), 4.12 (C–$H_{\alpha\text{-}N,C\text{-}5}$ d, *J* = 15.6 Hz, 0.6H, major rotamer), 3.98 (C–$H_{\alpha\text{-}N,C\text{-}5}$ dd, *J* = 15.6, 5.0 Hz, 0.4H, minor rotamer), 3.88 (C–$H_{\alpha\text{-}N,C\text{-}5}$ dd, *J* = 15.6, 5.0 Hz, 0.6H, major rotamer), 1.50 (–C(CH_3)$_3$ s, 3.6H, minor rotamer), 1.48 (–C(CH_3)$_3$ s, 5.4H, major rotamer).

^{13}C NMR (151 MHz, Chloroform-*d*; as a mixture of rotamers) δ 169.4, 169.1, 154.4, 153.8, 138.6, 129.7, 129.2, 128.4, 127.9, 126.4, 126.2, 125.6, 125.3, 125.3, 94.8, 94.5, 80.7, 80.2, 74.5, 74.4, 68.0, 67.9, 54.3, 54.2, 54.1, 53.0, 28.5.

^{19}F NMR (376 MHz, Chloroform-*d*; as a mixture of rotamers) δ -62.6, -62.7.

HRMS (APCI -) calc. mass for $C_{20}H_{20}O_4N^{35}Cl_3F_3$ [M - H]$^-$ 500.0416; obs. mass for $C_{20}H_{20}O_4N^{35}Cl_3F_3$ [M - H]$^-$ 500.0411.

[α]$^{20}_D$: -102.1° (c = 0.680 g/100 mL, EtOAc).

SFC (Regis (S,S) Whelk-O 1 Kromasil column; sample dissolved in *n*-Heptane; 1% cosolvent of 1:1 MeOH:IPA with 0.2% formic acid; 2.5 mL min^{-1} flow-rate; 10 min; UV 230 nm): retention times of 3.08 min (major) and 3.53 min (minor); 94% ee with Rh_2(*S*-PTAD)$_4$.

Determination of d.r.: *N*-Boc deprotection (1.5 equiv of *p*-Toluenesulfonic acid monohydrate in EtOAc [0.1 M] at 60 °C, 4 hours); observed >20:1 d.r.

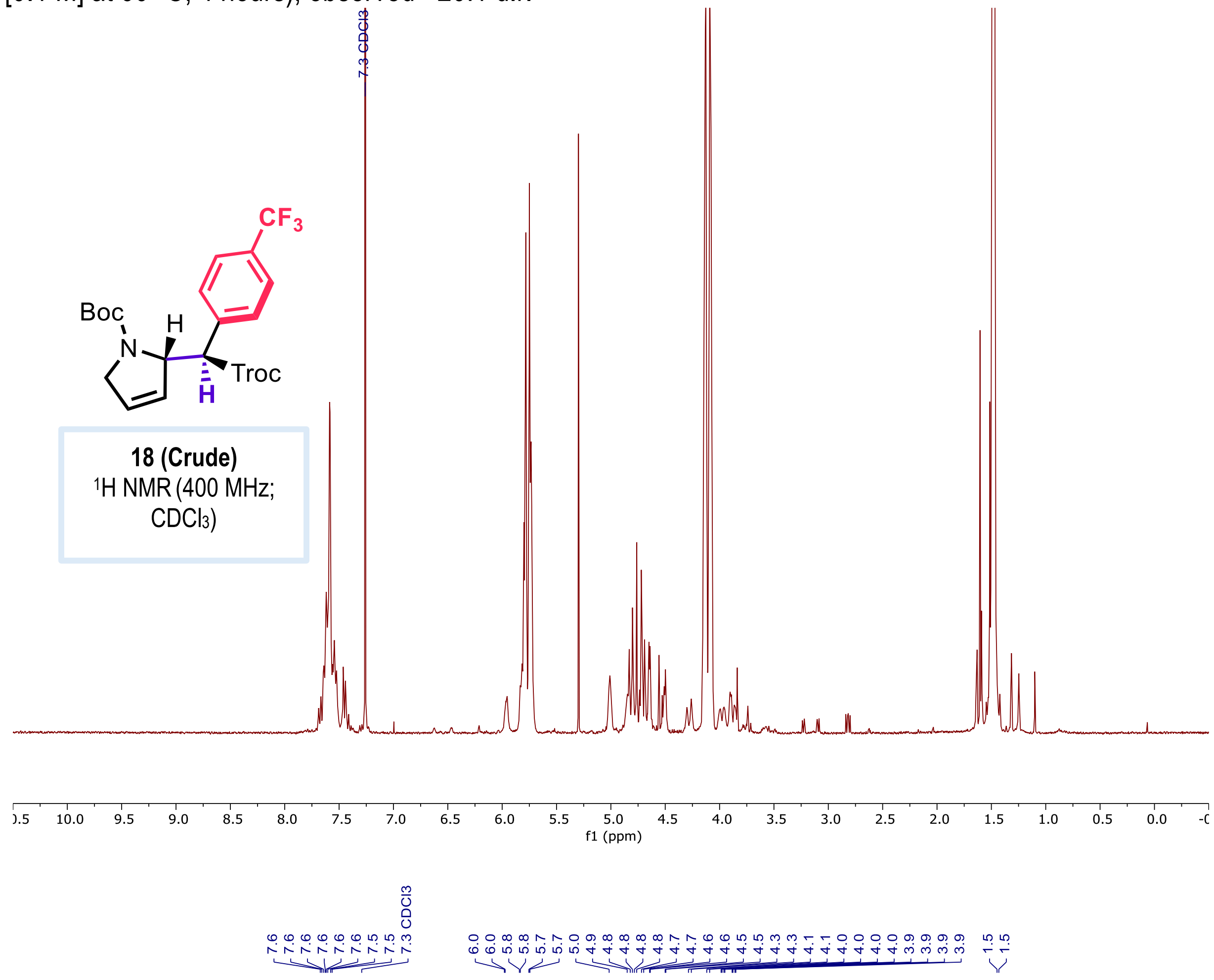

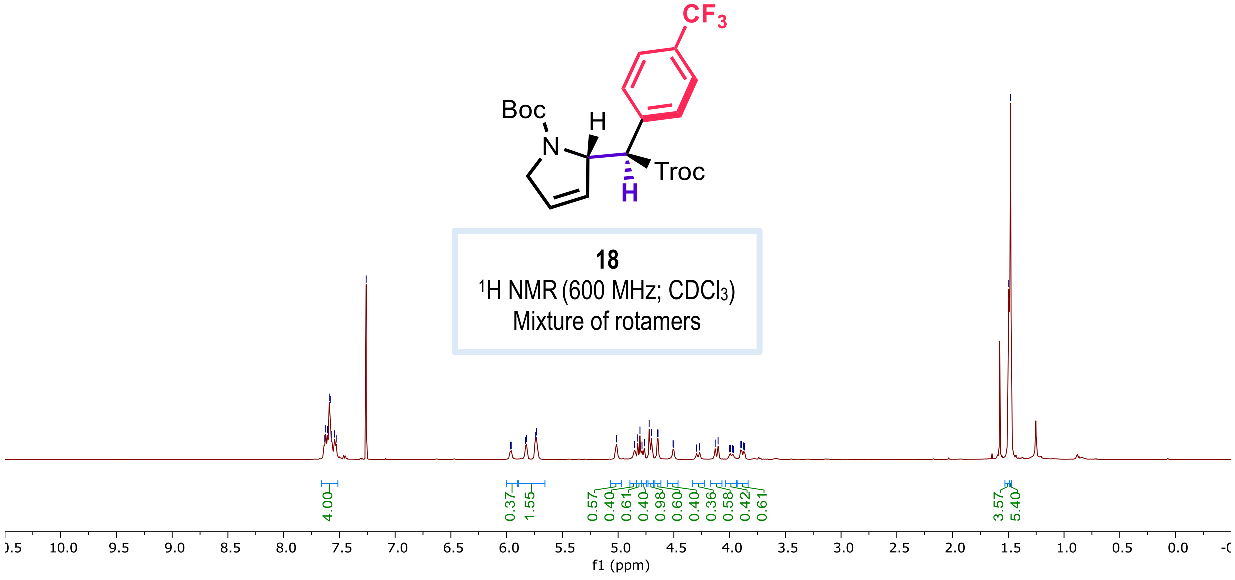

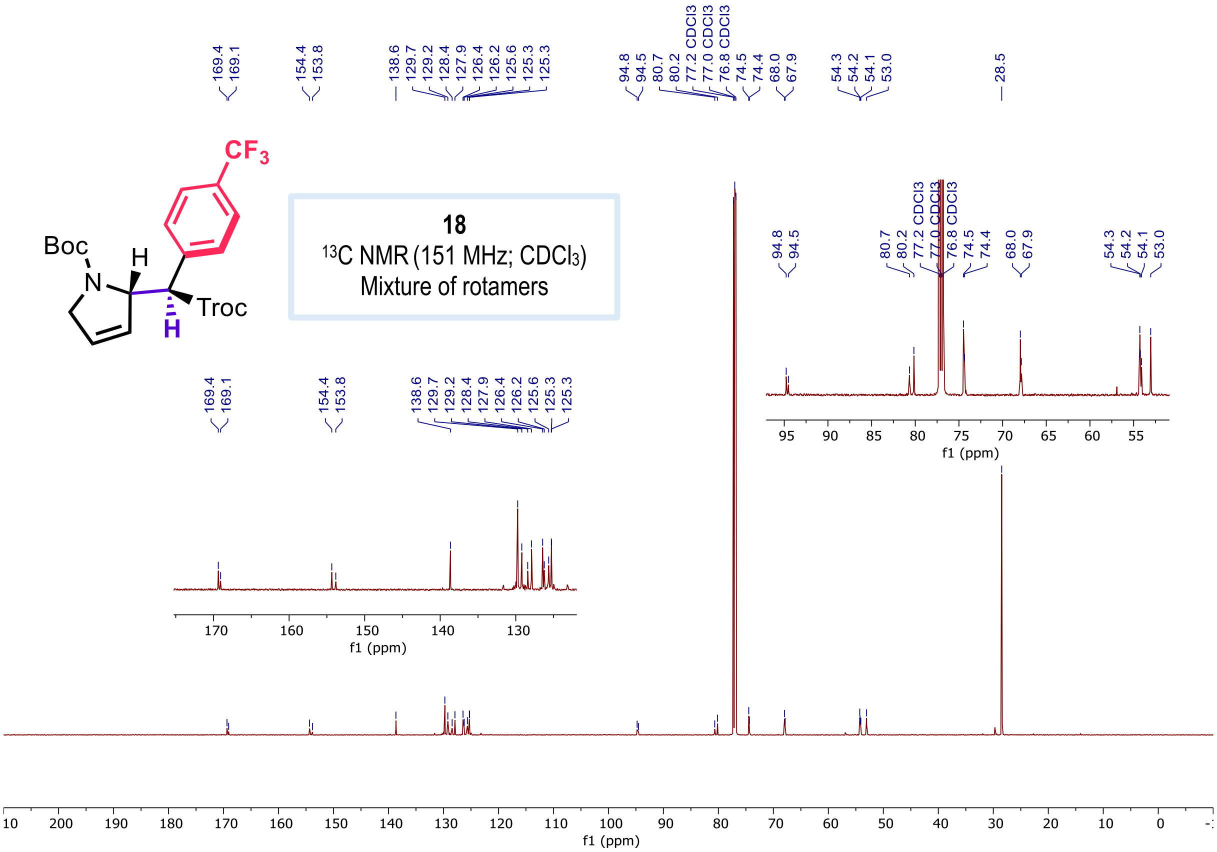
18
13C NMR (151 MHz; CDCl3)
Mixture of rotamers
Boc
N
H
H
Troc
CF3
169.4
169.1
154.4
153.8
138.6
129.7
129.2
128.4
127.9
126.4
126.2
125.6
125.3
125.3
94.8
94.5
80.7
80.2
77.2 CDCl3
77.0 CDCl3
76.8 CDCl3
74.5
74.4
68.0
67.9
54.3
54.2
54.1
53.0
28.5
f1 (ppm)

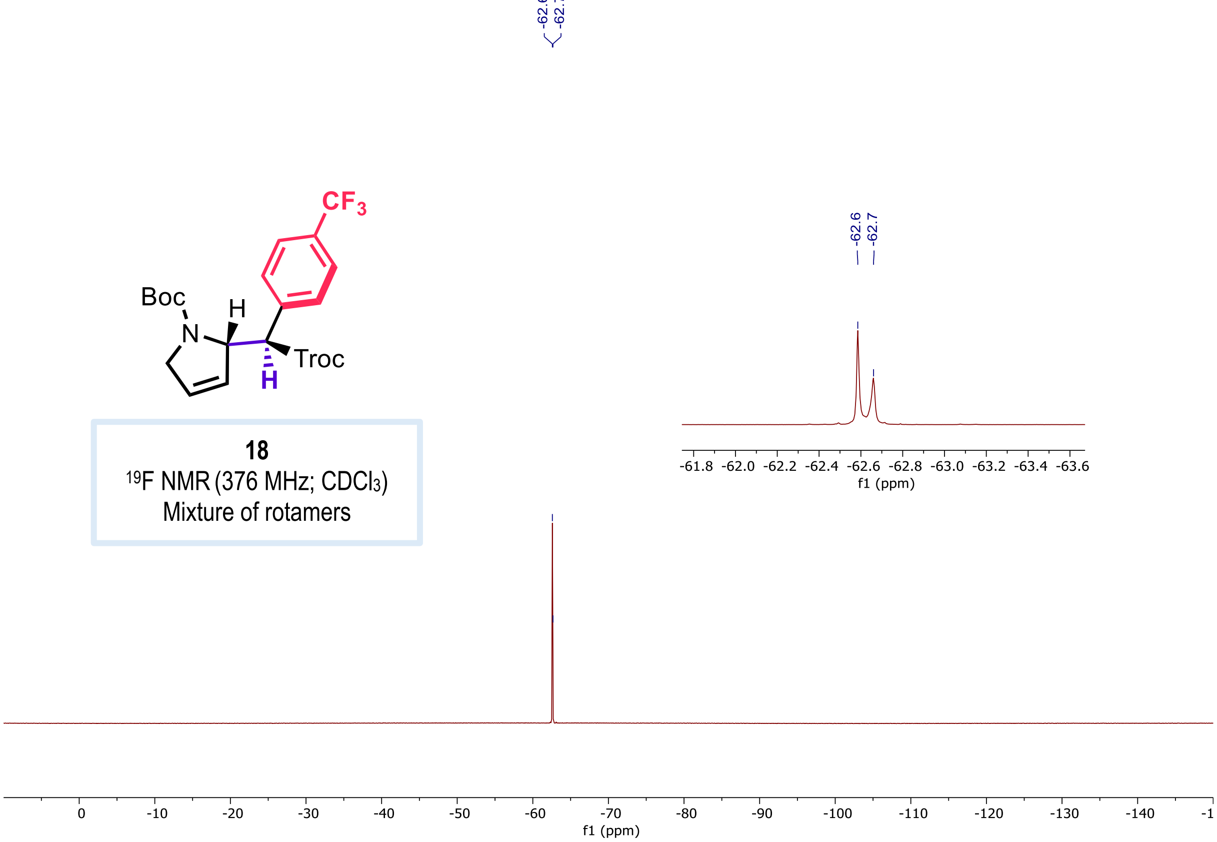
18
19F NMR (376 MHz; CDCl3)
Mixture of rotamers
Boc
N
H
H
Troc
CF3
-62.6
-62.7
f1 (ppm)

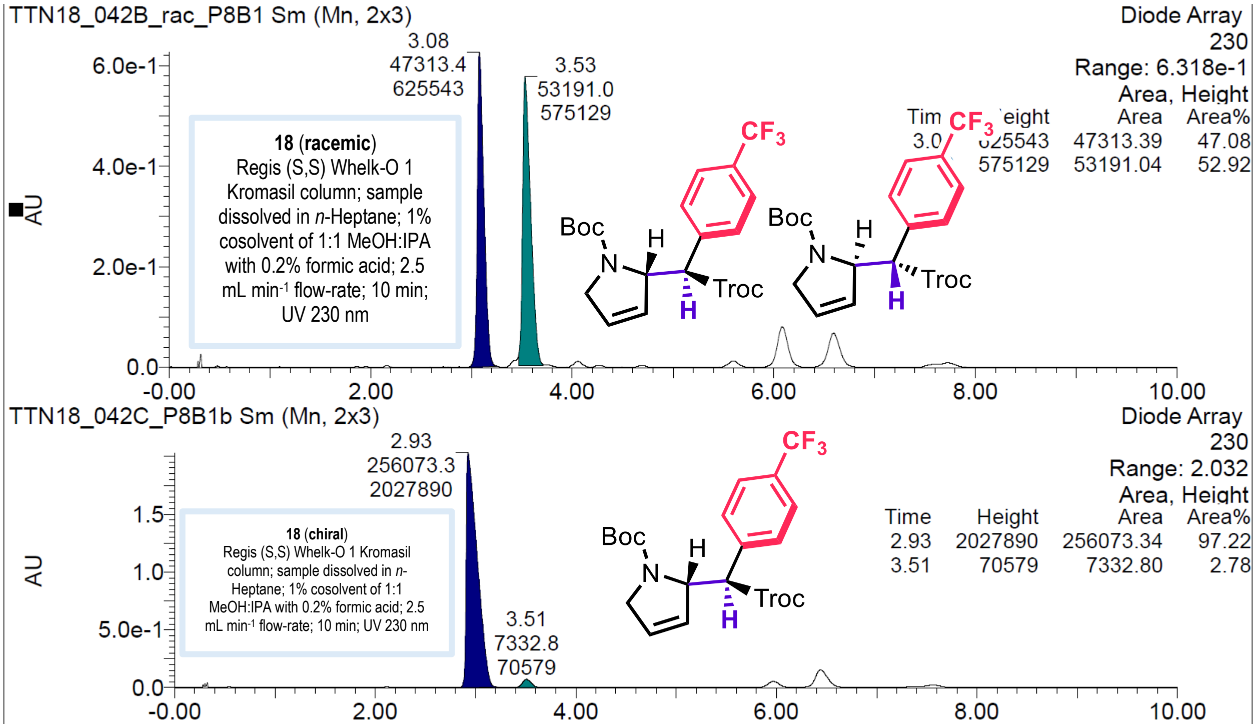
TTN18_042B_rac_P8B1 Sm (Mn, 2x3)
Diode Array
230
Range: 6.318e-1
Area, Height
3.08
47313.4
625543
3.53
53191.0
575129
18 (racemic)
Regis (S,S) Whelk-O 1
Kromasil column; sample
dissolved in n-Heptane; 1%
cosolvent of 1:1 MeOH:IPA
with 0.2% formic acid; 2.5
mL min-1 flow-rate; 10 min;
UV 230 nm
Area
Area%
47313.39
47.08
53191.04
52.92
625543
575129
AU
CF3
Boc
N
H
Troc
H
TTN18_042C_P8B1b Sm (Mn, 2x3)
Diode Array
230
Range: 2.032
Area, Height
2.93
256073.3
2027890
18 (chiral)
Regis (S,S) Whelk-O 1 Kromasil
column; sample dissolved in n-
Heptane; 1% cosolvent of 1:1
MeOH:IPA with 0.2% formic acid; 2.5
mL min-1 flow-rate; 10 min; UV 230 nm
3.51
7332.8
70579
Time
Height
Area
Area%
2.93
2027890
256073.34
97.22
3.51
70579
7332.80
2.78
AU

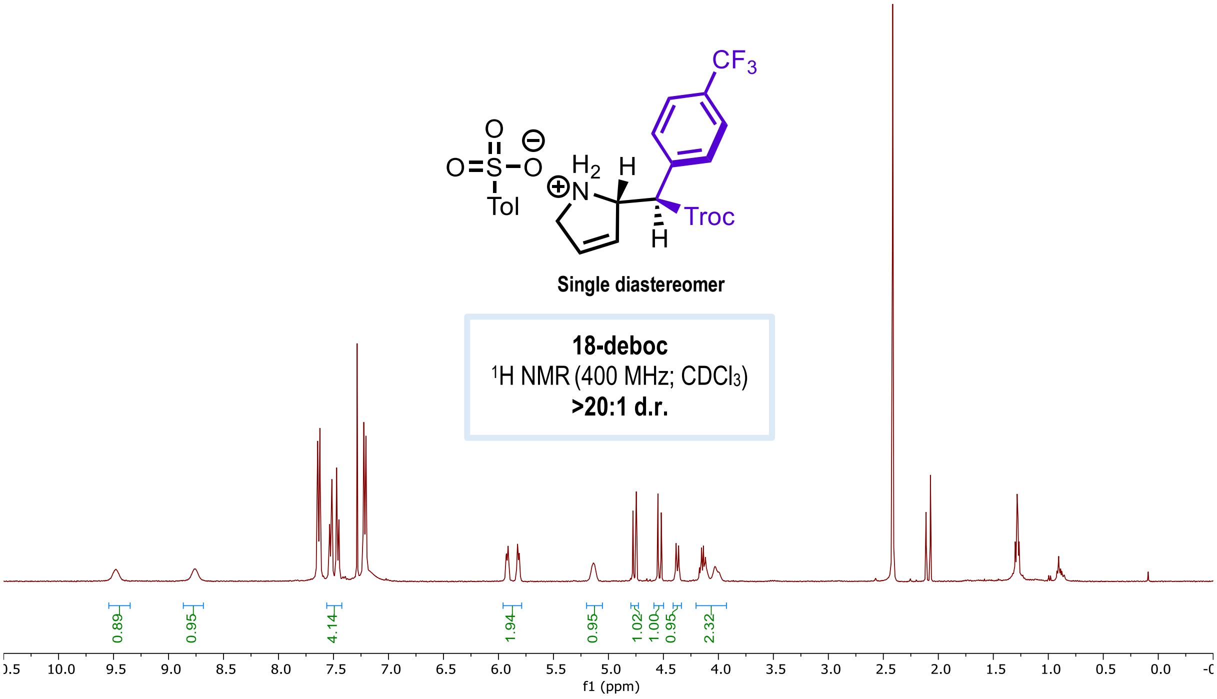
CF3
O
O=S-O
Tol
H2
N
H
Troc
H
Single diastereomer
18-deboc
1H NMR (400 MHz; CDCl3)
>20:1 d.r.
0.89
0.95
4.14
1.94
0.95
1.02
1.00
0.95
2.32
f1 (ppm)

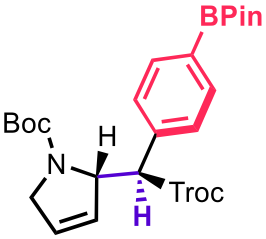
BPin
Boc
N
H
Troc
H

tert-butyl (S)-2-((R)-2-oxo-1-(4-(4,4,5,5-tetramethyl-1,3,2-dioxaborolan-2-yl)phenyl)-2-(2,2,2-trichloroethoxy)ethyl)-2,5-dihydro-1H-pyrrole-1-carboxylate (19)

To an 8 mL vial charged with a stir-bar and 0.4 g of activated 4Å MS was added 2,2,2-trichloroethyl 2-diazo-2-(4-(4,4,5,5-tetramethyl-1,3,2-dioxaborolan-2-yl)phenyl)acetate (171 mg, 1 equiv, 408 μmol) and tert-butyl 2,5-dihydro-1H-pyrrole-1-carboxylate (207 mg, 3 equiv, 1.22 mmol). The reaction vial was sealed, backfilled with nitrogen three times, and the vial topped with a nitrogen balloon. To the vial containing the diazo and olefin was added CH_2Cl_2 (1.22 mL). The solution was stirred at room temperature for at least 10 minutes. A stock solution of Rh_2(*S*-PTAD)$_4$ (318 μg, 408 μL CH_2Cl_2, 0.0005 molar, 0.0005 equiv, 0.204 μmol) was added into the reaction solution containing diazo and olefin drop-wise over 15 seconds. The reaction mixture was then allowed to stir for 20 hours at room temperature. After the elapsed time, the reaction mixture was filtered through a celite plug and the filtrate concentrated *in vacuo* at 45 °C. The crude was dry-loaded onto SiO_2 and subjected to SiO_2 flash chromatography pentane/diethyl ether (Biotage Sfär DLV 50 g column as running-column and Biotage Sfär DLV 50 g column for the dry-load; 100 mL/min flow-rate; 210/230 nm detector; 0% 6CV → 1% 6CV → 3% 6CV → 5% 6CV → 7% 6CV →10% 6CV → 13% 6CV → 16% 12CV; POI elutes at 16% diethyl ether). The collected fractions were concentrated *in vacuo* at 45 °C to afford tert-butyl (S)-2-((R)-2-oxo-1-(4-(4,4,5,5-tetramethyl-1,3,2-dioxaborolan-2-yl)phenyl)-2-(2,2,2-trichloroethoxy)ethyl)-2,5-dihydro-1H-pyrrole-1-carboxylate (111 mg, 198 μmol, 48.6% yield, 96% ee, >20:1 d.r.) as a clear pale yellow oil.

^{1}H NMR (600 MHz, Chloroform-*d*; as a 0.55:0.45 mixture of rotamers) δ 7.80 (Ar–H d, *J* = 7.6 Hz, 0.9H, minor rotamer), 7.78 (Ar–H d, *J* = 7.6 Hz, 1.1H, major rotamer), 7.45 (Ar–H d, *J* = 7.6 Hz, 1.1H, major rotamer), 7.39 (Ar–H d, *J* = 7.6 Hz, 0.9H, minor rotamer), 5.99 – 5.87 (=C–H brm, 0.45H, minor rotamer), 5.86 – 5.67 (=C–H brm, 1.55H, mixture of rotamers [0.45H minor rotamer + 1.1H major rotamer = 1.55H]), 4.99 – 4.89 (C–$H_{\alpha\text{-}N,C\text{-}2}$ brm, 0.55H, major rotamer), 4.84 (C–$H_{methylene}$ d, *J* = 12.1 Hz, 0.55H, major rotamer), 4.78 (C–$H_{methylene}$ & C–$H_{\alpha\text{-}N,C\text{-}2}$ d & brm, *J* = 12.1 Hz, 0.9H, minor rotamers [0.45H minor rotamer + 0.45H minor rotamer = 0.9H]), 4.72 (C–$H_{benzylic}$ d, *J* = 4.7 Hz, 0.55H, major rotamer), 4.69 – 4.61 (C–$H_{methylene}$ overlapping d's, *J* = 12.1 Hz each, 1H, mixture of rotamers [0.45H minor rotamer + 0.55H major rotamer = 1H]), 4.56 (C–$H_{benzylic}$ d, *J* = 4.7 Hz, 0.45H, minor rotamer), 4.25 (C–$H_{\alpha\text{-}N,C\text{-}5}$ d, *J* = 15.4 Hz, 0.45H, minor rotamer), 4.10 (C–$H_{\alpha\text{-}N,C\text{-}5}$ d, *J* = 15.4 Hz, 0.55H, major rotamer), 4.00 (C–$H_{\alpha\text{-}N,C\text{-}5}$ dd, *J* = 15.4, 4.6 Hz, 0.45H, minor rotamer), 3.92 (C–$H_{\alpha\text{-}N,C\text{-}5}$ dd, *J* = 15.4, 4.6 Hz, 0.55H, major rotamer), 1.53 (–C(CH_3)$_3$ s, 4.05H, minor rotamer), 1.49 (–C(CH_3)$_3$ s, 4.95H, major rotamer), 1.34 (–CH_3 s, 12H, mixture of rotamers).
^{13}C NMR (151 MHz, Chloroform-*d*; as a mixture of rotamers) δ 169.7, 169.3, 154.3, 153.9, 137.7, 135.2, 134.9, 128.6, 128.0, 127.7, 127.3, 126.7, 126.5, 94.9, 94.7, 83.9, 83.9, 80.5, 79.9, 74.2, 74.2, 68.2, 68.1, 54.4, 54.2, 54.1, 52.9, 28.5, 24.9.
HRMS (APCI -) calc. mass for $C_{25}H_{32}O_6N^{11}B^{35}Cl_3$ [M - H]$^-$ 558.1394; obs. mass for $C_{25}H_{32}O_6N^{11}B^{35}Cl_3$ [M - H]$^-$ 558.1387.
$[\alpha]^{20}_D$: -115.8° (c = 0.090 g/100 mL, EtOAc).
SFC (ChiralCel OJ-3 column; sample dissolved in *n*-Heptane; 1% cosolvent of 1:1 MeOH:IPA with 0.2% formic acid; 2.5 mL min^{-1} flow-rate; 10 min; UV 230 nm): retention times of 1.78 min (minor) and 2.39 min (major); 96% ee with Rh_2(*S*-PTAD)$_4$.
Determination of d.r.: *N*-Boc deprotection (1.5 equiv of *p*-Toluenesulfonic acid monohydrate in EtOAc [0.1 M] at 60 °C, 4 hours); observed >20:1 d.r.

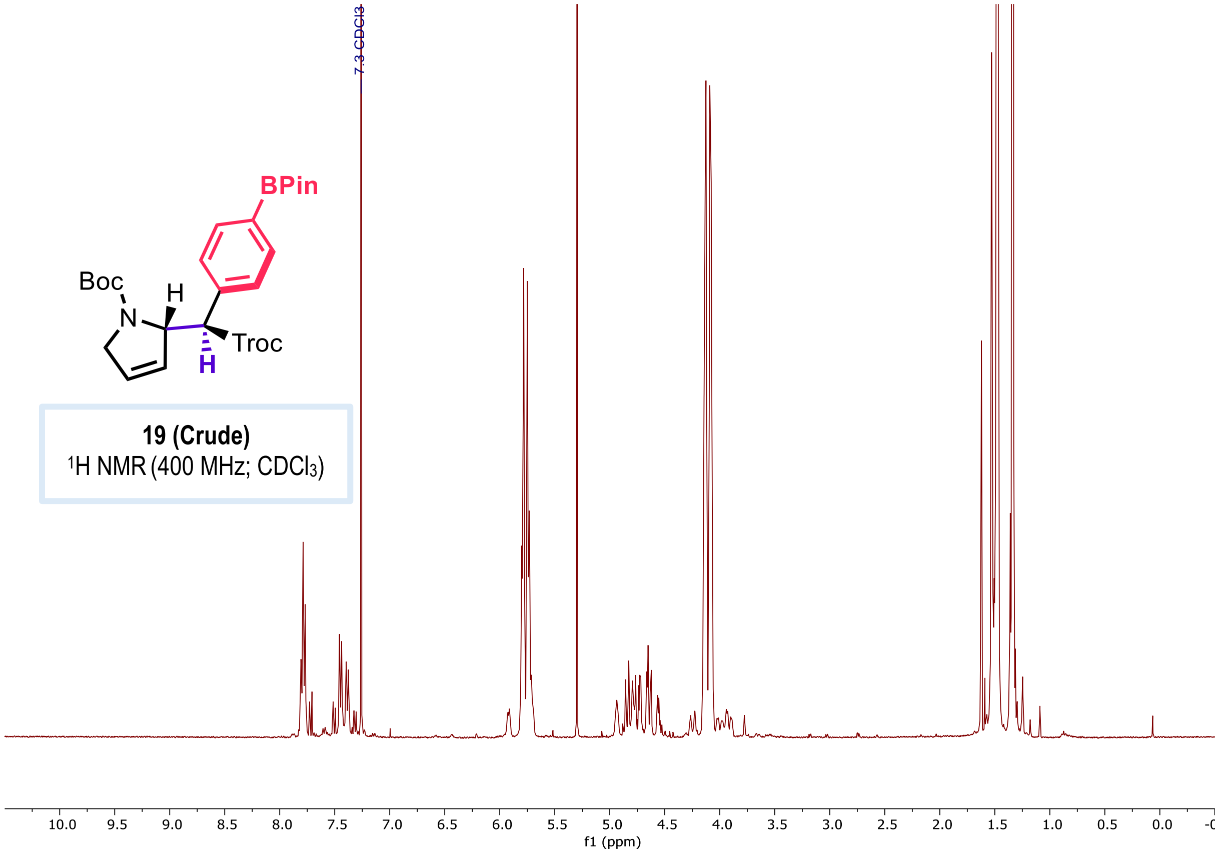

7.3 CDCl3
BPin
Boc
H
N
Troc
H
19 (Crude)
^{1}H NMR (400 MHz; CDCl$_3$)
f1 (ppm)

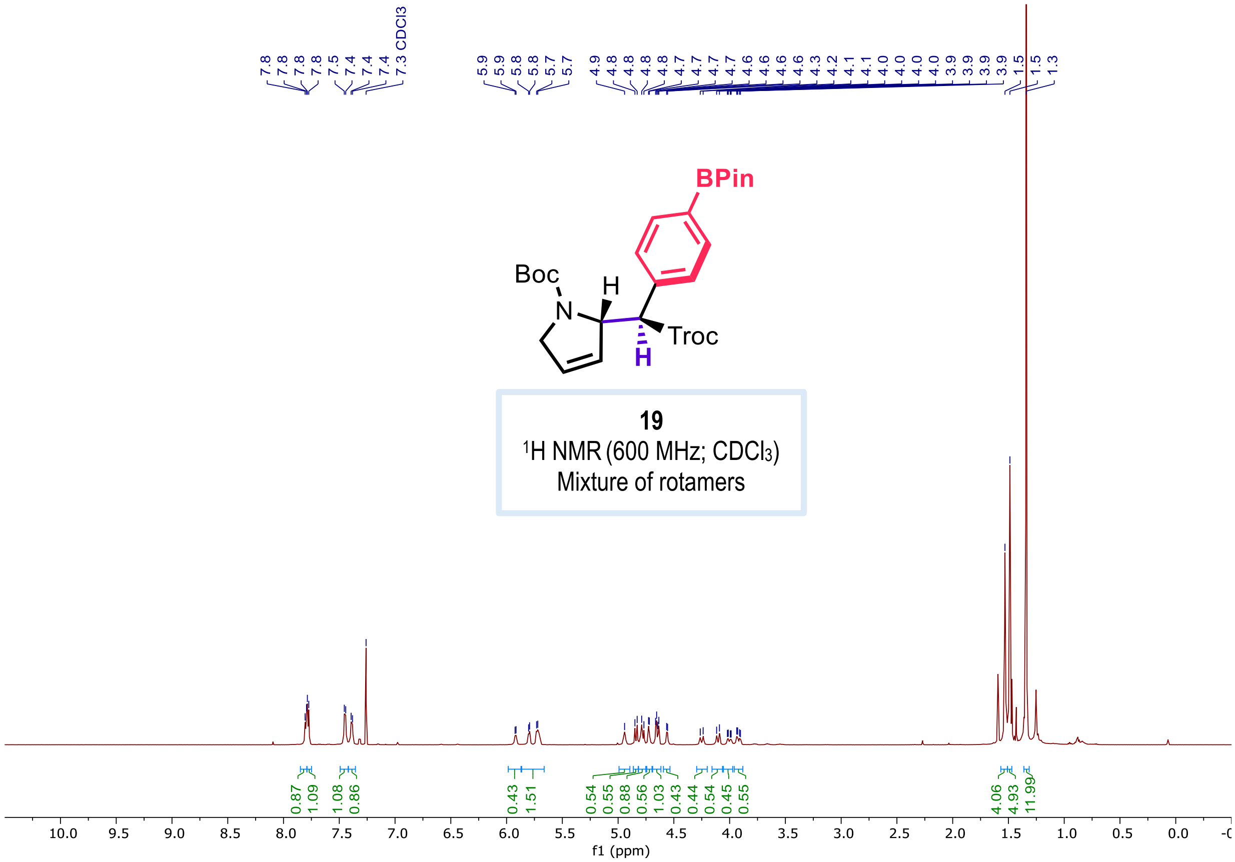

7.8
7.8
7.8
7.8
7.5
7.4
7.4
7.4
7.3 CDCl3
5.9
5.9
5.8
5.8
5.7
5.7
4.9
4.8
4.8
4.8
4.8
4.7
4.7
4.7
4.7
4.6
4.6
4.6
4.6
4.3
4.2
4.1
4.1
4.0
4.0
4.0
4.0
3.9
3.9
3.9
3.9
1.5
1.5
1.3
BPin
Boc
H
N
Troc
H
19
^{1}H NMR (600 MHz; CDCl$_3$)
Mixture of rotamers
0.87
1.09
1.08
0.86
0.43
1.51
0.54
0.55
0.88
0.56
1.03
0.43
0.44
0.54
0.45
0.55
4.06
4.93
11.99
f1 (ppm)

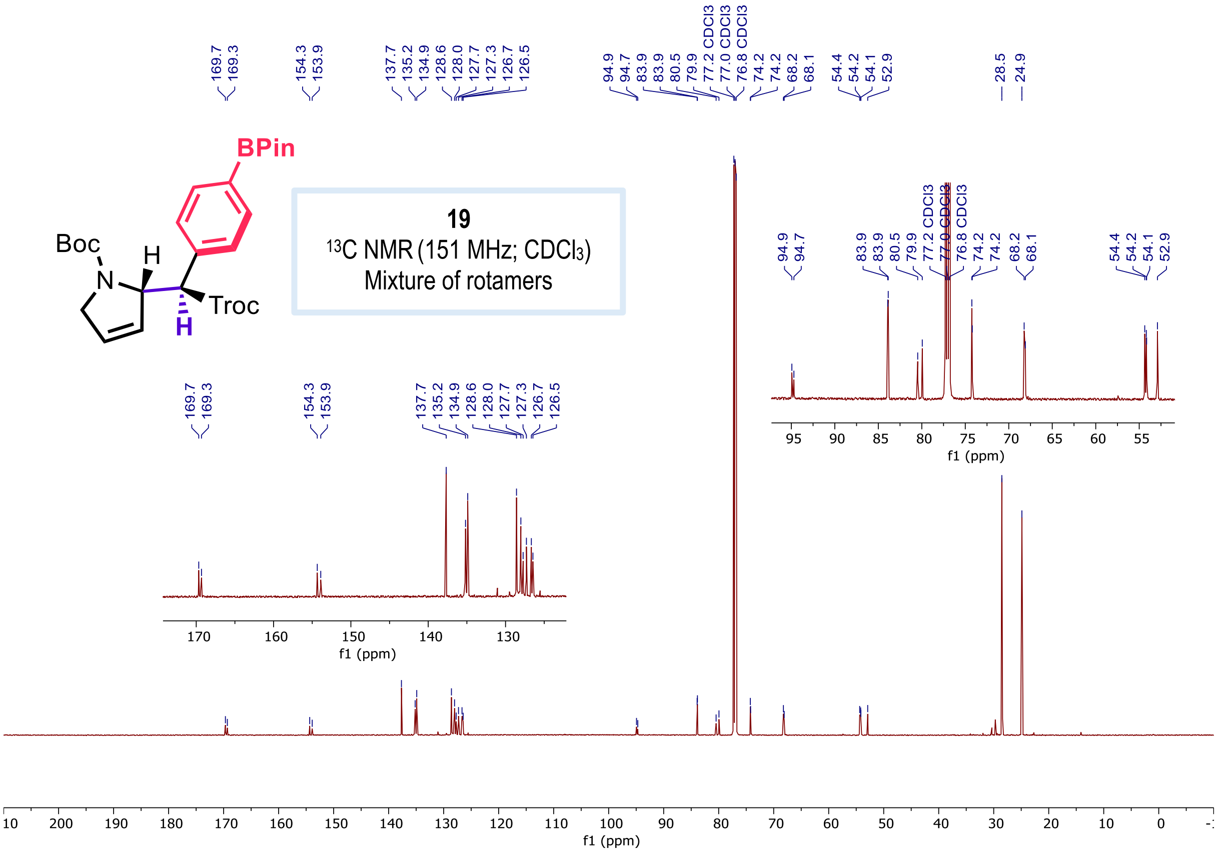

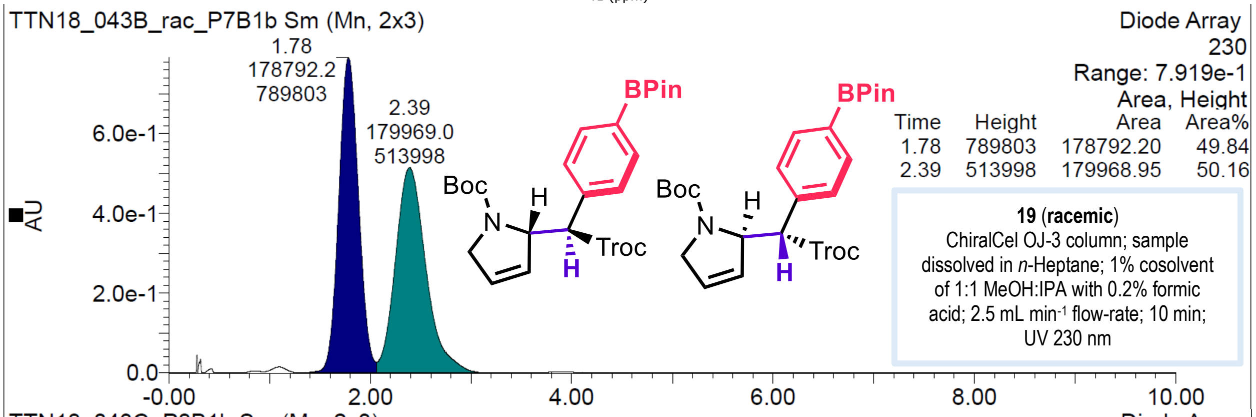

Time	Height	Area	Area%
1.78	789803	178792.20	49.84
2.39	513998	179968.95	50.16

19 (**racemic**)
ChiralCel OJ-3 column; sample dissolved in *n*-Heptane; 1% cosolvent of 1:1 MeOH:IPA with 0.2% formic acid; 2.5 mL min^{-1} flow-rate; 10 min; UV 230 nm

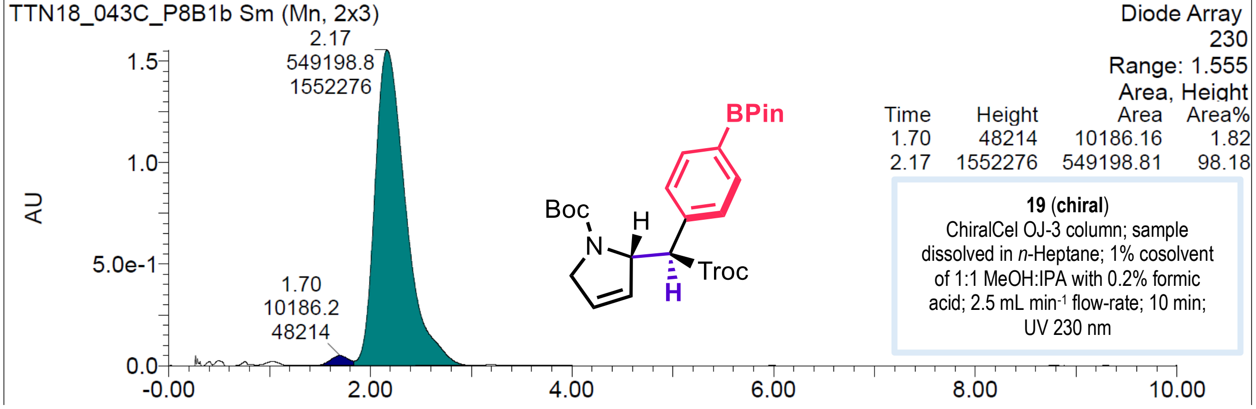

Time	Height	Area	Area%
1.70	48214	10186.16	1.82
2.17	1552276	549198.81	98.18

19 (**chiral**)
ChiralCel OJ-3 column; sample dissolved in *n*-Heptane; 1% cosolvent of 1:1 MeOH:IPA with 0.2% formic acid; 2.5 mL min^{-1} flow-rate; 10 min; UV 230 nm

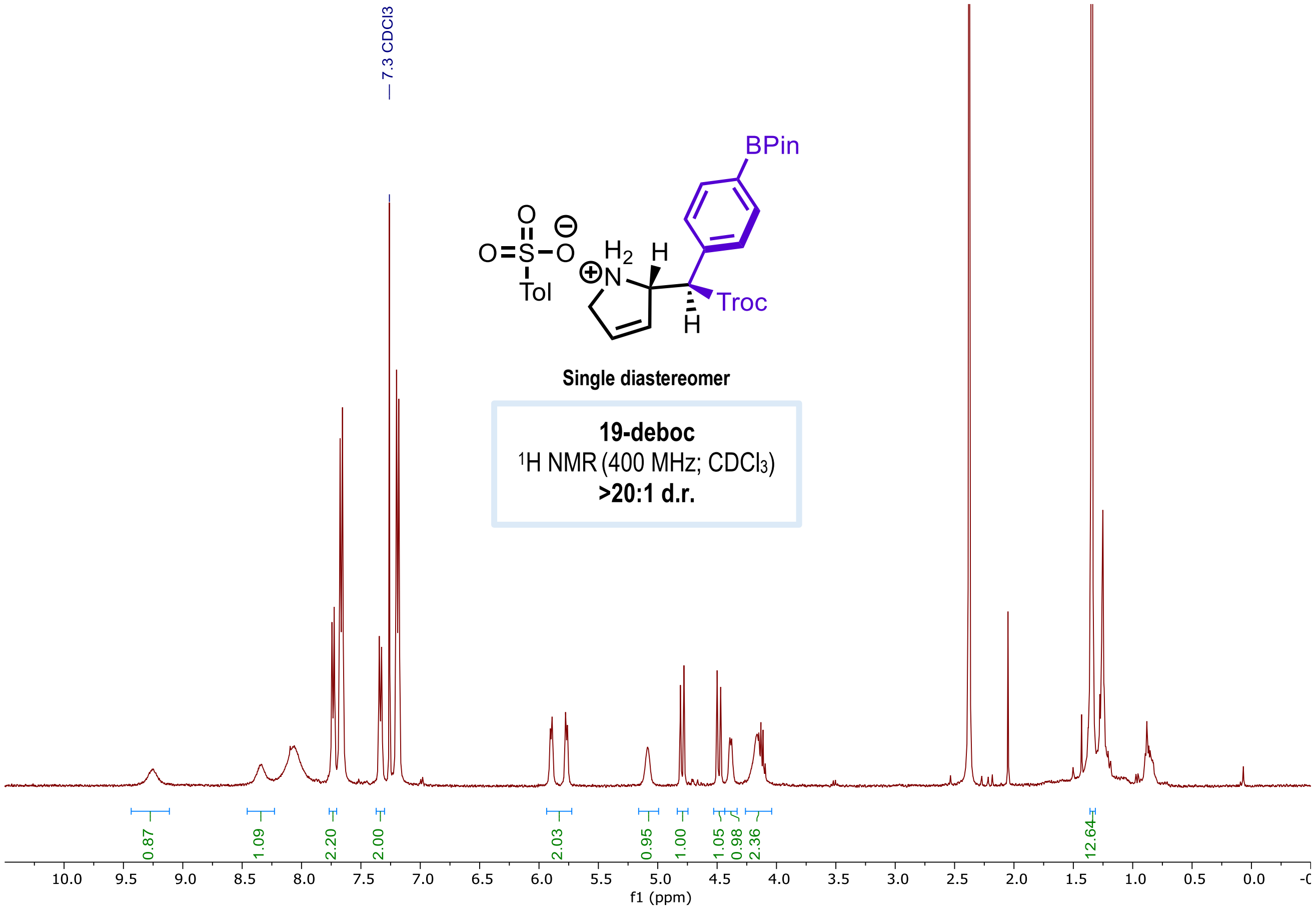

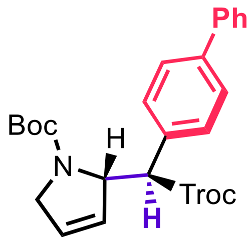

tert-butyl (S)-2-((R)-1-([1,1'-biphenyl]-4-yl)-2-oxo-2-(2,2,2-trichloroethoxy)ethyl)-2,5-dihydro-1H-pyrrole-1-carboxylate (20)

To an 8 mL vial charged with a stir-bar and 0.5 g of activated 4Å MS was added 2,2,2-trichloroethyl 2-([1,1'-biphenyl]-4-yl)-2-diazoacetate (185 mg, 1 equiv, 0.500 mmol) and tert-butyl 2,5-dihydro-1H-pyrrole-1-carboxylate (254 mg, 3 equiv, 1.50 mmol). The reaction vial was sealed, backfilled with nitrogen three times, and the vial topped with a nitrogen balloon. To the vial containing the diazo and olefin was added CH_2Cl_2 (1.5 mL). The solution was stirred at room temperature for at least 10 minutes. A stock solution of Rh_2(*S*-PTAD)$_4$ (390 µg, 500 µL CH_2Cl_2, 0.0005 molar, 0.0005 equiv, 0.250 µmol) was added into the reaction solution containing diazo and olefin drop-wise over 15 seconds. The reaction mixture was then allowed to stir for 20 hours at room temperature. After the elapsed time, the reaction mixture was filtered through a celite plug and the filtrate concentrated *in vacuo* at 45 ^oC. The crude was dry-loaded onto SiO_2 and subjected to SiO_2 flash chromatography pentane/diethyl ether (Biotage Sfär DLV 50 g column as the running-column and Biotage Sfär DLV 50 g column for the dry-load; 100 mL/min flow-rate; 210/230 nm detector; 0% 6CV → 1% 6CV → 3% 6CV → 5% 6CV → 7% 6CV → 10% 6CV → 13% 6CV → 16% 12CV; POI elutes at 10% ether with trap). The collected fractions were concentrated *in vacuo* at 45 ^oC to

afford tert-butyl (S)-2-((R)-1-([1,1'-biphenyl]-4-yl)-2-oxo-2-(2,2,2-trichloroethoxy)ethyl)-2,5-dihydro-1H-pyrrole-1-carboxylate (185 mg, 362 μmol, 72.4% yield) as a clear oil. The material was then dissolved in CH_2Cl_2, the solution passed through a celite plug into a 20 mL vial, and concentrated *in vacuo* at 45 °C. The vial containing the neat material was then subjected to Kugelrohr distillation at 8.0 x 10^{-1} torr vacuum and 95 °C air bath for at least 20 minutes to distill off the trap. The material was cooled to afford tert-butyl (S)-2-((R)-1-([1,1'-biphenyl]-4-yl)-2-oxo-2-(2,2,2-trichloroethoxy)ethyl)-2,5-dihydro-1H-pyrrole-1-carboxylate (185 mg, 362 μmol, 72.4% yield, 96% ee, >20:1 d.r.) as a pale-yellow oil.

^{1}H NMR (600 MHz, Chloroform-*d*; as a 0.55:0.45 mixture of rotamers) δ 7.66 – 7.50 (Ar–H m, 5H, mixture of rotamers), 7.50 – 7.32 (Ar–H m, 4H, mixture of rotamers), 6.00 – 5.93 (=C–H brm, 0.45H, minor rotamer), 5.88 – 5.79 (=C–H brm, 1.55, mixture of rotamers [0.45H minor rotamer + 1.1H major rotamer = 1.55H]), 5.04 – 4.94 (C–$H_{\alpha\text{-}N,C\text{-}2}$ brm, 0.55H, major rotamer), 4.87 (C–$H_{methylene}$ & C–$H_{\alpha\text{-}N,C\text{-}2}$ d & brm, *J* = 12.0 Hz, 0.9H, minor rotamers [0.45H minor rotamer + 0.45H minor rotamer = 0.9H]), 4.82 (C–$H_{methylene}$ d, *J* = 12.0 Hz, 0.55H, major rotamer), 4.74 (C–$H_{benzylic}$ d, *J* = 4.3 Hz, 0.55H, major rotamer), 4.67 (C–$H_{methylene}$ d, *J* = 12.0 Hz, 1H, mixture of rotamers [0.45H minor rotamer + 0.55H major rotamer = 1H]), 4.54 (C–$H_{benzylic}$ d, *J* = 4.3 Hz, 0.45H, minor rotamer), 4.29 (C–$H_{\alpha\text{-}N,C\text{-}5}$ d, *J* = 15.4 Hz, 0.45H, minor rotamer), 4.14 (C–$H_{\alpha\text{-}N,C\text{-}5}$ d, *J* = 15.4 Hz, 0.55H, major rotamer), 4.03 (C–$H_{\alpha\text{-}N,C\text{-}5}$ dd, *J* = 15.4, 5.3 Hz, 0.45H, minor rotamer), 3.96 (C–$H_{\alpha\text{-}N,C\text{-}5}$ dd, *J* = 15.4, 5.3 Hz, 0.55H, major rotamer), 1.53 (–$C(CH_3)_3$ s, 4.05H, minor rotamer), 1.50 (–$C(CH_3)_3$ s, 4.95H, major rotamer).
^{13}C NMR (151 MHz, Chloroform-*d*; as a mixture of rotamers) δ 169.9, 169.6, 154.4, 154.3, 154.0, 140.7, 140.6, 140.5, 140.5, 133.7, 129.6, 129.2, 128.9, 128.8, 128.8, 127.8, 127.5, 127.4, 127.4, 127.1, 127.1, 126.8, 126.7, 125.9, 125.8, 95.0, 94.7, 80.5, 80.0, 79.3, 74.3, 74.3, 68.3, 68.1, 54.4, 54.1, 54.0, 53.1, 52.9, 52.6, 28.6, 28.5.
HRMS (ESI +) calc. mass for $C_{25}H_{27}O_4N^{35}Cl_3$ $[M + H]^+$ 510.1000; obs. mass for $C_{25}H_{27}O_4N^{35}Cl_3$ $[M + H]^+$ 510.1007.
$[\alpha]^{20}_D$: -96.4° (c = 0.150 g/100 mL, EtOAc).
SFC (Regis (S,S) Whelk-O 1 Kromasil column; sample dissolved in *n*-Heptane; 5% cosolvent of 1:1 MeOH:IPA with 0.2% formic acid; 2.5 mL min^{-1} flow-rate; 10 min; UV 230 nm): retention times of 5.97 min (major) and 6.76 min (minor); 96% ee with Rh_2(*S*-PTAD)$_4$.
Determination of d.r.: *N*-Boc deprotection (1.5 equiv of *p*-Toluenesulfonic acid monohydrate in EtOAc [0.1 M] at 60 °C, 4 hours); observed >20:1 d.r.

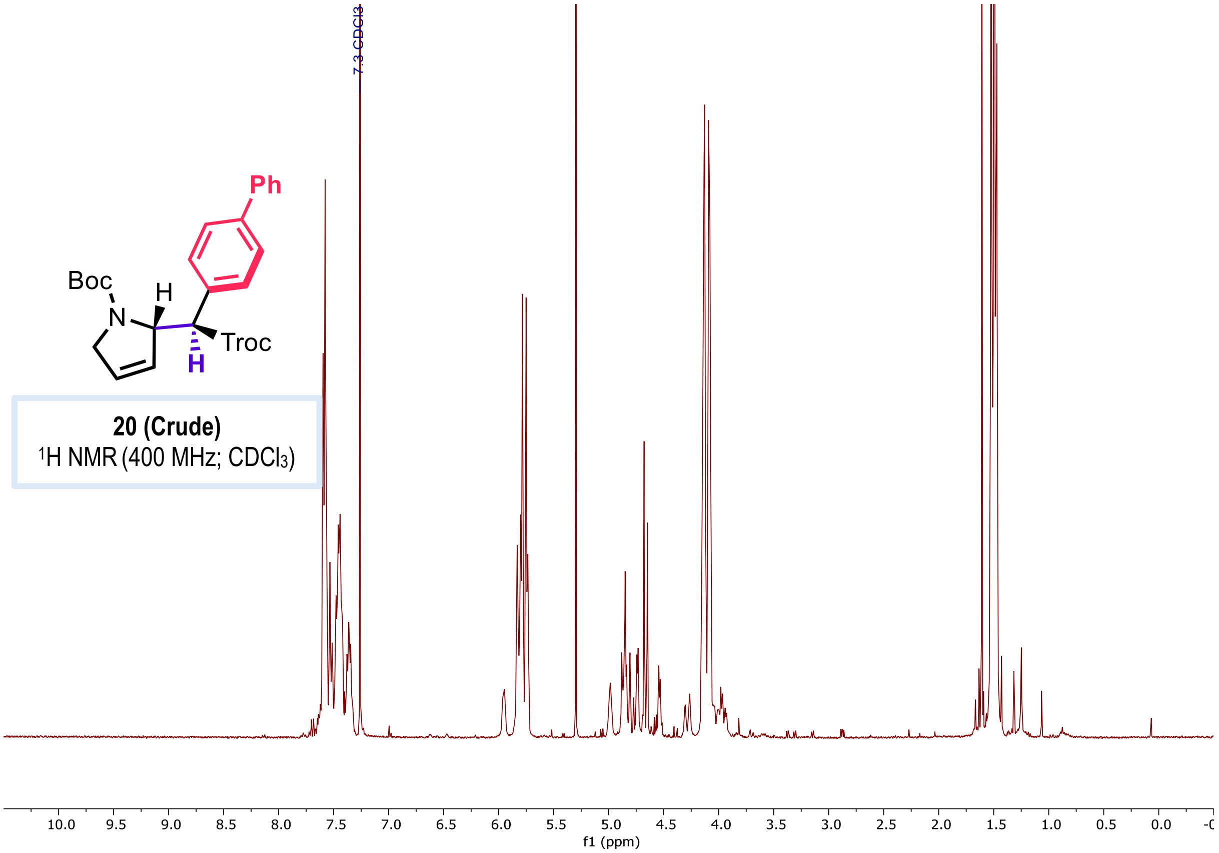
Ph
Boc
N
H
Troc
H
20 (Crude)
1H NMR (400 MHz; CDCl3)
7.3 CDCl3
f1 (ppm)

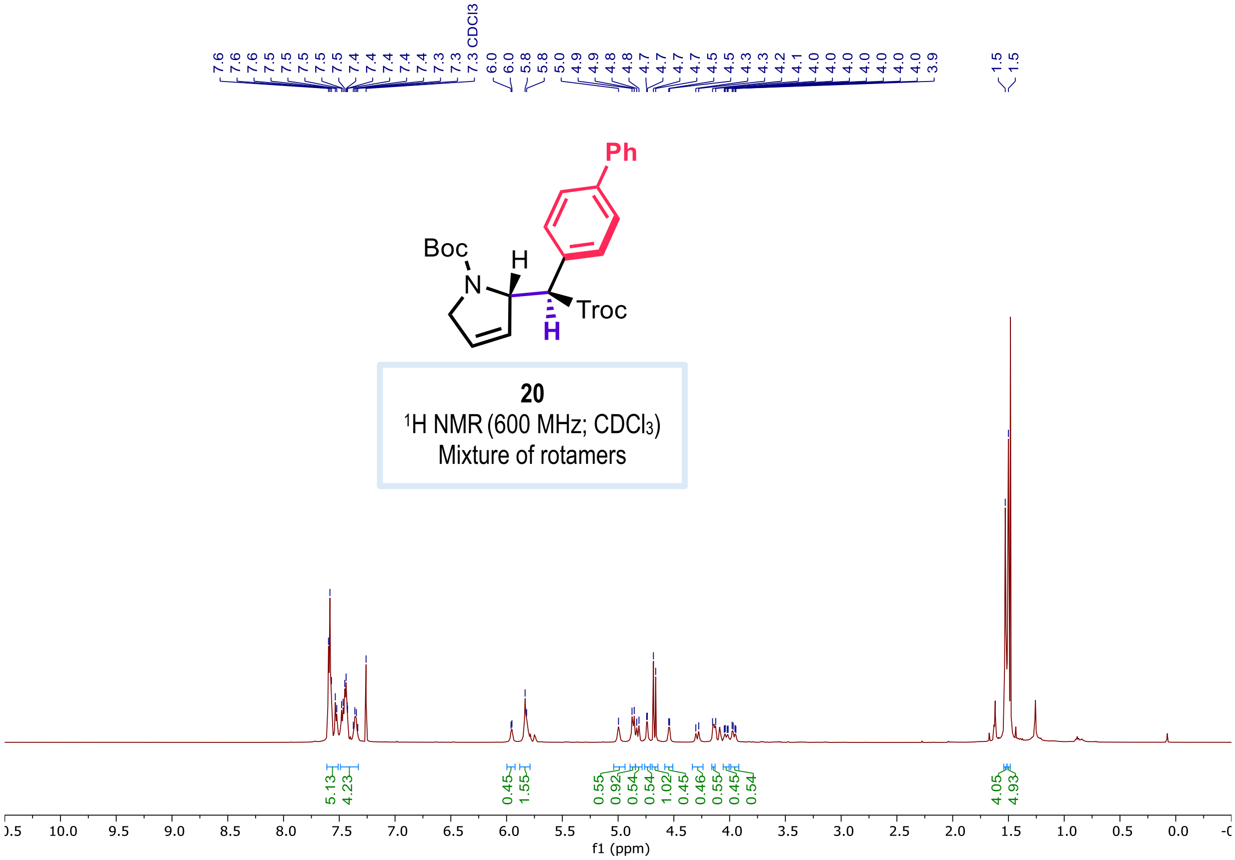
CDCl3
Ph
Boc
N
H
Troc
H
20
1H NMR (600 MHz; CDCl3)
Mixture of rotamers
f1 (ppm)

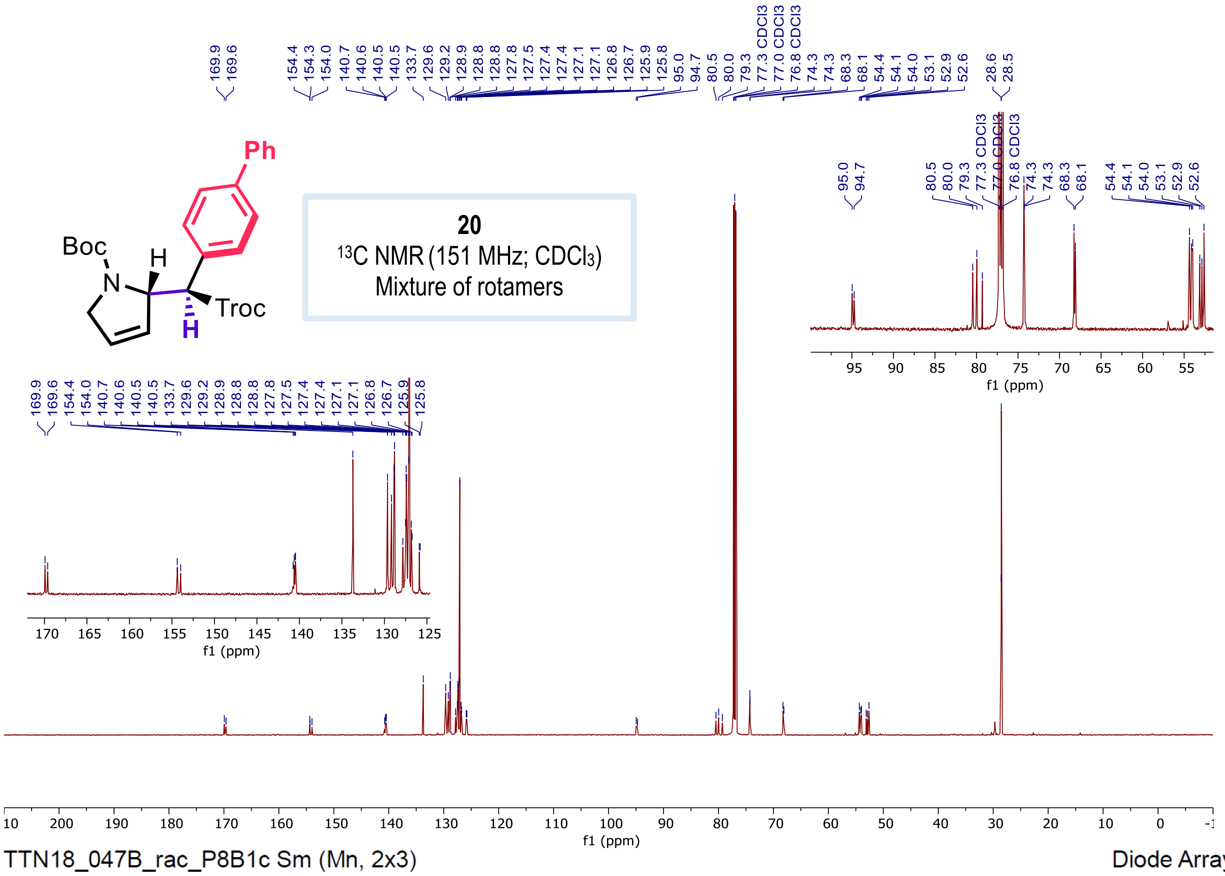

20
13C NMR (151 MHz; CDCl3)
Mixture of rotamers
Boc
Ph
Troc
f1 (ppm)

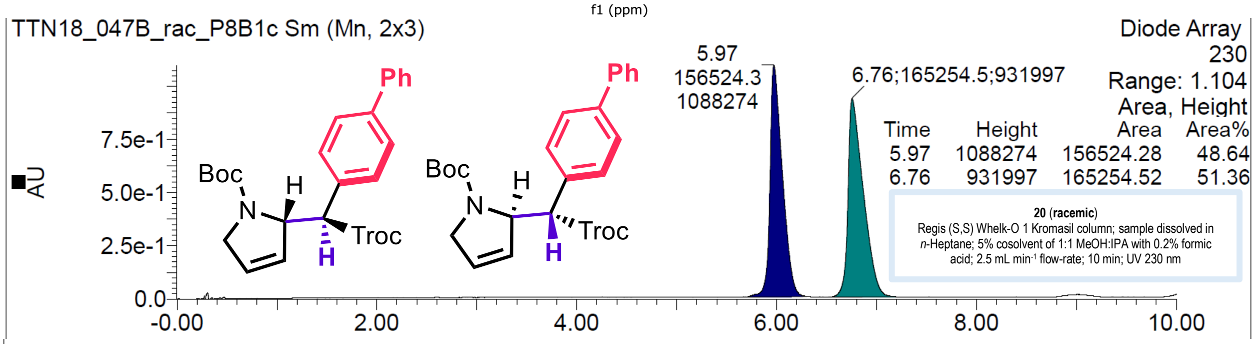

TTN18_047B_rac_P8B1c Sm (Mn, 2x3)
Diode Array
230
Range: 1.104
Area, Height
Time Height Area Area%
5.97 1088274 156524.28 48.64
6.76 931997 165254.52 51.36
5.97
156524.3
1088274
6.76;165254.5;931997
20 (racemic)
Regis (S,S) Whelk-O 1 Kromasil column; sample dissolved in n-Heptane; 5% cosolvent of 1:1 MeOH:IPA with 0.2% formic acid; 2.5 mL min-1 flow-rate; 10 min; UV 230 nm
AU

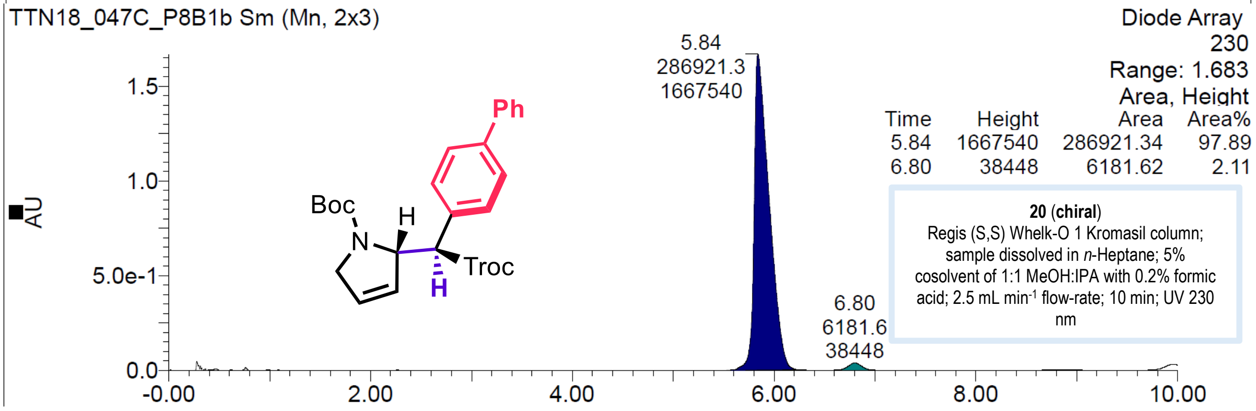

TTN18_047C_P8B1b Sm (Mn, 2x3)
Diode Array
230
Range: 1.683
Area, Height
Time Height Area Area%
5.84 1667540 286921.34 97.89
6.80 38448 6181.62 2.11
5.84
286921.3
1667540
6.80
6181.6
38448
20 (chiral)
Regis (S,S) Whelk-O 1 Kromasil column; sample dissolved in n-Heptane; 5% cosolvent of 1:1 MeOH:IPA with 0.2% formic acid; 2.5 mL min-1 flow-rate; 10 min; UV 230 nm
AU

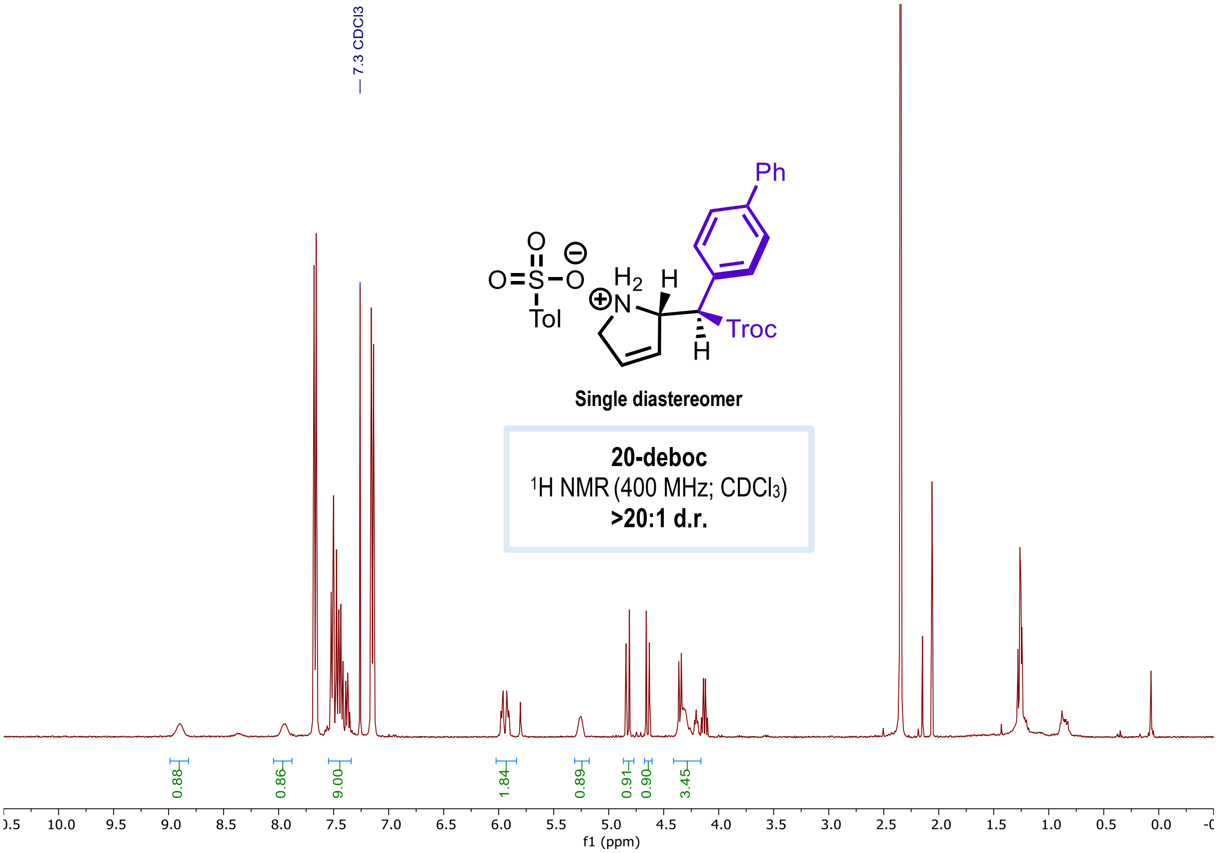

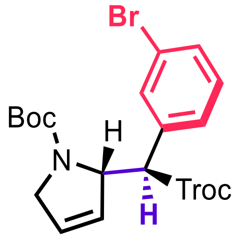

tert-butyl (S)-2-((R)-1-(3-bromophenyl)-2-oxo-2-(2,2,2-trichloroethoxy)ethyl)-2,5-dihydro-1H-pyrrole-1-carboxylate (21)

To an 8 mL vial charged with a stir-bar and 0.5 g of activated 4Å MS was added 2,2,2-trichloroethyl 2-(3-bromophenyl)-2-diazoacetate (186 mg, 1 equiv, 0.500 mmol) and tert-butyl 2,5-dihydro-1H-pyrrole-1-carboxylate (254 mg, 3 equiv, 1.50 mmol). The reaction vial was sealed, backfilled with nitrogen three times, and the vial topped with a nitrogen balloon. To the vial containing the diazo and olefin was added CH_2Cl_2 (1.5 mL). The solution was stirred at room temperature for at least 10 minutes. A stock solution of Rh_2(*S*-PTAD)$_4$ (390 µg, 500 µL CH_2Cl_2, 0.0005 molar, 0.0005 equiv, 0.250 µmol) was added into the reaction solution containing diazo and olefin drop-wise over 15 seconds. The reaction mixture was then allowed to stir or 20 hours at room temperature. After the elapsed time, the reaction mixture was filtered through a celite plug and the filtrate concentrated *in vacuo* at 45 °C. The crude was dry-loaded onto SiO_2 and subjected to SiO_2 flash chromatography pentane/diethyl ether (Biotage Sfär DLV 50 g column as the running-column and Biotage Sfär DLV 50 g column for the dry-load; 100 mL/min flow-rate; 210/230 nm detector; 0% 6CV → 1% 6CV → 3% 6CV → 5% 6CV → 7% 6CV → 10% 6CV → 13% 6CV → 16% 12CV; POI elutes at 10% diethyl ether with trap). The collected fractions were concentrated *in vacuo* at 45 °C to afford a mixture of product and trap. The material was then dissolved in CH_2Cl_2, the solution passed thru a celite plug into a 20 mL vial, and concentrated *in vacuo* at 45 °C. The vial containing the neat material was then subjected to Kugelrohr distillation at 8.0 x 10^{-1} torr vacuum and 95 °C heating bath for at least 20

minutes to distill off the trap. The material was cooled to afford tert-butyl (S)-2-((R)-1-(3-bromophenyl)-2-oxo-2-(2,2,2-trichloroethoxy)ethyl)-2,5-dihydro-1H-pyrrole-1-carboxylate (179 mg, 348 μmol, 69.7% yield, 92% ee, >20:1 d.r.) as a pale-yellow oil.

^{1}H NMR (600 MHz, Chloroform-*d*; as a 0.6:0.4 mixture of rotamers) δ 7.62 (Ar–H brs, 0.6H, major rotamer), 7.57 (Ar–H brs, 0.4H, minor rotamer), 7.45 (Ar–H d, *J* = 8.1 Hz, 0.4H, minor rotamer), 7.42 (Ar–H d, *J* = 8.1 Hz, 0.6H, major rotamer), 7.37 (Ar–H d, *J* = 8.1 Hz, 0.6H, major rotamer), 7.32 (Ar–H d, *J* = 8.1 Hz, 0.4H, minor rotamer), 7.25 – 7.18 (Ar–H m, 1H, mixture of rotamers), 5.99 – 5.89 (=C–H brm, 0.4H, minor rotamer), 5.88 – 5.69 (=C–H brm, 1.6H, mixture of rotamers [0.4H minor rotamer + 1.2H major rotamer = 1.6H]), 5.02 – 4.91 ($C–H_{\alpha\text{-}N,C\text{-}2}$ brm, 0.6H, major rotamer), 4.81 ($C–H_{methylene}$ & $C–H_{\alpha\text{-}N,C\text{-}2}$ d & brm, *J* = 11.9 Hz, 1H, mixture of rotamers [0.4H minor rotamer + 0.6H major rotamer = 1H]), 4.78 ($C–H_{methylene}$ d, *J* = 11.9 Hz, 0.4H, minor rotamer), 4.68 ($C–H_{methylene}$ d, *J* = 11.9 Hz, 1H, mixture of rotamers [0.4H minor rotamer + 0.6H major rotamer = 1H]), 4.57 ($C–H_{benzylic}$ d, *J* = 4.1 Hz, 0.6H, major rotamer), 4.41 ($C–H_{benzylic}$ d, *J* = 4.1 Hz, 0.4H, minor rotamer), 4.27 ($C–H_{\alpha\text{-}N,C\text{-}5}$ d, *J* = 15.4 Hz, 0.4H, minor rotamer), 4.11 ($C–H_{\alpha\text{-}N,C\text{-}5}$ d, *J* = 15.4 Hz, 0.6H, major rotamer), 3.99 ($C–H_{\alpha\text{-}N,C\text{-}5}$ dd, *J* = 15.4, 4.7 Hz, 0.4H, minor rotamer), 3.88 ($C–H_{\alpha\text{-}N,C\text{-}5}$ dd, *J* = 15.4, 4.7 Hz, 0.6H, major rotamer), 1.50 ($–C(CH_3)_3$ s, 3.6H, minor rotamer), 1.48 ($–C(CH_3)_3$ s, 5.4H, major rotamer).
^{13}C NMR (151 MHz, Chloroform-*d*; as a mixture of rotamers) δ 169.4, 169.1, 154.3, 153.9, 136.9, 136.8, 132.3, 131.7, 131.0, 130.8, 130.2, 129.9, 128.2, 128.0, 127.7, 127.5, 126.5, 126.3, 122.8, 122.4, 94.8, 94.6, 80.6, 80.1, 74.4, 74.3, 68.0, 67.9, 54.3, 54.1, 54.0, 52.7, 28.5.
HRMS (APCI -) calc. mass for $C_{19}H_{20}O_4N^{79}Br^{35}Cl_3$ $[M - H]^-$ 509.9647; obs. mass for $C_{19}H_{20}O_4N^{79}Br^{35}Cl_3$ $[M - H]^-$ 509.9632.
$[\alpha]^{20}_D$: -70.4° (c = 0.370 g/100 mL, EtOAc).
HPLC (Regis (S,S) Whelk-O 1 5/100 Kromasil column; sample dissolved in 20:80 IPA:*n*-Heptane; 2% IPA in *n*-Hexane; 1 mL min^{-1} flow-rate; 30 min; UV 230 nm): retention times of 10.81 min (major) and 12.60 min (minor); 92% ee with Rh_2(*S*-PTAD)$_4$.
Determination of d.r.: *N*-Boc deprotection (1.5 equiv of *p*-Toluenesulfonic acid monohydrate in EtOAc [0.1 M] at 60 °C, 4 hours); observed >20:1 d.r.

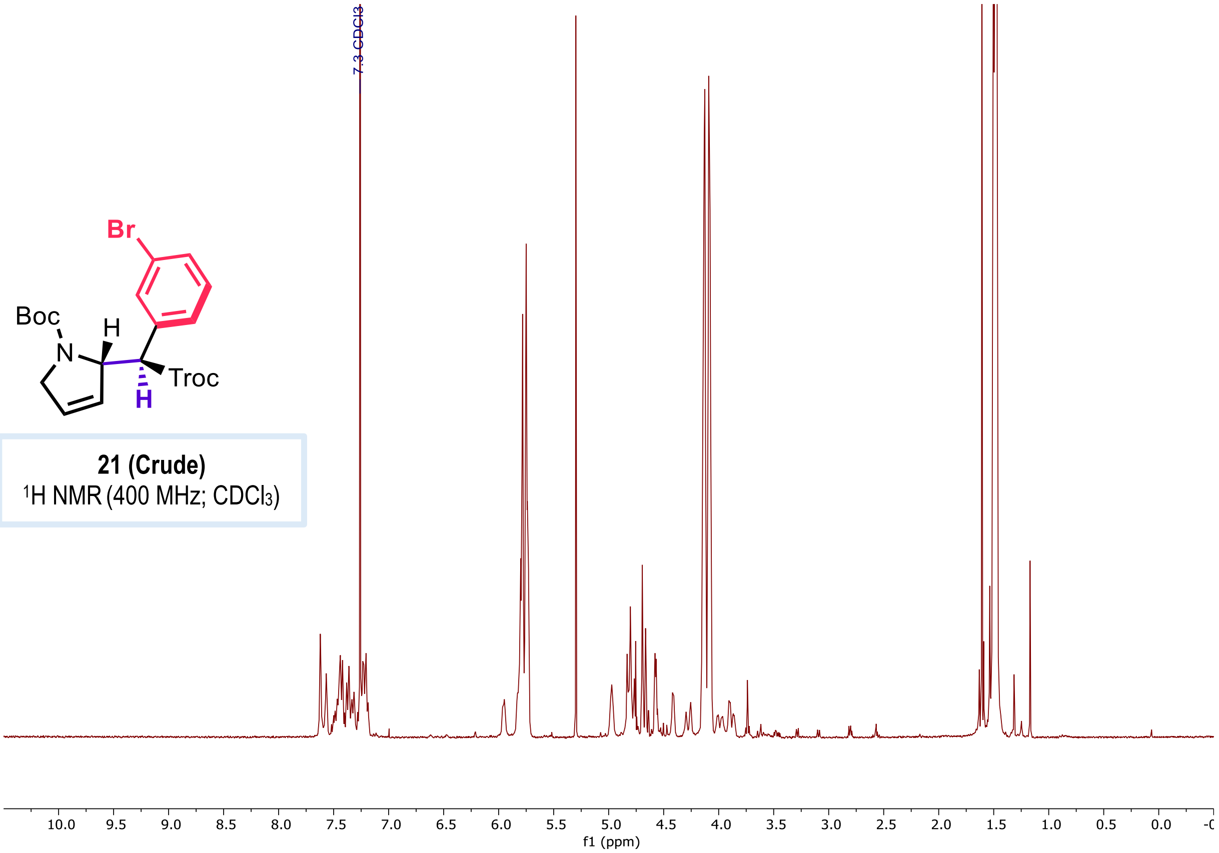

21 (Crude)
^{1}H NMR (400 MHz; $CDCl_3$)

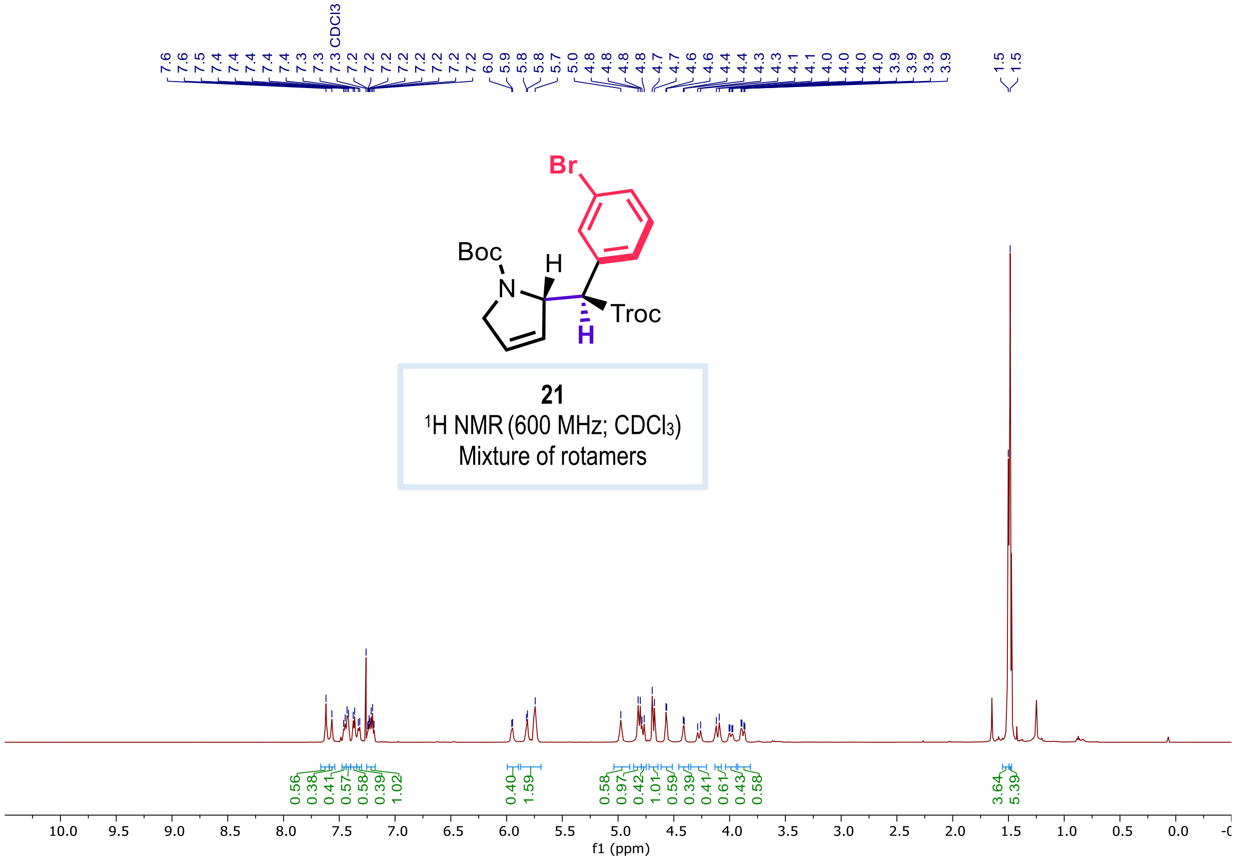

21
^{1}H NMR (600 MHz; $CDCl_3$)
Mixture of rotamers

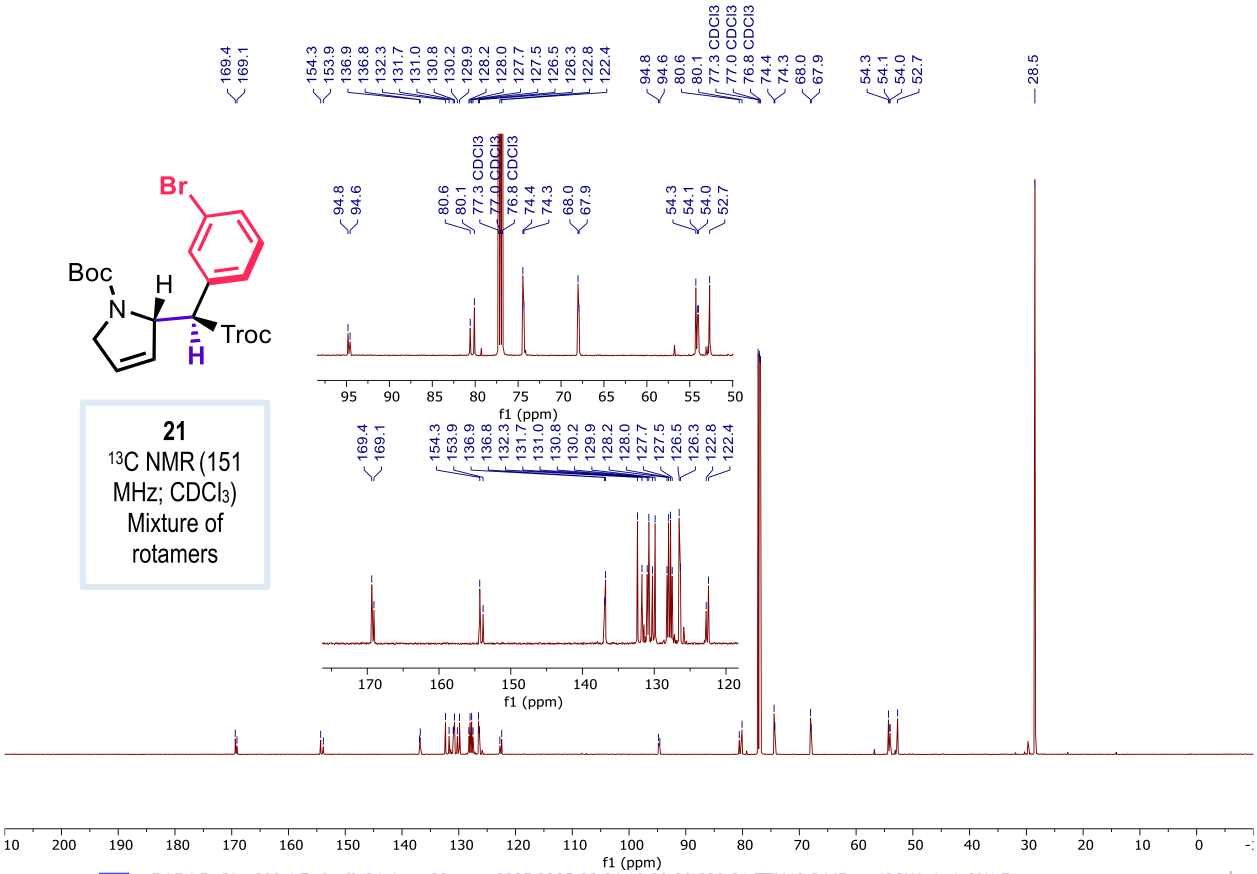

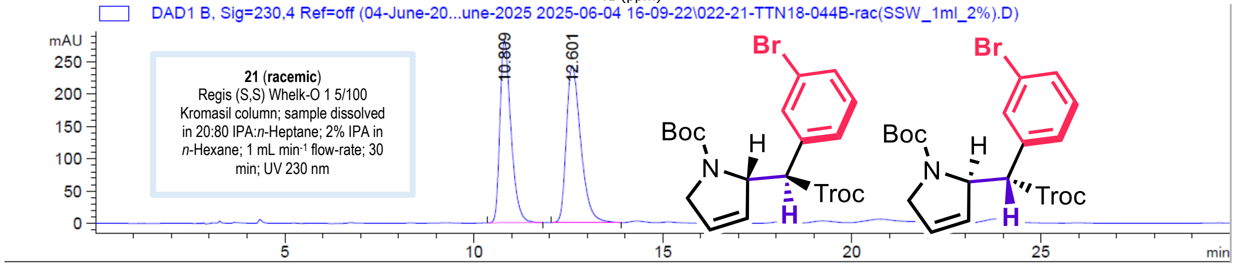


```
Signal 2: DAD1 B, Sig=230,4 Ref=off

Peak RetTime Type  Width     Area      Height     Area
  #   [min]        [min]   [mAU*s]     [mAU]        %
----|-------|----|-------|----------|----------|--------|
   1  10.809 BV R  0.3030 5710.91064  281.69656  47.0804
   2  12.601 BB    0.3727 6419.20410  242.57915  52.9196

Totals :                  1.21301e4   524.27571
```

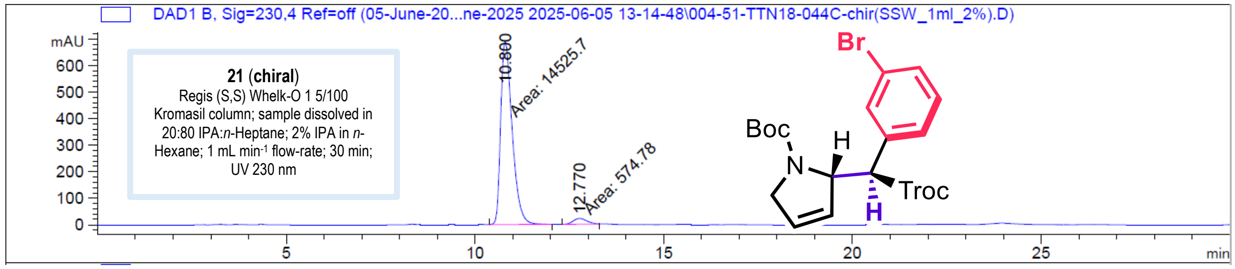

Signal 2: DAD1 B, Sig=230,4 Ref=off

Peak #	RetTime [min]	Type	Width [min]	Area [mAU*s]	Height [mAU]	Area %
1	10.800	MM	0.3516	1.45257e4	688.62500	96.1936
2	12.770	MM	0.4309	574.78033	22.23139	3.8064
Totals :				1.51005e4	710.85639	

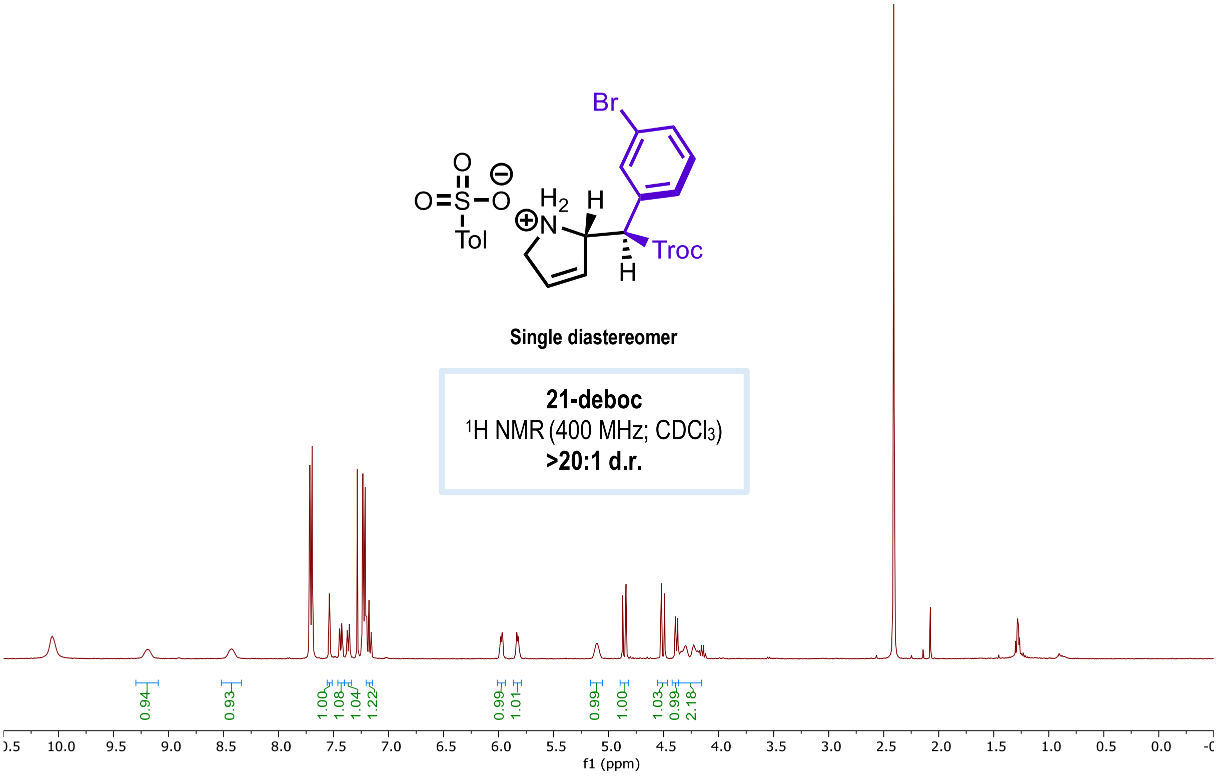

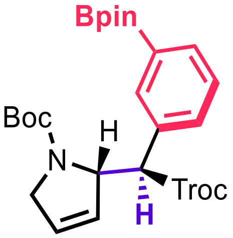

tert-butyl (S)-2-((R)-2-oxo-1-(3-(4,4,5,5-tetramethyl-1,3,2-dioxaborolan-2-yl)phenyl)-2-(2,2,2-trichloroethoxy)ethyl)-2,5-dihydro-1H-pyrrole-1-carboxylate (22)

To an 8 mL vial charged with a stir-bar and 0.5 g of activated 4Å MS was added 2,2,2-trichloroethyl 2-diazo-2-(3-(4,4,5,5-tetramethyl-1,3,2-dioxaborolan-2-yl)phenyl)acetate (210 mg, 1 equiv, 0.500 mmol) and tert-butyl 2,5-dihydro-1H-pyrrole-1-carboxylate (254 mg, 3 equiv, 1.50 mmol). The reaction vial was sealed, backfilled with nitrogen three times, and the vial topped with a nitrogen balloon. To the vial containing the diazo and olefin was added CH_2Cl_2 (1.5 mL). The solution was stirred at room temperature for at least 10 minutes. A stock solution of Rh_2(*S*-PTAD)$_4$ (390 µg, 500 µL CH_2Cl_2, 0.0005 molar, 0.0005 equiv, 0.250 µmol) was added into the reaction solution containing diazo and olefin drop-wise over 15 seconds. The reaction mixture was then allowed to stir for 20 hours at room temperature. After the elapsed

time, the reaction mixture was filtered through a celite plug and the filtrate concentrated *in vacuo* at 45 °C. The crude was dry-loaded onto SiO_2 and subjected to SiO_2 flash chromatography using pentane/diethyl ether (Biotage Sfär DLV 50 g column as the running-column and Biotage Sfär DLV 50 g column for the dry-load; 100 mL/min flow-rate; 210/230 nm detector; 0% 6CV → 1% 6CV → 3% 6CV → 5% 6CV → 7% 6CV → 10% 6CV → 13% 6CV → 16% 12CV; POI elutes at 13-16% ether). The collected fractions were concentrated *in vacuo* at 45 °C to afford tert-butyl (S)-2-((R)-2-oxo-1-(3-(4,4,5,5-tetramethyl-1,3,2-dioxaborolan-2-yl)phenyl)-2-(2,2,2-trichloroethoxy)ethyl)-2,5-dihydro-1H-pyrrole-1-carboxylate (121 mg, 216 µmol, 43.2% yield, 97% ee, >20:1 d.r.) as a foamy white amorphous material.

^{1}H NMR (600 MHz, Chloroform-*d*; as a 0.55:0.45 mixture of rotamers) δ 7.85 (Ar–H brs, 0.55H, major rotamer), 7.80 (Ar–H brs, 0.45H, minor rotamer), 7.74 (Ar–H dd, *J* = 8.0, 8.0 Hz, 1H, mixture of rotamers), 7.56 (Ar–H d, *J* = 8.0 Hz, 0.55H, major rotamer), 7.51 (Ar–H d, *J* = 8.0 Hz, 0.45H, minor rotamer), 7.40 – 7.31 (Ar–H m, 1H, mixture of rotamers), 5.99 – 5.88 (=C–H brm, 0.45H, minor rotamer), 5.88 – 5.68 (=C–H brm, 1.55H, mixture of rotamers [0.45H minor rotamer + 1.1H major rotamer = 1.55H]), 5.00 – 4.93 (C–$H_{\alpha\text{-}N,C\text{-}2}$ brm, 0.55H, major rotamer), 4.84 (C–$H_{methylene}$ & C–$H_{\alpha\text{-}N,C\text{-}2}$ d & brm, *J* = 12.0 Hz, 1H, mixture of rotamers [0.45H minor rotamer + 0.55H major rotamer = 1H]), 4.77 (C–$H_{methylene}$ d, *J* = 12.0 Hz, 0.45H, minor rotamer), 4.71 (C–$H_{benzylic}$ d, *J* = 4.3 Hz, 0.55H, major rotamer), 4.67 (C–$H_{methylene}$ d, *J* = 12.0 Hz, 0.45H, minor rotamer), 4.64 (C–$H_{methylene}$ d, *J* = 12.0 Hz, 0.55H, major rotamer), 4.50 (C–$H_{benzylic}$ d, *J* = 4.3 Hz, 0.45H, minor rotamer), 4.27 (C–$H_{\alpha\text{-}N,C\text{-}5}$ d, *J* = 15.1 Hz, 0.45H, minor rotamer), 4.11 (C–$H_{\alpha\text{-}N,C\text{-}5}$ d, *J* = 15.1 Hz, 0.55H, major rotamer), 4.02 (C–$H_{\alpha\text{-}N,C\text{-}5}$ dd, *J* = 15.1, 4.6 Hz, 0.45H, minor rotamer), 3.93 (C–$H_{\alpha\text{-}N,C\text{-}5}$ dd, *J* = 15.1, 4.6 Hz, 0.55H, major rotamer), 1.51 (–$C(CH_3)_3$ s, 4.05H, minor rotamer), 1.49 (–$C(CH_3)_3$ s, 4.95H, major rotamer), 1.33 (–CH_3 s, 12H, mixture of rotamers).
^{13}C NMR (151 MHz, Chloroform-*d*; as a mixture of rotamers) δ 169.9, 169.6, 154.3, 154.0, 135.9, 135.2, 134.2, 134.1, 134.0, 133.9, 131.8, 131.4, 128.1, 127.8, 127.7, 127.2, 126.9, 126.8, 95.0, 94.7, 83.9, 83.8, 80.4, 79.9, 74.2, 68.1, 68.0, 54.4, 54.1, 52.8, 28.5, 24.9.
HRMS (APCI -) calc. mass for $C_{25}H_{32}O_6N^{11}B^{35}Cl_3$ [M - H]$^-$ 558.1394; obs. mass for $C_{25}H_{32}O_6N^{11}B^{35}Cl_3$ [M - H]$^-$ 558.1382.
$[\alpha]^{20}_D$: -90.7° (c = 0.215 g/100 mL, EtOAc).
SFC (Regis (S,S) Whelk-O 1 Kromasil column; sample dissolved in *n*-Heptane; 2% cosolvent of 1:1 MeOH:IPA with 0.2% formic acid; 2.5 mL min^{-1} flow-rate; 15 min; UV 210 nm): retention times of 7.87 min (major) and 8.53 min (minor); 97% ee with Rh_2(*S*-PTAD)$_4$.
Determination of d.r.: *N*-Boc deprotection (1.5 equiv of *p*-Toluenesulfonic acid monohydrate in EtOAc [0.1 M] at 60 °C, 4 hours); observed >20:1 d.r.

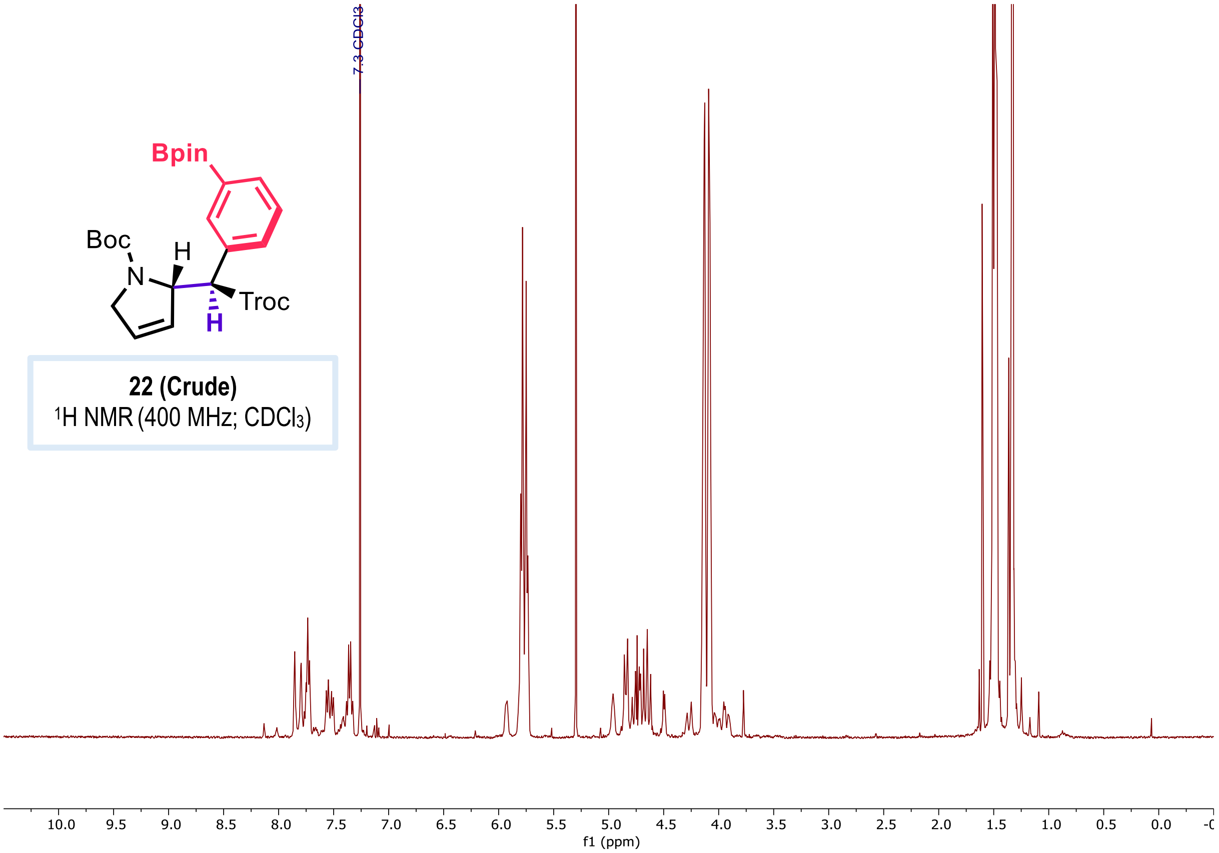
Bpin
Boc
N
H
Troc
H
22 (Crude)
¹H NMR (400 MHz; CDCl3)
7.3 CDCl3
f1 (ppm)

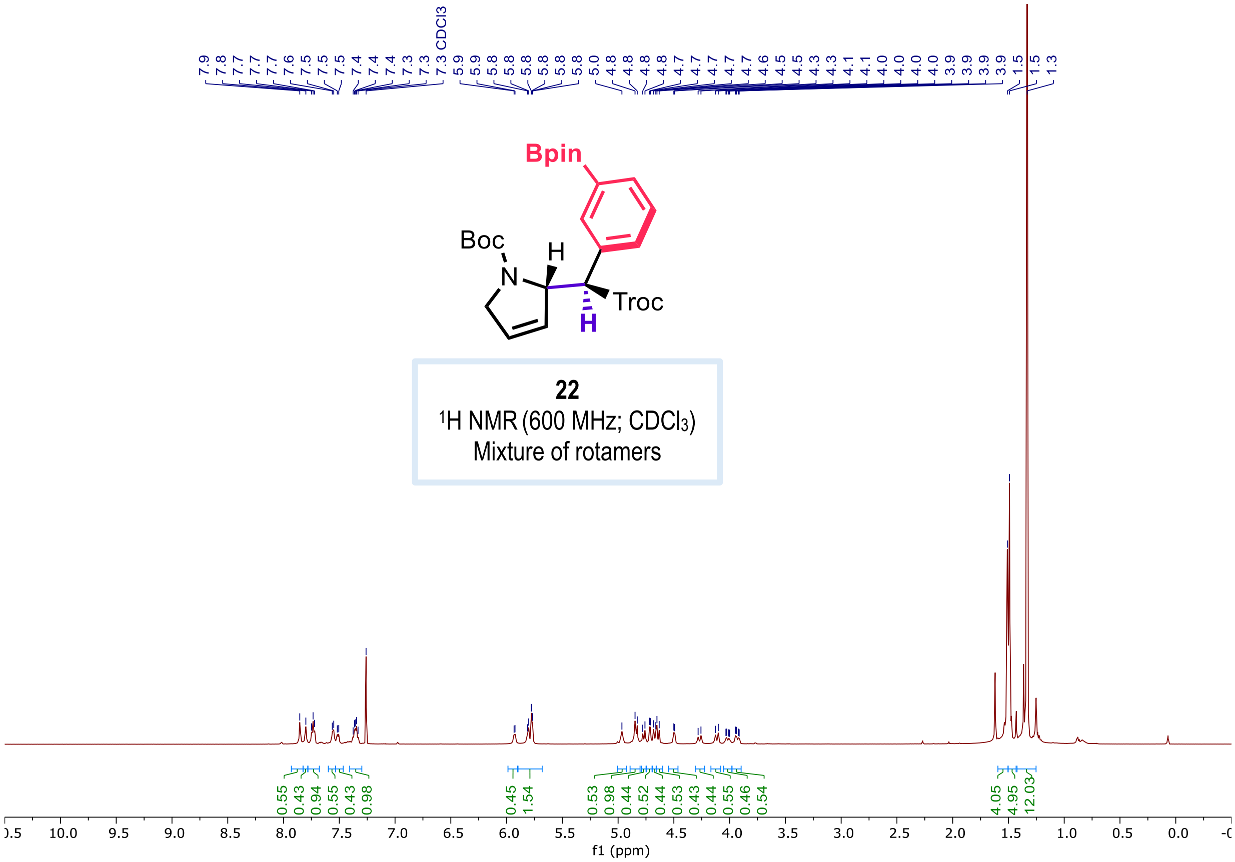
Bpin
Boc
N
H
Troc
H
22
¹H NMR (600 MHz; CDCl3)
Mixture of rotamers
7.3 CDCl3
f1 (ppm)

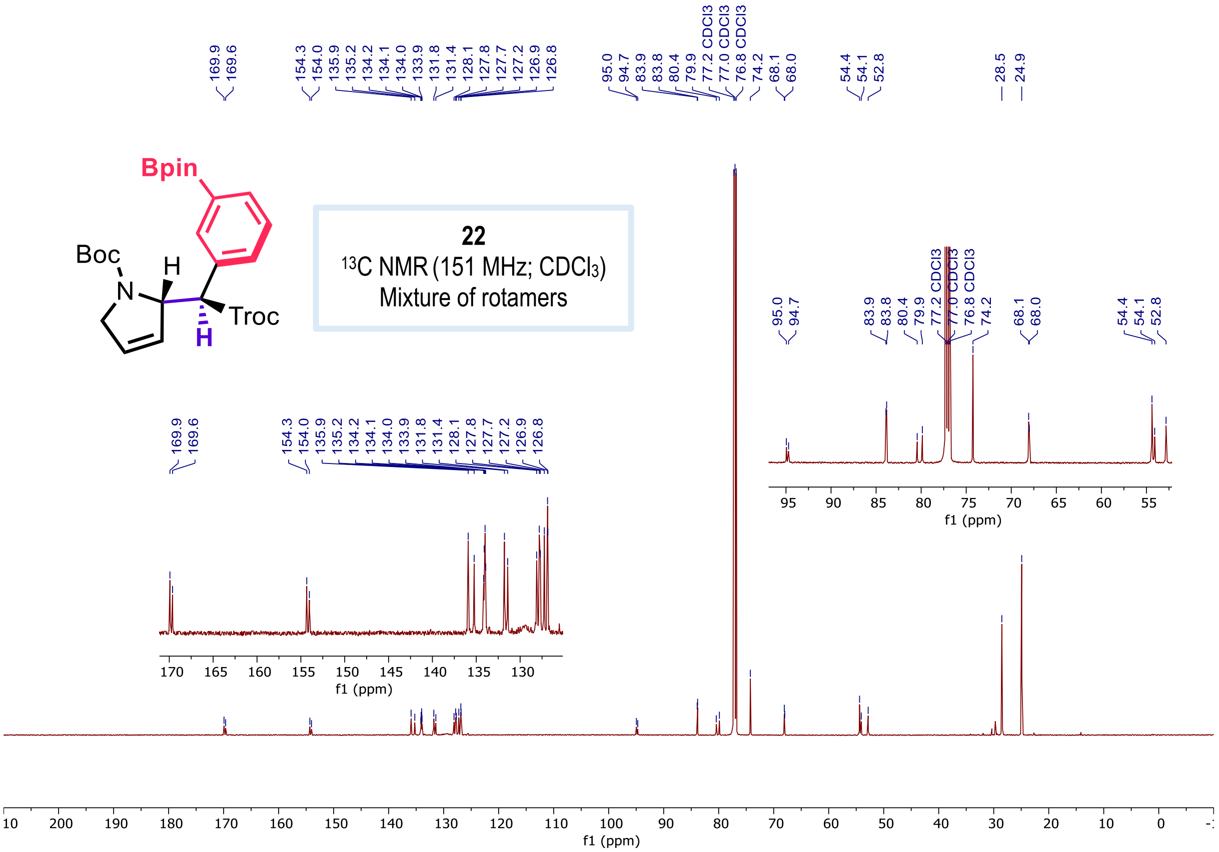
Bpin
Boc
N
H
Troc
H
22
^{13}C NMR (151 MHz; CDCl$_3$)
Mixture of rotamers
169.9 169.6 154.3 154.0 135.9 135.2 134.2 134.1 134.0 133.9 131.8 131.4 128.1 127.8 127.7 127.2 126.9 126.8
95.0 94.7 83.9 83.8 80.4 79.9 77.2 CDCl3 77.0 CDCl3 76.8 CDCl3 74.2 68.1 68.0 54.4 54.1 52.8 28.5 24.9
f1 (ppm)

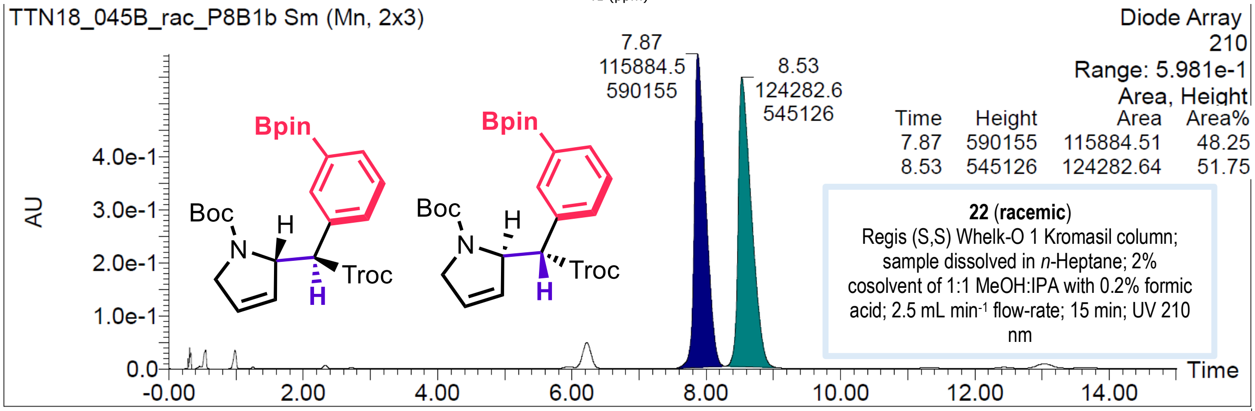
TTN18_045B_rac_P8B1b Sm (Mn, 2x3)
Diode Array
210
Range: 5.981e-1
Area, Height
Time Height Area Area%
7.87 590155 115884.51 48.25
8.53 545126 124282.64 51.75
7.87
115884.5
590155
8.53
124282.6
545126
22 (racemic)
Regis (S,S) Whelk-O 1 Kromasil column; sample dissolved in n-Heptane; 2% cosolvent of 1:1 MeOH:IPA with 0.2% formic acid; 2.5 mL min^{-1} flow-rate; 15 min; UV 210 nm
Bpin
Boc
N
H
Troc
H
AU
Time

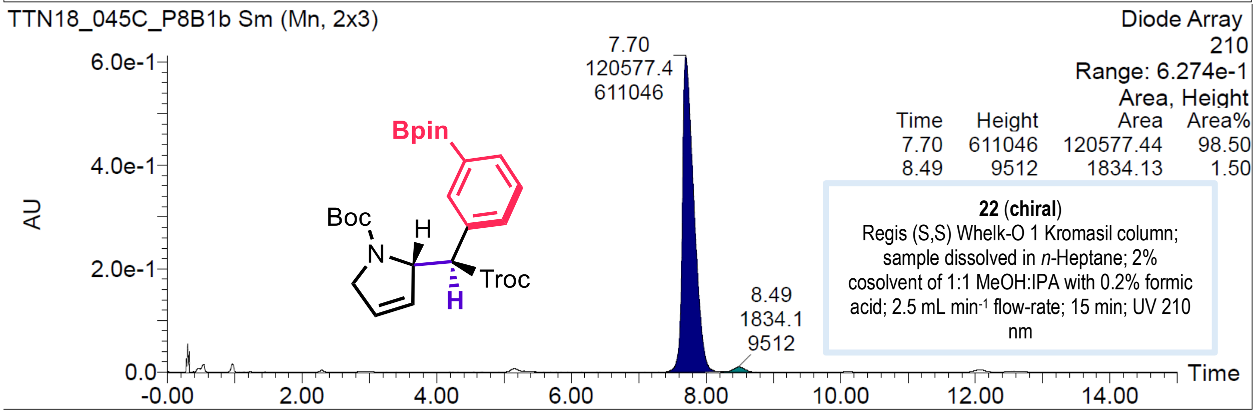
TTN18_045C_P8B1b Sm (Mn, 2x3)
Diode Array
210
Range: 6.274e-1
Area, Height
Time Height Area Area%
7.70 611046 120577.44 98.50
8.49 9512 1834.13 1.50
7.70
120577.4
611046
8.49
1834.1
9512
22 (chiral)
Regis (S,S) Whelk-O 1 Kromasil column; sample dissolved in n-Heptane; 2% cosolvent of 1:1 MeOH:IPA with 0.2% formic acid; 2.5 mL min^{-1} flow-rate; 15 min; UV 210 nm
Bpin
Boc
N
H
Troc
H
AU
Time

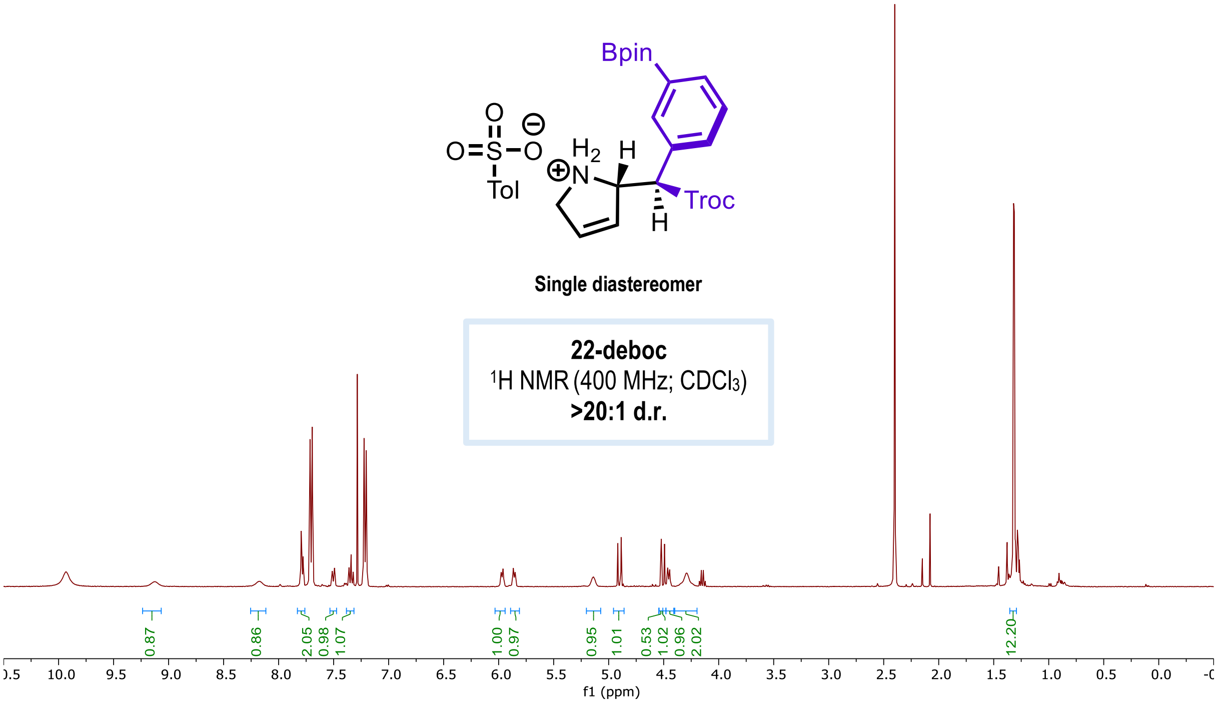

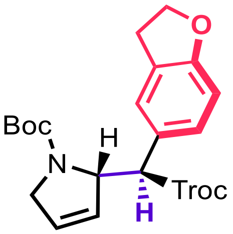

tert-butyl (S)-2-((R)-1-(2,3-dihydrobenzofuran-5-yl)-2-oxo-2-(2,2,2-trichloroethoxy)ethyl)-2,5-dihydro-1H-pyrrole-1-carboxylate (23)

To an 8 mL dram vial charged with a stir-bar and 0.5 g of activated 4Å MS was added tert-butyl 2,5-dihydro-1H-pyrrole-1-carboxylate (254 mg, 3 equiv, 1.50 mmol). The mixture was backfilled with nitrogen three times. Subsequently, a stock solution of Rh_2(*S*-PTAD)$_4$ (390 µg, 1.00 mL CH_2Cl_2, 0.00025 molar, 0.0005 equiv, 0.250 µmol) was added to the mixture. The reaction vessel containing olefin and catalyst was stirred at room temperature and for at least 10 minutes. After the elapsed time, to the reaction vessel was added 2,2,2-trichloroethyl 2-diazo-2-(2,3-dihydrobenzofuran-5-yl)acetate (168 mg, 1.00 mL CH_2Cl_2, 0.5 molar, 1 equiv, 0.500 mmol) via a multi-channel syringe pump [*syringe pump settings - 1 mL syringe, 0.30 mL/hr, diameter 4.71 mm, vol 1.0 mL; Air-Tite or SilverPoint 22Gx4" long hypodermic needle was used*] over 3 hours. After complete addition and an elapsed time of 3 hours, the resulting solution was reacted for an additional 17 hours and then subsequently, filtered over a celite plug, and then the filtrate concentrated *in vacuo* at 40 °C. The crude material was dry-loaded onto SiO_2 and subjected to SiO_2 flash chromatography using hexanes/diethyl ether (Biotage Sfär DLV 50 g column as the running-column and Biotage Sfär DLV 50 g column for the dry-load; 100 mL/min flow-rate; 210/230 nm detector; 0% 6CV → 1% 6CV → 5% 6CV → 10% 6CV → 15% 6CV → 20% 6CV → 40% 6CV; SM elutes at 10% ether and then POI elutes at 15% ether). The collected fractions were concentrated *in vacuo* at 40 °C to afford tert-

butyl (S)-2-((R)-1-(2,3-dihydrobenzofuran-5-yl)-2-oxo-2-(2,2,2-trichloroethoxy)ethyl)-2,5-dihydro-1H-pyrrole-1-carboxylate (190 mg, 399 μmol, 79.7% yield, 92% ee, >20:1 d.r.) as a yellow oil.

^{1}H NMR (400 MHz, Chloroform-*d*; as a 0.53:0.47 mixture of rotamers) δ 7.29 (Ar–H brs, 0.53H, major rotamer), 7.24 (Ar–H brs, 0.47H, minor rotamer), 7.18 (Ar–H d, *J* = 7.9 Hz, 0.53H, major rotamer), 7.09 (Ar–H d, *J* = 7.9 Hz, 0.47H, minor rotamer), 6.75 (Ar–H dd, *J* = 7.9, 7.9 Hz, 1H, mixture of rotamers), 5.95 – 5.87 (=C–H brm, 0.47H, minor rotamer), 5.87 – 5.73 (=C–H brm, 1.53H, mixture of rotamers [0.47H minor rotamer + 1.06H major rotamer = 1.53H]), 4.91 – 4.70 (C–$H_{\alpha\text{-}N,C\text{-}2}$ & C–$H_{methylene}$ brm's & d's, 2H, mixture of rotamers [0.94H minor rotamer + 1.06H major rotamer = 2H]), 4.66 – 4.53 (2 C–$H_{methylene}$ & C–$H_{benzylic}$ m, 3.53H, mixture of rotamers [1.41H minor rotamer + 2.12H major rotamer = 3.53H]), 4.44 (C–$H_{benzylic}$ d, *J* = 5.3 Hz, 0.47H, minor rotamer), 4.26 (C–$H_{\alpha\text{-}N,C\text{-}5}$ d, *J* = 15.7 Hz, 0.47H, minor rotamer), 4.12 (C–$H_{\alpha\text{-}N,C\text{-}5}$ d, *J* = 15.7 Hz, 0.53H, major rotamer), 4.04 – 3.90 (C–$H_{\alpha\text{-}N,C\text{-}5}$ m, 1H, mixture of rotamers [0.47H minor rotamer + 0.53H major rotamer = 1H]), 3.27 – 3.15 (C–$H_{methylene}$ m, 2H, mixture of rotamers [0.94H minor rotamer + 1.06H major rotamer = 2H]), 1.52 (–$C(CH_3)_3$ s, 4.23H, minor rotamer), 1.48 (–$C(CH_3)_3$ s, 4.77H, major rotamer).

^{13}C NMR (151 MHz, Chloroform-*d*; as a mixture of rotamers) δ 170.3, 170.0, 159.8, 159.6, 154.4, 154.0, 128.9, 128.6, 127.6, 127.5, 127.3, 127.1, 126.9, 126.8, 126.6, 126.5, 125.8, 125.2, 109.3, 109.1, 95.0, 94.8, 80.3, 79.9, 74.2, 74.1, 71.4, 71.4, 68.5, 68.3, 54.3, 54.1, 53.5, 52.2, 29.7, 29.7, 28.5.

HRMS (ESI +) calc. mass for $C_{21}H_{25}O_5N^{35}Cl_3$ [M + H]$^+$ 476.0793; obs. mass for $C_{21}H_{25}O_5N^{35}Cl_3$ [M + H]$^+$ 476.0798.

[α]$^{20}_D$: -91.8° (c = 0.450 g/100 mL, EtOAc).

HPLC (Chiralcel OD-H column; sample dissolved in 20:80 IPA:*n*-Heptane; 2% IPA in *n*-Hexane; 1 mL min^{-1} flow-rate; 30 min; UV 230 nm): retention times of 7.18 min (minor) and 10.83 min (major); 92% ee with Rh_2(*S*-PTAD)$_4$.

Determination of d.r.: *N*-Boc deprotection (10 equiv of TFA in CH_2Cl_2 [0.25 M] at 25 °C, 24 hours); observed >20:1 d.r.

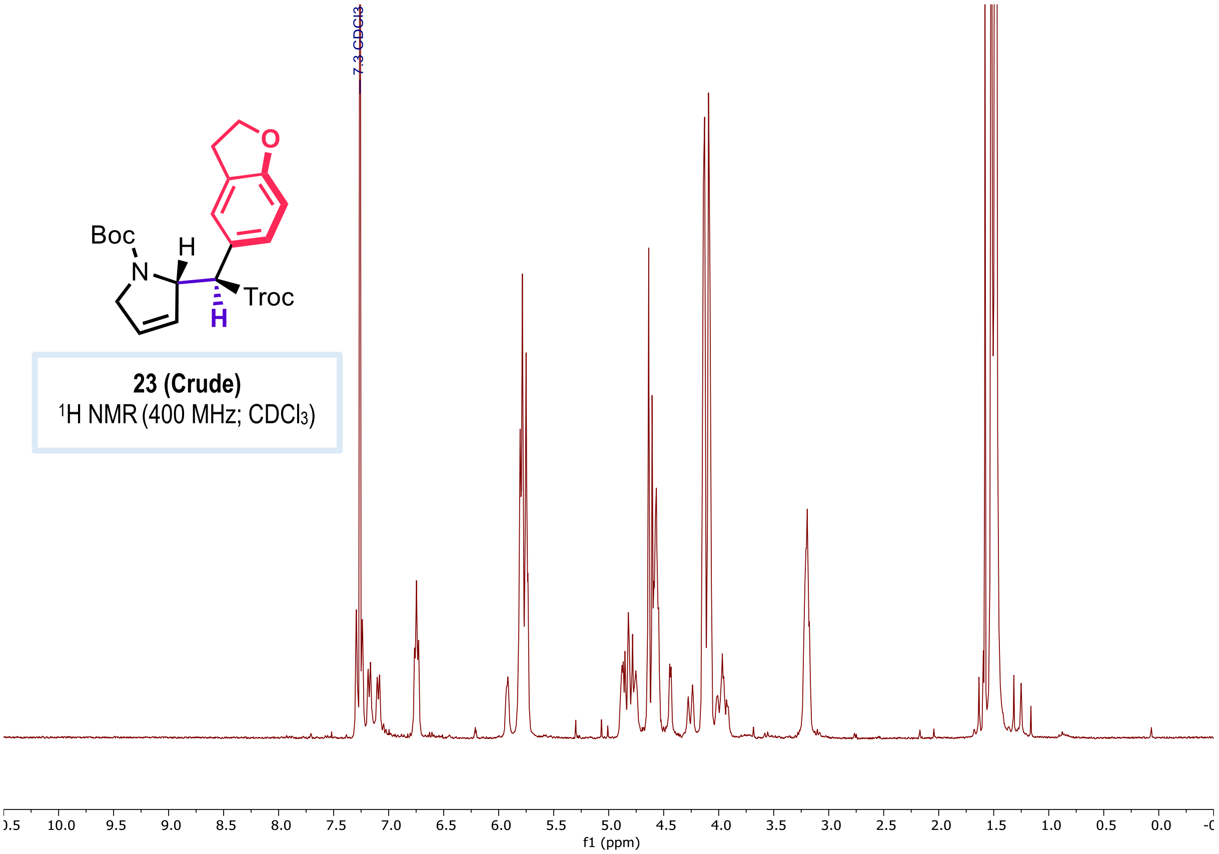

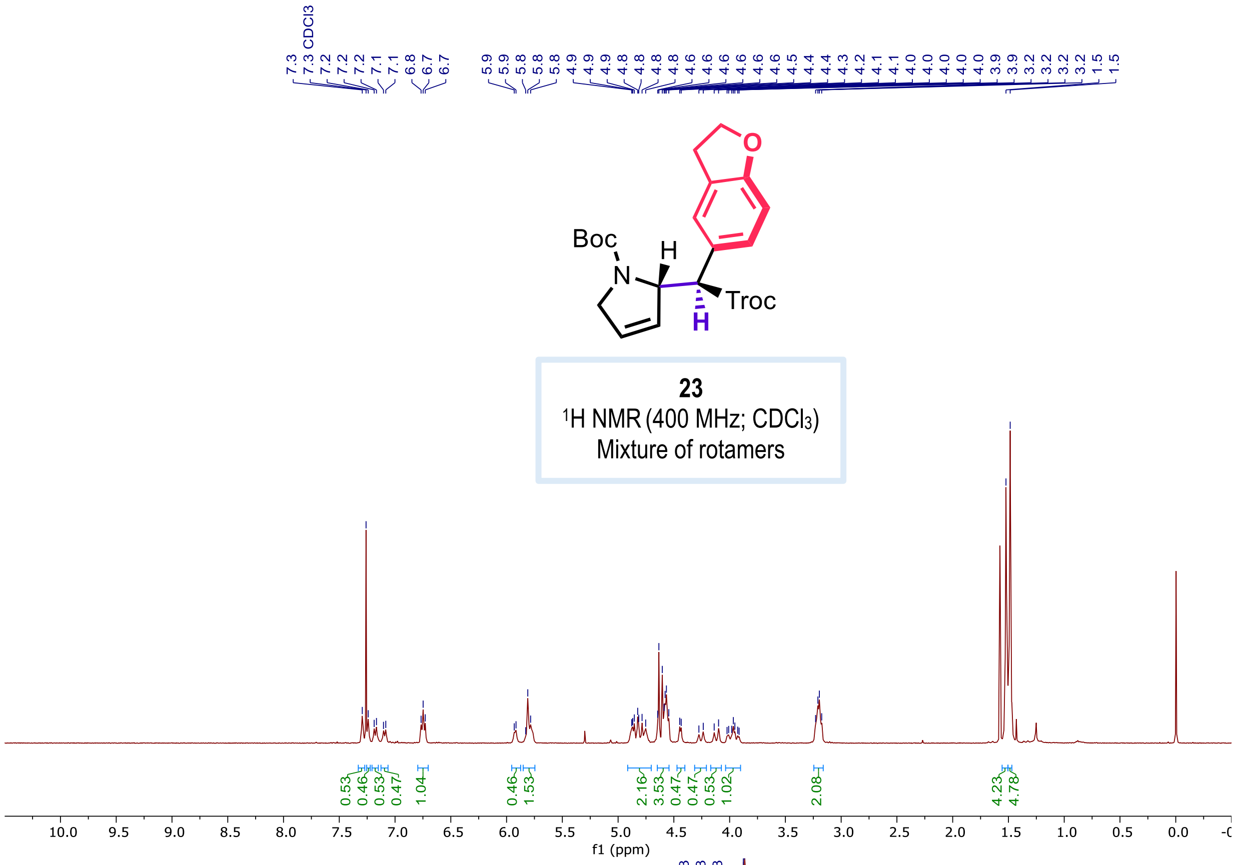
23
1H NMR (400 MHz; CDCl3)
Mixture of rotamers

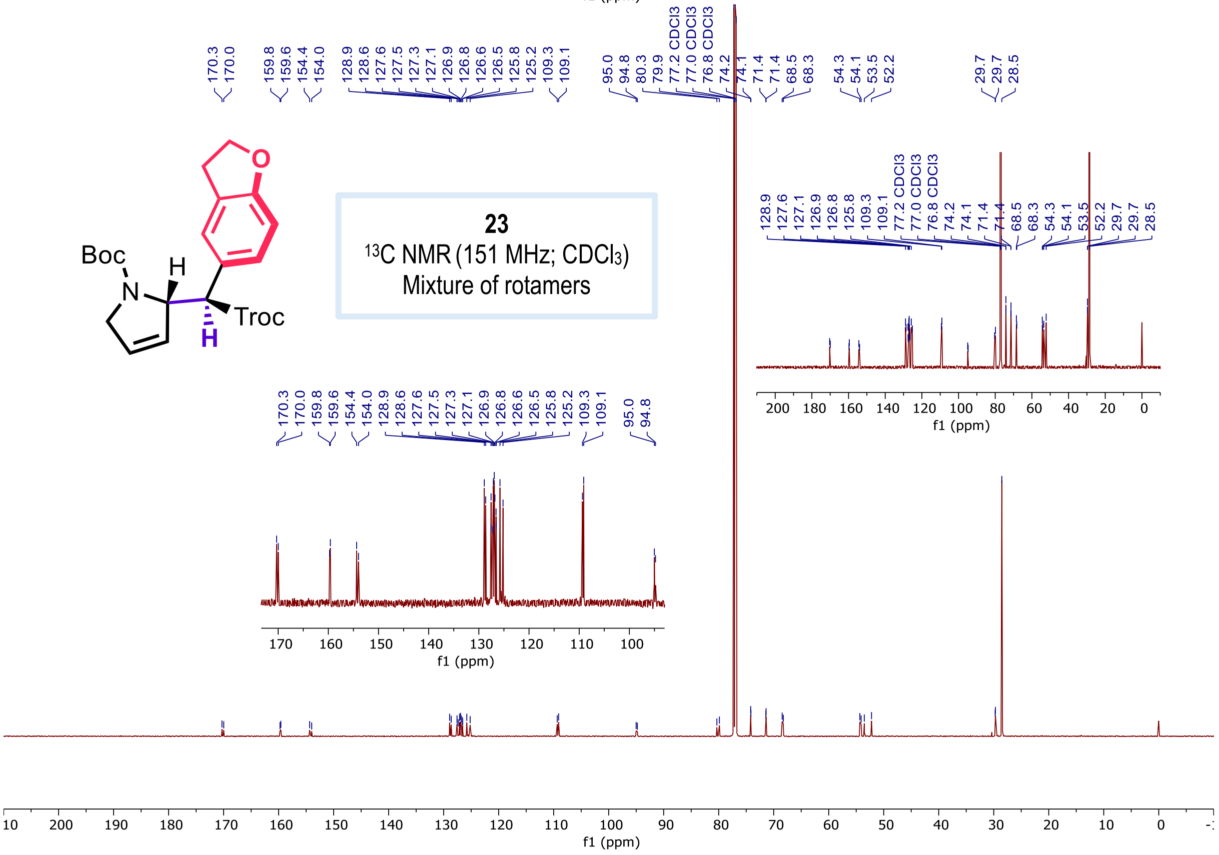
23
13C NMR (151 MHz; CDCl3)
Mixture of rotamers

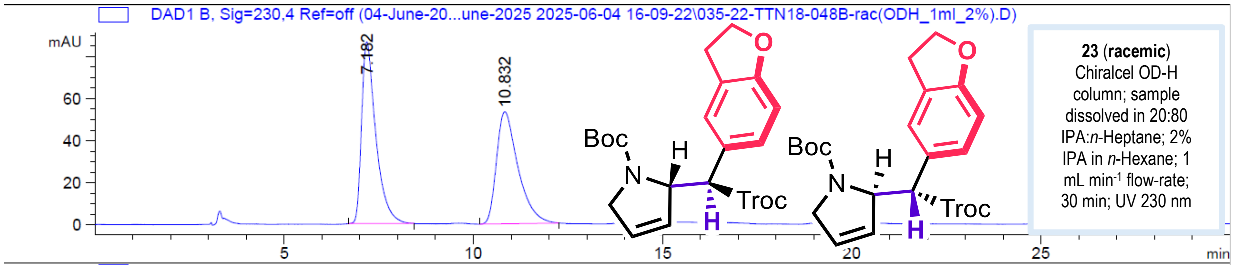

Signal 2: DAD1 B, Sig=230,4 Ref=off

Peak #	RetTime [min]	Type	Width [min]	Area [mAU*s]	Height [mAU]	Area %
1	7.182	BB	0.3573	2260.35010	86.37801	51.7025
2	10.832	VB R	0.4649	2111.49072	53.38577	48.2975

Totals : 4371.84082 139.76377

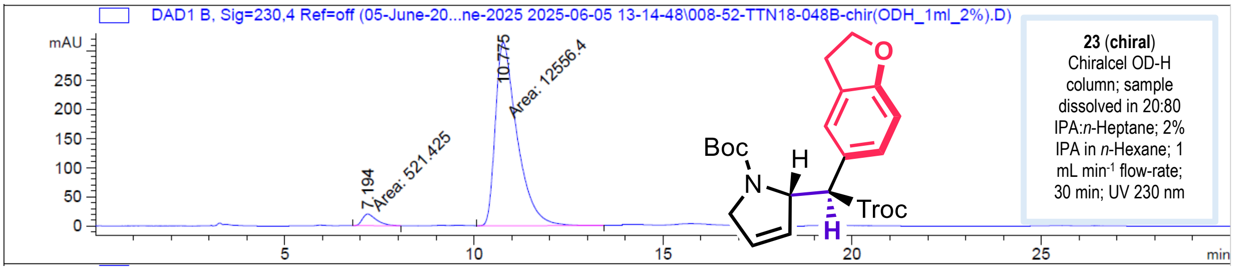

Signal 2: DAD1 B, Sig=230,4 Ref=off

Peak #	RetTime [min]	Type	Width [min]	Area [mAU*s]	Height [mAU]	Area %
1	7.194	MM	0.4340	521.42548	20.02467	3.9871
2	10.775	MM	0.6669	1.25564e4	313.79904	96.0129

Totals : 1.30778e4 333.82371

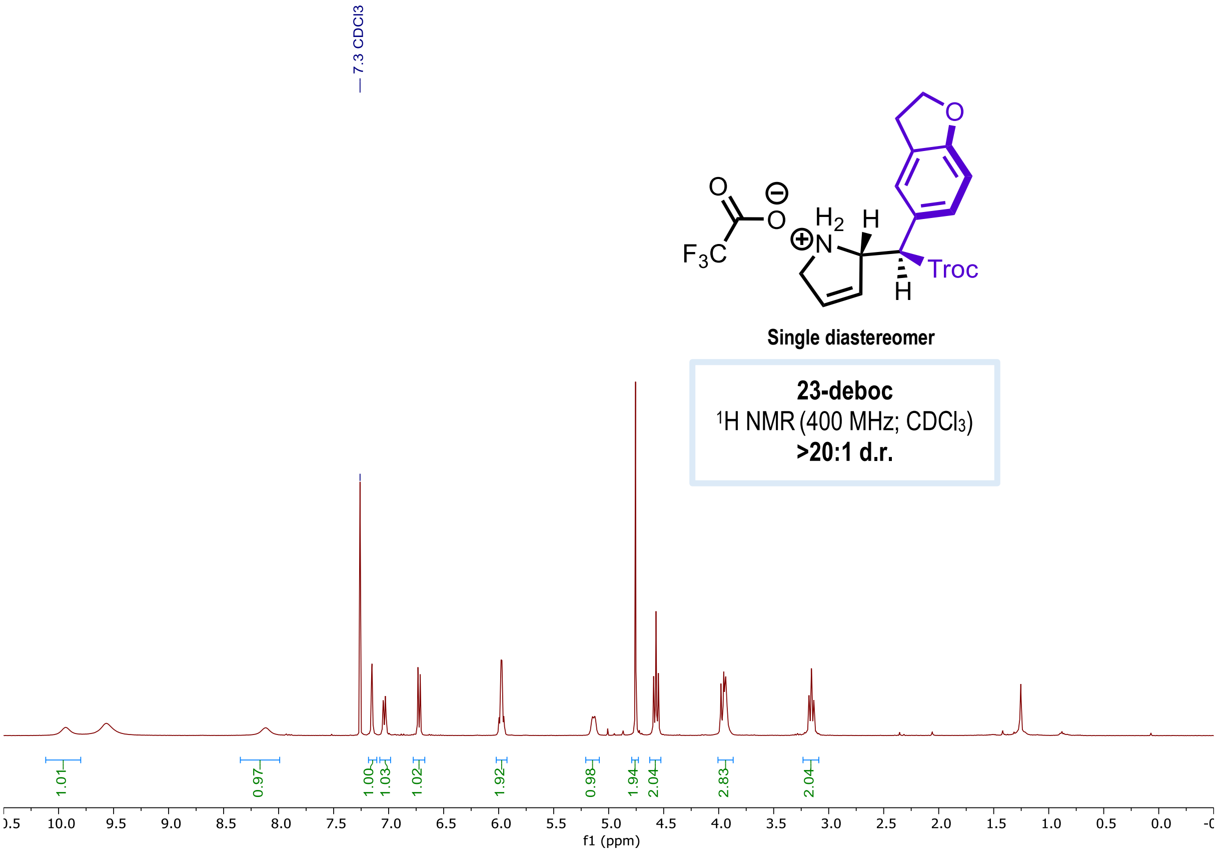

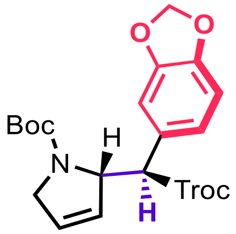

tert-butyl (S)-2-((R)-1-(benzo[d][1,3]dioxol-5-yl)-2-oxo-2-(2,2,2-trichloroethoxy)ethyl)-2,5-dihydro-1H-pyrrole-1-carboxylate (24)

To an 8 mL vial charged with a stir-bar and 0.5 g of activated 4Å MS was added 2,2,2-trichloroethyl 2-(benzo[d][1,3]dioxol-5-yl)-2-diazoacetate (169 mg, 1 equiv, 0.500 mmol) and tert-butyl 2,5-dihydro-1H-pyrrole-1-carboxylate (254 mg, 3 equiv, 1.50 mmol). The reaction vial was sealed, backfilled with nitrogen three times, and the vial topped with a nitrogen balloon. To the vial containing the diazo and olefin was added CH_2Cl_2 (1.5 mL). The solution was stirred at room temperature for at least 10 minutes. A stock solution of Rh_2(*S*-PTAD)$_4$ (390 µg, 500 µL CH_2Cl_2, 0.0005 molar, 0.0005 equiv, 0.250 µmol) was added into the reaction solution containing diazo and olefin drop-wise over 15 seconds. The reaction mixture was then allowed to stir for 20 hours at room temperature. After the elapsed time, the reaction mixture was filtered through a celite plug and the filtrate concentrated *in vacuo* at 45 °C. The crude was dry-loaded onto SiO_2 and subjected to SiO_2 flash chromatography using pentane/diethyl ether (Biotage Sfär DLV 50 g column as the running-column and Biotage Sfär DLV 50 g column for the dry-load; 100 mL/min flow-rate; 210/230 nm detector; 0% 6CV → 1% 6CV → 3% 6CV → 5% 6CV → 7% 6CV → 10% 6CV → 13% 6CV → 16% 12CV; POI elutes at 13% ether). The collected fractions were concentrated *in vacuo* at 45 °C to afford tert-butyl (S)-2-((R)-1-(benzo[d][1,3]dioxol-5-yl)-2-oxo-2-(2,2,2-trichloroethoxy)ethyl)-2,5-dihydro-1H-pyrrole-1-carboxylate (169 mg, 353 µmol, 70.6% yield, 83% ee, >20:1 d.r.) as a clear pale-yellow oil.

^{1}H NMR (600 MHz, Chloroform-*d*; as a 0.53:0.47 mixture of rotamers) δ 6.98 (Ar–H brs, 0.53H, major rotamer), 6.93 (Ar–H brs, 0.47H, minor rotamer), 6.87 (Ar–H d, *J* = 8.6 Hz, 0.53H, major rotamer), 6.81 (Ar–H d, *J* = 8.6 Hz, 0.47H, minor rotamer), 6.77 (Ar–H dd, *J* = 8.6, 8.6 Hz, 1H, mixture of rotamers), 6.01 – 5.89 (C–H$_{methylene}$ & =C–H m's, 2.53H, mixture of rotamers [1.41H minor rotamer + 1.06H major rotamer = 2.53H]), 5.86 – 5.72 (=C–H m, 1.53H, mixture of rotamers [0.47H minor rotamer + 1.06H major rotamer = 2.53H]), 4.93 – 4.87 (C–H$_{\alpha\text{-}N,C\text{-}2}$ brm, 0.53H, major rotamer), 4.82 (C–H$_{methylene}$ d, *J* = 12.0 Hz, 0.53H, major rotamer), 4.77 (C–H$_{methylene}$ & C–H$_{\alpha\text{-}N,C\text{-}2}$ d & brm, *J* = 12.0 Hz, 0.94H, minor rotamers), 4.67 – 4.61 (C–H$_{methylene}$ overlapping d's, *J* = 12.0 Hz, 1H mixture of rotamers [0.47H minor rotamer + 0.53H major rotamer = 1.0H]), 4.61 – 4.53 (C–H$_{benzylic}$ brm, 0.53H, major rotamer), 4.46 – 4.37 (C–H$_{benzylic}$ brm, 0.47H, minor rotamer), 4.25 (C–H$_{\alpha\text{-}N,C\text{-}5}$ d, *J* = 15.4 Hz, 0.47H, minor rotamer), 4.11 (C–H$_{\alpha\text{-}N,C\text{-}5}$ d, *J* = 15.4 Hz, 0.53H, major rotamer), 3.99 (C–H$_{\alpha\text{-}N,C\text{-}5}$ d, *J* = 15.4 Hz, 0.47H, minor rotamer), 3.93 (C–H$_{\alpha\text{-}N,C\text{-}5}$ d, *J* = 15.4 Hz, 0.53H, major rotamer), 1.52 (s, 4.23H, minor rotamer), 1.48 (s, 4.77H, major rotamer).
^{13}C NMR (151 MHz, Chloroform-*d*; as a mixture of rotamers) δ 170.0, 169.6, 154.3, 153.9, 147.9, 147.7, 147.2, 147.0, 128.3, 127.8, 127.3, 126.8, 126.6, 122.7, 122.3, 109.6, 109.1, 108.4, 108.2, 101.2, 101.1, 94.9, 94.7, 80.4, 79.9, 74.3, 74.2, 68.3, 68.2, 54.3, 54.1, 53.7, 52.5, 28.5.
HRMS (ESI +) calc. mass for $C_{20}H_{23}O_6N^{35}Cl_3$ [M + H]$^+$ 478.0586; obs. mass for $C_{20}H_{23}O_6N^{35}Cl_3$ [M + H]$^+$ 478.0588.
[α]$^{20}_D$: -86.2° (c = 0.165 g/100 mL, EtOAc).
HPLC (Chiralpak AD-H column; sample dissolved in 20:80 IPA:*n*-Heptane; 3% IPA in *n*-hexane; 1 mL min^{-1} flow-rate; 30 min; UV 230 nm): retention times of 7.73 min (minor) and 9.96 min (major); 83% ee with Rh_2(*S*-PTAD)$_4$.
Determination of d.r.: *N*-Boc deprotection (1.5 equiv of *p*-Toluenesulfonic acid monohydrate in EtOAc [0.1 M] at 60 °C, 4 hours); observed >20:1 d.r.

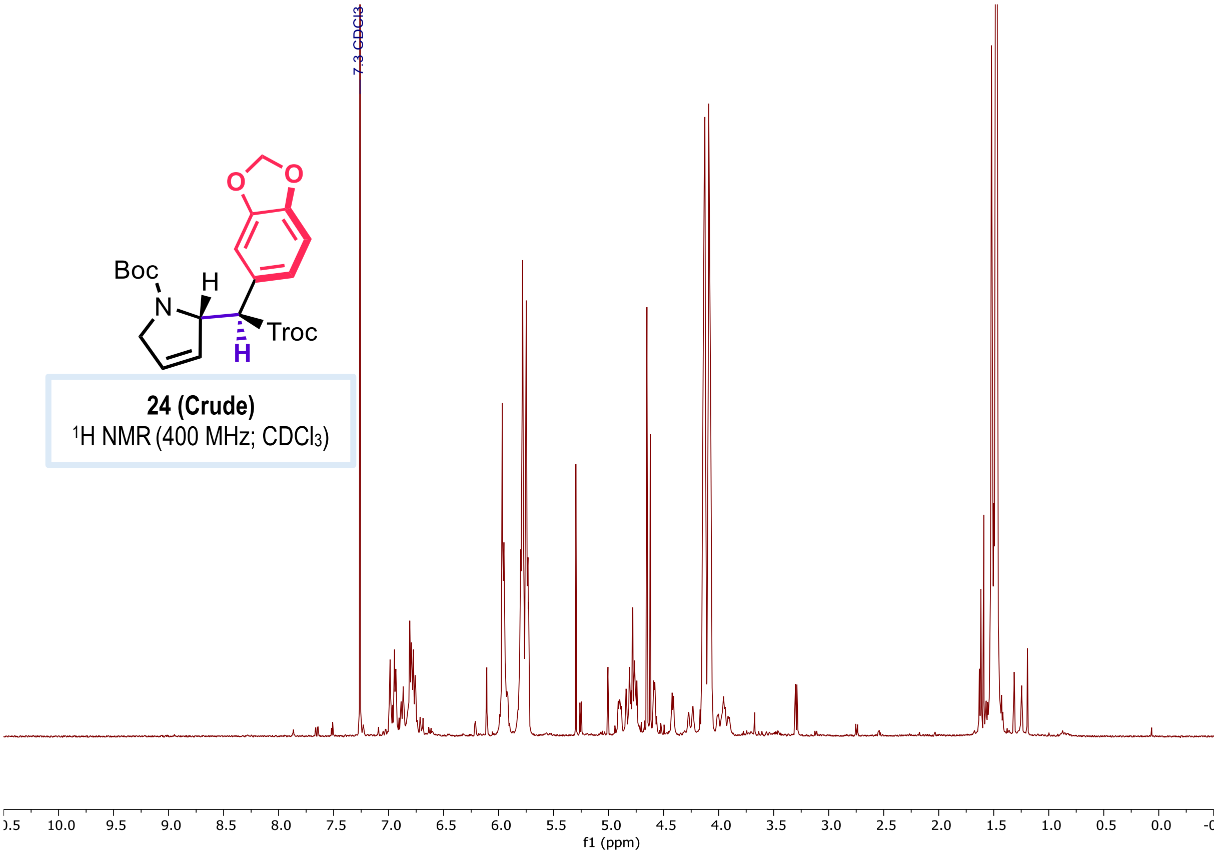

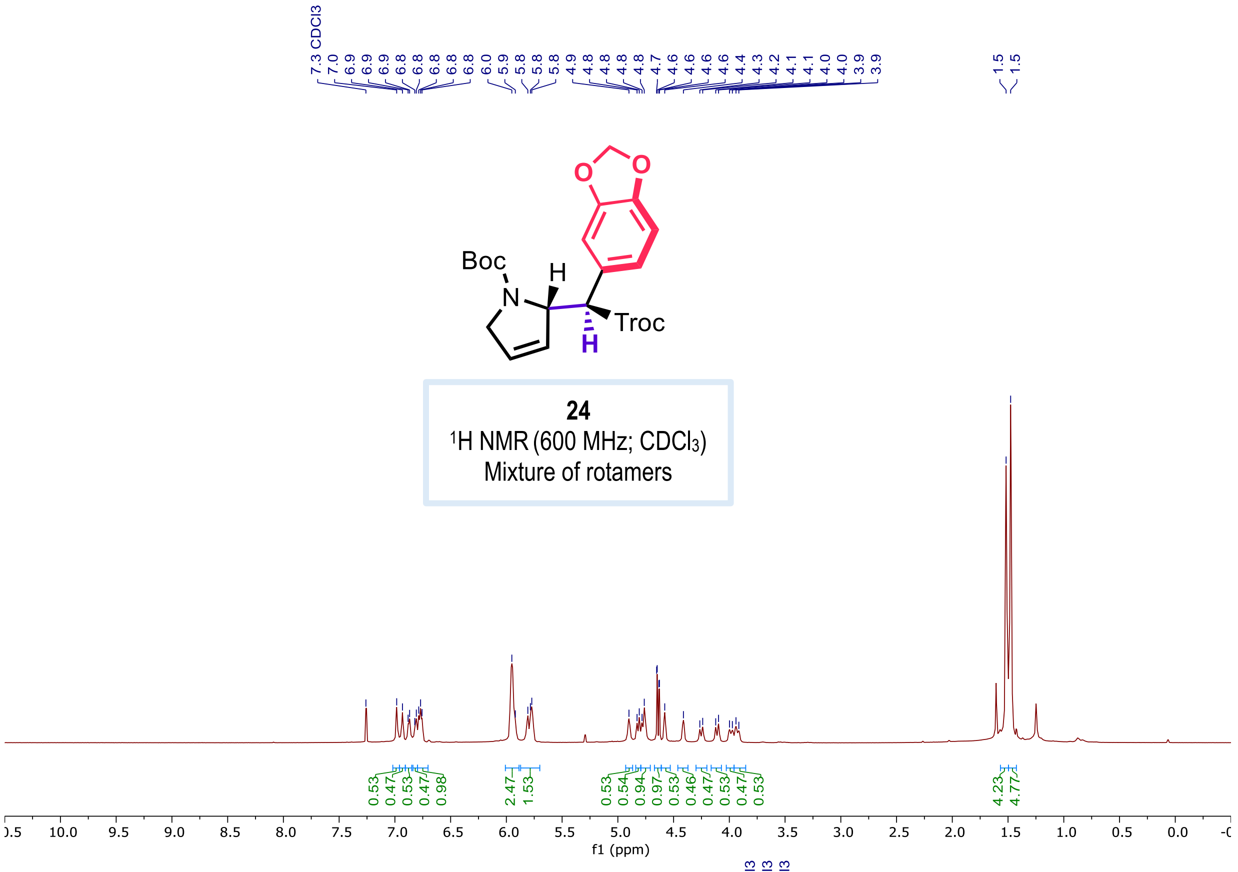
Boc
H
N
Troc
H
24
1H NMR (600 MHz; CDCl3)
Mixture of rotamers
f1 (ppm)

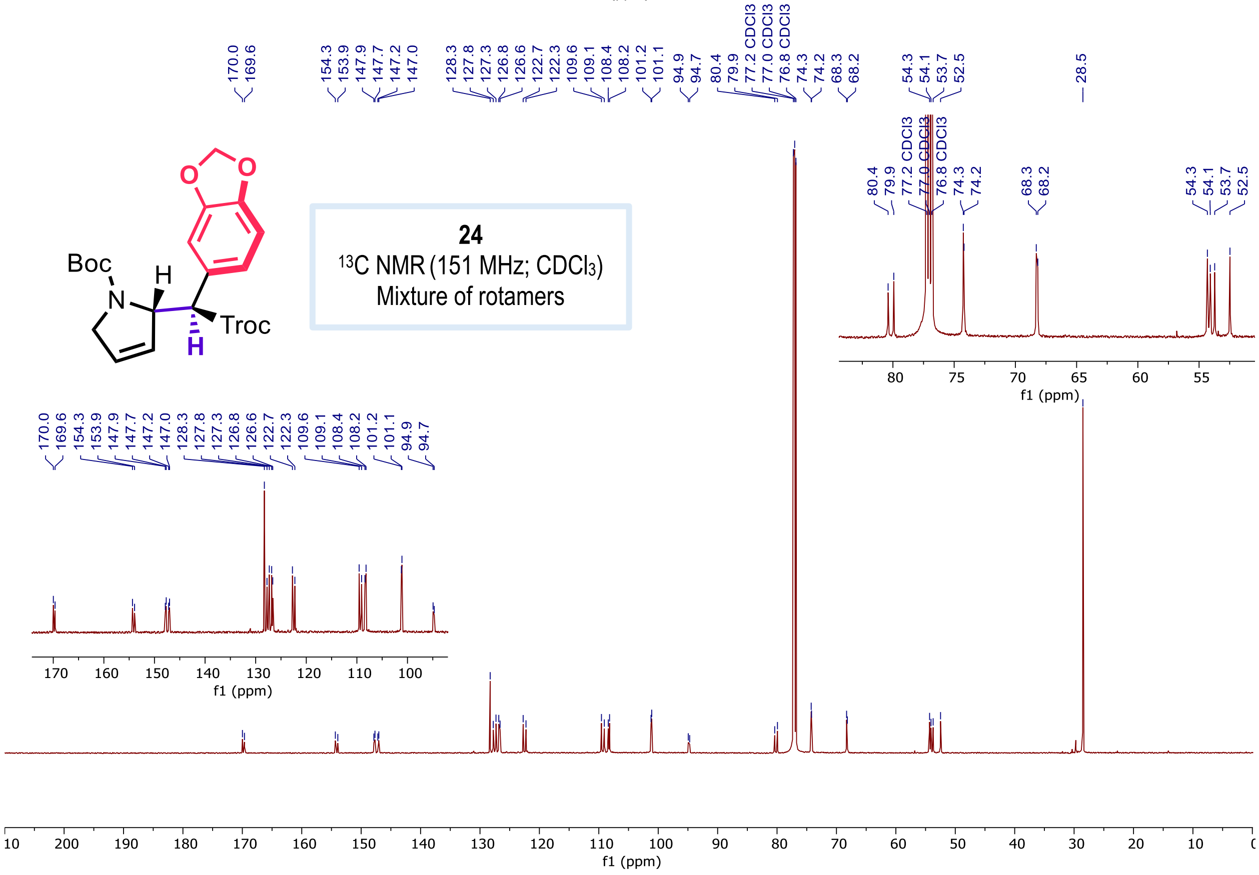
Boc
H
N
Troc
H
24
13C NMR (151 MHz; CDCl3)
Mixture of rotamers
f1 (ppm)

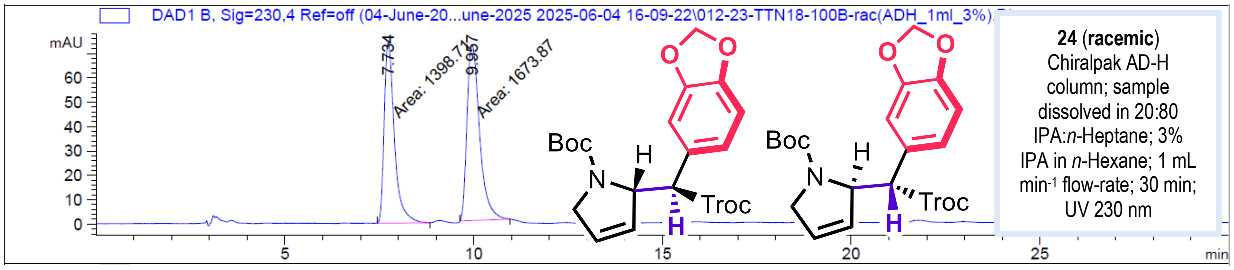

Signal 2: DAD1 B, Sig=230,4 Ref=off

Peak #	RetTime [min]	Type	Width [min]	Area [mAU*s]	Height [mAU]	Area %
1	7.734	MM	0.3152	1398.70776	73.95758	45.5222
2	9.957	MM	0.3877	1673.87354	71.95851	54.4778

Totals : 3072.58130 145.91609

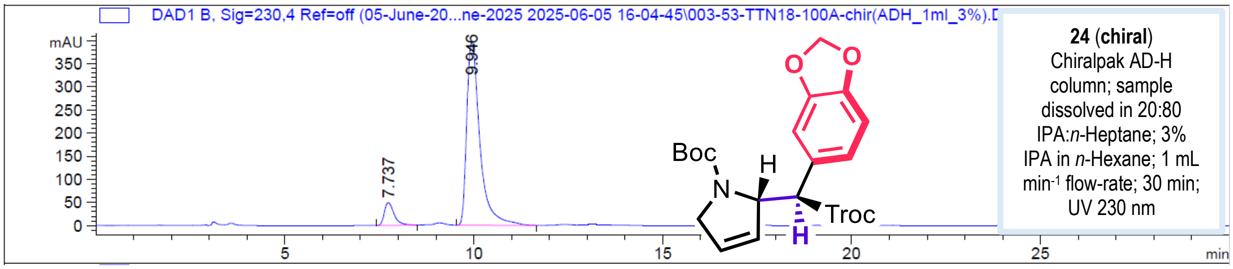

Signal 2: DAD1 B, Sig=230,4 Ref=off

Peak #	RetTime [min]	Type	Width [min]	Area [mAU*s]	Height [mAU]	Area %
1	7.737	BV R	0.2403	890.06879	49.02400	8.4917
2	9.946	BB	0.3638	9591.52344	394.94135	91.5083

Totals : 1.04816e4 443.96534

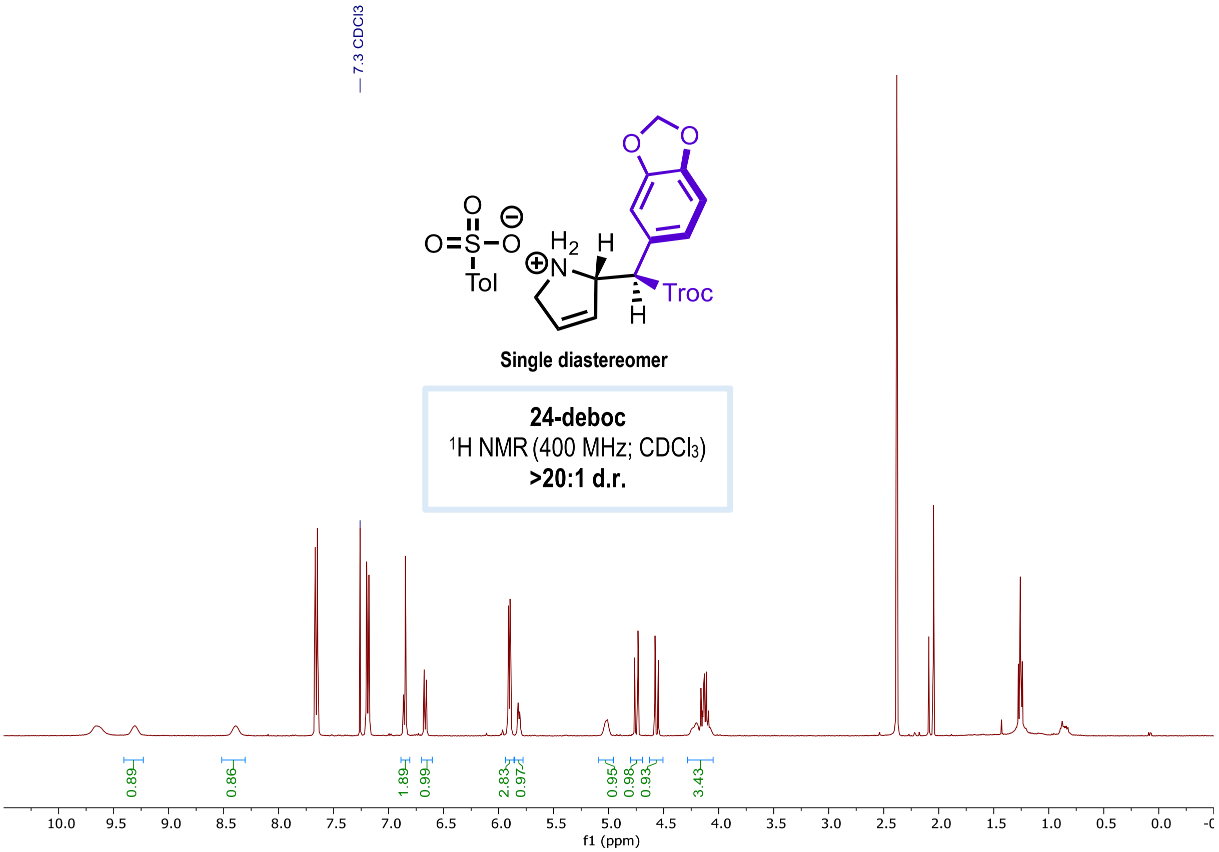

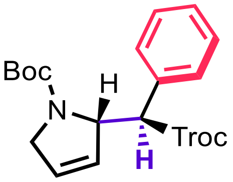

tert-butyl (S)-2-((R)-2-oxo-1-phenyl-2-(2,2,2-trichloroethoxy)ethyl)-2,5-dihydro-1H-pyrrole-1-carboxylate (25)

To an 8 mL vial charged with a stir-bar and 0.5 g of activated 4Å MS was added 2,2,2-trichloroethyl 2-diazo-2-phenylacetate (147 mg, 1 equiv, 0.500 mmol) and tert-butyl 2,5-dihydro-1H-pyrrole-1-carboxylate (254 mg, 3 equiv, 1.50 mmol). The reaction vial was sealed, backfilled with nitrogen three times, and the vial topped with a nitrogen balloon. To the vial containing the diazo and olefin was added CH_2Cl_2 (1.5 mL). The solution was stirred at room temperature for at least 10 minutes. A stock solution of Rh_2(*S*-PTAD)$_4$ (390 µg, 500 µL CH_2Cl_2, 0.0005 molar, 0.0005 equiv, 0.250 µmol) was added into the reaction solution containing diazo and olefin drop-wise over 15 seconds. The reaction mixture was then allowed to stir for 20 hours at room temperature. After the elapsed time, the reaction mixture was filtered through a celite plug and the filtrate concentrated *in vacuo* at 45 °C. The crude was dry-loaded onto SiO_2 and subjected to SiO_2 flash chromatography using pentane/diethyl ether (Biotage Sfär DLV 50 g column as the running-column and Biotage Sfär DLV 50 g column for the dry-load; 100 mL/min flow-rate; 210/230 nm detector; 0% 6CV → 1% 6CV → 3% 6CV → 5% 6CV → 7% 6CV → 10% 6CV → 13% 6CV → 16% 12CV; POI elutes at 10% ether with trap). The collected fractions were concentrated *in vacuo* at 45 °C to afford a mixture of product and trap as a clear oil. The material was then dissolved in CH_2Cl_2, the solution passed through a celite plug into a 20 mL vial, and concentrated *in vacuo* at 45 °C. The vial containing the neat material was then subjected to Kugelrohr distillation at 8.0 x 10^{-1} torr vacuum and 95 °C air bath for at least 20 minutes to distill off the trap. The material was cooled to afford tert-butyl (S)-2-((R)-2-oxo-1-phenyl-2-(2,2,2-trichloroethoxy)ethyl)-2,5-dihydro-1H-pyrrole-1-carboxylate (155 mg, 357 µmol, 71.3% yield, 90% ee, >20:1 d.r.) as a pale-yellow oil.

^{1}H NMR (600 MHz, Chloroform-*d*; as a 0.53:0.47 mixture of rotamers) δ 7.45 (Ar–H d, J = 7.3 Hz, 1H, mixture of rotamers), 7.40 (Ar–H d, *J* = 7.3 Hz, 1H, mixture of rotamers), 7.38 – 7.27 (Ar–H m, 3H, mixture of rotamers), 5.97 – 5.89 (=C–H brm, 0.47H, minor rotamer), 5.88 – 5.68 (=C–H brm, 1.53H, mixture of rotamers [0.47H minor rotamer + 1.06H major rotamer = 1.53H]), 5.00 – 4.90 (C–$H_{\alpha\text{-}N,C\text{-}2}$ brm, 0.53H, major rotamer), 4.88 – 4.77 (C–$H_{methylene}$ & C–$H_{\alpha\text{-}N,C\text{-}2}$ d's & brm, *J* = 12.1 Hz each d, 1.47H, mixture of rotamers [0.94H minor rotamer + 0.53H major rotamer = 1.47H]), 4.69 (C–$H_{benzylic}$ d, *J* = 4.4 Hz, 0.53H, major rotamer), 4.65 (C–$H_{methylene}$ d, *J* = 12.1 Hz, 1H, mixture of rotamers [0.47H minor rotamer + 0.53H major rotamer = 1H]), 4.52 (C–$H_{benzylic}$ d, *J* = 4.4 Hz, 0.47H, minor rotamer), 4.27 (C–$H_{\alpha\text{-}N,C\text{-}5}$ d, *J* = 15.4 Hz, 0.47H, minor rotamer), 4.12 (C–$H_{\alpha\text{-}N,C\text{-}5}$ d, *J* = 15.4 Hz, 0.53H, major rotamer), 4.01 (C–$H_{\alpha\text{-}N,C\text{-}5}$ dd, *J* = 15.4, 4.8 Hz, 0.47H, minor rotamer), 3.94 (C–$H_{\alpha\text{-}N,C\text{-}5}$ dd, *J* = 15.4, 4.8 Hz, 0.53H, major rotamer), 1.52 (–C(CH_3)$_3$ s, 4.23H, minor rotamer), 1.49 (–C(CH_3)$_3$ s, 4.77H, major rotamer).
^{13}C NMR (151 MHz, Chloroform-*d*; as a mixture of rotamers) δ 169.9, 169.6, 154.3, 153.9, 134.7, 129.2, 128.7, 128.7, 128.5, 127.8, 127.7, 127.6, 127.3, 126.8, 126.6, 95.0, 94.7, 80.4, 79.9, 74.2, 74.2, 68.2, 68.1, 54.4, 54.1, 52.9, 28.5.
HRMS (APCI -) calc. mass for $C_{19}H_{21}O_4N^{35}Cl_3$ [M - H]$^-$ 432.0542; obs. mass for $C_{19}H_{21}O_4N^{35}Cl_3$ [M - H]- 432.0527.
[α]$^{20}_{D}$: -100.1° (c = 0.220 g/100 mL, EtOAc).
SFC (Regis (S,S) Whelk-O 1 Kromasil column; sample dissolved in *n*-Heptane; 1% cosolvent of 1:1 MeOH:IPA with 0.2% formic acid; 2.5 mL min^{-1} flow-rate; 10 min; UV 210 nm): retention times of 5.10 min (major) and 5.49 min (minor); 90% ee with Rh_2(*S*-PTAD)$_4$.
Determination of d.r.: *N*-Boc deprotection (1.5 equiv of *p*-Toluenesulfonic acid monohydrate in EtOAc [0.1 M] at 60 °C, 4 hours); observed >20:1 d.r.

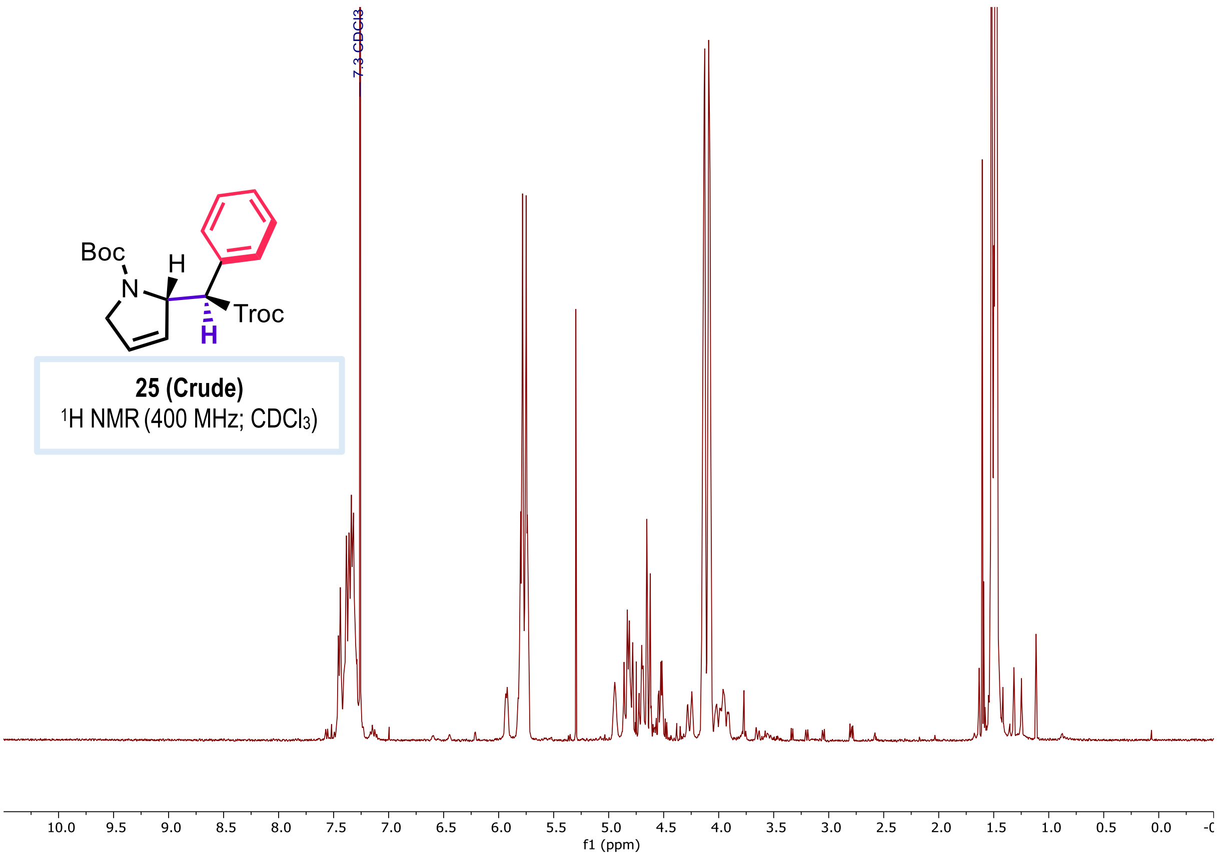

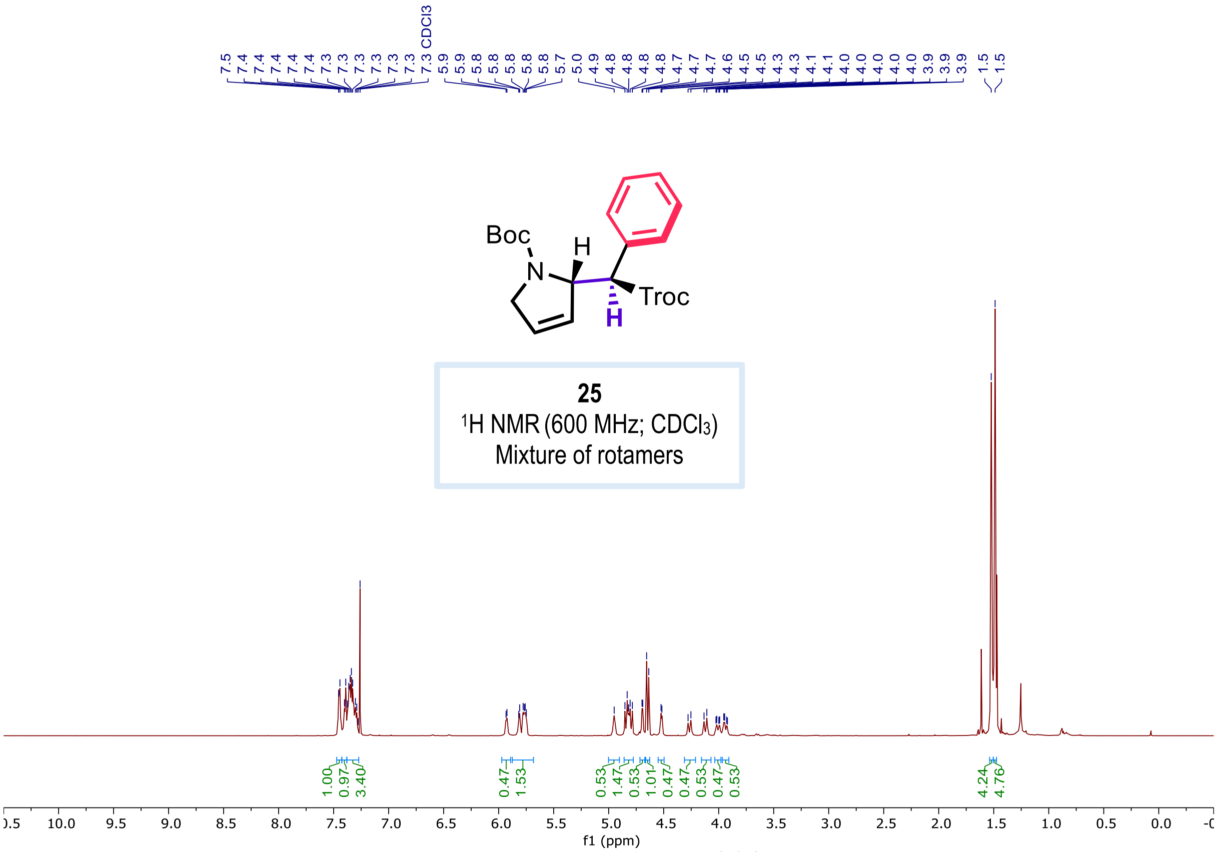
Boc
N
H
Troc
H
25
1H NMR (600 MHz; CDCl3)
Mixture of rotamers
f1 (ppm)

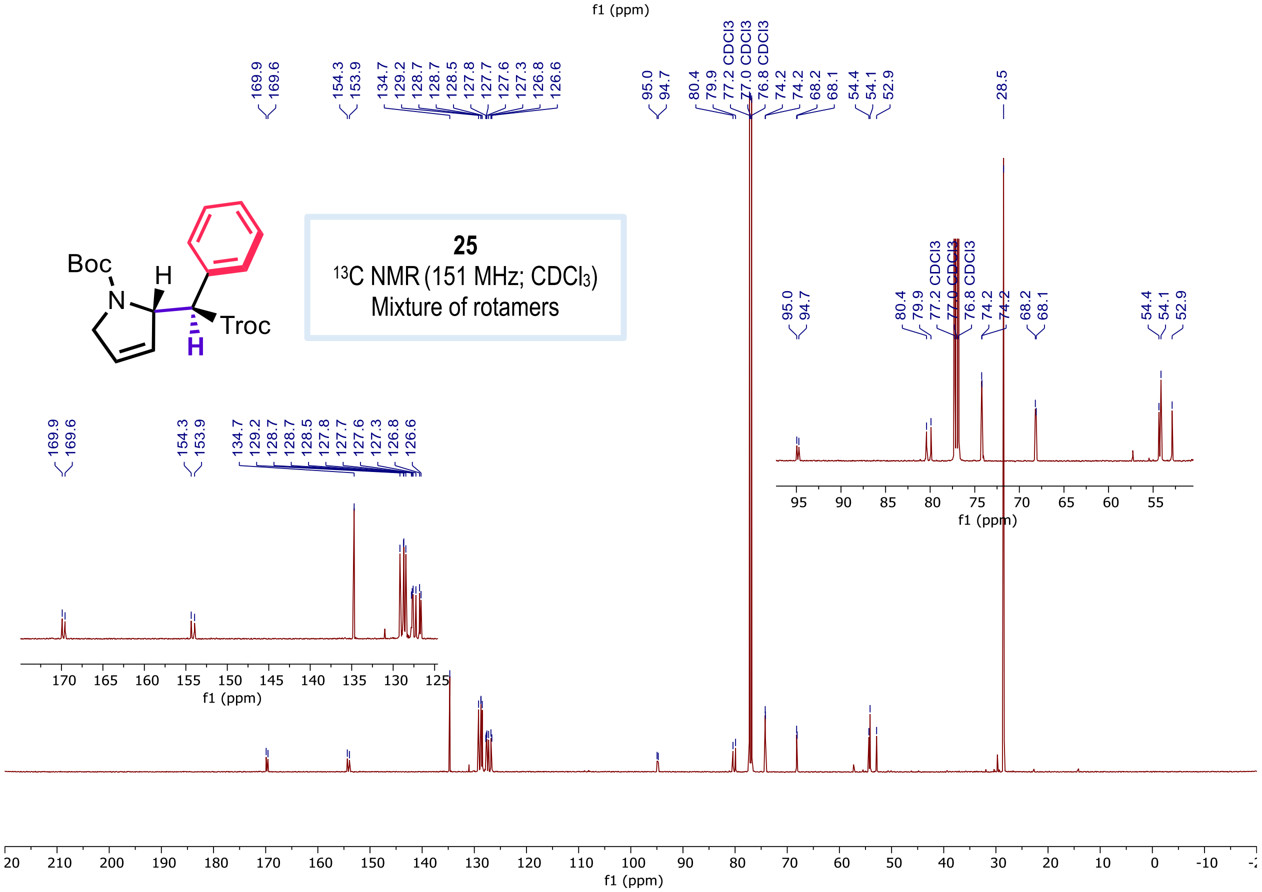
Boc
N
H
Troc
H
25
13C NMR (151 MHz; CDCl3)
Mixture of rotamers
f1 (ppm)

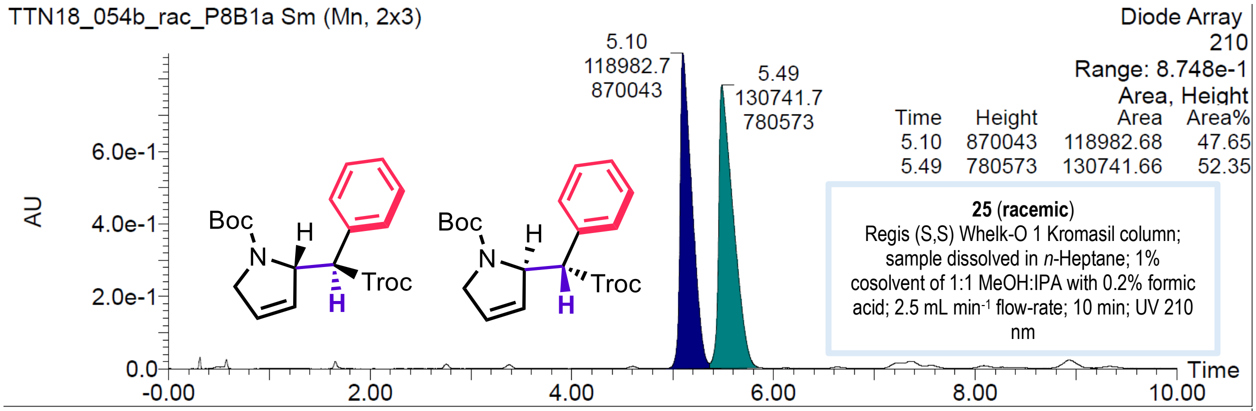

Time	Height	Area	Area%
5.10	870043	118982.68	47.65
5.49	780573	130741.66	52.35

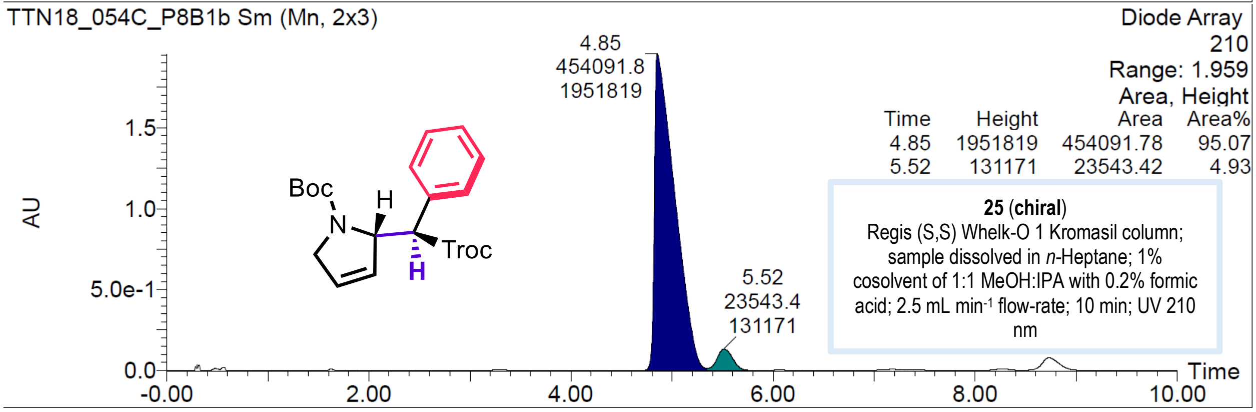

Time	Height	Area	Area%
4.85	1951819	454091.78	95.07
5.52	131171	23543.42	4.93

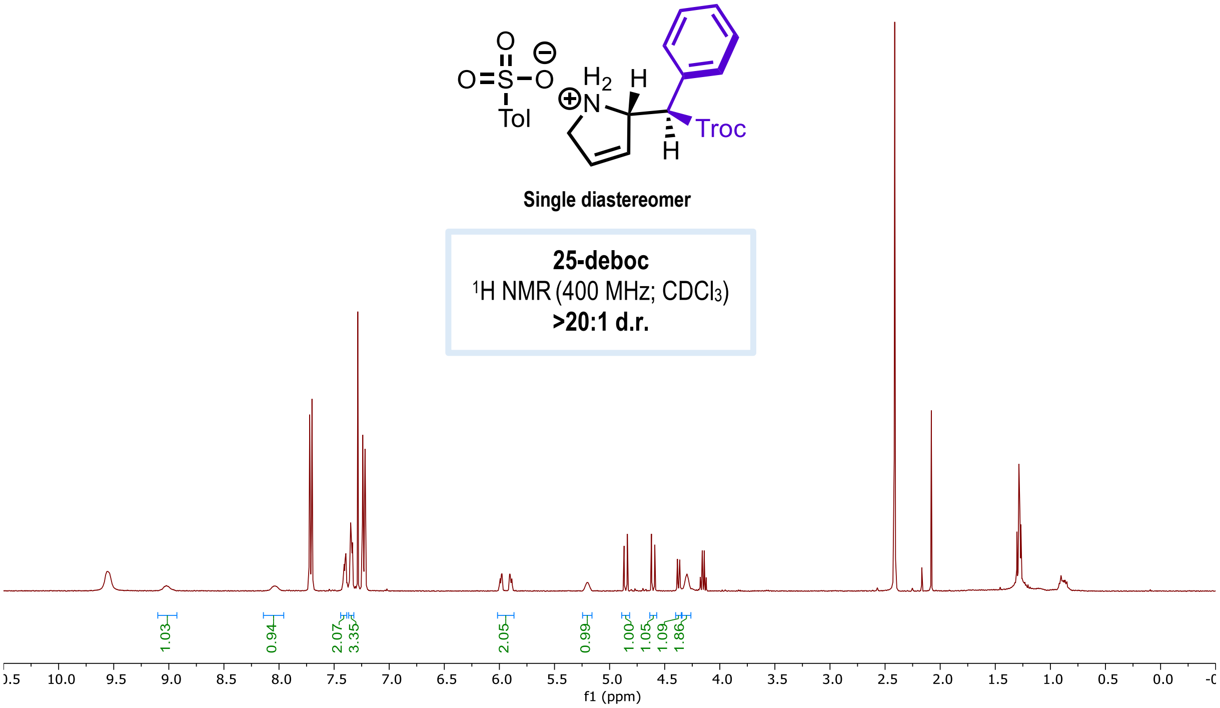

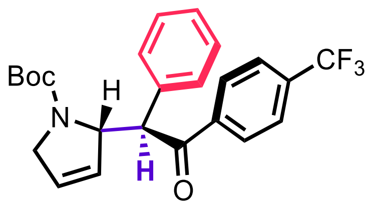

tert-butyl (S)-2-((R)-2-oxo-1-phenyl-2-(4-(trifluoromethyl)phenyl)ethyl)-2,5-dihydro-1H-pyrrole-1-carboxylate (26)

To an 8 mL vial charged with a stir-bar and 0.5 g of activated 4Å MS was added 2-diazo-2-phenyl-1-(4-(trifluoromethyl)phenyl)ethan-1-one (145 mg, 1 equiv, 0.500 mmol) and tert-butyl 2,5-dihydro-1H-pyrrole-1-carboxylate (254 mg, 3 equiv, 1.50 mmol). The reaction vial was sealed, backfilled with nitrogen three times, and the vial topped with a nitrogen balloon. To the vial containing the diazo and trap was added CH_2Cl_2 (1.5 mL). The solution was stirred at room temperature for at least 10 minutes. A stock solution of Rh_2(*S*-PTAD)$_4$ (390 μg, 500 μL CH_2Cl_2, 0.0005 molar, 0.0005 equiv, 0.250 μmol) was added into the reaction solution containing diazo and olefin drop-wise over 15 seconds. The reaction mixture was then allowed to stir for 20 hours at room temperature. After the elapsed time, the reaction mixture was filtered through a celite plug and the filtrate concentrated *in vacuo* at 45 °C. The crude was dry-loaded onto SiO_2 and subjected to SiO_2 flash chromatography using pentane/diethyl ether (Biotage Sfär DLV 50 g column as the running-column and Biotage Sfär DLV 50 g column for the dry-load; 100 mL/min flow-rate; 210/230 nm detector; 0% 6CV → 1% 6CV → 3% 6CV → 5% 6CV → 7% 6CV → 10% 6CV → 13% 6CV → 16% 12CV; POI elutes at 10% ether with trap). The collected fractions were concentrated *in vacuo* at 45 °C to afford a mixture of product and trap as a clear oil. The material was then dissolved in CH_2Cl_2, the solution passed through a celite plug into a 20 mL vial, and concentrated *in vacuo* at 45 °C. The vial containing the neat material was then subjected to Kugelrohr distillation at 8.0 x 10^{-1} torr vacuum and 95 °C air bath for at least 20 minutes to distill off the trap. The material was cooled to afford tert-butyl (S)-2-((R)-2-oxo-1-phenyl-2-(4-(trifluoromethyl)phenyl)ethyl)-2,5-dihydro-1H-pyrrole-1-carboxylate (156 mg, 362 μmol, 72.3% yield, 90% ee, >20:1 d.r.) as a yellow oil.

^{1}H NMR (600 MHz, Chloroform-*d*; as a 0.6:0.4 mixture of rotamers) δ 8.02 (Ar–H d, *J* = 7.8 Hz, 1.2H, major rotamers), 7.94 (Ar–H d, *J* = 7.8 Hz, 0.8H, minor rotamers), 7.63 (Ar–H d, *J* = 7.8 Hz, 2H, mixture of rotamers), 7.46 (Ar–H d, *J* = 7.5 Hz, 1H, mixture of rotamers), 7.36 – 7.26 (Ar–H m, 4H, mixture of rotamers), 5.95 – 5.75 (=C–H & C–$H_{\alpha\text{-}N,C\text{-}2}$ m, 2.6H, mixture of rotamers), 5.09 – 4.93 (C–$H_{\alpha\text{-}N,C\text{-}2}$ & C–$H_{benzylic}$ m, 1.4H, mixture of rotamers), 4.34 (C–$H_{\alpha\text{-}N,C\text{-}5}$ d, *J* = 15.4 Hz, 0.4H, minor rotamer), 4.10 (C–$H_{\alpha\text{-}N,C\text{-}5}$ d, *J* = 15.4 Hz, 0.6H, major rotamer), 3.96 (C–$H_{\alpha\text{-}N,C\text{-}5}$ brd, *J* = 15.4 Hz, 0.4H, minor rotamer), 3.85 (C–$H_{\alpha\text{-}N,C\text{-}5}$ dd, *J* = 15.4, 4.1 Hz, 0.6H, major rotamer), 1.37 (–C(CH_3)$_3$ s, 5.4H, major rotamer), 1.31 (–C(CH_3)$_3$ s, 3.6H, minor rotamer).
^{13}C NMR (151 MHz, Chloroform-*d*; as a mixture of rotamers) δ 198.9, 198.0, 154.4, 154.3, 140.7, 139.9, 135.6, 135.4, 134.3, 134.1, 133.9, 133.7, 129.1, 128.9, 128.7, 127.7, 127.6, 127.3, 126.4, 125.6, 125.5, 125.5, 122.7 (**–CF_3** dq, *J* = 270.8, 14.5 Hz), 80.1, 79.8, 68.8, 67.9, 57.5, 54.3, 53.8, 29.7, 28.3, 28.2.
^{19}F NMR (376 MHz, Chloroform-*d*; as a mixture of rotamers) δ -63.2, -63.2.
HRMS (APCI -) calc. mass for $C_{24}H_{23}O_3NF_3$ [M - H]$^-$ 430.1636; obs. mass for $C_{24}H_{23}O_3NF_3$ [M - H]$^-$ 430.1621.
$[\alpha]^{20}_D$: -31.0° (c = 0.245 g/100 mL, EtOAc).
SFC (Regis (S,S) Whelk-O 1 Kromasil column; sample dissolved in *n*-Heptane; 1% cosolvent of 1:1 MeOH:IPA with 0.2% formic acid; 2.5 mL min^{-1} flow-rate; 10 min; UV 210 nm): retention times of 4.96 min (major) and 5.72 min (minor); 90% ee with Rh_2(*S*-PTAD)$_4$.
Determination of d.r.: *N*-Boc deprotection (10 equiv of TFA in CH_2Cl_2 [0.25 M] at 25 °C, 24 hours); observed >20:1 d.r.

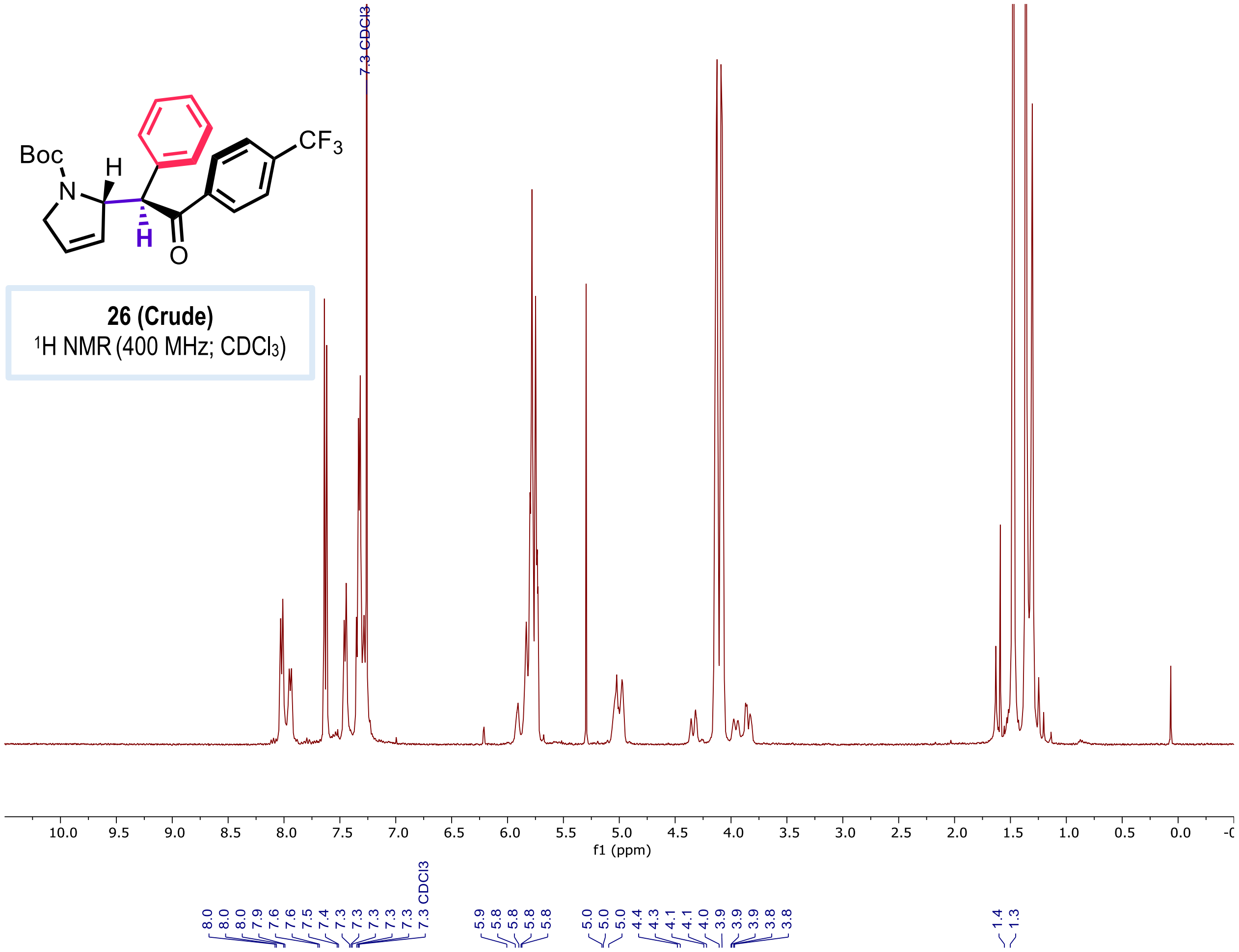
Boc
N
H
H
O
CF_3
26 (Crude)
^{1}H NMR (400 MHz; $CDCl_3$)
7.3 CDCl3
f1 (ppm)

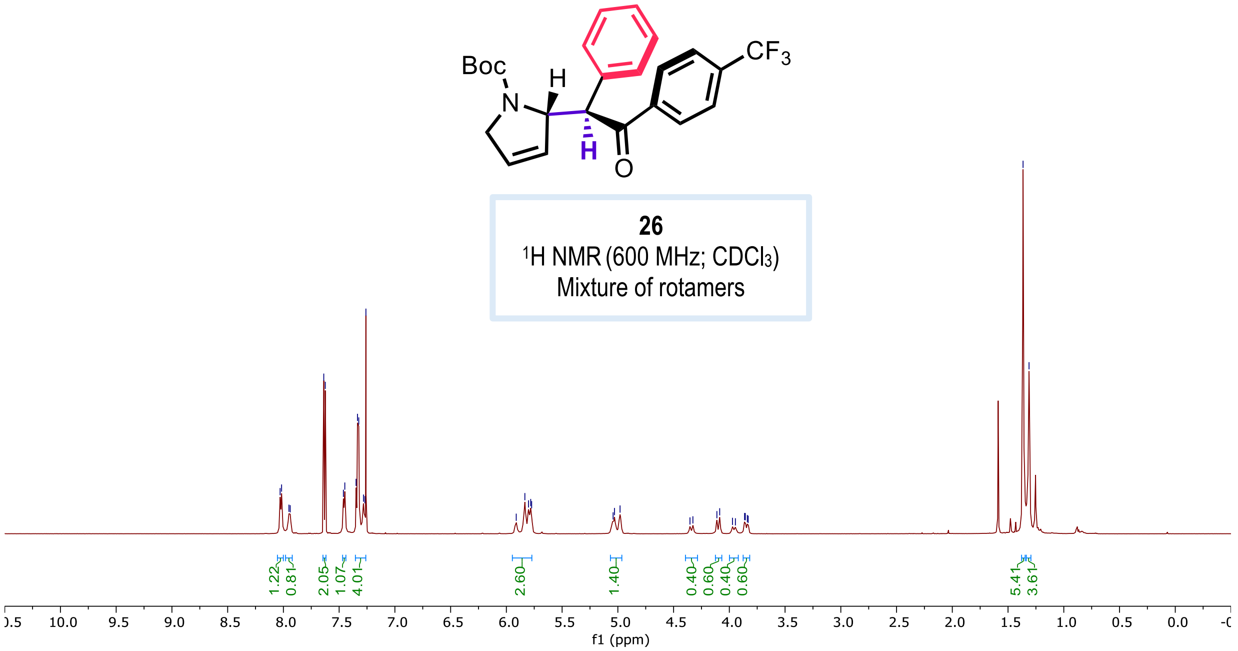
Boc
N
H
H
O
CF_3
26
^{1}H NMR (600 MHz; $CDCl_3$)
Mixture of rotamers
1.22
0.81
2.05
1.07
4.01
2.60
1.40
0.40
0.60
0.40
0.60
5.41
3.61
f1 (ppm)

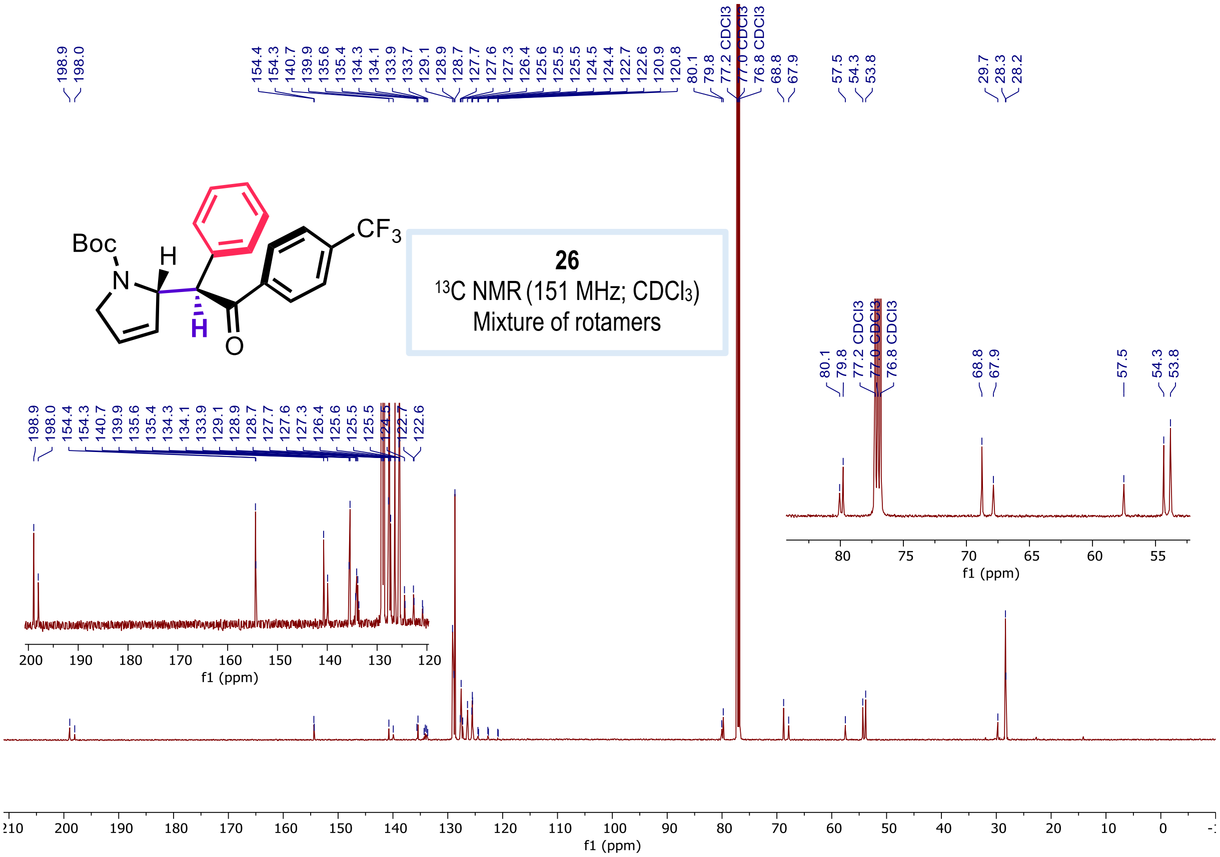
26
^{13}C NMR (151 MHz; CDCl$_3$)
Mixture of rotamers
f1 (ppm)

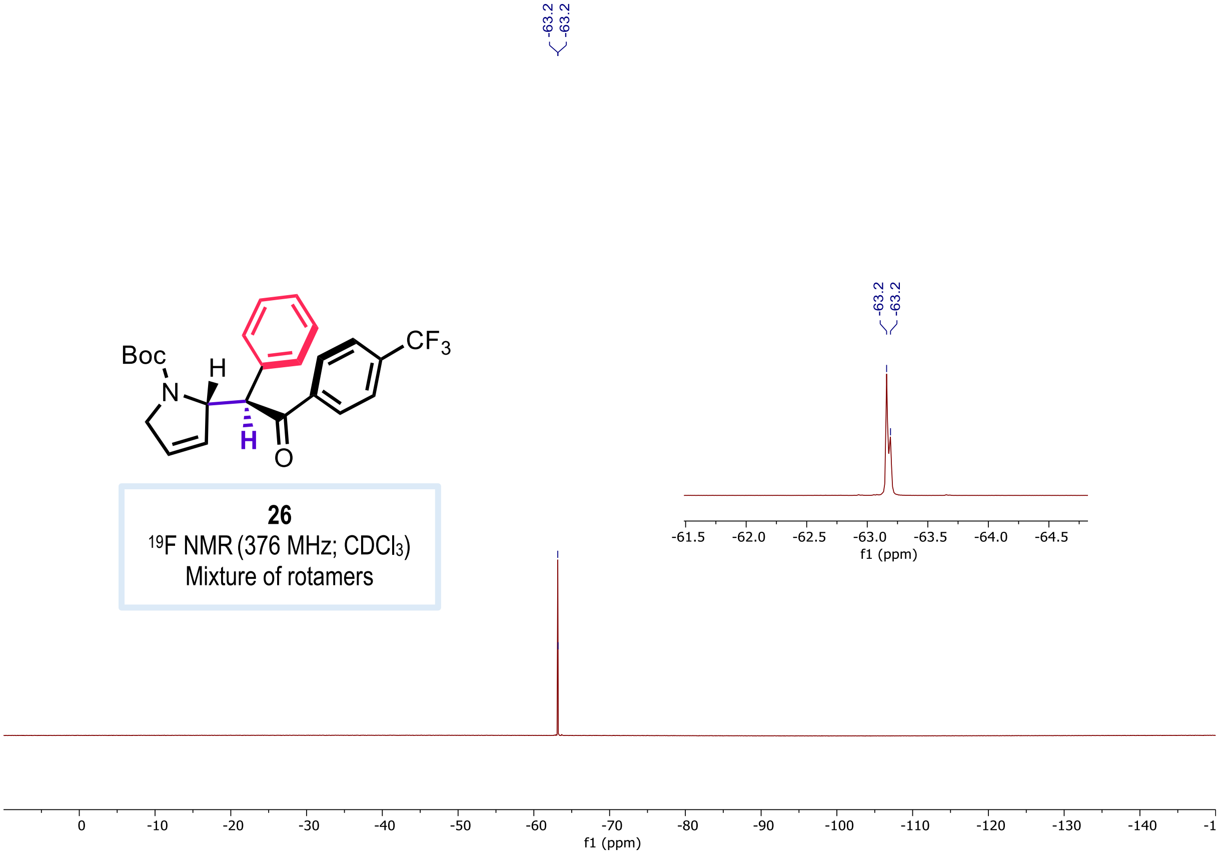
26
^{19}F NMR (376 MHz; CDCl$_3$)
Mixture of rotamers
f1 (ppm)

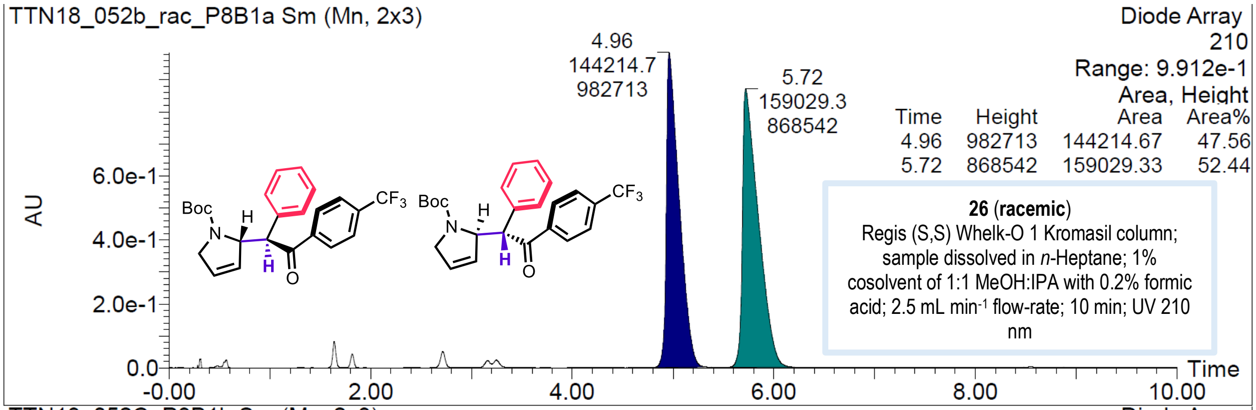
TTN18_052b_rac_P8B1a Sm (Mn, 2x3)
Diode Array
210
Range: 9.912e-1
Area, Height
4.96
144214.7
982713
5.72
159029.3
868542
Time Height Area Area%
4.96 982713 144214.67 47.56
5.72 868542 159029.33 52.44
26 (racemic)
Regis (S,S) Whelk-O 1 Kromasil column; sample dissolved in *n*-Heptane; 1% cosolvent of 1:1 MeOH:IPA with 0.2% formic acid; 2.5 mL min-1 flow-rate; 10 min; UV 210 nm
AU
Time

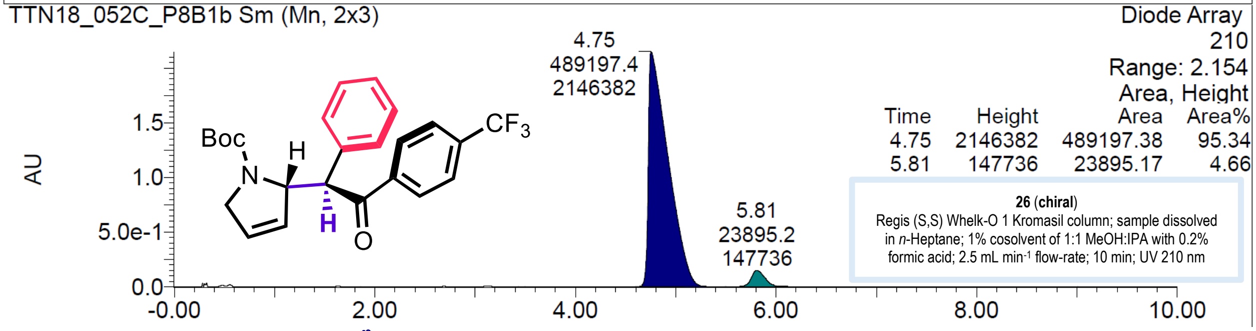
TTN18_052C_P8B1b Sm (Mn, 2x3)
Diode Array
210
Range: 2.154
Area, Height
4.75
489197.4
2146382
5.81
23895.2
147736
Time Height Area Area%
4.75 2146382 489197.38 95.34
5.81 147736 23895.17 4.66
26 (chiral)
Regis (S,S) Whelk-O 1 Kromasil column; sample dissolved in *n*-Heptane; 1% cosolvent of 1:1 MeOH:IPA with 0.2% formic acid; 2.5 mL min-1 flow-rate; 10 min; UV 210 nm
AU

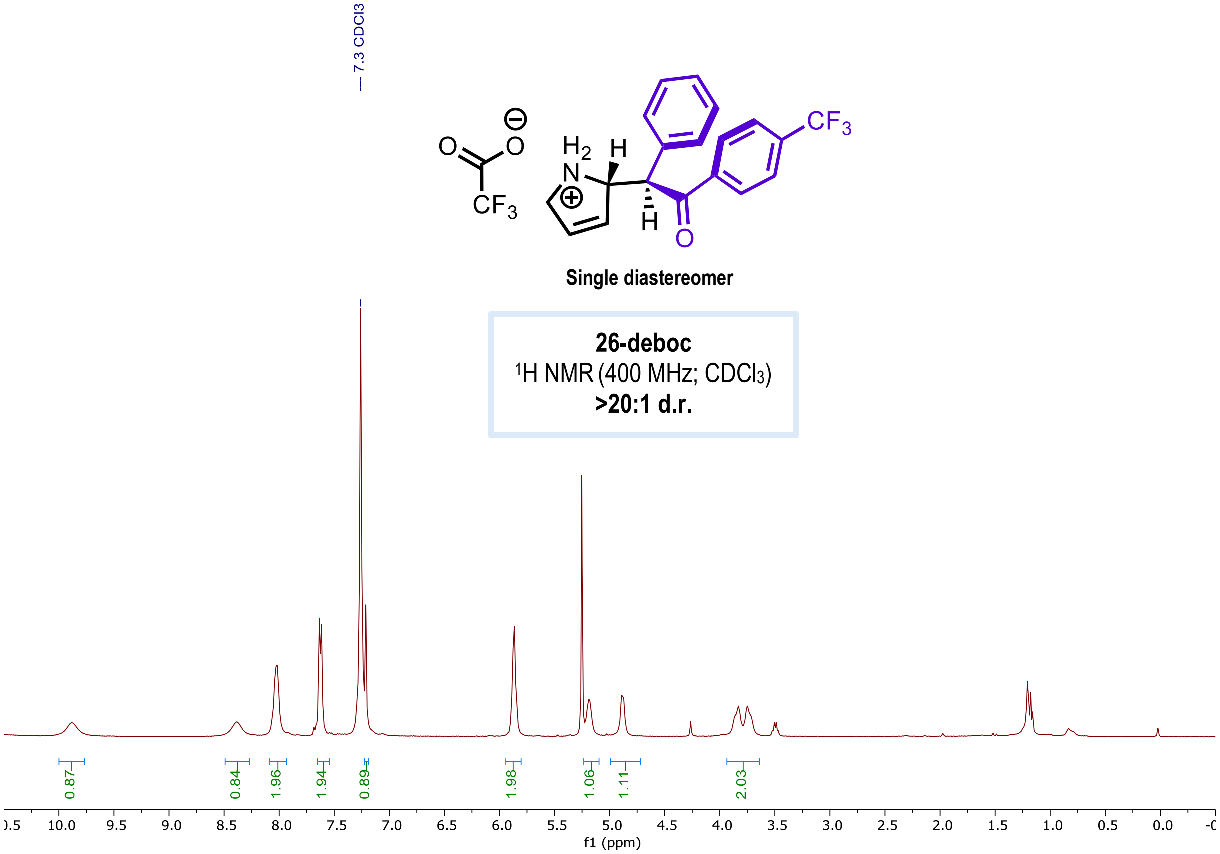
7.3 CDCl3
Single diastereomer
26-deboc
1H NMR (400 MHz; CDCl3)
>20:1 d.r.
f1 (ppm)

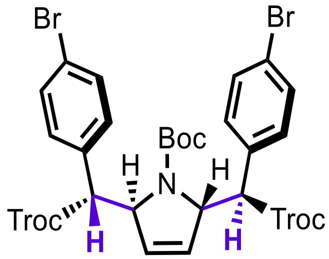

bis(2,2,2-trichloroethyl) 2,2'-((2S,5S)-1-(tert-butoxycarbonyl)-2,5-dihydro-1H-pyrrole-2,5-diyl)(2R,2'R)-bis(2-(4-bromophenyl)acetate) (28)

To a 16 mL dram vial charged with a stir-bar and 0.5 g of activated 4Å MS was added tert-butyl 2,5-dihydro-1H-pyrrole-1-carboxylate (84.6 mg, 1 equiv, 0.500 mmol) and was backfilled with nitrogen three times. CH_2Cl_2 (0.5 mL) followed by a stock solution of Rh_2(*S*-PTAD)$_4$ (390 µg, 500 µL CH_2Cl_2, 0.0005 molar, 0.0005 equiv, 0.250 µmol) was added to the vessel. The reaction vessel containing olefin and catalyst was stirred at room temperature for at least 10 minutes. After the elapsed time, to the reaction vessel was added 2,2,2-trichloroethyl 2-(4-bromophenyl)-2-diazoacetate (559 mg, 1.00 mL CH_2Cl_2, 1.5 molar, 3 equiv, 1.50 mmol) via a multi-channel syringe pump [*syringe pump settings -- 1 mL syringe, 0.333 mL/hr, diameter 4.71 mm, vol 1.0 mL; Air-Tite or SilverPoint 22Gx4" long hypodermic needle was used*] over 3 hours. After complete addition and an elapsed time of 3 hours, the resulting solution was reacted for an additional 17 hours and then subsequently, filtered through a celite plug and concentrated *in vacuo* at 50 °C. The crude material was dry-loaded onto SiO_2 and subjected to SiO_2 flash chromatography using pentane/diethyl ether (Biotage Sfär DLV 50 g column as the running-column and Biotage Sfär DLV 50 g column for the dry-load; 100 mL/min flow-rate; 210/230 nm detector; 0% 6CV → 1% 6CV → 3% 6CV → 5% 6CV → 7% 6CV → 10% 6CV → 13% 6CV; POI elutes at 10% ether). The collected fractions were concentrated *in vacuo* at 40 °C to afford bis(2,2,2-trichloroethyl) 2,2'-((2S,5S)-1-(tert-butoxycarbonyl)-2,5-dihydro-1H-pyrrole-2,5-diyl)(2R,2'R)-bis(2-(4-bromophenyl)acetate) (276 mg, 322 µmol, 64.3% yield, >99% ee, >20:1 d.r.) as a white foamy amorphous material.

^{1}H NMR (400 MHz, Chloroform-*d*; as a 0.5:0.5 mixture of rotamers) δ 7.49 (Ar–H d, *J* = 8.5 Hz, 2H7.46 (Ar–H d, *J* = 8.5 Hz, 2H), 7.27 (Ar–H d, *J* = 8.5 Hz, 2H), 7.24 (Ar–H d, *J* = 8.5 Hz, 2H), 5.86 (=C–H dt, *J* = 6.6, 1.9 Hz, 1H), 5.73 (=C–H dt, *J* = 6.6, 1.9 Hz, 1H), 4.98 – 4.89 (C–$H_{benzylic}$ & C–$H_{\alpha\text{-}N}$ d & m, *J* = 3.8 Hz, 2H), 4.87 (C–$H_{benzylic}$ d, *J* = 3.8 Hz, 1H4.77 – 4.71 (C–$H_{methylene}$ overlapping d's, *J* = 12.0 Hz each, 2H), 4.67 – 4.57 (C–$H_{methylene}$ & C–$H_{\alpha\text{-}N}$ overlapping d's & m, *J* = 12.0 Hz each, 3H), 1.62 (–C(CH_3)$_3$ s, 9H).
^{13}C NMR (151 MHz, Chloroform-*d*; as a mixture of rotamers) δ 169.3, 168.7, 152.9, 133.5, 133.5, 132.0, 131.6, 131.2, 130.3, 128.0, 127.5, 122.1, 121.9, 94.7, 94.5, 81.5, 74.4, 74.3, 69.2, 69.0, 51.8, 50.4, 28.6.
HRMS (ESI +) calc. mass for $C_{29}H_{28}O_6N^{79}Br_2{}^{35}Cl_6$ [M + H]$^+$ 853.8409; obs. mass for $C_{29}H_{28}O_6N^{79}Br_2{}^{35}Cl_6$ [M + H]$^+$ 853.8433.
$[\alpha]^{20}_D$: -180.4° (c = 0.110 g/100 mL, EtOAc).
SFC (Regis (S,S) Whelk-O 1 Kromasil column; sample dissolved in *n*-Heptane; 10% cosolvent of 1:1 MeOH:IPA with 0.2% formic acid; 2.5 mL min^{-1} flow-rate; 10 min; UV 210 nm): retention times of 4.56 min (major) and 6.13 min (minor); >99% ee with Rh_2(*S*-PTAD)$_4$.
Determination of d.r.: *N*-Boc deprotection (1.5 equiv of *p*-Toluenesulfonic acid monohydrate in EtOAc [0.1 M] at 60 °C, 4 hours); observed >20:1 d.r.

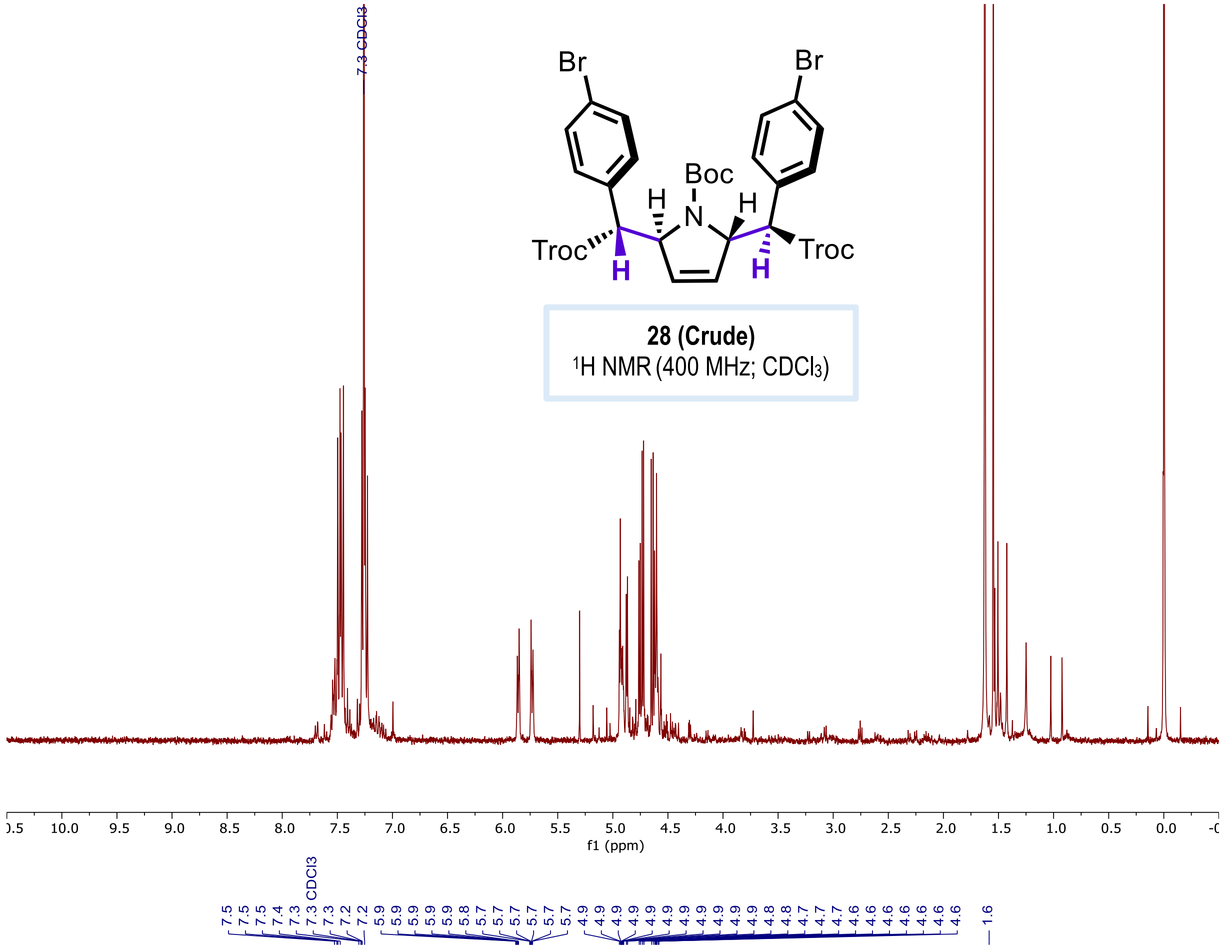
Br
Br
Boc
H
N
H
Troc
Troc
H
H
28 (Crude)
^{1}H NMR (400 MHz; CDCl$_3$)
7.3 CDCl3
f1 (ppm)

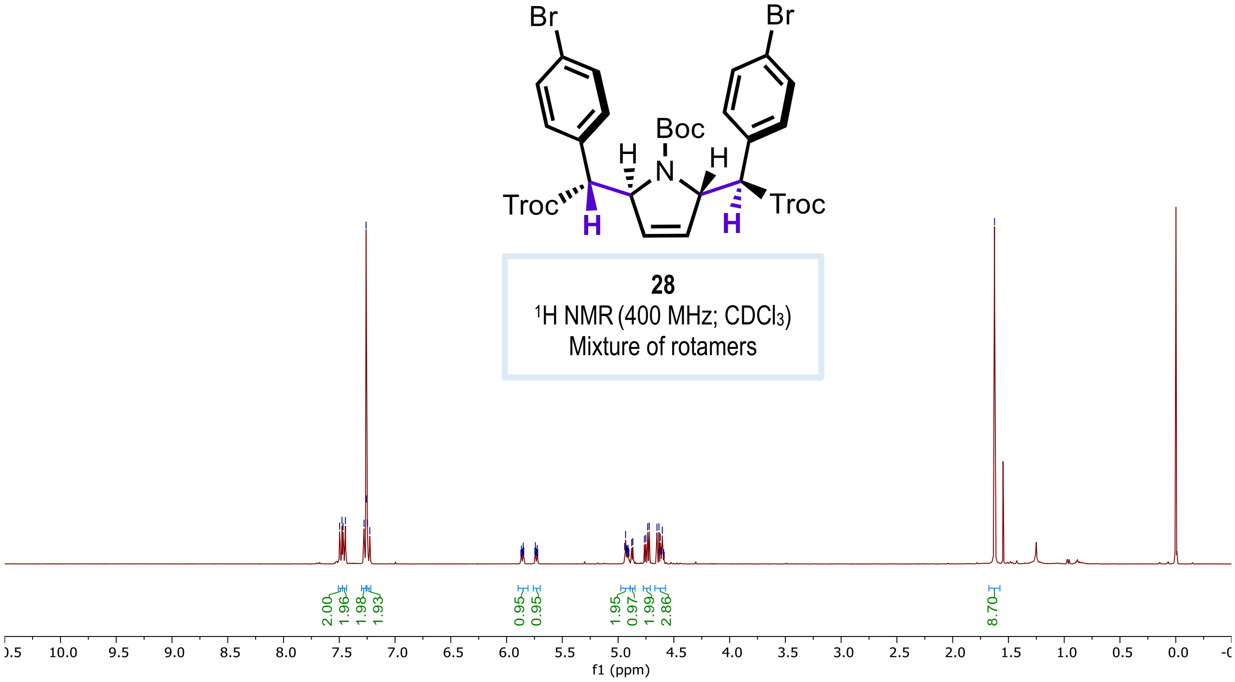
Br
Br
Boc
H
N
H
Troc
Troc
H
H
28
^{1}H NMR (400 MHz; CDCl$_3$)
Mixture of rotamers
2.00
1.96
1.98
1.93
0.95
0.95
1.95
0.97
1.99
2.86
8.70
f1 (ppm)

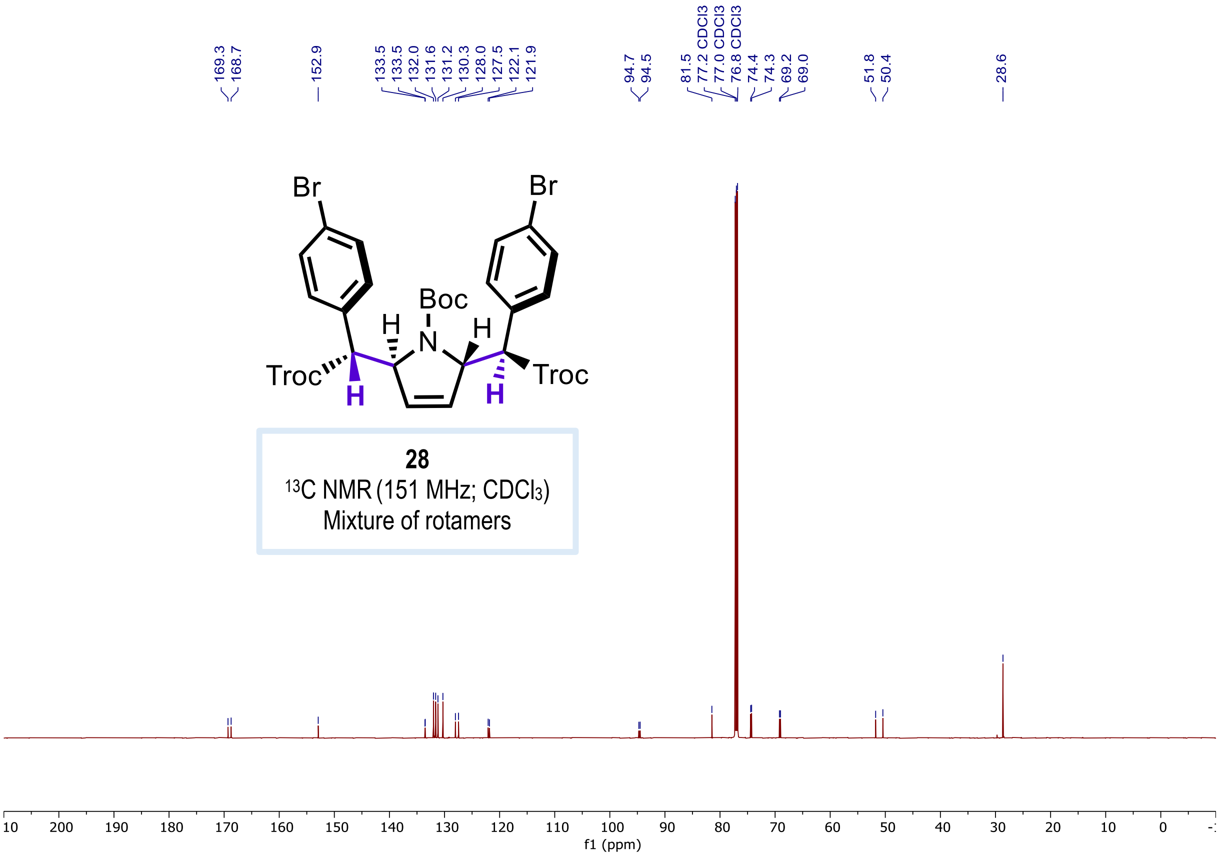
169.3
168.7
152.9
133.5
133.5
132.0
131.6
131.2
130.3
128.0
127.5
122.1
121.9
94.7
94.5
81.5
77.2 CDCl3
77.0 CDCl3
76.8 CDCl3
74.4
74.3
69.2
69.0
51.8
50.4
28.6
Br
Br
Boc
H
N
H
Troc
Troc
H
H
28
^{13}C NMR (151 MHz; $CDCl_3$)
Mixture of rotamers
f1 (ppm)

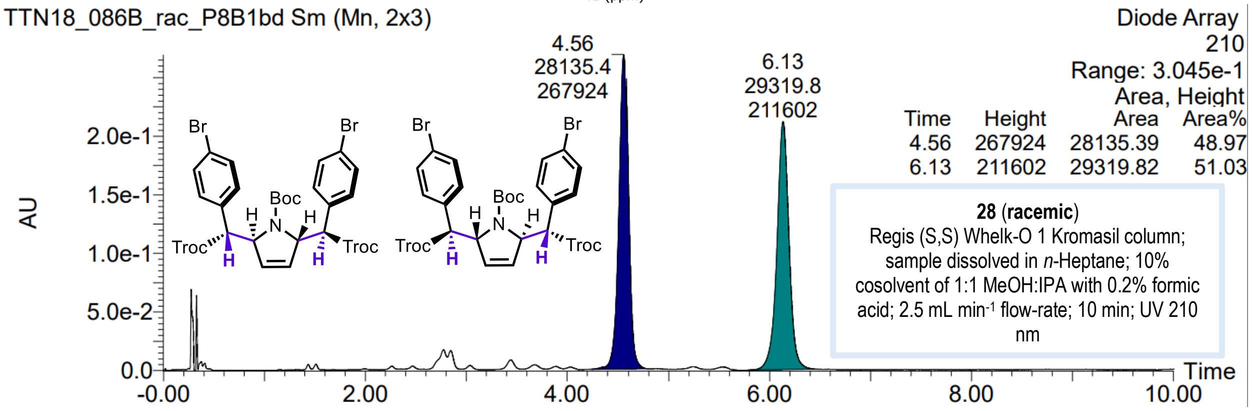
TTN18_086B_rac_P8B1bd Sm (Mn, 2x3)
Diode Array
210
Range: 3.045e-1
Area, Height
4.56
28135.4
267924
6.13
29319.8
211602
Time Height Area Area%
4.56 267924 28135.39 48.97
6.13 211602 29319.82 51.03
AU
Time
28 (racemic)
Regis (S,S) Whelk-O 1 Kromasil column; sample dissolved in n-Heptane; 10% cosolvent of 1:1 MeOH:IPA with 0.2% formic acid; 2.5 mL min-1 flow-rate; 10 min; UV 210 nm

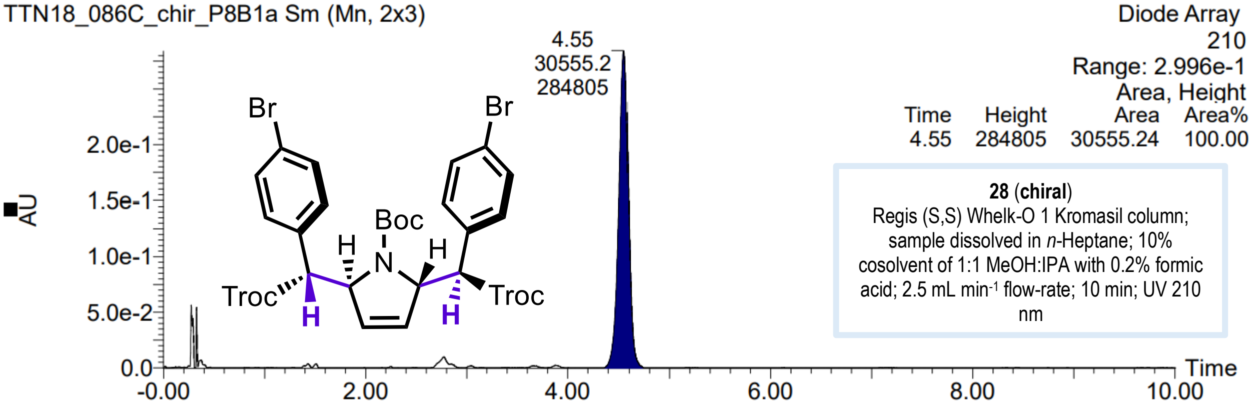
TTN18_086C_chir_P8B1a Sm (Mn, 2x3)
Diode Array
210
Range: 2.996e-1
Area, Height
4.55
30555.2
284805
Time Height Area Area%
4.55 284805 30555.24 100.00
AU
Time
28 (chiral)
Regis (S,S) Whelk-O 1 Kromasil column; sample dissolved in n-Heptane; 10% cosolvent of 1:1 MeOH:IPA with 0.2% formic acid; 2.5 mL min-1 flow-rate; 10 min; UV 210 nm

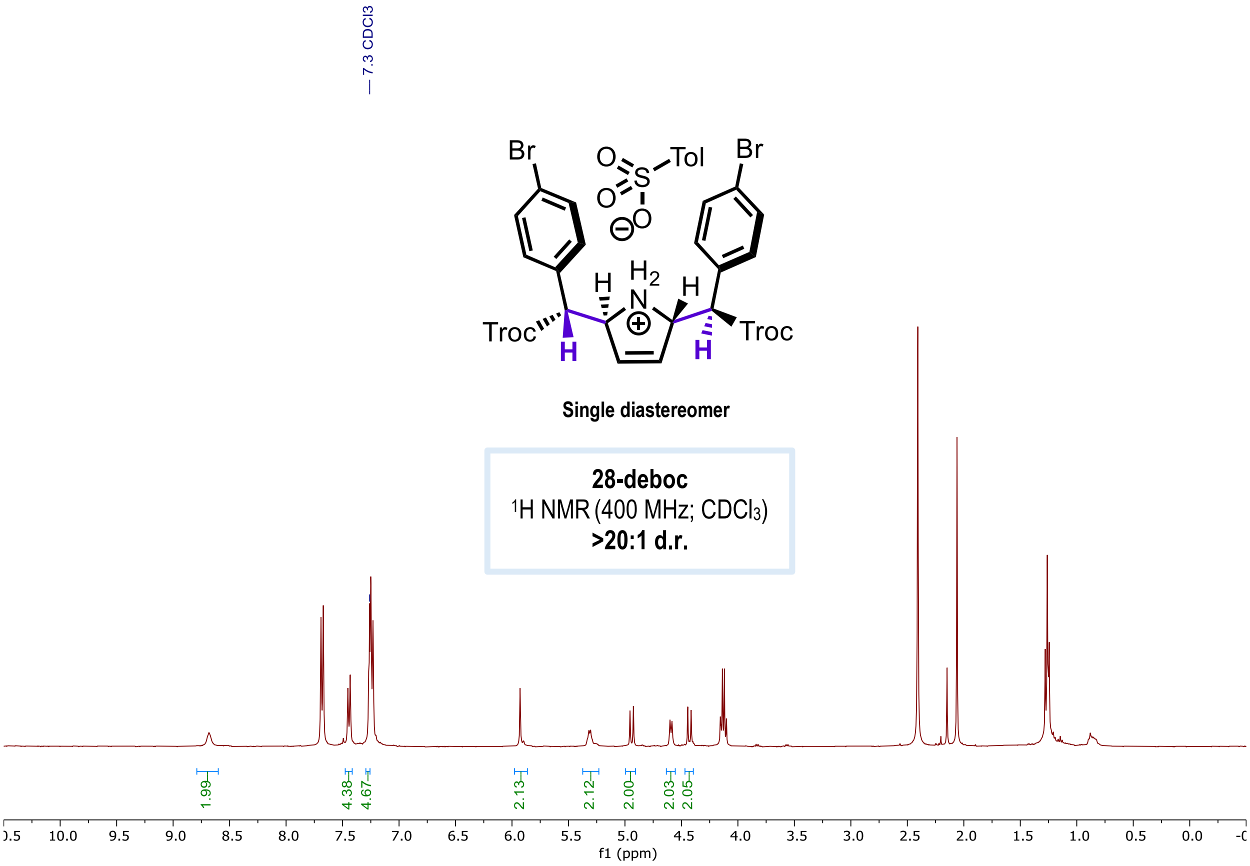

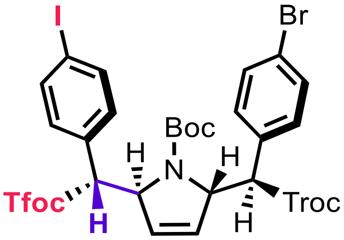

tert-butyl (2S,5S)-2-((R)-1-(4-bromophenyl)-2-oxo-2-(2,2,2-trichloroethoxy)ethyl)-5-((R)-1-(4-iodophenyl)-2-oxo-2-(2,2,2-trifluoroethoxy)ethyl)-2,5-dihydro-1H-pyrrole-1-carboxylate (30)

To an 16 mL dram vial charged with a stir-bar and 1 g of activated 4Å MS was added tert-butyl 2-(1-(4-bromophenyl)-2-oxo-2-(2,2,2-trichloroethoxy)ethyl)-2,5-dihydro-1H-pyrrole-1-carboxylate (782 mg, 1 equiv, 1.52 mmol) and was backfilled with nitrogen three times. CH_2Cl_2 (1.52 mL) followed by a stock solution of Rh_2(*S*-PTAD)$_4$ (1.19 mg, 1.52 mL CH_2Cl_2, 0.0005 molar, 0.0005 equiv, 0.761 µmol) was added to the vessel. The reaction vessel containing olefin and catalyst was stirred at room temperature and for at least 10 minutes. After the elapsed time, to the reaction vessel was added 2,2,2-trifluoroethyl 2-diazo-2-(4-iodophenyl)acetate (1.69 g, 3.04 mL, 1.50 molar, 3.0 equiv, 4.57 mmol) via a multi-channel syringe pump [*syringe pump settings -- 3 mL syringe, 0.666 mL/hr, diameter 9.83 mm, vol 2.00 mL; Air-Tite or SilverPoint 22Gx4" long hypodermic needle was used*] over 3 hours. After complete addition and an elapsed time of 3 hours, the resulting solution was reacted for an additional 17 hours and then subsequently, filtered through a celite plug and concentrated *in vacuo* at 45 °C. The crude material was dry-loaded onto SiO_2 and subjected to SiO_2 flash chromatography using pentane/diethyl ether (Biotage Sfär DLV 50 g column as the running-column and Biotage Sfär DLV 50 g column for the dry-load; 100 mL/min flow-rate; 210/230 nm detector; 0% 6CV → 1% 6CV → 3% 6CV → 5% 6CV → 7% 6CV → 10% 6CV → 13% 6CV → 16%

6CV; POI elutes at 13% ether). The collected fractions were concentrated *in vacuo* at 45 °C to afford tert-butyl (2S,5S)-2-((R)-1-(4-bromophenyl)-2-oxo-2-(2,2,2-trichloroethoxy)ethyl)-5-((R)-1-(4-iodophenyl)-2-oxo-2-(2,2,2-trifluoroethoxy)ethyl)-2,5-dihydro-1H-pyrrole-1-carboxylate (1.15 g, 1.34 mmol, 88.3% yield, 98% ee, >20:1 d.r.) as an amorphous white-yellow material.

^{1}H NMR (600 MHz, Chloroform-*d*; as a 0.55:0.45 mixture of rotamers) δ 7.69 (Ar–H d, *J* = 8.4 Hz, 0.9H, minor rotamer), 7.66 (Ar–H d, *J* = 8.4 Hz, 1.1H, major rotamer), 7.49 (Ar–H d, *J* = 8.4 Hz, 1.1H, major rotamer), 7.46 (Ar–H d, *J* = 8.4 Hz, 0.9H, minor rotamer), 7.28 (Ar–H d, *J* = 8.4 Hz, 0.9H, minor rotamer), 7.24 (Ar–H d, *J* = 8.4 Hz, 1.1H, major rotamer), 7.12 (Ar–H d, *J* = 8.4 Hz, 1.1H, major rotamer), 7.07 (Ar–H d, *J* = 8.4 Hz, 0.9H, minor rotamer), 5.86 (=C–H dt, *J* = 6.5, 2.0 Hz, 0.45H, minor rotamer), 5.82 (=C–H dt, *J* = 6.5, 2.0 Hz, 0.55H, major rotamer), 5.73 (=C–H dt, *J* = 6.5, 2.0 Hz, 0.55H, major rotamer), 5.68 (=C–H dt, *J* = 6.5, 2.0 Hz, 0.45H, minor rotamer), 4.95 – 4.92 (C–$H_{\alpha\text{-}N}$ m, 1H, mixture of rotamers), 4.89 – 4.82 (C–$H_{\alpha\text{-}N}$ & C–$H_{benzylic}$ m & overlapping d's, *J* = 4.4 Hz each d's, 2H, mixture of rotamers), 4.75 – 4.71 (C–$H_{methylene}$ overlapping d's, *J* = 12.0 Hz each d's, 1H, mixture of rotamers), 4.68 – 4.61 (C–$H_{methylene}$ overlapping d's, *J* = 12.0 Hz each d's, 1H), 4.59 (C–$H_{\alpha\text{-}N}$ ddt, *J* = 6.5, 4.4, 2.0 Hz, 0.45H, minor rotamer), 4.56 (C–$H_{\alpha\text{-}N}$ ddt, *J* = 6.5, 4.4, 2.0 Hz, 0.55H, major rotamer), 4.50 – 4.33 (C–$H_{methylene}$ m, 2H, mixture of rotamers), 1.62 (s, 4.95H, major rotamer), 1.61 (s, 4.05H, minor rotamer).
^{13}C NMR (151 MHz, Chloroform-*d*; as a mixture of rotamers) δ 169.3, 169.3, 168.7, 168.7, 152.9, 152.9, 138.0, 137.6, 134.1, 133.9, 133.5, 133.4, 132.0, 131.6, 131.3, 131.2, 130.4, 130.3, 128.2, 127.8, 127.6, 127.2, 123.8, 123.6, 122.1, 122.0, 121.9, 121.8, 94.7, 94.5, 93.7, 93.6, 81.5, 74.4, 74.3, 69.2, 69.2, 68.9, 68.8, 61.0, 60.8, 60.7, 60.6, 60.5, 60.4, 60.2, 60.1, 51.8, 51.5, 50.5, 50.1, 28.5, 28.5.
^{19}F NMR (565 MHz, Chloroform-*d*; as a mixture of rotamers) δ -73.3, -73.3, -73.3, -73.4, -73.5, -73.5.
HRMS (ESI +) calc. mass for $C_{29}H_{27}O_6N^{79}Br^{35}Cl_3F_3{}^{127}I^{23}Na$ I [M + Na]$^+$ 875.8976; obs. mass for $C_{29}H_{27}O_6N^{79}Br^{35}Cl_3F_3{}^{127}I^{23}Na$ I [M + Na]$^+$ 875.8981.
$[\alpha]^{20}_{D}$: -173.6° (c = 0.110 g/100 mL, EtOAc).
SFC (Regis (S,S) Whelk-O 1 Kromasil column; sample dissolved in *n*-Heptane; 10% cosolvent of 1:1 MeOH:IPA with 0.2% formic acid; 2.5 mL min^{-1} flow-rate; 10 min; UV 210 nm): retention times of 3.04 min (major) and 3.95 min (minor); 98% ee with Rh_2(*S*-PTAD)$_4$.
Determination of d.r.: *N*-Boc deprotection (10 equiv of TFA in CH_2Cl_2 [0.25 M] at 25 °C, 24 hours); observed >20:1 d.r.

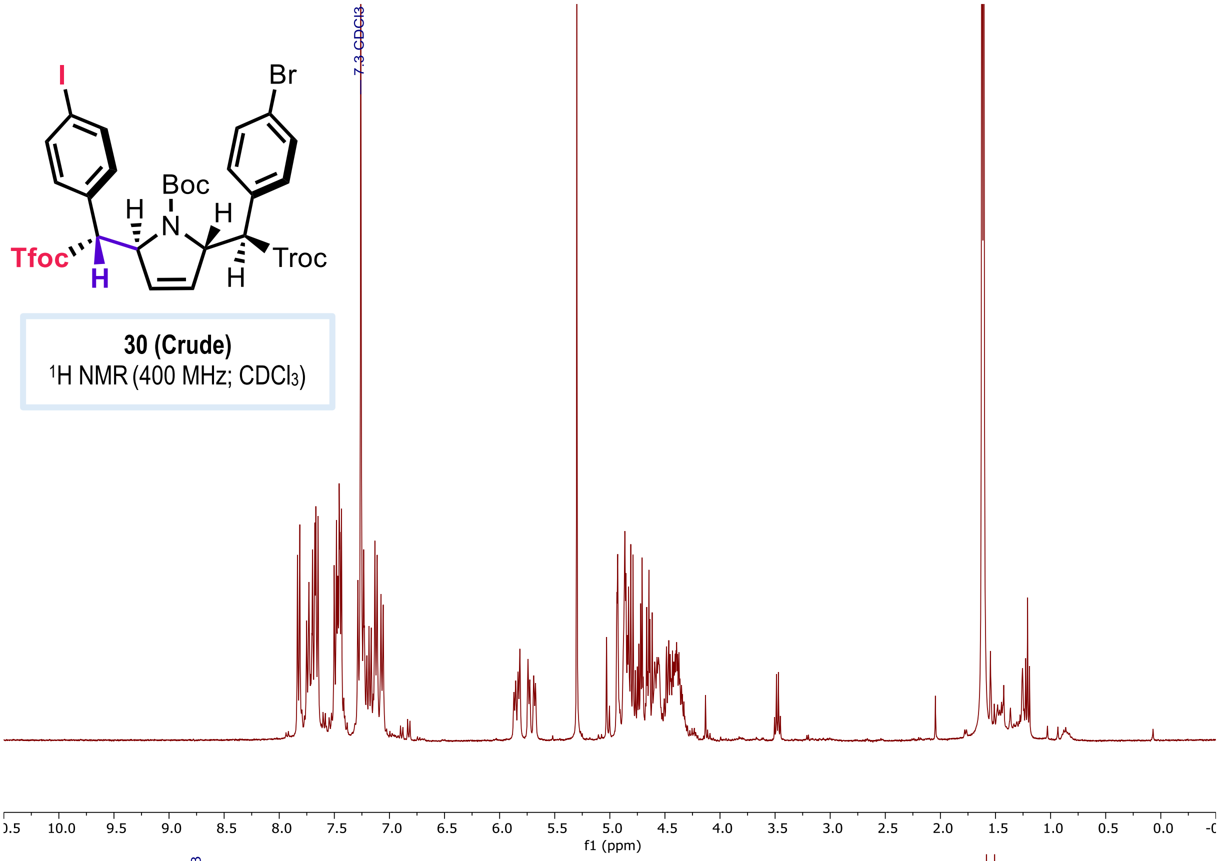
30 (Crude)
1H NMR (400 MHz; CDCl3)
Tfoc
Boc
Troc
f1 (ppm)

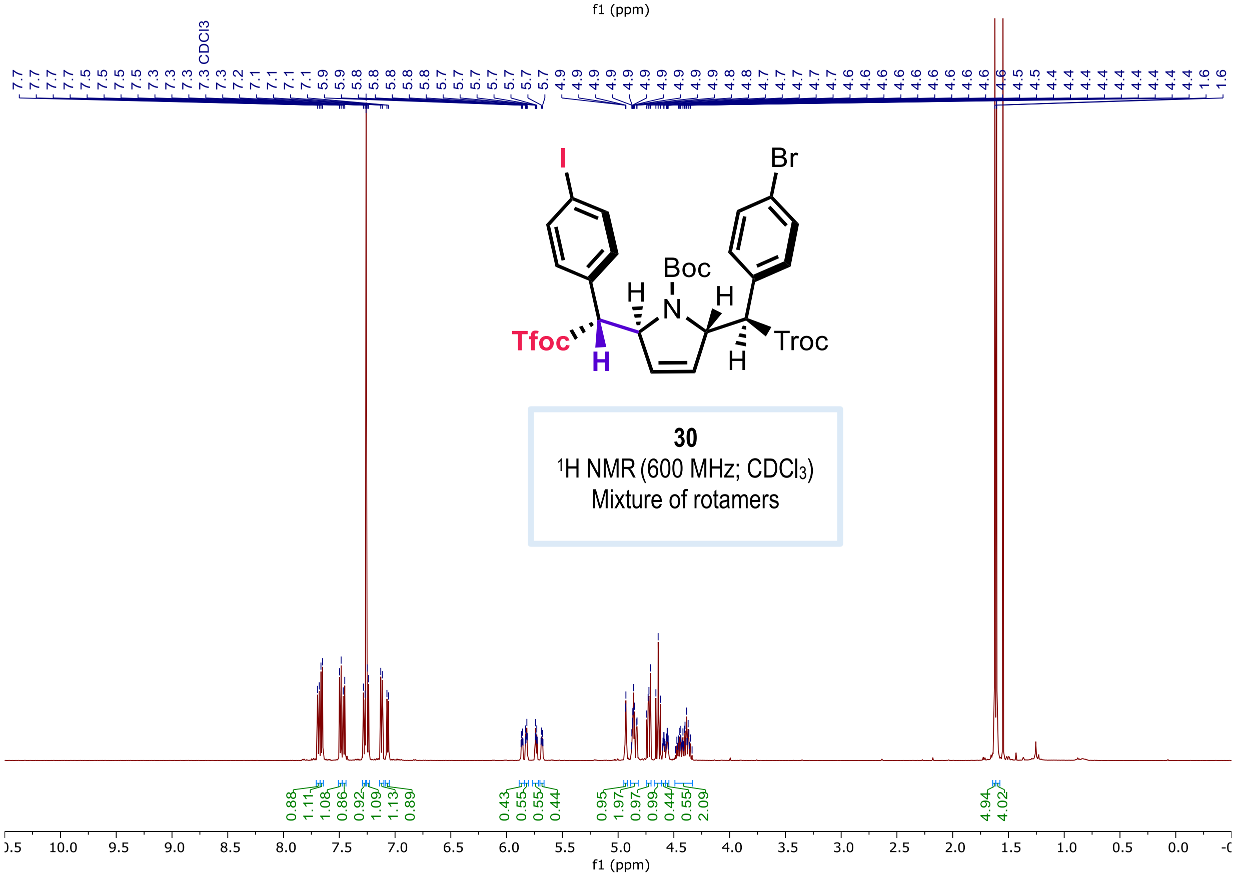
30
1H NMR (600 MHz; CDCl3)
Mixture of rotamers
Tfoc
Boc
Troc
f1 (ppm)

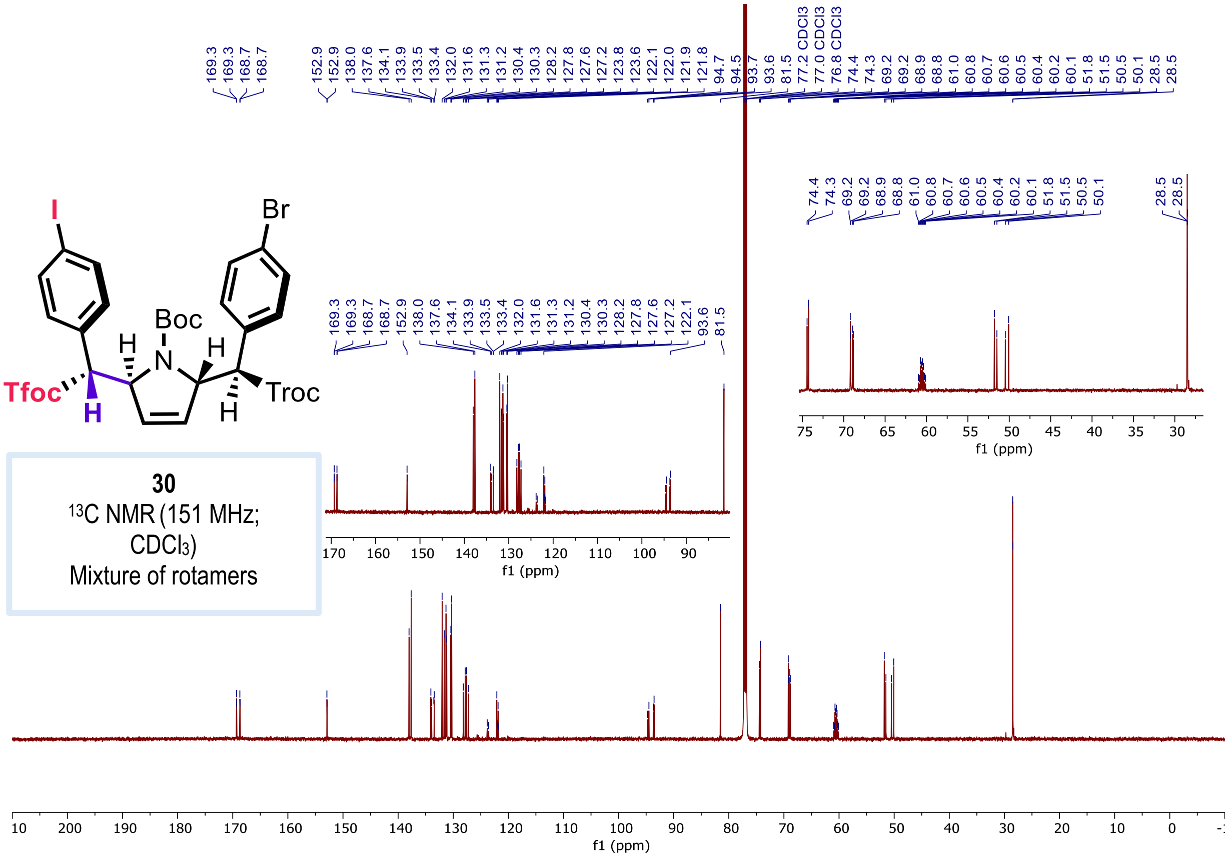

30
^{13}C NMR (151 MHz; $CDCl_3$)
Mixture of rotamers

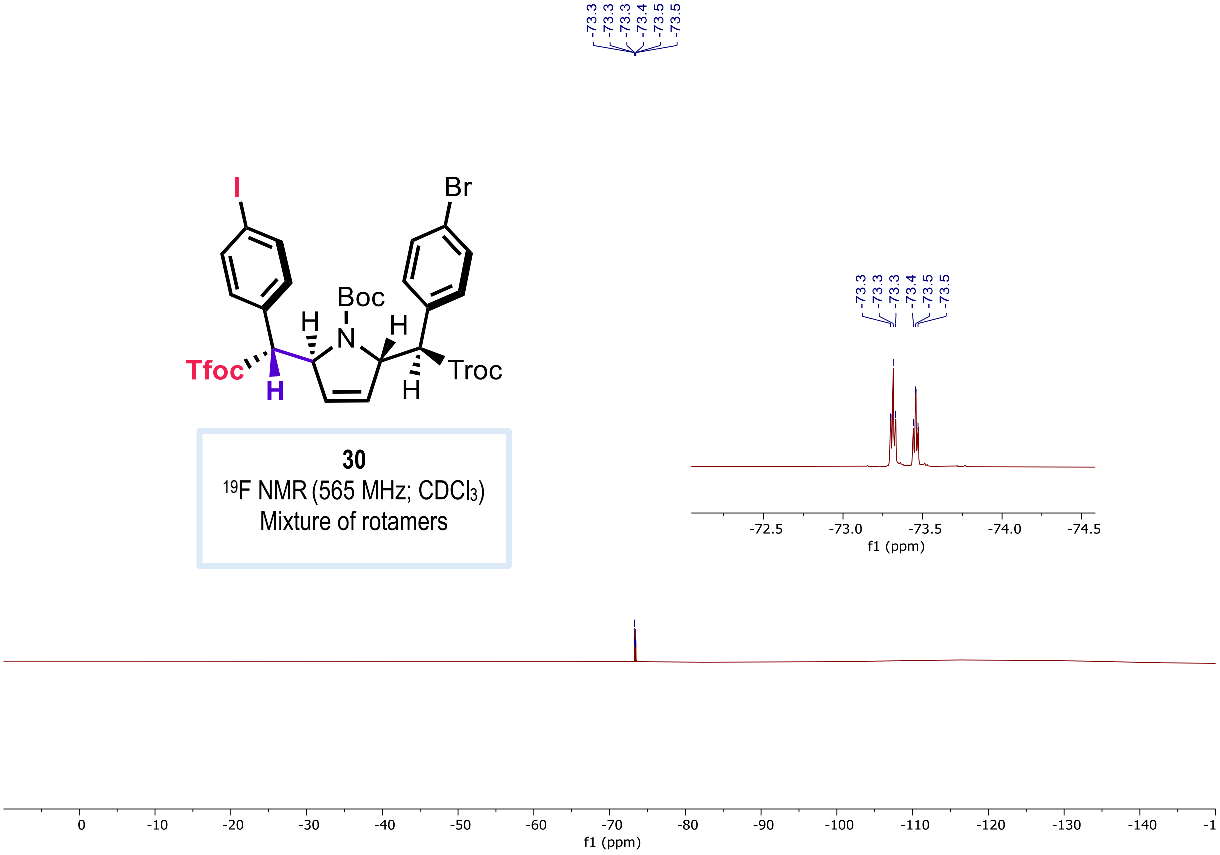

30
^{19}F NMR (565 MHz; $CDCl_3$)
Mixture of rotamers

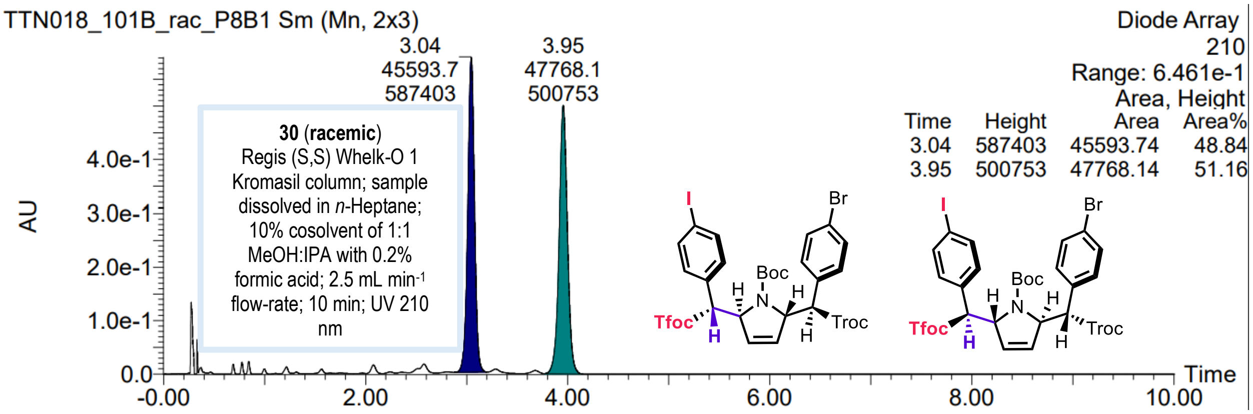
TTN018_101B_rac_P8B1 Sm (Mn, 2x3)
Diode Array
210
Range: 6.461e-1
Area, Height
Time	Height	Area	Area%
3.04	587403	45593.74	48.84
3.95	500753	47768.14	51.16
3.04
45593.7
587403
3.95
47768.1
500753
30 (racemic)
Regis (S,S) Whelk-O 1 Kromasil column; sample dissolved in n-Heptane; 10% cosolvent of 1:1 MeOH:IPA with 0.2% formic acid; 2.5 mL min-1 flow-rate; 10 min; UV 210 nm
AU
Time
I
Br
Boc
Tfoc
Troc
H

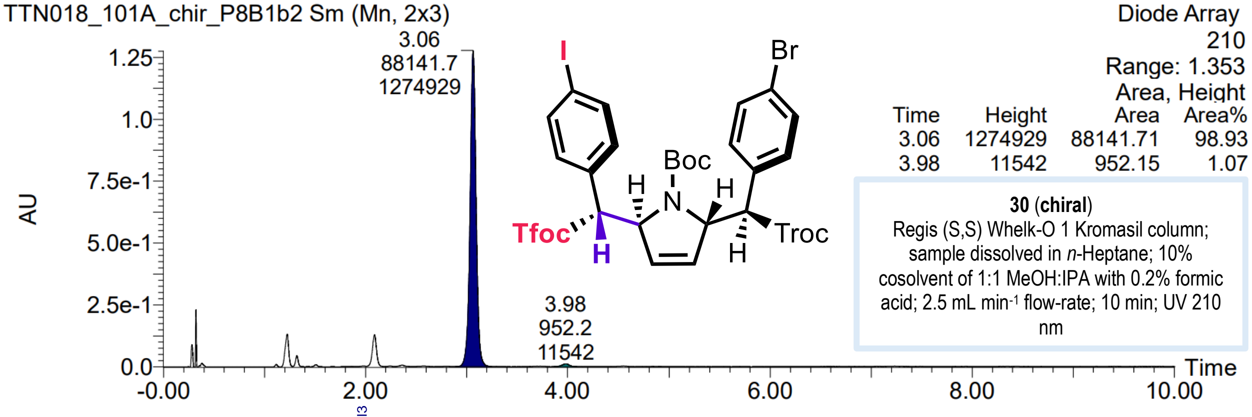
TTN018_101A_chir_P8B1b2 Sm (Mn, 2x3)
Diode Array
210
Range: 1.353
Area, Height
Time	Height	Area	Area%
3.06	1274929	88141.71	98.93
3.98	11542	952.15	1.07
3.06
88141.7
1274929
3.98
952.2
11542
30 (chiral)
Regis (S,S) Whelk-O 1 Kromasil column; sample dissolved in n-Heptane; 10% cosolvent of 1:1 MeOH:IPA with 0.2% formic acid; 2.5 mL min-1 flow-rate; 10 min; UV 210 nm
AU
Time
I
Br
Boc
Tfoc
Troc
H

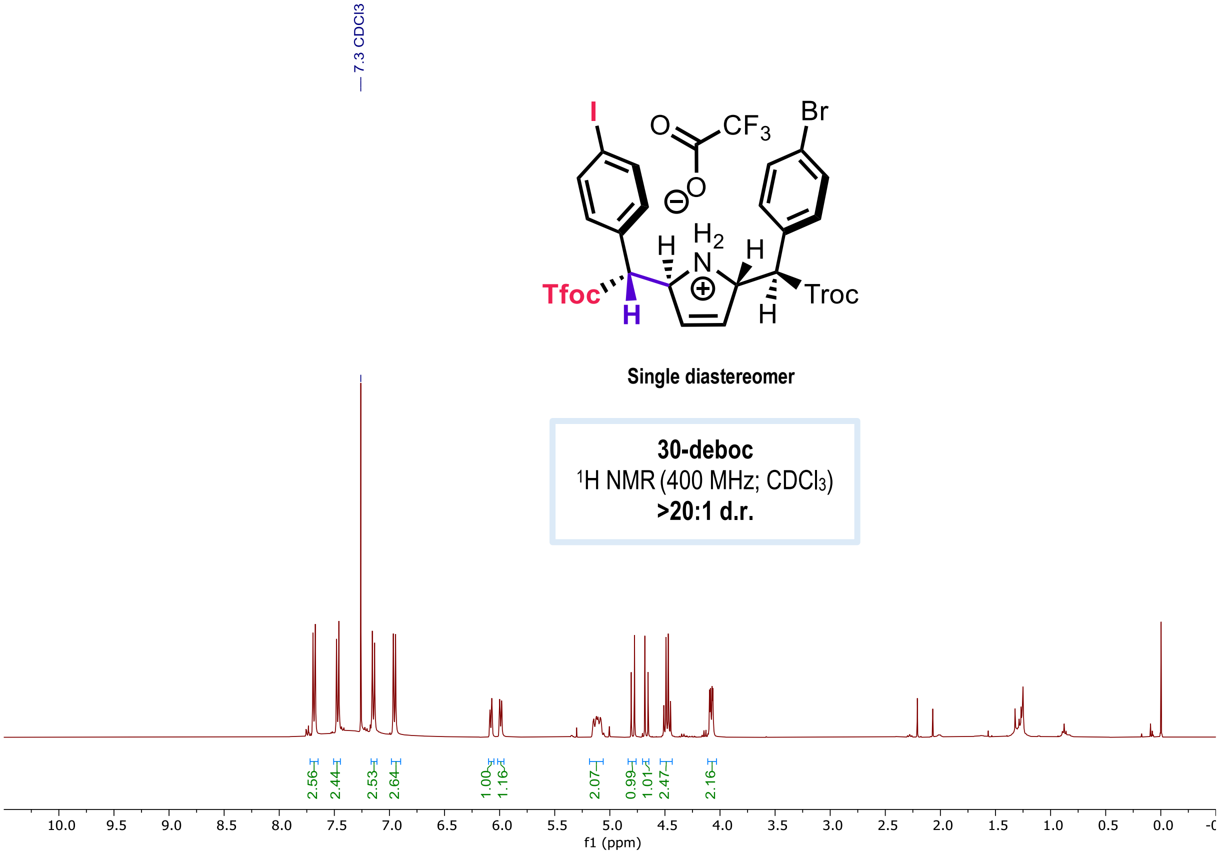
7.3 CDCl3
I
Br
CF3
Tfoc
Troc
Single diastereomer
30-deboc
1H NMR (400 MHz; CDCl3)
>20:1 d.r.
f1 (ppm)

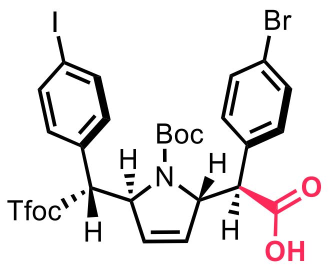

(R)-2-(4-bromophenyl)-2-((2S,5S)-1-(tert-butoxycarbonyl)-5-((R)-1-(4-iodophenyl)-2-oxo-2-(2,2,2-trifluoroethoxy)ethyl)-2,5-dihydro-1H-pyrrol-2-yl)acetic acid (31)

To a vial charged with a stir-bar was added tert-butyl (2S,5S)-2-((R)-1-(4-bromophenyl)-2-oxo-2-(2,2,2-trichloroethoxy)ethyl)-5-((R)-1-(4-iodophenyl)-2-oxo-2-(2,2,2-trifluoroethoxy)ethyl)-2,5-dihydro-1H-pyrrole-1-carboxylate (324 mg, 1 equiv, 379 µmol), zinc dust (<10 µm particle size) (248 mg, 10 equiv, 3.79 mmol) and then acetic acid (22.7 mg, 3.79 mL, 0.1 molar, 1 equiv, 379 µmol). The reaction mixture was stirred at 25 °C for 4 hours. The reaction mixture was then diluted with DI water and EtOAc, the aqueous solution acidified with 1M HCl solution (until pH of ~2 to 1), the organics extracted with EtOAc three times, the organics dried with $MgSO_4$, filtered, and the filtrate concentrated *in vacuo* at 45 °C. The white solids were subjected to SiO_2 flash chromatography using pentane/diethyl ether (Biotage Sfär DLV 25 g column as the running column; 0% 5CV → 2% 5CV → 5% 5CV → 10% 5CV → 15% 5CV → 50% 5CV → 100% 10CV; 150 mL/min flow-rate; 210/254 nm detector; POI elutes at 100% ether). The collected fractions were concentrated *in vacuo* at 45 °C to afford (R)-2-(4-bromophenyl)-2-((2S,5S)-1-(tert-butoxycarbonyl)-5-((R)-1-(4-iodophenyl)-2-oxo-2-(2,2,2-trifluoroethoxy)ethyl)-2,5-dihydro-1H-pyrrol-2-yl)acetic acid (147 mg, 203 µmol, 53.6% yield, 98% ee, >20:1 d.r.) as an off-white foam.

^{1}H NMR (600 MHz, Chloroform-*d*; as a 0.6:0.4 mixture of rotamers) δ 7.69 (Ar–H d, *J* = 8.4 Hz, 0.8H, minor rotamer), 7.64 (Ar–H d, *J* = 8.4 Hz, 1.2H, major rotamer), 7.48 (Ar–H d, *J* = 8.4 Hz, 1.2H, major rotamer), 7.42 (Ar–H d, *J* = 8.4 Hz, 0.8H, minor rotamer), 7.24 (Ar–H d, *J* = 8.4 Hz, 1.2H, major rotamer), 7.19 (Ar–H d, *J* = 8.4 Hz, 0.8H, minor rotamer), 7.12 (Ar–H d, *J* = 8.4 Hz, 1.2H, major rotamer), 7.07 (Ar–H d, *J* = 8.4 Hz, 0.8H, minor rotamer), 5.84 – 5.80 (=C–H m, 0.4H, minor rotamer), 5.69 (=C–H m, 1.6H, mixture of rotamers [0.4H minor rotamer + 1.2H major rotamer = 1.6H]), 4.94 (d, *J* = 4.1 Hz, 0.6H, major rotamer), 4.93 – 4.89 (C–$H_{\alpha\text{-}N}$ brm, 0.4H, minor rotamer), 4.79 (d, *J* = 4.1 Hz, 0.4H, minor rotamer), 4.72 – 4.69 (m, 0.6H, major rotamer), 4.68 (d, *J* = 3.0 Hz, 0.4H, minor rotamer), 4.66 (d, *J* = 4.3 Hz, 0.6H, major rotamer), 4.62 (t, *J* = 4.8 Hz, 0.6H, major rotamer), 4.52 – 4.44 (C–$H_{methylene}$ & C–$H_{\alpha\text{-}N}$ brm, 1H, mixture of rotamers [0.4H minor rotamer + 0.6 major rotamer = 1H]), 4.41 (C–$H_{methylene}$ q, *J* = 8.5 Hz, 1H, mixture of rotamers), 4.34 (C–$H_{methylene}$ dq, *J* = 12.7, 8.5 Hz, 0.6H, major rotamer), 1.59 (–C(CH_3)$_3$ s, 5.4H, major rotamer), 1.56 (–C(CH_3)$_3$ s, 3.6H, minor rotamers).

^{13}C NMR (151 MHz, Chloroform-*d*; as a mixture of rotamers) δ 173.6, 173.0, 169.2, 168.6, 153.6, 153.4, 138.0, 137.6, 134.0, 133.8, 133.6, 133.4, 131.8, 131.5, 131.3, 131.1, 130.7, 130.5, 128.6, 127.7, 127.6, 127.1, 123.8, 122.0, 121.9, 121.8, 93.7, 93.6, 81.9, 77.2, 77.0, 76.8, 69.3, 68.9, 68.7, 68.4, 60.9, 60.6, 60.6, 60.4, 60.4, 60.1, 51.5, 51.3, 51.2, 50.0, 30.3, 29.7, 28.4.

^{19}F NMR (376 MHz, Chloroform-*d*; as a mixture of rotamers) δ -73.3, -73.4, -73.4, -73.5, -73.5, -73.5.

HRMS (ESI +) calc. mass for $C_{27}H_{26}O_6N^{79}BrF_3{}^{127}I^{23}Na$ [M + Na]$^+$ 745.9833; obs. mass for $C_{27}H_{26}O_6N^{79}BrF_3{}^{127}I^{23}Na$ [M + Na]$^+$ 745.9835.

$[\alpha]^{20}_D$: -214.3° (c = 0.080 g/100 mL, EtOAc).

SFC (Regis (S,S) Whelk-O 1 Kromasil column; sample dissolved in MeOH; 7% cosolvent of 1:1 MeOH:IPA with 0.2% formic acid; 2.5 mL min^{-1} flow-rate; 10 min; UV 210 nm): retention times of 6.03 min (major) and 7.73 min (minor); 98% ee.

Determination of d.r.: variable temperature ^{1}H NMR; observed >20:1 d.r.

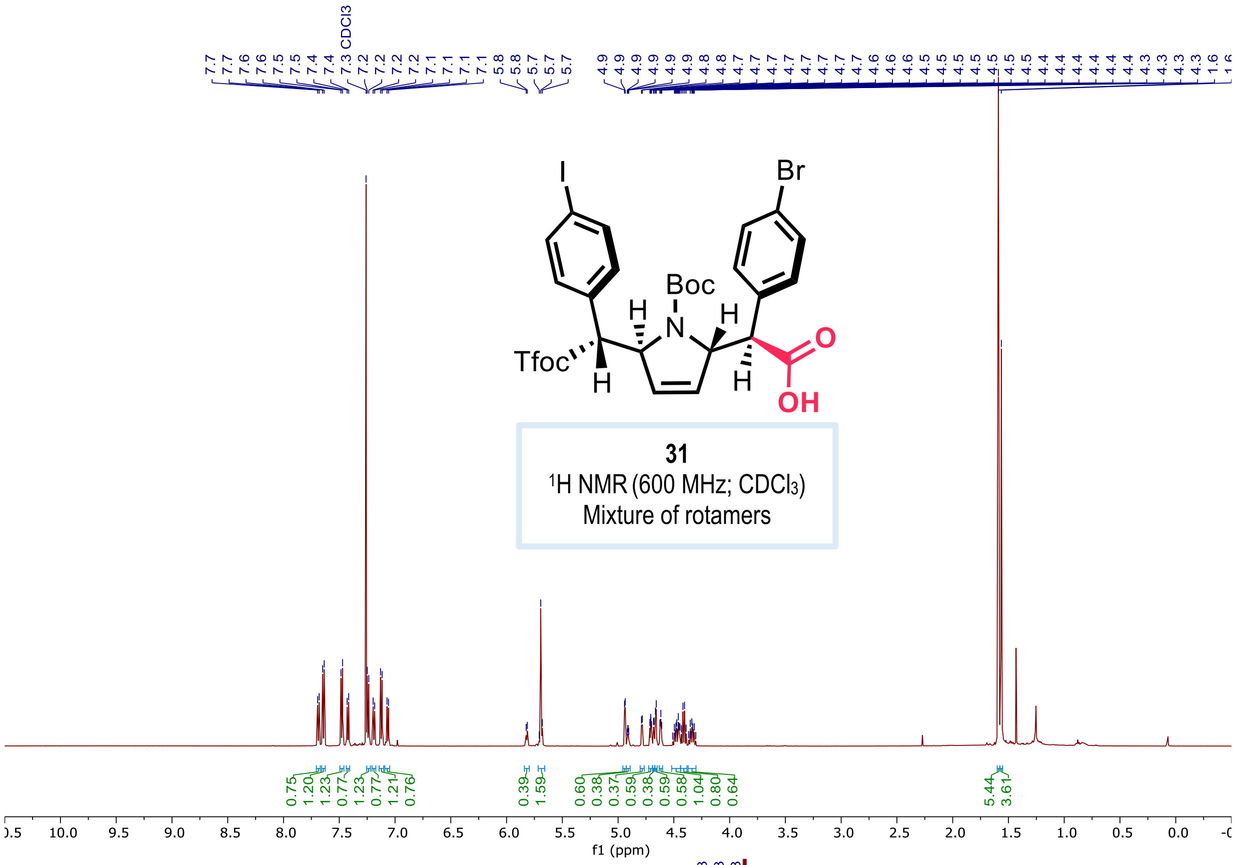

31
^1H NMR (600 MHz; $CDCl_3$)
Mixture of rotamers
f1 (ppm)

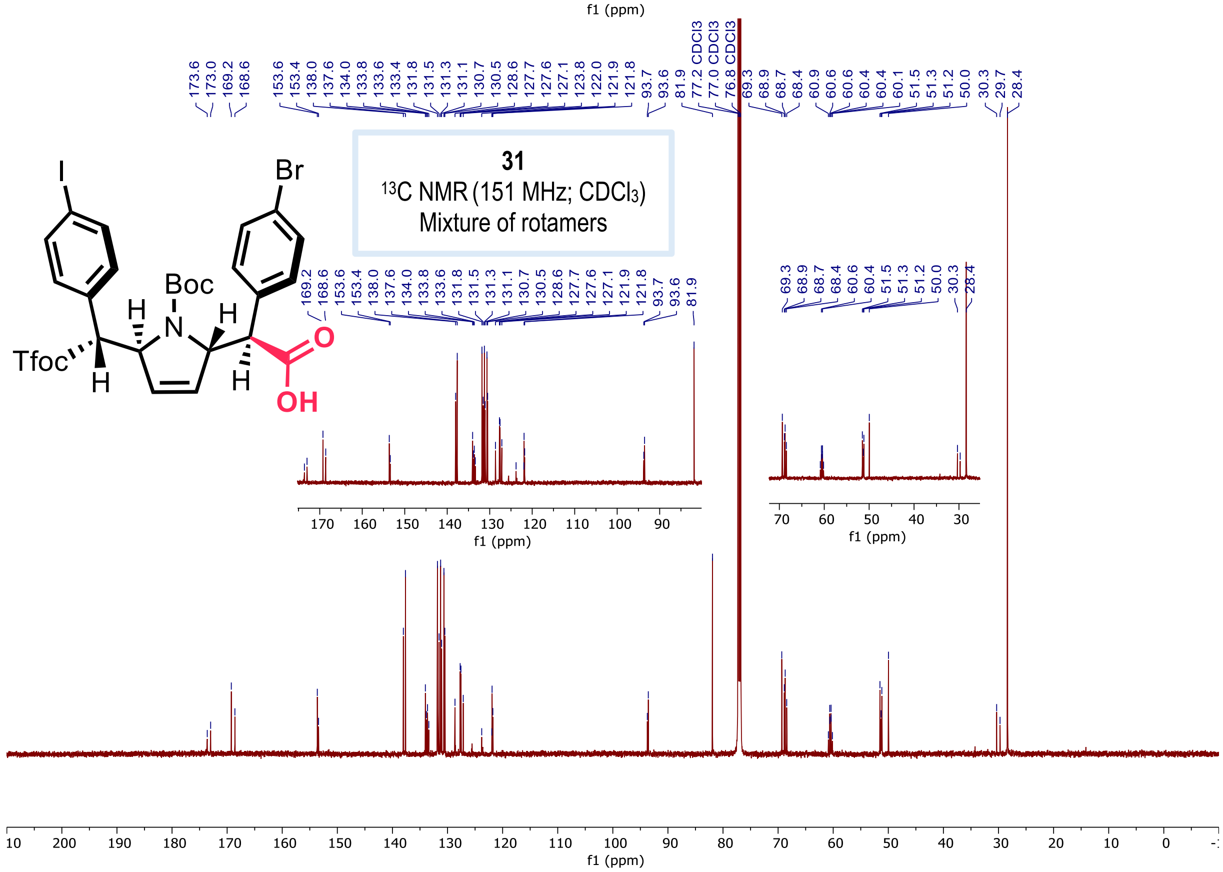

31
^{13}C NMR (151 MHz; $CDCl_3$)
Mixture of rotamers
f1 (ppm)

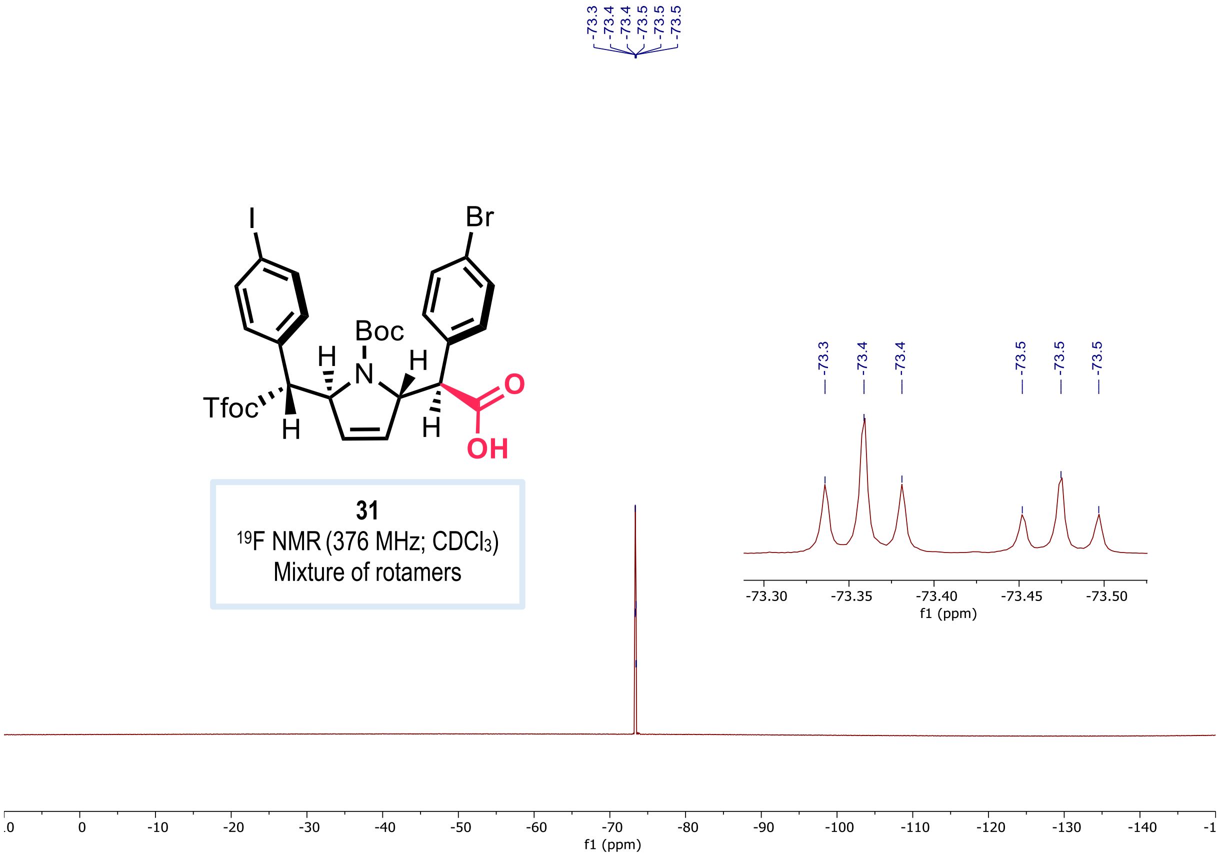
-73.3
-73.4
-73.4
-73.5
-73.5
-73.5
I
Br
Boc
H
N
H
Tfoc
H
H
O
OH
31
19F NMR (376 MHz; CDCl3)
Mixture of rotamers
-73.30
-73.35
-73.40
-73.45
-73.50
f1 (ppm)
0
-10
-20
-30
-40
-50
-60
-70
-80
-90
-100
-110
-120
-130
-140
f1 (ppm)

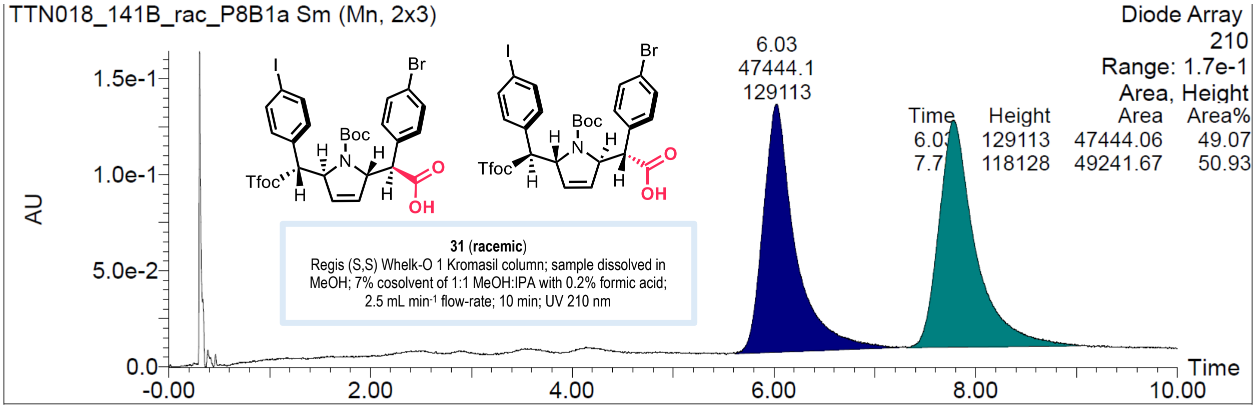
TTN018_141B_rac_P8B1a Sm (Mn, 2x3)
Diode Array
210
Range: 1.7e-1
Area, Height
6.03
47444.1
129113
Time Height Area Area%
6.03 129113 47444.06 49.07
7.7 118128 49241.67 50.93
31 (racemic)
Regis (S,S) Whelk-O 1 Kromasil column; sample dissolved in MeOH; 7% cosolvent of 1:1 MeOH:IPA with 0.2% formic acid; 2.5 mL min-1 flow-rate; 10 min; UV 210 nm
AU
1.5e-1
1.0e-1
5.0e-2
0.0
-0.00
2.00
4.00
6.00
8.00
10.00
Time

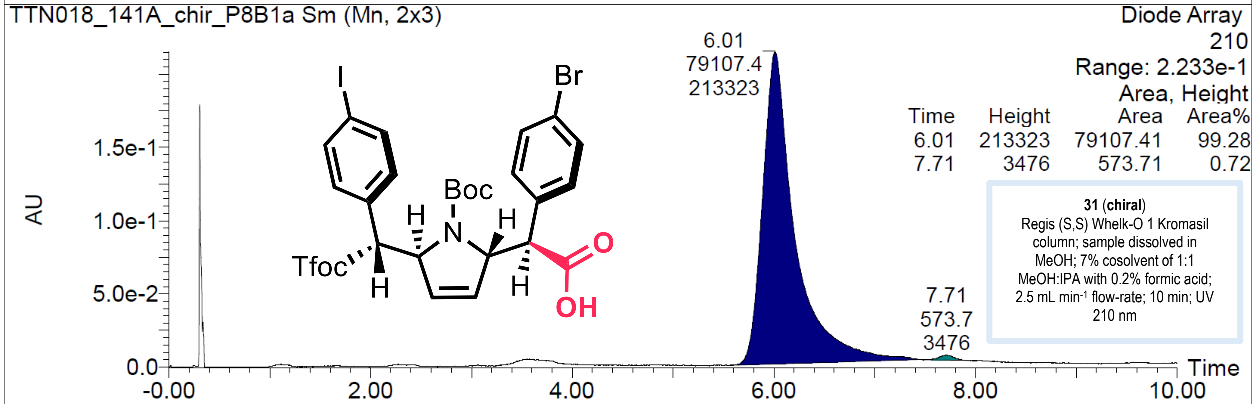
TTN018_141A_chir_P8B1a Sm (Mn, 2x3)
Diode Array
210
Range: 2.233e-1
Area, Height
6.01
79107.4
213323
Time Height Area Area%
6.01 213323 79107.41 99.28
7.71 3476 573.71 0.72
7.71
573.7
3476
31 (chiral)
Regis (S,S) Whelk-O 1 Kromasil column; sample dissolved in MeOH; 7% cosolvent of 1:1 MeOH:IPA with 0.2% formic acid; 2.5 mL min-1 flow-rate; 10 min; UV 210 nm
AU
1.5e-1
1.0e-1
5.0e-2
0.0
-0.00
2.00
4.00
6.00
8.00
10.00
Time

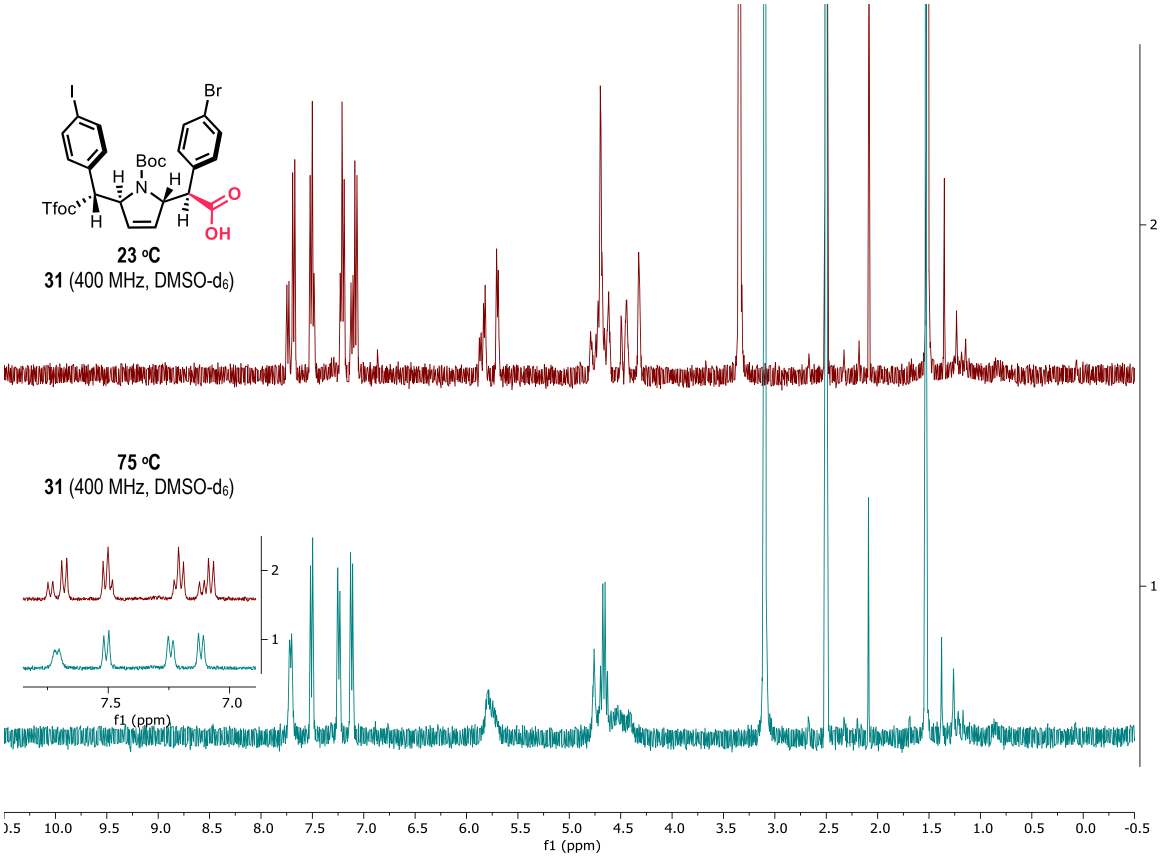

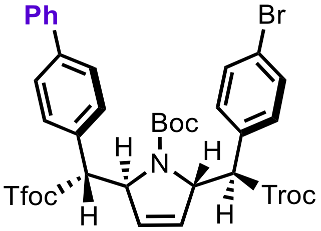

tert-butyl (2S,5S)-2-((R)-1-([1,1'-biphenyl]-4-yl)-2-oxo-2-(2,2,2-trifluoroethoxy)ethyl)-5-((R)-1-(4-bromophenyl)-2-oxo-2-(2,2,2-trichloroethoxy)ethyl)-2,5-dihydro-1H-pyrrole-1-carboxylate (32)

To a flame-dried 8 mL vial charged with a stir-bar was added benzene boronic acid (40.2 mg, 1.1 equiv, 330 µmol), tert-butyl (2S,5S)-2-((R)-1-(4-bromophenyl)-2-oxo-2-(2,2,2-trichloroethoxy)ethyl)-5-((R)-1-(4-iodophenyl)-2-oxo-2-(2,2,2-trifluoroethoxy)ethyl)-2,5-dihydro-1H-pyrrole-1-carboxylate (257 mg, 1 equiv, .300 mmol), cesium carbonate (215 mg, 2.2 equiv, 660 µmol), and then tetrakis(triphenylphosphine)palladium(0) (34.7 mg, 0.10 equiv, 30.0 µmol). To the solid material was added toluene (1.36 mL) and DI water (136 µL). The vessel was sparged with nitrogen for at least 15 minutes at room temperature. The reaction mixture was then capped with a nitrogen balloon and allowed to stir at 100 °C for 2 to 4 hours [**Note:** *the reaction sometimes finishes at 2 hours and sometimes at 4 hours so monitor carefully with ^{1}H NMR analyses of crude aliquots*]. After the elapsed time, the reaction mixture was cooled to room temperature and then diluted with diethyl ether and saturated bicarbonate solution, the organics extracted with ether three times, the combined organics dried with $MgSO_4$, filtered, and the filtrate was concentrated *in vacuo* at 45 °C. The crude material was dry-loaded onto SiO_2 and subjected to SiO_2 flash chromatography (Biotage Sfär DLV 50 g column as the running-column and Biotage Sfär DLV 25 g for the dry-load; 100 mL/min flow-rate; 210/230 nm detector; 0% 7CV → 1% 7CV → 3% 7CV → 5% 7CV → 7% 7CV → 10% 15CV → 13% 7CV → 16% 7CV; POI elutes at 10% ether). The collected fractions

were concentrated *in vacuo* at 45 °C to afford tert-butyl (2S,5S)-2-((R)-1-([1,1'-biphenyl]-4-yl)-2-oxo-2-(2,2,2-trifluoroethoxy)ethyl)-5-((R)-1-(4-bromophenyl)-2-oxo-2-(2,2,2-trichloroethoxy)ethyl)-2,5-dihydro-1H-pyrrole-1-carboxylate (134 mg, 166 µmol, 55.4% yield, 98% ee, >20:1 d.r.) as a white amorphous material.

^{1}H NMR (600 MHz, Chloroform-*d*; as a 0.5:0.5 mixture of rotamers) δ 7.60 – 7.55 (Ar–H m, 4H), 7.49 (Ar–H d, *J* = 8.4 Hz, 1H), 7.48 – 7.42 (Ar–H m, 4H), 7.39 (Ar–H d, *J* = 8.4 Hz), 7.37 – 7.35 (Ar–H m, 1H), 7.30 (Ar–H d, *J* = 8.4 Hz, 1H), 7.26 (Ar–H d, *J* = 8.4 Hz, 1H), 5.92 (=C–H dt, *J* = 6.6, 2.0 Hz, 0.5H), 5.88 (=C–H dt, *J* = 6.6, 2.0 Hz, 0.5H), 5.79 (=C–H dt, *J* = 6.6, 2.0 Hz, 0.5H), 5.76 (=C–H dt, *J* = 6.6, 2.0 Hz, 0.5H), 5.12 ($C–H_{benzylic}$ d, *J* = 3.6 Hz, 0.5H), 4.96 ($C–H_{benzylic}$ d, *J* = 4.4 Hz, 0.5H), 4.95 ($C–H_{benzylic}$ d, *J* = 3.6 Hz, 0.5H), 4.93 – 4.89 ($C–H_{α\text{-}N}$ m, 1H), 4.88 ($C–H_{benzylic}$ d, *J* = 4.4 Hz, 0.5H), 4.76 – 4.72 ($C–H_{methylene}$ overlapping d's, *J* = 12.1 Hz each d, 1H), 4.69 – 4.61 ($C–H_{methylene}$ & $C–H_{α\text{-}N}$ overlapping d's & m, *J* = 12.1 Hz each d, 2H), 4.53 – 4.33 ($C–H_{methylene}$ m, 2H), 1.64 ($–C(CH_3)_3$ s, 4.5H), 1.64 ($–C(CH_3)_3$ s, 4.5H).
^{13}C NMR (Chloroform-*d*; as a mixture of rotamers) δ 169.7, 169.4, 169.1, 168.8, 153.0, 152.9, 140.9, 140.6, 140.5, 140.3, 133.6, 133.6, 133.5, 133.2, 132.0, 131.6, 131.2, 130.3, 129.7, 128.9, 128.9, 128.9, 128.8, 128.2, 127.8, 127., 127., 127.6, 127.5, 127.3, 127.1, 127.1, 122.0, 121.8, 121.8, 94.8, 94.5, 81.4, 81.4, 74.4, 74.2, 69.4, 69.2, 69.1, 69.0, 60.6, 60.5, 60.4, 60.3, 51.8, 51.6, 50.5, 50.1, 29.7, 28.5, 28.5.
^{19}F NMR (565 MHz, Chloroform-*d*; as a mixture of rotamers) δ -73.4, -73.4, -73.4, -73.5, -73.5, -73.5.
HRMS (APCI -) calc. mass for $C_{35}H_{32}O_6N^{79}Br^{35}Cl_4F_3$ [M + Cl]$^-$ 838.0125; obs. mass for $C_{35}H_{32}O_6N^{79}Br^{35}Cl_4F_3$ [M + Cl]$^-$ 838.0109.
$[\alpha]^{20}_D$: -184.7 (c = 0.390 g/100 mL, EtOAc).
SFC (Regis (S,S) Whelk-O 1 Kromasil column; sample dissolved in *n*-Heptane; 10% cosolvent of 1:1 MeOH:IPA with 0.2% formic acid; 2.5 mL min^{-1} flow-rate; 10 min; UV 210 nm): retention times of 4.52 min (major) and 6.35 min (minor); 98% ee.
Determination of d.r.: variable temperature ^{1}H NMR; observed >20:1 d.r.

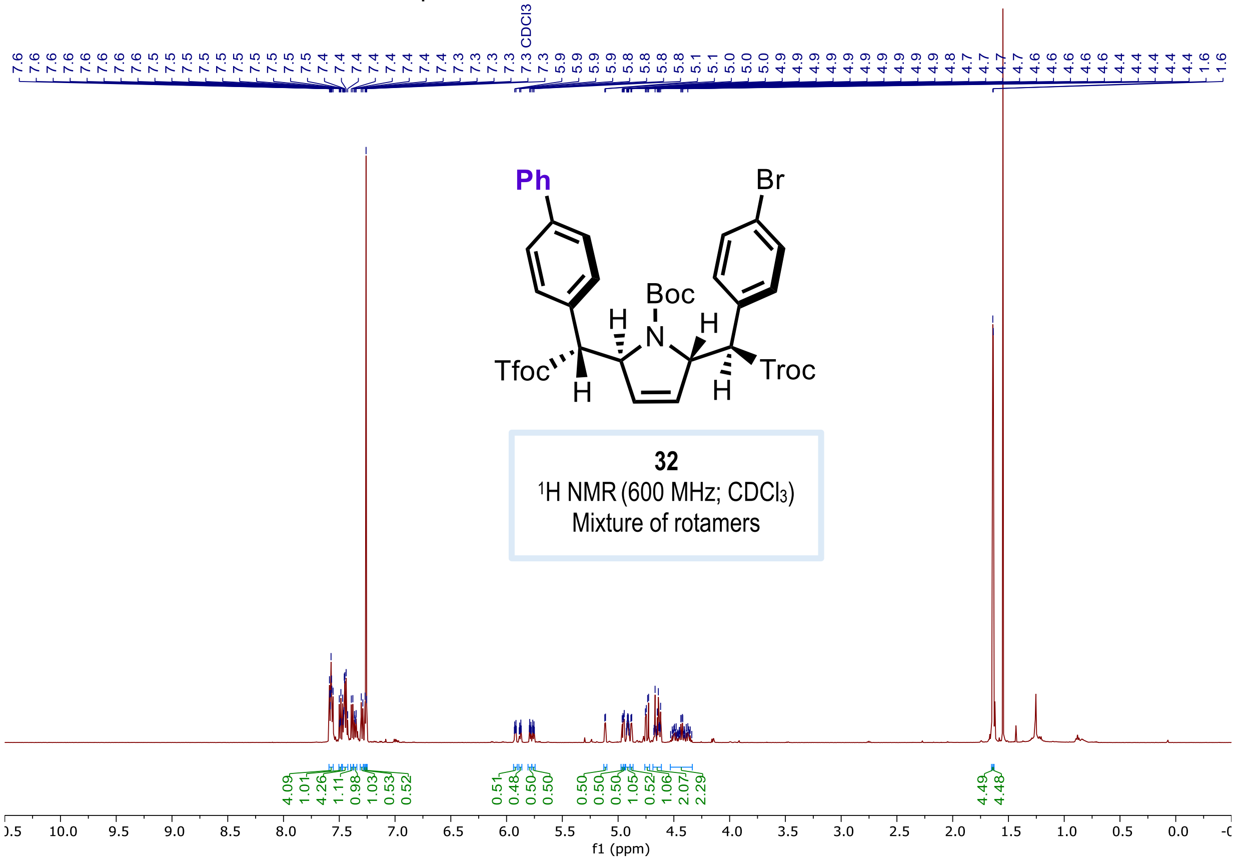

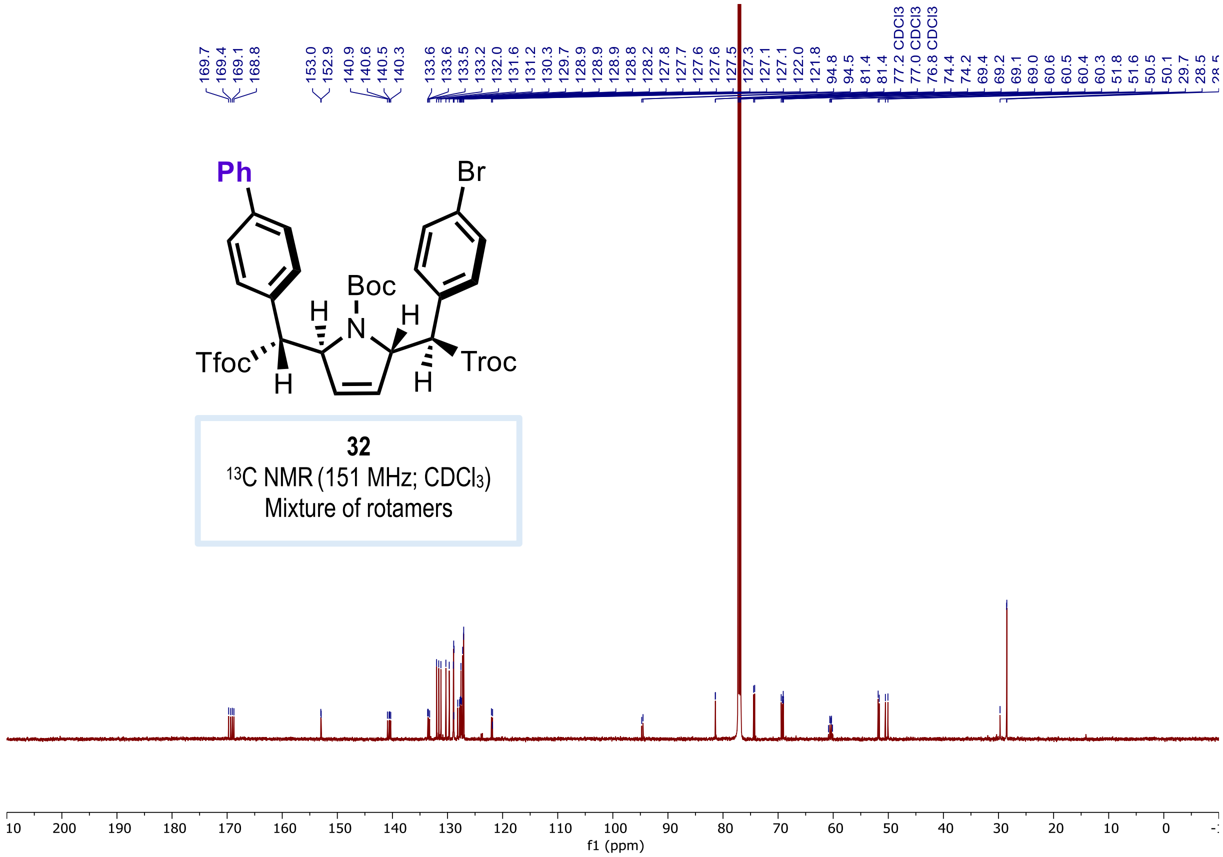
32
^{13}C NMR (151 MHz; CDCl$_3$)
Mixture of rotamers
f1 (ppm)

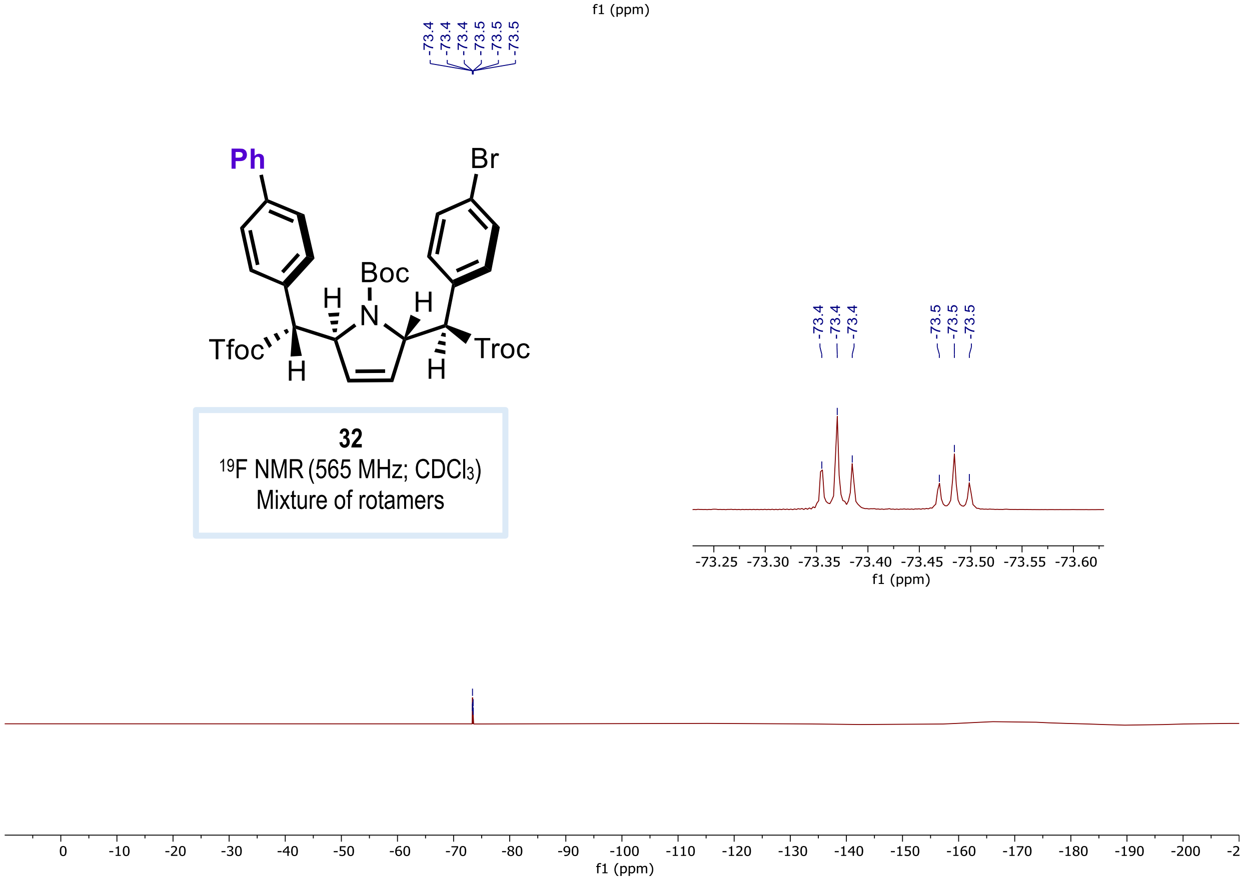
32
^{19}F NMR (565 MHz; CDCl$_3$)
Mixture of rotamers
f1 (ppm)

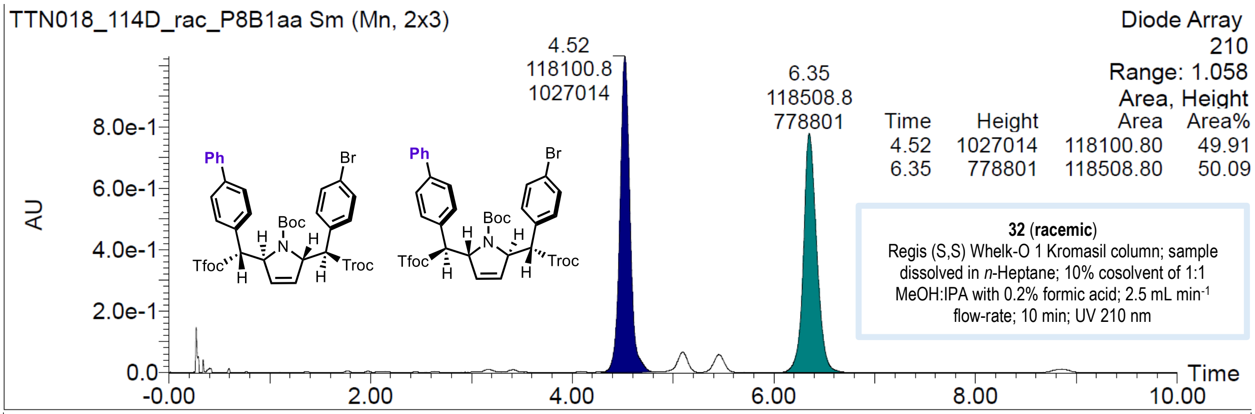

TTN018_114D_rac_P8B1aa Sm (Mn, 2x3)
Diode Array
210
Range: 1.058
Area, Height
4.52
118100.8
1027014
6.35
118508.8
778801
Time Height Area Area%
4.52 1027014 118100.80 49.91
6.35 778801 118508.80 50.09
32 (racemic)
Regis (S,S) Whelk-O 1 Kromasil column; sample dissolved in n-Heptane; 10% cosolvent of 1:1 MeOH:IPA with 0.2% formic acid; 2.5 mL min-1 flow-rate; 10 min; UV 210 nm
AU
Time
Ph
Br
Boc
Tfoc
Troc

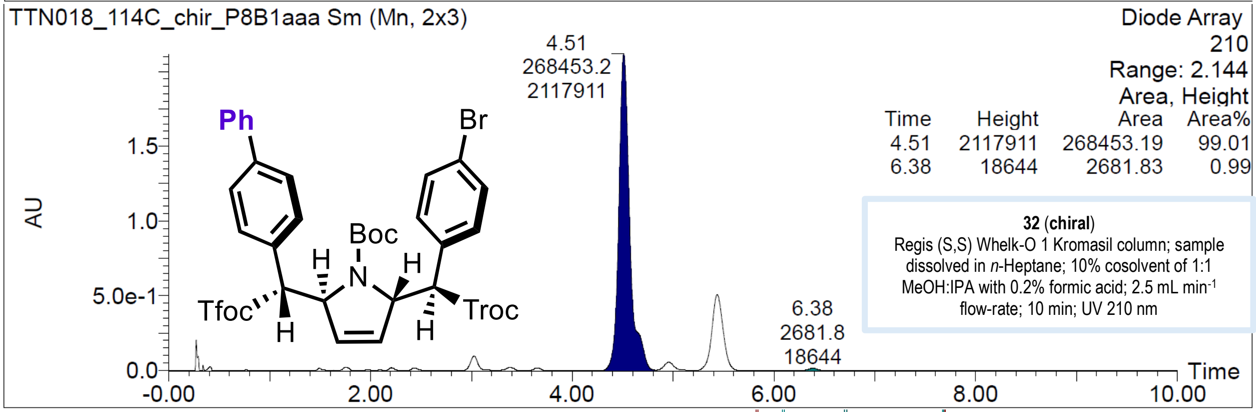

TTN018_114C_chir_P8B1aaa Sm (Mn, 2x3)
Diode Array
210
Range: 2.144
Area, Height
4.51
268453.2
2117911
6.38
2681.8
18644
Time Height Area Area%
4.51 2117911 268453.19 99.01
6.38 18644 2681.83 0.99
32 (chiral)
Regis (S,S) Whelk-O 1 Kromasil column; sample dissolved in n-Heptane; 10% cosolvent of 1:1 MeOH:IPA with 0.2% formic acid; 2.5 mL min-1 flow-rate; 10 min; UV 210 nm
AU
Time
Ph
Br
Boc
Tfoc
Troc

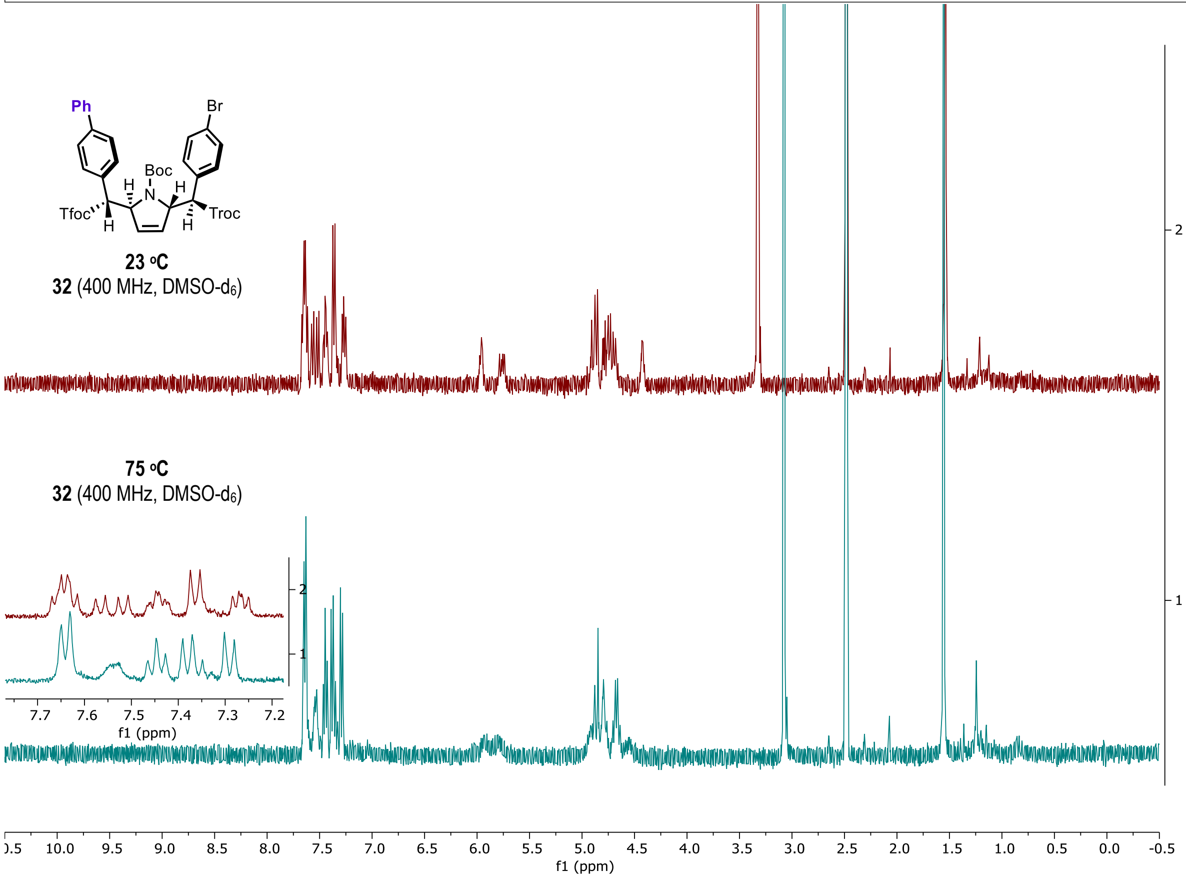

Ph
Br
Boc
Tfoc
Troc
23 °C
32 (400 MHz, DMSO-d6)
75 °C
32 (400 MHz, DMSO-d6)
f1 (ppm)

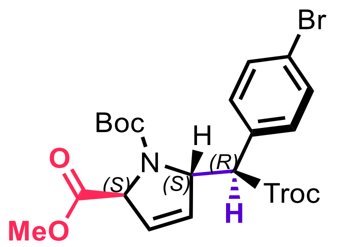

1-(tert-butyl) 2-methyl (2S,5S)-5-((R)-1-(4-bromophenyl)-2-oxo-2-(2,2,2-trichloroethoxy)ethyl)-2,5-dihydro-1H-pyrrole-1,2-dicarboxylate (33)

To an 8 mL dram vial charged with a stir-bar and 0.5 g of activated 4Å MS was added 1-(tert-butyl) 2-methyl (S)-2,5-dihydro-1H-pyrrole-1,2-dicarboxylate (114 mg, 1 equiv, 0.500 mmol) and was backfilled with nitrogen three times. CH_2Cl_2 (0.5 mL) followed by a stock solution of Rh_2(*S*-PTAD)$_4$ (390 µg, 500 µL CH_2Cl_2, 0.0005 molar, 0.0005 equiv, 0.250 µmol) was added to the vessel. The reaction vessel containing the olefin and catalyst was stirred at room temperature and for at least 10 minutes. After the elapsed time, to the reaction vessel was added 2,2,2-trichloroethyl 2-(4-bromophenyl)-2-diazoacetate (559 mg, 1.00 mL CH_2Cl_2, 1.50 molar, 3.0 equiv, 1.50 mmol) via a multi-channel syringe pump [*syringe pump settings -- 1 mL syringe, 0.333 mL/hr, diameter 4.71 mm, vol 1.0 mL; Air-Tite or SilverPoint 22Gx4" long hypodermic needle was used*] over 3 hours. After complete addition and an elapsed time of 3 hours, the resulting solution was reacted for an additional 17 hours and then subsequently, filtered through a celite plug and concentrated *in vacuo* at 45 °C. The crude material was dry-loaded onto SiO_2 and subjected to SiO_2 flash chromatography (Biotage Sfär DLV 50 g column as the running-column and Biotage Sfär DLV 50 g column for the dry-load; 100 mL/min flow-rate; 210/230 nm detector; 0% 6CV → 3% 6CV → 5% 6CV → 7% 6CV → 10% 6CV → 13% 6CV → 16% 6CV → 20% 6CV; POI elutes at 13% to 16% ether). The collected fractions were concentrated *in vacuo* at 45 °C to afford 1-(tert-butyl) 2-methyl (2S,5S)-5-((R)-1-(4-bromophenyl)-2-oxo-2-(2,2,2-trichloroethoxy)ethyl)-2,5-dihydro-1H-pyrrole-1,2-dicarboxylate (262 mg, 458 µmol, 91.7% yield, 98% ee, >20:1 d.r.) as an amorphous white material.

With $Rh_2(OAc)_4$ instead of Rh_2(*S*-PTAD)$_4$ – 190 mg, 332 µmol, 66.5% yield, >20:1 d.r.

^{1}H NMR (400 MHz, Chloroform-*d*; as a 0.75:0.25 mixture of rotamers) δ 7.51 (Ar–H d, *J* = 8.5 Hz, 0.5H, minor rotamer), 7.48 (Ar–H d, *J* = 8.5 Hz, 1.5H, major rotamer), 7.33 (Ar–H d, *J* = 8.5 Hz, 1.5H, major rotamer), 7.29 (Ar–H d, *J* = 8.5 Hz, 0.5H, minor rotamer), 5.93 (=C–H dt, *J* = 6.4, 1.9 Hz, 0.75H, major rotamer), 5.88 (=C–H dt, *J* = 6.4, 1.9 Hz, 0.25H, minor rotamer), 5.82 (=C–H dt, *J* = 6.4, 1.9 Hz, 0.25H, minor rotamer), 5.73 (=C–H dt, *J* = 6.4, 1.9 Hz, 0.75H, major rotamer), 5.13 – 5.04 (C–$H_{\alpha\text{-}N}$ m, 0.75H, major rotamer), 4.99 – 4.90 (C–$H_{\alpha\text{-}N}$ m, 0.5H, minor rotamers), 4.88 – 4.84 (C–$H_{benzylic}$ & C–$H_{methylene}$ d's, *J* = 4.0 Hz & 12.1 Hz respectively, 1.5H, major rotamers), 4.85 – 4.81 (C–$H_{\alpha\text{-}N}$ m, 0.75H, major rotamer), 4.81 – 4.75 (C–$H_{benzylic}$ & C–$H_{methylene}$ m, 0.5H, minor rotamers), 4.68 (C–$H_{methylene}$ d, *J* = 12.1 Hz, 0.25H, minor rotamer), 4.64 (C–$H_{methylene}$ d, *J* = 12.1 Hz, 0.75H, major rotamer), 3.72 (–CH_3 s, 0.75H, minor rotamer), 3.70 (–CH_3 s, 2.25H, major rotamer), 1.58 (–$C(CH_3)_3$ s, 2.25H, minor rotamer), 1.43 (–$C(CH_3)_3$ s, 6.75H, major rotamer).

^{13}C NMR (151 MHz, Chloroform-*d*; as a mixture of rotamers) δ 170.5, 170.2, 169.2, 168.8, 153.3, 153.2, 133.4, 132.1, 131.8, 130.8, 130.2, 130.1, 129.7, 128.3, 126.0, 125.8, 122.2, 122.0, 94.8, 94.5, 81.6, 81.1, 74.3, 74.3, 68.7, 68.4, 68.0, 52.5, 52.4, 52.3, 51.3, 28.5, 28.2.

HRMS (ESI +) calc. mass for $C_{21}H_{24}O_6N^{79}Br^{35}Cl_3$ $[M + H]^+$ 569.9847; obs. mass for $C_{21}H_{24}O_6N^{79}Br^{35}Cl_3$ $[M + H]^+$ 569.9851.

$[\alpha]^{20}_D$: -206.8° (c = 0.305 g/100 mL, EtOAc).

SFC (Regis (S,S) Whelk-O 1 Kromasil column; sample dissolved in 80:20 *n*-Heptane:IPA; 5% cosolvent of 1:1 MeOH:IPA with 0.2% formic acid; 2.5 mL min^{-1} flow-rate; 10 min; UV 210 nm): retention times of 3.06 min (major) and 3.38 min (minor); 98% ee.

Determination of d.r.: variable temperature ^{1}H NMR; observed >20:1 d.r.

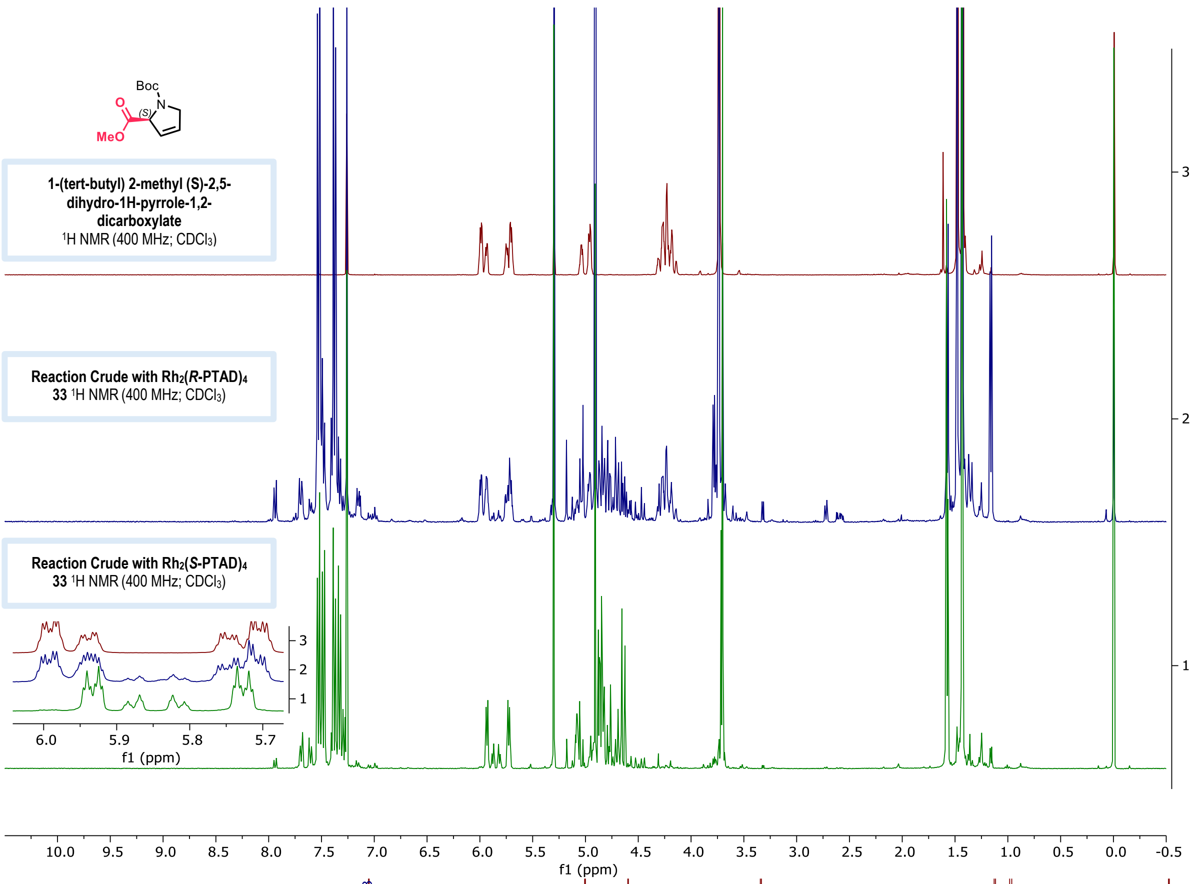
Boc
(S)
MeO
O
1-(tert-butyl) 2-methyl (S)-2,5-dihydro-1H-pyrrole-1,2-dicarboxylate
¹H NMR (400 MHz; CDCl₃)
Reaction Crude with Rh₂(R-PTAD)₄
33 ¹H NMR (400 MHz; CDCl₃)
Reaction Crude with Rh₂(S-PTAD)₄
33 ¹H NMR (400 MHz; CDCl₃)
f1 (ppm)
f1 (ppm)

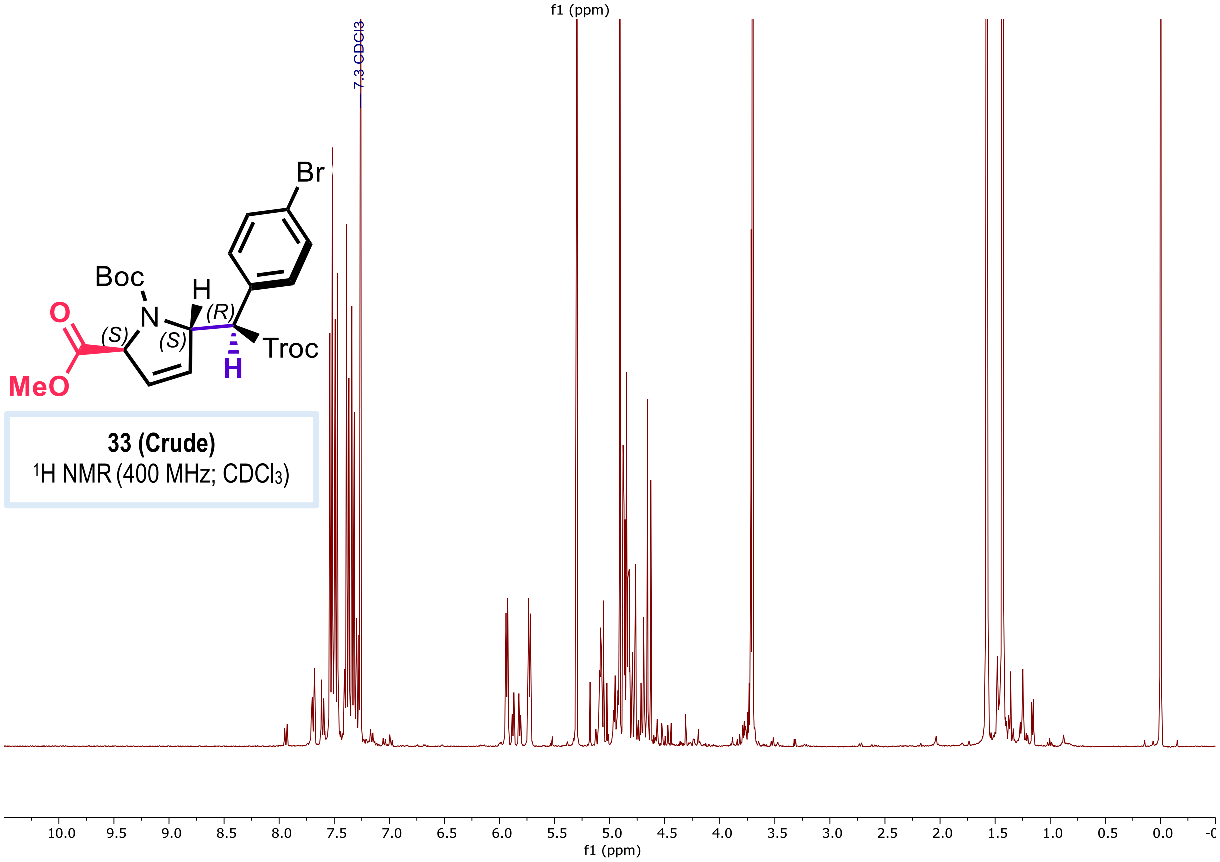
7.3 CDCl3
Br
Boc
H
(R)
N
(S)
(S)
Troc
H
MeO
O
33 (Crude)
¹H NMR (400 MHz; CDCl₃)
f1 (ppm)

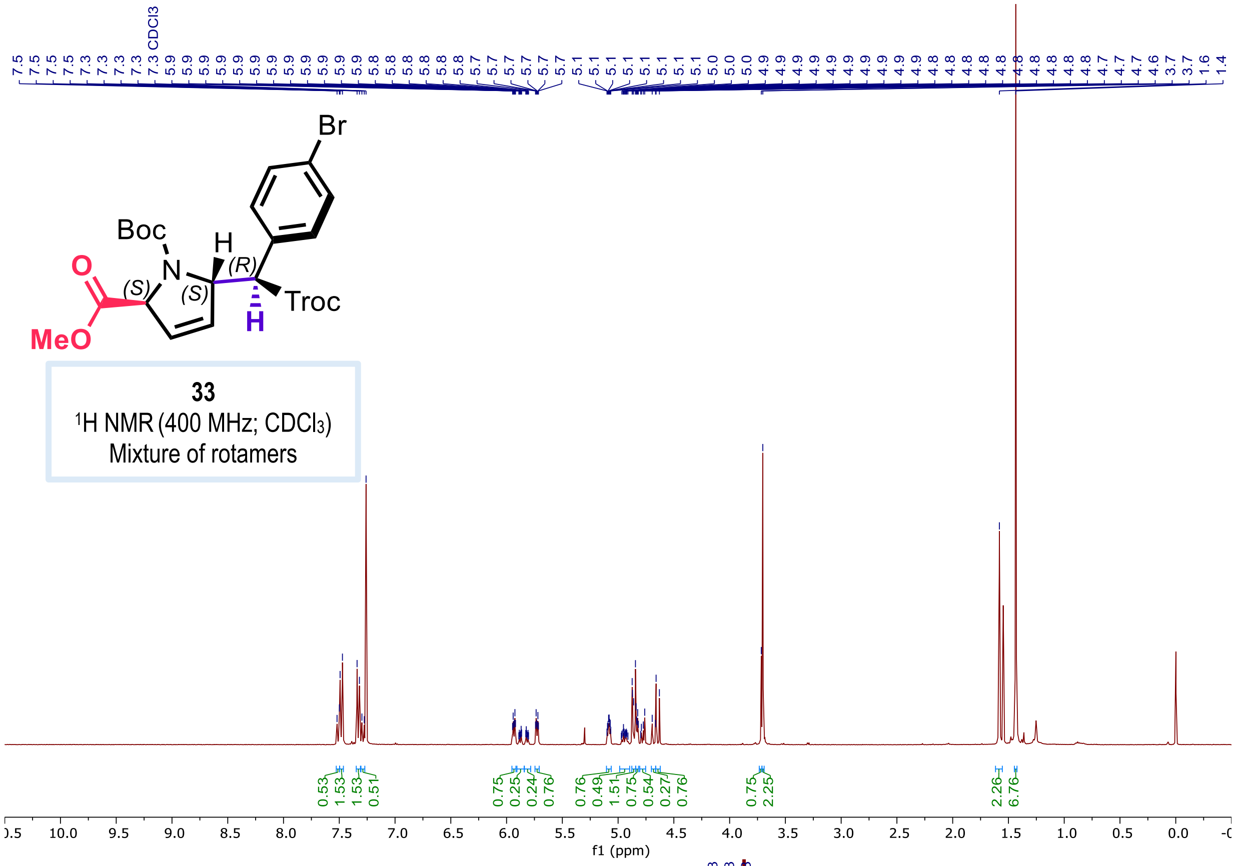
33
^{1}H NMR (400 MHz; CDCl$_3$)
Mixture of rotamers
Boc
MeO
Troc
Br
f1 (ppm)

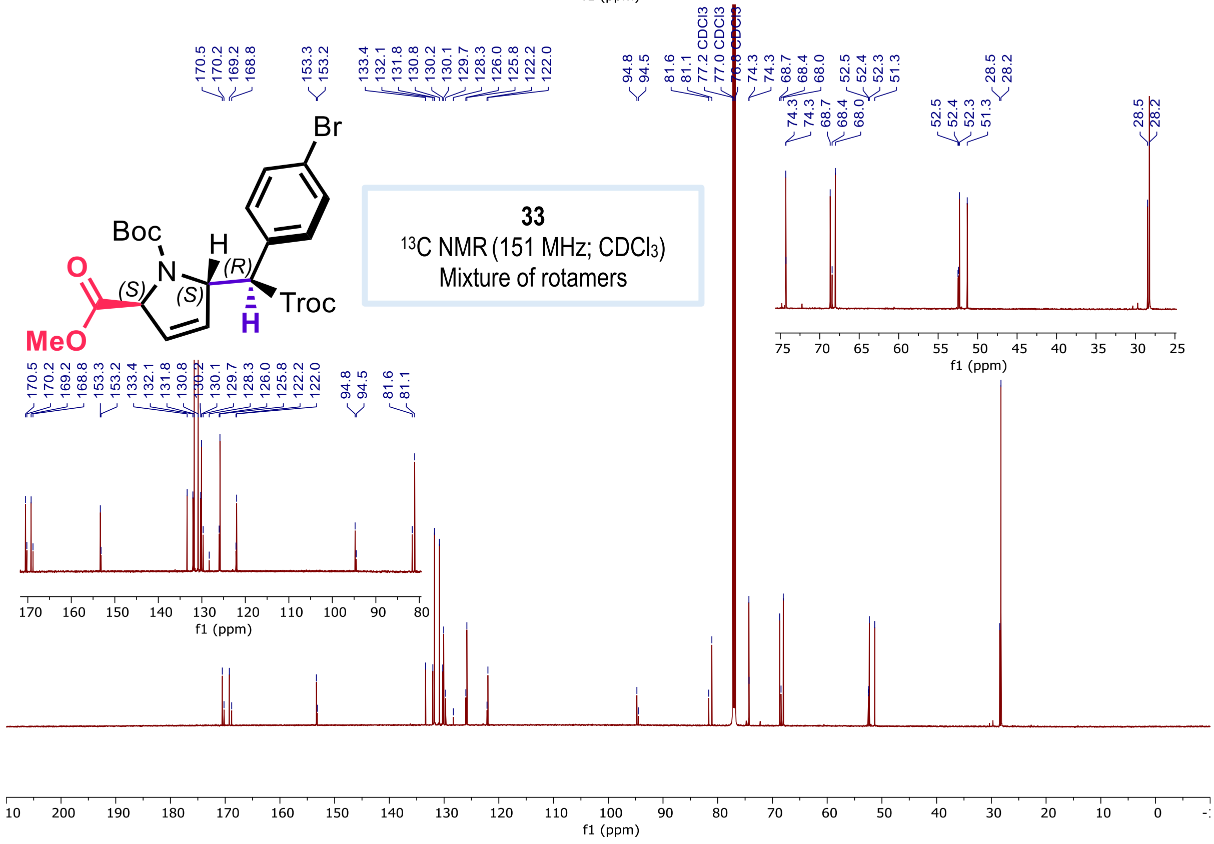
33
^{13}C NMR (151 MHz; CDCl$_3$)
Mixture of rotamers
Boc
MeO
Troc
Br
f1 (ppm)

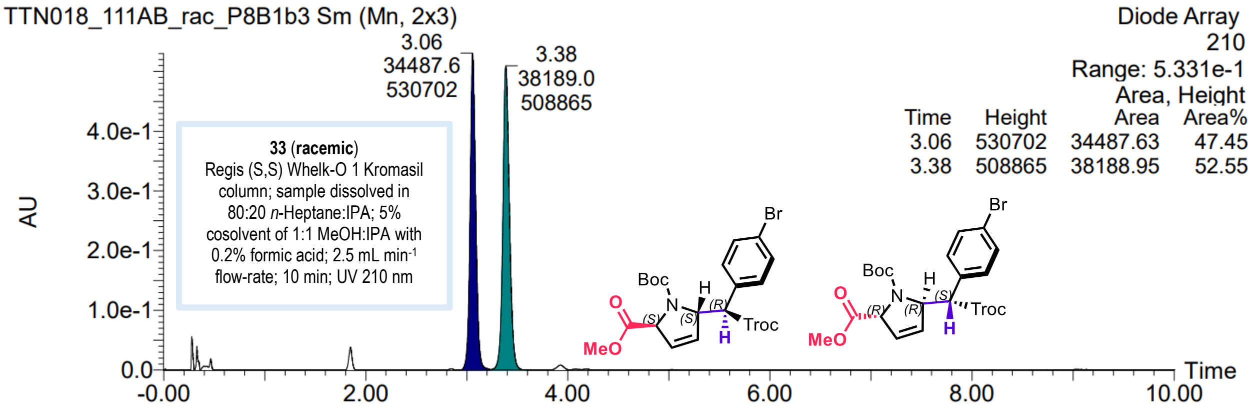
TTN018_111AB_rac_P8B1b3 Sm (Mn, 2x3)
Diode Array
210
Range: 5.331e-1
Area, Height
33 (racemic)
Regis (S,S) Whelk-O 1 Kromasil column; sample dissolved in 80:20 n-Heptane:IPA; 5% cosolvent of 1:1 MeOH:IPA with 0.2% formic acid; 2.5 mL min-1 flow-rate; 10 min; UV 210 nm
3.06
34487.6
530702
3.38
38189.0
508865
Time Height Area Area%
3.06 530702 34487.63 47.45
3.38 508865 38188.95 52.55
AU
Time
Boc
MeO
Troc
Br

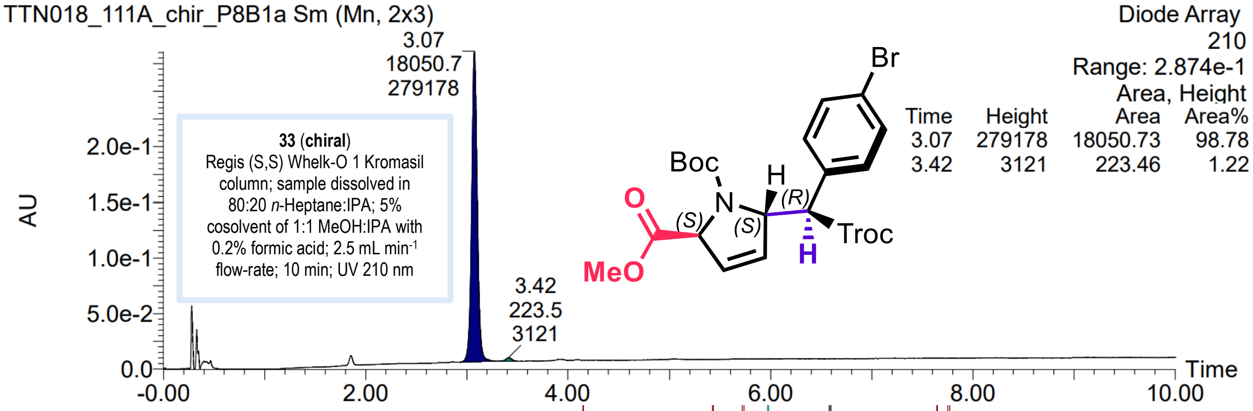
TTN018_111A_chir_P8B1a Sm (Mn, 2x3)
Diode Array
210
Range: 2.874e-1
Area, Height
33 (chiral)
Regis (S,S) Whelk-O 1 Kromasil column; sample dissolved in 80:20 n-Heptane:IPA; 5% cosolvent of 1:1 MeOH:IPA with 0.2% formic acid; 2.5 mL min-1 flow-rate; 10 min; UV 210 nm
3.07
18050.7
279178
3.42
223.5
3121
Time Height Area Area%
3.07 279178 18050.73 98.78
3.42 3121 223.46 1.22
AU
Time
Boc
MeO
Troc
Br

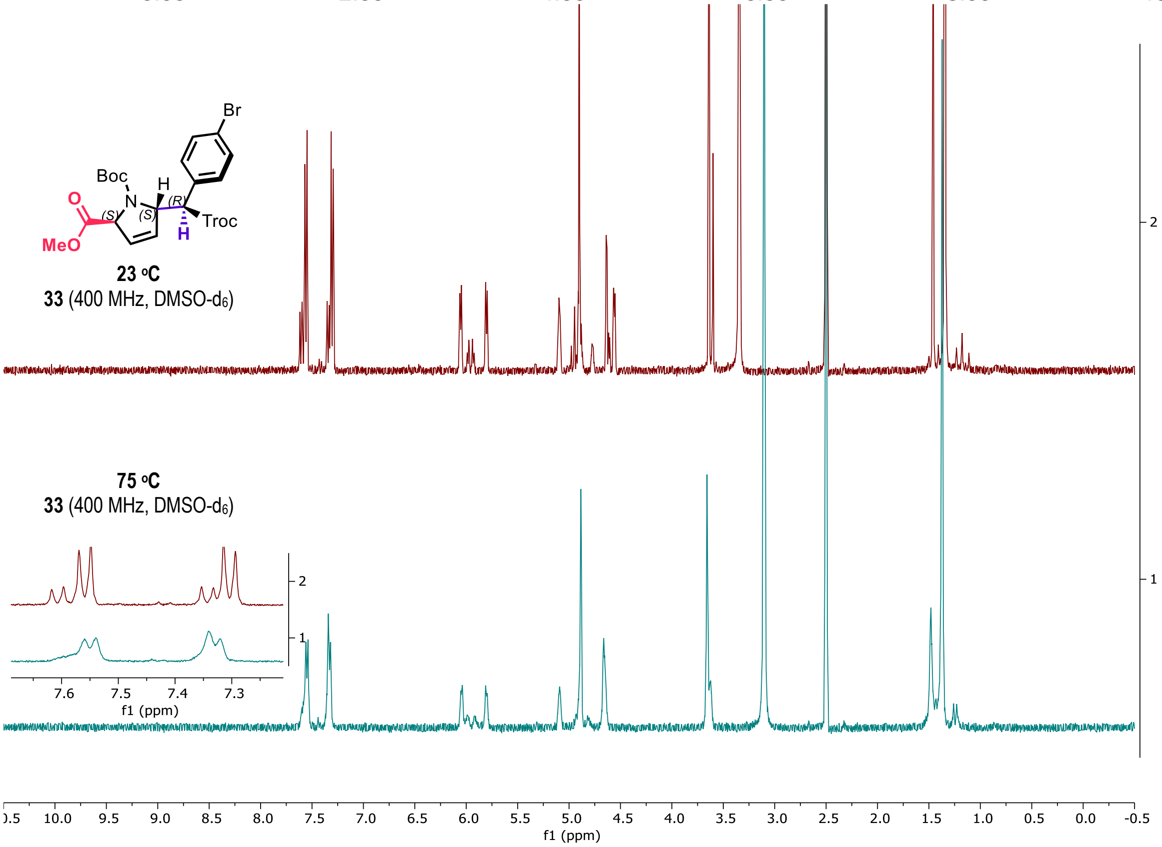
23 °C
33 (400 MHz, DMSO-d6)
75 °C
33 (400 MHz, DMSO-d6)
f1 (ppm)
Boc
MeO
Troc
Br

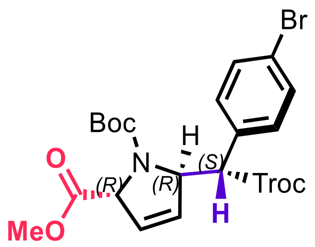

1-(tert-butyl) 2-methyl (2R,5R)-5-((S)-1-(4-bromophenyl)-2-oxo-2-(2,2,2-trichloroethoxy)ethyl)-2,5-dihydro-1H-pyrrole-1,2-dicarboxylate (34)

To an 8 mL dram vial charged with a stir-bar and 0.5 g of activated 4 Å MS was added 1-(tert-butyl) 2-methyl (R)-2,5-dihydro-1H-pyrrole-1,2-dicarboxylate (114 mg, 1 equiv, 0.500 mmol) and was backfilled with nitrogen three times. CH_2Cl_2 (0.5 mL) followed by stock solution of Rh_2(*R*-PTAD)$_4$ (390 µg, 500 µL CH_2Cl_2, 0.0005 molar, 0.0005 equiv, 0.250 µmol) was added to the vessel. The reaction vessel containing olefin and catalyst was stirred at room temperature for at least 10 minutes. After the elapsed time, to the reaction vessel was added 2,2,2-trichloroethyl 2-(4-bromophenyl)-2-diazoacetate (559 mg, 1.00 mL CH_2Cl_2, 1.50 molar, 3.0 equiv, 1.50 mmol) via a multi-channel syringe pump [*syringe pump settings - 1 mL syringe, 0.333 mL/hr, diameter 4.71 mm, vol 1.0 mL; Air-Tite or SilverPoint 22Gx4" long hypodermic needle was used*] over 3 hours. After complete addition and an elapsed time of 3 hours, the resulting solution was reacted for an additional 17 hours and then subsequently, filtered through a celite plug and concentrated *in vacuo* at 45 °C. The crude material was dry-loaded onto SiO_2 and subjected to SiO_2 flash chromatography using pentane/diethyl ether (Biotage Sfär DLV 50 g column as the running-column and Biotage Sfär DLV 50 g column for the dry-load; 100 mL/min flow-rate; 210/230 nm detector; 0% 6CV → 3% 6CV → 5% 6CV → 7% 6CV → 10% 6CV → 13% 6CV → 16% 6CV → 20% 6CV; POI elutes at 13% to 16% ether). The collected fractions were concentrated *in vacuo* at 45 °C to afford 1-(tert-butyl) 2-methyl (2R,5R)-5-((S)-1-(4-bromophenyl)-2-oxo-2-(2,2,2-trichloroethoxy)ethyl)-2,5-dihydro-1H-pyrrole-1,2-dicarboxylate (274 mg, 479 µmol, 95.9% yield, >99% ee, >20:1 d.r.) as a pale yellow oil.

^{1}H NMR (400 MHz, Chloroform-*d*; as a 0.75:0.25 mixture of rotamers) δ 7.51 (Ar–H d, *J* = 8.5 Hz, 0.5H, minor rotamer), 7.48 (Ar–H d, *J* = 8.5 Hz, 1.5H, major rotamer), 7.33 (Ar–H d, *J* = 8.5 Hz, 1.5H, major rotamer), 7.29 (Ar–H d, *J* = 8.5 Hz, 0.5H, minor rotamer), 5.93 (=C–H dt, *J* = 6.4, 2.0 Hz, 0.75H, major rotamer), 5.88 (=C–H dt, *J* = 6.4, 2.0 Hz, 0.25H, minor rotamer), 5.82 (=C–H dt, *J* = 6.4, 2.0 Hz, 0.25H, minor rotamer), 5.73 (=C–H dt, *J* = 6.4, 2.0 Hz, 0.75H, major rotamer), 5.12 – 5.04 (C–$H_{\alpha\text{-}N}$ m, 0.75H, major rotamer), 4.98 – 4.90 (C–$H_{\alpha\text{-}N}$ m, 0.5H, minor rotamers), 4.89 – 4.81 (C–$H_{benzylic}$ & C–$H_{methylene}$ d's, *J* = 3.9 Hz & 12.0 Hz respectively, 1.5H, major rotamers), 4.84 – 4.81 (C–$H_{\alpha\text{-}N}$ m, 0.75H, major rotamers), 4.80 – 4.74 (C–$H_{benzylic}$ & C–$H_{methylene}$ m, 0.5H, minor rotamers), 4.68 (C–$H_{methylene}$ d, *J* = 12.0 Hz, 0.25H, minor rotamers), 4.64 (C–$H_{methylene}$ d, *J* = 12.0 Hz, 0.75H, major rotamers), 3.72 (–CH_3 s, 0.75H, minor rotamer), 3.70 (–CH_3 s, 2.25H, major rotamer), 1.58 (–C(CH_3)$_3$ s, 2.25H, minor rotamer), 1.43 (–C(CH_3)$_3$ s, 6.75H, major rotamer).
^{13}C NMR (151 MHz, Chloroform-*d*; as a mixture of rotamers) δ 170.5, 170.2, 169.2, 168.8, 153.3, 153.2, 133.4, 132.1, 131.8, 130.8, 130.2, 130.1, 129.7, 128.3, 126.0, 125.8, 122.2, 122.0, 94.8, 94.5, 81.6, 81.1, 74.3, 74.3, 68.7, 68.4, 68.0, 52.5, 52.4, 52.3, 51.3, 28.5, 28.2.
HRMS (APCI -) calc. mass for $C_{21}H_{22}O_6N^{79}Br^{35}Cl_3$ [M - H]$^-$ 567.9702; obs. mass for $C_{21}H_{22}O_6N^{79}Br^{35}Cl_3$ [M - H]$^-$ 567.9683.
$[\alpha]^{20}_D$: +208.2° (c = 0.425 g/100 mL, EtOAc).
SFC (Regis (S,S) Whelk-O 1 Kromasil column; sample dissolved in 80:20 *n*-Heptane:IPA; 5% cosolvent of 1:1 MeOH:IPA with 0.2% formic acid; 2.5 mL min^{-1} flow-rate; 10 min; UV 210 nm): retention times of 3.06 min (minor) and 3.38 min (major); >99% ee.
Determination of d.r.: variable temperature ^{1}H NMR; observed >20:1 d.r.

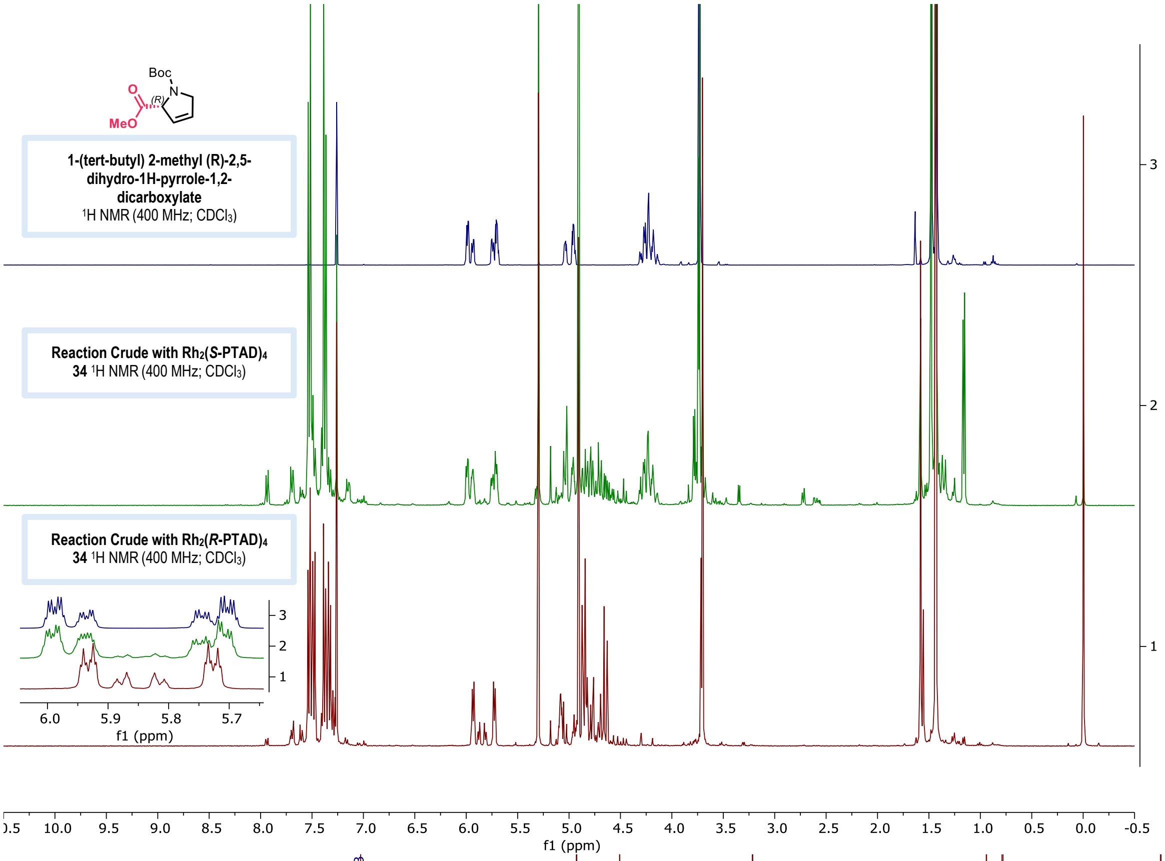
Boc
MeO
1-(tert-butyl) 2-methyl (R)-2,5-dihydro-1H-pyrrole-1,2-dicarboxylate
1H NMR (400 MHz; CDCl3)
Reaction Crude with Rh2(S-PTAD)4
34 1H NMR (400 MHz; CDCl3)
Reaction Crude with Rh2(R-PTAD)4
34 1H NMR (400 MHz; CDCl3)
f1 (ppm)

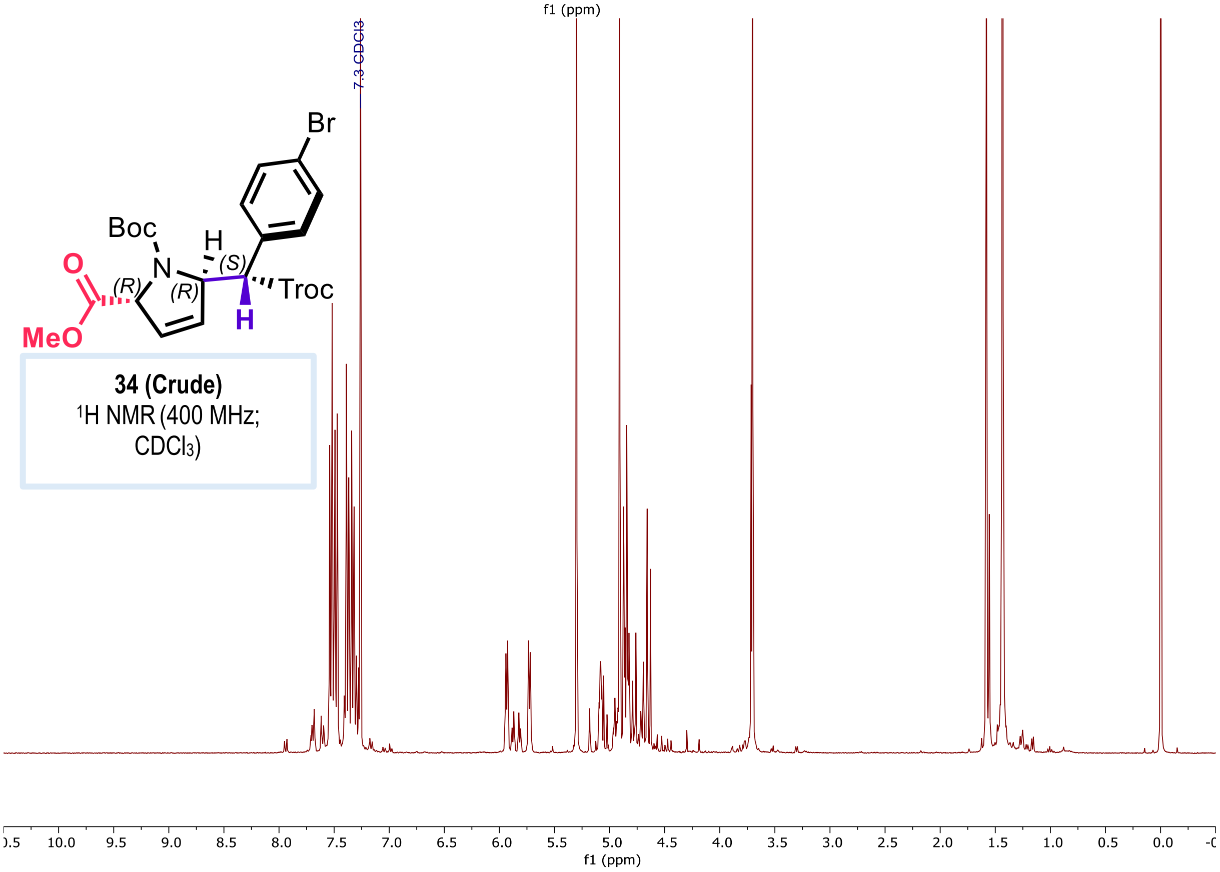
7.3 CDCl3
Br
Boc
MeO
Troc
34 (Crude)
1H NMR (400 MHz; CDCl3)
f1 (ppm)

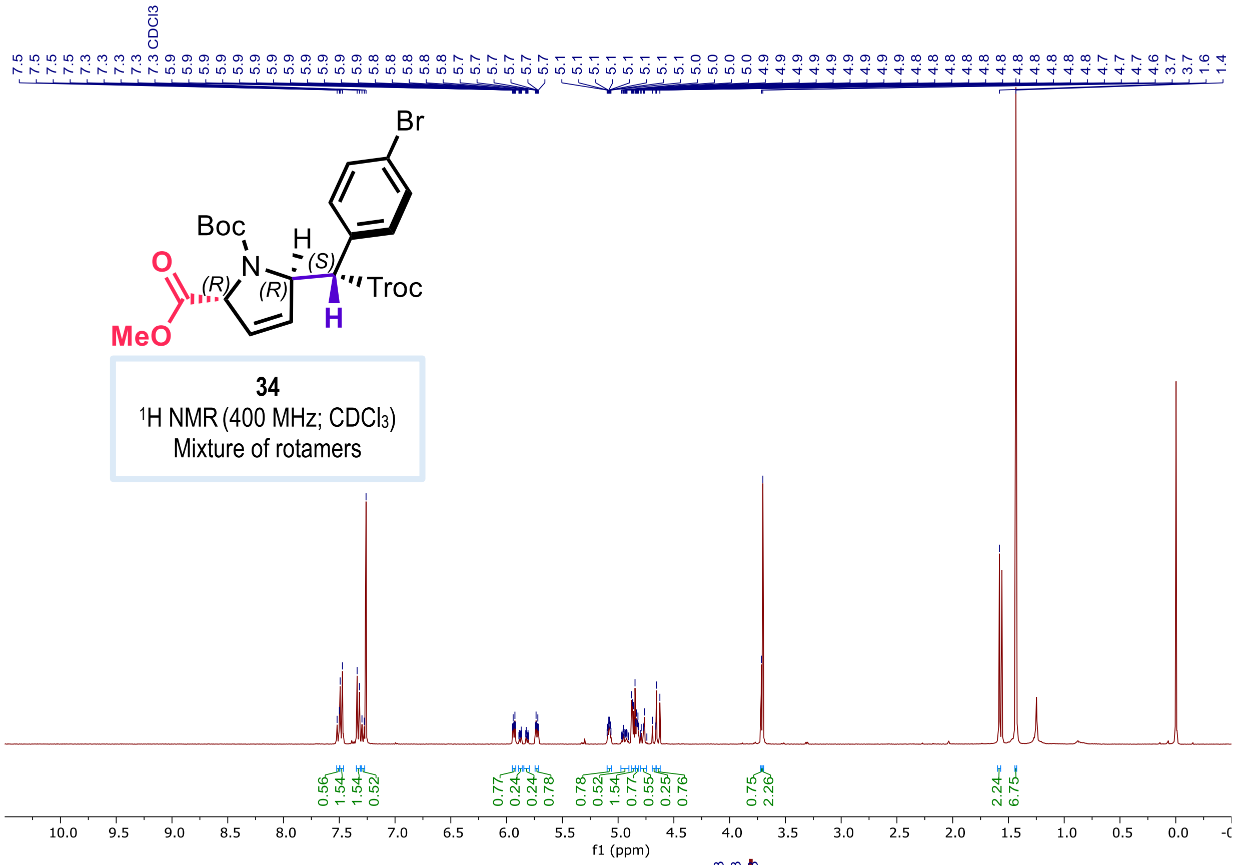
34
1H NMR (400 MHz; CDCl3)
Mixture of rotamers
Br
Boc
N
H
(S)
(R)
(R)
Troc
H
O
MeO
7.3 CDCl3
f1 (ppm)

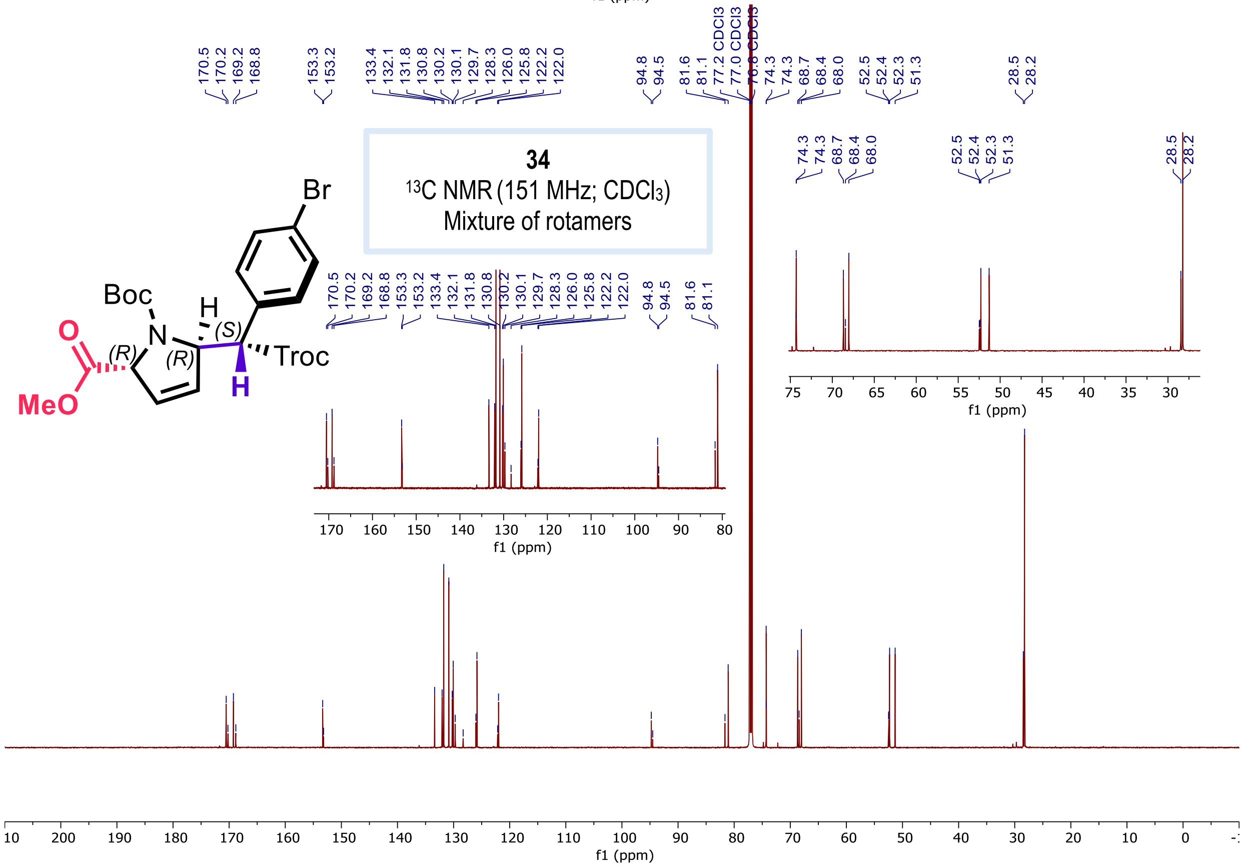
34
13C NMR (151 MHz; CDCl3)
Mixture of rotamers
Br
Boc
N
H
(S)
(R)
(R)
Troc
H
O
MeO
170.5
170.2
169.2
168.8
153.3
153.2
133.4
132.1
131.8
130.8
130.2
130.1
129.7
128.3
126.0
125.8
122.2
122.0
94.8
94.5
81.6
81.1
77.2 CDCl3
77.0 CDCl3
76.8 CDCl3
74.3
74.3
68.7
68.4
68.0
52.5
52.4
52.3
51.3
28.5
28.2
f1 (ppm)

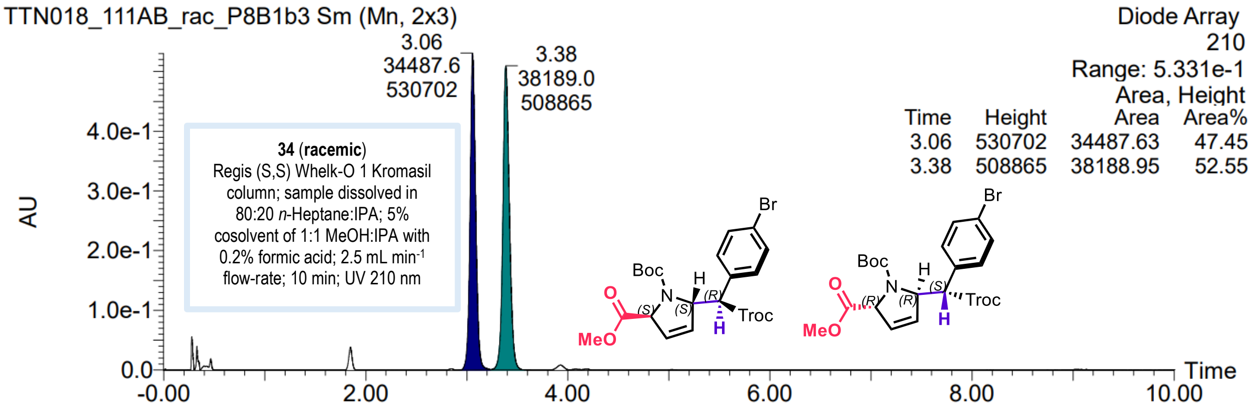
TTN018_111AB_rac_P8B1b3 Sm (Mn, 2x3)
Diode Array
210
Range: 5.331e-1
Area, Height
3.06
34487.6
530702
3.38
38189.0
508865
Time Height Area Area%
3.06 530702 34487.63 47.45
3.38 508865 38188.95 52.55
34 (racemic)
Regis (S,S) Whelk-O 1 Kromasil column; sample dissolved in 80:20 n-Heptane:IPA; 5% cosolvent of 1:1 MeOH:IPA with 0.2% formic acid; 2.5 mL min-1 flow-rate; 10 min; UV 210 nm
AU
Time
Boc
MeO
Troc
Br

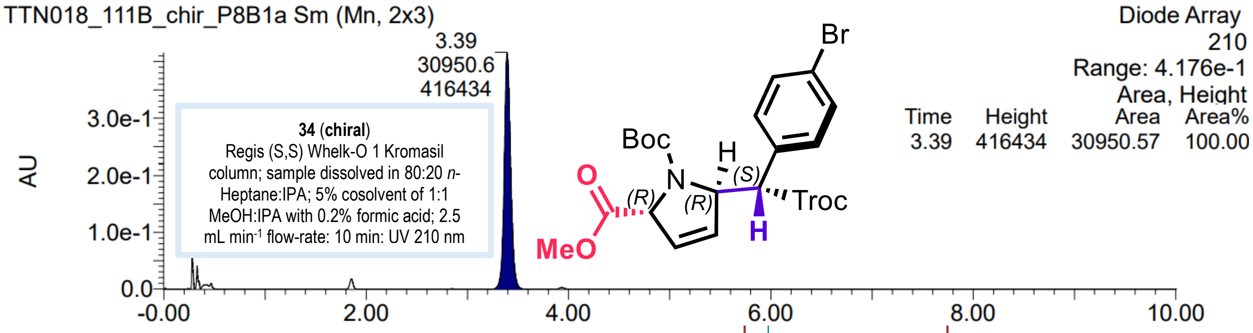
TTN018_111B_chir_P8B1a Sm (Mn, 2x3)
Diode Array
210
Range: 4.176e-1
Area, Height
3.39
30950.6
416434
Time Height Area Area%
3.39 416434 30950.57 100.00
34 (chiral)
Regis (S,S) Whelk-O 1 Kromasil column; sample dissolved in 80:20 n-Heptane:IPA; 5% cosolvent of 1:1 MeOH:IPA with 0.2% formic acid; 2.5 mL min-1 flow-rate: 10 min: UV 210 nm
AU
Boc
MeO
Troc
Br

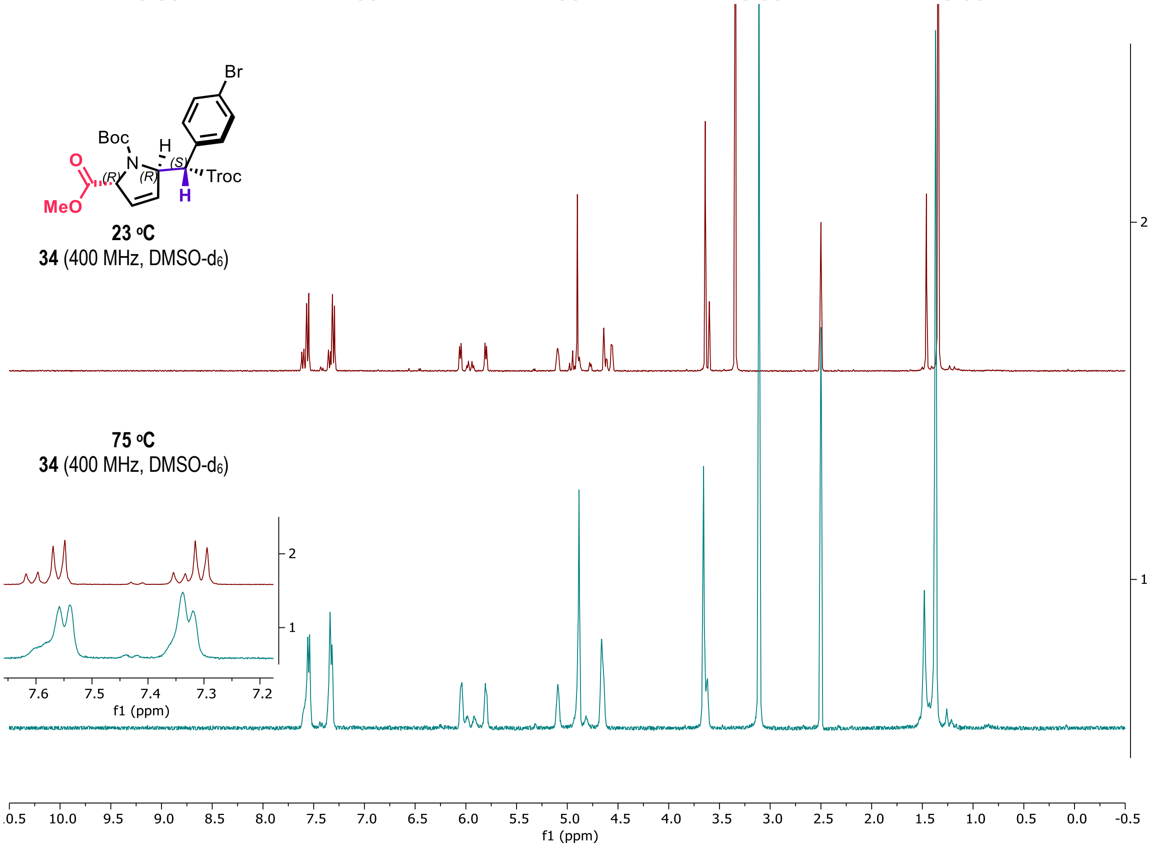
23 °C
34 (400 MHz, DMSO-d6)
75 °C
34 (400 MHz, DMSO-d6)
f1 (ppm)
Boc
MeO
Troc
Br

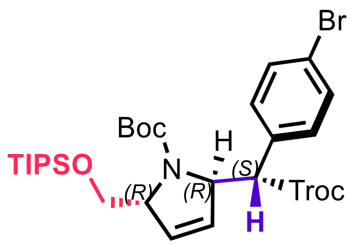

tert-butyl (2R,5R)-2-((S)-1-(4-bromophenyl)-2-oxo-2-(2,2,2-trichloroethoxy)ethyl)-5-(((triisopropylsilyl)oxy)methyl)-2,5-dihydro-1H-pyrrole-1-carboxylate (35)

To an 8 mL dram vial charged with a stir-bar and 0.5 g of activated 4Å MS was added tert-butyl (R)-2-(((triisopropylsilyl)oxy)methyl)-2,5-dihydro-1H-pyrrole-1-carboxylate (178 mg, 1 equiv, 0.500 mmol) and was backfilled with nitrogen three times. CH_2Cl_2 (0.5 mL) followed by a stock solution of Rh_2(*R*-PTAD)$_4$ (390 µg, 500 µL CH_2Cl_2, 0.0005 molar, 0.0005 equiv, 0.250 µmol) was added to the vessel. The reaction vessel containing olefin and catalyst was stirred at room temperature and for at least 10 minutes. After the elapsed time, to the solution was added 2,2,2-trichloroethyl 2-(4-bromophenyl)-2-diazoacetate (559 mg, 1.00 mL CH_2Cl_2, 1.50 molar, 3.0 equiv, 1.50 mmol) to the reaction vessel via a multi-channel syringe pump [*syringe pump settings - 1 mL syringe, 0.333 mL/hr, diameter 4.71 mm, vol 1.0 mL; Air-Tite or SilverPoint 22Gx4" long hypodermic needle was used*] over 3 hours. After complete addition and an elapsed time of 3 hours, the resulting solution was reacted for an additional 17 hours and then subsequently, filtered through a celite plug and concentrated *in vacuo* at 45 °C. The crude material was dry-loaded onto SiO_2 and subjected to SiO_2 flash chromatography using pentane/diethyl ether (Biotage Sfär DLV 50 g column as the running-column and Biotage Sfär DLV 50 g column for the dry-load; 100 mL/min flow-rate; 210/230 nm detector; 0% 6CV → 1% 6CV → 3% 6CV → 5% 6CV → 7% 6CV → 10% 6CV → 13% 6CV → 16% 6CV; POI elutes at 5% ether). The collected fractions were concentrated *in vacuo* at 45 °C to afford tert-butyl (2R,5R)-2-((S)-1-(4-bromophenyl)-2-oxo-2-(2,2,2-trichloroethoxy)ethyl)-5-(((triisopropylsilyl)oxy)methyl)-2,5-dihydro-1H-pyrrole-1-carboxylate (235 mg, 336 µmol, 67.1% yield, >99% ee, >20:1 d.r.) as a pale yellow oil.

With $Rh_2(OAc)_4$ instead of Rh_2(*R*-PTAD)$_4$ – complex mixture with insignificant product formation.

^{1}H NMR (600 MHz, Chloroform-*d*; as a 0.65:0.35 mixture of rotamers) δ 7.50 (Ar–H d, *J* = 8.5 Hz, 0.7H, minor rotamer), 7.46 (Ar–H d, *J* = 8.5 Hz, 1.3H, major rotamer), 7.36 – 7.30 (Ar–H m, 2H, mixture of rotamers), 5.92 (=C–H dt, *J* = 6.6, 1.9 Hz, 0.65H, major rotamer), 5.86 (=C–H dt, *J* = 6.6, 1.9 Hz, 0.35H, minor rotamer), 5.78 (=C–H dt, *J* = 6.6, 1.9 Hz, 0.65H, major rotamer), 5.72 (=C–H dt, *J* = 6.6, 1.9 Hz, 0.35H, minor rotamer), 4.93 – 4.90 (C–H$_{α\text{-}N}$ m, 0.65H, major rotamer), 4.89 (C–H$_{benzylic}$ d, *J* = 4.0 Hz, 0.65H, major rotamer), 4.86 (C–H$_{benzylic}$ d, *J* = 4.0 Hz, 0.35H, minor rotamer), 4.76 (C–H$_{methylene}$ overlapping d's, *J* = 12.0 Hz each d's, 1H, mixture of rotamers), 4.71 (C–H$_{methylene}$ d, *J* = 12.0 Hz, 0.65H, major rotamer), 4.68 (C–H$_{methylene}$ & C–H$_{α\text{-}N}$ d & m, *J* = 12.0 Hz, 0.7H, minor rotamers), 4.55 – 4.50 (C–H$_{α\text{-}N}$ m, 0.35H, minor rotamer), 4.39 – 4.33 (C–H$_{α\text{-}N}$ m, 0.65H, major rotamer), 4.18 (C–H$_{methylene}$ overlapping d's, *J* = 9.9 Hz each d's, 0.35H, minor rotamer), 4.08 (C–H$_{methylene}$ overlapping d's, *J* = 9.0 Hz each d's, 0.65H, major rotamer), 3.85 (C–H$_{methylene}$ overlapping d's, *J* = 9.9 Hz each d's, 0.35H, minor rotamer), 3.62 (C–H$_{methylene}$ overlapping d's, *J* = 9.0 Hz each d's, 0.65H, major rotamer), 1.58 (–C(CH_3)$_3$ s, 3.15H, minor rotamer), 1.50 (–C(CH_3)$_3$ s, 5.85H, major rotamer), 1.04 – 0.97 (–CH(CH_3)$_2$ m, 21H, mixture of rotamers).

^{13}C NMR (151 MHz, Chloroform-*d*; as a mixture of rotamers) δ 169.6, 169.1, 153.7, 152.7, 134.0, 133.9, 131.9, 131.5, 131.2, 131.2, 130.6, 130.4, 126.1, 125.7, 121.8, 121.7, 94.8, 94.6, 80.4, 80.3, 74.5, 74.3, 69.3, 69.2, 67.3, 66.4, 64.4, 61.5, 52.3, 51.0, 28.7, 28.5, 18.0, 18.0, 11.9, 11.9.

HRMS (ESI +) calc. mass for $C_{29}H_{44}O_5N^{79}Br^{35}Cl_3{}^{28}Si$ [M + H]$^+$ 698.1232; obs. mass for $C_{29}H_{44}O_5N^{79}Br^{35}Cl_3{}^{28}Si$ [M + H]$^+$ 698.1235.

[α]$^{20}_{D}$: +181.9° (c = 0.145 g/100 mL, EtOAc).
SFC (Regis (S,S) Whelk-O 1 Kromasil column; sample dissolved in 80:20 *n*-Heptane:IPA; 2% cosolvent of 1:1 MeOH:IPA with 0.2% formic acid; 2.5 mL min^{-1} flow-rate; 15 min; UV 230 nm): retention times of 7.50 min (minor) and 9.31 min (major); >99% ee.
Determination of d.r.: variable temperature ^{1}H NMR; observed >20:1 d.r.

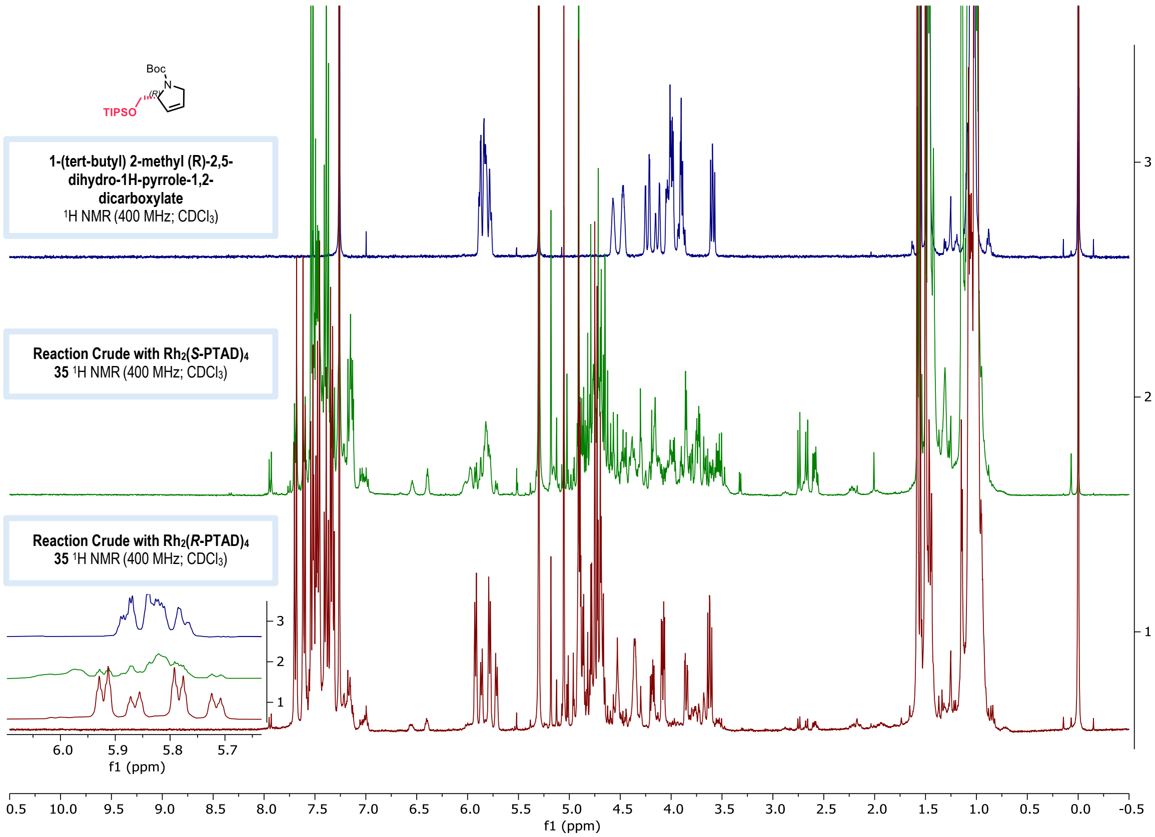

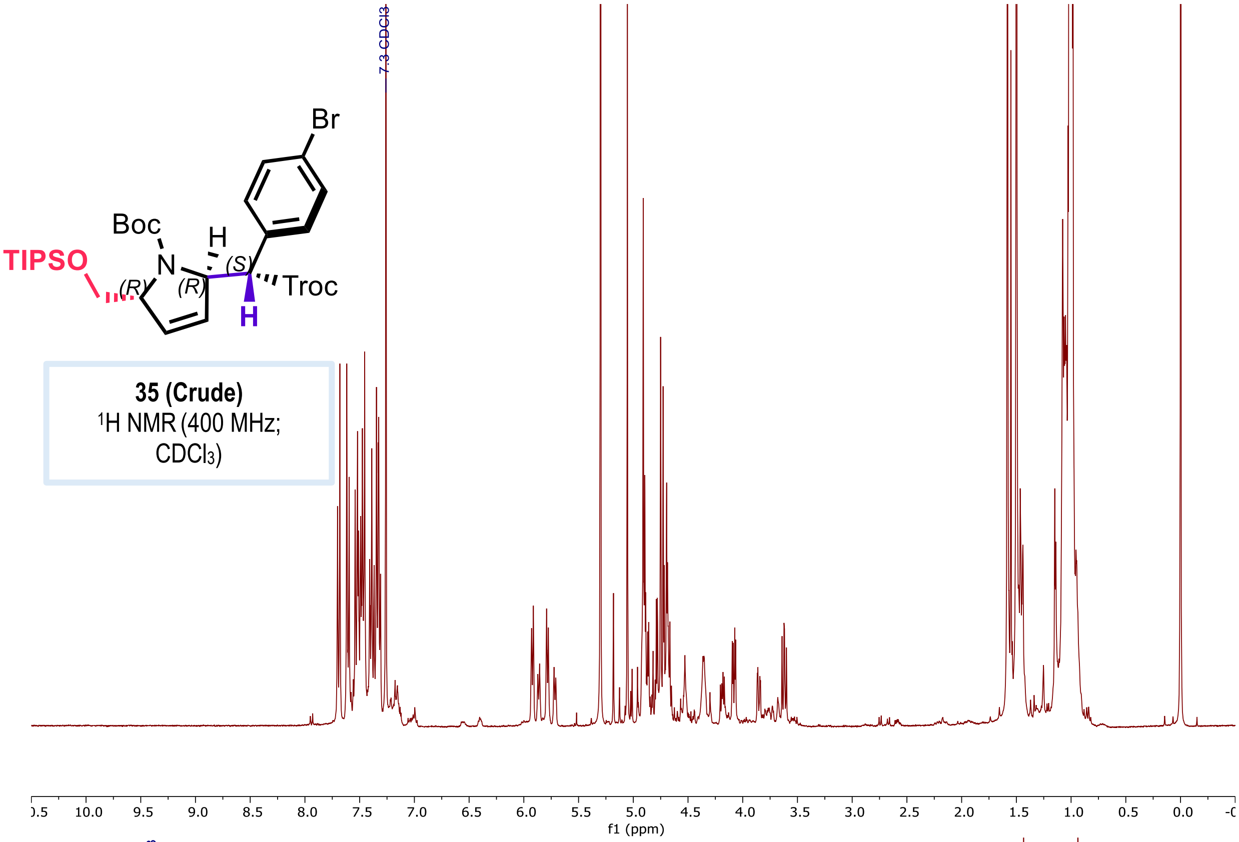

35 (Crude)
^{1}H NMR (400 MHz; $CDCl_3$)

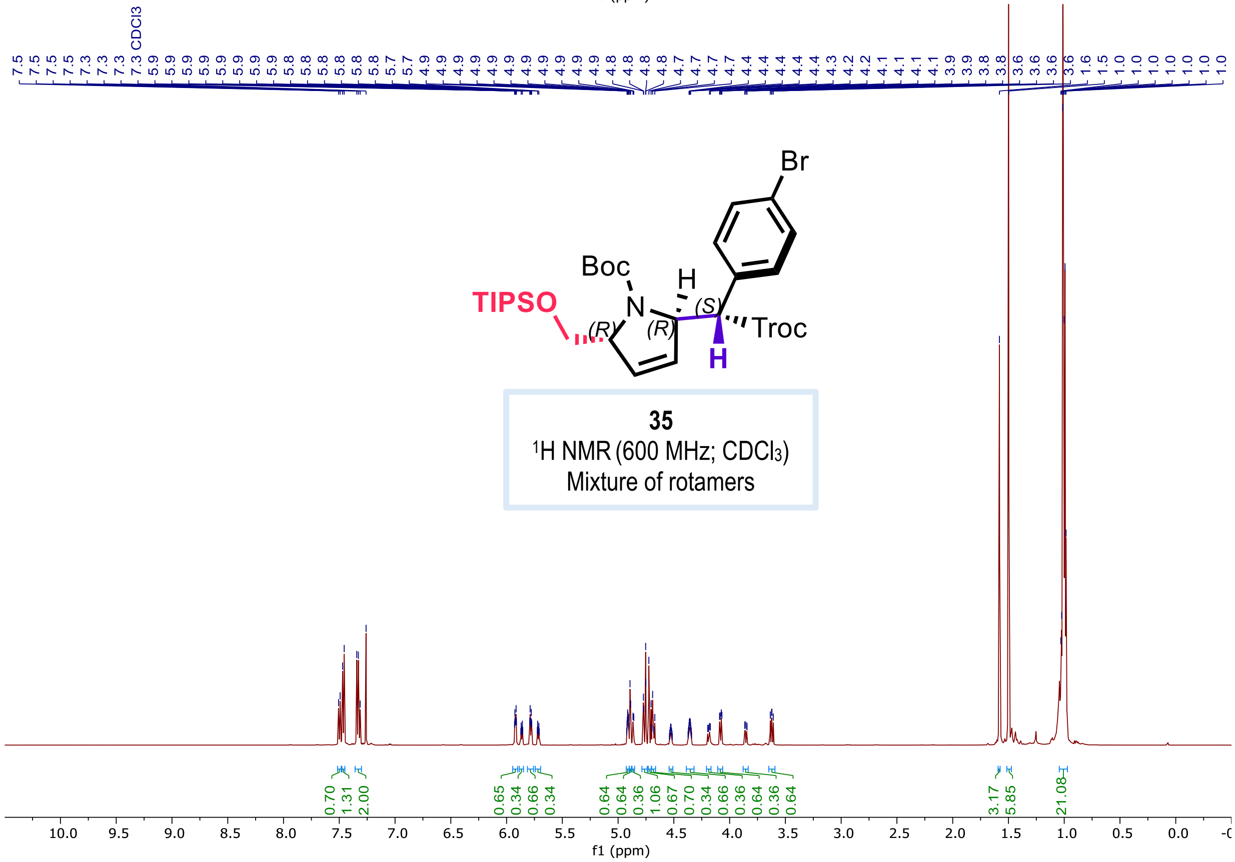

35
^{1}H NMR (600 MHz; $CDCl_3$)
Mixture of rotamers

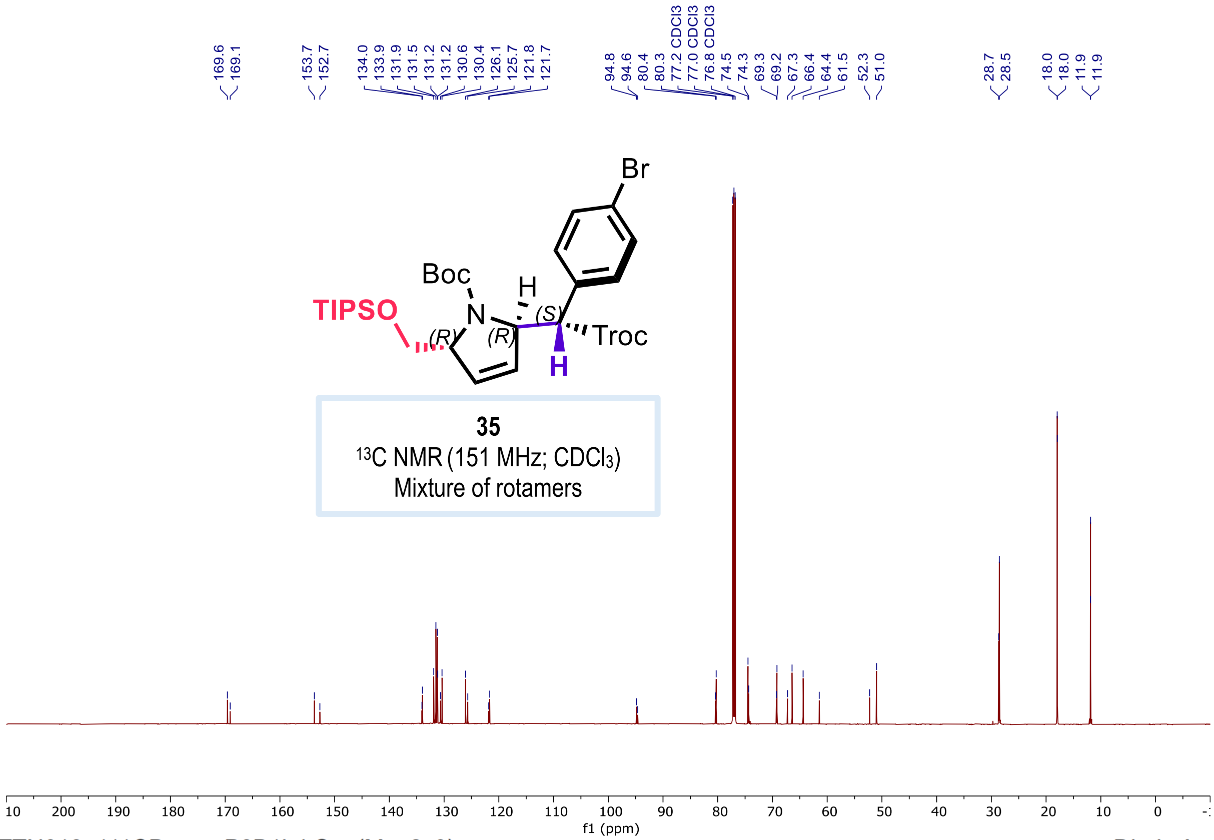
169.6
169.1
153.7
152.7
134.0
133.9
131.9
131.5
131.2
131.2
130.6
130.4
126.1
125.7
121.8
121.7
94.8
94.6
80.4
80.3
77.2 CDCl3
77.0 CDCl3
76.8 CDCl3
74.5
74.3
69.3
69.2
67.3
66.4
64.4
61.5
52.3
51.0
28.7
28.5
18.0
18.0
11.9
11.9
Br
Boc
H
TIPSO
N
(R)
(R)
(S)
Troc
H
35
^{13}C NMR (151 MHz; $CDCl_3$)
Mixture of rotamers
f1 (ppm)

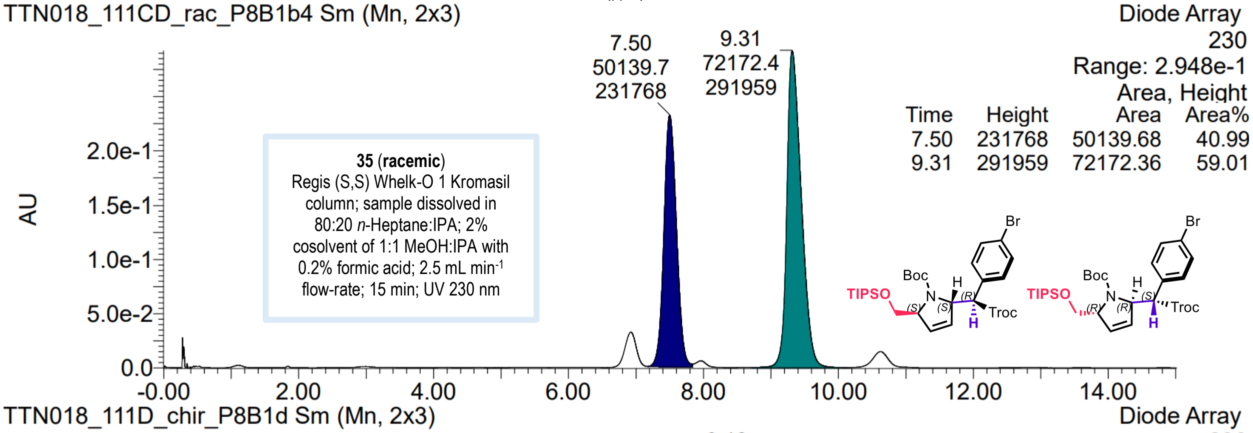
TTN018_111CD_rac_P8B1b4 Sm (Mn, 2x3)
Diode Array
230
Range: 2.948e-1
Area, Height
7.50
50139.7
231768
9.31
72172.4
291959
Time Height Area Area%
7.50 231768 50139.68 40.99
9.31 291959 72172.36 59.01
AU
35 (racemic)
Regis (S,S) Whelk-O 1 Kromasil column; sample dissolved in 80:20 n-Heptane:IPA; 2% cosolvent of 1:1 MeOH:IPA with 0.2% formic acid; 2.5 mL min-1 flow-rate; 15 min; UV 230 nm
TIPSO
Boc
N
H
Troc
Br

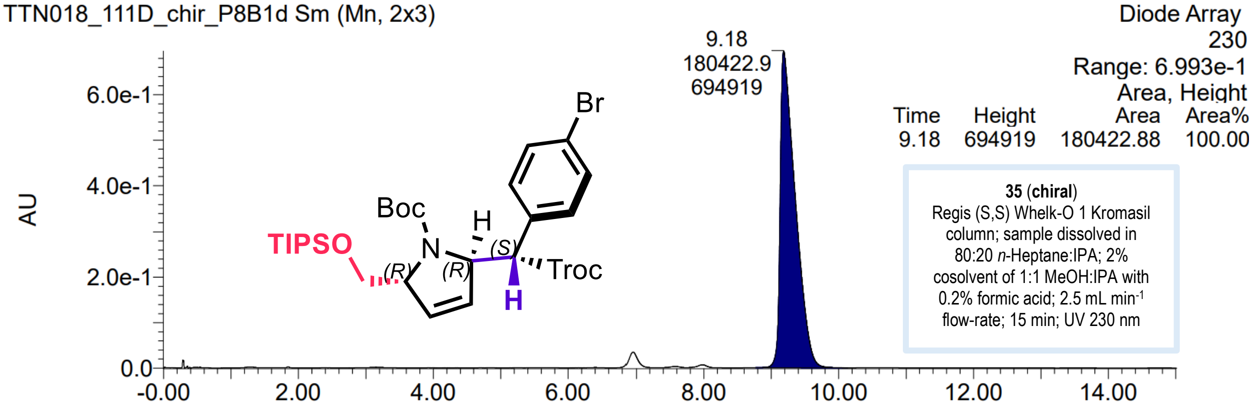
TTN018_111D_chir_P8B1d Sm (Mn, 2x3)
Diode Array
230
Range: 6.993e-1
Area, Height
9.18
180422.9
694919
Time Height Area Area%
9.18 694919 180422.88 100.00
AU
Br
Boc
H
TIPSO
N
(R)
(R)
(S)
Troc
H
35 (chiral)
Regis (S,S) Whelk-O 1 Kromasil column; sample dissolved in 80:20 n-Heptane:IPA; 2% cosolvent of 1:1 MeOH:IPA with 0.2% formic acid; 2.5 mL min-1 flow-rate; 15 min; UV 230 nm

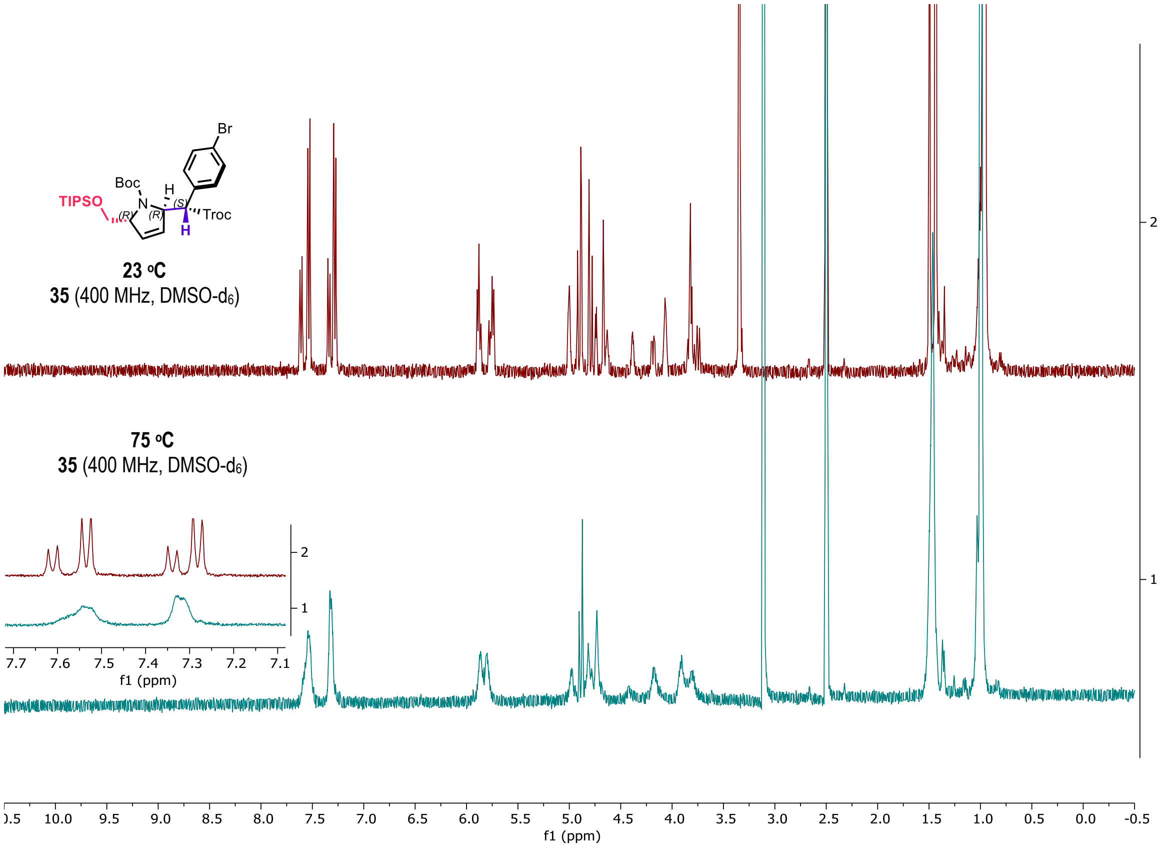

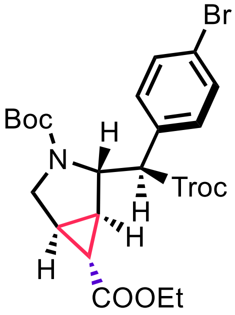

3-(tert-butyl) 6-ethyl (1R,2S,5S,6R)-2-(1-(4-bromophenyl)-2-oxo-2-(2,2,2-trichloroethoxy)ethyl)-3-azabicyclo[3.1.0]hexane-3,6-dicarboxylate (*exo*-36)

To a 4 mL flame-dried vial charged with a stir-bar and 0.25 g of activated 4Å MS was added tert-butyl (S)-2-((R)-1-(4-bromophenyl)-2-oxo-2-(2,2,2-trichloroethoxy)ethyl)-2,5-dihydro-1H-pyrrole-1-carboxylate (514 mg, 1 equiv, 1.00 mmol) and $Rh_2(esp)_2$ (7.58 mg, .01 equiv, 10.0 µmol) to the vial. The vessel was back-filled with nitrogen three times. The reaction vessel was sealed, and a nitrogen balloon attached. CH_2Cl_2 (1.29 mL) was then added to the reaction vessel. The reaction vessel containing olefin and catalyst was stirred at 45 °C and for at least 10 minutes making sure there is adequate stirring. After the elapsed time, to the reaction vessel was added ethyl 2-diazoacetate (771 mg, 711 µL CH_2Cl_2, 74% Wt, 5 equiv, 5.00 mmol) in CH_2Cl_2 to the reaction vessel via a dual-channel syringe pump [*dual-syringe pump settings - 1 mL syringe, 0.257 mL/hr, diameter 4.71 mm, vol 0.771 mL; Air-Tite 22Gx4" long hypodermic needle was used*] over 3 hours. After complete addition and an elapsed time of 3 hours, the resulting solution was reacted for an additional 17 h and then subsequently, cooled and filtered through a celite plug and the filtrate concentrated *in vacuo* at 50 °C. The crude was dry-loaded onto SiO_2 and subjected to SiO_2 flash chromatography using pentanes/diethyl ether (Biotage Sfär DLV 50 g column as the running column and Biotage Sfär DLV 50 g column for the dry-load; 0% 8CV → 1% 8CV → 3% 8CV → 5% 8CV → 7%

8CV → 10% 8CV → 13% 8CV → 16% 8CV → 20% 8CV → 25% 8CV; 100 mL/min flow-rate; 210/230 nm detector; POI elutes at 16% to 20% ether). The collected fractions were concentrated *in vacuo* at 45 ^{o}C to 3-(tert-butyl) 6-ethyl (1R,2S,5S,6R)-2-(1-(4-bromophenyl)-2-oxo-2-(2,2,2-trichloroethoxy)ethyl)-3-azabicyclo[3.1.0]hexane-3,6-dicarboxylate (452 mg, 754 µmol, 75.4% combined yield [***exo*** & ***endo***], 94% ee ***exo***, 1:1 [***exo***:***endo***] d.r. based on crude; >20:1 d.r. ***exo*** after purified). Structural assignment is tentatively assigned based on chemical shifts of *exo*- and *endo*-3-azabicyclo[3.1.0]hexane-6-carboxylates.[3]

^{1}H NMR (400 MHz, Chloroform-*d*; as a 0.55:0.45 mixture of rotamers) δ 7.47 (Ar–H d, *J* = 8.3 Hz, 2H, mixture of rotamers), 7.31 (Ar–H d, *J* = 8.3 Hz, 0.9H, minor rotamer), 7.21 (Ar–H d, *J* = 8.3 Hz, 1.1H, major rotamer), 4.85 (dd, *J* = 12.0, 5.1 Hz, 1H, mixture of rotamers), 4.74 (dd, *J* = 12.0, 5.1 Hz, 1H, mixture of rotamers), 4.49 (d, *J* = 6.1 Hz, 0.45H, minor rotamer), 4.41 (d, *J* = 8.2 Hz, 0.55H, major rotamer), 4.27 – 4.20 (m, 1H, mixture of rotamers), 4.18 (d, *J* = 6.1 Hz, 0.45H, minor rotamer), 4.16 – 4.06 (m, 2H, mixture of rotamers), 3.88 (dd, *J* = 14.0, 9.9 Hz, 1H, mixture of rotamers), 3.59 (d, *J* = 11.3 Hz, 0.45H, minor rotamer), 3.36 (dd, *J* = 11.3, 3.5 Hz, 0.55H, major rotamer), 3.29 (dd, *J* = 11.3, 3.5 Hz, 0.45H, minor rotamer), 2.16 – 2.00 (m, 2H, mixture of rotamers), 1.48 – 1.41 (m, 1H, mixture of rotamers), 1.33 – 1.17 (m, 12H, mixture of rotamers).
^{13}C NMR (151 MHz, Chloroform-*d*; as a mixture of rotamers) δ 171.9, 171.8, 169.8, 169.8, 154.0, 153.8, 133.4, 133.2, 131.9, 131.7, 130.8, 130.5, 122.2, 122.0, 94.6, 94.5, 80.6, 80.3, 74.4, 74.3, 68.2, 62.3, 62.1, 61.1, 60.9, 60.8, 54.1, 53.1, 48.2, 47.1, 29.7, 29.5, 28.6, 28.3, 28.1, 26.2, 24.9, 24.5, 23.7, 14.2.
HRMS (ESI +) calc. mass for $C_{23}H_{28}O_6N^{79}Br^{35}Cl_3$ $[M + H]^+$ 598.0160; obs. mass for $C_{23}H_{28}O_6N^{79}Br^{35}Cl_3$ $[M + H]^+$ 598.0163.
$[\alpha]^{20}_{D}$: -5.6^{o} (c = 0.180 g/100 mL, EtOAc).
SFC (Regis (S,S) Whelk-O 1 Kromasil column; sample dissolved in 80:20 *n*-Heptane:IPA; 1% cosolvent of 1:1 MeOH:IPA with 0.2% formic acid; 2.5 mL min^{-1} flow-rate; 30 min; UV 210 nm): retention times of 20.21 min (minor) and 23.72 min (major); 94% ee.
Determination of d.r.: variable temperature ^{1}H NMR; observed >20:1 d.r. ***exo***

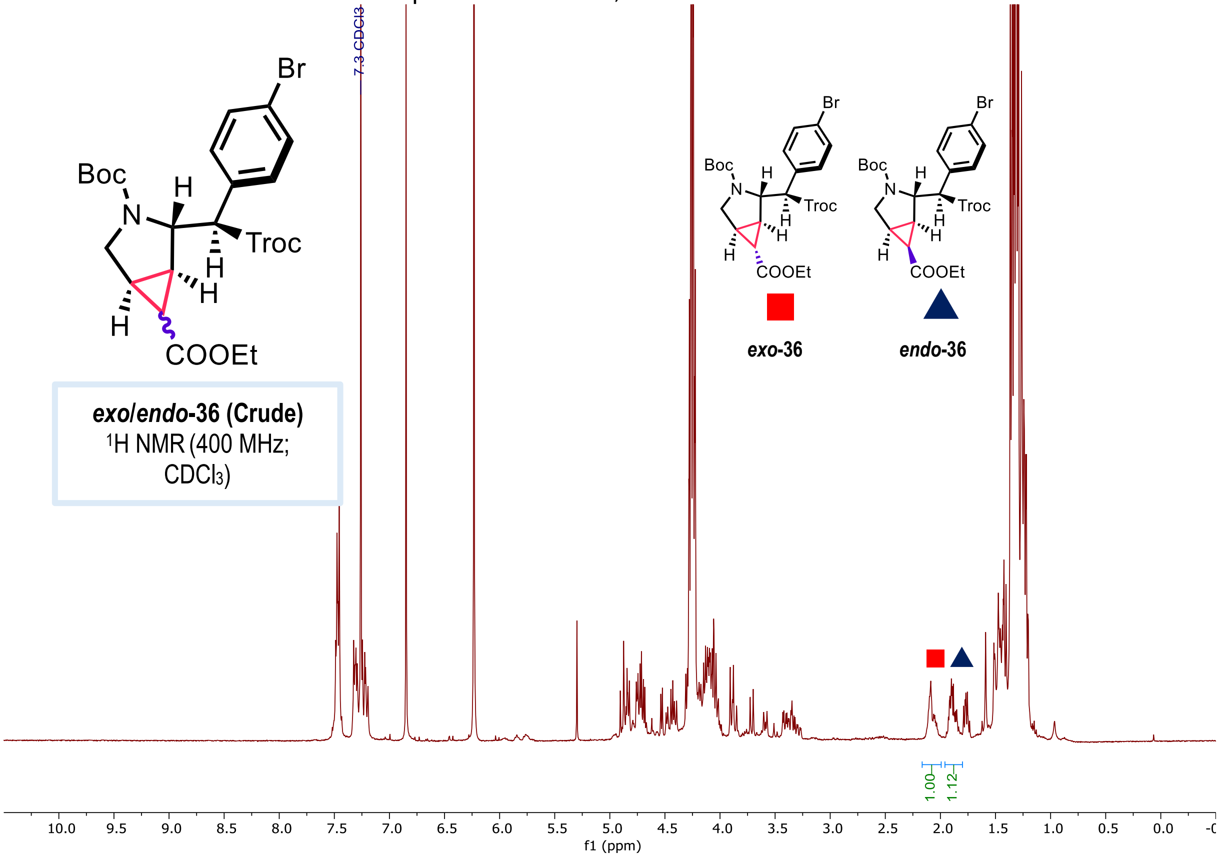

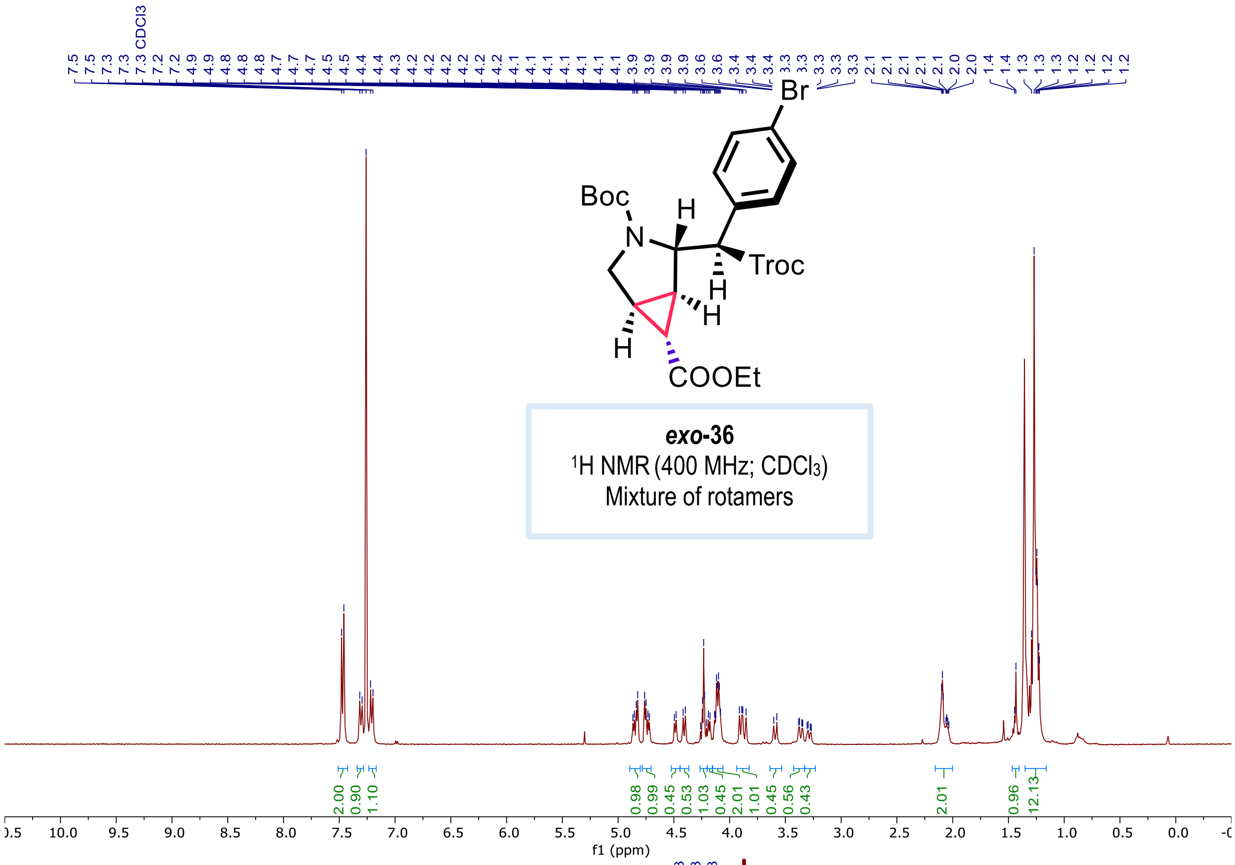

Boc
N
H
Br
Troc
H
H
H
COOEt
exo-36
1H NMR (400 MHz; CDCl3)
Mixture of rotamers
f1 (ppm)

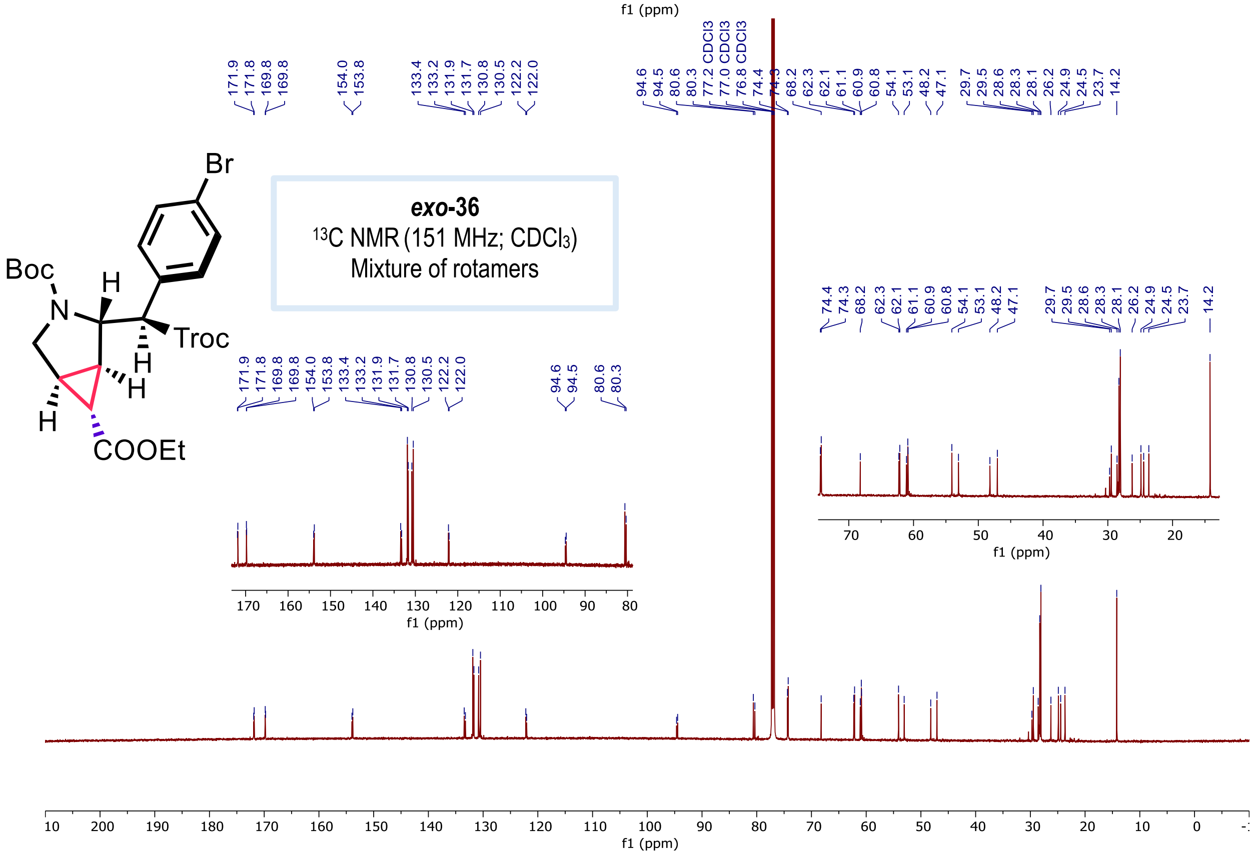

Br
Boc
N
H
Troc
H
H
H
COOEt
exo-36
13C NMR (151 MHz; CDCl3)
Mixture of rotamers
f1 (ppm)

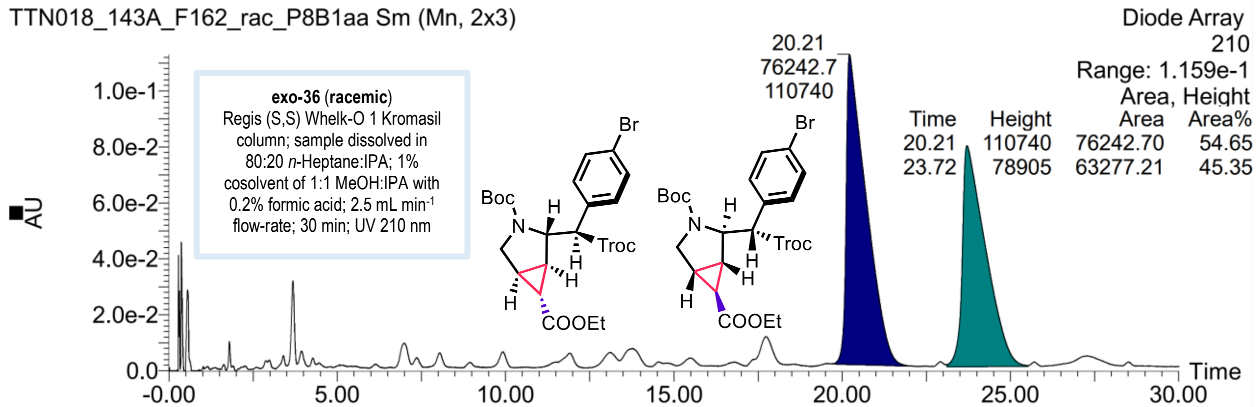

TTN018_143A_F162_rac_P8B1aa Sm (Mn, 2x3)
Diode Array
210
Range: 1.159e-1
Area, Height
exo-36 (racemic)
Regis (S,S) Whelk-O 1 Kromasil column; sample dissolved in 80:20 n-Heptane:IPA; 1% cosolvent of 1:1 MeOH:IPA with 0.2% formic acid; 2.5 mL min-1 flow-rate; 30 min; UV 210 nm
20.21
76242.7
110740
Time Height Area Area%
20.21 110740 76242.70 54.65
23.72 78905 63277.21 45.35
AU
Time

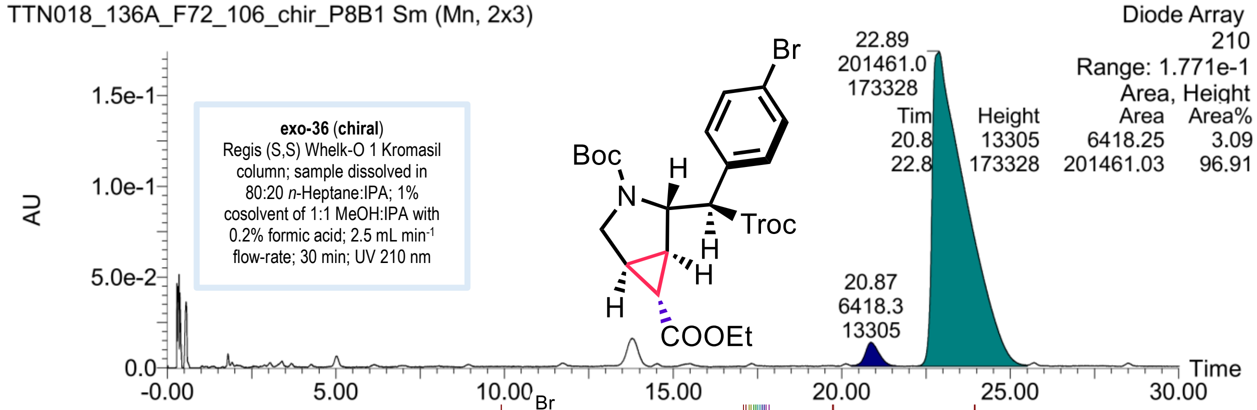

TTN018_136A_F72_106_chir_P8B1 Sm (Mn, 2x3)
Diode Array
210
Range: 1.771e-1
Area, Height
exo-36 (chiral)
Regis (S,S) Whelk-O 1 Kromasil column; sample dissolved in 80:20 n-Heptane:IPA; 1% cosolvent of 1:1 MeOH:IPA with 0.2% formic acid; 2.5 mL min-1 flow-rate; 30 min; UV 210 nm
22.89
201461.0
173328
Height Area Area%
13305 6418.25 3.09
173328 201461.03 96.91
20.87
6418.3
13305
AU
Time

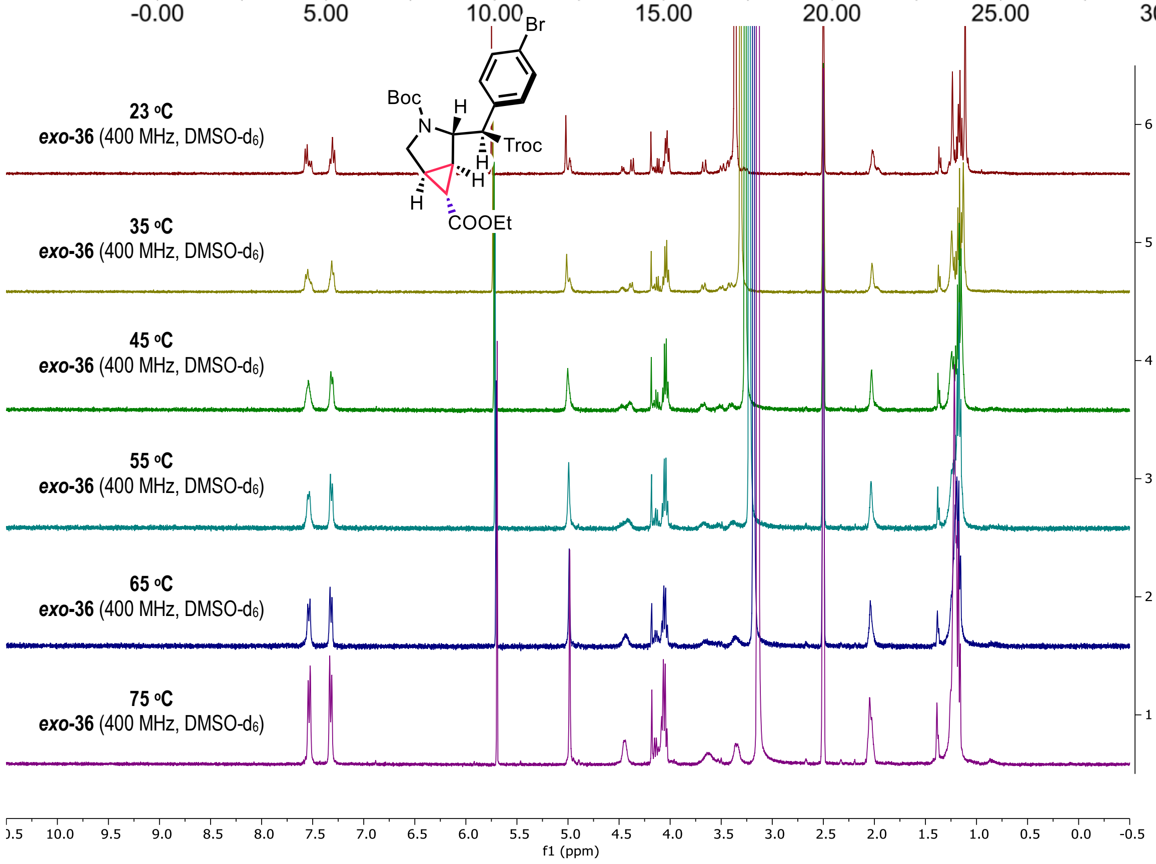

23 °C
exo-36 (400 MHz, DMSO-d6)
35 °C
exo-36 (400 MHz, DMSO-d6)
45 °C
exo-36 (400 MHz, DMSO-d6)
55 °C
exo-36 (400 MHz, DMSO-d6)
65 °C
exo-36 (400 MHz, DMSO-d6)
75 °C
exo-36 (400 MHz, DMSO-d6)
f1 (ppm)

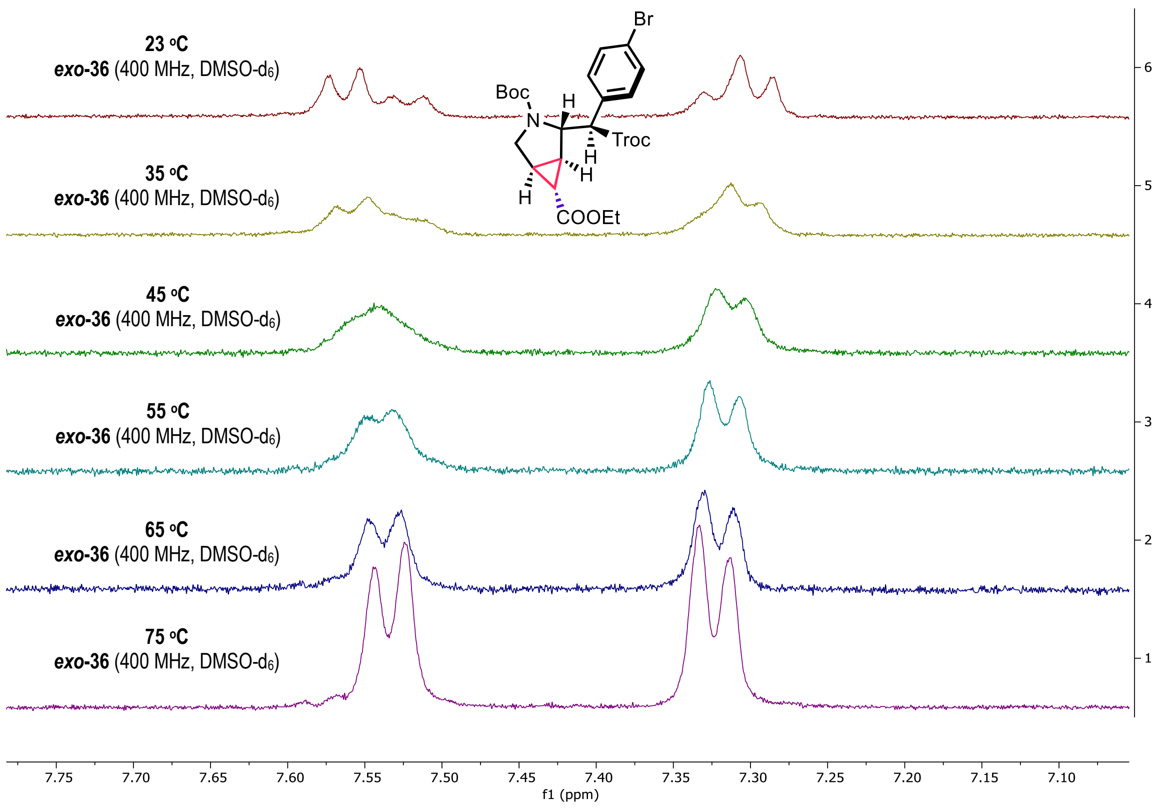

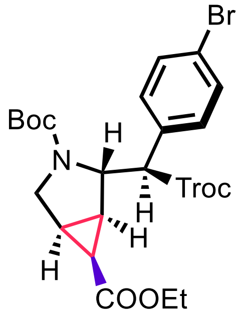

3-(tert-butyl) 6-ethyl (1R,2S,5S)-2-(1-(4-bromophenyl)-2-oxo-2-(2,2,2-trichloroethoxy)ethyl)-3-azabicyclo[3.1.0]hexane-3,6-dicarboxylate (*endo*-36)

To a 4 mL flame-dried vial charged with a stir-bar and 0.25 g of activated 4Å MS was added tert-butyl (S)-2-((R)-1-(4-bromophenyl)-2-oxo-2-(2,2,2-trichloroethoxy)ethyl)-2,5-dihydro-1H-pyrrole-1-carboxylate (514 mg, 1 equiv, 1.00 mmol) and $Rh_2(esp)_2$ (7.58 mg, .01 equiv, 10.0 µmol) to the vial. The vessel was back-filled with nitrogen three times. The reaction vessel was sealed, and a nitrogen balloon attached. CH_2Cl_2 (1.29 mL) was then added to the reaction vessel. The reaction vessel containing olefin and catalyst was stirred at 45 °C and for at least 10 minutes making sure there is adequate stirring. After the elapsed time, to the reaction vessel was added ethyl 2-diazoacetate (771 mg, 711 µL CH_2Cl_2, 74% Wt, 5 equiv, 5.00 mmol) in CH_2Cl_2 to the reaction vessel via a dual-channel syringe pump [*dual-syringe pump settings - 1 mL syringe, 0.257 mL/hr, diameter 4.71 mm, vol 0.771 mL; Air-Tite 22Gx4" long hypodermic needle was used*] over 3 hours. After complete addition and an elapsed time of 3 hours, the resulting solution was reacted for an additional 17 h and then subsequently, cooled and filtered through a celite plug and the filtrate concentrated *in vacuo* at 50 °C. The crude was dry-loaded onto SiO_2 and subjected to SiO_2 flash chromatography using pentanes/diethyl ether (Biotage Sfär DLV 50 g column as the running column and Biotage Sfär DLV 50 g column for the dry-load; 0% 8CV → 1% 8CV → 3% 8CV → 5% 8CV → 7%

8CV → 10% 8CV → 13% 8CV → 16% 8CV → 20% 8CV → 25% 8CV; 100 mL/min flow-rate; 210/230 nm detector; POI elutes at 16% to 20% ether). The collected fractions were concentrated *in vacuo* at 45 °C to 3-(tert-butyl) 6-ethyl (1R,2S,5S)-2-(1-(4-bromophenyl)-2-oxo-2-(2,2,2-trichloroethoxy)ethyl)-3-azabicyclo[3.1.0]hexane-3,6-dicarboxylate (452 mg, 754 µmol, 75.4% combined yield [***exo*** & ***endo***], 94% ee ***endo***, 1:1 [***exo***:***endo***] d.r. based on crude, >20:1 d.r. ***endo*** after purified). Structural assignment is tentatively assigned based on chemical shifts of *exo*- and *endo*-3-azabicyclo[3.1.0]hexane-6-carboxylates.[3]

^{1}H NMR (400 MHz, Chloroform-*d*; as a 0.55:0.45 mixture of rotamers) δ 7.47 (Ar–H overlapping d's, *J* = 8.4 Hz each d's, 2H, mixture of rotamers), 7.32 (Ar–H d, *J* = 8.4 Hz, 0.9H, minor rotamer), 7.24 (Ar–H d, *J* = 8.4 Hz, 1.1H, major rotamer), 4.87 (overlapping d's, *J* = 12.2 Hz each d's, 1H, mixture of rotamers), 4.72 (d, *J* = 4.5 Hz, 0.55H, major rotamer), 4.69 (d, *J* = 4.5 Hz, 0.45H, minor rotamer), 4.54 (d, *J* = 5.8 Hz, 0.55H, major rotamer), 4.44 (d, *J* = 6.9 Hz, 0.45H, minor rotamer), 4.31 (d, *J* = 5.8 Hz, 0.45H, minor rotamer), 4.15 (d, *J* = 6.9 Hz, 0.55H, major rotamer), 4.10 – 3.98 (m, 2H, mixture of rotamers), 3.90 (d, *J* = 11.3 Hz, 0.45H, minor rotamer), 3.72 (d, *J* = 11.3 Hz, 0.55H, major rotamer), 3.41 (dd, *J* = 11.3, 4.1 Hz, 0.45H, minor rotamer), 3.34 (dd, *J* = 11.3, 4.1 Hz, 0.55H, major rotamer), 1.99 – 1.82 (m, 2H, mixture of rotamers), 1.80 – 1.71 (m, 1H, mixture of rotamers), 1.37 (s, 4.05H, minor rotamer), 1.35 (s, 4.95H, major rotamer), 1.24 – 1.18 (m, 3H, mixture of rotamers).
^{13}C NMR (151 MHz, Chloroform-*d*; as a mixture of rotamers) δ 170.1, 170.0, 168.6, 168.4, 153.8, 153.4, 133.7, 133.6, 131.9, 131.7, 130.7, 130.4, 122.0, 121.9, 94.7, 94.5, 80.2, 79.9, 74.3, 74.2, 60.6, 60.6, 60.4, 53.8, 52.9, 46.1, 45.5, 28.3, 28.2, 24.9, 24.4, 22.6, 22.5, 22.0, 21.1, 14.2, 14.2.
HRMS (ESI +) calc. mass for $C_{23}H_{28}O_6N^{79}Br^{35}Cl_3$ $[M + H]^+$ 598.0160; obs. mass for $C_{23}H_{28}O_6N^{79}Br^{35}Cl_3$ $[M + H]^+$ 598.0163.
$[\alpha]^{20}_D$: -2.2° (c = 0.190 g/100 mL, EtOAc).
HPLC (Chiralpak AD-H column; sample dissolved in 20:80 IPA:*n*-Heptane; 3% IPA in *n*-Hexane; 1 mL min^{-1} flow-rate; 30 min; UV 230 nm): retention times of 11.55 min (major) and 13.21 min (minor); 94% ee.
Determination of d.r.: *N*-Boc deprotection (10 equiv of TFA in CH_2Cl_2 [0.25 M] at 25 °C, 24 hours); observed >20:1 d.r.

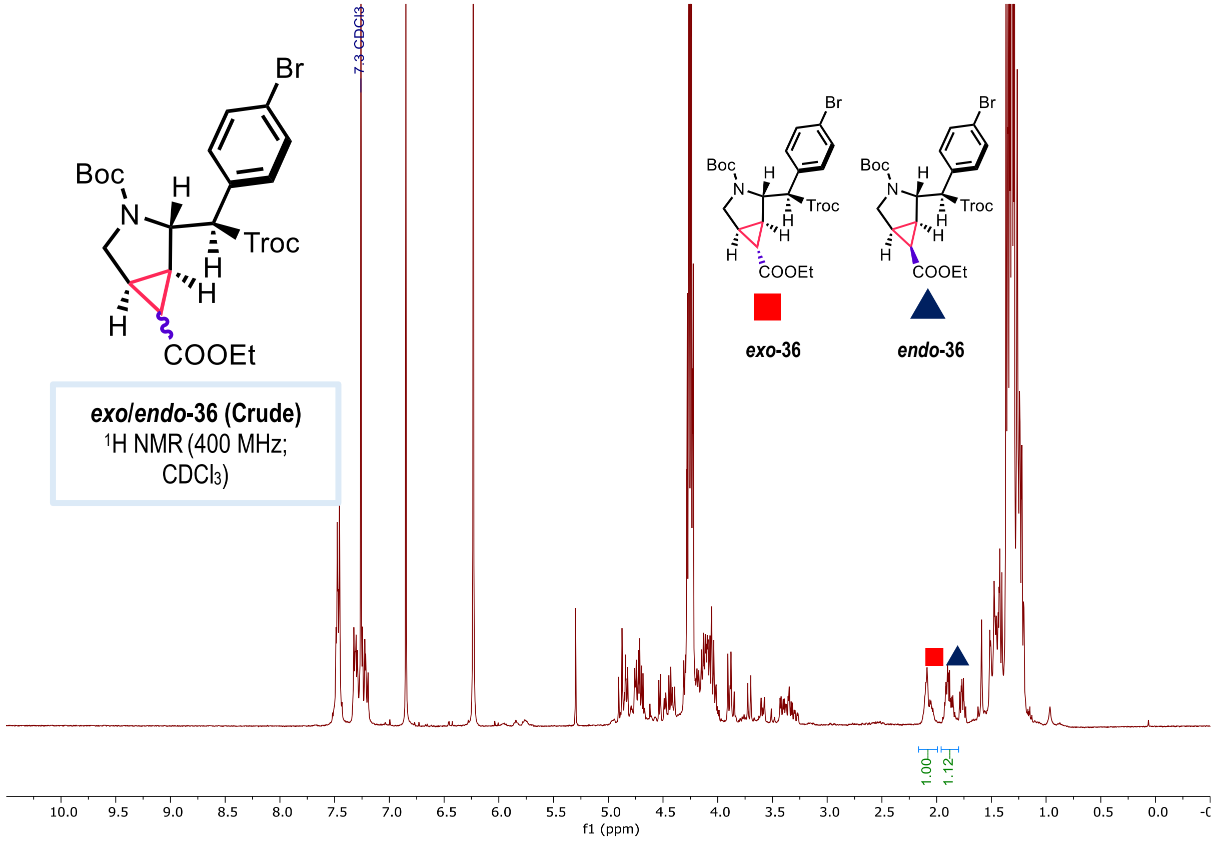

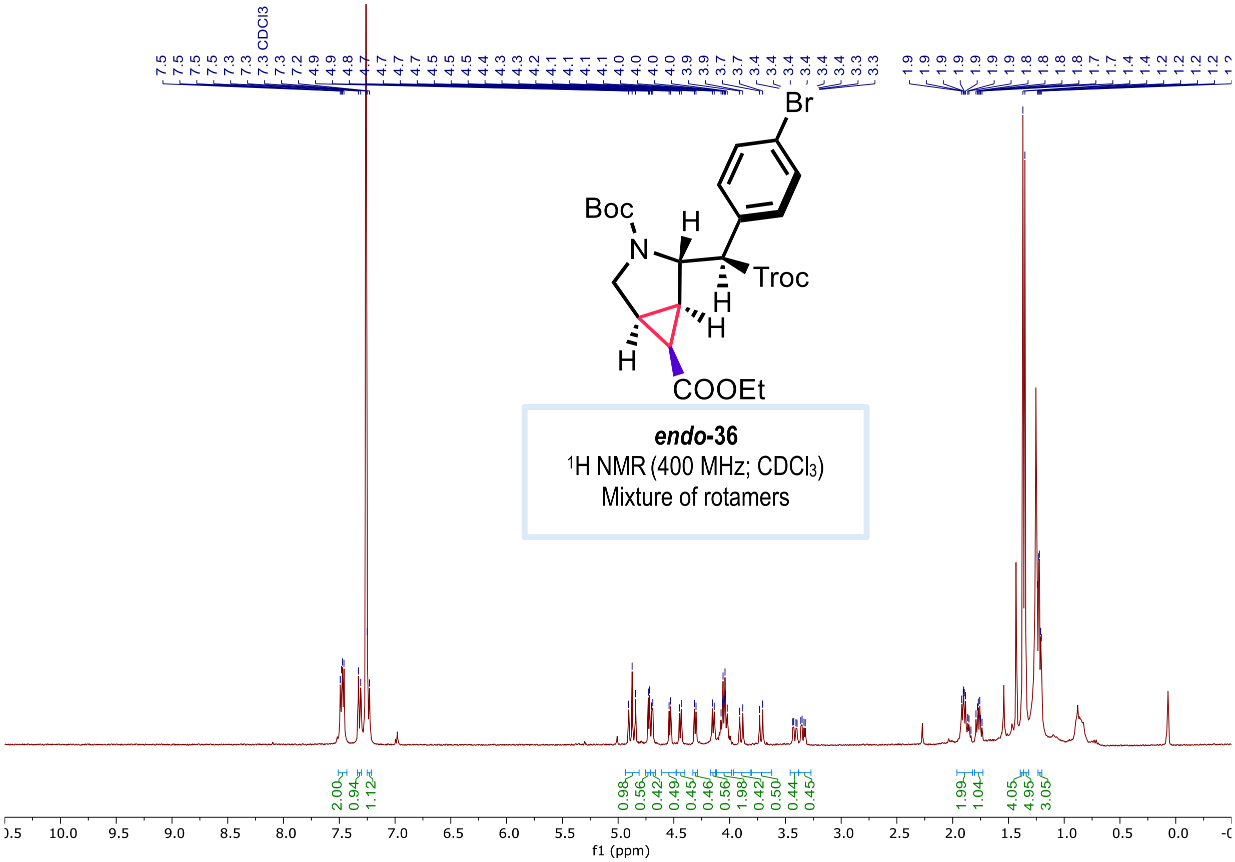
endo-36
¹H NMR (400 MHz; CDCl3)
Mixture of rotamers
Boc
N
H
Troc
Br
H
H
H
COOEt
f1 (ppm)

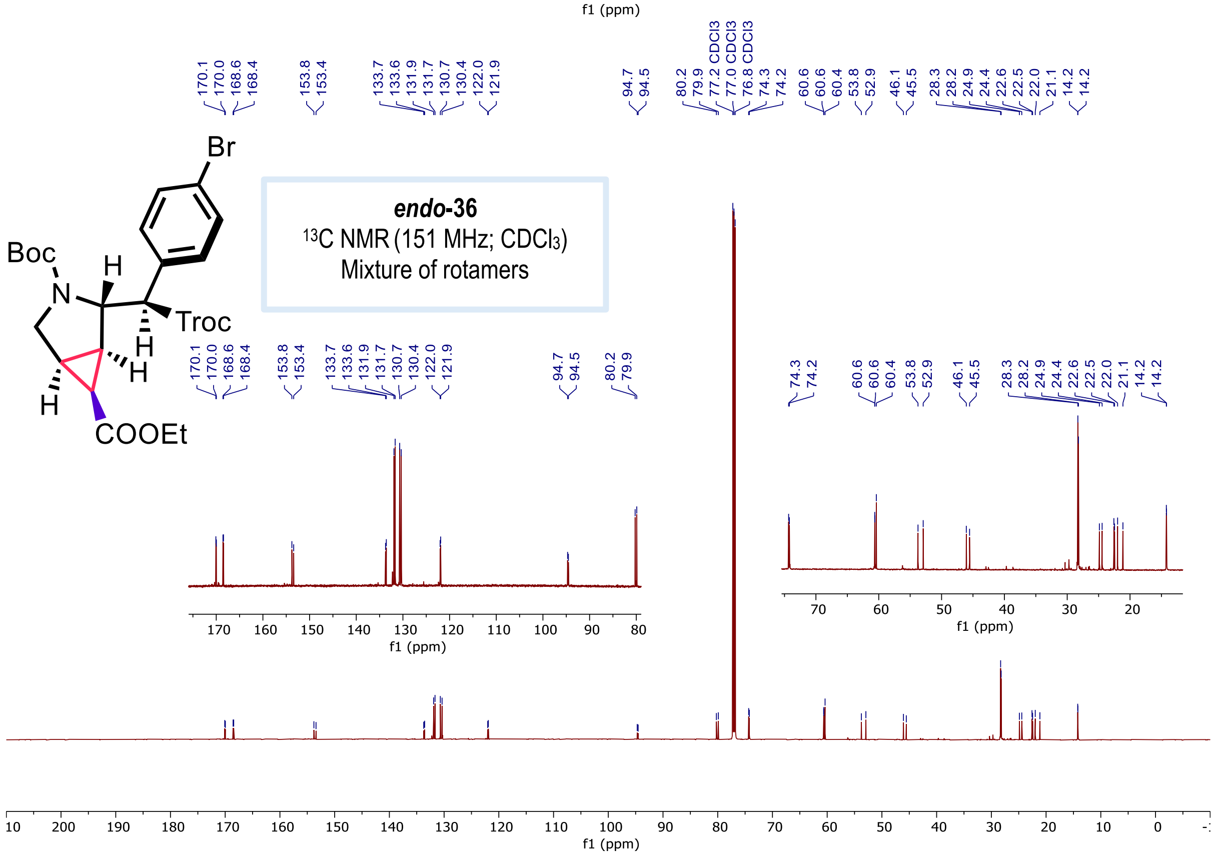
endo-36
¹³C NMR (151 MHz; CDCl3)
Mixture of rotamers
Boc
N
H
Troc
Br
H
H
H
COOEt
f1 (ppm)

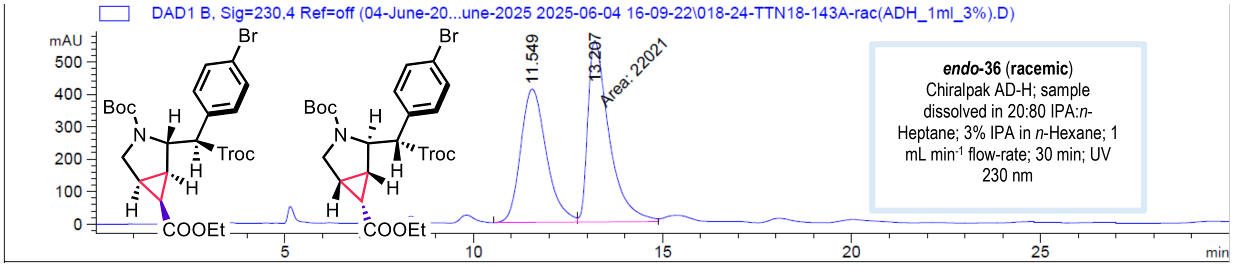

Signal 2: DAD1 B, Sig=230,4 Ref=off

Peak #	RetTime [min]	Type	Width [min]	Area [mAU*s]	Height [mAU]	Area %
1	11.549	BV	0.5659	1.94824e4	411.13239	46.9417
2	13.207	MF	0.6568	2.20210e4	558.76581	53.0583
Totals :				4.15035e4	969.89819	

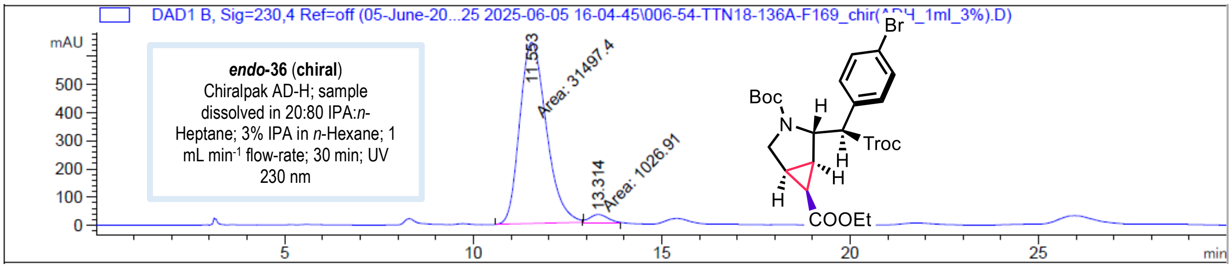

Signal 2: DAD1 B, Sig=230,4 Ref=off

Peak #	RetTime [min]	Type	Width [min]	Area [mAU*s]	Height [mAU]	Area %
1	11.553	MM	0.8172	3.14974e4	642.42200	96.8426
2	13.314	MM	0.5686	1026.91479	30.10030	3.1574
Totals :				3.25244e4	672.52229	

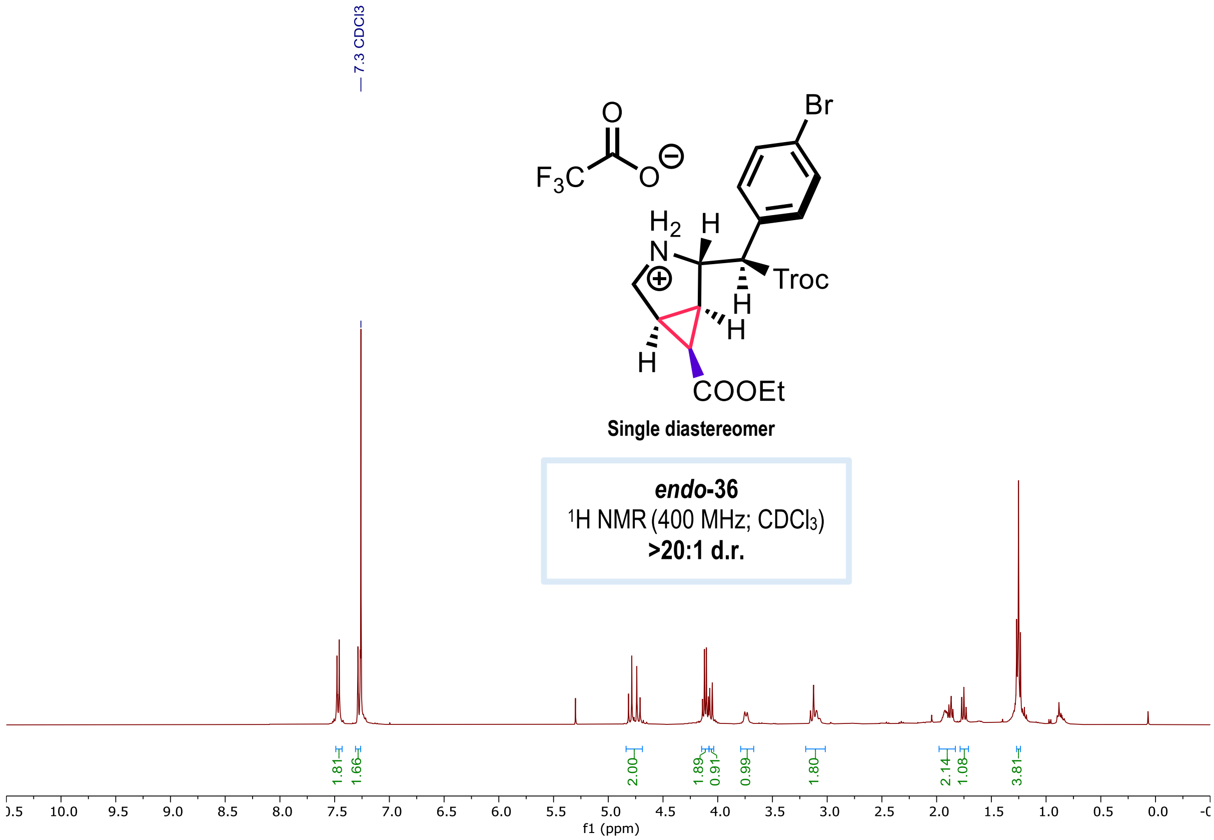

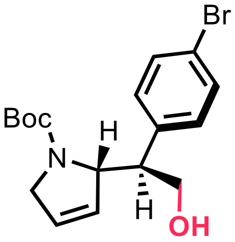

tert-butyl (S)-2-((R)-1-(4-bromophenyl)-2-hydroxyethyl)-2,5-dihydro-1H-pyrrole-1-carboxylate (37)

To a flame-dried 20 mL vial charged with a stir-bar was added tert-butyl (S)-2-((R)-1-(4-bromophenyl)-2-oxo-2-(2,2,2-trichloroethoxy)ethyl)-2,5-dihydro-1H-pyrrole-1-carboxylate (770 mg, 1.50 mL in THF, 1 molar, 1 equiv, 1.50 mmol) and methanol (24.0 mg, 30.3 µL, 0.50 equiv, 750 µmol). The reaction vessel was capped with a septum and a nitrogen balloon. $LiBH_4$ in 2 M THF (65.3 mg, 1.50 mL THF, 2 molar, 2 equiv, 3.00 mmol) was added drop-wise to the reaction solution at room temperature. The mixture was stirred at room temperature for 2.5 hours. After the elapsed time, the reaction solution was quenched by addition of DI water. The aqueous solution was extracted with EtOAc three times, the combined organic layers dried with $MgSO_4$, filtered, and the filtrate concentrated *in vacuo* at 45 °C. The crude was dry-loaded onto SiO_2 and subjected to SiO_2 flash chromatography using hexanes/diethyl ether (Biotage Sfär DLV 50 g column; 0% 5CV → 1% 5CV → 3% 5CV → 7% 5CV → 10% 5CV → 20% 5CV → 25% 5CV → 30% 5CV → 40% 5CV → 60% 5CV → 80% 5CV → 100% 10CV; 100 mL/min flow-rate; 210/230 nm detector; POI elutes at 20 to 30% ether; TLC 1:1 hexanes/ether rf ~0.3 stains with $KMnO_4$). The collected fractions were concentrated *in vacuo* at 45 °C to afford tert-butyl (S)-2-((R)-1-(4-bromophenyl)-2-hydroxyethyl)-2,5-dihydro-1H-pyrrole-1-carboxylate (451 mg, 1.22 mmol, 81.6% yield, 94% ee, >20:1 d.r.) as a pale-caramel-colored oil.

^{1}H NMR (400 MHz, DMSO-d$_6$; as a mixture of rotamers) δ 7.49 (dd, *J* = 7.9, 7.9 Hz, 2H, mixture of rotamers), 7.16 (brd, *J* = 7.9 Hz, 2H, mixture of rotamers), 5.88 – 5.64 (brm, 2H, mixture of rotamers), 4.57 (brm, 2H, mixture of rotamers), 4.11 – 3.97 (brm, 1H, mixture of rotamers), 3.79 – 3.56 (brm, 3H, mixture of rotamers), 3.31 – 3.18 (brm, 1H, mixture of rotamers), 1.50 – 1.41 (overlapping s's, 9H, mixture of rotamers).
^{13}C NMR (151 MHz, DMSO-d$_6$; as a mixture of rotamers) δ 154.3, 153.9, 140.3, 140.1, 131.4, 131.3, 131.3, 127.8, 127.7, 127.2, 127.0, 119.9, 79.6, 79.4, 67.6, 67.2, 60.9, 60.4, 54.6, 54.4, 51.1, 50.5, 28.6.
HRMS (APCI +) calc. mass for $C_{17}H_{23}O_3N^{79}Br$ [M + H]$^+$ 368.0856; obs. mass for $C_{17}H_{23}O_3N^{79}Br$ [M + H]$^+$ 368.0856.
[α]$^{20}_{D}$: -185.0° (c = 0.655 g/100 mL, EtOAc).
SFC (Trefoil CEL2 column; sample dissolved in MeOH; 3% cosolvent of 1:1 MeOH:IPA with 0.2% formic acid; 2.5 mL min^{-1} flow-rate; 20 min; UV 210 nm): retention times of 14.11 min (minor) and 17.29 min (major); 94% ee.
Determination of d.r.: variable temperature ^{1}H NMR; observed >20:1 d.r.

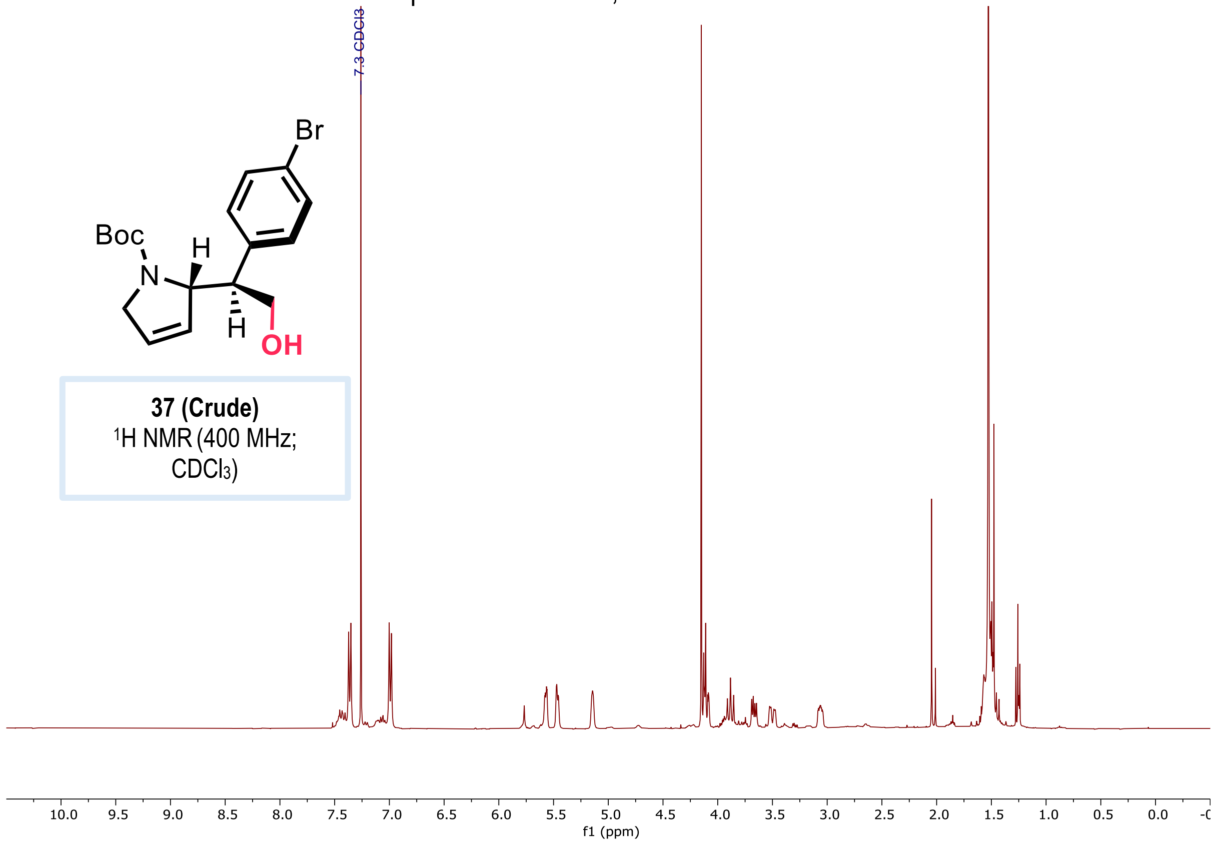

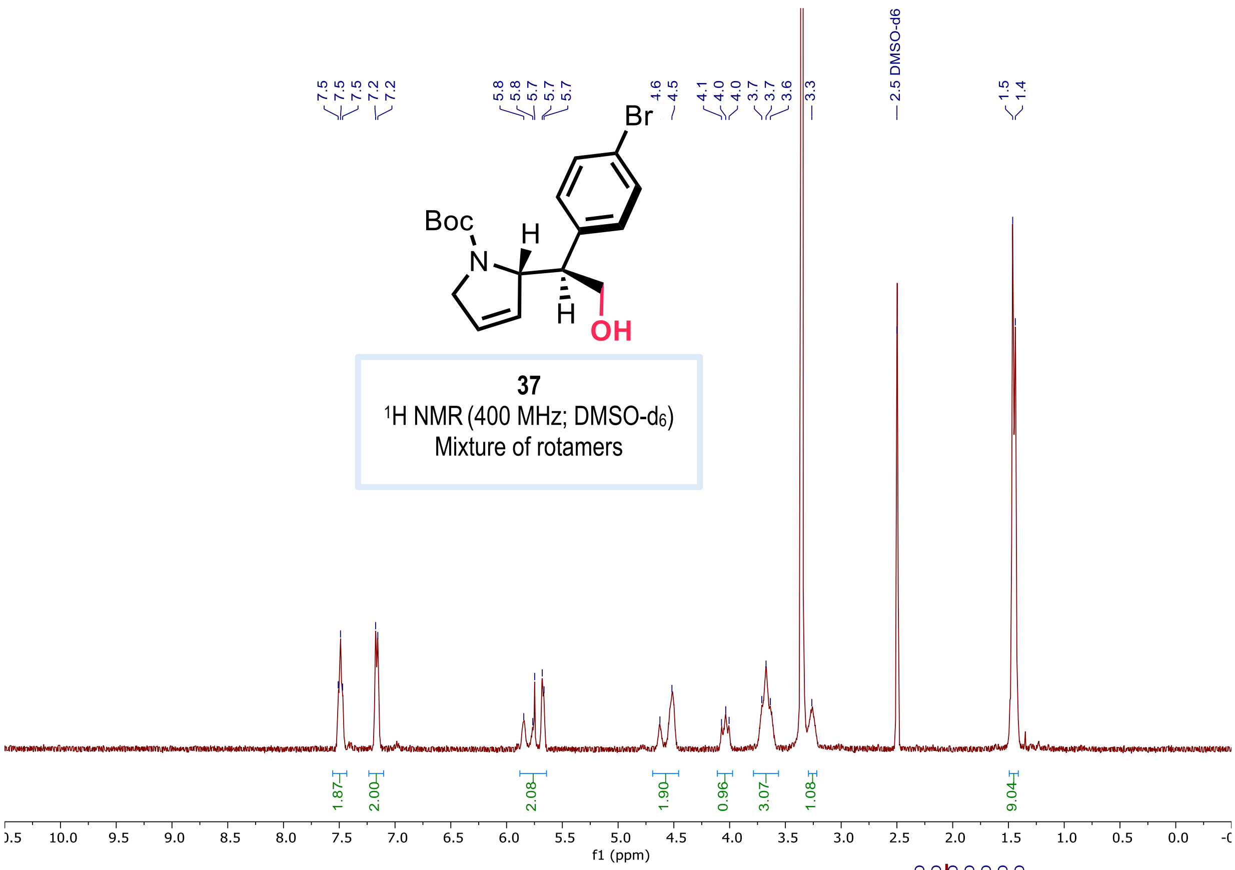
Br
Boc
N
H
H
OH
37
1H NMR (400 MHz; DMSO-d6)
Mixture of rotamers
f1 (ppm)

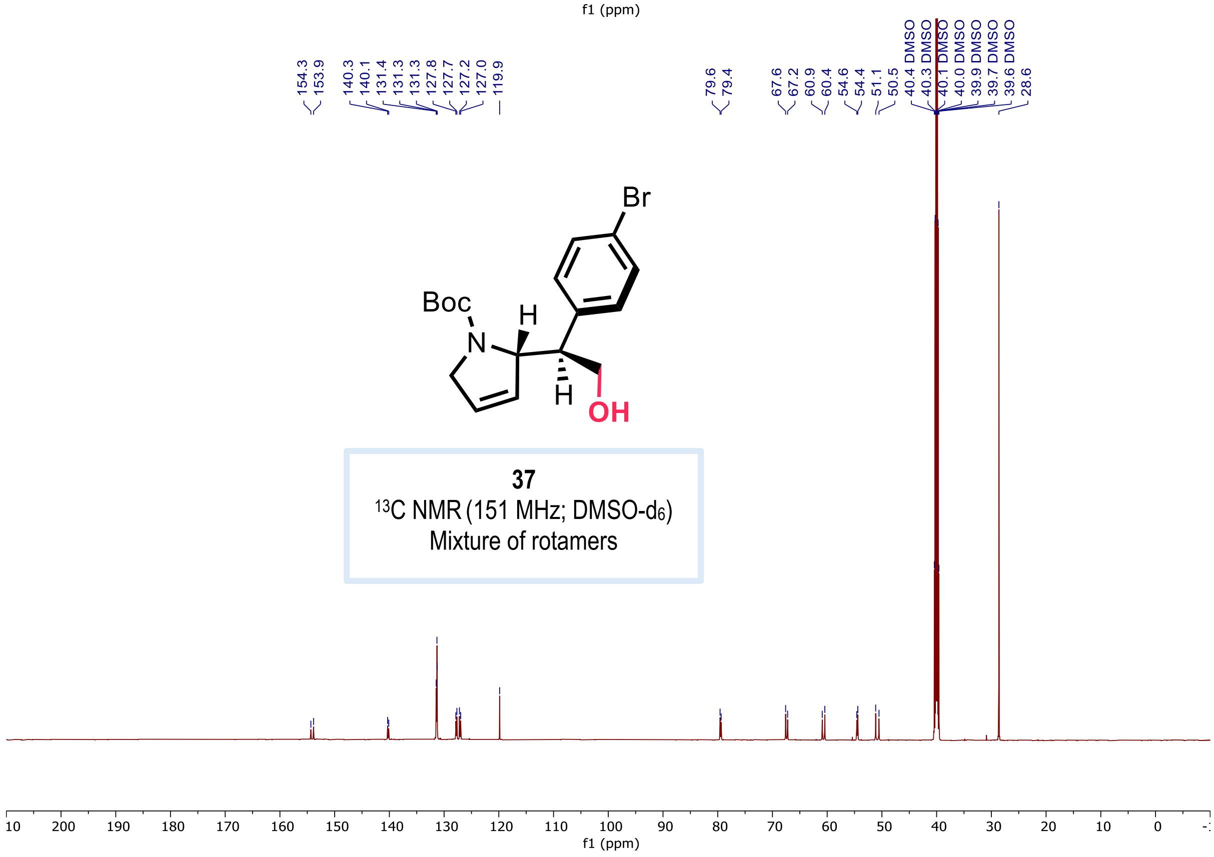
Br
Boc
N
H
H
OH
37
13C NMR (151 MHz; DMSO-d6)
Mixture of rotamers
f1 (ppm)

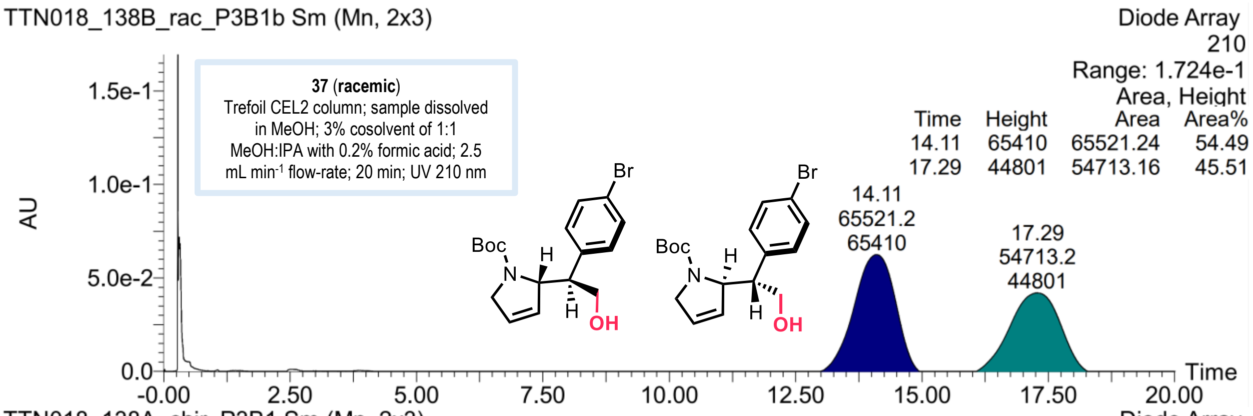

TTN018_138B_rac_P3B1b Sm (Mn, 2x3)
Diode Array
210
Range: 1.724e-1
Area, Height
37 (racemic)
Trefoil CEL2 column; sample dissolved in MeOH; 3% cosolvent of 1:1 MeOH:IPA with 0.2% formic acid; 2.5 mL min-1 flow-rate; 20 min; UV 210 nm
Time Height Area Area%
14.11 65410 65521.24 54.49
17.29 44801 54713.16 45.51
14.11
65521.2
65410
17.29
54713.2
44801
AU
Time

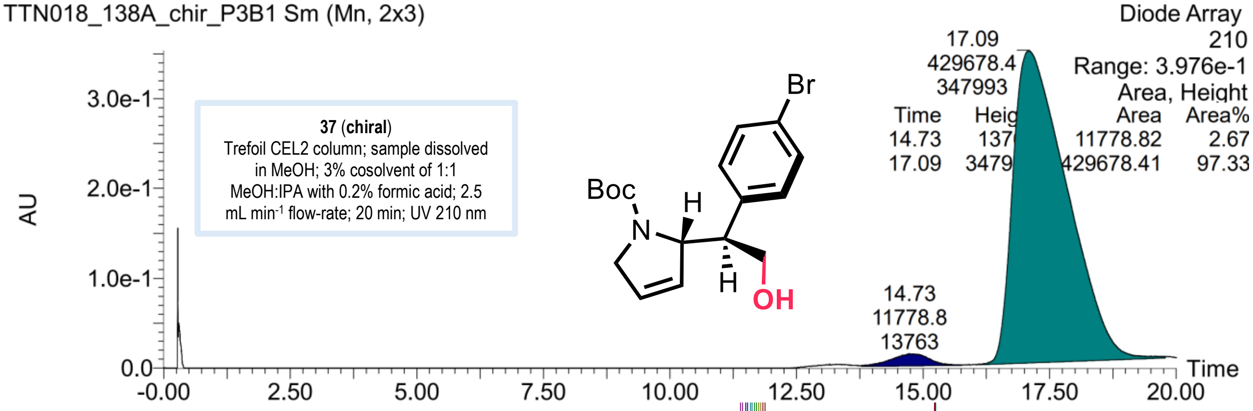

TTN018_138A_chir_P3B1 Sm (Mn, 2x3)
Diode Array
210
Range: 3.976e-1
Area, Height
37 (chiral)
Trefoil CEL2 column; sample dissolved in MeOH; 3% cosolvent of 1:1 MeOH:IPA with 0.2% formic acid; 2.5 mL min-1 flow-rate; 20 min; UV 210 nm
17.09
429678.4
347993
Time Heig Area Area%
14.73 137 11778.82 2.67
17.09 3479 429678.41 97.33
14.73
11778.8
13763
AU
Time

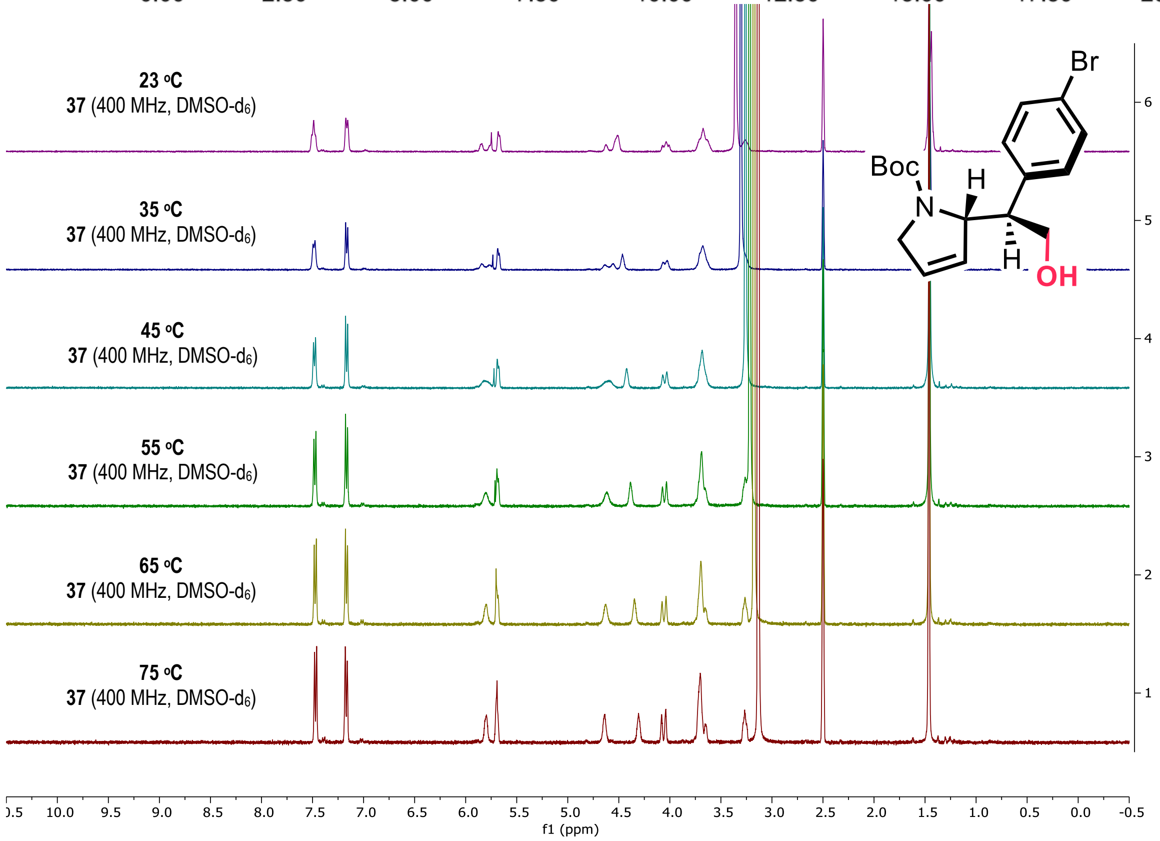

23 °C
37 (400 MHz, DMSO-d6)
35 °C
37 (400 MHz, DMSO-d6)
45 °C
37 (400 MHz, DMSO-d6)
55 °C
37 (400 MHz, DMSO-d6)
65 °C
37 (400 MHz, DMSO-d6)
75 °C
37 (400 MHz, DMSO-d6)
f1 (ppm)

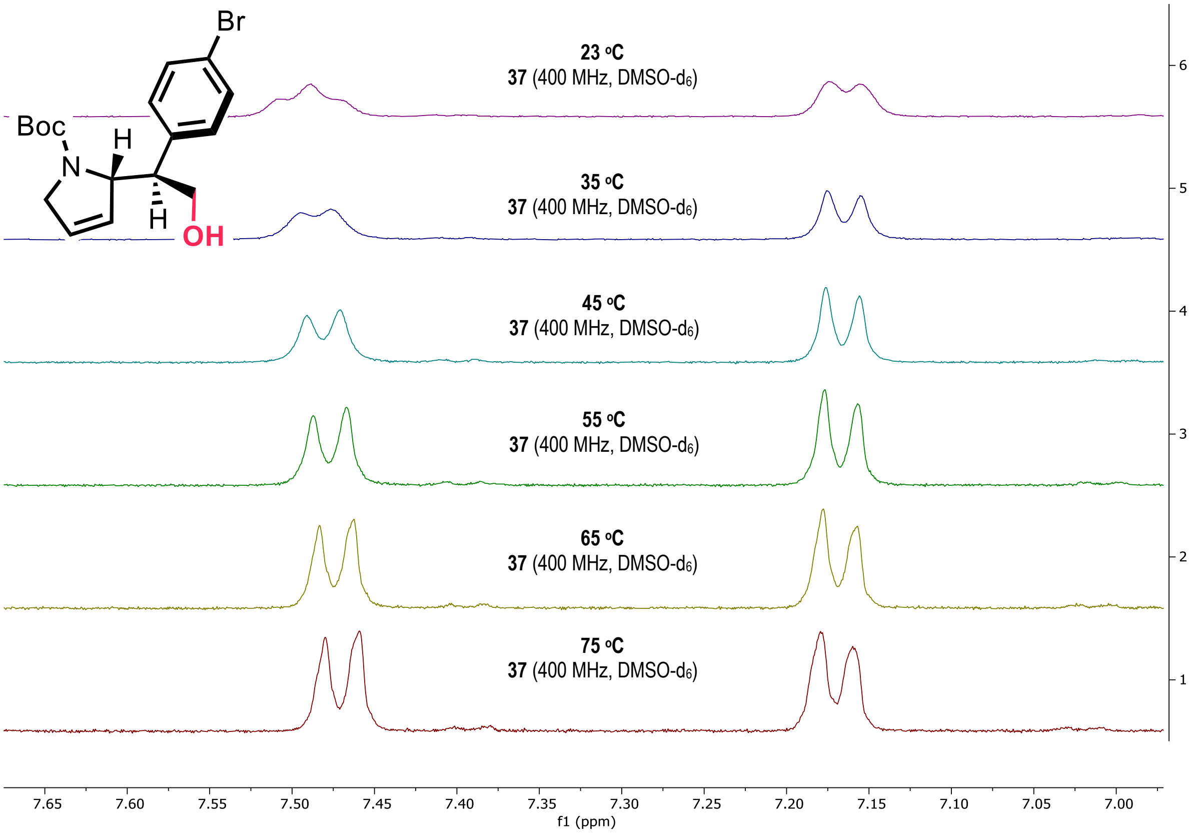

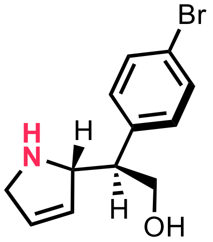

(R)-2-(4-bromophenyl)-2-((S)-2,5-dihydro-1H-pyrrol-2-yl)ethan-1-ol (38)

To a vial charged with a stir-bar was added tert-butyl (S)-2-((R)-1-(4-bromophenyl)-2-hydroxyethyl)-2,5-dihydro-1H-pyrrole-1-carboxylate (200 mg, 1 equiv, 543 µmol), CH_2Cl_2 (46.1 mg, 2.17 mL, 0.25 molar, 1 equiv, 543 µmol), and TFA (619 mg, 418 µL, 10 equiv, 5.43 mmol). The reaction was allowed to react for 20 hours at 25 °C. After the elapsed time, the reaction mixture was neutralized with saturated bicarbonate solution, extracted with EtOAc three times, dried with $MgSO_4$, filtered, and the filtrate concentrated *in vacuo* at 45 °C. The dried material was dissolved in minimal ether and then hexanes to precipitate the product as a brown suspension. The precipitates were allowed to settle and the solution aspirated off, the vial containing the solids concentrated *in vacuo* at 45 °C to (R)-2-(4-bromophenyl)-2-((S)-2,5-dihydro-1H-pyrrol-2-yl)ethan-1-ol (118 mg, 440 µmol, 81.0% yield, >20:1 d.r.) as a brown amorphous powder.

^{1}H NMR (400 MHz, Chloroform-*d*) δ 7.43 (d, *J* = 8.4 Hz, 2H), 7.05 (d, *J* = 8.4 Hz, 2H), 5.95 (dq, *J* = 6.4, 2.2 Hz, 1H), 5.63 (dq, *J* = 6.4, 2.2 Hz, 1H), 4.66 – 4.58 (brm, 1H), 4.18 (dd, *J* = 10.6, 9.2 Hz, 1H), 3.89 – 3.79 (m, 2H), 3.79 – 3.69 (m, 1H), 3.21 – 3.11 (m, 1H).
^{13}C NMR 151 MHz, Chloroform-*d*) δ 139.1, 131.6, 130.2, 129.8, 127.5, 120.7, 69.9, 64.1, 53.3, 49.5.

HRMS (ESI +) calc. mass for $C_{12}H_{15}ON^{79}Br$ $[M + H]^+$ 268.0332; obs. mass for $C_{12}H_{15}ON^{79}Br$ $[M + H]^+$ 268.0338.

$[\alpha]^{20}_D$: -119.3° (c = 0.202 g/100 mL, EtOAc).

Determination of d.r.: ^{1}H NMR (400 MHz, $CDCl_3$) at 23 °C; observed >20:1 d.r.

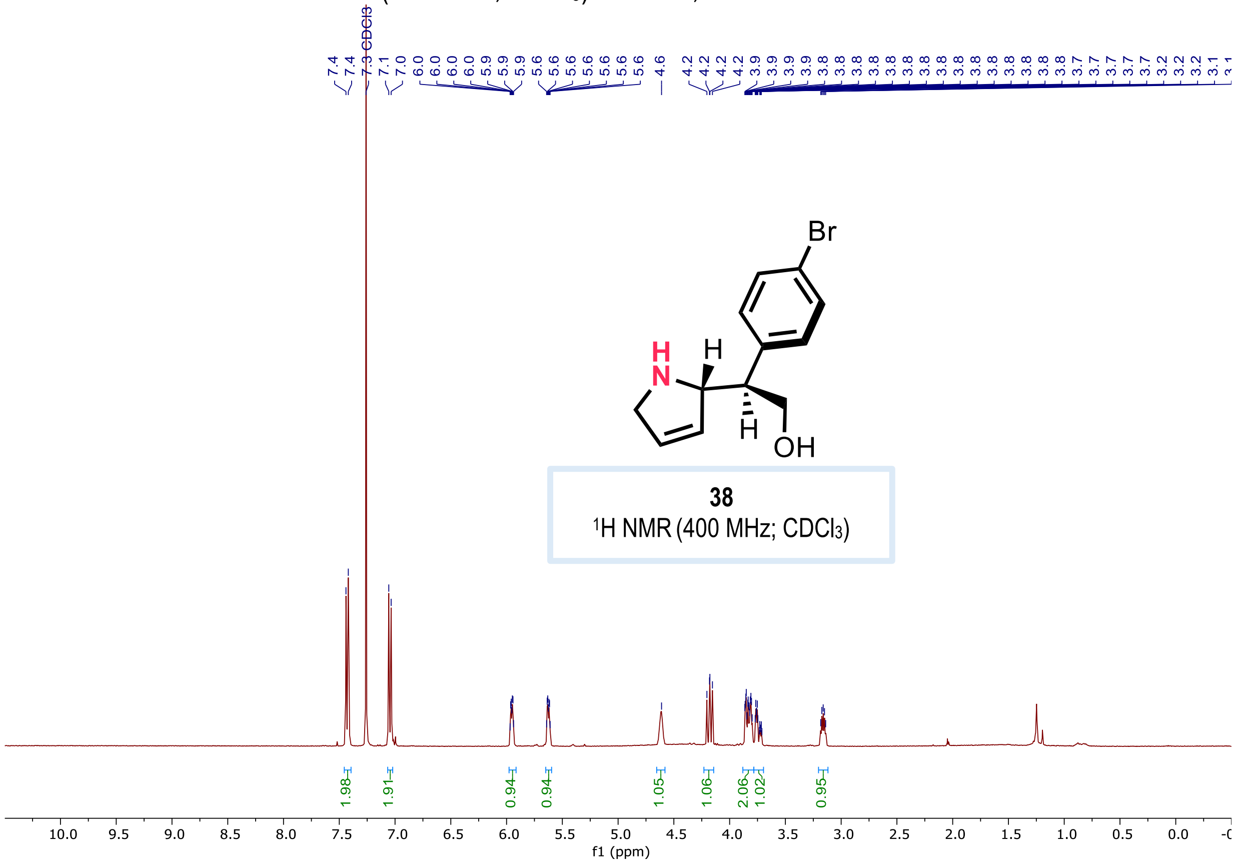

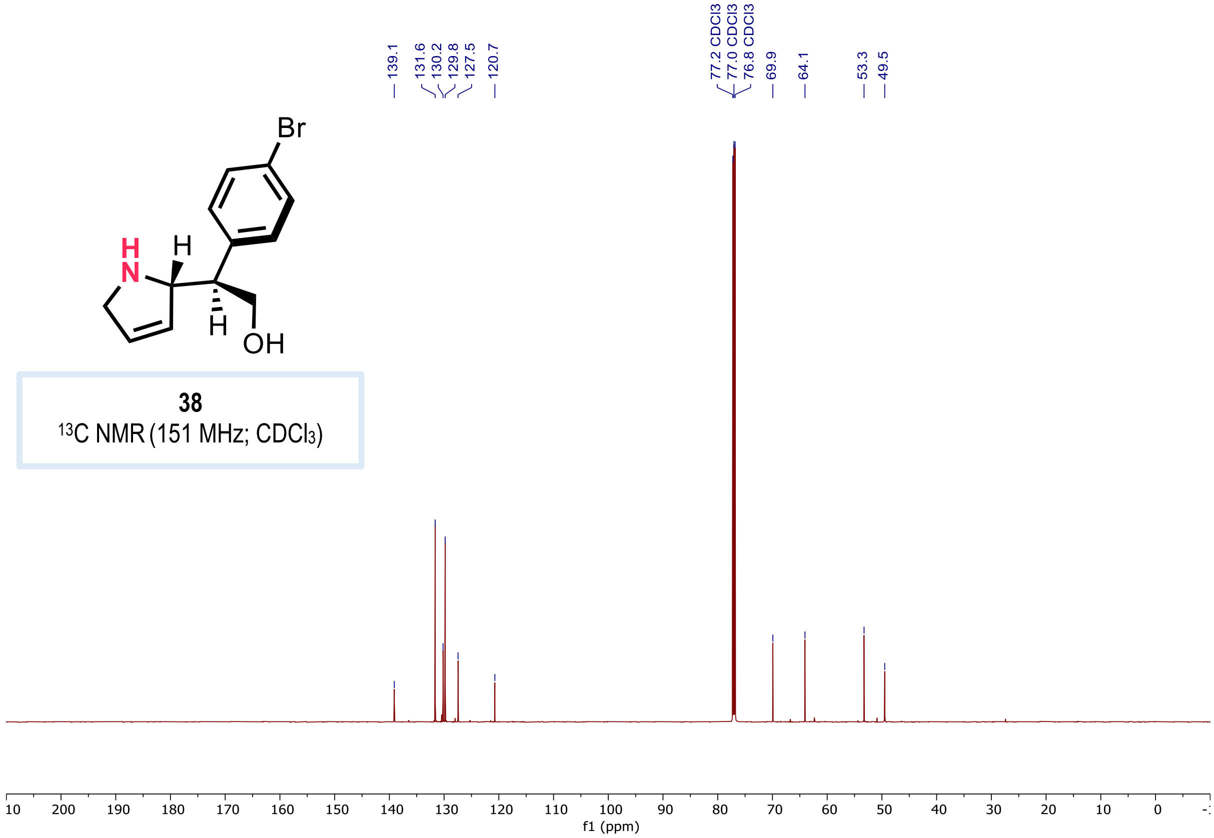

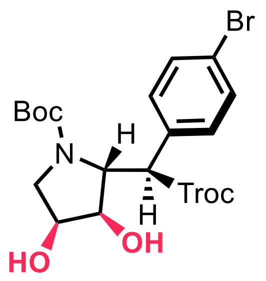

tert-butyl (2R,3R,4S)-2-((R)-1-(4-bromophenyl)-2-oxo-2-(2,2,2-trichloroethoxy)ethyl)-3,4-dihydroxypyrrolidine-1-carboxylate (39)

To a 20 mL vial charged with a stir-bar was added tert-butyl (S)-2-((R)-1-(4-bromophenyl)-2-oxo-2-(2,2,2-trichloroethoxy)ethyl)-2,5-dihydro-1H-pyrrole-1-carboxylate (770 mg, 1 equiv, 1.50 mmol), THF (5.00 mL), 4-methylmorpholine 4-oxide hydrate (304 mg, 1.5 equiv, 2.25 mmol) and then osmium tetroxide in water (1.91 g, 1.91 mL H_2O, 4% Wt, 0.20 equiv, 300 µmol). **Note:** *It is observed that order of addition is important: OsO_4 then NMO results in a black solution and purification leads to leeching of black material; NMO then OsO_4 results in an orange solution and purification is qualitatively cleaner.* The reaction solution was allowed to react at 25 °C for 20 hours. After the elapsed time, the reaction was quenched by addition of saturated sodium bisulfite solution. The reaction solution was extracted with EtOAc three times (sonication helps break the emulsion), the combined organic layer dried with $MgSO_4$, filtered, and the filtrate concentrated *in vacuo* at 45 °C. The crude was dry-loaded onto SiO_2 and subjected to SiO_2 flash chromatography using pentane/diethyl ether (Biotage Sfär DLV 25 g column as the running column and Biotage Sfär DLV 25 g column for the dry-load; 0% 5CV → 0 to 100% 10CV → 100% 5CV; 150 mL/min flow-rate; 210/230 nm detector; POI elutes at around 80% ether). The collected fractions were concentrated *in vacuo* at 45 °C to afford tert-butyl (2R,3R,4S)-2-((R)-1-(4-bromophenyl)-2-oxo-2-(2,2,2-trichloroethoxy)ethyl)-3,4-dihydroxypyrrolidine-1-carboxylate (631 mg, 1.15 mmol, 76.8% yield, 95% ee, >20:1 d.r.) initially as a clear oil that turned into an off-white foam under vacuum overnight.

^{1}H NMR (600 MHz, DMSO-*d*$_6$; as a 0.55:0.45 mixture of rotamers) δ 7.54 (d, *J* = 8.2 Hz, 0.9H, minor rotamer), 7.51 (d, *J* = 8.2 Hz, 1.1H, major rotamer), 7.29 (d, *J* = 8.2 Hz, 2H, mixture of rotamers), 5.10 – 4.83 (m, 4H, mixture of rotamers), 4.34 – 4.24 (m, 0.45H, minor rotamer), 4.24 – 4.18 (m, 0.55H, major rotamer), 4.13 (d, *J* = 5.5 Hz, 0.55H, major rotamer), 4.08 (d, *J* = 8.8 Hz, 0.45H, minor rotamer), 4.02 (d, *J* = 8.8 Hz, 0.55H, major rotamer), 3.98 – 3.92 (m, 0.45H, minor rotamer), 3.87 – 3.79 (m, 1H, mixture of rotamers), 3.29 – 3.04 (m, 2H, mixture of rotamers), 1.32 (s, 4.95H, major rotamer), 1.17 (s, 4.05H, minor rotamer).
^{13}C NMR (151 MHz, DMSO-*d*$_6$; as a mixture of rotamers) δ 170.5, 154.9, 154.3, 134.7, 134.2, 132.4, 131.7, 131.6, 131.2, 121.3, 95.5, 95.5, 79.7, 79.4, 79.2, 79.1, 74.4, 74.0, 73.9, 73.6, 69.3, 66.5, 65.5, 51.9, 51.8, 51.3, 51.1, 40.5, 28.6, 28.4, 28.1.
HRMS (ESI +) calc. mass for $C_{19}H_{24}O_6N^{79}Br^{35}Cl_3$ $[M + H]^+$ 545.9847; obs. mass for $C_{19}H_{24}O_6N^{79}Br^{35}Cl_3$ $[M + H]^+$ 545.9849.
$[\alpha]^{20}_D$: -23.4° (c = 0.175 g/100 mL, EtOAc).
SFC (Regis (S,S) Whelk-O 1 Kromasil column; sample dissolved in MeOH; 3% cosolvent of 1:1 MeOH:IPA with 0.2% formic acid; 2.5 mL min^{-1} flow-rate; 15 min; UV 230 nm): retention times of 12.38 min (minor) and 13.78 min (major); 95% ee.
Determination of d.r.: variable temperature ^{1}H NMR; observed >20:1 d.r.

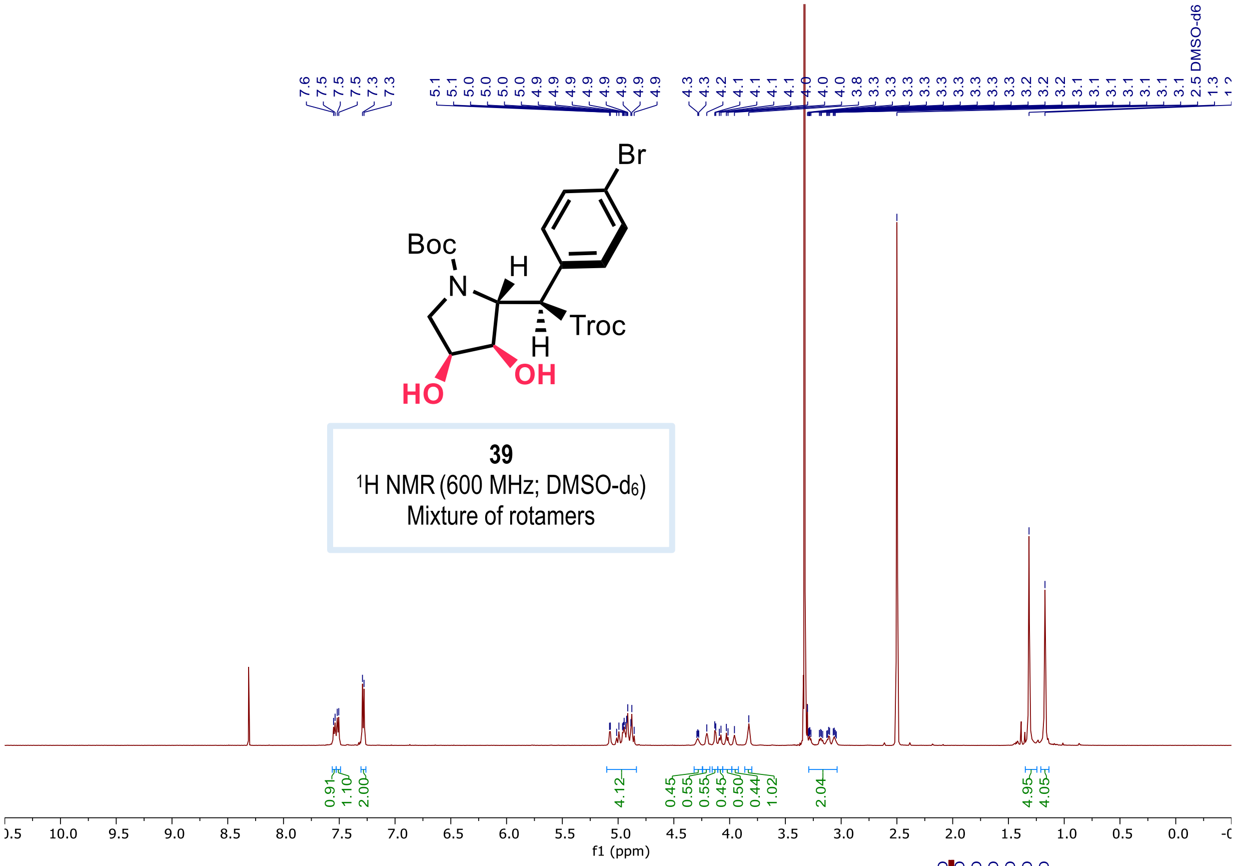

39
^1H NMR (600 MHz; DMSO-d_6)
Mixture of rotamers

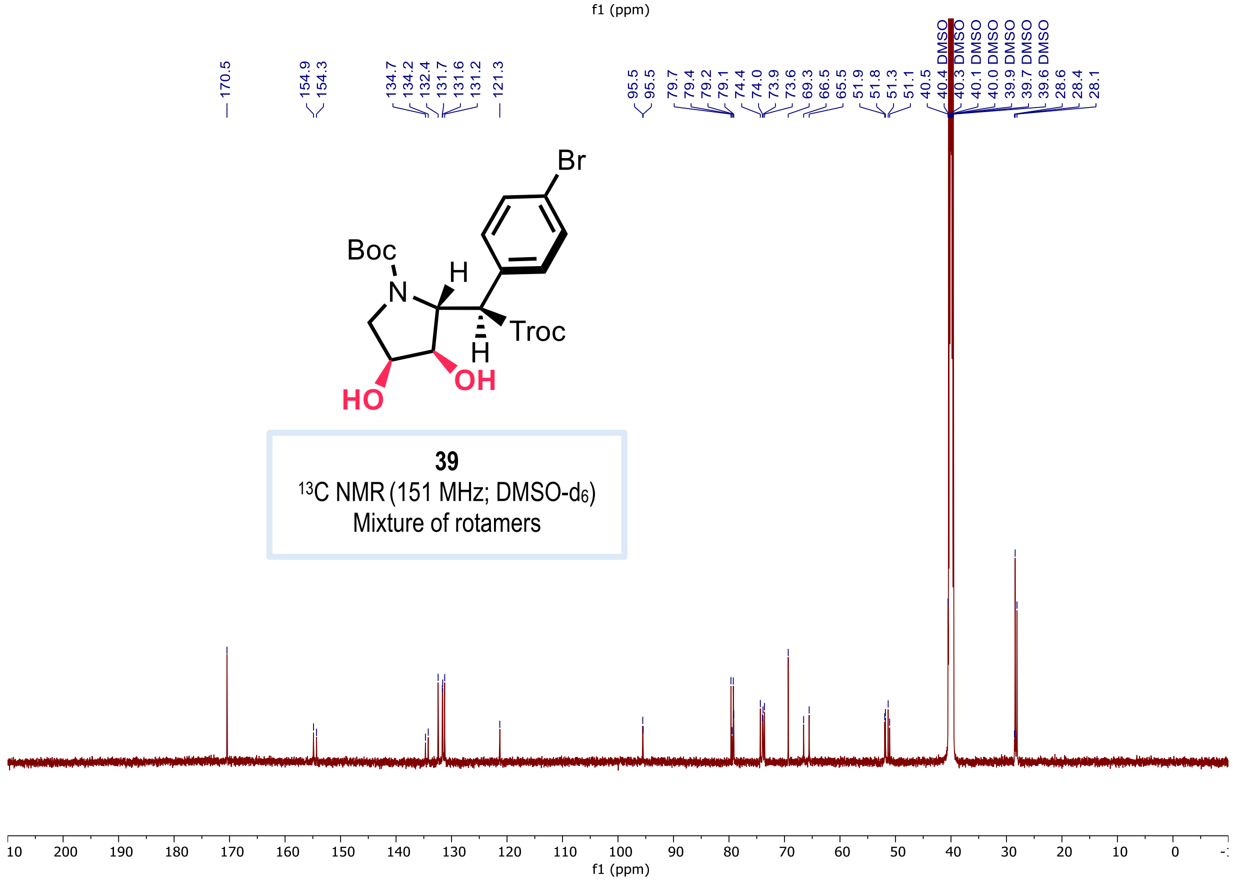

39
^{13}C NMR (151 MHz; DMSO-d_6)
Mixture of rotamers

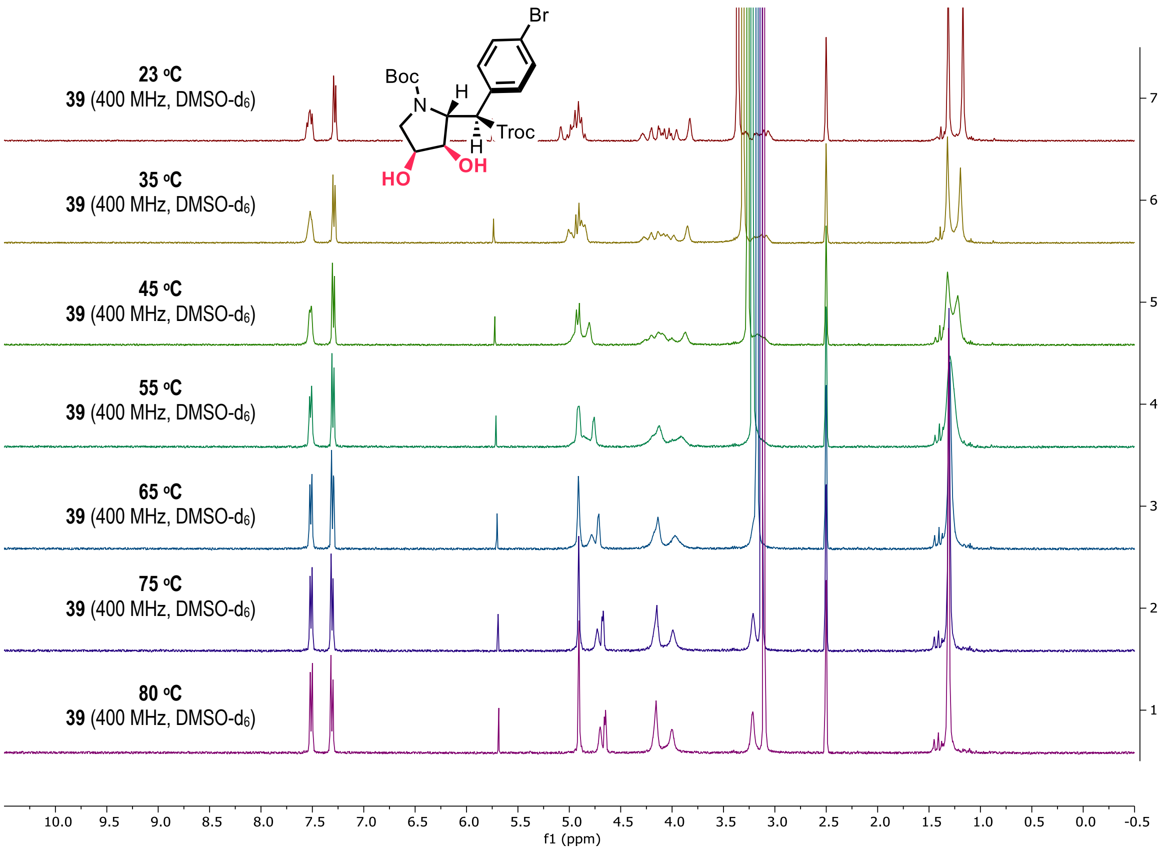
23 °C
39 (400 MHz, DMSO-d6)
35 °C
39 (400 MHz, DMSO-d6)
45 °C
39 (400 MHz, DMSO-d6)
55 °C
39 (400 MHz, DMSO-d6)
65 °C
39 (400 MHz, DMSO-d6)
75 °C
39 (400 MHz, DMSO-d6)
80 °C
39 (400 MHz, DMSO-d6)
Br
Boc
N
H
Troc
H
HO
OH
f1 (ppm)

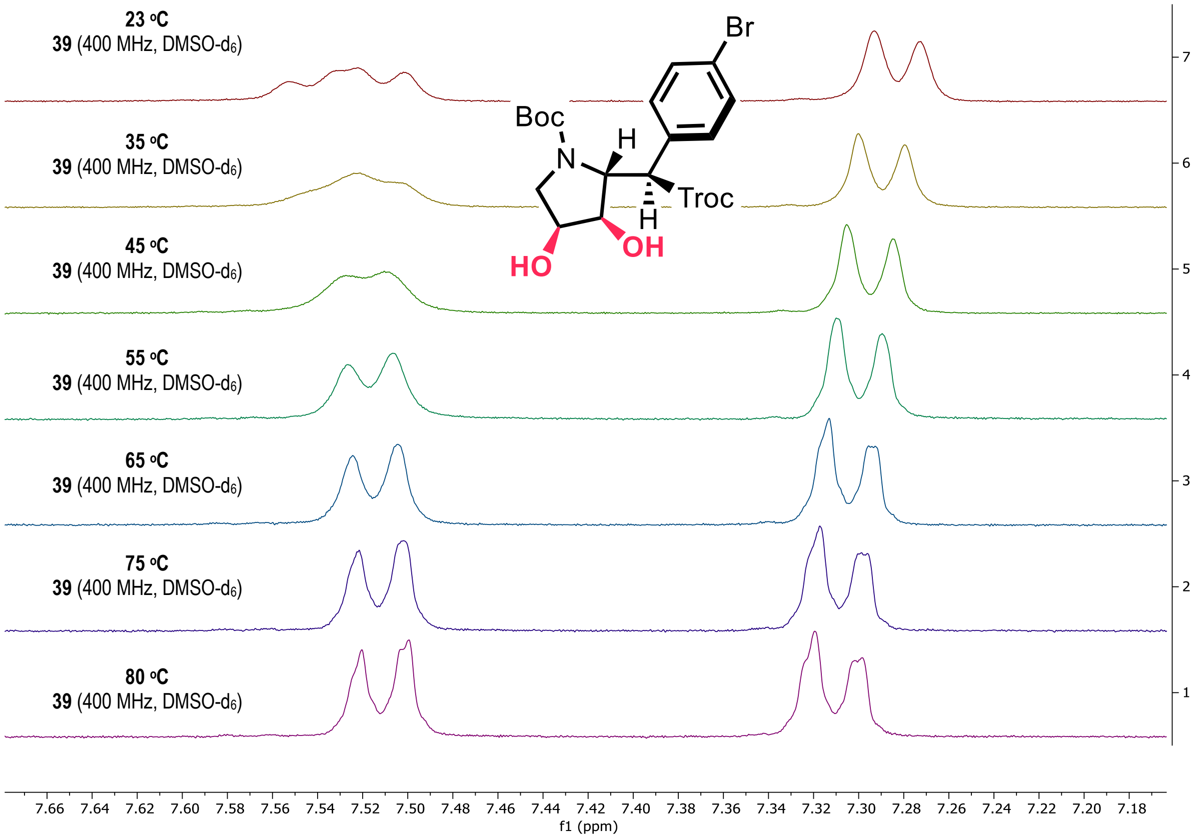
23 °C
39 (400 MHz, DMSO-d6)
35 °C
39 (400 MHz, DMSO-d6)
45 °C
39 (400 MHz, DMSO-d6)
55 °C
39 (400 MHz, DMSO-d6)
65 °C
39 (400 MHz, DMSO-d6)
75 °C
39 (400 MHz, DMSO-d6)
80 °C
39 (400 MHz, DMSO-d6)
Br
Boc
N
H
Troc
H
HO
OH
f1 (ppm)

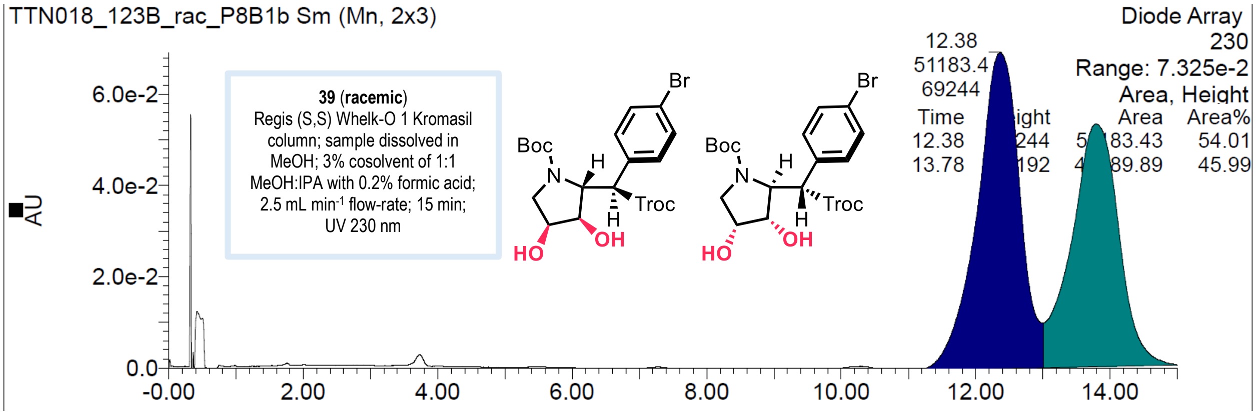

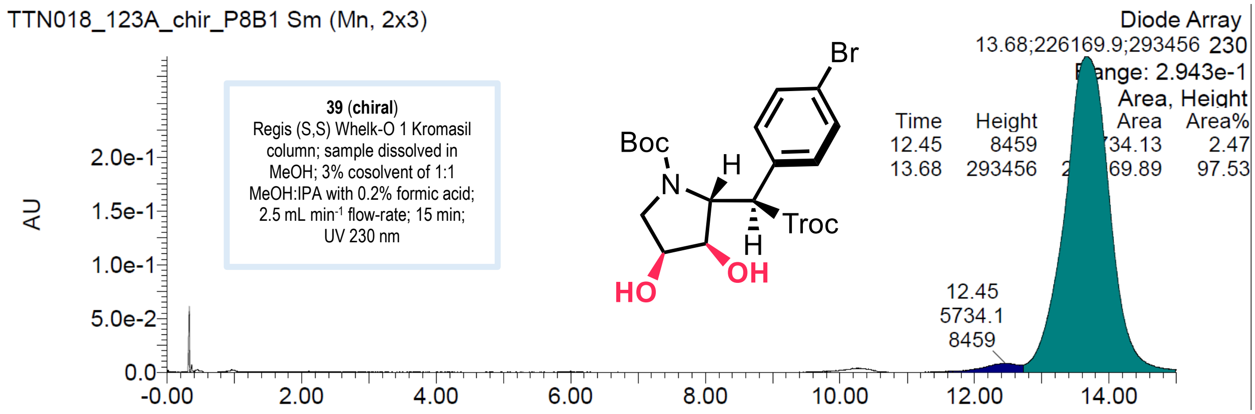

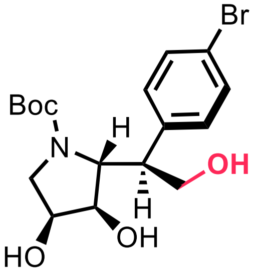

tert-butyl (2R,3R,4S)-2-((R)-1-(4-bromophenyl)-2-hydroxyethyl)-3,4-dihydroxypyrrolidine-1-carboxylate (40)

To a flame-dried 20 mL vial charged with a stir-bar was added tert-butyl (2R,3R,4S)-2-((R)-1-(4-bromophenyl)-2-oxo-2-(2,2,2-trichloroethoxy)ethyl)-3,4-dihydroxypyrrolidine-1-carboxylate (201 mg, 367 µL in THF, 1 molar, 1 equiv, 367 µmol) and methanol (5.88 mg, 7.42 µL, 0.50 equiv, 184 µmol). The reaction vessel was capped with a septum and nitrogen balloon. $LiBH_4$ in 2M THF (48.0 mg, 1.10 mL THF, 2 molar, 6 equiv, 2.20 mmol) was added drop-wise to the reaction solution. The mixture was stirred at 25 °C for 2.5 hours. After the elapsed time, the reaction solution was quenched by addition of saturated sodium potassium tartrate solution followed by 1M HCl solution and then saturated ammonium chloride solution until there is no evidence of bubbling. The aqueous solution was extracted with EtOAc three times, the organic layers dried with $MgSO_4$, filtered, and the filtrate concentrated *in vacuo* at 45 ^{o}C. The crude was dry-loaded onto SiO_2 and subjected to SiO_2 flash chromatography pentane/EtOAc (Biotage Sfär DLV 10 g column as the running-column and Biotage Sfär DLV 10 g column for the dry-load; 0% 3CV → 0 to 100 % 3CV → 100% 10 CV; 150 mL/min flow-rate; 254/280 nm detector; the POI eluted at around 50% to 100% EtOAc; TLC - product Rf in 100% EtOAc is 0.4 Rf). The collected fractions were concentrated *in vacuo* at 50 ^{o}C and then subjected to Kugelrohr distillation at 65 ^{o}C under 1 torr for at least an hour to remove

residual solvent to afford tert-butyl (2R,3R,4S)-2-((R)-1-(4-bromophenyl)-2-hydroxyethyl)-3,4-dihydroxypyrrolidine-1-carboxylate (127 mg, 316 μmol, 86.0% yield, >20:1 d.r.) as a pale-yellow viscous oil.

^{1}H NMR (400 MHz, DMSO-*d*$_6$; as a mixture of rotamers) δ 7.47 (d, *J* = 8.0 Hz, 2H, mixture of rotamers), 7.15 (brd, *J* = 8.0 Hz, 2H, mixture of rotamers), 4.89 – 4.56 (brm, 3H, mixture of rotamers), 3.95 – 3.47 (brm, 5H, mixture of rotamers), 3.13 – 2.90 (brm, 3H, mixture of rotamers), 1.41 – 1.30 (overlapping s's, 9H, mixture of rotamers)
^{13}C NMR (151 MHz, DMSO-*d*$_6$; as a mixture of rotamers) δ 155.7, 154.8, 140.8, 140.3, 131.6, 131.2, 120.0, 119.9, 79.1, 74.0, 69.7, 69.4, 67.0, 65.8, 63.0, 62.5, 51.9, 51.5, 50.1, 28.5, 28.4.
HRMS (ESI +) calc. mass for $C_{17}H_{25}O_5N^{79}Br$ $[M + H]^+$ 402.0911; obs. mass for $C_{17}H_{25}O_5N^{79}Br$ $[M + H]^+$ 402.0910.
$[\alpha]^{20}_D$: -80.6° (c = 0.170 g/100 mL, EtOAc).
Determination of d.r.: variable temperature ^{1}H NMR; observed >20:1 d.r.

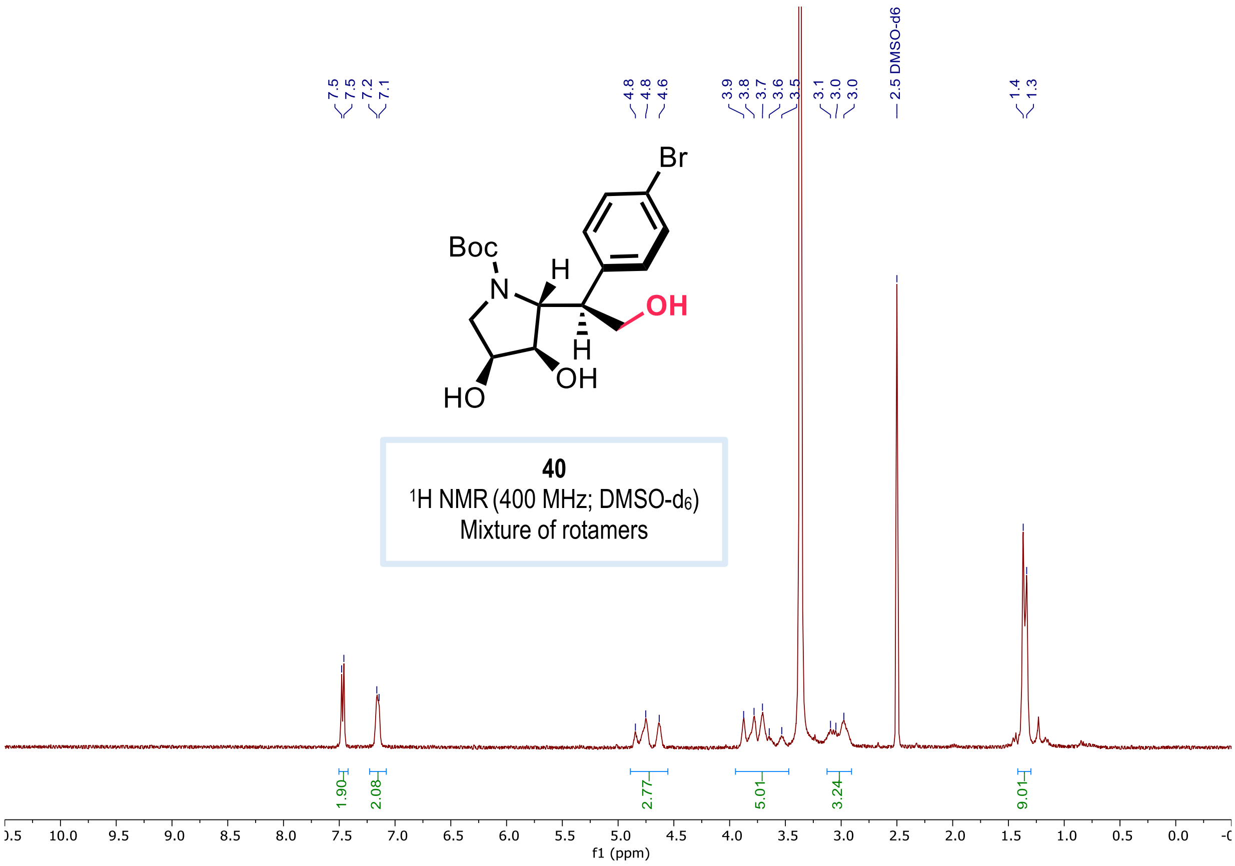

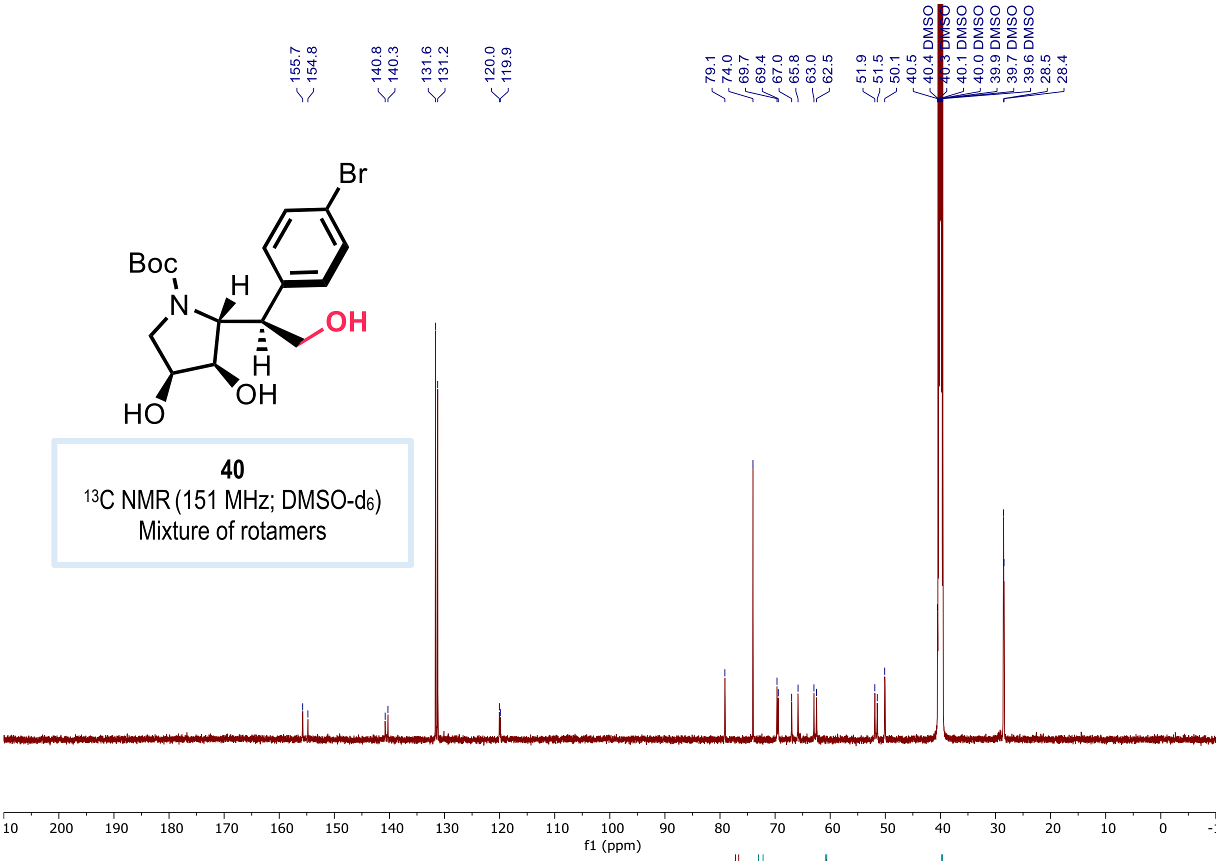
155.7
154.8
140.8
140.3
131.6
131.2
120.0
119.9
79.1
74.0
69.7
69.4
67.0
65.8
63.0
62.5
51.9
51.5
50.1
40.5
40.4 DMSO
40.3 DMSO
40.1 DMSO
40.0 DMSO
39.9 DMSO
39.7 DMSO
39.6 DMSO
28.5
28.4
Br
Boc
N
H
OH
H
OH
HO
40
13C NMR (151 MHz; DMSO-d6)
Mixture of rotamers
f1 (ppm)

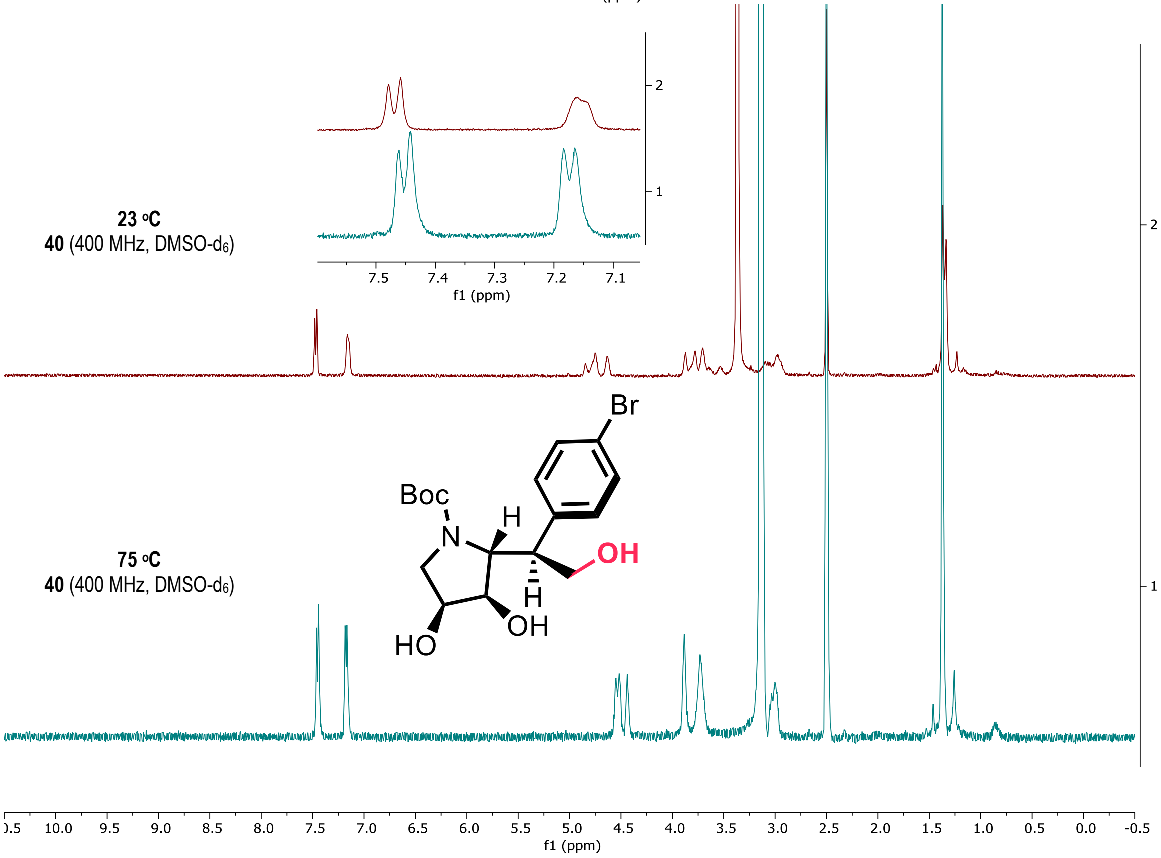
23 °C
40 (400 MHz, DMSO-d6)
75 °C
40 (400 MHz, DMSO-d6)
Br
Boc
N
H
OH
H
OH
HO
f1 (ppm)
f1 (ppm)

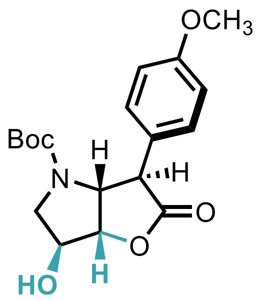

tert-butyl (3R,3aR,6S,6aS)-6-hydroxy-3-(4-methoxyphenyl)-2-oxohexahydro-4H-furo[3,2-b]pyrrole-4-carboxylate (41)

[Epoxidation] - To a flame-dried RBF charged with a stir-bar was added tert-butyl (S)-2-((R)-1-(4-methoxyphenyl)-2-oxo-2-(2,2,2-trichloroethoxy)ethyl)-2,5-dihydro-1H-pyrrole-1-carboxylate (38.3 g, 1 equiv, 82.4 mmol), dichloromethane (7.00 g, 330 mL, 0.25 molar, 1 equiv, 82.4 mmol), and then mCPBA (56.9 g, 75% Wt, 3 equiv, 247 mmol). The reaction vessel was sealed and topped with a nitrogen balloon. The reaction was stirred for 18 hours at 25 °C. After the elapsed time, the reaction solution was quenched by transferring the reaction solution slowly to a 1 L RBF of 500 mL saturated sodium metabisulfite solution, washed with saturated sodium metabisulfite three times, and then the suspension vacuum filtered through a plug of celite. The organic filtrate was then washed with saturated bicarbonate solution three times, and then the organic layer was dried with $MgSO_4$, vacuum filtered through a plug of celite washing with dichloromethane, and the filtrate concentrated *in vacuo* at 55 °C to obtain tert-butyl (1R,2R,5S)-2-((R)-1-(4-methoxyphenyl)-2-oxo-2-(2,2,2-trichloroethoxy)ethyl)-6-oxa-3-azabicyclo[3.1.0]hexane-3-carboxylate **(S16)** as a yellow amorphous powder. The material was pushed forwards to the next step without purification.

[γ-lactonization] - To a flame-dried RBF charged with a stir-bar was added crude tert-butyl (1R,2R,5S)-2-((R)-1-(4-methoxyphenyl)-2-oxo-2-(2,2,2-trichloroethoxy)ethyl)-6-oxa-3-azabicyclo[3.1.0]hexane-3-carboxylate, THF (5.94 g, 206 mL, 0.4 molar, 1 equiv, 82.4 mmol), potassium dihydrogen phosphate solution (potassium phosphate, monobasic solution) (28.0 g, 206 mL, 1 molar in DI Water, 2.5 equiv, 206 mmol), and then zinc dust (<10 μm particle size) (53.9 g, 10 equiv, 824 mmol). The reaction mixture was topped with a glass fritted adapter (to vent off volatiles) and stirred vigorously at 25 °C for 18 hours.

After the elapsed time, the reaction mixture was vacuum filtered through a plug of celite washing with EtOAc, the filtrate diluted with DI water and EtOAc, extracted with EtOAc three times, the combined organics dried with $MgSO_4$, vacuum filtered through a plug of celite, and the filtrate concentrated *in vacuo* at 55 °C.

The crude was dry-loaded onto SiO_2 and then subjected to SiO_2 flash chromatography using hexanes/diethyl ether (100 g Biotage Sfär DLV column as the running-column and 200 g Biotage Sfär DLV column for the dry-load; 100 g KP-Sil setting; 150 mL/min flow rate; 210/230 nm detector; 0% 10CV → 1% 3CV → 3% 3CV → 5% 3CV → 10% 3CV → 15% 3CV → 30% 3CV → 50% 3CV → 75% 3CV → 100% 10CV; TLC conditions 100% ether POI Rf is ~0.6 rf; POI elutes at around 100% ether, byproduct elutes at 75% ether).

The collected fractions were concentrated *in vacuo* at 55 °C to afford tert-butyl (3R,3aR,6S,6aS)-6-hydroxy-3-(4-methoxyphenyl)-2-oxohexahydro-4H-furo[3,2-b]pyrrole-4-carboxylate (21 g, 60 mmol, 73% yield, >20:1 d.r.) as a white foam.

^{1}H NMR (800 MHz, DMSO-*d*$_6$; as a 0.65:0.35 mixture of rotamers) δ 7.23 (d, *J* = 8.7 Hz, 2H, mixture of rotamers), 6.97 – 6.94 (m, 2H, mixture of rotamers), 5.62 (d, *J* = 4.1 Hz, 0.65H, major rotamer), 5.61 (d, *J* = 4.1 Hz, 0.35H, minor rotamer), 5.01 (d, *J* = 5.2 Hz, 0.65H, major rotamer), 4.96 (d, *J* = 5.2 Hz, 0.35H, minor rotamer), 4.37 (d, *J* = 5.2 Hz, 0.65H, major rotamer), 4.35 (d, *J* = 5.2 Hz, 0.35H, minor rotamer), 4.31 (t, *J* = 4.1 Hz, 1H, mixture of rotamers), 3.91 (brm, 0.65H, major rotamer), 3.87 (brm, 0.35H, minor

rotamer), 3.75 (overlapping s's, 3H, mixture of rotamers), 3.52 (t, J = 12.0, Hz, 1H, mixture of rotamers), 3.46 (dd, J = 12.0, 3.9 Hz, 0.35H, minor rotamer), 3.43 (dd, J = 12.0, 3.9 Hz, 0.65H, major rotamer), 1.43 (s, 3.15H, minor rotamer), 1.34 (s, 5.85H, major rotamer).

^{13}C NMR (151 MHz, DMSO-d_6; as a mixture of rotamers) δ 175.9, 158.7, 153.7, 153.0, 128.8, 128.7, 127.9, 127.5, 114.4, 114.4, 85.8, 84.8, 79.7, 79.6, 70.7, 70.2, 64.2, 64.0, 55.2, 52.7, 52.6, 52.0, 51.8, 28.1, 28.0.

HRMS (ESI -) calc. mass for $C_{18}H_{22}O_6N$ $[M-H]^-$ 348.1453; obs. mass for $C_{18}H_{22}O_6N$ $[M-H]^-$ 348.1451.

$[\alpha]^{20}_D$: +21.4° (c = 0.370 g/100 mL, EtOAc).

Determination of d.r.: observed >20:1 d.r. with ^{1}H NMR analysis at 23 °C in DMSO-d_6.

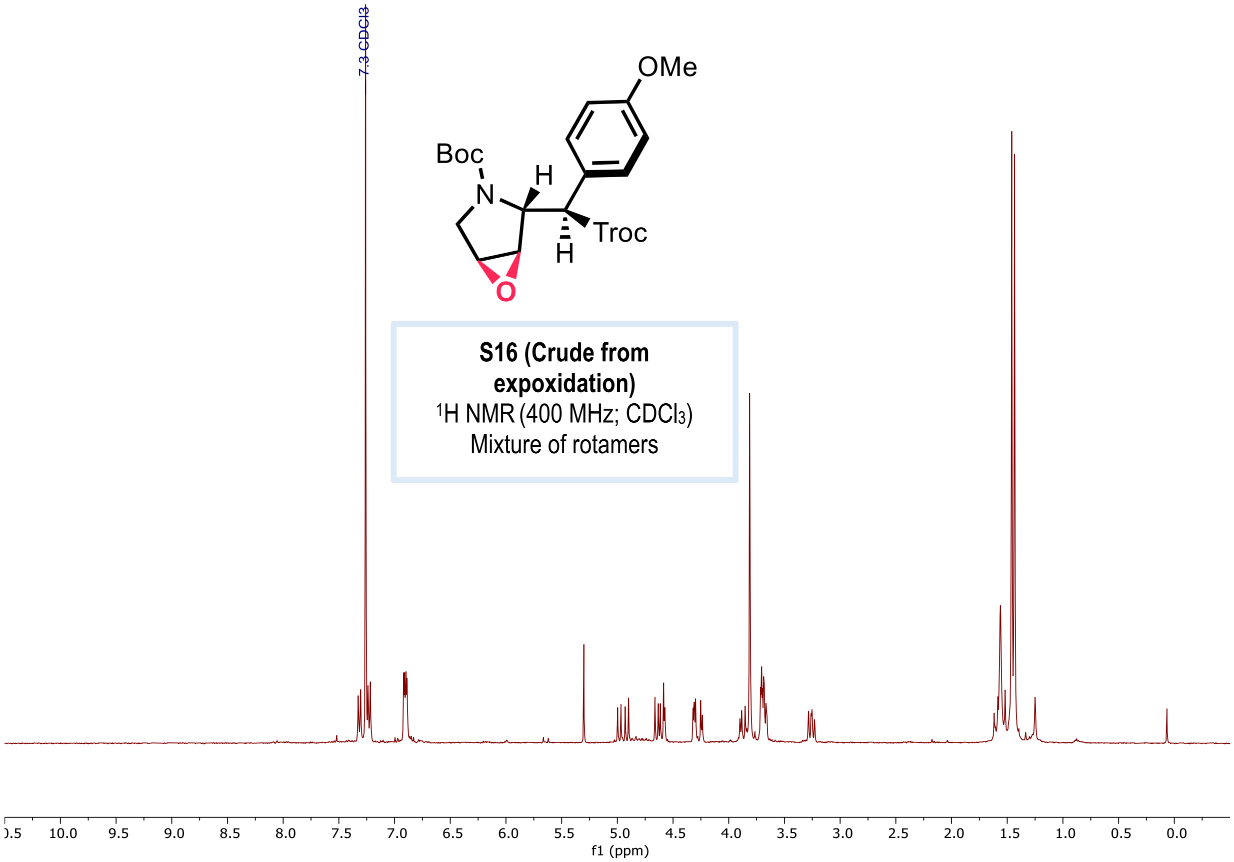

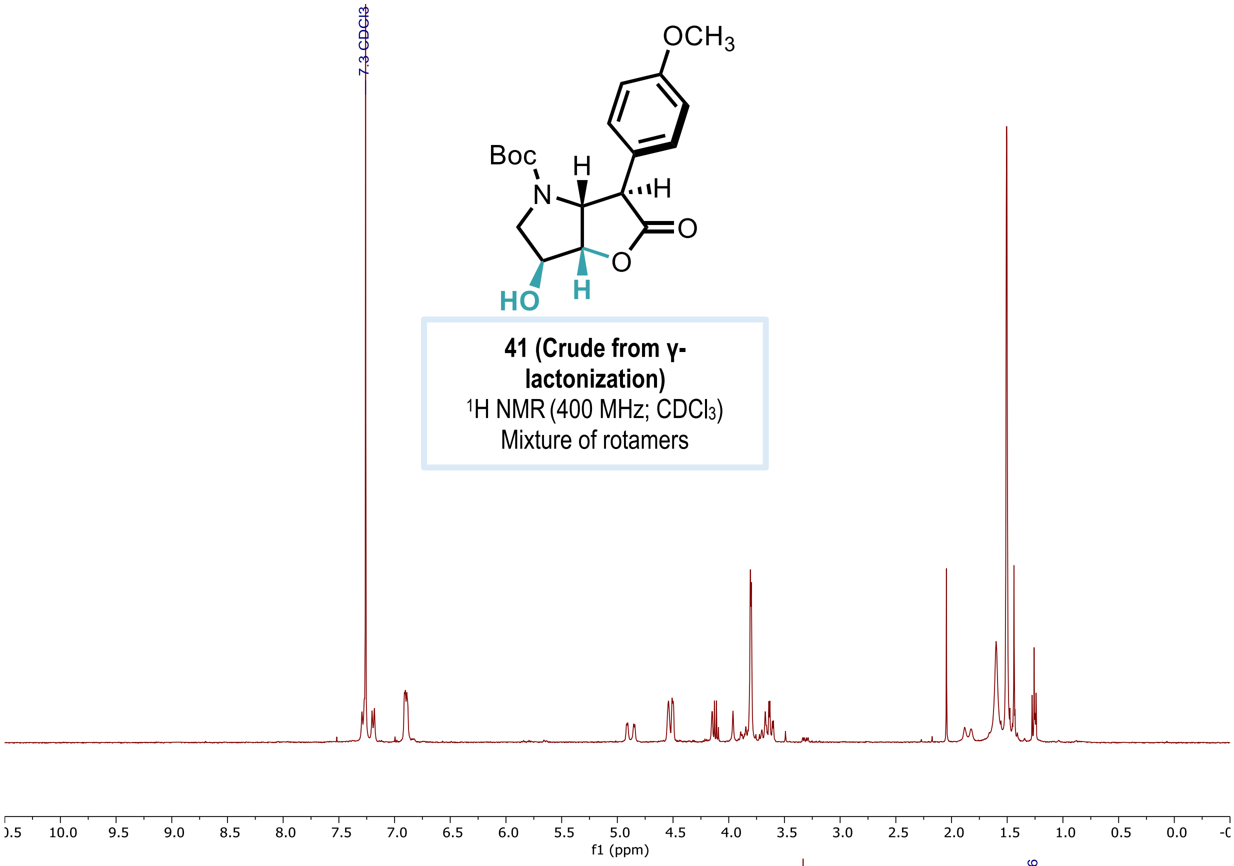

OCH3
Boc
N
H
H
O
O
HO
H
41 (Crude from γ-lactonization)
1H NMR (400 MHz; CDCl3)
Mixture of rotamers
7.3 CDCl3
f1 (ppm)

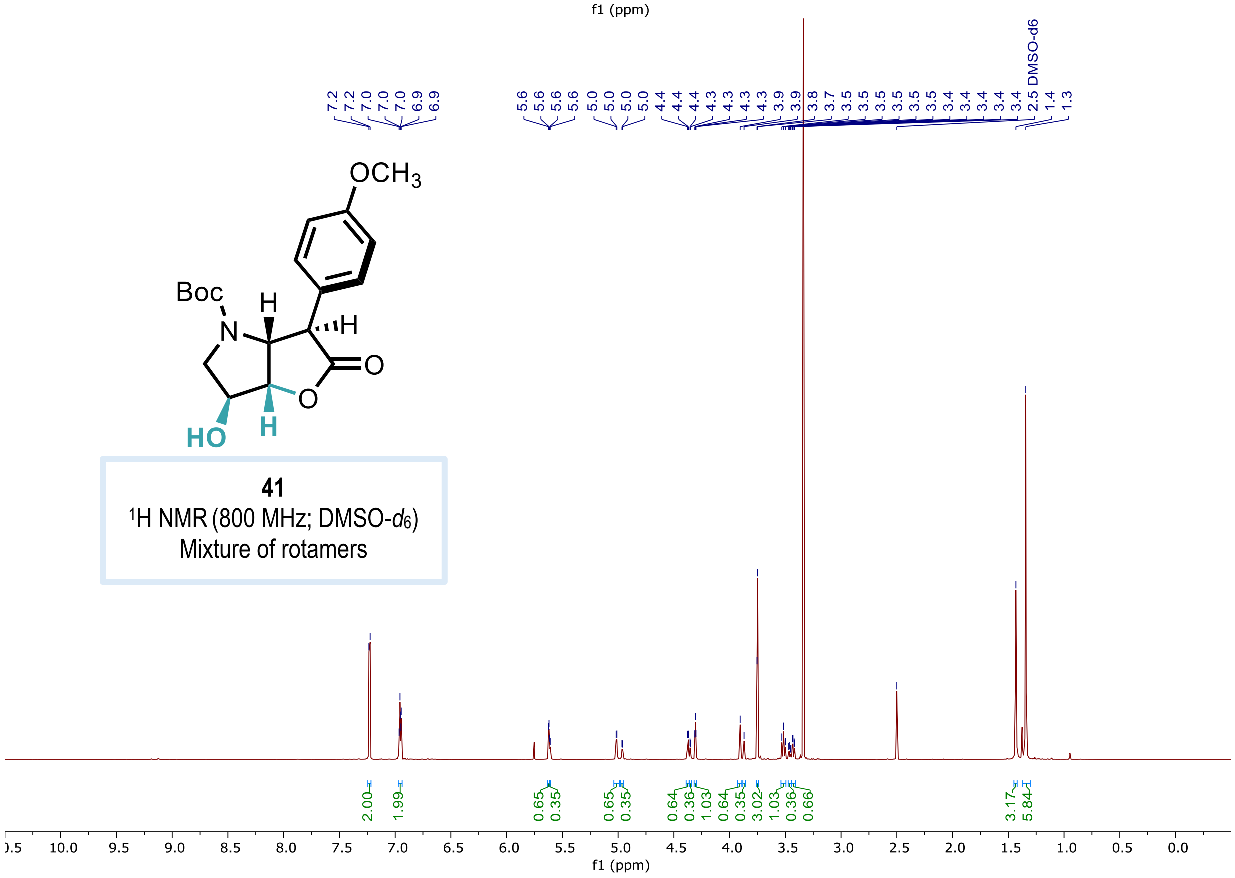

OCH3
Boc
N
H
H
O
O
HO
H
41
1H NMR (800 MHz; DMSO-d6)
Mixture of rotamers
2.5 DMSO-d6
2.00
1.99
0.65
0.35
0.65
0.35
0.64
0.36
1.03
0.64
0.35
3.02
1.03
0.36
0.66
3.17
5.84
f1 (ppm)

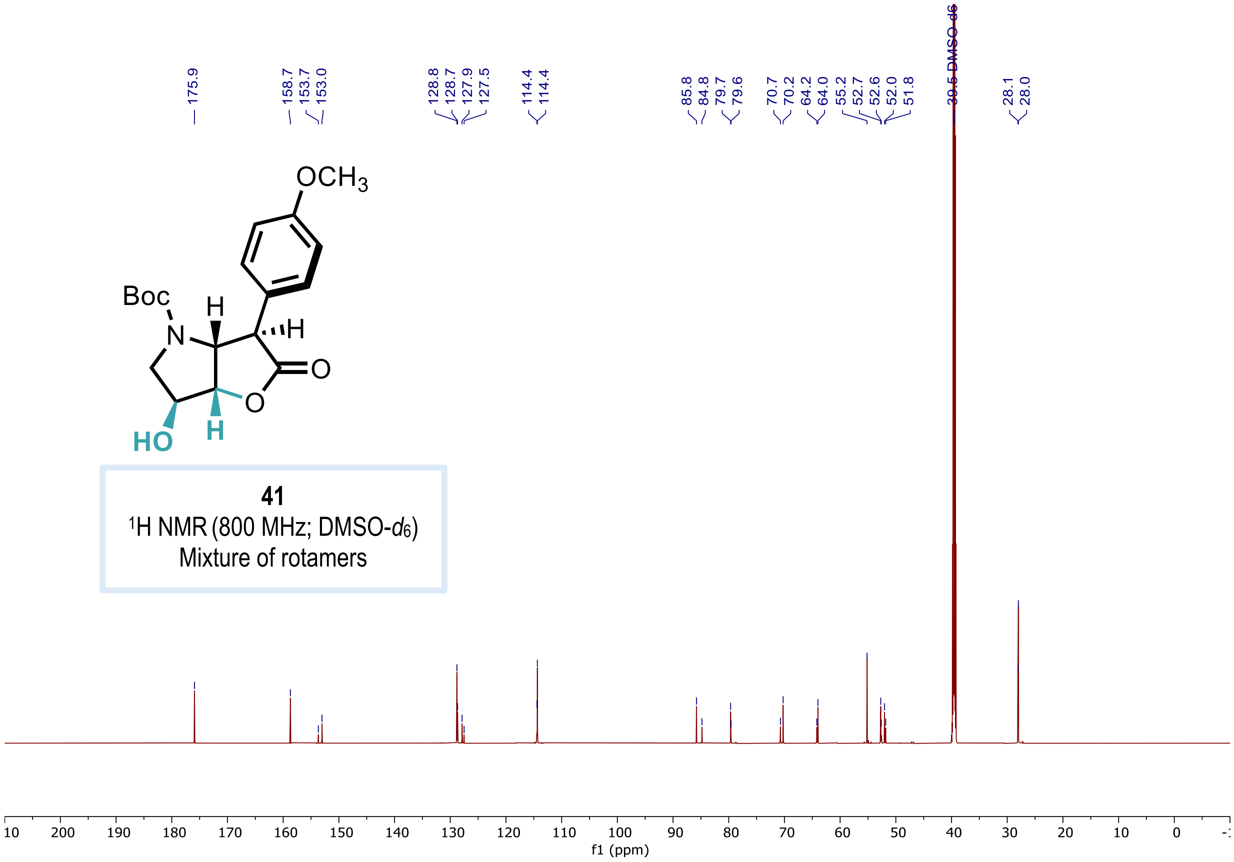

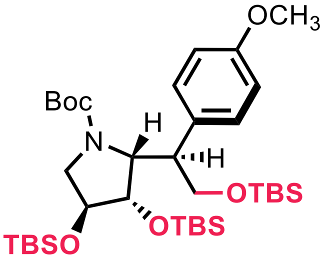

tert-butyl (2R,3S,4S)-3,4-bis((tert-butyldimethylsilyl)oxy)-2-((R)-2-((tert-butyldimethylsilyl)oxy)-1-(4-methoxyphenyl)ethyl)pyrrolidine-1-carboxylate (42)

[$LiBH_4$ Reduction] – *Adapted as a modified procedure by Soai, K. et al.* ***1986***:[25] To a flame-dried 1 L RBF charged with a stir-bar was added tert-butyl (3R,3aR,6S,6aS)-6-hydroxy-3-(4-methoxyphenyl)-2-oxohexahydro-4H-furo[3,2-b]pyrrole-4-carboxylate (20.3 g, 291 mL in anhydrous THF, 0.20 molar, 1 equiv, 58.1 mmol) and anhydrous methanol (7.45 g, 9.40 mL, 4 equiv, 232 mmol). The reaction vessel was sparged with nitrogen for at least 15 minutes and then capped with 2 nitrogen balloons. The reaction solution cooled to 0 °C via a saltwater ice bath.

$LiBH_4$ in 2 M THF (5.06 g, 116 mL, 2 molar, 4.0 equiv, 232 mmol) was added slowly drop-wise over 20 minutes to the cooled reaction solution. The 2 nitrogen balloons were replaced with vent needles once the bubbling started. During the slow addition, addition was paused when bubbling was vigorous and continued when the bubbling was less vigorous.

After the complete addition, the mixture was stirred in the cooling bath at 0 °C for 4 hours. After the elapsed time, the cooled reaction solution was quenched by slow addition of saturated ammonium chloride solution, extracted with EtOAc three times, the combined organics dried with $MgSO_4$, vacuum filtered through a plug of celite, and the filtrate evaporated with a positive stream of air overnight. The tert-

butyl (2R,3S,4S)-3,4-dihydroxy-2-((R)-2-hydroxy-1-(4-methoxyphenyl)ethyl)pyrrolidine-1-carboxylate **(S17)** (20.18 g, 57.10 mmol) as white solid material was pushed forwards to the next step without any purification.

[Global TBS Protection] - To a 1 L RBF charged with a stir-bar was added tert-butyl (2R,3S,4S)-3,4-dihydroxy-2-((R)-2-hydroxy-1-(4-methoxyphenyl)ethyl)pyrrolidine-1-carboxylate (20 g, 1 equiv, 57 mmol), DMF (4.1 g, 0.57 L, 0.1 molar, 1 equiv, 57 mmol), and tert-butylchlorodimethylsilane (51 g, 6 equiv, 0.34 mol). The reaction mixture was mixed and then to the reaction vessel was added 1H-imidazole (46 g, 12 equiv, 0.68 mol) and DMAP (1.4 g, 0.2 equiv, 11 mmol). The vessel is capped with a vent-needle. The reaction mixture was heated to 55 °C for 21 hours.

After the elapsed time, the reaction mixture was diluted with saturated sodium bicarbonate solution, diluted with diethyl ether, the organics extracted with diethyl ether three times, the combined organics dried with $MgSO_4$, vacuum filtered through a plug of celite, and the filtrate concentrated *in vacuo* at 55 °C.

The crude was dry-loaded onto SiO_2 and subjected to SiO_2 flash chromatography using hexanes/diethyl ether (200 g Biotage Sfär DLV column as the running-column and 200 g Biotage Sfär DLV column for the dry-load; 100g KP-Sil setting; 0% 5CV → 1% 5CV → 3% 5CV → 5% 5CV → 7% 5CV → 10% 5CV → 15% 5CV; 200 mL/min flow-rate; 210/230 nm detector; POI elutes at 7 to 10% ether).

The collected fractions were concentrated *in vacuo* at 55 °C to afford tert-butyl (2R,3S,4S)-3,4-bis((tert-butyldimethylsilyl)oxy)-2-((R)-2-((tert-butyldimethylsilyl)oxy)-1-(4-methoxyphenyl)ethyl)pyrrolidine-1-carboxylate (24 g, 34 mmol, 61% yield) as a clear pale-yellow oil.

^{1}H NMR (800 MHz, Chloroform-*d*; as a 0.50:0.50 mixture of rotamers) δ 7.10 (d, *J* = 8.3 Hz, 2H), 6.80 (d, *J* = 8.3 Hz, 2H), 4.25 (d, *J* = 8.0 Hz, 0.5H), 4.16 (d, *J* = 8.0 Hz, 0.5H), 4.03 – 3.91 (m, 2H, mixture of rotamers), 3.90 – 3.82 (m, 1H, mixture of rotamers), 3.77 (overlapping s's, 3H, mixture of rotamers), 3.33 (t, *J* = 7.9 Hz, 0.5H), 3.26 (t, *J* = 7.9 Hz, 0.5H), 3.21 (q, *J* = 7.9 Hz, 0.5H), 3.10 (q, *J* = 7.9 Hz, 0.5H), 2.91 (dd, *J* = 10.9, 7.5 Hz, 0.5H), 2.87 (dd, *J* = 10.9, 7.5 Hz, 0.5H), 2.77 (dd, *J* = 10.9, 7.5 Hz, 0.5H), 2.73 (dd, *J* = 10.9, 7.5 Hz, 0.5H), 1.54 (s, 4.5H), 1.48 (s, 4.5H), 1.00 (s, 9H), 0.77 (s, 9H), 0.73 (s, 4.5H), 0.71 (s, 4.5H), 0.14 (s, 3H), 0.11 (s, 3H), 0.02 – -0.04 (m, 3H), -0.08 – -0.13 (m, 3H), -0.13 – -0.17 (m, 3H), -0.20 – -0.27 (m, 3H).

^{13}C NMR (201 MHz, Chloroform-*d*; as a mixture of rotamers) δ 158.7, 158.5, 155.2, 132.9, 132.4, 131.1, 131.0, 113.6, 113.4, 80.2, 79.3, 78.0, 77.6, 74.3, 73.6, 66.8, 66.5, 60.5, 59.5, 55.4, 51.0, 50.8, 47.1, 47.0, 28.7, 28.6, 26.3, 26.2, 26.1, 26.0, 25.9, 18.3, 18.3, 18.2, 18.0, 18.0, -4.3, -4.4, -4.4, -4.5, -4.5, -4.6, -4.9, -5.1, -5.1, -5.3.

HRMS (ESI +) calc. mass for $C_{36}H_{70}O_6N^{28}Si_3$ $[M + H]^+$ 696.4505; obs. mass for $C_{36}H_{70}O_6N^{28}Si_3$ $[M + H]^+$ 696.4505.

$[\alpha]^{20}_D$: -14.2° (c = 0.590 g/100 mL, EtOAc).

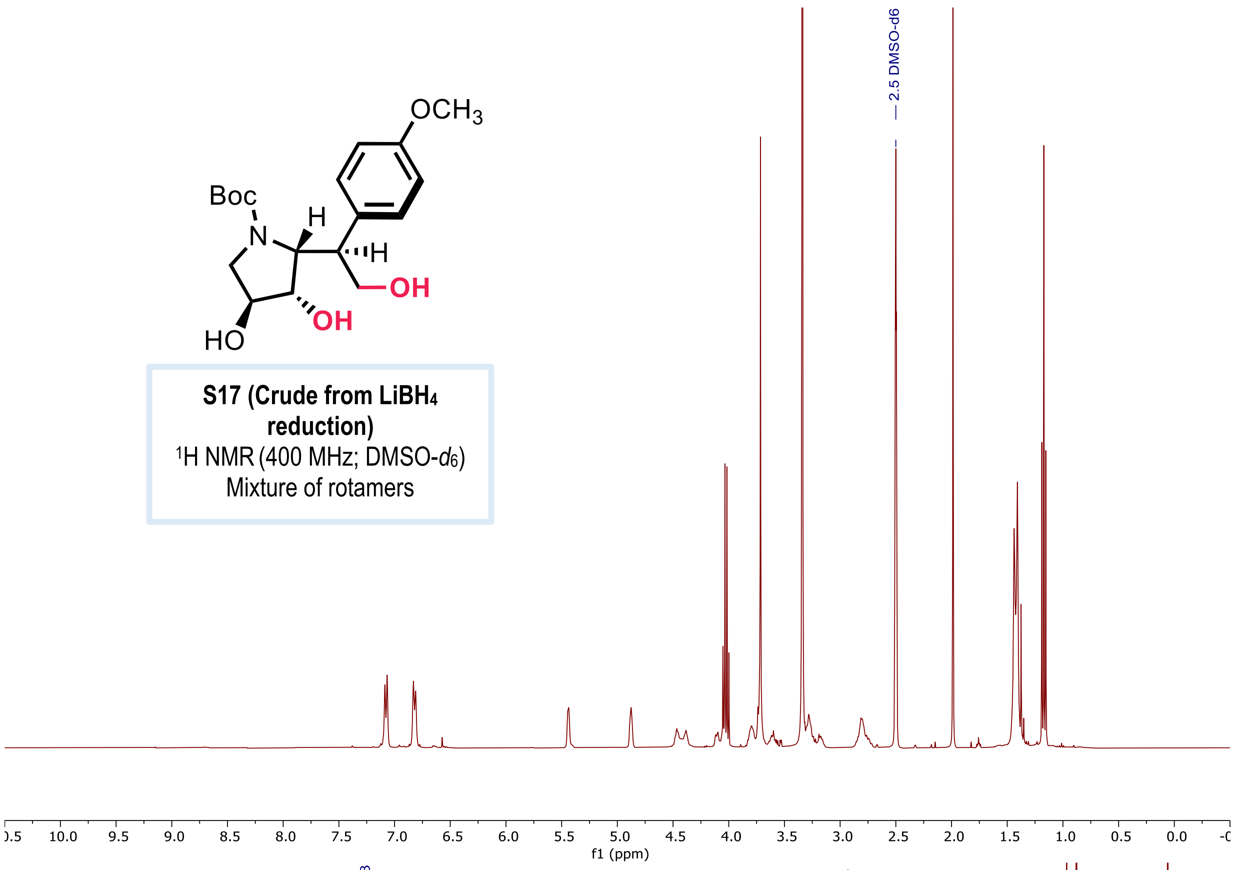
OCH3
Boc
N
H
H
OH
OH
HO
S17 (Crude from LiBH4 reduction)
1H NMR (400 MHz; DMSO-d6)
Mixture of rotamers
2.5 DMSO-d6
f1 (ppm)

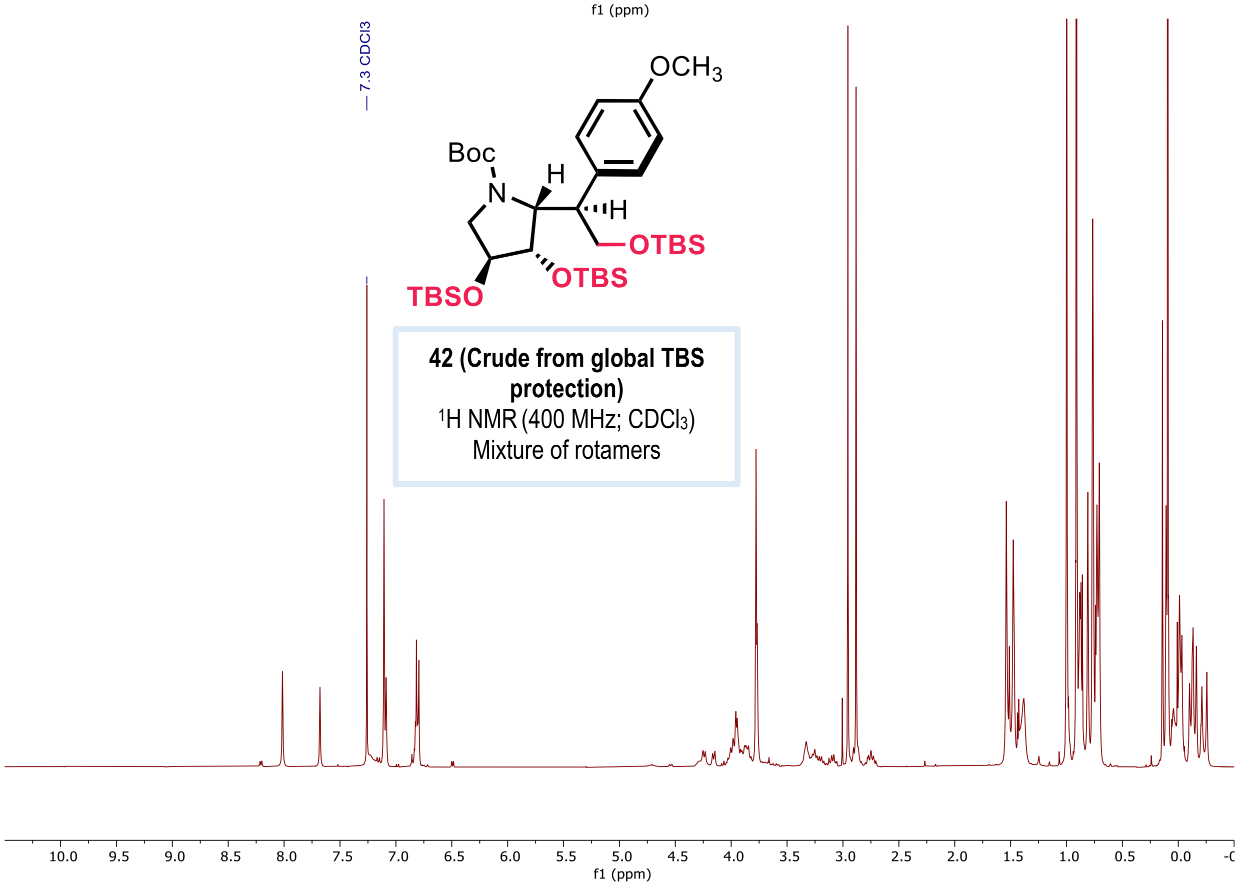
7.3 CDCl3
OCH3
Boc
N
H
H
OTBS
OTBS
TBSO
42 (Crude from global TBS protection)
1H NMR (400 MHz; CDCl3)
Mixture of rotamers
f1 (ppm)

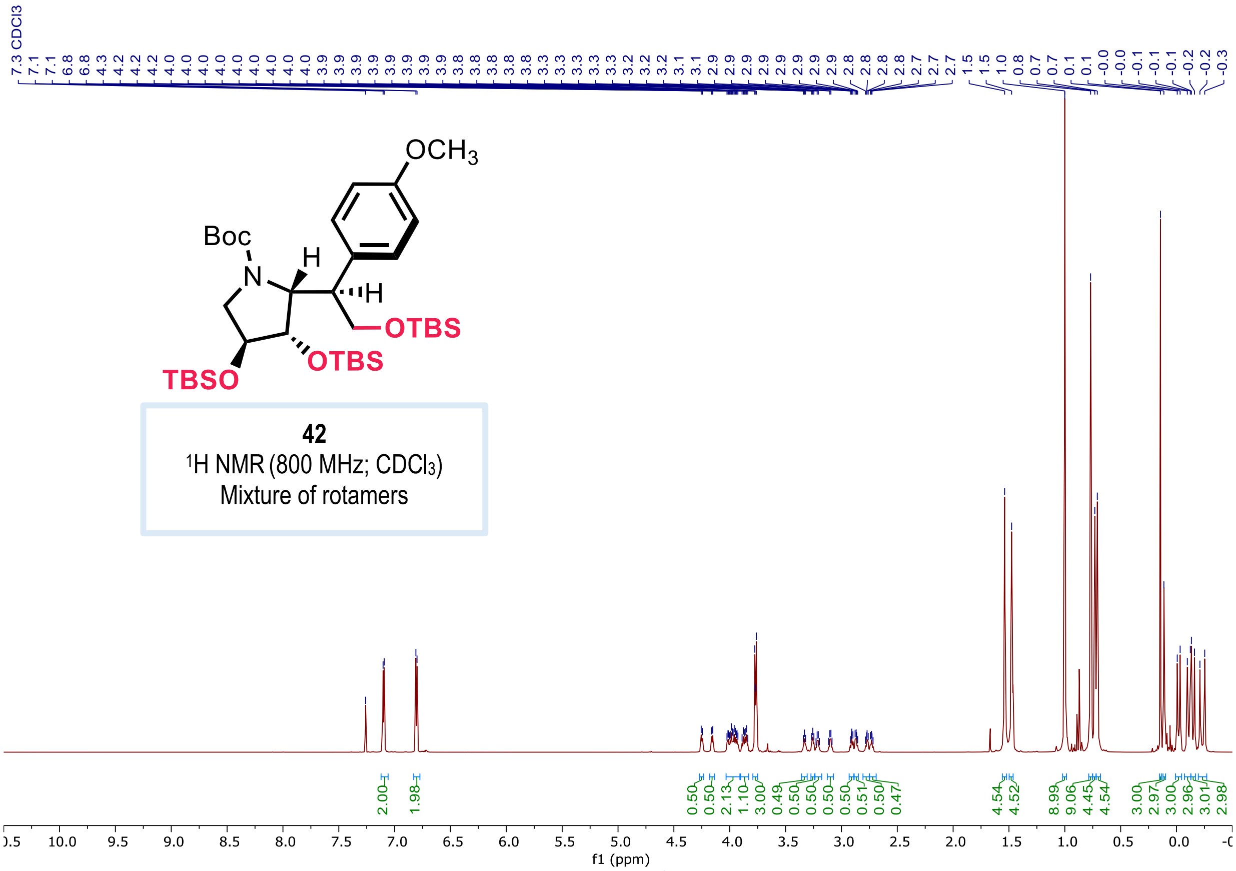

42
^{1}H NMR (800 MHz; $CDCl_3$)
Mixture of rotamers

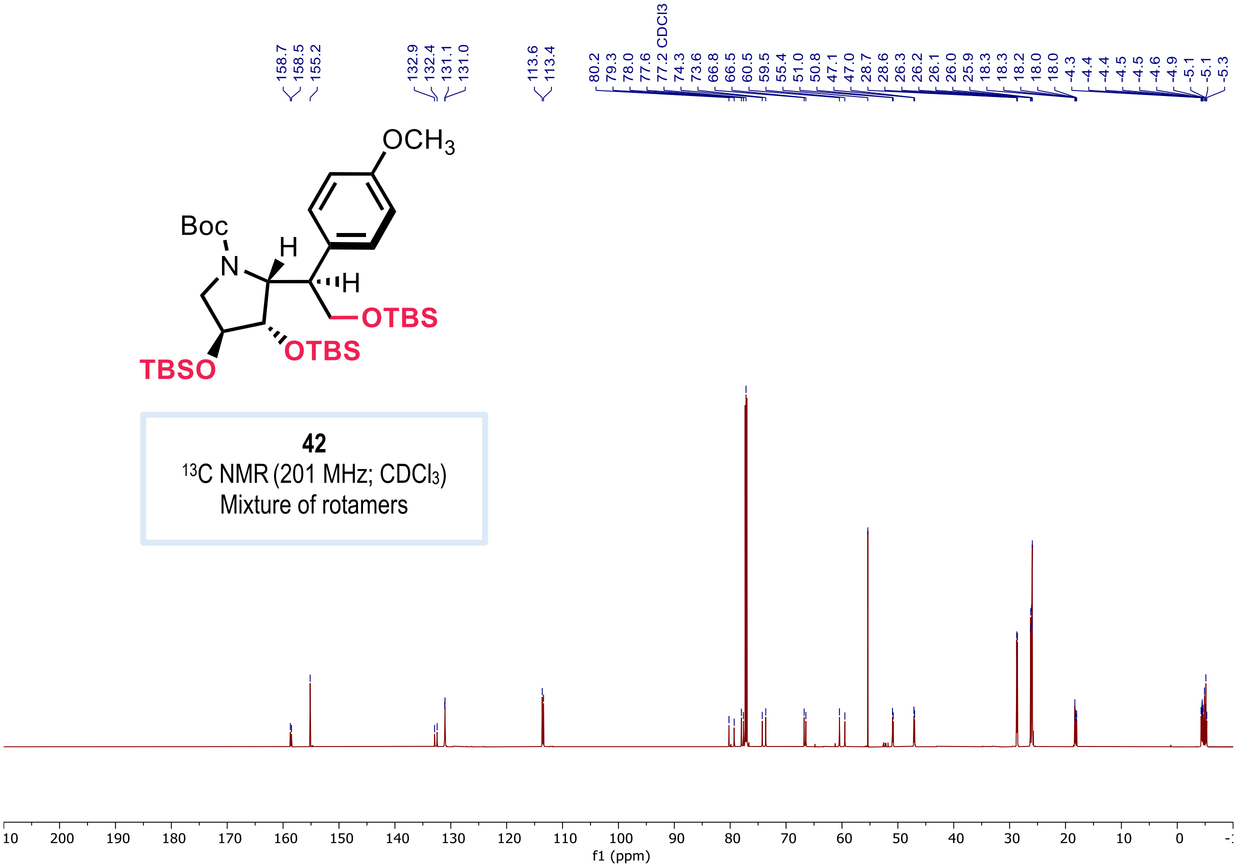

42
^{13}C NMR (201 MHz; $CDCl_3$)
Mixture of rotamers

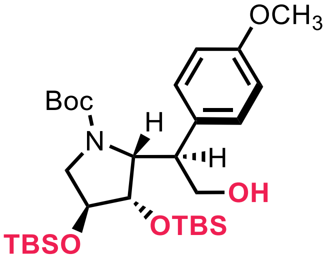

tert-butyl (2R,3S,4S)-3,4-bis((tert-butyldimethylsilyl)oxy)-2-((R)-2-hydroxy-1-(4-methoxyphenyl)ethyl)pyrrolidine-1-carboxylate (43)

Adapted as a modified procedure by Huang, F. et al. ***2019***:[26] To a 1 L RBF charged with a stir-bar containing tert-butyl (2R,3S,4S)-3,4-bis((tert-butyldimethylsilyl)oxy)-2-((R)-2-((tert-butyldimethylsilyl)oxy)-1-(4-methoxyphenyl)ethyl)pyrrolidine-1-carboxylate (23 g, 1 equiv, 33 mmol) was dissolved in acetonitrile (0.25 L). Once the SM was fully solubilized by the acetonitrile with sonication, DI Water (33 mL) was added to the stirring solution resulting in a white colloidal suspension. Afterwards, formic acid (50 mL, 88% FA) was added to the reaction suspension. The total reaction concentration is 0.100 M and the cocktail of ACN/DI water/FA is 15:2:3 ratio. The reaction was stirred at 25 °C for 3 hours. At the end of the reaction, the reaction solution is clear. After the elapsed time, the reaction was quenched by adding the reaction solution dropwise into an RBF containing saturated bicarbonate solution while stirring, the quenched aqueous solution (pH of ~7) diluted with diethyl ether, extracted with diethyl ether three times, the combined organics were dried with $MgSO_4$, vacuum filtered through a plug of celite, and the filtrate concentrated *in vacuo* at 55 ^{o}C.

The crude was dry-loaded onto SiO_2 and subjected to SiO_2 flash chromatography using hexanes/diethyl ether (200 g Biotage Sfär DLV column as the running-column and 200 g Biotage Sfär DLV column for the dry-load; 100 g KP-Sil setting; 0% 5CV → 1% 5CV → 3% 5CV → 5% 5CV → 7% 5CV → 10% 5CV → 15% 5CV → 30% 5CV → 50% 5CV → 75% 5CV → 100% 5CV; 200 mL/min flow-rate; 210/230 nm detector; POI elutes at 30 to 50% ether as two peaks coalescing one another [higher 230 nm detection than 210 nm]; TLC 1:1 hexane/ether ~0.8 Rf desired spot).

The collected fractions were concentrated *in vacuo* at 55 ^{o}C to afford tert-butyl (2R,3S,4S)-3,4-bis((tert-butyldimethylsilyl)oxy)-2-((R)-2-hydroxy-1-(4-methoxyphenyl)ethyl)pyrrolidine-1-carboxylate (15 g, 26 mmol, 78% yield) as a colorless clear oil.

^{1}H NMR (400 MHz, Chloroform-*d*; as a mixture of rotamers) δ 7.14 (d, *J* = 8.7 Hz, 2H), 6.83 (d, *J* = 8.7 Hz, 2H), 4.54 (dd, *J* = 9.5, 5.7 Hz, 1H), 4.42 (dd, *J* = 8.2, 2.0 Hz, 1H), 4.03 (t, *J* = 8.2 Hz, 1H), 3.81 – 3.74 (s & dd overlap, 4H), 3.62 (ddd, *J* = 11.9, 9.5, 5.7 Hz, 1H), 3.21 (ddd, *J* = 11.2, 5.7, 1.9 Hz, 1H), 3.05 (dd, *J* = 10.2, 7.8 Hz, 1H), 2.89 (q, *J* = 7.8 Hz, 1H), 2.81 (dd, *J* = 10.2, 7.8 Hz, 1H), 1.57 – 1.48 (overlapping s's, 9H), 1.00 (s, 9H), 0.72 (s, 9H), 0.17 (s, 3H), 0.13 – 0.07 (overlapping s's, 3H), -0.17 (s, 3H), -0.26 (s, 3H).
^{13}C NMR (201 MHz, Chloroform-*d*; as a mixture of rotamers) δ 159.0, 156.7, 131.9, 131.1, 113.7, 80.7, 77.5, 73.7, 63.6, 58.3, 55.4, 51.0, 46.0, 28.6, 26.3, 26.0, 25.8, 18.3, 18.0, -3.4, -4.4, -4.5, -4.6, -5.0.
HRMS (ESI +) calc. mass for $C_{30}H_{56}O_6N^{28}Si_2$ [M + H]$^+$ 582.3641; obs. mass for $C_{30}H_{56}O_6N^{28}Si_2$ [M + H]$^+$ 582.3642.
[α]$^{20}_{D}$: -6.0^{o} (c = 0.300 g/100 mL, EtOAc).

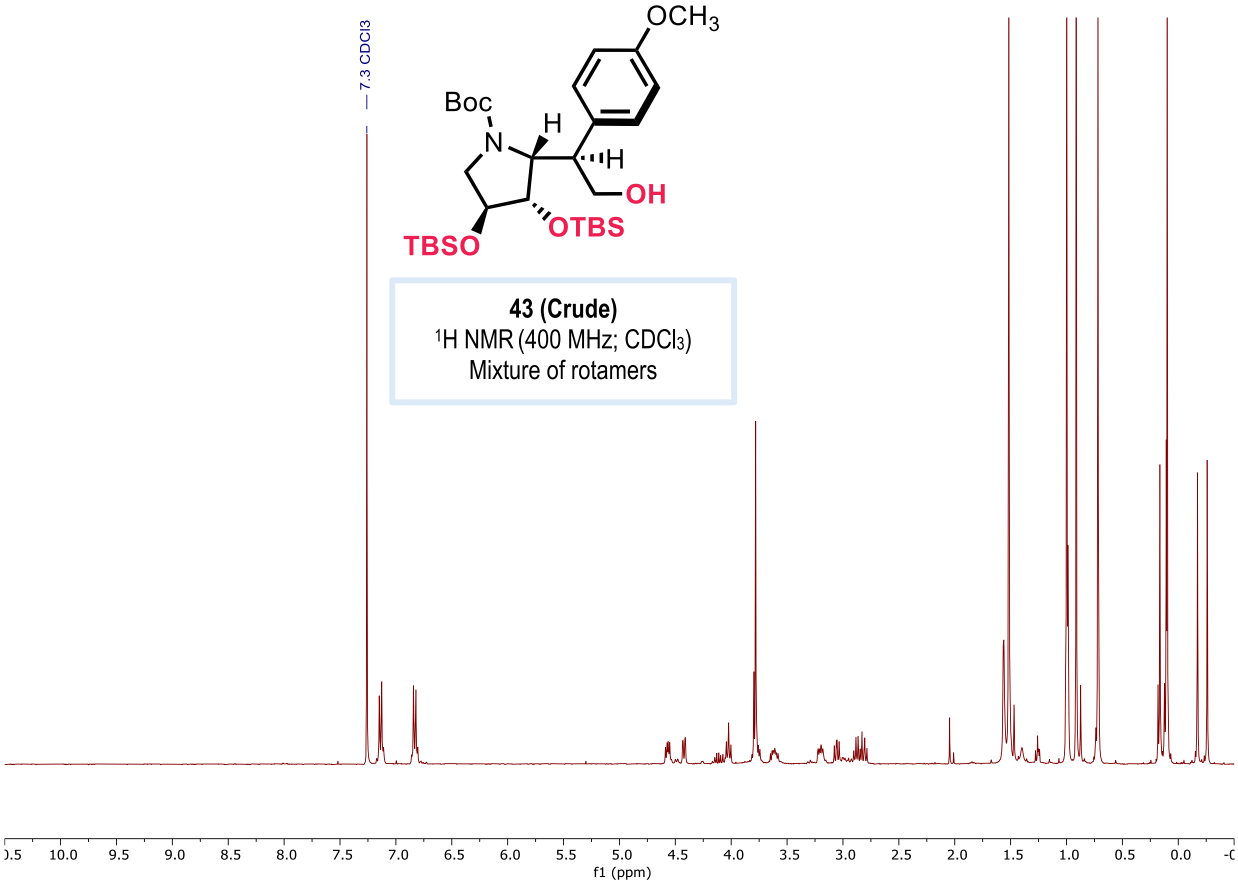
OCH3
Boc
N
H
H
OH
OTBS
TBSO
43 (Crude)
1H NMR (400 MHz; CDCl3)
Mixture of rotamers
7.3 CDCl3
f1 (ppm)

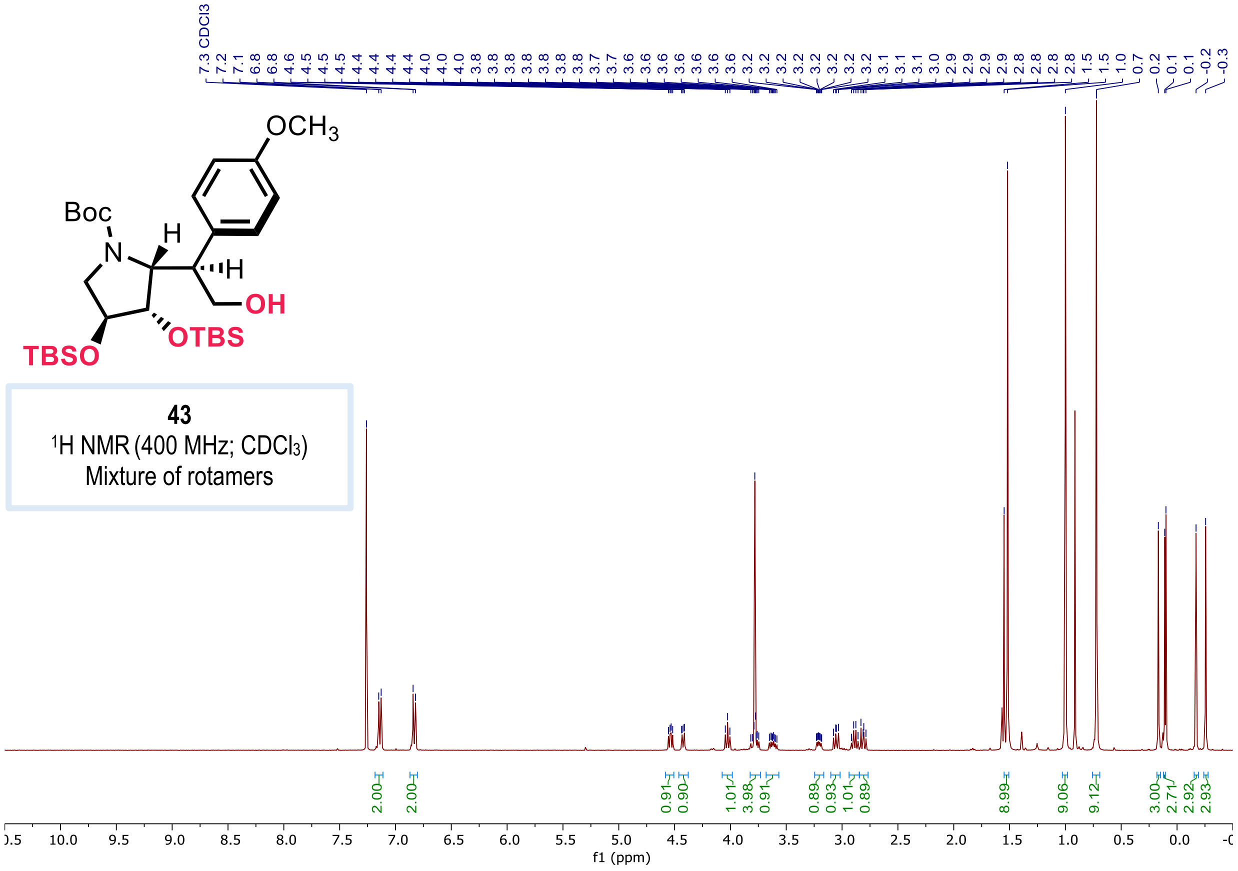
OCH3
Boc
N
H
H
OH
OTBS
TBSO
43
1H NMR (400 MHz; CDCl3)
Mixture of rotamers
7.3 CDCl3
f1 (ppm)

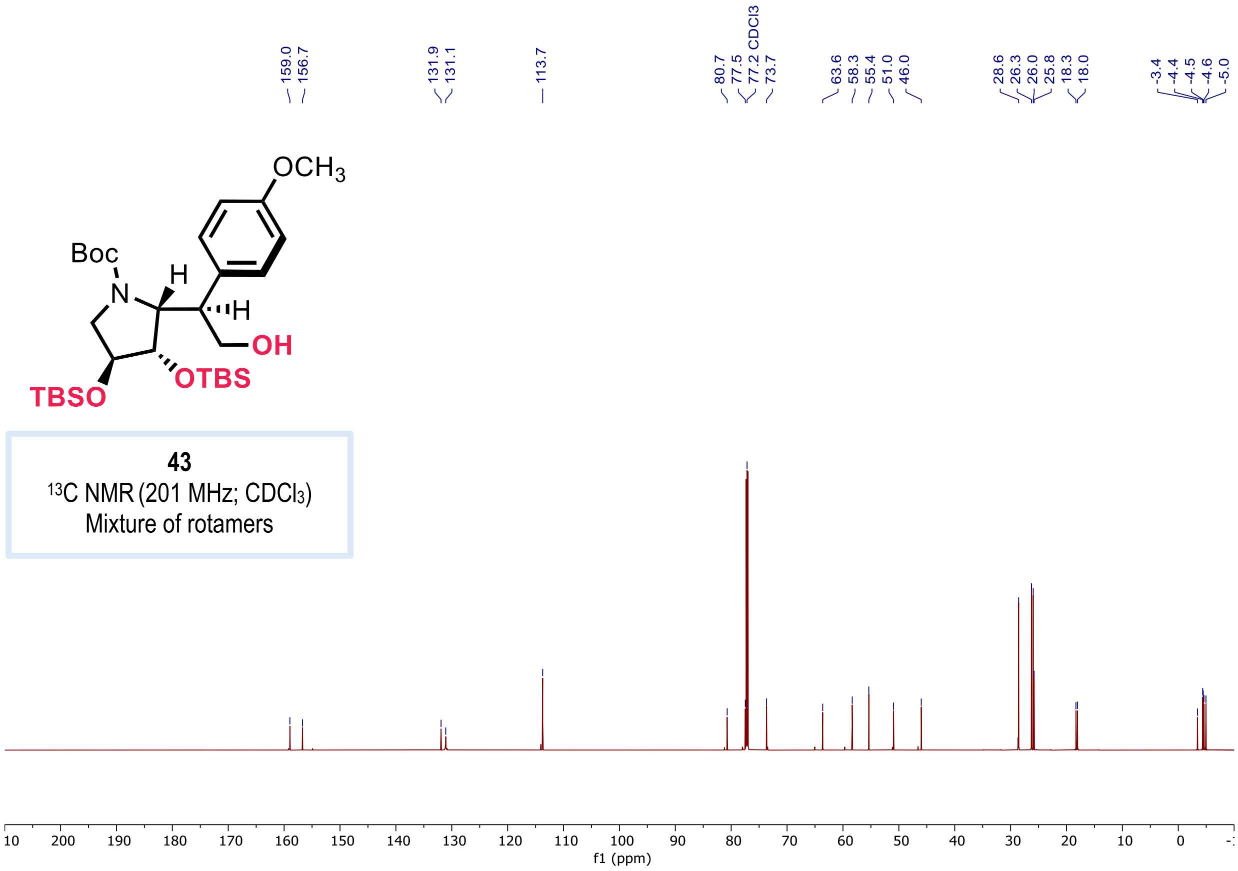

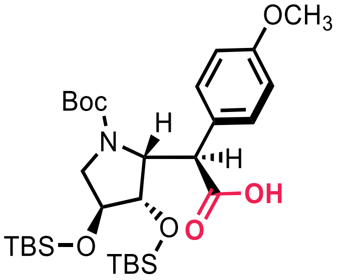

(R)-2-((2R,3S,4S)-1-(tert-butoxycarbonyl)-3,4-bis((tert-butyldimethylsilyl)oxy)pyrrolidin-2-yl)-2-(4-methoxyphenyl)acetic acid (44)

[TEMPO Oxidation] - *Adapted as a modified procedure by Chandrasekhar, S. et al.* ***2012***:[27] To a vial charged with a stir-bar was added tert-butyl (2R,3S,4S)-3,4-bis((tert-butyldimethylsilyl)oxy)-2-((R)-2-hydroxy-1-(4-methoxyphenyl)ethyl)pyrrolidine-1-carboxylate (14.8 g, 1 equiv, 25.4 mmol), (diacetoxyiodo)benzene (24.6 g, 3 equiv, 76.3 mmol), acetonitrile (127 mL) and DI water (127 mL). The final reaction concentration is 0.100 M. The mixture was sonicated and stirred vigorously at room temperature. To the stirred solution was added TEMPO (1.19 g, 0.3 equiv, 7.63 mmol) and the reaction mixture capped and stirred vigorously at room temperature for 6 hours.

After the elapsed time, the reaction mixture was quenched with saturated sodium thiosulfate solution, diluted with EtOAc, extracted with EtOAc three times, the organics dried with $MgSO_4$, vacuum filtered through a plug of celite, and the filtrate concentrated *in vacuo* at 55 °C to afford crude tert-butyl (2R,3S,4S)-3,4-bis((tert-butyldimethylsilyl)oxy)-2-((R)-1-(4-methoxyphenyl)-2-oxoethyl)pyrrolidine-1-carboxylate **(S18)** as a yellow oil. The crude material was subjected to the next step without purification.

[Pinnick Oxidation] - *Adapted as a modified procedure by Kalesse, M. et al.* ***2023***:[28] An RBF charged with a stirbar containing tert-butyl (2R,3S,4S)-3,4-bis((tert-butyldimethylsilyl)oxy)-2-((R)-1-(4-methoxyphenyl)-2-oxoethyl)pyrrolidine-1-carboxylate (14.7 g, 1 equiv, 25.4 mmol) was added 2-methyl-2-butene (35.6 g, 53.8 mL, 20 equiv, 508 mmol), acetone (1.48 g, 254 mL, 0.1 molar, 1 equiv, 25.4 mmol),

and sodium dihydrogen phosphate hydrate solution (38.6 g, 140 mL, 2.0 molar in DI water, 11 equiv, 279 mmol). The reaction mixture was stirred at room temperature for at least a minute.

To the stirring solution was added sodium chlorite (34.5 g, 80% Wt, 12 equiv, 305 mmol), the reaction solution capped with a nitrogen balloon and allowed to stir at room temperature for 18 hours.

After the elapsed time, the reaction mixture was diluted with saturated citric acid solution and EtOAc, the organics extracted with EtOAc three times, the combined organics dried with $MgSO_4$, vacuum filtered through a plug of celite, and the filtrate concentrated *in vacuo* at 55 °C.

The crude was dry-loaded onto SiO_2 and subjected to SiO_2 flash chromatography hexanes/EtOAc (Biotage Sfär DLV 100 g column as the running-column and Biotage Sfär DLV 200 g column for the dry-load; 0% 5CV → 0 % to 100% 10CV → 100% 5CV; 200 mL/min flow-rate; 254/280 nm detector; POI has a little higher 280 nm absorbance than 254 nm; POI elutes at 20% to 40% EtOAc; TLC 1:1 hexanes/EtOAc POI Rf is 0.8 and 0.3, spot at 0.8 Rf stains yellow with bromocresol green and heat and spot at 0.3 Rf does not stain)

The collected fractions were concentrated *in vacuo* at 55 °C to afford (R)-2-((2R,3S,4S)-1-(tert-butoxycarbonyl)-3,4-bis((tert-butyldimethylsilyl)oxy)pyrrolidin-2-yl)-2-(4-methoxyphenyl)acetic acid (13.8 g, 23.2 mmol, 91.2% yield) as a white foam after leaving it under high-vac overnight.

^{1}H NMR (600 MHz, Chloroform-*d*; as a 0.6:0.4 mixture of rotamers) δ 7.17 (Ar–H d, *J* = 8.2 Hz, 2H, mixture of rotamers), 6.89 – 6.72 (Ar–H m, 2H, mixture of rotamers), 4.67 (d, *J* = 6.6 Hz, 0.4H, minor rotamer), 4.57 (d, *J* = 6.6 Hz, 0.6H, major rotamer), 4.37 (brs, 0.6H, major rotamer), 4.17 (brs, 0.4H, minor rotamer), 4.11 (t, *J* = 7.7 Hz, 1H, mixture of rotamers), 3.78 (s, 3H, mixture of rotamers), 3.59 – 3.49 (brm, 0.6H, major rotamer), 3.38 – 3.27 (brm, 0.4H, minor rotamer), 2.98 – 2.89 (brm, Hz, 0.8H, minor rotamers), 2.89 – 2.82 (m, 0.6H, major rotamer), 2.80 – 2.71 (m, 0.6H, major rotamer), 0.99 (s, 9H, mixture of rotamers), 0.81 – 0.71 (overlapping s's, 9H, mixture of rotamers), 0.20 – 0.09 (overlapping s's, 6H, mixture of rotamers), -0.01 – -0.20 (overlapping s's, 6H, mixture of rotamers).
^{13}C NMR (151 MHz, Chloroform-*d*; as a mixture of rotamers) δ 177.1, 174.6, 159.2, 156.4, 154.6, 132.3, 131.5, 127.6, 126.8, 113.8, 81.5, 81.1, 77.8, 74.1, 73.6, 61.8, 60.3, 55.4, 50.5, 49.8, 48.2, 28.4, 28.3, 26.1, 25.9, 18.2, 18.0, -4.3, -4.4, -4.5, -4.7.
HRMS (ESI +) calc. mass for $C_{30}H_{54}O_7N^{28}Si_2$ $[M + H]^+$ 596.3433; obs. mass for $C_{30}H_{54}O_7N^{28}Si_2$ $[M + H]^+$ 596.3428.
$[\alpha]^{20}_D$: +6.9° (c = 0.390 g/100 mL, EtOAc).

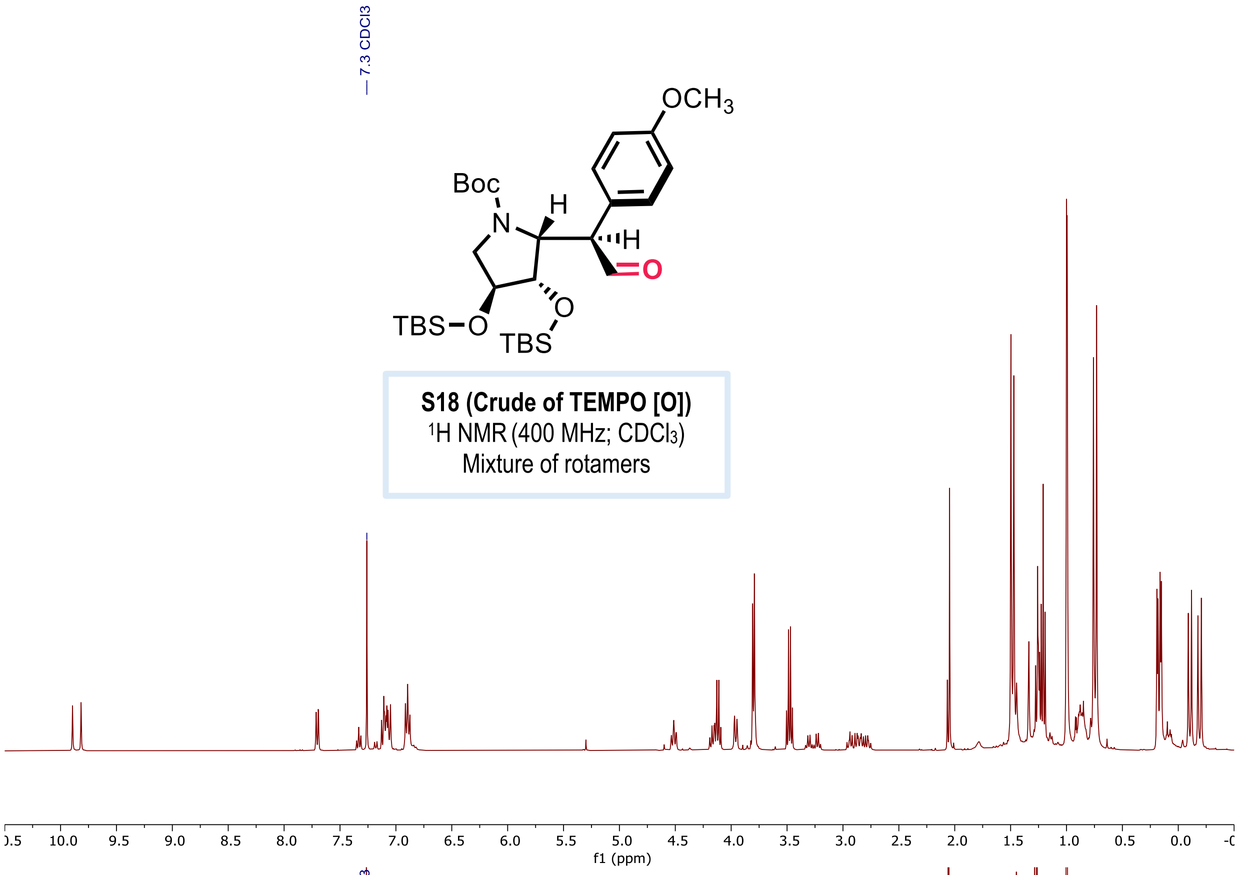
7.3 CDCl3
OCH3
Boc
N
H
H
O
TBS
TBS–O
S18 (Crude of TEMPO [O])
1H NMR (400 MHz; CDCl3)
Mixture of rotamers
f1 (ppm)

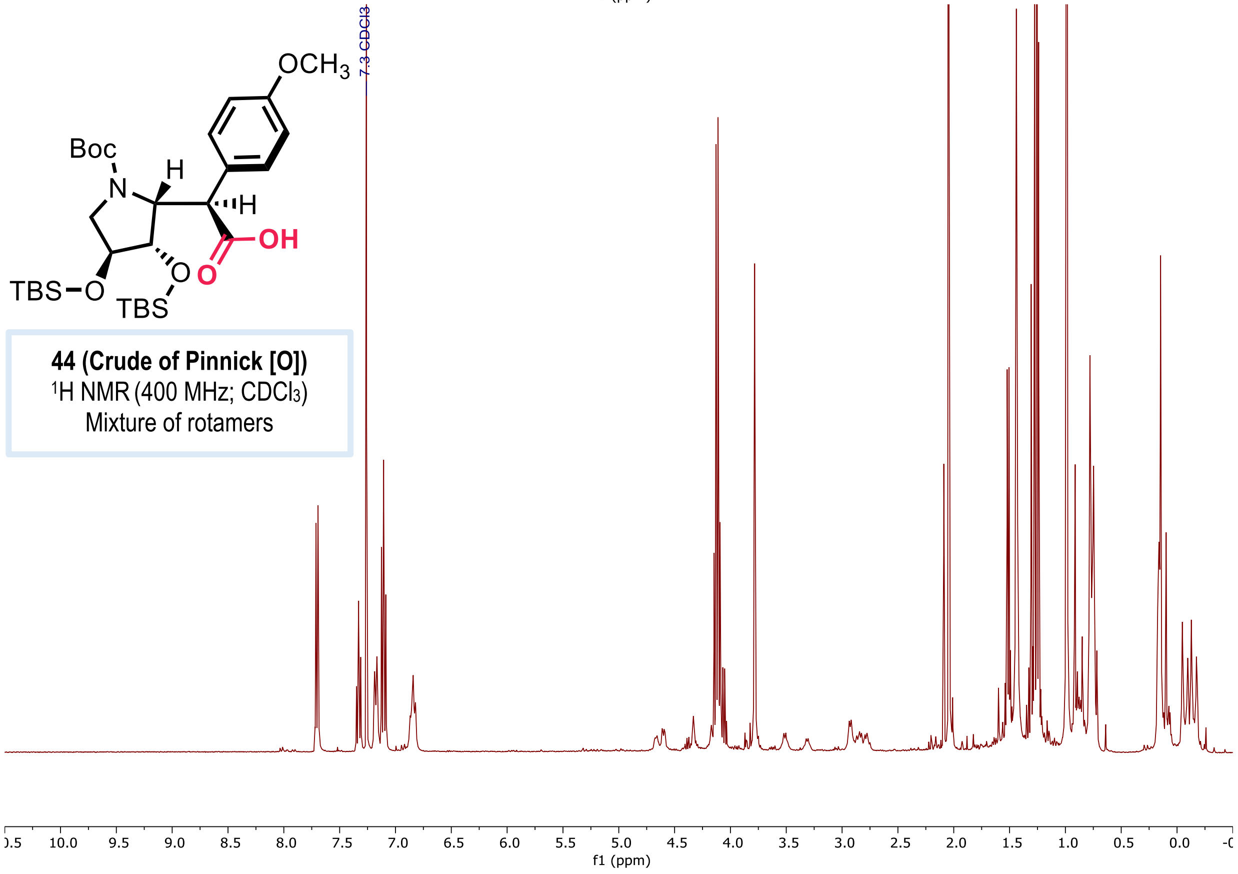
7.3 CDCl3
OCH3
Boc
N
H
H
OH
O
O
TBS
TBS–O
44 (Crude of Pinnick [O])
1H NMR (400 MHz; CDCl3)
Mixture of rotamers
f1 (ppm)

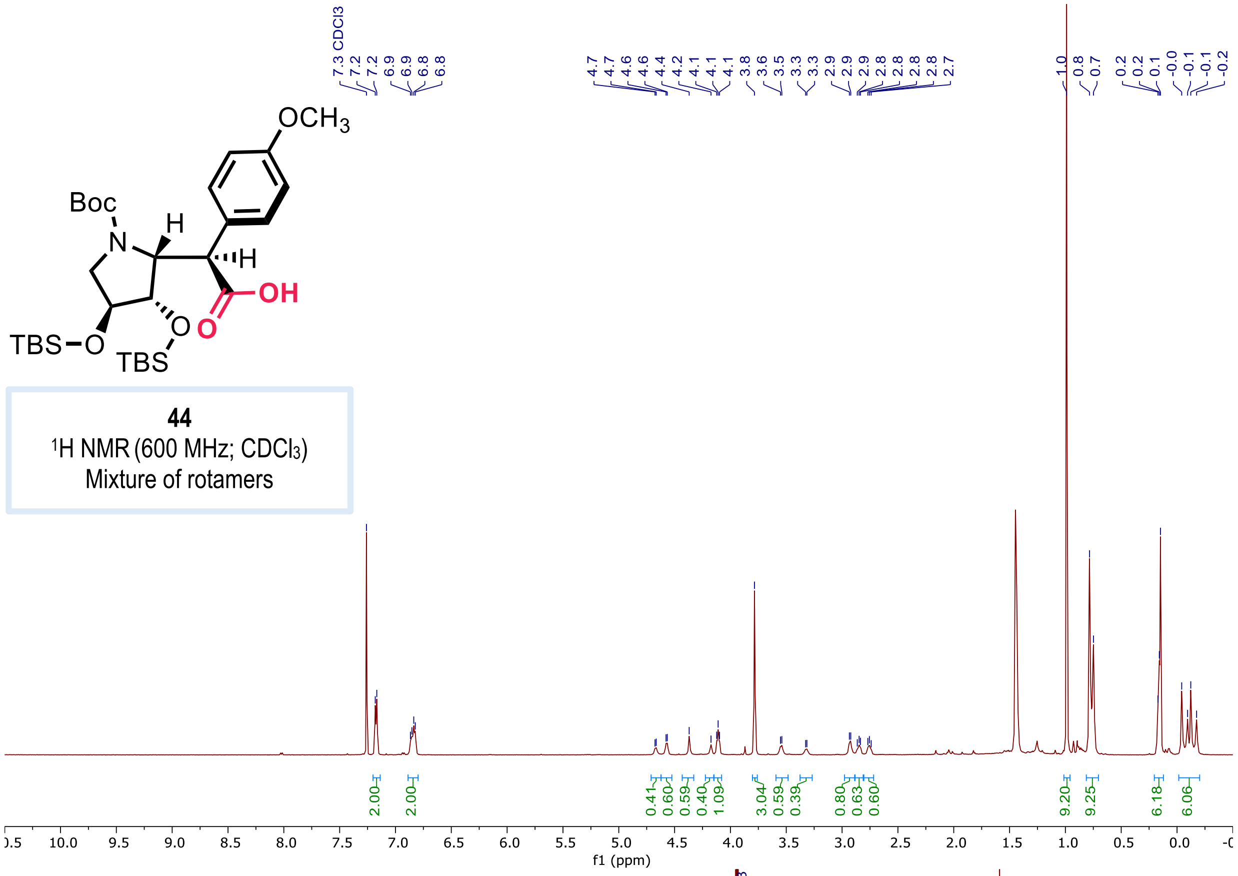
44
^{1}H NMR (600 MHz; $CDCl_3$)
Mixture of rotamers
f1 (ppm)

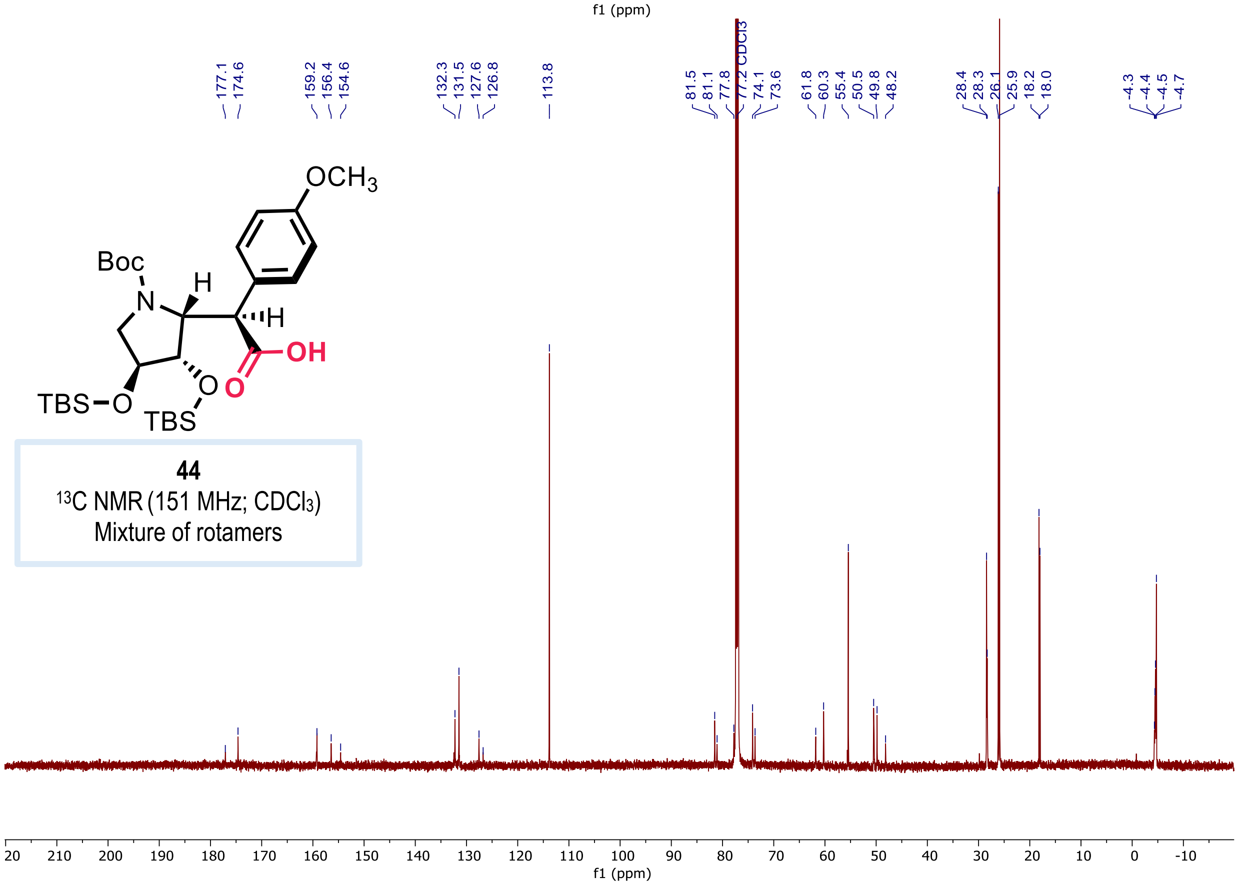
44
^{13}C NMR (151 MHz; $CDCl_3$)
Mixture of rotamers
f1 (ppm)

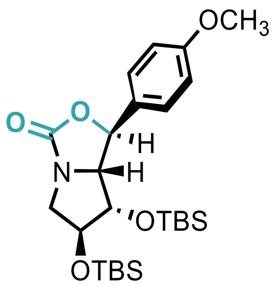

(1S,6S,7S,7aR)-6,7-bis((tert-butyldimethylsilyl)oxy)-1-(4-methoxyphenyl)tetrahydro-1H,3H-pyrrolo[1,2-c]oxazol-3-one (45)

Adapted as a modified procedure by Tunge, J. A. et al. ***2019***:[29] To a flame-dried 3-neck RBF charged with a stir bar equipped with a findenser and actiavted 4Å MS (0.100 g of MS / 1 mmol of carboxylic acid) was added (R)-2-((2R,3S,4S)-1-(tert-butoxycarbonyl)-3,4-bis((tert-butyldimethylsilyl)oxy)pyrrolidin-2-yl)-2-(4-methoxyphenyl)acetic acid (11.7 g, 1 equiv, 19.6 mmol), copper diacetate (10.7 g, 3 equiv, 58.9 mmol), and 9-mesityl-10-methylacridinium tetrafluoroborate (196 mg, 0.025 equiv, 491 µmol).

To the reaction vessel was added acetonitrile (806 mg, 98.2 mL, 0.20 molar, 1 equiv, 19.6 mmol) and the reaction solution sparged with nitrogen for at least 10 minutes (vent needle in septum of findenser and stream of nitrogen in septum of unused RBF neck). Afterwards, the vessel was capped with an argon balloon and placed in a light bath two flanking 440 nm Kessil lamp. The reaction vessel was positioned directly in front of the lamp with minimal distance in between. The reaction vessel was stirred for 24 hours under blue light irradiation. The temperature of the reaction was assessed to be approximately 78 ^{o}C via a digital thermometer.

After the elapsed time, the reaction vessel was diluted with EtOAc and saturated sodium bicarbonate solution, the organics extracted with EtOAc five times, the combined organics dried with $MgSO_4$, vacuum filtered through a plug of celite, and the filtrate concentrated *in vacuo* at 55 ^{o}C.

The crude was dry-loaded onto SiO_2 and subjected to SiO_2 flash chromatography using hexanes/diethyl ether (Biotage Sfär DLV 100 g column as the running-column and Biotage Sfär DLV 200 g column for the dry-load; 0% 5CV → 1% 5CV → 3% 5CV → 5% 5CV → 7% 5CV → 10% 5CV → 15% 5CV → 20% 5CV → 30% 5CV → 50% 5CV → 100% 5CV; 200 mL/min flow-rate; 210/230 nm detector; POI elutes at 20% ether; 230 nm detection is a little higher than 210 nm; TLC conditions 1:1 hexanes/ether product Rf is ~0.6).

The collected fractions were concentrated *in vacuo* at 55 ^{o}C to afford (1S,6S,7S,7aR)-6,7-bis((tert-butyldimethylsilyl)oxy)-1-(4-methoxyphenyl)tetrahydro-1H,3H-pyrrolo[1,2-c]oxazol-3-one (6.6 g, 13 mmol, 68% yield, 20:1 d.r.) as an off-white amorphous material.

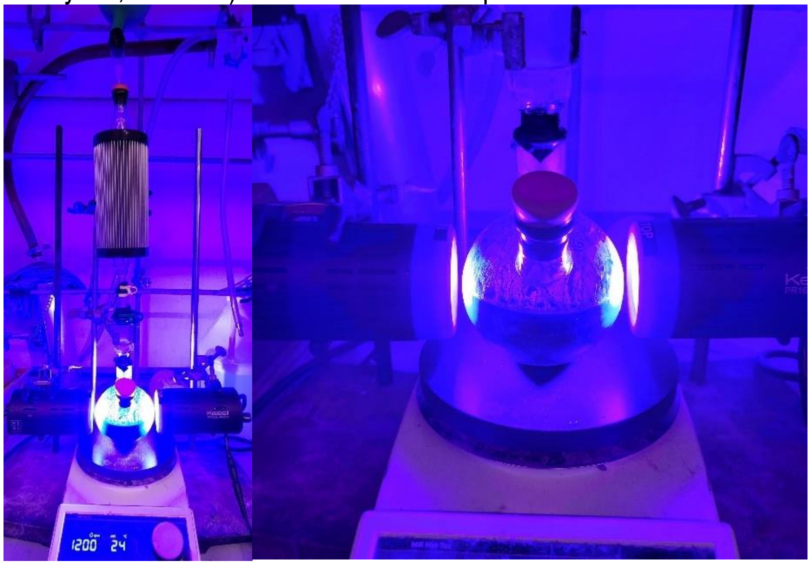

Figure S8. Setup of photochemical reaction to afford **45**.

^{1}H NMR (400 MHz, Chloroform-*d*) δ 7.33 (d, *J* = 8.7 Hz, 2H), 6.94 (d, *J* = 8.7 Hz, 2H), 5.32 (d, *J* = 3.5 Hz, 1H), 4.20 (brd, *J* = 4.6 Hz, 1H), 3.99 (t, *J* = 3.0 Hz, 1H), 3.89 – 3.85 (brm, 2H), 3.83 (s, 3H), 3.80 – 3.75 (brm, 1H), 3.05 (d, *J* = 11.9 Hz, 1H), 0.92 (s, 9H), 0.85 (s, 9H), 0.17 (s, 3H), 0.14 (s, 3H), 0.09 – 0.03 (overlapping s's, 6H)
^{13}C NMR (151 MHz, Chloroform-*d*) δ 162.2, 160.1, 131.9, 127.8, 114.4, 78.4, 76.6, 76.0, 69.6, 55.5, 54.3, 25.8, 25.7, 18.1, 17.9, -4.1, -4.6, -4.6, -4.7.
HRMS (ESI +) calc. mass for $C_{25}H_{44}O_5N^{28}Si_2$ $[M + H]^+$ 494.2753; obs. mass for $C_{25}H_{44}O_5N^{28}Si_2$ $[M + H]^+$ 494.2750.
$[\alpha]^{20}_D$: -43.3° (c = 0.180 g/100 mL, EtOAc).
Determination of d.r.: observed 20:1 d.r. based on crude ^{1}H NMR analysis of minor diastereomer peak at 5.85 ppm (d, *J* = 7.5, 1H) vs. major diastereomer peak at 5.32 ppm (d, *J* = 3.5, 1H). Assignment of diastereomers was based on relative chemical shifts and coupling constants in literature precedent by *Yoon, T. P. et al.* ***2018*** where the diastereomer of **11major** C–H$_{benzylic}$ peak was relatively more shielded and had smaller coupling (5.19 (d, *J* = 4.2 Hz, 1 H)) than **11minor** C–H$_{benzylic}$ peak (5.77 (d, *J* = 7.8 Hz, 1 H)).[30]

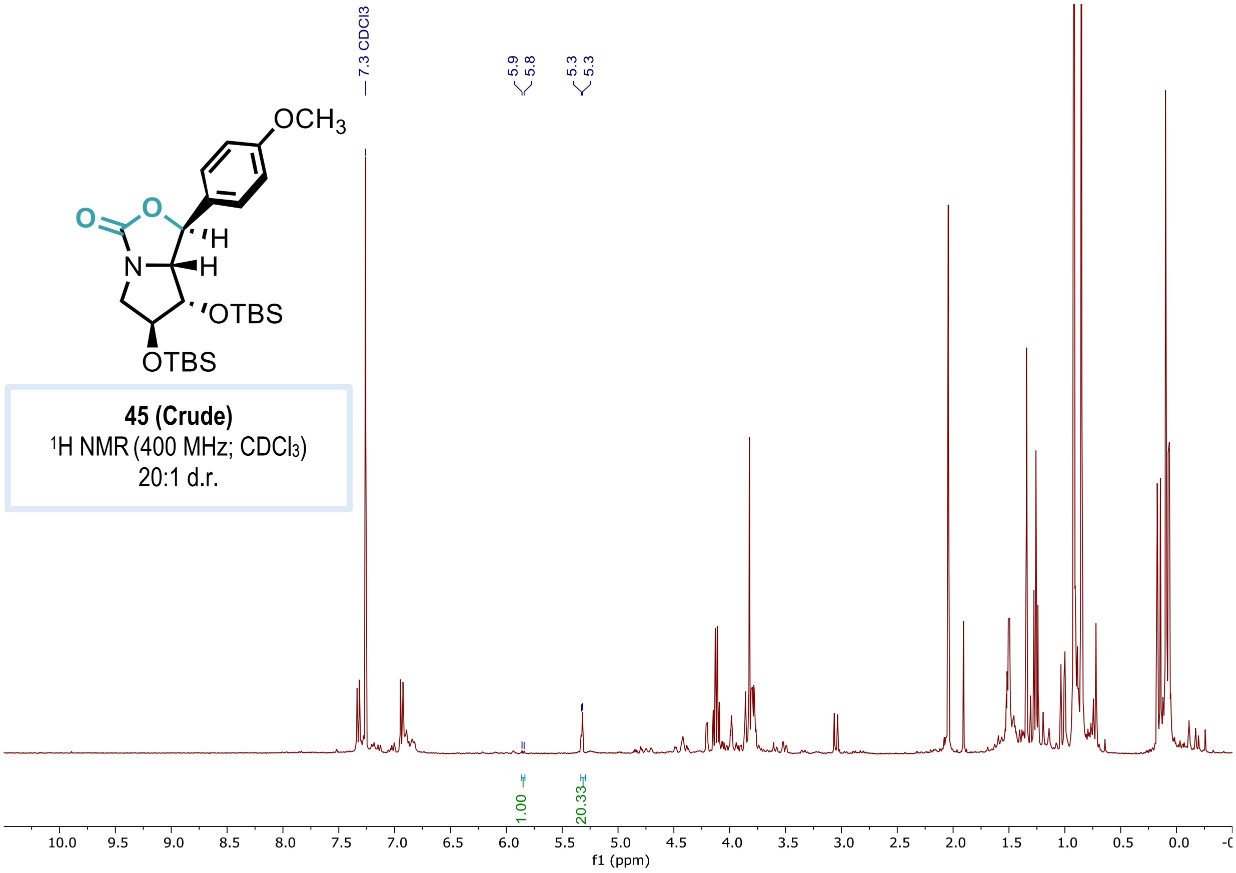

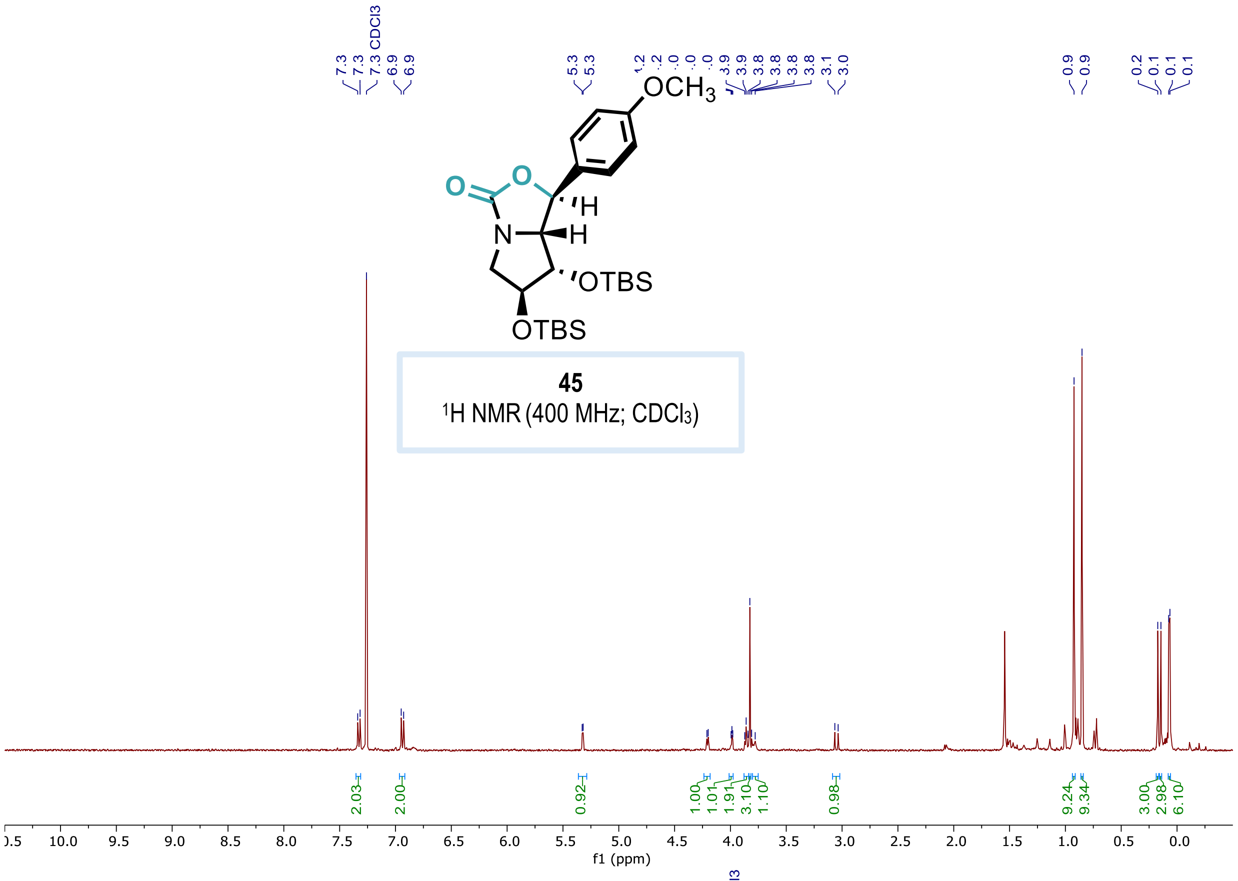
OCH3
O
O
N
H
H
OTBS
OTBS
45
1H NMR (400 MHz; CDCl3)
f1 (ppm)

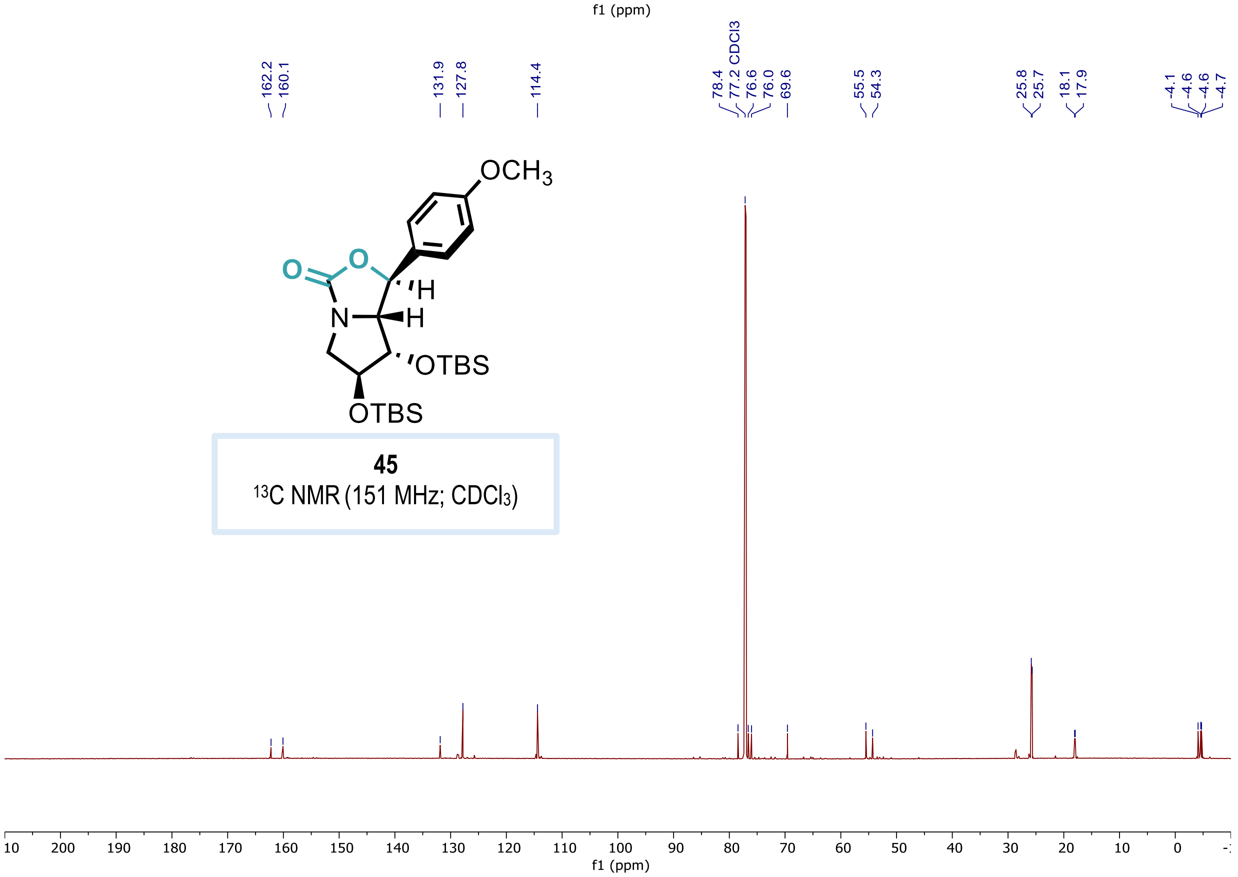
OCH3
O
O
N
H
H
OTBS
OTBS
45
13C NMR (151 MHz; CDCl3)
f1 (ppm)

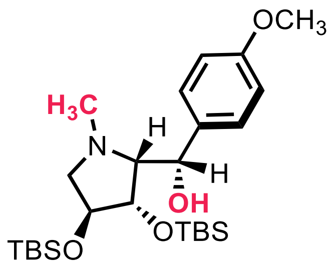

((2R,3S,4S)-3,4-bis((tert-butyldimethylsilyl)oxy)-1-methylpyrrolidin-2-yl)(4-methoxyphenyl)methanol (46)

Adapted as a modified procedure by Gilbert, J. C. et al. ***1994*** *& Cuny, G. D. et al.* ***2015***:[31, 32] To a flame-dried vial charged with a stir-bar was added (1S,6S,7S,7aR)-6,7-bis((tert-butyldimethylsilyl)oxy)-1-(4-methoxyphenyl)tetrahydro-1H,3H-pyrrolo[1,2-c]oxazol-3-one (5.40 g, 1 equiv, 10.9 mmol). The reaction vessel was sealed with a septum and backfilled with nitrogen three times and then capped with a nitrogen balloon. Dichloromethane (929 mg, 72.9 mL, 0.15 molar, 1 equiv, 10.9 mmol) was added to the reaction solution and then the reaction vessel cooled using a saltwater ice bath and allowed to stir in the cooling bath for at least 10 minutes. To the cooled solution was slowly added DIBAL-H, 1.0 M in THF (9.33 g, 65.6 mL, 1 molar, 6 equiv, 65.6 mmol). The reaction solution was stirred in the cooling bath for 1.5 hours.

After the elapsed time, the cooled reaction solution was quenched by slowly adding saturated sodium potassium tartrate solution to the cooled stirring solution and then followed by dilution with EtOAc resulting in an emulsion, the emulsion stirred vigorously for at least 15 minutes, the aqueous solution extracted with EtOAc three times, the combined organics dried with Na_2SO_4, vacuum filtered through a plug of celite, and the filtrate concentrated *in vacuo* at 55 ^oC.

The crude was dry-loaded onto SiO_2 and subjected to SiO_2 flash chromatography using hexanes/EtOAc (25 g Biotage Sfär DLV column as the running column and 50 g Biotage Sfär DLV column for the dry-load; 0% 3CV → 2% 3CV → 5% 3CV → 10% 3CV → 15% 3CV → 20% 3CV → 30% 3CV → 40% 3CV → 50% 3CV; 35 mL/min flowrate; 254/280 nm detector; POI elutes at 10% EtOAc; TLC conditions 1:1 hexanes/EtOAc, POI spot drags between 0.1 to 0.3 Rf and stains yellow with $KMnO_4$ with heat, major byproduct spot is at 0.5 Rf and stains white with $KMnO_4$ with heat; strangely, the major byproduct elutes after POI even though it has a higher Rf on TLC). Fractions that contained a mixture of desired product and byproduct were resubjected to SiO_2 chromatography.

The collected fractions were concentrated *in vacuo* at 55 ^oC to afford (S)-((2R,3S,4S)-3,4-bis((tert-butyldimethylsilyl)oxy)-1-methylpyrrolidin-2-yl)(4-methoxyphenyl)methanol (2.963 g, 6.150 mmol, 56.2% yield) as a yellow oil.

^{1}H NMR (400 MHz, Chloroform-*d*) δ 7.30 (d, *J* = 8.4 Hz, 2H), 6.87 (d, *J* = 8.4 Hz, 2H), 4.89 (brs, 1H), 4.19 – 4.03 (m, 2H), 3.94 (brs, 1H), 3.80 (s, 3H), 3.23 (dd, *J* = 9.6, 5.0 Hz, 1H), 2.91 (dd, *J* = 7.5, 1.7 Hz, 1H), 2.24 (dd, *J* = 9.6, 6.9 Hz, 1H), 1.96 (s, 3H), 0.94 (s, 9H), 0.89 (s, 9H), 0.14 (s, 6H), 0.08 (s, 3H), 0.06 (s, 3H).
^{13}C NMR (201 MHz, Chloroform-*d*) δ 158.5, 136.2, 126.7, 113.7, 80.2, 72.7, 68.9, 60.6, 55.4, 44.8, 26.0, 18.1, 18.1, -4.1, -4.4, -4.4, -4.5.
HRMS (ESI +) calc. mass for $C_{25}H_{48}O_4N^{28}Si_2$ $[M + H]^+$ 482.3116; obs. mass for $C_{25}H_{48}O_4N^{28}Si_2$ $[M + H]^+$ 482.3116.
$[\alpha]^{20}_D$: +18.9^o (c = 0.140 g/100 mL, EtOAc).

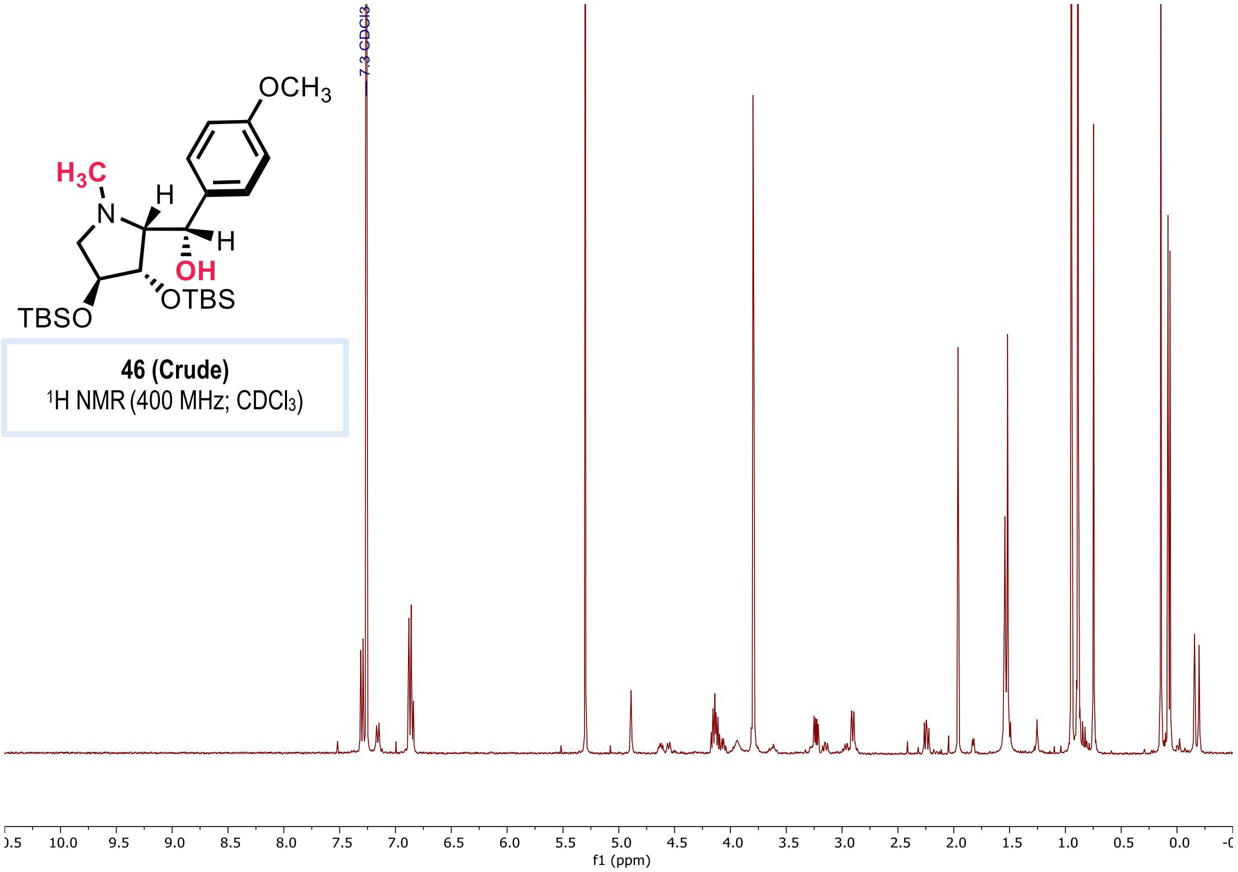

46 (Crude)
^{1}H NMR (400 MHz; $CDCl_3$)

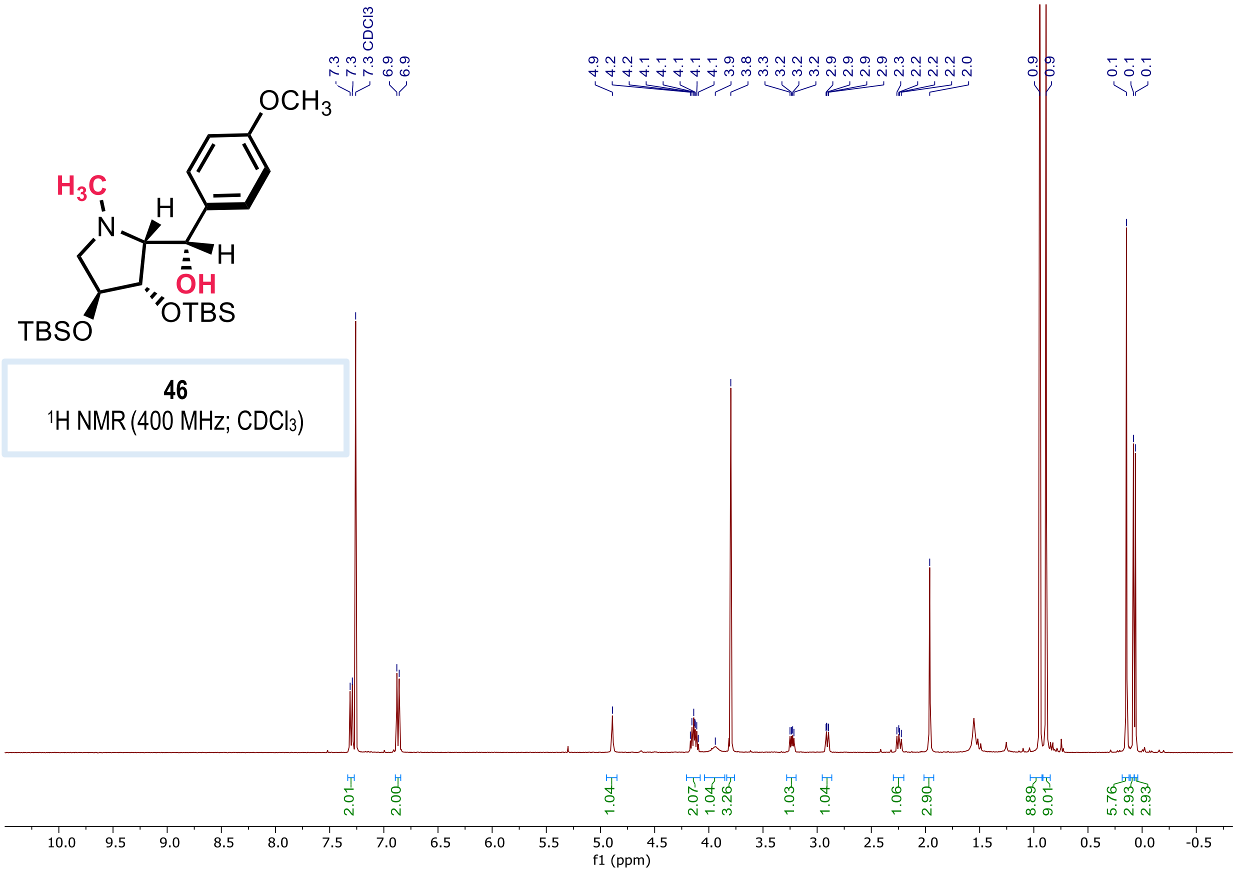

46
^{1}H NMR (400 MHz; $CDCl_3$)

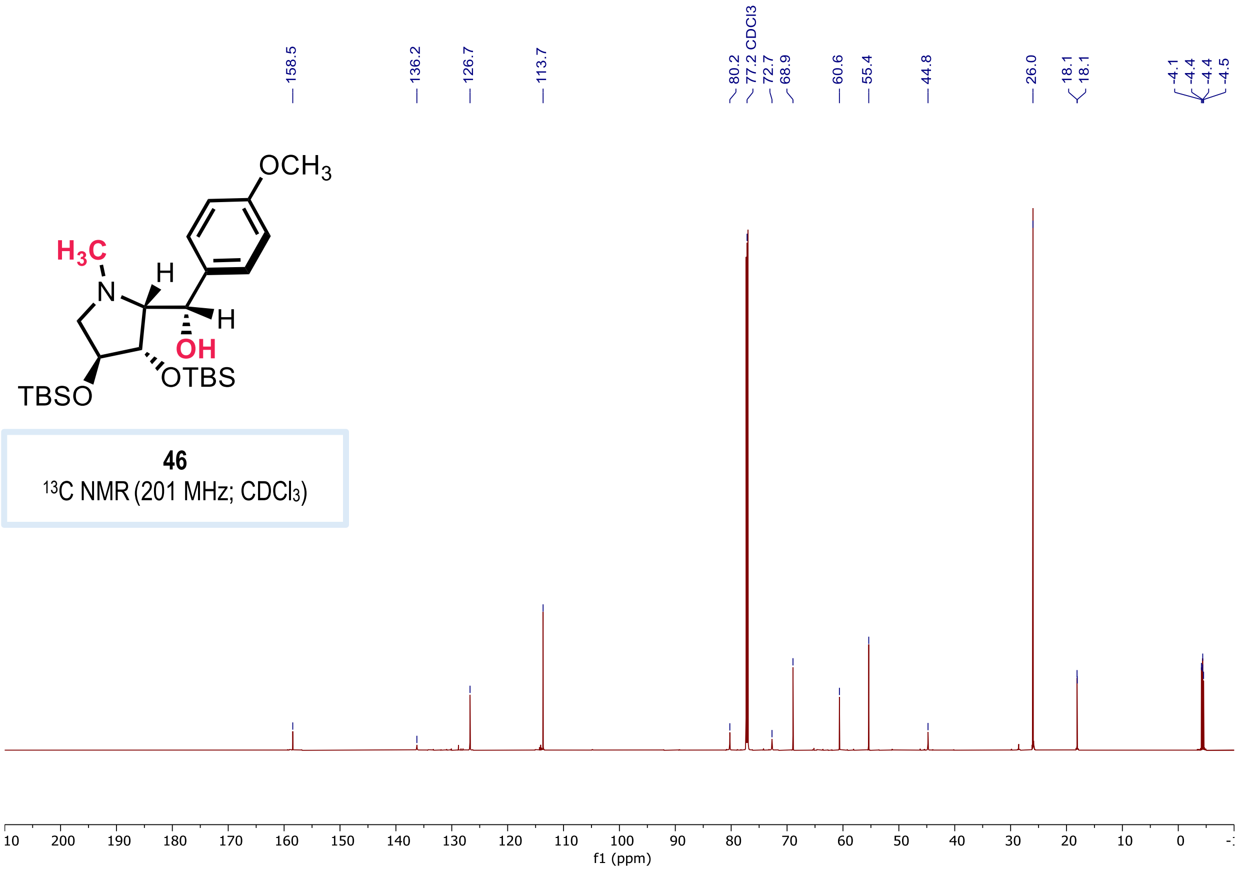

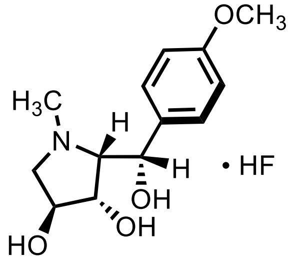

(–)-6-*epi*-Dragocin D • HF; (2R,3S,4S)-2-((S)-hydroxy(4-methoxyphenyl)methyl)-1-methylpyrrolidine-3,4-diol hydrofluoride (49)

Adapted as a modified procedure by Robins, M. J. et al. ***1992***:[33] To a flame-dried vial charged with a stir bar was added (S)-((2R,3S,4S)-3,4-bis((tert-butyldimethylsilyl)oxy)-1-methylpyrrolidin-2-yl)(4-methoxyphenyl)methanol (2.3 mg, 1 equiv, 4.8 μmol), Ammonium fluoride (2.3 mg, 13 equiv, 62 μmol), and methanol (0.15 mg, 95 μL, 0.05 molar, 1 equiv, 4.8 μmol). The reaction solution was capped with a nitrogen balloon and heated to 65 °C. The reaction solution was stirred at this temperature for 8 hours.

After the elapsed time, the reaction mixture was concentrated *in vacuo* at 55 ^{o}C, resuspend and triturate/sonicate solids with excess EtOAc and the suspension was filtered through a plug of cotton. The solids were further triturated with excess EtOAc and filtered through the same plug of cotton.

The combined EtOAc filtrate was concentrated *in vacuo* at 55 ^{o}C to afford pure (–)-6-*epi*-Dragocin D • HF (2R,3S,4S)-2-((S)-hydroxy(4-methoxyphenyl)methyl)-1-methylpyrrolidine-3,4-diol hydrofluoride (1.0 mg, 3.7 μmol, 77% yield, 90% qNMR yield from crude, single diastereomer) as a white amorphous powder. **Note:** *It was observed that the use of either celite or* SiO_2 *as the filtering aid led to decomposition of the product.*

^{1}H NMR (400 MHz, Methanol-*d*$_4$) δ 7.45 (d, *J* = 8.7 Hz, 2H), 6.94 (d, *J* = 8.7 Hz, 2H), 5.14 (d, *J* = 10.2 Hz, 1H), 4.10 (brm, 1H), 4.02 (brd, *J* = 12.3 Hz, 1H), 3.80 (s, 3H), 3.76 (dd, *J* = 10.2, 3.1 Hz, 1H), 3.64 (brm, 1H), 3.24 (s, 3H), 3.12 (brd, *J* = 12.3 Hz, 1H).
^{13}C NMR (201 MHz, Methanol-*d*$_4$) δ 161.4, 134.5, 129.2, 114.9, 78.5, 76.7, 75.9, 71.5, 64.0, 55.7, 55.6, 47.5.
^{19}F NMR (376 MHz, Methanol-*d*$_4$) δ -155.8, -155.8, -155.8, -155.9.
HRMS (ESI +) calc. mass for $C_{13}H_{20}O_4N$ [M - F]$^+$ 254.1387; obs. mass for $C_{13}H_{20}O_4N$ [M - F]$^+$ 254.1383.
$[\alpha]^{20}_{D}$: -0.9° (c = 0.110 g/100 mL, MeOH).

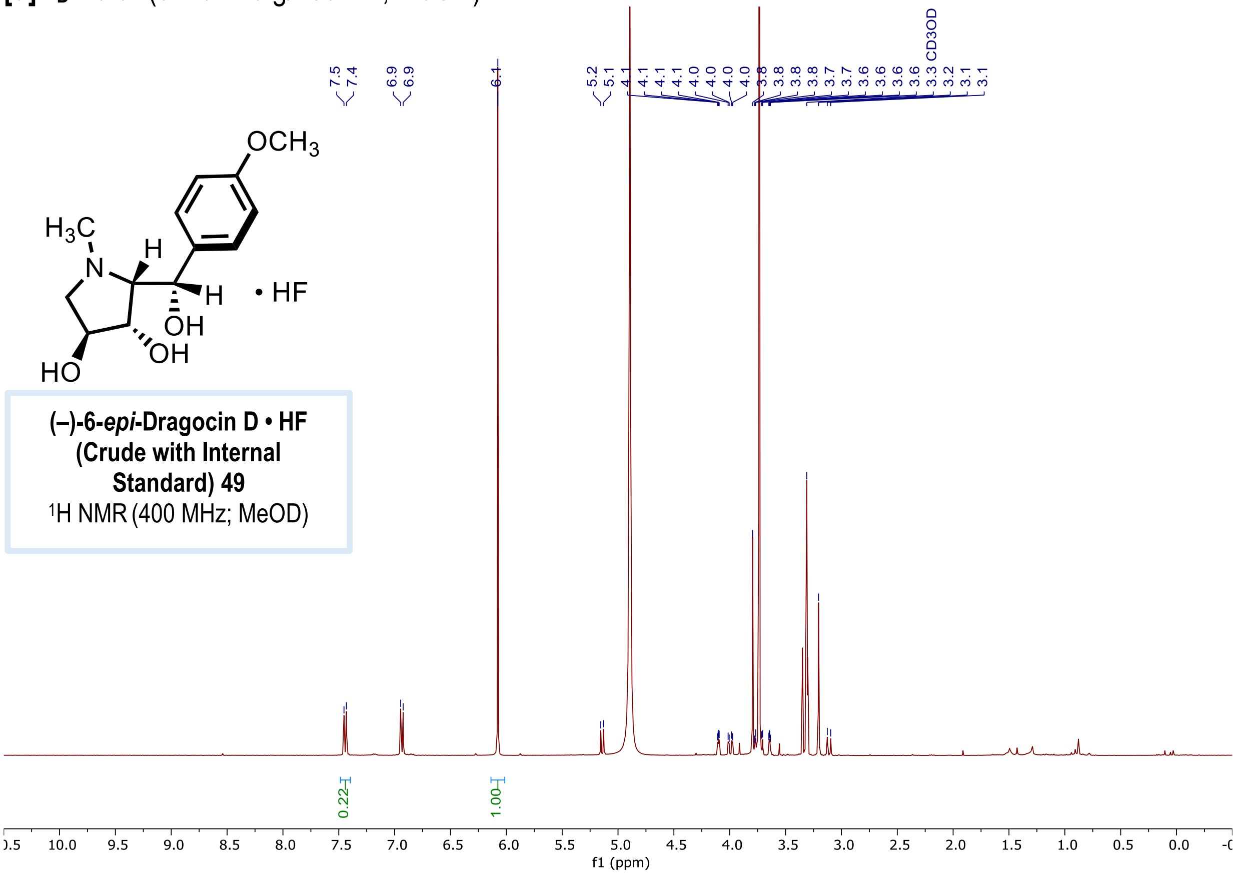

Figure S9. 1,3,5-trimethoxybenzene was added to neat crude material and quantitative ^{1}H NMR analysis integrating 6.1 ppm (IS Ar-H s, 3H) and 7.44 ppm (product Ar-H d, *J* = 8.7 Hz, 2H) was performed in MeOD. Quantitative NMR yield was assessed to be 90% yield.

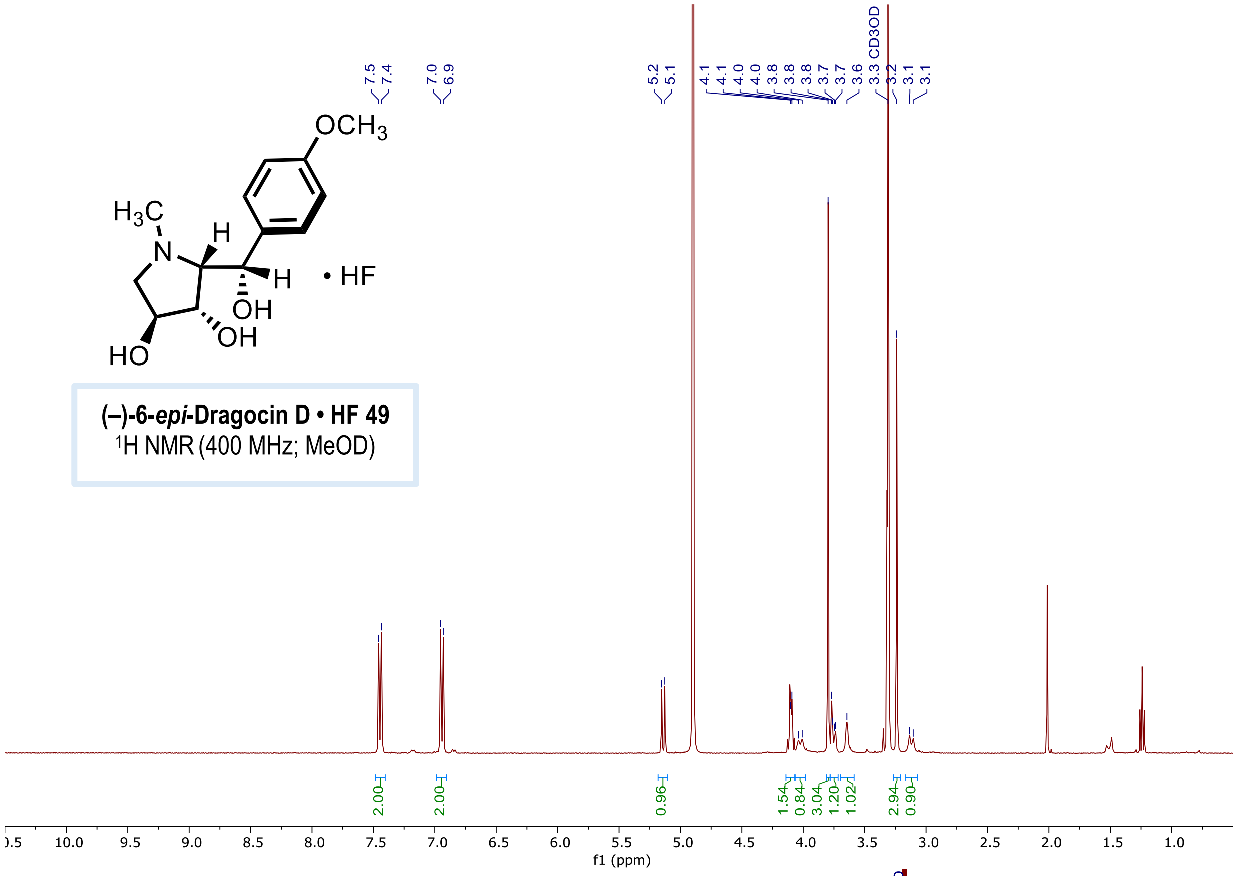
OCH3
H3C
N
H
H
OH
OH
HO
• HF
(–)-6-*epi*-Dragocin D • HF 49
1H NMR (400 MHz; MeOD)
7.5
7.4
7.0
6.9
5.2
5.1
4.1
4.1
4.0
4.0
3.8
3.8
3.8
3.7
3.6
3.3 CD3OD
3.2
3.1
3.1
2.00
2.00
0.96
1.54
0.84
3.04
1.20
1.02
2.94
0.90
f1 (ppm)

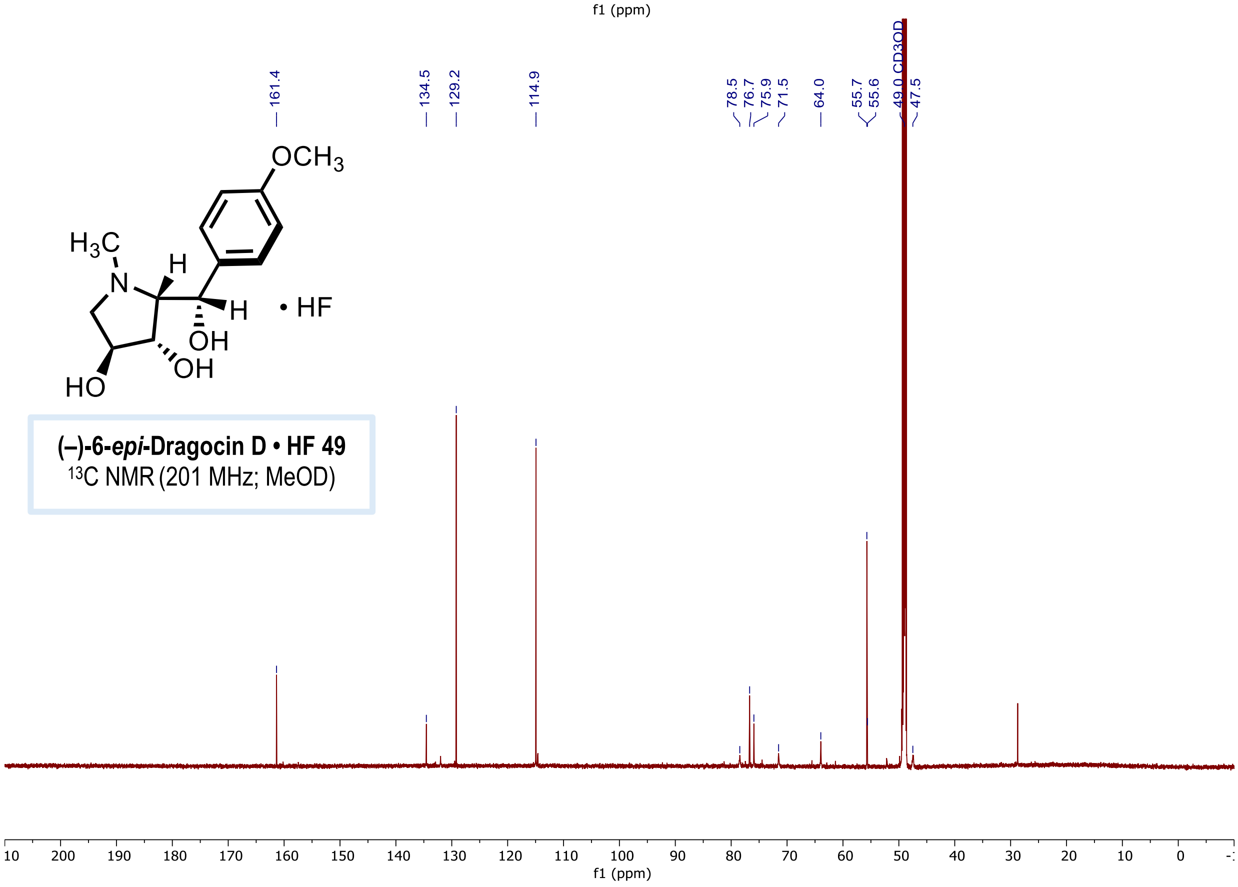
OCH3
H3C
N
H
H
OH
OH
HO
• HF
(–)-6-*epi*-Dragocin D • HF 49
13C NMR (201 MHz; MeOD)
161.4
134.5
129.2
114.9
78.5
76.7
75.9
71.5
64.0
55.7
55.6
49.0 CD3OD
47.5
f1 (ppm)

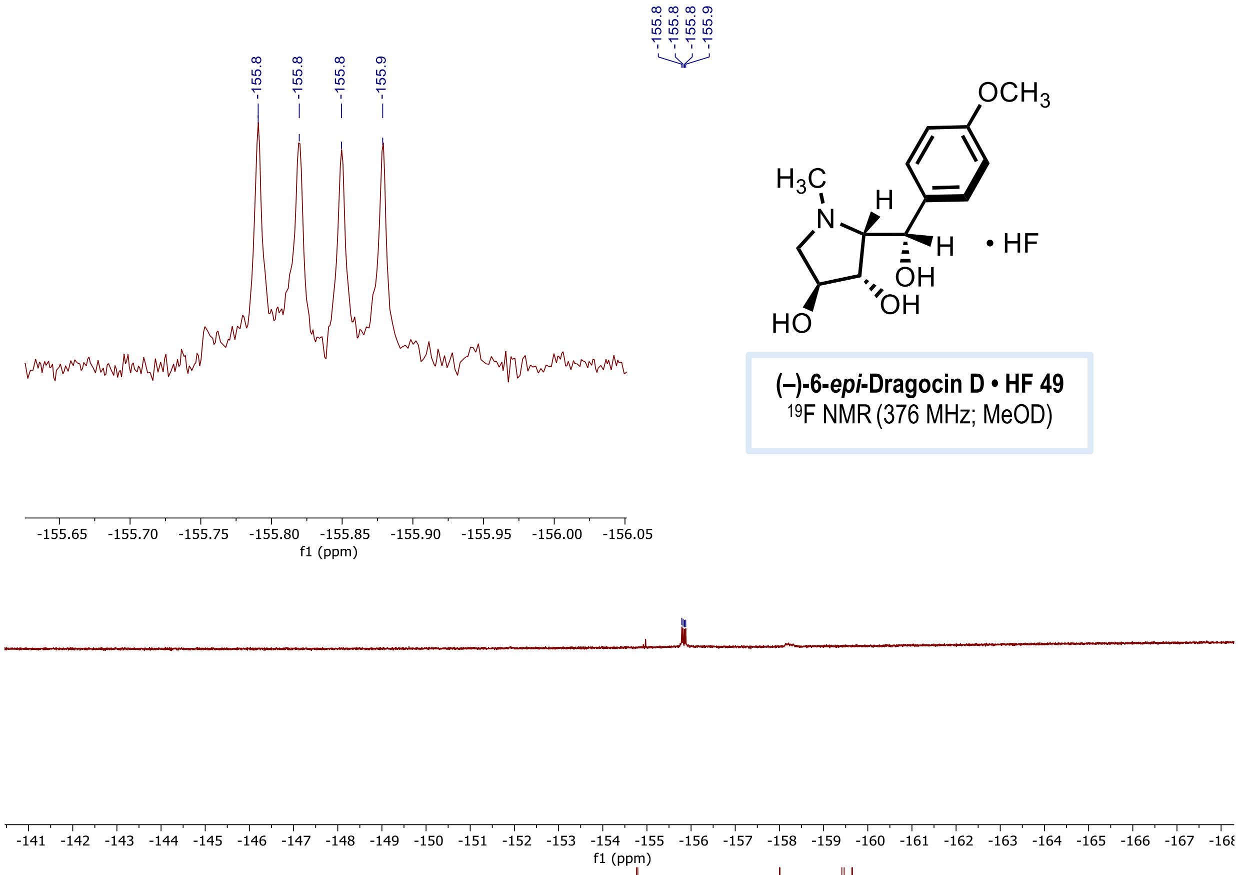

(–)-6-*epi*-Dragocin D • HF 49
^{19}F NMR (376 MHz; MeOD)

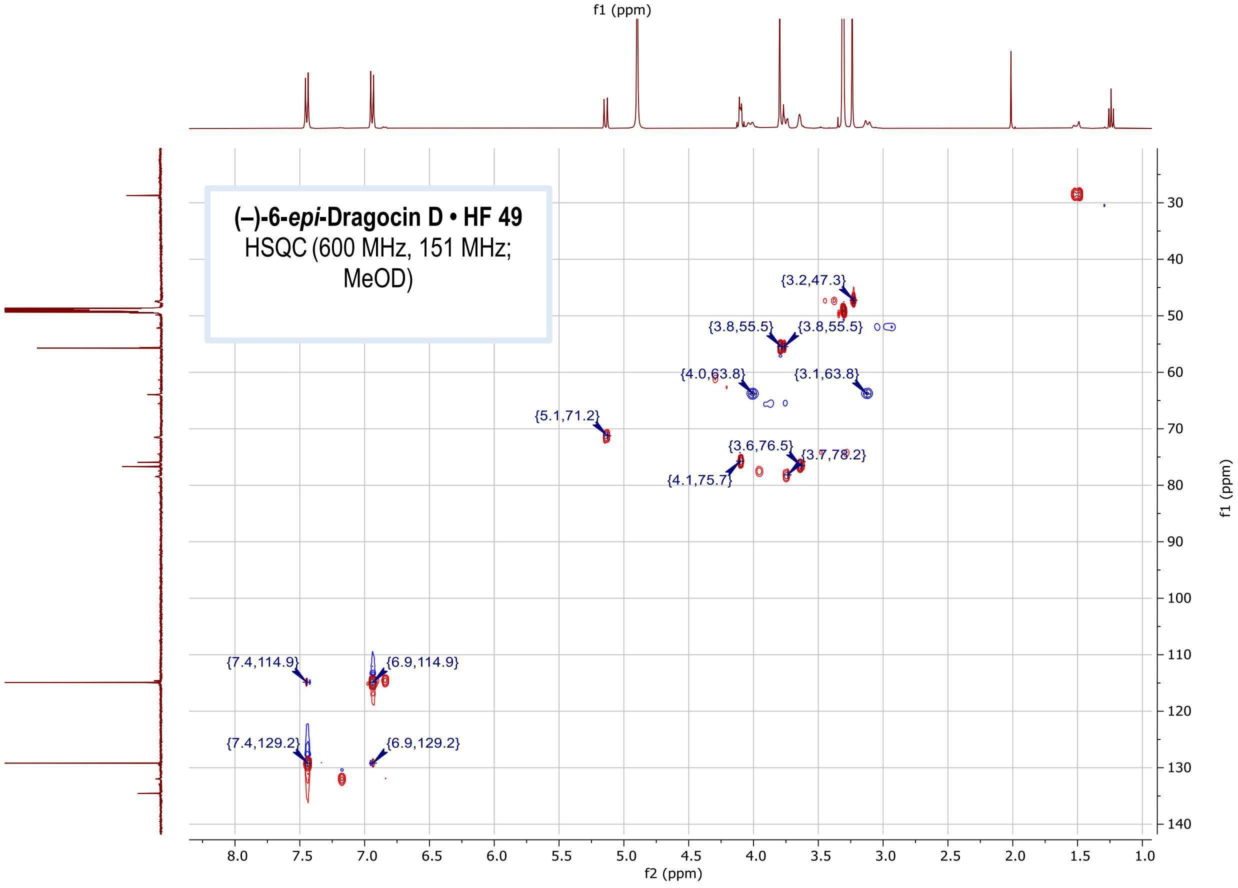

(–)-6-*epi*-Dragocin D • HF 49
HSQC (600 MHz, 151 MHz; MeOD)

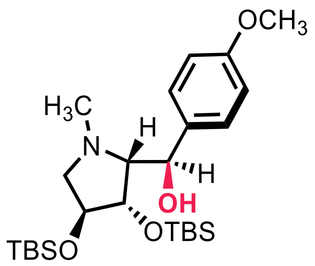

(R)-((2R,3S,4S)-3,4-bis((tert-butyldimethylsilyl)oxy)-1-methylpyrrolidin-2-yl)(4-methoxyphenyl)methanol (47)

[Swern Oxidation] - *Adapted as a modified procedure by Corey, E. J. et al.* ***2004***:[34] To a flame-dried vial charged with a stirbar was added dichloromethane (51.8 mg, 1.22 mL, 0.50 molar, 1 equiv, 610 µmol) and oxalyl chloride (116 mg, 80.1 µL, 1.5 equiv, 915 µmol). The reaction solution was cooled to -78 ^{o}C using an acetone/dry-ice bath and stirred for at least 10 minutes. To the cooled reaction solution was added DMSO (119 mg, 108 µL, 2.5 equiv, 1.53 mmol) slowly drop-wise. After complete addition, the reaction mixture was allowed to stir at -78 °C for 10 min to generate the dimethylchlorosulfonium ion.

After the elapsed time, a solution of (S)-((2R,3S,4S)-3,4-bis((tert-butyldimethylsilyl)oxy)-1-methylpyrrolidin-2-yl)(4-methoxyphenyl)methanol (294 mg, 1.22 mL, 0.50 molar in dichloromethane, 1 equiv, 610 µmol) was added to the cooled solution slowly drop-wise. After complete addition, the reaction mixture was allowed to stir at -78 °C for 50 min to form the alkoxysulfonium ion.

After the elapsed time, triethylamine (309 mg, 425 µL, 5 equiv, 3.05 mmol) was added to the cooled solution slowly drop-wise. After complete addition, the reaction mixture was allowed to stir at -78 °C for 35 min to form the ketone.

After the elapsed time, the resulting pale-yellow suspension was diluted with DI water and then diethyl ether, the solution extracted with diethyl ether three times, the combined organics dried with Na_2SO_4, filtered through a celite plug, and concentrated *in vacuo* at 35 ^{o}C to afford ((2S,3S,4S)-3,4-bis((tert-butyldimethylsilyl)oxy)-1-methylpyrrolidin-2-yl)(4-methoxyphenyl)methanone as an opaque yellow oil. The material was dissolved in diethyl ether, dried with Na_2SO_4, and filtered through a celite plug into the reaction vessel for the next step, the filtrate concentrated *in vacuo* at 35 ^{o}C to afford ((2S,3S,4S)-3,4-bis((tert-butyldimethylsilyl)oxy)-1-methylpyrrolidin-2-yl)(4-methoxyphenyl)methanone **(S19)** as a clear yellow oil. The crude was immediately subjected to the next step to minimize product decomposition. Additionally, the ketone is not stable with SiO_2 chromatography.

[$NaBH_4$ Reduction] - *Adapted as a modified procedure by Zhai, H. et al.* ***2007***:[35] To a vial charged with a stir-bar containing ((2S,3S,4S)-3,4-bis((tert-butyldimethylsilyl)oxy)-1-methylpyrrolidin-2-yl)(4-methoxyphenyl)methanone (293 mg, 1 equiv, 610 µmol) was dissolved in anhydrous methanol (19.5 mg, 4.07 mL, 0.15 molar, 1 equiv, 610 µmol). The reaction mixture was cooled to 0 ^{o}C using a saltwater ice bath. The cooled solution was stirred at 0 ^{o}C for at least 10 mins. To the cooled reaction vessel was slowly added sodium borohydride (346 mg, 15 equiv, 9.15 mmol) into the open-to-air cooled stirring reaction vessel. The reaction was stirred for 1.5 hours at 0 ^{o}C. After the elapsed time, the reaction mixture was quenched by slow addition of saturated sodium potassium tartrate solution, the diluted with dichloromethane, the organic layer washed with DI water three times, the organic layer dried with Na_2SO_4, vacuum filtered through a celite plug, and the filtrate concentrated *in vacuo* at 55 ^{o}C.

The crude was subjected to SiO_2 flash chromatography hexanes/EtOAc (10 g Biotage Sfär DLV column as the running column and 25 g Biotage Sfär DLV column for the dry-load; 0% 3CV → 2% 3CV → 5% 3CV → 10% 3CV → 15% 3CV → 20% 3CV → 30% 3CV → 40% 3CV → 50% 3CV; 25 mL/min flowrate; 254/280 nm detector; POI elutes at 5 - 10% EtOAc, the minor diastereomer elutes at 15% EtOAc; TLC conditions 1:1 hexanes/EtOAc POI spot Rf is ~0.6 and minor diastereomer spot Rf drags from 0.1 - 0.3, both spots stain yellow with KMnO4 stain with heat).

The collected fractions were concentrated *in vacuo* at 55 °C to afford (R)-((2R,3S,4S)-3,4-bis((tert-butyldimethylsilyl)oxy)-1-methylpyrrolidin-2-yl)(4-methoxyphenyl)methanol (175 mg, 363 μmol, 59.5% yield, >20:1 d.r. *enriched from chromatography*) as a clear pale-yellow oil.

^{1}H NMR (600 MHz, Chloroform-*d*) δ 7.38 (d, *J* = 8.6 Hz, 2H), 6.88 (d, *J* = 8.6 Hz, 2H), 4.81 (d, *J* = 6.6 Hz, 1H), 4.15 – 4.11 (m, 1H), 4.02 – 3.97 (m, 1H), 3.81 (s, 3H), 3.46 (dd, *J* = 10.5, 4.5 Hz, 1H), 3.34 (brs, 1H), 2.87 (t, *J* = 5.8 Hz, 1H), 2.28 (dd, *J* = 10.5, 3.6 Hz, 1H), 2.11 (s, 3H), 0.90 (s, 9H), 0.87 (s, 9H), 0.15 – -0.05 (m, 12H).
^{13}C NMR (151 MHz, Chloroform-*d*) δ 158.9, 135.5, 128.0, 113.6, 80.9, 72.6, 72.6, 62.7, 55.4, 43.9, 26.0, 26.0, 18.1, 18.0, -3.9, -4.2, -4.4, -4.9.
HRMS (ESI +) calc. mass for $C_{25}H_{48}O_4N^{28}Si_2$ [M + H]$^+$ 482.3116; obs. mass for $C_{25}H_{48}O_4N^{28}Si_2$ [M + H]$^+$ 482.3113.
$[\alpha]^{20}_D$: -9.2° (c = 0.380 g/100 mL, EtOAc).
Determination of d.r.: 15:1 d.r. based on crude ^{1}H NMR analysis of minor diastereomer peak at 4.9 ppm (brs, 1H) vs. major diastereomer peak at 4.8 ppm (d, *J* = 6.6, 1H).

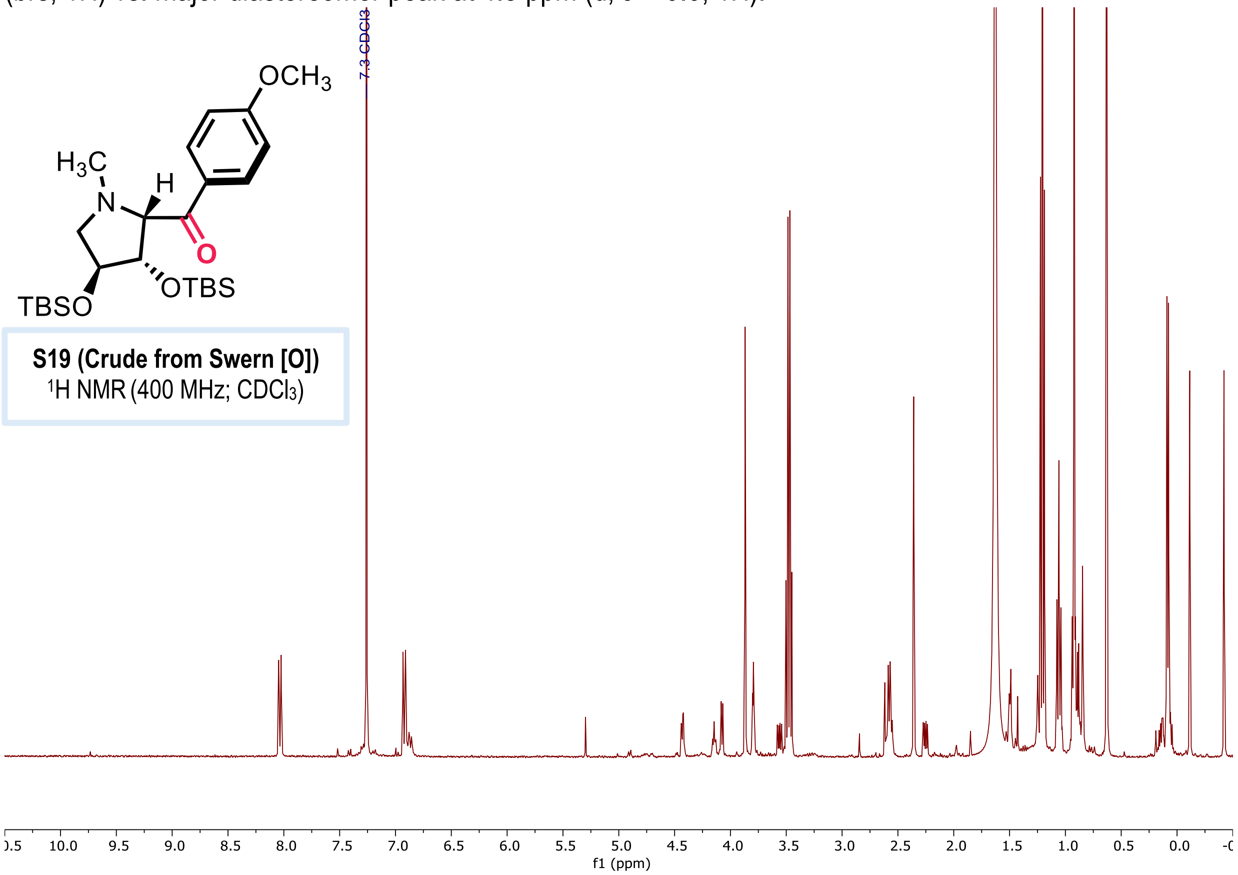

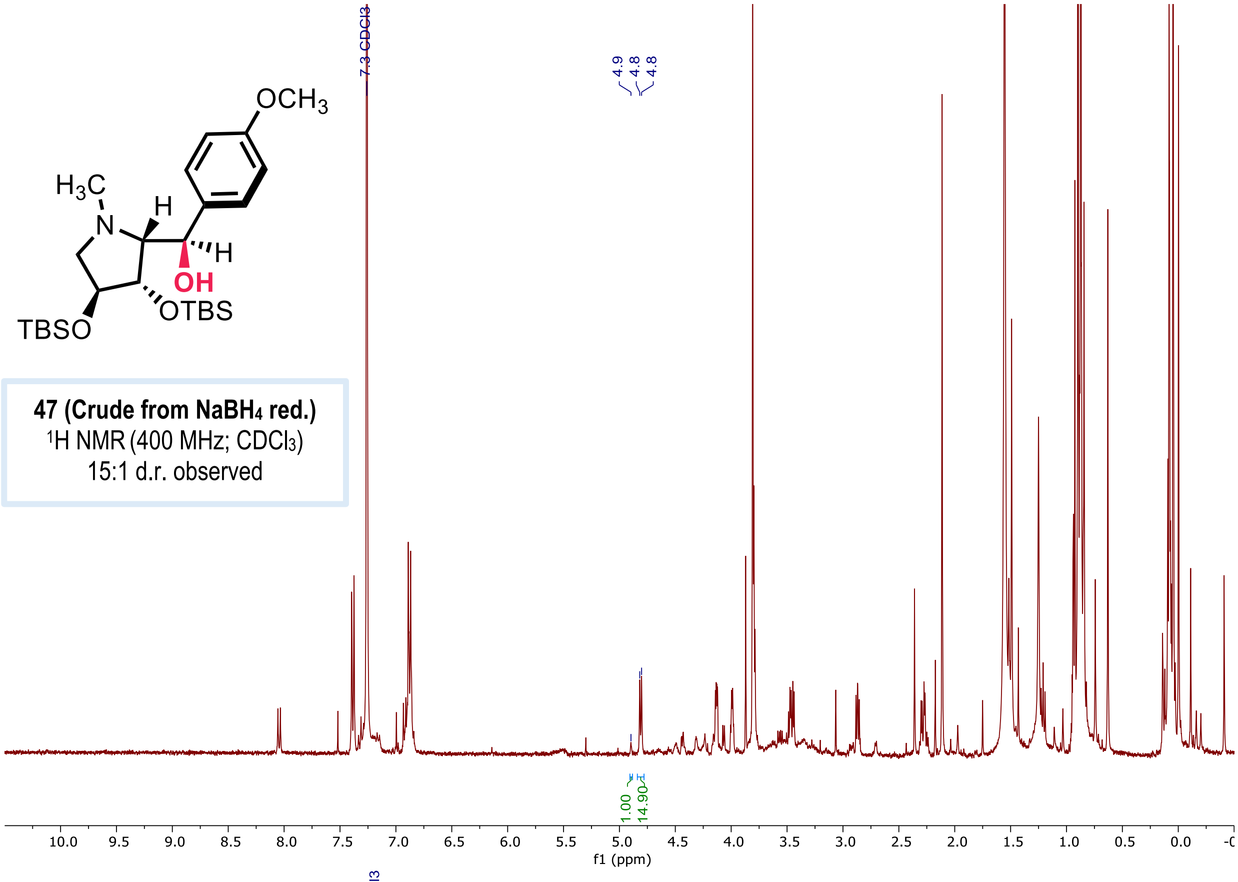

47 (Crude from NaBH4 red.)
^{1}H NMR (400 MHz; $CDCl_3$)
15:1 d.r. observed

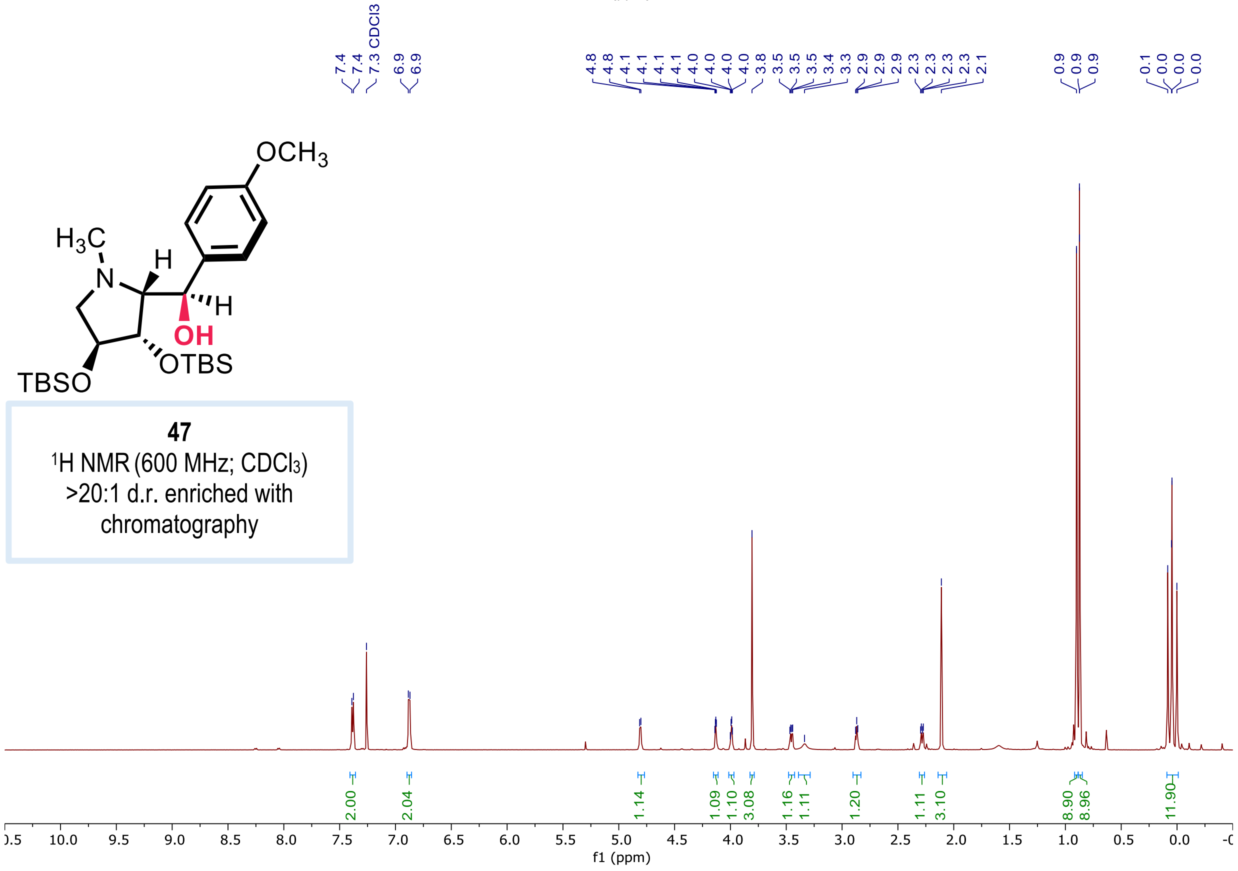

47
^{1}H NMR (600 MHz; $CDCl_3$)
>20:1 d.r. enriched with chromatography

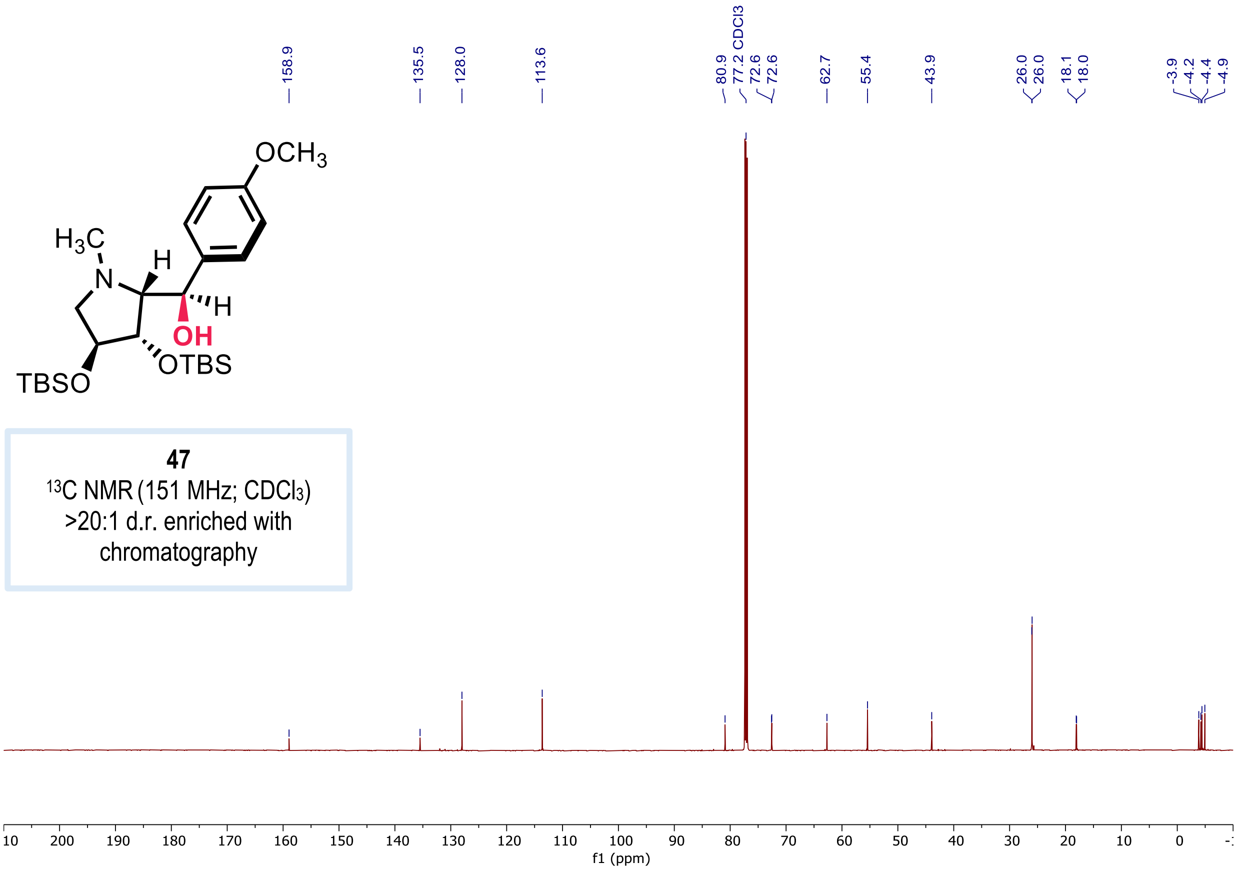

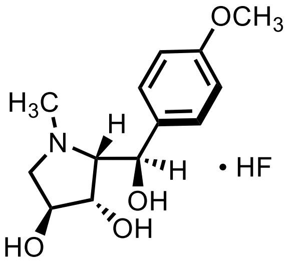

(–)-Dragocin D • HF; (2R,3S,4S)-2-((R)-hydroxy(4-methoxyphenyl)methyl)-1-methylpyrrolidine-3,4-diol hydrofluoride (48)

Adapted as a modified procedure by Robins, M. J. et al. ***1992***:[33] To a flame-dried vial charged with a stir bar was added (R)-((2R,3S,4S)-3,4-bis((tert-butyldimethylsilyl)oxy)-1-methylpyrrolidin-2-yl)(4-methoxyphenyl)methanol (9.0 mg, 1 equiv, 19 µmol), Ammonium fluoride (9.0 mg, 13 equiv, 0.24 mmol), and methanol (0.60 mg, 0.37 mL, 0.05 molar, 1 equiv, 19 µmol). The reaction solution was capped with a nitrogen balloon and heated to 65 °C. The reaction solution was stirred at this temperature for 8 hours.

After the elapsed time, the reaction mixture was concentrated *in vacuo* at 55 °C, resuspend and triturate/sonicate solids with excess EtOAc and the suspension was filtered through a plug of cotton. The solids were further triturated with excess EtOAc and filtered through the same plug of cotton.

The combined EtOAc filtrate was concentrated *in vacuo* at 55 °C to afford pure (–)-Dragocin D • HF (2R,3S,4S)-2-((R)-hydroxy(4-methoxyphenyl)methyl)-1-methylpyrrolidine-3,4-diol hydrofluoride (3.7 mg, 14 µmol, 72% yield, 86% qNMR yield from crude, single diastereomer) as a clear amorphous solid. **Note:** *It was observed that the use of either celite or SiO_2 as the filtering aid led to decomposition of the product.*

^{1}H NMR (400 MHz, Methanol-*d*$_4$) δ 7.45 (d, *J* = 8.7 Hz, 2H), 6.98 (d, *J* = 8.7 Hz, 2H), 5.05 (d, *J* = 7.0 Hz, 1H), 4.17 (brm, 1H), 4.11 (brd, *J* = 4.5 Hz, 1H), 3.94 (dd, *J* = 12.1, 4.5 Hz, 1H), 3.81 (s, 3H), 3.48 (brm, 1H), 2.87 (d, *J* = 12.1 Hz, 1H), 2.49 (s, 3H).
^{13}C NMR (101 MHz, Methanol-*d*$_4$) δ 161.3, 134.2, 129.0, 115.2, 78.2, 75.4, 75.2, 70.7, 64.7, 55.8, 43.3.
^{19}F NMR (376 MHz, Methanol-*d*$_4$) δ -152.7.
HRMS (ESI +) calc. mass for $C_{13}H_{20}O_4N$ [M - F]$^+$ 254.1387; obs. mass for $C_{13}H_{20}O_4N$ [M - F]$^+$ 254.1383.
$[\alpha]^{20}_{D}$: -23.7° (c = 0.140 g/100 mL, MeOH).

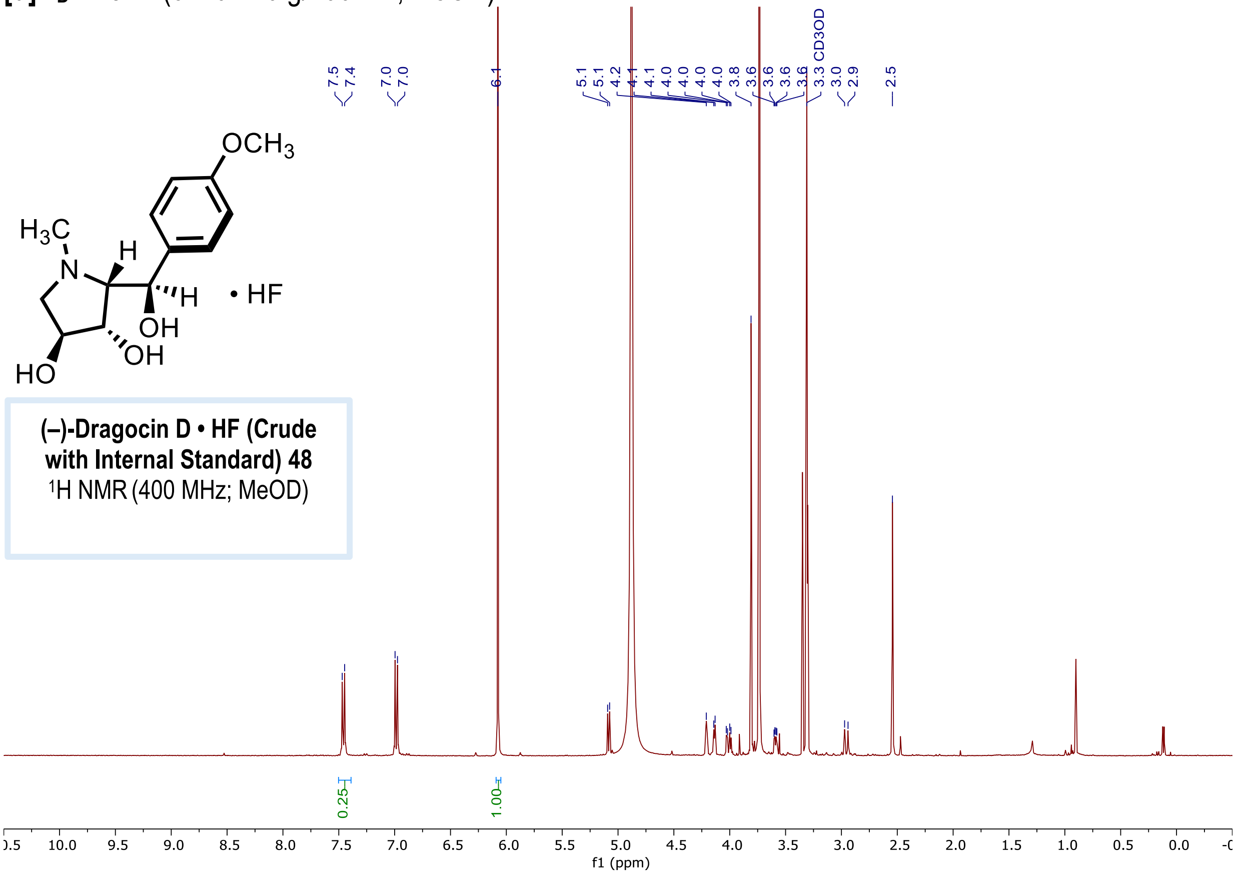

Figure S10. 1,3,5-trimethoxybenzene was added to neat crude material and quantitative ^{1}H NMR analysis integrating 6.1 ppm (IS Ar-H s, 3H) and 7.46 ppm (product Ar-H d, *J* = 8.7 Hz, 2H) was performed in MeOD. Quantitative NMR yield was assessed to be 86% yield.

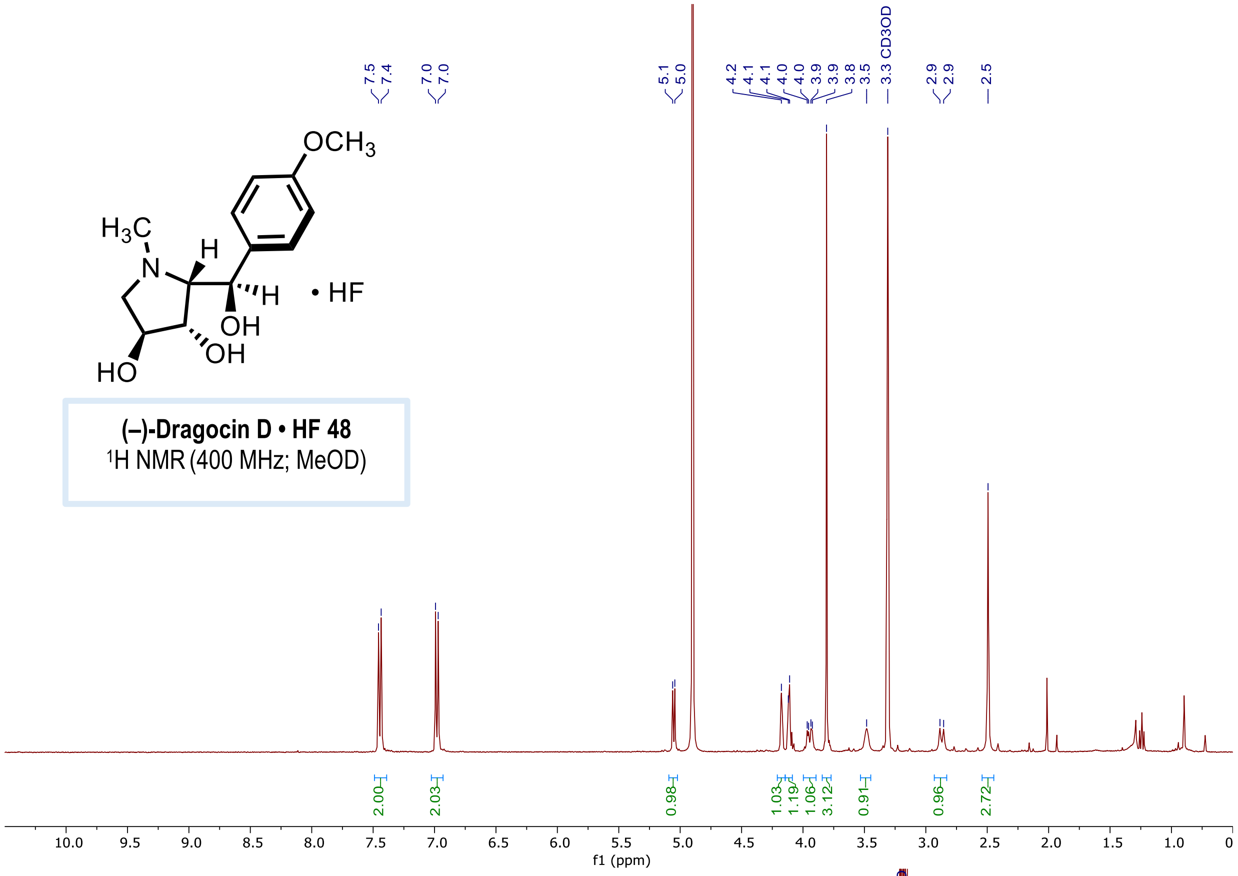

(–)-Dragocin D • HF 48
^{1}H NMR (400 MHz; MeOD)

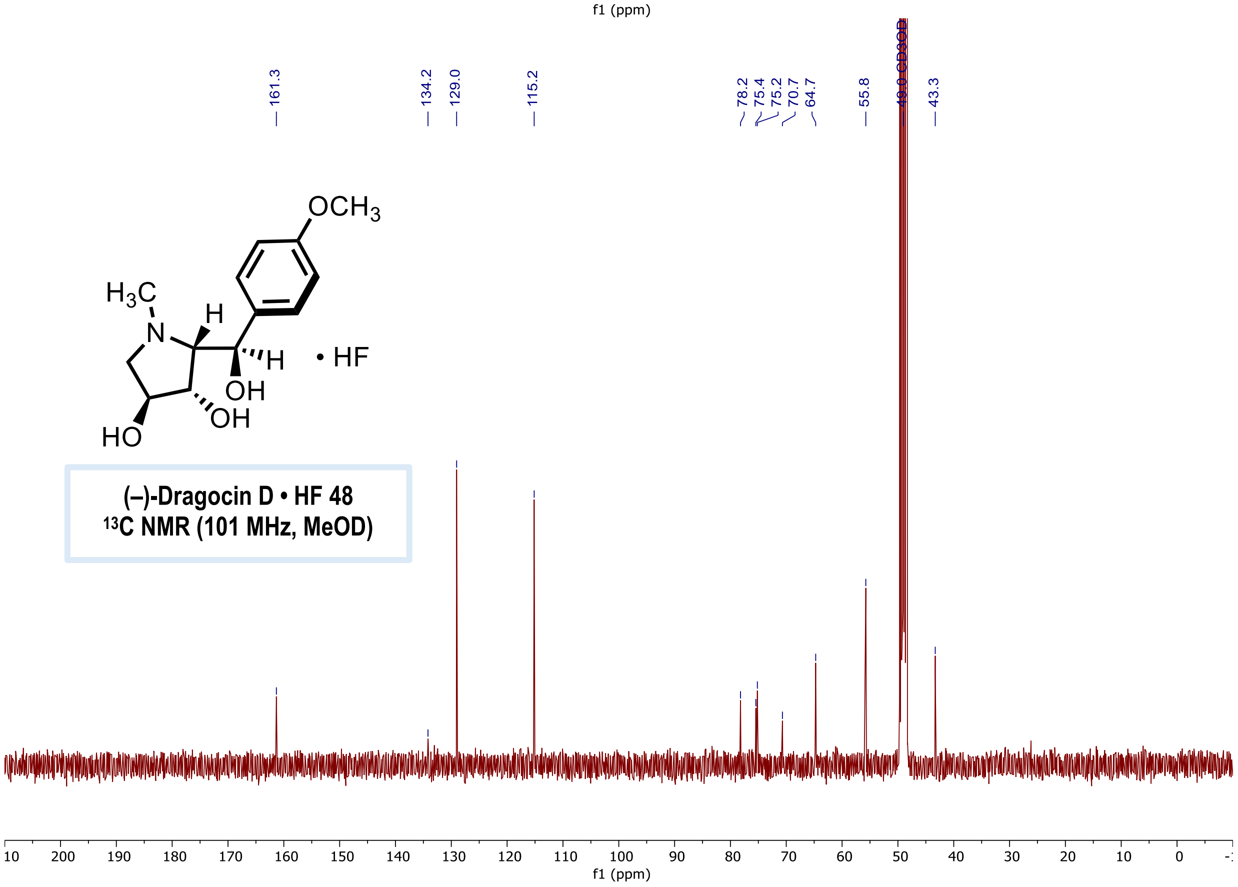

(–)-Dragocin D • HF 48
^{13}C NMR (101 MHz, MeOD)

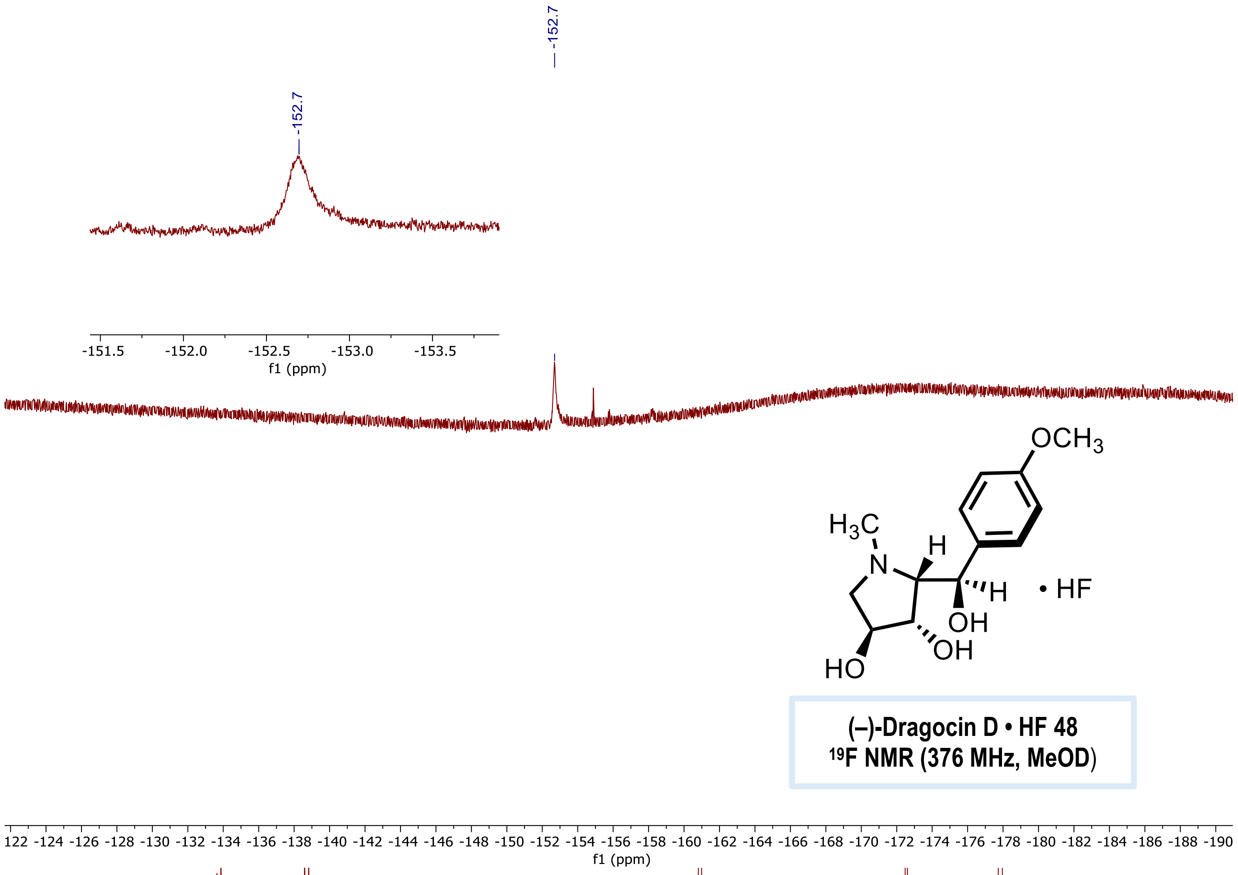
-152.7
-152.7
-151.5 -152.0 -152.5 -153.0 -153.5
f1 (ppm)
OCH3
H3C
N
H
H
• HF
OH
OH
HO
(–)-Dragocin D • HF 48
19F NMR (376 MHz, MeOD)
122 -124 -126 -128 -130 -132 -134 -136 -138 -140 -142 -144 -146 -148 -150 -152 -154 -156 -158 -160 -162 -164 -166 -168 -170 -172 -174 -176 -178 -180 -182 -184 -186 -188 -190
f1 (ppm)

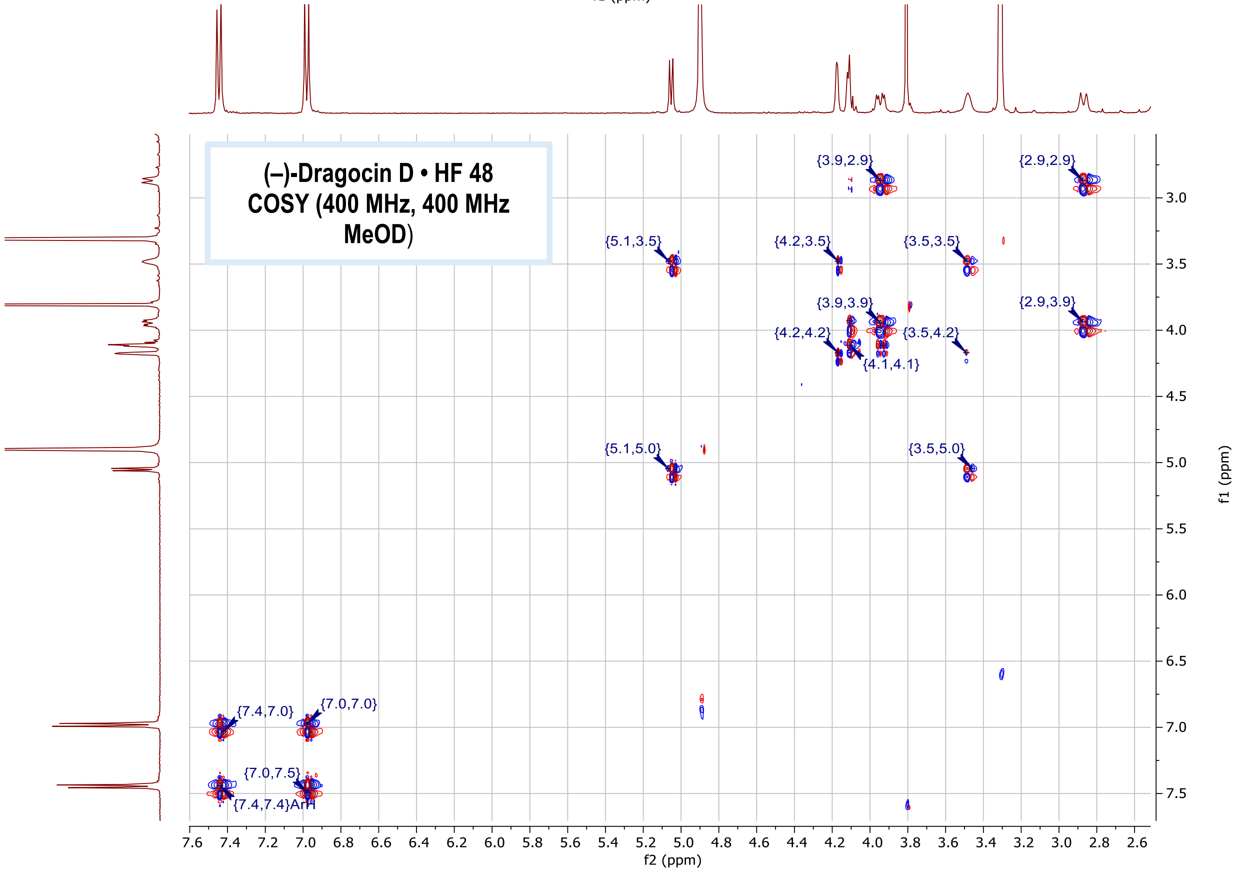
(–)-Dragocin D • HF 48
COSY (400 MHz, 400 MHz
MeOD)
{3.9,2.9}
{2.9,2.9}
{5.1,3.5}
{4.2,3.5}
{3.5,3.5}
{3.9,3.9}
{2.9,3.9}
{4.2,4.2}
{3.5,4.2}
{4.1,4.1}
{5.1,5.0}
{3.5,5.0}
{7.4,7.0}
{7.0,7.0}
{7.0,7.5}
{7.4,7.4}ArH
7.6 7.4 7.2 7.0 6.8 6.6 6.4 6.2 6.0 5.8 5.6 5.4 5.2 5.0 4.8 4.6 4.4 4.2 4.0 3.8 3.6 3.4 3.2 3.0 2.8 2.6
f2 (ppm)
3.0 3.5 4.0 4.5 5.0 5.5 6.0 6.5 7.0 7.5
f1 (ppm)

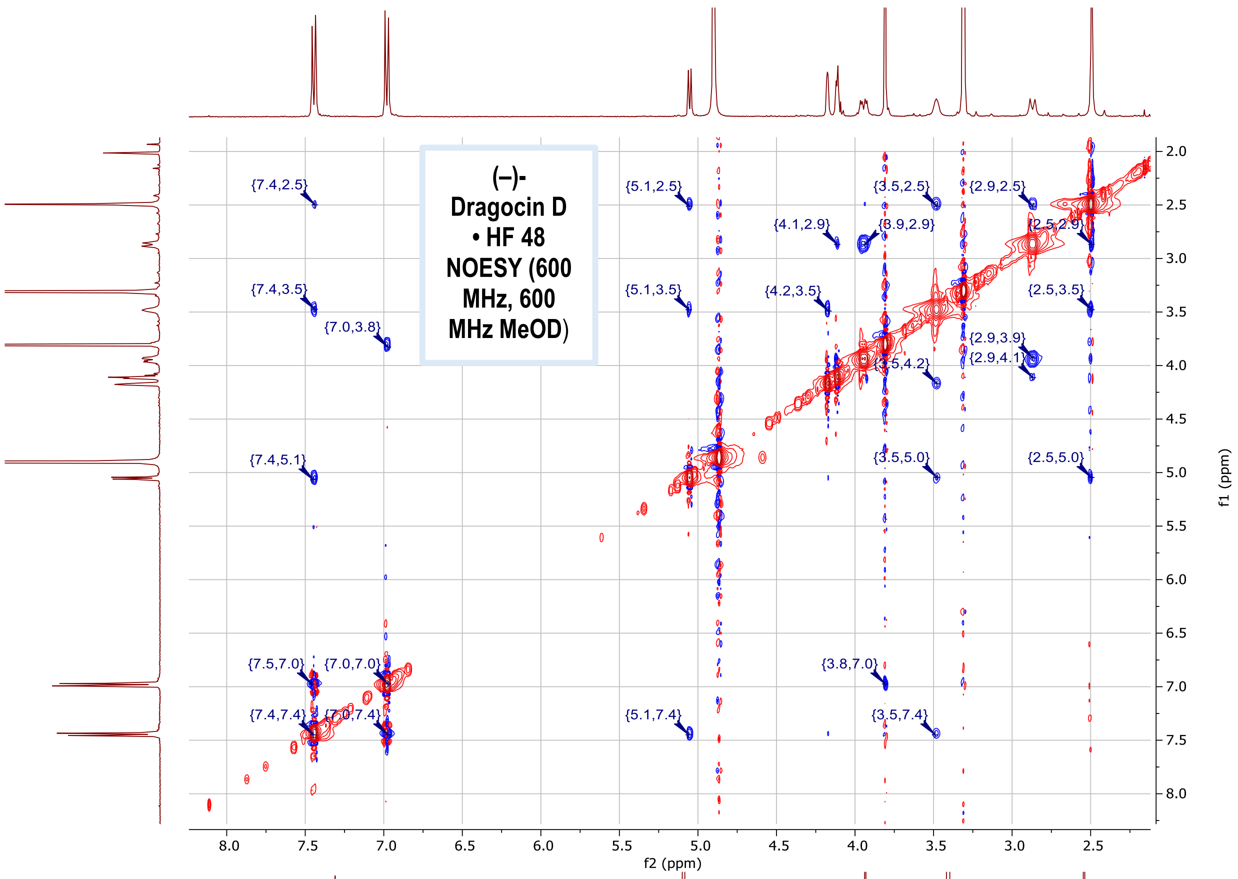
(–)-Dragocin D • HF 48 NOESY (600 MHz, 600 MHz MeOD)
{7.4,2.5}
{5.1,2.5}
{3.5,2.5}
{2.9,2.5}
{4.1,2.9}
{3.9,2.9}
{2.5,2.9}
{7.4,3.5}
{5.1,3.5}
{4.2,3.5}
{2.5,3.5}
{7.0,3.8}
{2.9,3.9}
{2.9,4.1}
{3.5,4.2}
{7.4,5.1}
{3.5,5.0}
{2.5,5.0}
{7.5,7.0}
{7.0,7.0}
{3.8,7.0}
{7.4,7.4}
{7.0,7.4}
{5.1,7.4}
{3.5,7.4}
f1 (ppm)
f2 (ppm)

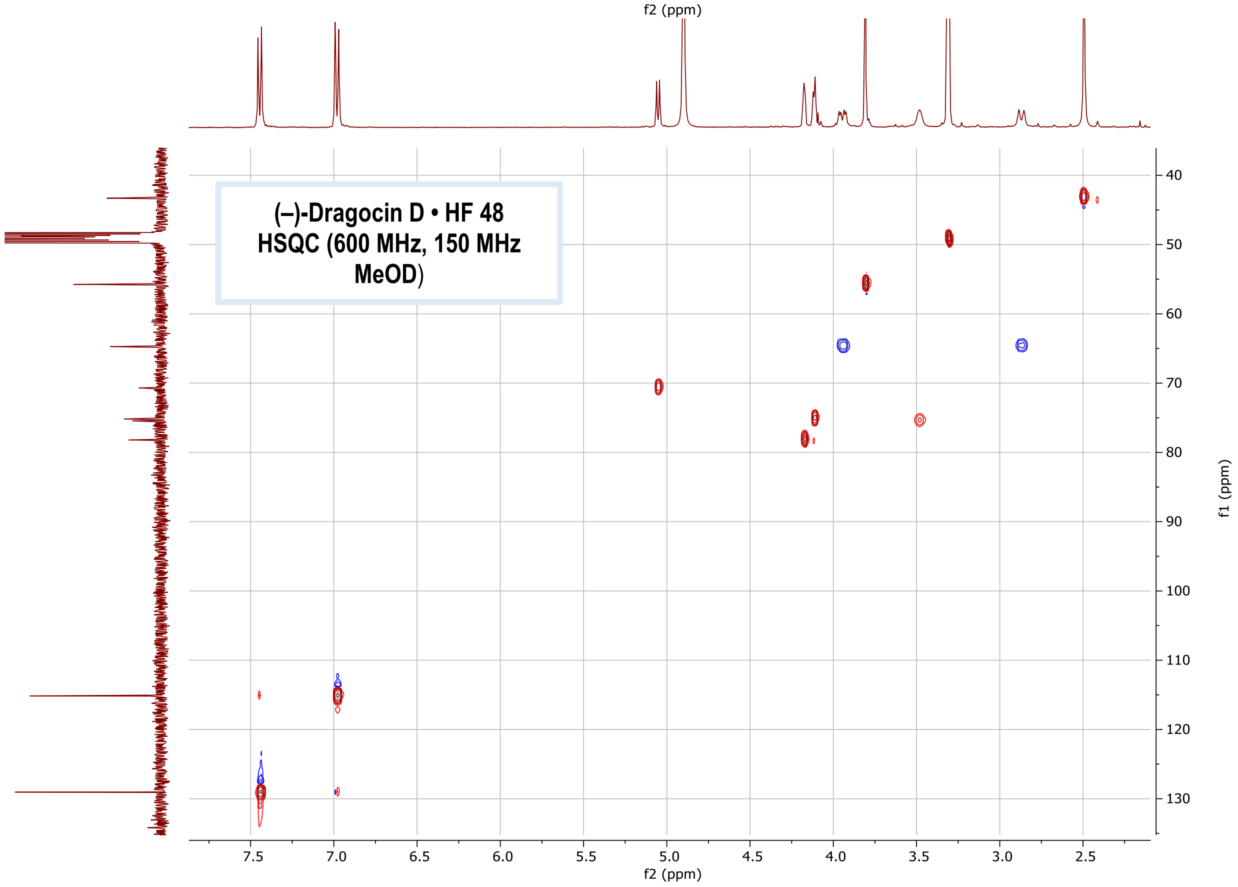
(–)-Dragocin D • HF 48 HSQC (600 MHz, 150 MHz MeOD)
f1 (ppm)
f2 (ppm)

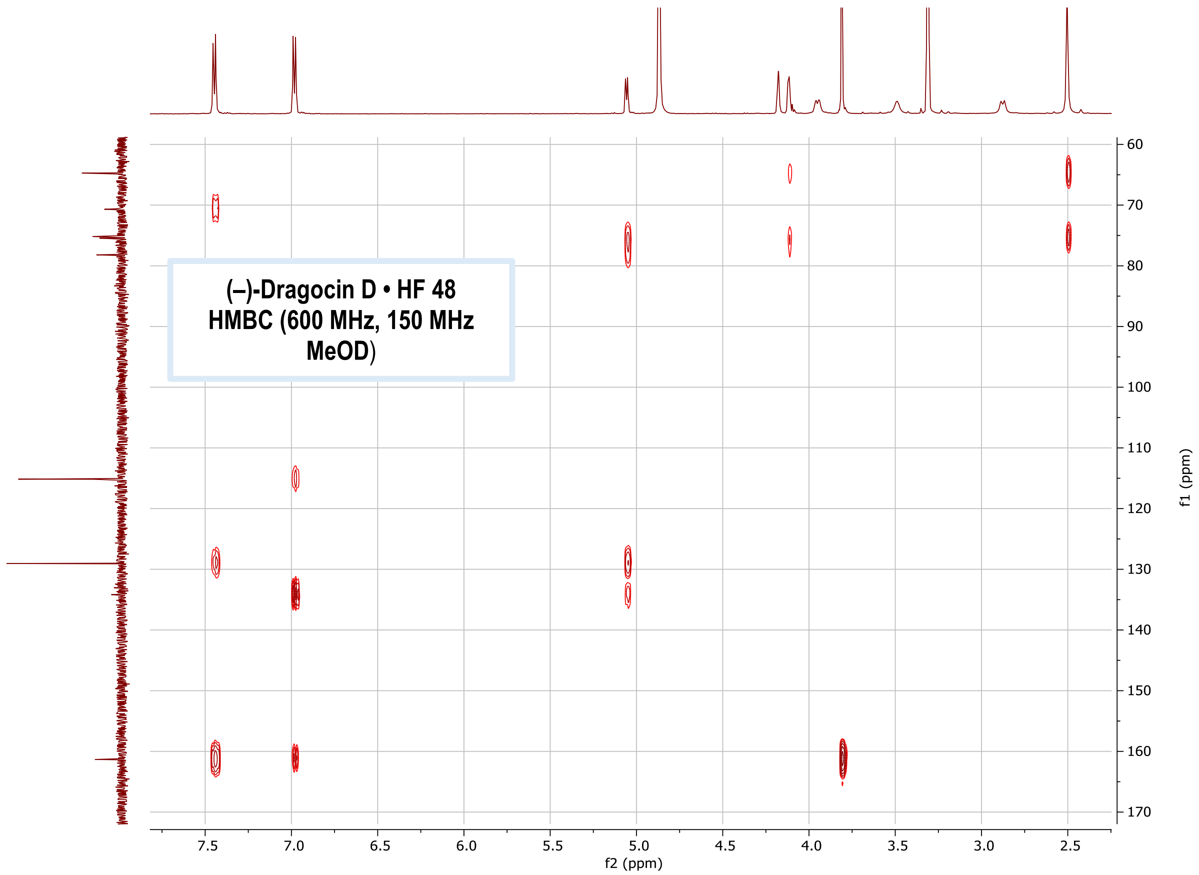
(–)-Dragocin D • HF 48
HMBC (600 MHz, 150 MHz
MeOD)
7.5
7.0
6.5
6.0
5.5
5.0
4.5
4.0
3.5
3.0
2.5
f2 (ppm)
60
70
80
90
100
110
120
130
140
150
160
170
f1 (ppm)

8. Route Scouting for the Synthesis of (–)-6-*epi*-Dragocin D • HF and (–)-Dragocin D • HF

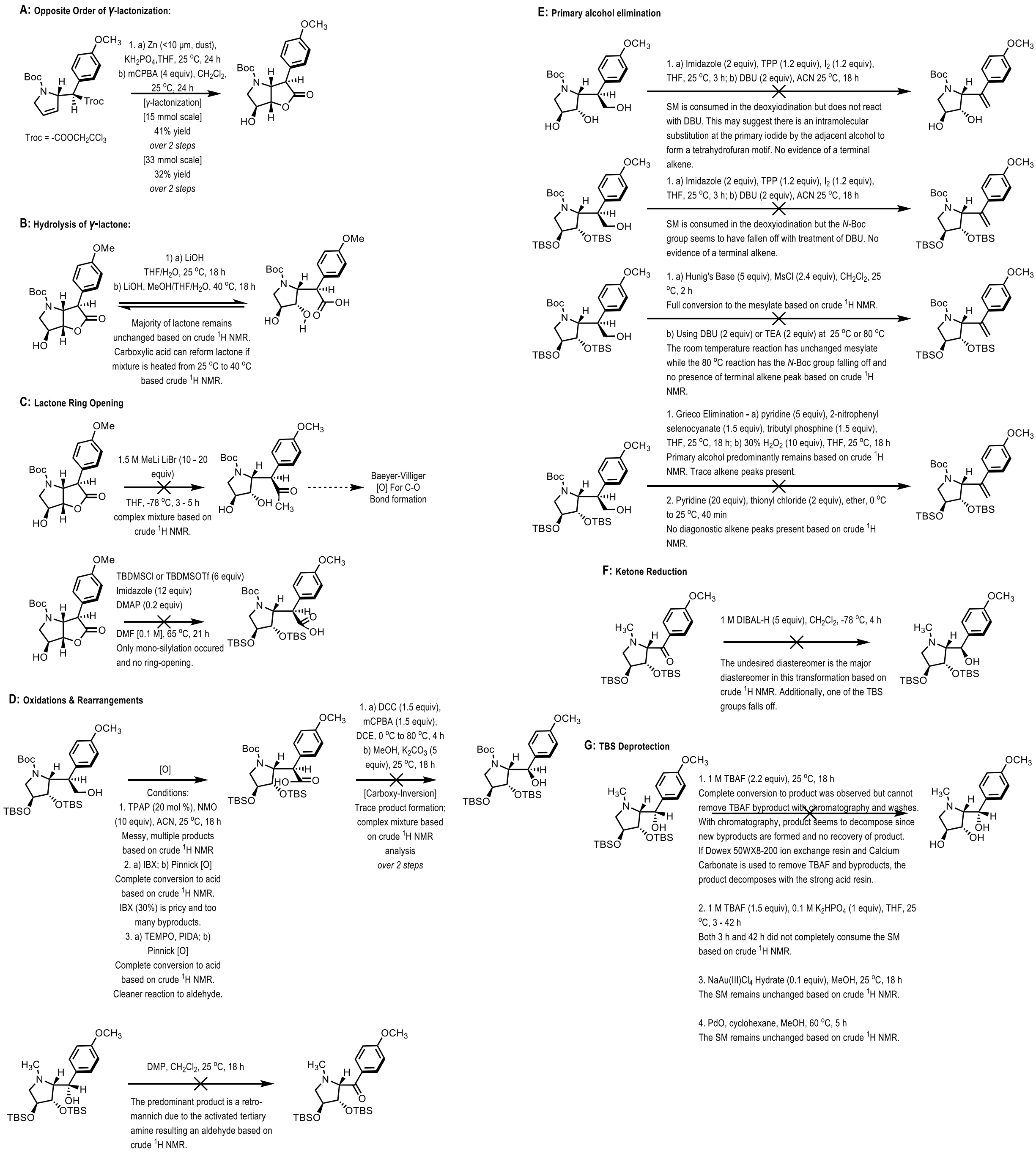

Figure S11. Reactions used to probe the synthetic route to access (–)-6-*epi*-Dragocin D • HF and (–)-Dragocin D • HF.

9. Comparison of NMR data between Natural (–)-Dragocin D and Synthetic (–)-Dragocin D • HF

Numbering pattern used in this work:

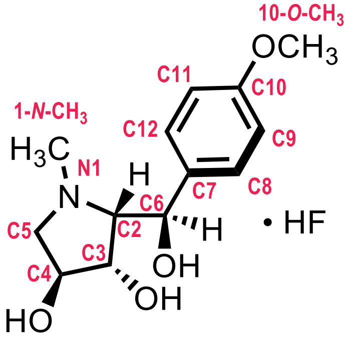

(–)-Dragocin D • HF

i.e. C2 substitution vs. C5 substitution in manuscript

Numbering pattern used in Gerwick *et al.* 2019:

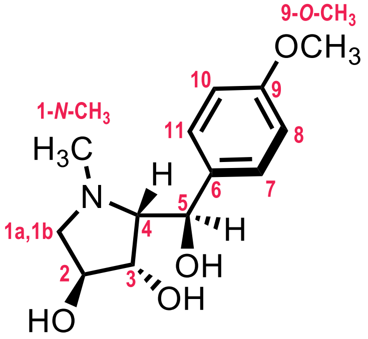

(–)-Dragocin D

Position	[a]Natural (–)-Dragocin D (500 MHz ^{1}H NMR) δ_H (*J* in Hz)	Synthetic (–)-Dragocin D • HF (400 MHz ^{1}H NMR) δ_H (*J* in Hz)	[a]Natural (–)-Dragocin D (125 MHz ^{13}C NMR) δ_C, (type)	Synthetic (–)-Dragocin D • HF (151 MHz ^{13}C NMR) δ_C, (type)
**1a[b] (C5)**	4.12, dd (12.5, 4.3)	3.94, dd (12.1, 4.5)	65.1, CH_2	64.7, CH_2
**1b[b] (C5)**	3.10, d (12.5)	2.87, d (12.1)	65.1, CH_2	64.7, CH_2
**2[b] (C4)**	4.16, brd (4.3)	4.11, brd (4.5)	74.7, CH	75.2, CH
**3[b] (C3)**	4.23, m	4.18, brm	77.6, CH	78.2, CH
**4[b] (C2)**	3.78, dd (7.2, 2.9)	3.48, brm	76.4, CH	75.4, CH
**5[b] (C6)**	5.12, d (7.2)	5.05, d (7.0)	69.8, CH	70.7, CH
**6[b] (C7)**			133.5, C	134.2, C
**7[b] (C8)**	7.47, d (8.7)	7.45, d (8.7)	129.3, CH	129.0, CH
**8[b] (C9)**	7.00, d (8.7)	6.98, d (8.7)	115.5, CH	115.2, CH

**9[b] (C10)**			161.8, C	161.3, C
**10[b] (C11)**	7.00, d (8.7)	6.98, d (8.7)	115.5 CH	115.2, CH
**11[b] (C12)**	7.47, d (8.7)	7.45, d (8.7)	129.3, CH	129.0, CH
**1-*N*-CH_3[b] (1-*N*-CH_3)**	2.64, s	2.49, s	43.7, CH_3	43.3, CH_3
**9-*O*-CH_3[b] (10-*O*-CH_3)**	3.81, s	3.81, s	76.0, CH_3	55.8, CH_3

Table S1. NMR data comparison in deuterated methanol (MeOD). [a]Natural (–)-Dragocin D ^{1}H NMR and ^{13}C NMR adapted from Gerwick, W. H. *et al*. **2019**.[36] [b]Numbering pattern adapted from Gerwick, W. H. *et al*. **2019**.[36]

10. Crystal Structure and Experimental

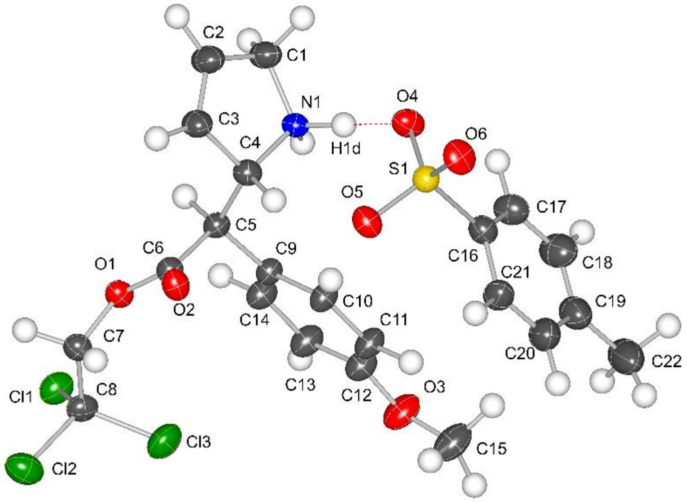

Crystal Data. $C_{22}H_{24}Cl_3NO_6S$, M_r = 536.863, monoclinic, $P2_1$ (No. 4), a = 14.2890(7) Å, b = 5.8147(3) Å, c = 15.3517(8) Å, β = 98.200(5)°, $\alpha = \gamma$ = 90°, V = 1262.47(11) Å^3, T = 173.04(18) K, Z = 2, Z' = 1, μ(Cu K_α) = 4.385, 17627 reflections measured, 4257 unique (R_{int} = 0.0610) which were used in all calculations. The final wR_2 was 0.1819 (all data) and R_1 was 0.0653 (I≥2 σ(I)).

Experimental. Single colorless needle-shaped crystals of **15** were chosen from the sample as supplied. Sample was recrystallized by dissolving the solids in deuterated chloroform in an NMR tube and allowed to slowly evaporate over several days to form crystals. A suitable crystal with dimensions 0.19 × 0.06 × 0.02 mm^3 was selected and mounted on a loop on a XtaLAB AFC11 (RCD3): quarter-chi single diffractometer. The crystal was kept at a steady T = 173.04(18) K during data collection. The structure was solved with ShelXT 2018/2 (Sheldrick, 2018) and by using Olex2 1.5-alpha (Dolomanov et al., 2009) as the graphical interface. The model was refined with olex2.refine 1.5-alpha (Bourhis et al., 2015) using full matrix least squares minimisation on $\boldsymbol{F}^2$.

Compound	15
Formula	$C_{22}H_{24}Cl_3NO_6S$
$D_{calc.}$/ g cm^{-3}	1.412
μ/mm^{-1}	4.385
Formula Weight	536.863
Color	colorless
Shape	needle-shaped
Size/mm^3	0.19×0.06×0.02
T/K	173.04(18)
Crystal System	monoclinic
Flack Parameter	0.010(12)
Hooft Parameter	0.010(12)
Space Group	$P2_1$
a/Å	14.2890(7)
b/Å	5.8147(3)
c/Å	15.3517(8)
α/°	90
β/°	98.200(5)
γ/°	90
V/Å^3	1262.47(11)
Z	2
Z'	1
Wavelength/Å	1.54184
Radiation type	Cu K$_\alpha$
Θ_{min}/°	2.91
Θ_{max}/°	68.25
Measured Refl's.	17627
Indep't Refl's	4257
Refl's I≥2 σ(I)	3521
R_{int}	0.0610
Parameters	442
Restraints	623
Largest Peak	0.9670
Deepest Hole	-0.3524
GooF	1.0562
wR_2 (all data)	0.1819
wR_2	0.1700
R_1 (all data)	0.0790
R_1	0.0653

Structure Quality Indicators

**Reflections:**	d min (CuKα) 2Θ=136.5° 0.83	I/σ(I) 17.1	Rint m=4.00 6.10%	Full 135.4° 96.7	
**Refinement:**	Shift 0.001	Max Peak 1.0	Min Peak -0.4	GooF 1.056	Hooft .010(12)

A colorless needle-shaped-shaped crystal with dimensions 0.19 × 0.06 × 0.02 mm^3 was mounted on a loop. Data were collected using a XtaLAB AFC11 (RCD3): quarter-chi single diffractometer equipped with an Oxford Cryosystems low-temperature device operating at T = 173.04(18) K.

Data were measured using ω scans with Cu K$_\alpha$ radiation. The diffraction pattern was indexed and the total number of runs and images was based on the strategy calculation from the program CrysAlisPro system (CCD

43.92a 64-bit (release 05-10-2023)). The maximum resolution that was achieved was Θ = 68.25° (0.83 Å).

The unit cell was refined using CrysAlisPro 1.171.43.103a (Rigaku OD, 2023) on 5445 reflections, 31% of the observed reflections.

Data reduction, scaling and absorption corrections were performed using CrysAlisPro 1.171.43.103a (Rigaku OD, 2023). The final completeness is 97.98 % out to 68.25° in Θ. A numerical absorption correction based on gaussian integration over a multifaceted crystal model was performed using CrysAlisPro 1.171.42.74a (Rigaku Oxford Diffraction, 2022). An empirical absorption correction using spherical harmonics, implemented in SCALE3 ABSPACK scaling algorithm was also applied. The absorption coefficient μ of this material is 4.385 mm^{-1} at this wavelength (λ = 1.54184Å) and the minimum and maximum transmissions are 0.484 and 1.000.

The structure was solved and the space group $P2_1$ (# 4) determined by the ShelXT 2018/2 (Sheldrick, 2018) structure solution program and refined by full matrix least squares minimisation on $\boldsymbol{F}^2$ using version of olex2.refine 1.5-alpha (Bourhis et al., 2015). Hydrogen atom positions were located from the electron densities and freely refined using Hirshfeld scattering factors. Refinement was by using NoSpherA2, an implementation of non-spherical atom-form-factors (F. Kleemiss, H. Puschmann, O. Dolomanov, S.Grabowsky - https://doi.org/10.1039/D0SC05526C – 2020). NoSpherA2 implementation of HAR makes use of tailor-made aspherical atomic form factors calculated from a Hirshfeld-partitioned electron density (ED) not from spherical-atom form factors. The ED was calculated from a Gaussian basis set single determinant SCF wavefunction from DFT using selected functionals for a fragment of this crystal. This fragment was embedded in an electrostatic crystal field by employing cluster charges. The following options were used: SOFTWARE: ORCA PARTITIONING: NoSpherA2 INT ACCURACY: Normal METHOD: PBE BASIS SET: def2-SVP CHARGE: 0 MULTIPLICITY: 1 SOLVATION: Chloroform DATE: 2024-04-16_17-13-57

There is a single formula unit in the asymmetric unit, which is represented by the reported sum formula. In other words: Z is 2 and Z' is 1. The moiety formula is C15 H17 Cl3 N O3, C7 H7 O3 S.

The Flack parameter was refined to 0.010(12). Determination of absolute structure using Bayesian statistics on Bijvoet differences using the Olex2 results in 0.010(12). The chiral atoms in this structure are: C4(S), C5(R). Note: The Flack parameter is used to determine chirality of the crystal studied, the value should be near 0, a value of 1 means that the stereochemistry is wrong and the model should be inverted. A value of 0.5 means that the crystal consists of a racemic mixture of the two enantiomers.

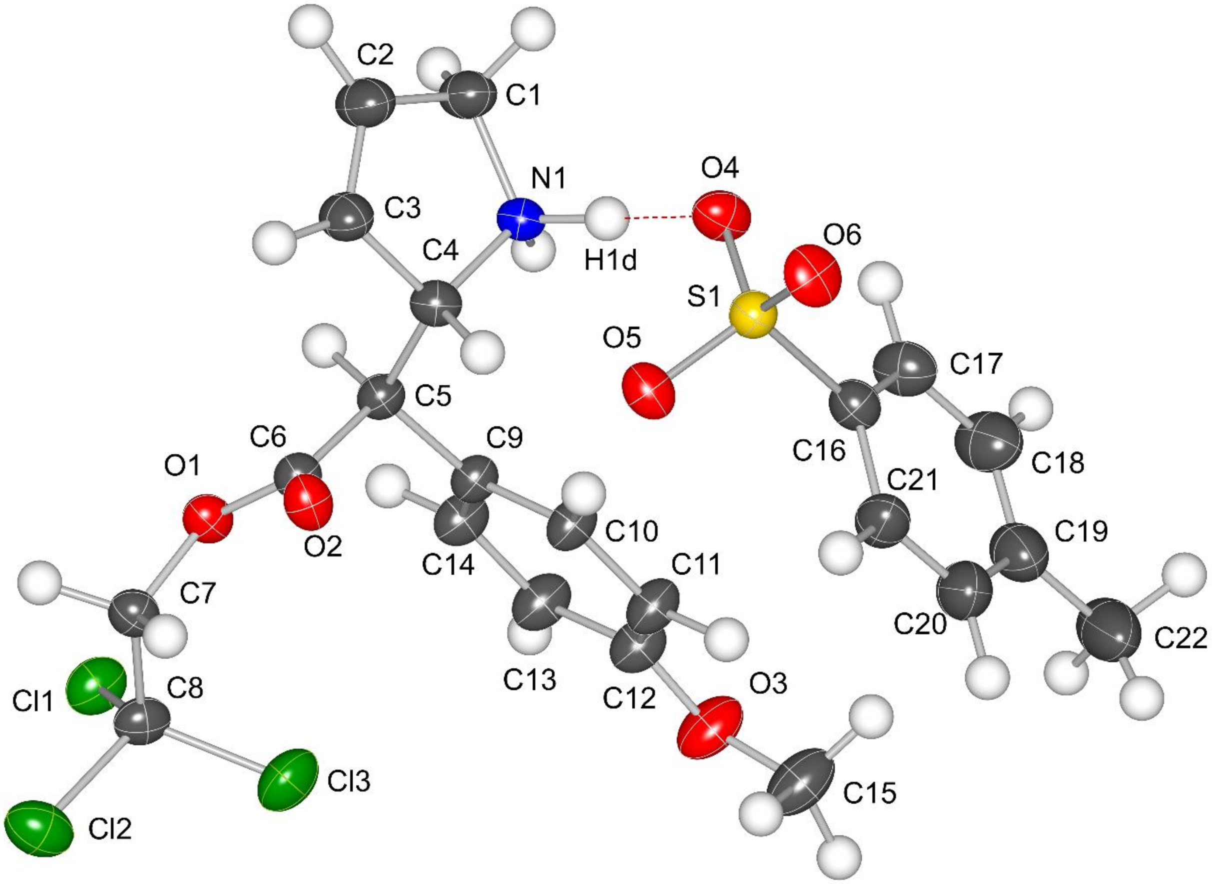

Figure S12. Thermal ellipsoidal (50% probability) representation of the molecular structure in the crystal structure. The chiral atoms in this structure are: C4(S), C5(R).

Data Plots: Diffraction Data

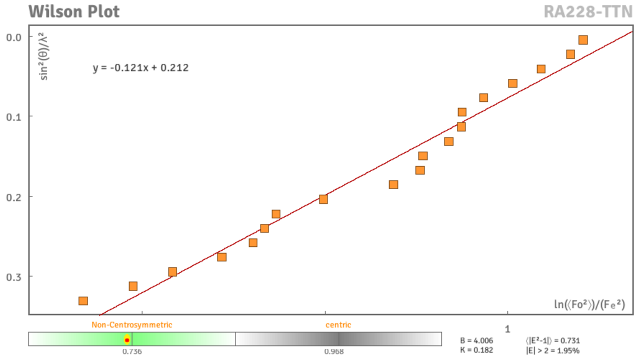

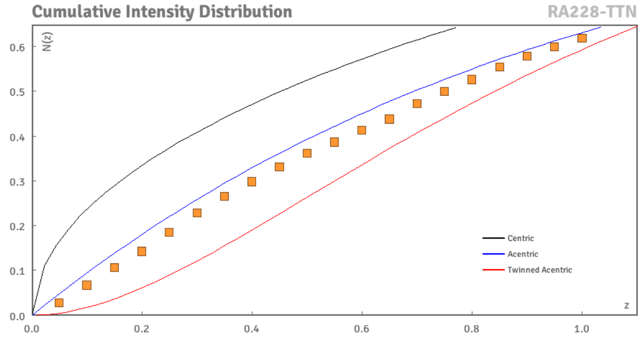

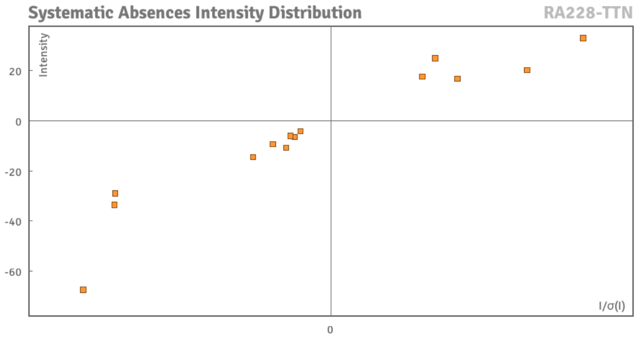

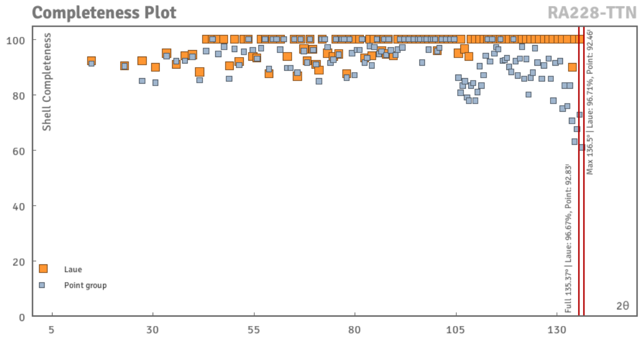

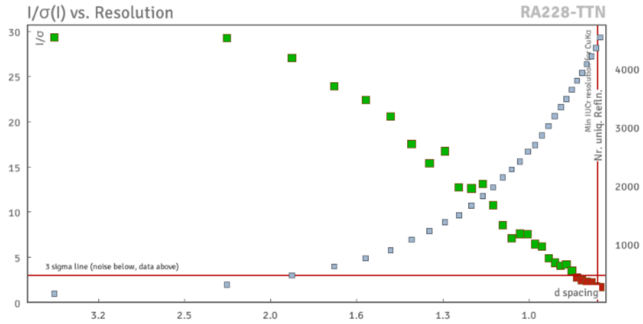

Data Plots: Refinement and Data

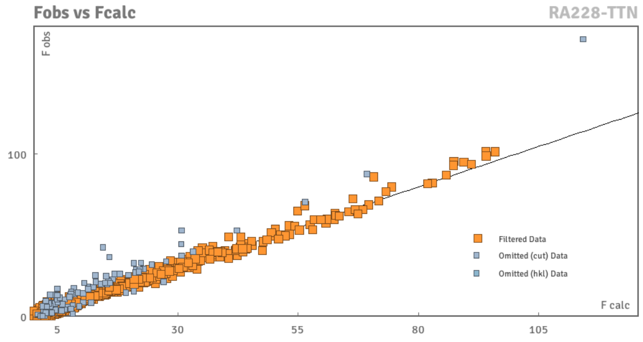

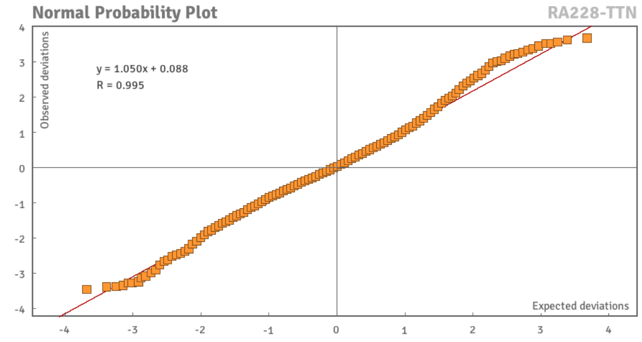

Reflection Statistics

Total reflections (after filtering)	17037	Unique reflections	4257
Completeness	0.925	Mean I/σ	11.57
hkl_{max} collected	(17, 7, 17)	hkl_{min} collected	(-15, -6, -18)
hkl_{max} used	(17, 6, 18)	hkl_{min} used	(-17, -6, 0)
Lim d_{max} collected	20.0	Lim d_{min} collected	0.83
d_{max} used	15.19	d_{min} used	0.83
Friedel pairs	2294	Friedel pairs merged	0
Inconsistent equivalents	0	R_{int}	0.0606
R_{sigma}	0.0584	Intensity transformed	0
Omitted reflections	573	Omitted by user (OMIT hkl)	0
Multiplicity	(2290, 1323, 773, 635, 490, 347, 248, 112, 63, 7, 3, 1)	Maximum multiplicity	13
Removed systematic absences	14	Filtered off (Shel/OMIT)	31

Table S2: Fractional Atomic Coordinates (×10^4) and Equivalent Isotropic Displacement Parameters ($Å^2$×10^3) for **15.** U_{eq} is defined as 1/3 of the trace of the orthogonalised U_{ij}.

Atom	x	y	z	U_{eq}
C1	300(4)	6108(12)	891(4)	58.5(14)
C2	-447(5)	7287(13)	1266(4)	63.5(16)
C3	-103(4)	8518(11)	1984(4)	53.1(13)
C4	952(4)	8348(9)	2196(3)	42.3(10)
C5	1302(4)	7158(9)	3070(3)	40.3(10)
C6	1028(4)	8616(9)	3805(3)	39.7(10)
C7	811(4)	8428(10)	5317(3)	44.9(11)
C8	1578(4)	7607(10)	6016(4)	49.4(11)
C9	2370(4)	6907(8)	3245(3)	39.0(10)
C10	2971(4)	8667(9)	3048(4)	43.7(11)
C11	3946(4)	8554(10)	3260(4)	48.6(12)
C12	4344(4)	6607(9)	3686(4)	48.9(13)
C13	3760(4)	4798(10)	3894(4)	53.8(14)
C14	2786(4)	4945(9)	3671(4)	47.7(12)
C15	5879(5)	8208(12)	3823(6)	77(2)
Cl1	1525.9(10)	4565(2)	6157.8(10)	52.6(4)
Cl2	1355.7(13)	8957(2)	7013.0(10)	62.7(5)
Cl3	2698.6(11)	8352(2)	5779.5(11)	62.9(5)
N1	1201(3)	6973(8)	1423(3)	44.1(10)
O3	5282(3)	6342(7)	3943(4)	68.0(12)
O1	905(3)	7249(6)	4505(2)	44.2(8)
O2	950(3)	10665(6)	3804(3)	47.4(9)
C16	3625(4)	11682(9)	1015(4)	48.8(13)
C17	4005(5)	9736(12)	712(4)	61.6(15)
C18	4979(5)	9364(15)	873(5)	79.4(19)
C19	5578(5)	10820(14)	1369(5)	74.3(17)
C20	5172(5)	12813(14)	1696(5)	73.4(18)
C21	4222(5)	13265(12)	1545(4)	60.7(15)
C22	6616(6)	10412(17)	1532(6)	102(3)
O5	2116(3)	12851(6)	1652(2)	49.1(10)
O6	2289(3)	14221(6)	209(3)	56.7(11)
O4	1959(3)	10217(6)	427(3)	54.3(11)
S1	2412.2(10)	12287(2)	798.6(9)	45.5(4)

Table S3: Anisotropic Displacement Parameters (×10^4) for **15.** The anisotropic displacement factor exponent takes the form: $-2\pi^2[h^2a^{*2} \times U_{11} + ... + 2hka^* \times b^* \times U_{12}]$

Atom	U_{11}	U_{22}	U_{33}	U_{23}	U_{13}	U_{12}
C1	54(2)	64(3)	55(3)	-1.5(15)	0.7(13)	-10.0(18)
H1a	56(12)	64(4)	59(11)	-2(2)	5(5)	-10(2)
H1b	55(12)	68(11)	56(4)	2(5)	1(2)	-9(2)
C2	54(2)	75(4)	59(3)	2.7(16)	-0.3(13)	-13(2)
H2	53(3)	50(10)	46(8)	0.4(18)	2.5(17)	2(3)
C3	49(2)	60(3)	49(2)	5.1(14)	1.7(12)	-1.1(17)
H3	54(10)	190(60)	120(30)	22(6)	-8(6)	-90(20)
C4	48(2)	34(2)	44.1(18)	2.9(13)	3.1(10)	0.0(12)
H4	46(10)	34(4)	45(11)	4(3)	5(4)	-1(2)
C5	42(2)	33(2)	46.5(18)	0.6(12)	5.3(10)	-0.9(11)
H5	41(14)	33(4)	45(11)	1(3)	2(5)	1(2)
C6	44(2)	30.3(16)	45.0(18)	1.9(12)	5.9(11)	0.4(10)
C7	52(2)	36(2)	46.3(18)	-1.3(15)	6.7(11)	-4.4(11)

Atom	U_{11}	U_{22}	U_{33}	U_{23}	U_{13}	U_{12}
H7a	53(4)	40(11)	43(10)	-3(3)	5(3)	-5(4)
H7b	57(11)	36(3)	49(11)	-1.9(19)	6(4)	-3.9(17)
C8	56(2)	35(2)	55(2)	-5.3(11)	0.1(11)	-1.2(11)
C9	39.6(19)	27(2)	51(2)	1.7(10)	6.8(11)	0.2(14)
C10	39.4(18)	29(2)	61(3)	1.0(11)	4.7(13)	6.1(16)
H10	37(10)	46(12)	130(50)	-3(3)	-1(5)	41(12)
C11	39.1(19)	33(2)	73(3)	2.6(11)	4.9(13)	6.4(16)
H11	45(6)	37(5)	48(8)	-3(2)	4(2)	-2(2)
C12	44.4(19)	31(2)	70(3)	4.2(11)	2.4(13)	3.1(15)
C13	48(2)	32(2)	79(4)	2.9(12)	2.1(14)	6.4(18)
H13	51(8)	27(8)	50(20)	0(3)	5(5)	0(6)
C14	48(2)	29(2)	64(3)	1.6(12)	2.9(14)	5.3(16)
H14	51(8)	27(8)	50(20)	0(3)	5(5)	0(6)
C15	50(3)	56(3)	120(6)	1.8(17)	0(2)	8(3)
H15a	42(12)	59(12)	119(6)	5(5)	-1(3)	8(3)
H15b	45(11)	53(6)	112(12)	1(3)	-3(5)	10(4)
H15c	50(4)	59(12)	123(11)	3(3)	-2(3)	8(5)
Cl1	53.6(8)	35.8(7)	67.0(9)	-1.8(6)	3.5(6)	4.2(6)
Cl2	85.0(11)	48.6(10)	52.6(8)	-3.3(7)	3.2(7)	-3.9(6)
Cl3	49.9(8)	53.5(9)	84.4(11)	-11.6(7)	6.7(7)	0.7(8)
N1	49(2)	39(2)	43.2(18)	3.8(14)	2.5(11)	-0.9(14)
H1c	51(8)	39(6)	40(10)	4(3)	2(3)	-3(3)
H1d	47(9)	42(8)	44(8)	5(3)	1(4)	1(3)
O3	44.2(18)	50(2)	106(3)	6.8(12)	-3.2(14)	6(2)
O1	55(2)	31.3(16)	46.3(15)	-2.9(13)	8.0(10)	-1.8(10)
O2	62(2)	30.8(15)	52(2)	2.6(12)	17.4(17)	2.1(11)
C16	56(3)	42(3)	48(3)	2.5(15)	9.7(16)	-1.8(16)
C17	68(3)	52(3)	64(3)	12.4(16)	3.8(17)	-10.5(19)
H17	68(9)	68(13)	130(40)	20(4)	-7(5)	-44(11)
C18	67(3)	91(4)	78(4)	19.2(16)	2.8(17)	-9(2)
H18	76(8)	85(6)	80(10)	15(2)	15(3)	-4(3)
C19	66(3)	97(4)	61(3)	8.5(17)	11.2(17)	-4(2)
C20	60(2)	93(4)	69(3)	0.2(17)	13.6(17)	-8(2)
H20	63(9)	120(20)	120(50)	0(5)	9(6)	-43(15)
C21	60(2)	60(3)	63(3)	-5.0(17)	12.9(17)	-15(2)
H21	68(9)	68(13)	130(40)	20(4)	-7(5)	-44(11)
C22	67(3)	164(9)	73(5)	19(2)	8.9(19)	-10(4)
H22a	75(12)	167(15)	76(7)	19(5)	13(4)	-11(5)
H22b	70(11)	166(10)	76(12)	15(4)	12(4)	-12(5)
H22c	66(13)	164(11)	73(9)	23(4)	13(4)	-10(5)
O5	67(2)	33(2)	48(2)	9.7(18)	14.4(17)	4.5(16)
O6	80(3)	29(2)	62(2)	6.4(19)	14(2)	17.1(18)
O4	71(3)	38(2)	51(2)	-8(2)	2.9(19)	-1.8(17)
S1	56.5(8)	31.4(7)	48.5(7)	2.2(6)	7.2(6)	5.3(6)

Table S4: Bond Lengths in Å for **15**.

Atom	Atom	Length/Å
C1	H1a	1.0900
C1	H1b	1.0900
C1	C2	1.455(9)
C1	N1	1.510(7)
C2	H2	1.0883(19)
C2	C3	1.347(9)
C3	H3	1.0881(19)

Atom	Atom	Length/Å
C3	C4	1.499(8)
C4	H4	1.0988(19)
C4	C5	1.529(7)
C4	N1	1.515(7)
C5	H5	1.101(9)
C5	C6	1.507(7)
C5	C9	1.519(7)

Atom	Atom	Length/Å
C6	O1	1.368(6)
C6	O2	1.197(6)
C7	H7a	1.0979(13)
C7	H7b	1.0979(13)
C7	C8	1.499(8)
C7	O1	1.445(6)
C8	Cl1	1.785(6)
C8	Cl2	1.788(6)
C8	Cl3	1.747(6)
C9	C10	1.396(7)
C9	C14	1.405(7)
C10	H10	1.0780
C10	C11	1.386(7)
C11	H11	1.0780
C11	C12	1.388(8)
C12	C13	1.407(8)
C12	O3	1.352(7)
C13	H13	1.0780
C13	C14	1.387(8)
C14	H14	1.0780
C15	H15a	1.0899
C15	H15b	1.0899

Atom	Atom	Length/Å
C15	H15c	1.0899
C15	O3	1.408(8)
N1	H1c	1.068(9)
N1	H1d	1.068(9)
C16	C17	1.365(8)
C16	C21	1.427(8)
C16	S1	1.753(6)
C17	H17	1.0780
C17	C18	1.395(10)
C18	H18	1.0780
C18	C19	1.358(11)
C19	C20	1.420(11)
C19	C22	1.486(11)
C20	H20	1.0780
C20	C21	1.370(10)
C21	H21	1.0780
C22	H22a	1.0899
C22	H22b	1.0899
C22	H22c	1.0899
O5	S1	1.470(4)
O6	S1	1.439(4)
O4	S1	1.445(4)

Table S5: Bond Angles in ° for **15**.

Atom	Atom	Atom	Angle/°
H1b	C1	H1a	108.9
C2	C1	H1a	110.9(4)
C2	C1	H1b	110.9(4)
N1	C1	H1a	110.9(3)
N1	C1	H1b	110.9(3)
N1	C1	C2	104.2(5)
H2	C2	C1	124.1(3)
C3	C2	C1	111.9(6)
C3	C2	H2	124.1(4)
H3	C3	C2	123.8(4)
C4	C3	C2	112.3(5)
C4	C3	H3	123.8(3)
H4	C4	C3	110(3)
C5	C4	C3	114.4(4)
C5	C4	H4	107(3)
N1	C4	C3	101.9(4)
N1	C4	H4	112(3)
N1	C4	C5	111.4(4)
H5	C5	C4	109.6(3)
C6	C5	C4	108.4(4)
C6	C5	H5	109.6(3)
C9	C5	C4	113.2(4)
C9	C5	H5	109.6(3)
C9	C5	C6	106.4(4)
O1	C6	C5	109.7(4)
O2	C6	C5	126.4(5)
O2	C6	O1	123.8(5)
H7b	C7	H7a	108.4
C8	C7	H7a	110.0(3)

Atom	Atom	Atom	Angle/°
C8	C7	H7b	110.0(3)
O1	C7	H7a	110.0(3)
O1	C7	H7b	110.0(3)
O1	C7	C8	108.6(4)
Cl1	C8	C7	111.4(4)
Cl2	C8	C7	105.8(4)
Cl2	C8	Cl1	108.4(3)
Cl3	C8	C7	111.7(4)
Cl3	C8	Cl1	109.0(3)
Cl3	C8	Cl2	110.4(3)
C10	C9	C5	121.7(5)
C14	C9	C5	120.6(5)
C14	C9	C10	117.6(5)
H10	C10	C9	118.6(3)
C11	C10	C9	122.9(5)
C11	C10	H10	118.6(3)
H11	C11	C10	120.7(3)
C12	C11	C10	118.7(5)
C12	C11	H11	120.7(3)
C13	C12	C11	120.0(5)
O3	C12	C11	123.6(5)
O3	C12	C13	116.3(5)
H13	C13	C12	119.9(3)
C14	C13	C12	120.3(5)
C14	C13	H13	119.9(3)
C13	C14	C9	120.5(5)
H14	C14	C9	119.7(3)
H14	C14	C13	119.7(3)
H15b	C15	H15a	109.5

Atom	Atom	Atom	Angle/°
H15c	C15	H15a	109.5
H15c	C15	H15b	109.5
O3	C15	H15a	109.5
O3	C15	H15b	109.5
O3	C15	H15c	109.5
C4	N1	C1	108.8(4)
H1c	N1	C1	109.9(3)
H1c	N1	C4	109.9(3)
H1d	N1	C1	109.9(3)
H1d	N1	C4	109.9(3)
H1d	N1	H1c	108.3
C15	O3	C12	117.9(5)
C7	O1	C6	116.1(4)
C21	C16	C17	119.7(6)
S1	C16	C17	122.1(5)
S1	C16	C21	118.2(4)
H17	C17	C16	119.9(4)
C18	C17	C16	120.1(7)
C18	C17	H17	119.9(4)
H18	C18	C17	118.8(4)
C19	C18	C17	122.5(7)
C19	C18	H18	118.8(5)

Atom	Atom	Atom	Angle/°
C20	C19	C18	116.7(7)
C22	C19	C18	122.0(7)
C22	C19	C20	121.3(7)
H20	C20	C19	118.6(5)
C21	C20	C19	122.9(7)
C21	C20	H20	118.6(4)
C20	C21	C16	118.0(6)
H21	C21	C16	121.0(4)
H21	C21	C20	121.0(4)
H22a	C22	C19	109.5
H22b	C22	C19	109.5
H22b	C22	H22a	109.5
H22c	C22	C19	109.5
H22c	C22	H22a	109.5
H22c	C22	H22b	109.5
O5	S1	C16	106.1(3)
O6	S1	C16	107.8(3)
O6	S1	O5	111.2(2)
O4	S1	C16	106.5(3)
O4	S1	O5	111.6(2)
O4	S1	O6	113.2(2)

Table S6: Torsion Angles in ° for **15.**

Atom	Atom	Atom	Atom	Angle/°
C1	C2	C3	C4	0.3(7)
C1	N1	C4	C3	-8.7(5)
C1	N1	C4	C5	113.7(5)
C2	C3	C4	C5	-115.0(6)
C2	C3	C4	N1	5.3(6)
C3	C4	C5	C6	-65.0(5)
C3	C4	C5	C9	177.2(5)
C4	C5	C6	O1	151.2(4)
C4	C5	C6	O2	-30.5(5)
C4	C5	C9	C10	40.6(5)
C4	C5	C9	C14	-143.9(5)
C5	C6	O1	C7	169.1(4)
C5	C9	C10	C11	175.1(5)
C5	C9	C14	C13	-174.9(5)
C6	O1	C7	C8	-121.5(5)
C9	C10	C11	C12	0.1(7)
C9	C14	C13	C12	-0.7(7)
C10	C11	C12	C13	0.1(7)
C10	C11	C12	O3	-178.2(5)
C11	C12	C13	C14	0.3(7)
C11	C12	O3	C15	4.3(8)
C16	C17	C18	C19	3.6(8)
C16	C21	C20	C19	-1.7(8)
C17	C18	C19	C20	-2.2(9)
C17	C18	C19	C22	-179.7(7)
C18	C19	C20	C21	1.3(8)

Table S7: Hydrogen Fractional Atomic Coordinates (×10[4]) and Equivalent Isotropic Displacement Parameters (Å[2]×10[3]) for **15.** U_{eq} is defined as 1/3 of the trace of the orthogonalised U_{ij}.

Atom	x	y	z	U_{eq}
H1a	237(4)	4249(12)	954(4)	60(6)
H1b	277(4)	6539(12)	197(4)	60(6)
H2	-1193(5)	7186(13)	999(4)	50(6)
H3	-541(4)	9523(11)	2368(4)	120(30)
H4	1270(30)	10080(40)	2220(40)	42(5)
H5	969(5)	5452(17)	3082(3)	40(7)
H7a	116(4)	8066(10)	5511(3)	45(5)
H7b	872(4)	10293(10)	5227(3)	48(5)
H10	2663(4)	10179(9)	2716(4)	70(20)
H11	4386(4)	9950(10)	3097(4)	44(5)
H13	4071(4)	3291(10)	4227(4)	45(9)
H14	2346(4)	3541(9)	3826(4)	45(9)
H15a	5910(20)	8430(40)	3123(6)	75(6)
H15b	5600(16)	9768(18)	4080(20)	71(6)
H15c	6586(9)	7870(30)	4170(20)	79(6)
H1c	1643(5)	5554(14)	1654(3)	44(5)
H1d	1578(5)	8032(12)	1022(4)	45(5)
H17	3550(5)	8483(12)	347(4)	90(17)
H18	5266(5)	7865(15)	594(5)	80(7)
H20	5631(5)	14023(14)	2080(5)	100(20)
H21	3933(5)	14770(12)	1819(4)	90(17)
H22a	6858(8)	9850(90)	925(12)	106(7)
H22b	6976(6)	12000(30)	1760(30)	104(7)
H22c	6773(7)	9080(70)	2030(30)	101(7)

Table S8: Hydrogen Bond information for **15.**

D	H	A	d(D-H)/Å	d(H-A)/Å	d(D-A)/Å	D-H-A/deg
N1	H1c	O5[1]	1.068(9)	1.711(10)	2.729(6)	157.4(2)
N1	H1d	O4	1.068(9)	1.700(11)	2.747(6)	165.2(2)

----

[1]+x,-1+y,+z

References From Section 10 X-ray Determination

CrysAlisPro (ROD), Rigaku Oxford Diffraction, Poland (?).

CrysAlisPro Software System, Rigaku Oxford Diffraction, (2023).

L.J. Bourhis and O.V. Dolomanov and R.J. Gildea and J.A.K. Howard and H. Puschmann, The Anatomy of a Comprehensive Constrained, Restrained, Refinement Program for the Modern Computing Environment - Olex2 Disected, *Acta Cryst. A*, (2015), **A71**, 59-71.

O.V. Dolomanov and L.J. Bourhis and R.J. Gildea and J.A.K. Howard and H. Puschmann, Olex2: A complete structure solution, refinement and analysis program, *J. Appl. Cryst.*, (2009), **42**, 339-341.

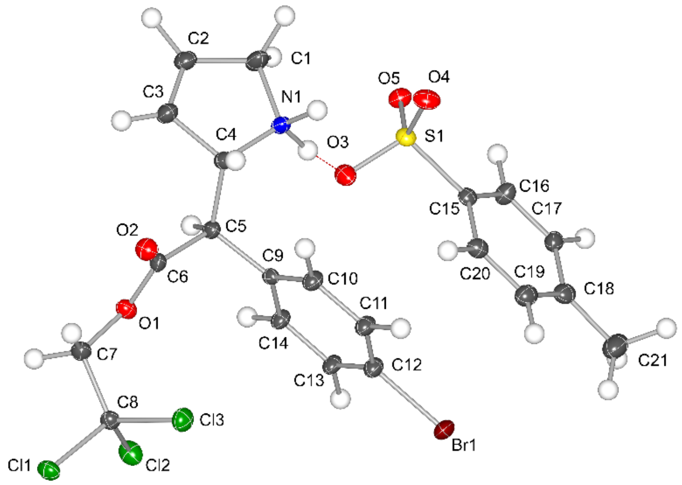

Experimental. Single colorless needle-shaped crystals of **17** were chosen from the sample as supplied. Sample was recrystallized by dissolving the solids in dichloromethane and layering decane on top. The bilayer was allowed to sit slanted over several days to form crystals. A suitable crystal with dimensions 0.40 × 0.03 × 0.02 mm^3 was selected and mounted on a loop with paratone on a XtaLAB Synergy, Dualflex, HyPix diffractometer. The crystal was kept at a steady T = 100(1) K during data collection. The structure was solved with the ShelXT (Sheldrick, 2015) solution program using dual methods and by using Olex2 1.5-alpha (Dolomanov et al., 2009) as the graphical interface. The model was refined with olex2.refine 1.5-alpha (Bourhis et al., 2015) using full matrix least squares minimisation on $\boldsymbol{F}^2$.

Crystal Data. $C_{21}H_{21}BrCl_3NO_5S$, M_r = 585.732, orthorhombic, $P2_12_12$ (No. 18), a = 19.2342(10) Å, b = 21.7904(11) Å, c = 5.7159(3) Å, $\alpha = \beta = \gamma = 90^\circ$, V = 2395.6(2) $Å^3$, T = 100.01(18) K, Z = 4, Z' = 1, μ(Mo K_α) = 2.171, 16011 reflections measured, 5035 unique (R_{int} = 0.0902) which were used in all calculations. The final wR_2 was 0.0698 (all data) and R_1 was 0.0514 (I≥2 σ(I)).

**Compound**	**17**
Formula	$C_{21}H_{21}BrCl_3NO_5S$
$D_{calc.}$/ g cm^{-3}	1.624
μ/mm^{-1}	2.171
Formula Weight	585.732
Color	colorless
Shape	needle-shaped
Size/mm^3	0.40×0.03×0.02
T/K	100.01(18)
Crystal System	orthorhombic
Flack Parameter	-0.018(10)
Hooft Parameter	-0.018(10)
Space Group	$P2_12_12$
a/Å	19.2342(10)
b/Å	21.7904(11)
c/Å	5.7159(3)
α/°	90
β/°	90
γ/°	90
V/Å^3	2395.6(2)
Z	4
Z'	1
Wavelength/Å	0.71073
Radiation type	Mo K$_\alpha$
Θ_{min}/°	2.83
Θ_{max}/°	27.09
Measured Refl's.	16011
Indep't Refl's	5035
Refl's I≥2 σ(I)	3445
R_{int}	0.0902
Parameters	412
Restraints	763
Largest Peak	0.8043
Deepest Hole	-0.7688
GooF	0.9572
wR_2 (all data)	0.0698
wR_2	0.0619
R_1 (all data)	0.0931
R_1	0.0514

Structure Quality Indicators

**Reflections:**	d min (MoKα) 2Θ=54.2° 0.78	I/σ(I) 8.2	Rint m=3.13 9.02%	Full 50.5° 97% to 54.2° 99.3	
**Refinement:**	Shift -0.005	Max Peak 0.8	Min Peak -0.8	GooF 0.957	Hooft -.018(10)

A colorless needle-shaped-shaped crystal with dimensions 0.40 × 0.03 × 0.02 mm^3 was mounted on a loop with paratone. Data were collected using a XtaLAB Synergy, Dualflex, HyPix diffractometer operating at T = 100.01(18) K.

Data were measured using ω scans with Mo K_α radiation. The diffraction pattern was indexed and the total number of runs and images was based on the strategy calculation from the program CrysAlisPro system (CCD 43.92a 64-bit (release 05-10-2023)). The maximum resolution that was achieved was Θ = 27.09° (0.78 Å). The unit cell was refined using CrysAlisPro 1.171.43.123a (Rigaku OD, 2024) on 4191 reflections, 26% of the observed reflections.

Data reduction, scaling and absorption corrections were performed using CrysAlisPro 1.171.43.123a (Rigaku OD, 2024). The final completeness is 99.68 % out to 27.09° in Θ. A numerical absorption correction based on gaussian integration over a multifaceted crystal model was performed using CrysAlisPro 1.171.42.74a (Rigaku Oxford Diffraction, 2022). An empirical absorption correction using spherical harmonics, implemented in SCALE3 ABSPACK scaling algorithm was also applied. The absorption coefficient μ of this material is 2.171 mm^{-1} at this wavelength (λ = 0.71073Å) and the minimum and maximum transmissions are 0.509 and 1.000.

The structure was solved and the space group $P2_12_12$ (# 18) determined by the ShelXT (Sheldrick, 2015) structure solution program using using dual methods and refined by full matrix least squares minimisation on $\boldsymbol{F}^2$ using version of olex2.refine 1.5-alpha (Bourhis et al., 2015). Hydrogen atom positions were located from the electron densities and freely refined using Hirshfeld scattering factors. Refinement was by using NoSpherA2, an implementation of non-spherical atom-form-factors (F. Kleemiss, H. Puschmann, O. Dolomanov, S.Grabowsky - https://doi.org/10.1039/D0SC05526C – 2020). NoSpherA2 implementation of HAR makes use of tailor-made aspherical atomic form factors calculated from a Hirshfeld-partitioned electron density (ED) not from spherical-atom form factors. The ED was calculated from a Gaussian basis set single determinant SCF wavefunction from DFT using selected functionals for a fragment of this crystal. This fragment was embedded in an electrostatic crystal field by employing cluster charges. The following options were used: SOFTWARE: SOFTWARE: ORCA PARTITIONING: NoSpherA2 INT ACCURACY: Normal METHOD: PBE BASIS SET: def2-SVP CHARGE: 0 MULTIPLICITY: 1 SOLVATION: Methanol DATE: 2024-04-04_10-24-50

There is a single formula unit in the asymmetric unit, which is represented by the reported sum formula. In other words: Z is 4 and Z' is 1. The moiety formula is C14 H14 Br Cl3 N O2, C7 H7 O3 S.

The Flack parameter was refined to -0.018(10). Determination of absolute structure using Bayesian statistics on Bijvoet differences using the Olex2 results in -0.018(10). The chiral atoms in this structure are: C4(S), C5(R). Note: The Flack parameter is used to determine chirality of the crystal studied, the value should be near 0, a value of 1 means that the stereochemistry is wrong and the model should be inverted. A value of 0.5 means that the crystal consists of a racemic mixture of the two enantiomers.

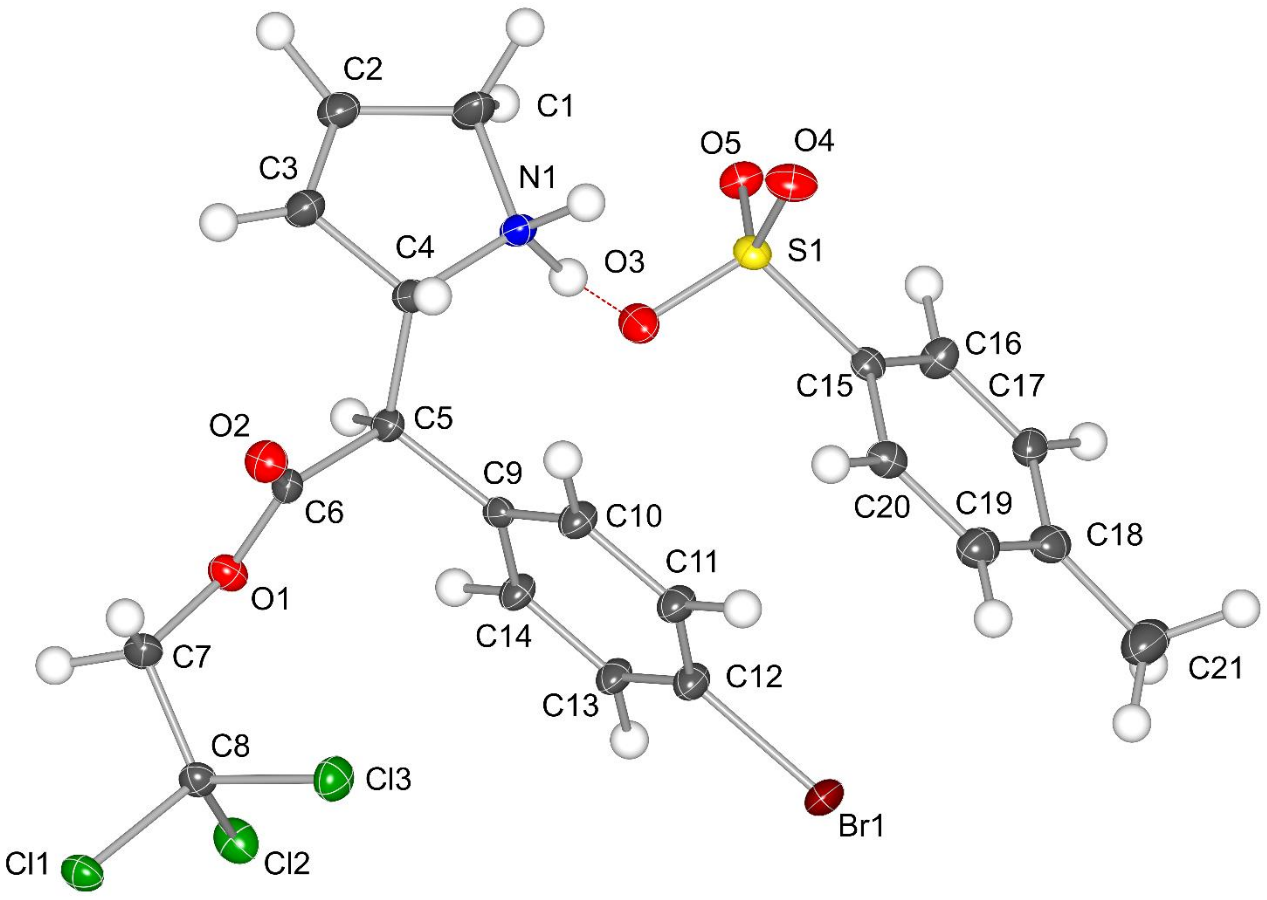

Figure S13. Thermal ellipsoidal (50% probability) representation of the molecular structure in the crystal structure. The chiral atoms in this structure are: C4(S), C5(R).

Data Plots: Diffraction Data

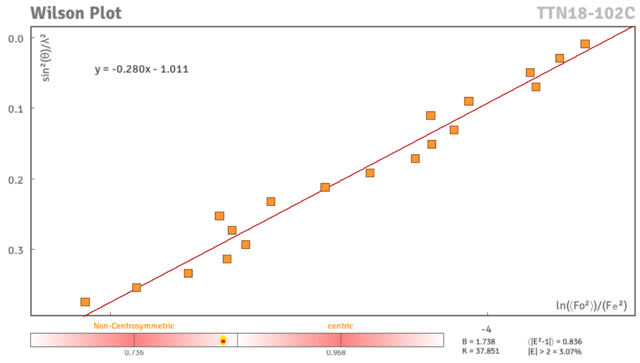

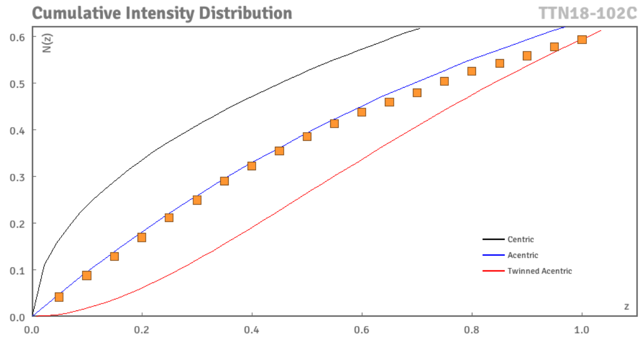

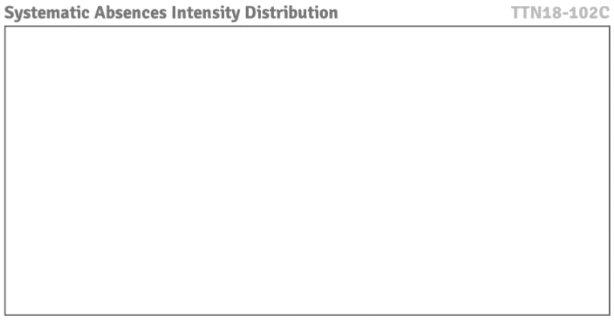

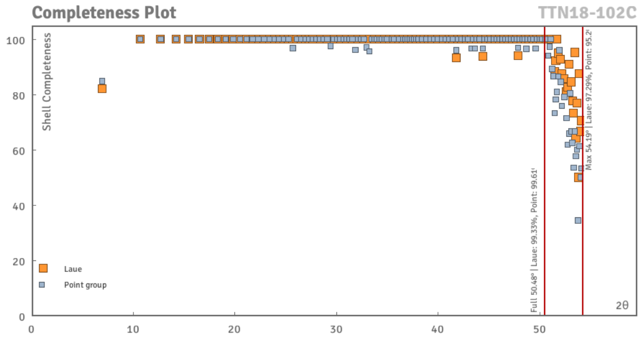

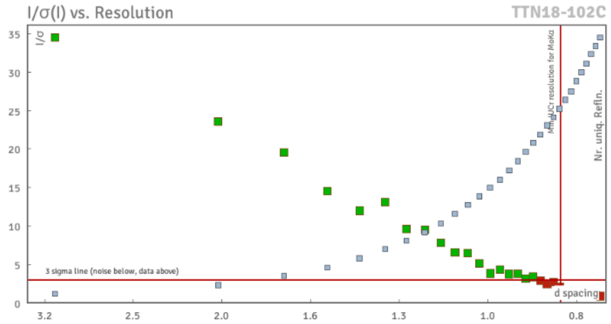

Data Plots: Refinement and Data

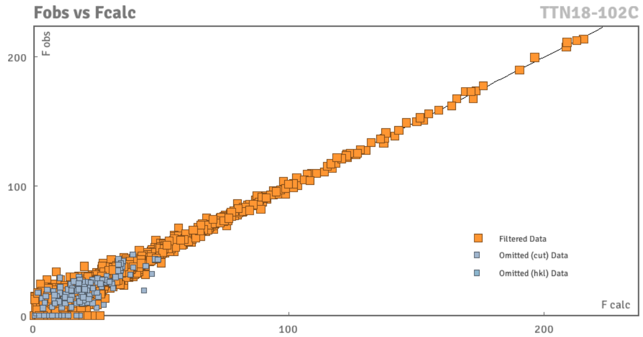

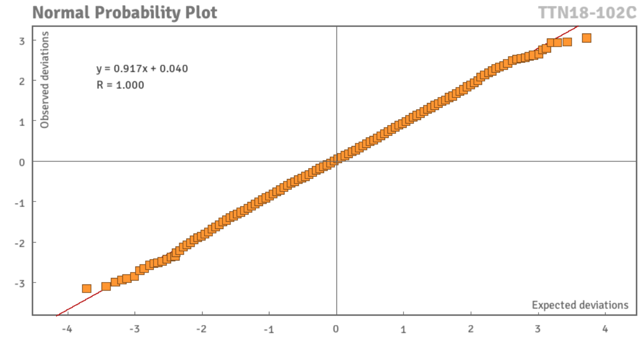

Reflection Statistics

Total reflections (after filtering)	15774	Unique reflections	5035
Completeness	0.952	Mean I/σ	8.04
hkl_{max} collected	(20, 18, 6)	hkl_{min} collected	(-23, -28, -7)
hkl_{max} used	(23, 27, 7)	hkl_{min} used	(-23, 0, 0)
Lim d_{max} collected	20.0	Lim d_{min} collected	0.78
d_{max} used	7.21	d_{min} used	0.78
Friedel pairs	1891	Friedel pairs merged	0
Inconsistent equivalents	0	R_{int}	0.09
R_{sigma}	0.122	Intensity transformed	0
Omitted reflections	34	Omitted by user (OMIT hkl)	0
Multiplicity	(5197, 2899, 1412, 163, 22, 3)	Maximum multiplicity	13
Removed systematic absences	0	Filtered off (Shel/OMIT)	203

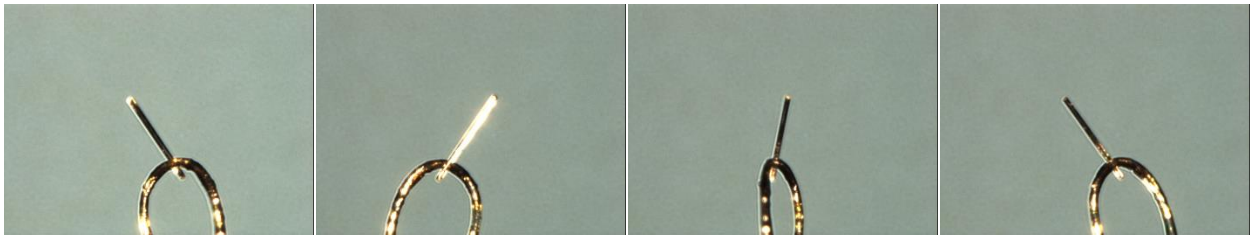

Table S9: Fractional Atomic Coordinates (×10[4]) and Equivalent Isotropic Displacement Parameters (Å[2]×10[3]) for **17.** U_{eq} is defined as 1/3 of the trace of the orthogonalised U_{ij}.

Atom	x	y	z	U_{eq}
Br1	5514.6(2)	4244.61(18)	4805.6(8)	26.71(13)
C1	9520.1(19)	5725.0(16)	5134(7)	27.3(9)
C2	9567(2)	6371.5(17)	5969(7)	22.8(10)
C3	9005(2)	6540(2)	7165(7)	22.7(10)
C4	8465(2)	6039.1(17)	7262(8)	18.7(8)
C5	7773.5(19)	6237.9(16)	6138(8)	17.5(8)
C6	7444(2)	6709.9(18)	7744(8)	16.5(9)
C7	6576(2)	7481.0(18)	7872(8)	21.2(9)
C8	5834(2)	7258.8(17)	7843(7)	18.2(5)
C9	7241.1(18)	5726.9(17)	5808(6)	14.2(8)
C10	7124(2)	5285.9(17)	7526(7)	20.1(10)
C11	6614(2)	4836.1(19)	7211(7)	19.7(10)
C12	6218(2)	4852.2(16)	5186(8)	20.0(9)
C13	6319(2)	5285.9(17)	3441(7)	19.3(9)
C14	6830.6(19)	5724.9(19)	3787(7)	17.9(9)
Cl1	5331.0(5)	7820.5(4)	9288.6(18)	21.5(3)
Cl2	5538.5(6)	7177.9(4)	4903.6(18)	25.2(3)
Cl3	5745.6(5)	6540.8(4)	9251.2(18)	24.4(3)
N1	8814.5(18)	5523.2(15)	5973(6)	18.4(7)
O1	7007.5(14)	7070.7(12)	6553(5)	19.9(7)
O2	7544.2(15)	6741.7(11)	9820(6)	22.8(6)
C15	8030(2)	4010.3(15)	183(7)	15.3(8)
C16	8079(2)	3720.5(17)	-1980(7)	19.5(10)
C17	7553(2)	3322.5(18)	-2680(8)	20.8(10)
C18	6968(2)	3216.8(18)	-1269(8)	22.4(10)
C19	6931(2)	3509.0(17)	906(8)	24.6(10)
C20	7452(2)	3904.7(18)	1629(8)	19.6(10)
C21	6390(2)	2797.4(19)	-2090(8)	33.7(12)
O3	8326.4(15)	5087.5(12)	1752(5)	22.0(7)
O4	9015.1(14)	4234.1(13)	3143(4)	23.4(7)
O5	9142.4(14)	4603.6(11)	-863(4)	20.0(7)
S1	8687.1(6)	4515.6(5)	1139.7(18)	16.7(3)

Table S10: Anisotropic Displacement Parameters (×10[4]) for **17.** The anisotropic displacement factor exponent takes the form: $-2\pi^2[h^2a^{*2} \times U_{11} + ... + 2hka^* \times b^* \times U_{12}]$

Atom	U_{11}	U_{22}	U_{33}	U_{23}	U_{13}	U_{12}
Br1	17.3(2)	21.6(2)	41.3(3)	-5.0(2)	-2.8(3)	-0.3(3)
C1	17.4(13)	26.6(14)	38(2)	-3.8(8)	8.0(10)	-3.8(11)
H1a	25(8)	41(8)	37(3)	-5(3)	8.3(15)	-4.6(15)
H1b	20(5)	27(6)	45(7)	-3(2)	5(2)	-3(3)
C2	18.0(16)	24.7(14)	26(2)	-0.7(8)	-1.4(11)	0.9(11)

Atom	U_{11}	U_{22}	U_{33}	U_{23}	U_{13}	U_{12}
H2	40(8)	39(7)	110(30)	-18(3)	39(7)	-28(6)
C3	17.8(14)	23.2(14)	27(2)	-2.1(8)	-1.0(10)	-0.7(10)
H3	40(8)	39(7)	110(30)	-18(3)	39(7)	-28(6)
C4	15.0(11)	20.5(14)	20.7(17)	0.7(6)	0.2(7)	-2.1(9)
H4	16(5)	22(6)	21(2)	1(2)	0.5(11)	-1.6(13)
C5	15.0(11)	16.6(12)	20.8(15)	0.1(5)	-0.2(7)	-3.2(7)
H5	17(6)	18(7)	21(3)	0(2)	0.5(15)	-2.5(18)
C6	15.6(17)	14.0(15)	19.8(11)	-0.9(8)	1.6(8)	-1.1(7)
C7	17.1(11)	20.3(14)	26(2)	0.9(4)	3.3(5)	-1.2(10)
H7a	17(8)	22(3)	29(7)	0.7(15)	3(3)	0.2(18)
H7b	14(7)	28(7)	26(3)	2(3)	4.1(15)	-1.8(16)
C8	17.0(11)	19.9(6)	17.7(6)	1.1(4)	3.1(4)	-0.1(3)
C9	12.9(14)	14.8(13)	14.9(14)	2.0(8)	0.5(8)	-2.7(8)
C10	20.1(19)	21.2(16)	18.9(15)	-3.8(10)	-3.6(10)	2.2(9)
H10	20(11)	25(11)	20(5)	-7(5)	-4(4)	4(4)
C11	18.7(18)	20.0(17)	20.4(15)	-2.5(9)	-3.1(10)	1.5(9)
H11	65(15)	65(14)	39(7)	-46(7)	-34(5)	29(5)
C12	19.2(18)	20.0(16)	20.6(15)	-2.6(10)	-3.4(10)	1.5(9)
C13	19.0(18)	19.2(16)	19.7(15)	-3.1(9)	-4.0(9)	0.7(9)
H13	65(15)	65(14)	39(7)	-46(7)	-34(5)	29(5)
C14	18.0(17)	18.5(16)	17.3(14)	-2.3(9)	-3.0(9)	0.2(9)
H14	20(11)	25(11)	20(5)	-7(5)	-4(4)	4(4)
Cl1	18.8(6)	25.2(5)	20.4(6)	4.5(4)	0.7(5)	-3.9(4)
Cl2	29.2(6)	27.2(5)	19.2(5)	5.5(5)	-1.5(5)	-2.3(4)
Cl3	26.5(6)	22.1(5)	24.7(6)	-1.9(4)	-0.5(5)	3.7(4)
N1	14.2(13)	19.4(13)	21.7(17)	-0.8(6)	1.7(10)	-1.9(9)
H1c	14(5)	21(5)	22(3)	-2(2)	2.6(16)	-1.7(16)
H1d	11(6)	20(3)	23(4)	-1.3(15)	1.6(19)	-1.4(16)
O1	18.1(13)	19.6(12)	21.9(14)	2.4(7)	2.4(7)	1.4(8)
O2	23.6(16)	24.7(14)	20.1(11)	1.4(12)	0.2(8)	-2.1(7)
C15	16.3(15)	15.4(16)	14.1(14)	2.6(8)	-2.7(8)	2.2(9)
C16	20.6(18)	22(2)	16.2(15)	-3.4(10)	0.7(9)	-1.4(10)
H16	47(11)	90(30)	37(10)	-45(9)	24(5)	-38(8)
C17	19.9(16)	18(2)	24.3(18)	-0.9(10)	-3.5(9)	-0.3(10)
H17	39(8)	61(17)	41(8)	-25(6)	13(4)	-20(6)
C18	19.5(16)	22(2)	25.5(16)	0.9(10)	-3.4(9)	3.4(10)
C19	21.9(17)	25(2)	26.5(17)	-1.0(10)	-2.3(9)	1.6(10)
H19	39(8)	61(17)	41(8)	-25(6)	13(4)	-20(6)
C20	18.7(15)	22(2)	18.1(17)	1.5(9)	0.3(9)	2.6(10)
H20	41(13)	80(30)	37(9)	-35(9)	21(5)	-31(7)
C21	28(2)	30(2)	43(3)	-5.7(12)	-11.0(14)	3.7(15)
H21a	31(8)	38(8)	43(3)	-7(3)	-11.5(18)	4.4(19)
H21b	31(8)	30(3)	47(8)	-5.2(19)	-12(3)	4(2)
H21c	29(4)	31(8)	45(7)	-5(2)	-9(2)	5(3)
O3	21.6(17)	23.7(15)	20.7(16)	1.6(10)	-5.9(13)	-6.1(11)
O4	20.8(16)	32.0(16)	17.5(14)	4.0(13)	-2.8(10)	2.4(11)
O5	18.9(15)	26.7(16)	14.5(14)	-1.8(12)	0.2(10)	-0.4(11)
S1	16.6(6)	19.4(6)	14.1(5)	1.9(5)	-2.1(5)	-0.5(5)

Table S11: Bond Lengths in Å for **17**.

Atom	Atom	Length/Å
Br1	C12	1.905(4)
C1	H1a	1.13(2)
C1	H1b	1.13(2)

Atom	Atom	Length/Å
C1	C2	1.490(5)
C1	N1	1.505(4)
C2	H2	1.12(3)

Atom	Atom	Length/Å
C2	C3	1.330(5)
C3	H3	1.01(4)
C3	C4	1.507(5)
C4	H4	1.12(3)
C4	C5	1.540(5)
C4	N1	1.503(5)
C5	H5	1.14(3)
C5	C6	1.517(5)
C5	C9	1.525(5)
C6	O1	1.336(5)
C6	O2	1.204(5)
C7	H7a	1.08(2)
C7	H7b	1.08(2)
C7	C8	1.508(5)
C7	O1	1.434(4)
C8	Cl1	1.765(4)
C8	Cl2	1.782(4)
C8	Cl3	1.768(4)
C9	C10	1.392(5)
C9	C14	1.399(5)
C10	H10	1.080(4)
C10	C11	1.399(5)
C11	H11	1.118(4)
C11	C12	1.386(5)

Atom	Atom	Length/Å
C12	C13	1.388(5)
C13	H13	1.118(4)
C13	C14	1.387(5)
C14	H14	1.081(4)
N1	H1c	1.10(4)
N1	H1d	1.09(4)
C15	C16	1.391(5)
C15	C20	1.405(5)
C15	S1	1.763(4)
C16	H16	1.14(4)
C16	C17	1.391(5)
C17	H17	1.11(4)
C17	C18	1.404(5)
C18	C19	1.398(6)
C18	C21	1.513(5)
C19	H19	1.09(4)
C19	C20	1.385(5)
C20	H20	1.05(4)
C21	H21a	1.091(18)
C21	H21b	1.091(18)
C21	H21c	1.091(18)
O3	S1	1.468(3)
O4	S1	1.444(3)
O5	S1	1.454(3)

Table S12: Bond Angles in ° for **17**.

Atom	Atom	Atom	Angle/°
H1b	C1	H1a	109.1
C2	C1	H1a	111.1(2)
C2	C1	H1b	111.1(2)
N1	C1	H1a	111.1(2)
N1	C1	H1b	111.1(2)
N1	C1	C2	103.2(3)
H2	C2	C1	124.0(2)
C3	C2	C1	112.1(4)
C3	C2	H2	124.0(3)
H3	C3	C2	123.9(3)
C4	C3	C2	112.3(4)
C4	C3	H3	123.9(2)
H4	C4	C3	109.7(2)
C5	C4	C3	112.1(3)
C5	C4	H4	109.7(2)
N1	C4	C3	102.5(3)
N1	C4	H4	109.7(2)
N1	C4	C5	113.1(3)
H5	C5	C4	109.1(2)
C6	C5	C4	107.4(3)
C6	C5	H5	109.1(2)
C9	C5	C4	115.2(3)
C9	C5	H5	109.1(2)
C9	C5	C6	106.8(3)
O1	C6	C5	110.7(4)
O2	C6	C5	124.6(4)
O2	C6	O1	124.7(4)

Atom	Atom	Atom	Angle/°
H7b	C7	H7a	108.2
C8	C7	H7a	109.7(2)
C8	C7	H7b	109.7(2)
O1	C7	H7a	109.7(2)
O1	C7	H7b	109.7(2)
O1	C7	C8	110.0(3)
Cl1	C8	C7	106.9(3)
Cl2	C8	C7	110.1(3)
Cl2	C8	Cl1	109.6(2)
Cl3	C8	C7	111.7(3)
Cl3	C8	Cl1	110.4(2)
Cl3	C8	Cl2	108.1(2)
C10	C9	C5	121.7(4)
C14	C9	C5	118.9(4)
C14	C9	C10	119.3(4)
H10	C10	C9	119.8(2)
C11	C10	C9	120.4(4)
C11	C10	H10	119.8(3)
H11	C11	C10	120.8(3)
C12	C11	C10	118.4(4)
C12	C11	H11	120.8(2)
C11	C12	Br1	117.9(3)
C13	C12	Br1	119.4(3)
C13	C12	C11	122.7(4)
H13	C13	C12	121.1(2)
C14	C13	C12	117.8(4)
C14	C13	H13	121.1(2)

Atom	Atom	Atom	Angle/°
C13	C14	C9	121.4(4)
H14	C14	C9	119.3(2)
H14	C14	C13	119.3(2)
C4	N1	C1	109.9(3)
H1c	N1	C1	103(2)
H1c	N1	C4	118(2)
H1d	N1	C1	106(2)
H1d	N1	C4	110(2)
H1d	N1	H1c	109(3)
C7	O1	C6	117.5(3)
C20	C15	C16	120.1(4)
S1	C15	C16	120.7(3)
S1	C15	C20	119.1(3)
H16	C16	C15	120.3(3)
C17	C16	C15	119.3(4)
C17	C16	H16	120.3(3)
H17	C17	C16	119.3(3)
C18	C17	C16	121.3(4)
C18	C17	H17	119.3(3)
C19	C18	C17	118.5(4)

Atom	Atom	Atom	Angle/°
C21	C18	C17	120.6(4)
C21	C18	C19	120.9(4)
H19	C19	C18	119.6(3)
C20	C19	C18	120.8(4)
C20	C19	H19	119.6(3)
C19	C20	C15	119.9(4)
H20	C20	C15	120.0(2)
H20	C20	C19	120.0(3)
H21a	C21	C18	109.5
H21b	C21	C18	109.5
H21b	C21	H21a	109.5
H21c	C21	C18	109.5
H21c	C21	H21a	109.5
H21c	C21	H21b	109.5
O3	S1	C15	105.39(17)
O4	S1	C15	107.09(18)
O4	S1	O3	112.22(16)
O5	S1	C15	105.64(17)
O5	S1	O3	111.10(16)
O5	S1	O4	114.65(15)

Table S13: Torsion Angles in ° for **17**.

Atom	Atom	Atom	Atom	Angle/°
Br1	C12	C11	C10	-179.0(3)
Br1	C12	C13	C14	179.4(3)
C1	C2	C3	C4	-2.2(4)
C1	N1	C4	C3	-1.0(4)
C1	N1	C4	C5	119.8(3)
C2	C3	C4	C5	-119.5(4)
C2	C3	C4	N1	2.0(4)
C3	C4	C5	C6	-70.6(4)
C3	C4	C5	C9	170.5(3)
C4	C5	C6	O1	155.9(3)
C4	C5	C6	O2	-26.3(4)
C4	C5	C9	C10	41.9(4)
C4	C5	C9	C14	-142.1(3)
C5	C6	O1	C7	171.0(3)
C5	C9	C10	C11	177.6(4)
C5	C9	C14	C13	-177.3(3)
C6	O1	C7	C8	-107.7(4)
C9	C10	C11	C12	-1.8(4)
C9	C14	C13	C12	1.1(4)
C10	C11	C12	C13	1.6(5)
C11	C12	C13	C14	-1.3(5)
C15	C16	C17	C18	1.3(4)
C15	C20	C19	C18	-0.8(4)
C16	C17	C18	C19	-1.7(4)
C16	C17	C18	C21	178.1(4)
C17	C18	C19	C20	1.5(4)

Table S14: Hydrogen Fractional Atomic Coordinates (×10^4) and Equivalent Isotropic Displacement Parameters

(Å²×10³) for **17**. U_{eq} is defined as 1/3 of the trace of the orthogonalised U_{ij}.

Atom	x	y	z	U_{eq}
H1a	9557(2)	5700.5(17)	3170(40)	35(4)
H1b	9944(9)	5434(6)	5923(16)	31(4)
H2	10024(15)	6681(10)	5629(13)	63(12)
H3	8947(3)	6956(16)	7920(30)	63(12)
H4	8374(3)	5901(4)	9130(60)	20(3)
H5	7885(4)	6460(7)	4370(50)	19(3)
H7a	6604(2)	7934(10)	7130(17)	23(4)
H7b	6760(4)	7504.8(19)	9650(40)	23(4)
H10	7430(2)	5291.0(17)	9112(9)	22(7)
H11	6528(2)	4472(2)	8559(9)	56(10)
H13	5998(2)	5281.4(17)	1809(9)	56(10)
H14	6913.9(19)	6073(2)	2469(8)	22(7)
H1c	8560(20)	5353(16)	4360(80)	19(3)
H1d	8910(20)	5139(18)	7160(70)	18(3)
H16	8543(16)	3808(3)	-3160(40)	58(15)
H17	7597(3)	3084(9)	-4390(60)	47(9)
H19	6489(16)	3424(3)	2040(40)	47(9)
H20	7414(3)	4125(8)	3250(60)	54(15)
H21a	6322(3)	2849(2)	-3970(30)	37(4)
H21b	6525(3)	2323(8)	-1689(10)	36(4)
H21c	5908(8)	2919(3)	-1199(16)	35(4)

Table S15: Hydrogen Bond information for **17**.

D	H	A	d(D-H)/Å	d(H-A)/Å	d(D-A)/Å	D-H-A/deg
N1	H1c	O3	1.10(4)	1.66(4)	2.758(4)	170(3)
N1	H1d	O5[1]	1.09(4)	1.69(4)	2.772(4)	172(4)

––––

[1]+x,+y,1+z

References From Section 10 X-ray Determination

CrysAlisPro (ROD), Rigaku Oxford Diffraction, Poland (?).

CrysAlisPro Software System, Rigaku Oxford Diffraction, (2024).

L.J. Bourhis and O.V. Dolomanov and R.J. Gildea and J.A.K. Howard and H. Puschmann, The Anatomy of a Comprehensive Constrained, Restrained, Refinement Program for the Modern Computing Environment - Olex2 Disected, *Acta Cryst. A*, (2015), **A71**, 59-71.

O.V. Dolomanov and L.J. Bourhis and R.J. Gildea and J.A.K. Howard and H. Puschmann, Olex2: A complete structure solution, refinement and analysis program, *J. Appl. Cryst.*, (2009), **42**, 339-341.

Sheldrick, G.M., ShelXT-Integrated space-group and crystal-structure determination, *Acta Cryst.*, (2015), **A71**, 3-8.

11. DFT Calculations of C–H Functionalization & Cyclopropanation

All calculations were performed using Gaussian-16 suite of programs.[1] Geometry and vibrational frequencies of the presented $Rh_2(OAc)_4$-carbene complexes were calculated at the B3LYP-D3(BJ)[2-7] level of theory in conjunction with LANL2DZ[8,9] basis set for rhodium and 6-31G(d,p)[10,11] basis set for other atoms. The solvent effect (CH_2Cl_2 was chosen as a solvent) was incorporated in all presented calculations by using the CPCM method.[12,13] Frequency analyses and Gibbs free energy and zero-point energy corrections were calculated at a temperature and pressure corresponding to standard reaction conditions (i.e. at the 298.15K and 1 atm, respectively) and are included in the Cartesian coordinates section. Structures of additional calculated complexes and their important geometry parameters are given in Figure S14.

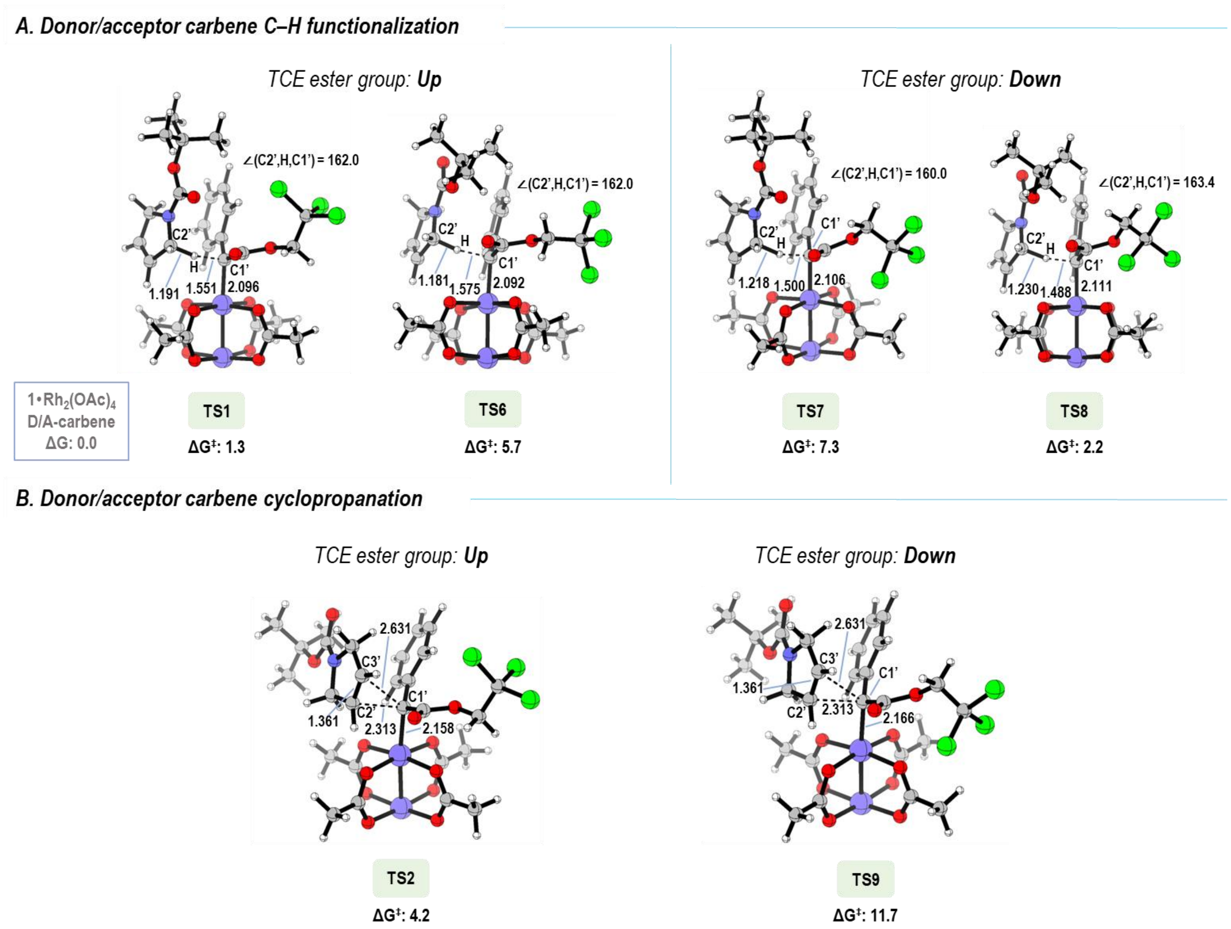

Figure S14. Transition state complexes of D/A-carbenes with various substrate *N*-Boc-2,5-dihydro-1H-pyrrole (**1**) and TCE-ester functionality orientations for C–H functionalization and cyclopropanation reactions. B3LYP-D3(BJ)/6-31G(d,p)/LANL2DZ/CPCM(DCM)

Coordinates

N-Boc-2,5-dihydro-1H-pyrrole, 1

B3LYP-D3(BJ) SCF energy in solution (au): -557.027119
B3LYP-D3(BJ) enthalpy in solution (au): -557.013407
B3LYP-D3(BJ) free energy in solution (au): -557.066274

Symbol	X	Y	Z
C	0.2127280	4.6863900	-3.1272800
C	-1.0803050	5.1681620	-3.7267430
C	-1.2593930	4.6926630	-4.9595850
C	-0.1152400	3.8137340	-5.3885620
H	0.8971390	5.5076530	-2.8734560
H	0.0656810	4.1007210	-2.2093770
H	-1.7558180	5.8219720	-3.1879910
H	-2.1067220	4.8891640	-5.6058940
H	0.4009610	4.1941630	-6.2803490
H	-0.4309970	2.7868120	-5.6169270

N	0.7532870	3.8570480	-4.2068640
C	1.9374790	3.2137250	-4.0722600
O	2.6324160	3.2764760	-3.0606640
O	2.2216140	2.5131300	-5.1966460
C	3.4574840	1.7271310	-5.3063760
C	3.4731970	0.6253520	-4.2452270
H	4.3359740	-0.0274490	-4.4088820
H	3.5348930	1.0511800	-3.2440870
H	2.5658620	0.0180640	-4.3190600
C	3.3467750	1.1268680	-6.7076230
H	3.2907990	1.9175560	-7.4611820
H	4.2222610	0.5066300	-6.9184260
H	2.4513500	0.5041980	-6.7893200
C	4.6740280	2.6492860	-5.2069770
H	5.5845380	2.0763480	-5.4070870
H	4.6026370	3.4488610	-5.9506650
H	4.7452700	3.0931530	-4.2143010

$Rh_2(OAc)_4$ D/A-carbene

B3LYP-D3(BJ) SCF energy in solution (au): -3049.176992
B3LYP-D3(BJ) enthalpy in solution (au): -3049.138675
B3LYP-D3(BJ) free energy in solution (au): -3049.252599

Symbol	X	Y	Z
O	-0.9590010	0.8965170	-0.1354840
O	-1.7879220	1.3407710	-2.8838430
O	0.9049150	0.7802900	-3.8277380
O	1.7831460	0.3171120	-1.0691960
O	-2.3141160	-0.8583030	-3.0907570
O	0.4146450	-1.4280800	-4.0070090
O	1.2466950	-1.8811100	-1.2402870
O	-1.4843400	-1.3029400	-0.3257210
C	-1.4956140	-0.1885120	0.2752980
C	2.0110890	-0.9303820	-0.9017080
C	0.9171560	-0.3474250	-4.4329210
C	-2.5518700	0.3786050	-3.2347570
C	3.3093510	-1.2744150	-0.2133580
H	4.1186990	-0.6628850	-0.6175820
H	3.2164960	-1.0427470	0.8524940
H	3.5358680	-2.3338690	-0.3310610
C	-3.8699340	0.7751260	-3.8541790
H	-3.7265140	1.6145310	-4.5370370
H	-4.3165200	-0.0711960	-4.3760060
H	-4.5477560	1.1019870	-3.0588830
C	-2.2012380	-0.1039190	1.6072190
H	-2.6292070	-1.0683430	1.8788600
H	-1.4905650	0.2171700	2.3738450
H	-2.9908770	0.6505650	1.5506340

Rh	-0.5539470	-1.4473100	-2.1733510
Rh	0.0183720	0.9346050	-1.9631250
C	1.5836600	-0.3584710	-5.7868140
H	2.5486040	0.1502790	-5.7291530
H	1.7136760	-1.3804790	-6.1416580
H	0.9586260	0.1943110	-6.4950120
C	0.5301070	2.8718270	-1.7074470
C	1.7410260	3.5256150	-2.0455110
C	-0.5094880	3.6368440	-0.9940200
C	1.9299950	4.9114750	-1.7615040
C	2.8088300	2.8027350	-2.6507560
O	-1.4747370	4.1463420	-1.5227920
O	-0.2642350	3.6244890	0.3422630
C	3.1214310	5.5390820	-2.0763630
H	1.1294150	5.4772880	-1.2981710
C	4.0019050	3.4405450	-2.9502800
H	2.6714540	1.7528500	-2.8619230
C	-1.3083540	4.0628030	1.2021690
C	4.1580380	4.8031460	-2.6686590
H	3.2565650	6.5934200	-1.8617700
H	4.8154720	2.8854560	-3.4045290
H	5.0943640	5.2969970	-2.9093770
H	-2.2140120	4.2713440	0.6318170
H	-1.4861790	3.2757510	1.9360740
C	-0.8795270	5.3355370	1.9387240
Cl	-0.5954410	6.6747660	0.7729980
Cl	-2.2121800	5.7831570	3.0585410
Cl	0.6246690	5.0420060	2.8759540

$Rh_2(OAc)_4$ A-carbene

B3LYP-D3(BJ) SCF energy in solution (au): -1439.355500
B3LYP-D3(BJ) enthalpy in solution (au): -1439.324693
B3LYP-D3(BJ) free energy in solution (au): -1439.421650

Symbol	X	Y	Z
O	-1.3881280	0.9582690	-0.2250010
O	-2.0543370	1.2555060	-3.0438430
O	0.7588910	1.0037470	-3.7490650
O	1.4327790	0.5715460	-0.9297220
O	-2.3540580	-0.9833000	-3.2282180
O	0.4695340	-1.2396970	-3.9655390
O	1.1492300	-1.6681560	-1.1471610
O	-1.6492730	-1.2909390	-0.3977550
C	-1.8170870	-0.1770680	0.1812910
C	1.7755280	-0.6461820	-0.7416210
C	0.9036770	-0.1152780	-4.3549880
C	-2.6972110	0.2176510	-3.4295400
C	3.0460710	-0.8527590	0.0450100

H	3.8666030	-0.3170510	-0.4391870
H	2.9212790	-0.4318990	1.0466490
H	3.2822940	-1.9139670	0.1164240
C	-3.9775900	0.4813500	-4.1809980
H	-3.7773250	1.1485440	-5.0230330
H	-4.4103110	-0.4532820	-4.5363900
H	-4.6850200	0.9879690	-3.5182140
C	-2.6186030	-0.1563990	1.4593940
H	-2.6662120	-1.1540520	1.8956950
H	-2.1808740	0.5514810	2.1656060
H	-3.6349750	0.1808170	1.2312060
Rh	-0.6058840	-1.3777900	-2.1939360
Rh	-0.3008130	1.0465790	-1.9788890
C	1.6442800	-0.0605190	-5.6685240
H	2.5171500	0.5892500	-5.5797790
H	1.9432250	-1.0611740	-5.9797970
H	0.9828620	0.3688280	-6.4279380
C	0.0955040	2.9294840	-1.7431370
C	-0.3510640	3.6683920	-0.5634420
O	-1.2992690	4.4237440	-0.7343490
O	0.2448250	3.3818720	0.5796590
C	-0.3587430	3.9301420	1.7914710
H	-0.1727320	5.0071540	1.8096460
H	-1.4364640	3.7618110	1.7374520
H	0.6744630	3.4904230	-2.4828700
C	0.2765360	3.2144440	2.9634820
H	1.3569530	3.3802900	2.9858770
H	-0.1509400	3.5930600	3.8962790
H	0.0878010	2.1389440	2.9055800

TS (D/A-carbene): C–H Functionalization Isomer 1 (TCE: Up), TS1
B3LYP-D3(BJ) SCF energy in solution (au): -3606.226525
B3LYP-D3(BJ) enthalpy in solution (au): -3606.174732
B3LYP-D3(BJ) free energy in solution (au): -3606.316744
Imaginary frequency (cm^{-1}): -149.81

Symbol	X	Y	Z
O	3.7934990	-0.8839750	-0.0994100
O	2.4243470	-0.5650360	-2.6938090
O	4.7890720	-1.5206960	-4.0734980
O	6.1942170	-1.7837480	-1.5161090
O	1.6693930	-2.6995960	-2.5261400
O	4.0592400	-3.6625420	-3.9206950
O	5.4598080	-3.9278000	-1.3600820
O	3.0510260	-3.0221510	0.0476730
C	3.2810810	-1.8711110	0.5257790
C	6.3492230	-3.0278480	-1.2731450
C	4.5730270	-2.6879170	-4.5446220

C	1.5221800	-1.4654490	-2.7763730
C	7.7255910	-3.4456770	-0.8121130
H	8.4900580	-2.8869970	-1.3550950
H	7.8272280	-3.2072710	0.2518180
H	7.8659870	-4.5182130	-0.9476490
C	0.1485300	-0.9900220	-3.1850970
H	-0.3639060	-0.5960760	-2.3011680
H	0.2303760	-0.1820450	-3.9141620
H	-0.4369470	-1.8154580	-3.5906870
C	2.9477910	-1.6284880	1.9786710
H	2.3072580	-2.4213620	2.3645260
H	3.8776000	-1.6059290	2.5562690
H	2.4615610	-0.6571030	2.0908580
Rh	3.5316610	-3.4087650	-1.9314670
Rh	4.3328550	-1.0880780	-2.0942810
C	5.0009790	-2.9092330	-5.9756550
H	4.7871300	-2.0206030	-6.5725670
H	6.0832290	-3.0753860	-5.9970000
H	4.5023360	-3.7827240	-6.3958080
C	5.0114300	0.8943980	-2.1284660
C	6.2290150	1.3895770	-2.7216290
C	4.4319490	1.6127720	-0.9646380
C	6.7068470	2.6975510	-2.4466100
C	6.9156820	0.6077800	-3.6837080
O	3.3157700	2.0687240	-0.8607010
O	5.3408420	1.5754400	0.0637420
C	7.8223270	3.1965690	-3.1023280
H	6.1892540	3.3149320	-1.7222290
C	8.0343600	1.1133000	-4.3322180
H	6.5459860	-0.3825460	-3.9068400
C	4.8757420	1.9647790	1.3444510
C	8.4837260	2.4087340	-4.0520670
H	8.1782540	4.1978680	-2.8859810
H	8.5545630	0.5065910	-5.0662730
H	3.8017750	2.1524010	1.3225570
H	5.1136680	1.1680640	2.0509060
C	5.5938770	3.2431930	1.7901970
Cl	7.3736540	2.9852210	1.8276700
Cl	5.2230890	4.5981240	0.6699470
Cl	5.0095190	3.6462840	3.4429940
H	9.3530180	2.8037880	-4.5686630
C	3.5726440	2.0218440	-4.1277470
C	3.6770610	1.0822470	-5.2678370
C	4.5845370	1.5229650	-6.1503650
C	5.1354790	2.8570890	-5.7451350
H	2.5972250	2.3104300	-3.7343490
H	4.0237620	1.4290270	-3.1987580

H	3.1440860	0.1436880	-5.2849780
H	4.8965910	1.0192480	-7.0568580
H	4.9197340	3.6477030	-6.4737070
H	6.2215810	2.8469660	-5.5886720
N	4.4116520	3.1087270	-4.4924190
C	4.3881610	4.3125250	-3.8245460
O	3.6854280	4.5185060	-2.8477030
C	5.3631360	6.5622440	-3.9296340
C	5.8213970	6.5850810	-2.4713370
C	6.4425100	7.1284480	-4.8493710
C	4.0305570	7.2831150	-4.1306270
H	5.0548560	6.1745750	-1.8156900
H	6.7420950	6.0084020	-2.3489920
H	6.0263200	7.6178100	-2.1750370
H	6.1262800	7.0717560	-5.8945940
H	6.6323090	8.1754120	-4.5989240
H	7.3746960	6.5680400	-4.7360410
H	4.1482760	8.3420220	-3.8830210
H	3.7131910	7.2081860	-5.1748430
H	3.2566870	6.8576740	-3.4910750
O	5.2408150	5.1703670	-4.4067400

TS (D/A-carbene): Cyclopropanation (TCE: Up), TS2
B3LYP-D3(BJ) SCF energy in solution (au): -3606.223115
B3LYP-D3(BJ) enthalpy in solution (au): -3606.171701
B3LYP-D3(BJ) free energy in solution (au): -3606.312169
Imaginary frequency (cm^{-1}): -138.18

Symbol	X	Y	Z
O	-1.2646350	1.0510200	-0.2975570
O	-2.1636940	1.0847990	-3.0696100
O	0.5720920	0.6116060	-3.9926820
O	1.4961890	0.6226030	-1.2021000
O	-2.4953230	-1.1559260	-2.9782530
O	0.2526360	-1.6280160	-3.8868610
O	1.1735010	-1.6222060	-1.1116720
O	-1.5802400	-1.1916510	-0.2035230
C	-1.6785470	-0.0169240	0.2627920
C	1.8439000	-0.5664280	-0.8974610
C	0.6725220	-0.5771230	-4.4524540
C	-2.8459900	0.0243410	-3.2773090
C	3.1718540	-0.7065450	-0.1932120
H	3.8947780	-0.0071430	-0.6171280
H	3.0385410	-0.4528230	0.8635710
H	3.5389540	-1.7303380	-0.2667260
C	-4.1985300	0.2207360	-3.9169790
H	-4.1219610	0.9345690	-4.7398600
H	-4.5969460	-0.7292240	-4.2726250

H	-4.8826090	0.6415550	-3.1730830
C	-2.3156560	0.1468940	1.6217080
H	-2.9411270	-0.7145040	1.8565330
H	-1.5240240	0.2203840	2.3749840
H	-2.9029660	1.0660560	1.6550960
Rh	-0.6728030	-1.4674550	-2.0397620
Rh	-0.3133630	0.9638430	-2.1456120
C	1.3793060	-0.7126550	-5.7782670
H	1.2049290	-1.6978460	-6.2101130
H	1.0431060	0.0698860	-6.4612810
H	2.4539740	-0.5765310	-5.6185970
C	0.1191490	3.0764160	-2.0520590
C	1.4664550	3.5869330	-2.2106330
C	-0.7372650	3.6074090	-0.9583870
C	1.8890990	4.7926670	-1.5927830
C	2.4118440	2.8648580	-2.9754270
O	-1.8999540	3.9515720	-1.0094930
O	-0.0330990	3.5134300	0.2128890
C	3.2011850	5.2322120	-1.7066760
H	1.1835050	5.3725580	-1.0098540
C	3.7204260	3.3165360	-3.0943670
H	2.1066110	1.9469200	-3.4561150
C	-0.7574140	3.6787430	1.4187780
C	4.1202580	4.4955110	-2.4605440
H	3.5069530	6.1511290	-1.2176560
H	4.4354510	2.7451030	-3.6756950
H	5.1445610	4.8421920	-2.5548700
C	0.2216910	5.6803690	-4.2585420
C	-0.8833940	5.0240690	-3.5086000
C	-1.0767040	3.7403830	-3.9168160
C	-0.1989930	3.4312020	-5.0983340
H	-0.1199810	6.5930170	-4.7684370
H	1.0521000	5.9775120	-3.6006300
H	-1.4461380	5.5240900	-2.7339470
H	-1.8934880	3.0967700	-3.6227000
H	-0.8100910	3.2903820	-6.0013560
H	0.3989930	2.5304270	-4.9620000
N	0.6360100	4.6350220	-5.1899080
H	-1.8259080	3.7602590	1.2194740
H	-0.5481520	2.8219390	2.0601840
C	1.8111870	4.7326220	-5.8619810
O	2.5314630	5.7255190	-5.8169360
O	2.0444340	3.6026900	-6.5675380
C	3.1731730	3.5171630	-7.5061130
C	2.9949920	2.1276190	-8.1158580
H	2.0244530	2.0466990	-8.6135140
H	3.7808340	1.9394010	-8.8523840

H	3.0547730	1.3588390	-7.3410480
C	4.4979640	3.6081710	-6.7484840
H	5.3277390	3.4650360	-7.4472820
H	4.6033780	4.5779870	-6.2633950
H	4.5515170	2.8227980	-5.9898910
C	3.0395500	4.6006640	-8.5775730
H	3.8082900	4.4543230	-9.3420440
H	2.0597870	4.5346770	-9.0604300
H	3.1563440	5.5939810	-8.1446330
C	-0.2831060	4.9496690	2.1317680
Cl	-0.6203810	6.4020510	1.1243390
Cl	-1.1827310	5.0700330	3.6833580
Cl	1.4818660	4.8644740	2.4614980

TS (A-carbene), TS3

B3LYP-D3(BJ) SCF energy in solution (au): -1996.405732

B3LYP-D3(BJ) enthalpy in solution (au): -1996.362057

B3LYP-D3(BJ) free energy in solution (au): -1996.484307

Imaginary frequency (cm^{-1}): -76.67

Symbol	X	Y	Z
O	3.6313480	-0.9634380	-0.0236400
O	2.5337830	-0.4358650	-2.6428340
O	5.1242960	0.6362430	-3.5336390
O	6.2598360	0.0376030	-0.9238740
O	3.1378760	-2.4907200	-3.3870120
O	5.7396960	-1.4411590	-4.2153860
O	6.8319710	-2.0609760	-1.5715060
O	4.1991890	-3.0490340	-0.7196400
C	3.6983730	-2.2339020	0.1103770
C	7.0629900	-0.9546620	-1.0007030
C	5.6487110	-0.1858360	-4.3613520
C	2.3188000	-1.5329060	-3.2648120
C	8.3991650	-0.7703880	-0.3236980
H	9.0789120	-1.5782790	-0.5934160
H	8.8262550	0.1953560	-0.6026990
H	8.2503640	-0.7674580	0.7605640
C	0.9581480	-1.6677110	-3.8998860
H	0.8701940	-2.6207730	-4.4203620
H	0.1888690	-1.5925690	-3.1263780
H	0.8067790	-0.8389810	-4.5956280
C	3.1385400	-2.7909210	1.3967310
H	3.0682600	-3.8769950	1.3435980
H	3.7989170	-2.5104930	2.2230100
H	2.1572710	-2.3547700	1.5944760
Rh	5.0022100	-2.3392180	-2.4974140
Rh	4.3591120	-0.0901210	-1.7521730
C	6.2265430	0.4038210	-5.6248520

H	5.9289740	-0.2022050	-6.4833780
H	5.9028850	1.4352790	-5.7499230
H	7.3190720	0.3738130	-5.5595680
C	3.9184100	1.7066610	-1.1025410
C	3.0334960	1.9249160	0.0587070
O	1.8223800	2.0335320	-0.0270460
O	3.7494820	1.9573790	1.1873330
C	3.0046560	2.0338360	2.4402290
H	3.7093090	2.4878120	3.1378640
H	2.1546260	2.7027530	2.2955290
C	2.2313520	2.7970870	-3.2457600
C	1.1435620	2.1100850	-4.0090430
C	1.5004630	1.9070360	-5.2796360
C	2.8848550	2.4266250	-5.5519200
H	1.9290690	3.7060200	-2.7150130
H	2.6409740	2.1085170	-2.4600710
H	0.2132770	1.8135450	-3.5422640
H	0.9051820	1.4247180	-6.0456620
H	2.9229700	3.1546720	-6.3710190
H	3.5851130	1.6183240	-5.7977490
N	3.2231950	3.0563640	-4.2725460
C	4.3863840	3.6870490	-3.9791950
O	4.6372940	4.1437570	-2.8630750
C	6.5004280	4.4289160	-4.9803210
C	7.3931450	3.7310040	-3.9524140
C	7.0534300	4.2514570	-6.3936110
C	6.2950250	5.9111560	-4.6649960
H	7.0131080	3.8748970	-2.9417910
H	7.4466370	2.6590430	-4.1578300
H	8.4042570	4.1442370	-4.0151360
H	6.3725360	4.6871950	-7.1299510
H	8.0216700	4.7523380	-6.4761640
H	7.1926980	3.1934630	-6.6302970
H	7.2563500	6.4304890	-4.7203870
H	5.6191840	6.3634550	-5.3969930
H	5.8795820	6.0441730	-3.6664270
O	5.1901660	3.7533770	-5.0585440
C	2.5667690	0.6526490	2.8902050
H	3.4289400	-0.0087910	3.0035170
H	2.0553170	0.7290560	3.8548380
H	1.8813570	0.2103230	2.1645810
H	4.4619700	2.5856340	-1.4651600

TS (A-carbene), TS4

B3LYP-D3(BJ) SCF energy in solution (au): -1996.437629
B3LYP-D3(BJ) enthalpy in solution (au): -1996.393745
B3LYP-D3(BJ) free energy in solution (au): -1996.516397

Imaginary frequency (cm^{-1}): -1018.79

Symbol	X	Y	Z
O	0.1826610	-1.2032140	2.3123660
O	-1.6106700	-0.7459540	0.0289310
O	0.6894470	0.1850860	-1.5574290
O	2.4213410	-0.1980570	0.7589770
O	-1.2076800	-2.8708230	-0.6538590
O	1.0614500	-1.9295740	-2.2712010
O	2.8410300	-2.2944090	-0.0001440
O	0.5877930	-3.3120150	1.5866110
C	0.3253670	-2.4540450	2.4896260
C	3.1983590	-1.1629200	0.4566060
C	0.9529970	-0.6781300	-2.4562440
C	-1.9743720	-1.8890090	-0.4031160
C	4.6760150	-0.9152150	0.6331270
H	5.2314280	-1.8524490	0.6048950
H	5.0210090	-0.2693540	-0.1811200
H	4.8579100	-0.3911670	1.5734860
C	-3.4523410	-2.0699650	-0.6483090
H	-3.7461620	-1.4696920	-1.5153280
H	-3.6881820	-3.1163150	-0.8401600
H	-4.0140460	-1.7057870	0.2150050
C	0.1922790	-2.9536680	3.9075020
H	1.1153260	-2.7252080	4.4502750
H	-0.6277320	-2.4359400	4.4087180
H	0.0306450	-4.0315880	3.9222350
Rh	0.8325380	-2.6601270	-0.3521700
Rh	0.3899960	-0.4025490	0.4186820
C	1.1828110	-0.1621620	-3.8554520
H	0.7014520	-0.8203810	-4.5813500
H	0.8100630	0.8571260	-3.9569840
H	2.2584210	-0.1688670	-4.0603250
C	0.0172950	1.8926540	1.5486090
C	-1.2384890	2.0308660	2.2824920
O	-2.1201540	2.8436180	2.0168240
O	-1.3833730	1.1010980	3.2669830
C	-2.7044860	0.9509610	3.8195490
H	-2.5466950	0.5131460	4.8079390
H	-3.1652400	1.9351360	3.9320280
C	-2.2948320	4.2694380	-0.9788810
C	-3.3214550	3.3515150	-0.7910590
C	-2.7464880	2.0793280	-0.7873530
C	-1.3331900	2.1975570	-0.9439160
H	-2.3102750	5.3488380	-0.9998040
H	-4.3606410	3.6018990	-0.6365550
H	-3.2462000	1.1311980	-0.6557940
H	-0.7601520	1.5497210	-1.6050120

H	-0.7202390	1.8196740	0.1327610
N	-1.1200490	3.6032320	-1.1156260
C	0.1253250	4.2454220	-1.3090680
O	0.2122160	5.4521240	-1.4083280
C	2.5168560	3.7105550	-1.4646730
C	2.9077570	4.5344880	-0.2398480
C	3.2201620	2.3568330	-1.4683220
C	2.7382450	4.4552200	-2.7792750
H	2.3763080	5.4863810	-0.2144260
H	2.6928890	3.9772230	0.6765000
H	3.9821170	4.7359460	-0.2725240
H	2.9099370	1.7654850	-2.3321470
H	4.3009500	2.5156870	-1.5217030
H	2.9858010	1.7889490	-0.5660300
H	3.8106870	4.6179020	-2.9193420
H	2.3716370	3.8576290	-3.6190070
H	2.2341010	5.4215920	-2.7807740
O	1.0818070	3.3315980	-1.3496540
C	-3.5545610	0.0426880	2.9418480
H	-3.0666780	-0.9264210	2.8061870
H	-4.5352790	-0.1174130	3.4016360
H	-3.6991880	0.4941770	1.9574900
H	0.7803220	1.3233640	2.0747530
H	0.4120290	2.8388790	1.1852980

TS (A-carbene), TS5

B3LYP-D3(BJ) SCF energy in solution (au): -1996.451209
B3LYP-D3(BJ) enthalpy in solution (au): -1996.407998
B3LYP-D3(BJ) free energy in solution (au): -1996.529028
Imaginary frequency (cm^{-1}): -218.37

Symbol	X	Y	Z
O	-0.0669980	-1.1618640	2.4715550
O	-1.6465860	-0.5837110	0.0612330
O	0.8487790	0.0776620	-1.3829820
O	2.3824490	-0.4778150	1.0398240
O	-1.4379200	-2.7515170	-0.5868220
O	1.0610000	-2.0880130	-2.0314630
O	2.5994920	-2.6467000	0.3956340
O	0.1255220	-3.3297580	1.8184450
C	-0.0952490	-2.4184640	2.6759250
C	3.0484980	-1.5476790	0.8476310
C	1.1039940	-0.8386210	-2.2363150
C	-2.1020100	-1.6892370	-0.3781800
C	4.5262310	-1.4828720	1.1615520
H	4.9233900	-2.4821280	1.3425950
H	5.0498040	-1.0490700	0.3028160
H	4.7028350	-0.8390640	2.0248530

C	-3.5832610	-1.7431040	-0.6786190
H	-3.7340160	-2.0072000	-1.7292720
H	-4.0448300	-2.5287970	-0.0739420
H	-4.0565040	-0.7858900	-0.4649570
C	-0.4633290	-2.8671100	4.0721940
H	0.0420140	-3.8030060	4.3156260
H	-0.2136900	-2.0960910	4.8018860
H	-1.5437710	-3.0438170	4.1106830
Rh	0.5948240	-2.7967230	-0.1294090
Rh	0.3582070	-0.4499820	0.5723300
C	1.5192850	-0.3753980	-3.6141310
H	1.2137060	-1.1016830	-4.3685440
H	1.1010950	0.6075000	-3.8341720
H	2.6114210	-0.2949210	-3.6409550
C	0.2891010	1.6931340	1.1357960
C	-0.9904360	1.9199920	1.7162860
O	-2.0775780	2.2703220	1.1525360
O	-1.0581530	1.6564620	3.0313960
C	-2.3676800	1.5484830	3.6415240
H	-2.1677550	1.6454640	4.7101360
H	-2.9852510	2.3883920	3.3170090
C	-2.3589840	3.0952160	-0.4986520
C	-3.4108050	2.2204810	-1.0171210
C	-2.9243420	1.5422490	-2.0678970
C	-1.5119670	1.9402680	-2.3468500
H	-2.5164000	4.0316360	0.0163010
H	-4.3836950	2.1305850	-0.5565480
H	-3.4406030	0.7870750	-2.6459140
H	-1.3557640	2.3275750	-3.3576100
H	-0.8035490	1.1263400	-2.1626170
N	-1.3152320	3.0165480	-1.3623940
C	-0.2193140	3.8892090	-1.2980890
O	-0.1720520	4.8064470	-0.5018600
C	2.0062150	4.1968280	-2.2825950
C	2.7373710	4.0174120	-0.9535580
C	2.6967300	3.4166870	-3.3967320
C	1.8099390	5.6619830	-2.6643960
H	2.2377390	4.5552100	-0.1477480
H	2.7960140	2.9565350	-0.6941700
H	3.7557990	4.4033310	-1.0528690
H	2.1286940	3.4875380	-4.3283870
H	3.6958020	3.8261380	-3.5666200
H	2.7928750	2.3637380	-3.1215240
H	2.7874130	6.1205070	-2.8387520
H	1.2265490	5.7394110	-3.5864380
H	1.3013760	6.2115450	-1.8722290
O	0.6803210	3.5268370	-2.2041040

C	-3.0175510	0.2132780	3.3127000
H	-2.3974450	-0.6098240	3.6714900
H	-4.0011140	0.1555280	3.7902340
H	-3.1370090	0.1012970	2.2331740
H	1.1095720	1.7625590	1.8442690
H	0.4794540	2.1695970	0.1857680

TS (D/A-carbene): C–H Functionalization Isomer 2 (TCE: Up), TS6

B3LYP-D3(BJ) SCF energy in solution (au): -3606.227554
B3LYP-D3(BJ) enthalpy in solution (au): -3606.176210
B3LYP-D3(BJ) free energy in solution (au): -3606.315395
Imaginary frequency (cm^{-1}): -152.29

Symbol	X	Y	Z
O	3.6918300	-0.7351600	-0.1325050
O	2.3985050	-0.6528320	-2.7797190
O	4.8523030	-1.5085820	-4.0401060
O	6.2012110	-1.5367380	-1.4239110
O	1.7977770	-2.8286830	-2.5355350
O	4.3035520	-3.6932100	-3.7754870
O	5.6062700	-3.7098280	-1.1365150
O	3.0816450	-2.9061520	0.1032750
C	3.2127590	-1.7184780	0.5251370
C	6.4266650	-2.7444370	-1.0764410
C	4.7458000	-2.7149390	-4.4465700
C	1.5694230	-1.6220430	-2.8504690
C	7.8010270	-3.0244860	-0.5168280
H	8.0061590	-4.0949830	-0.5189360
H	8.5574620	-2.4879630	-1.0927780
H	7.8417610	-2.6554560	0.5133220
C	0.1812250	-1.2710330	-3.3300240
H	-0.3913700	-0.8729210	-2.4857160
H	0.2316910	-0.4963120	-4.0971240
H	-0.3275430	-2.1565370	-3.7116810
C	2.7767450	-1.4188640	1.9395170
H	2.2897750	-2.2863350	2.3840770
H	3.6530880	-1.1449790	2.5340130
H	2.0928690	-0.5659610	1.9401280
Rh	3.6770180	-3.3663880	-1.8256850
Rh	4.3182670	-1.0066180	-2.0979500
C	5.2209350	-2.9781710	-5.8553290
H	4.9482620	-2.1439230	-6.5045660
H	6.3132690	-3.0560930	-5.8492880
H	4.8034460	-3.9111800	-6.2339590
C	4.8848250	1.0037390	-2.2139850
C	6.0786940	1.5394090	-2.8140570
C	4.2446350	1.7289760	-1.0876680
C	6.4808920	2.8806480	-2.5847650

C	6.8182680	0.7633760	-3.7416660
O	3.0872820	2.0801620	-0.9941990
O	5.1379410	1.8370430	-0.0635640
C	7.5652440	3.4241570	-3.2552200
H	5.9271240	3.4886680	-1.8814500
C	7.9108370	1.3097460	-4.4009480
H	6.5086830	-0.2541640	-3.9304880
C	4.5669670	2.0984580	1.2110440
C	8.2785050	2.6406870	-4.1708120
H	7.8497480	4.4559360	-3.0806760
H	8.4725260	0.7076890	-5.1077070
H	4.2106800	3.1288890	1.2824270
H	3.7446910	1.4071120	1.3993680
C	5.6627560	1.8820540	2.2509230
Cl	6.3003410	0.2066580	2.1719960
Cl	7.0117890	3.0428230	1.9864830
Cl	4.9294140	2.1719320	3.8688310
H	9.1244820	3.0673520	-4.7007410
C	3.4098230	1.9956690	-4.2773250
C	3.5732200	1.0114310	-5.3751680
C	4.4669720	1.4555260	-6.2692610
C	4.9495870	2.8308290	-5.9209310
H	2.4109300	2.2539780	-3.9230160
H	3.8692960	1.4701720	-3.3244310
H	3.0876290	0.0470740	-5.3529960
H	4.8128930	0.9256820	-7.1480390
H	4.7055270	3.5799750	-6.6843210
H	6.0335720	2.8802250	-5.7555350
N	4.2055660	3.1051990	-4.6879820
C	4.2256540	4.3615440	-4.1279240
O	4.9039770	5.2688140	-4.5881340
C	3.2536570	5.6607370	-2.2842340
C	2.7391950	6.7728770	-3.1989570
C	2.1971820	5.2783070	-1.2506850
C	4.5754530	6.0250080	-1.6081450
H	3.4949170	7.0776580	-3.9217250
H	1.8476600	6.4351320	-3.7357570
H	2.4614770	7.6369440	-2.5882030
H	2.5274030	4.4169160	-0.6675610
H	2.0180740	6.1234220	-0.5802770
H	1.2553290	5.0199180	-1.7430590
H	4.4451310	6.9421330	-1.0263520
H	4.8857180	5.2337040	-0.9190330
H	5.3598680	6.1903850	-2.3475990
O	3.4269590	4.4087260	-3.0517850

TS (D/A-carbene): C–H Functionalization Isomer 1 (TCE: down), TS7

B3LYP-D3(BJ) SCF energy in solution (au): -3606.219752
B3LYP-D3(BJ) enthalpy in solution (au): -3606.168529
B3LYP-D3(BJ) free energy in solution (au): -3606.307251
Imaginary frequency (cm^{-1}): -232.81

Symbol	X	Y	Z
O	3.8484050	-1.1245980	-0.0442110
O	2.3369360	-0.7148470	-2.5706150
O	4.6862090	-1.4440760	-4.0920520
O	6.2083600	-1.7968320	-1.5945330
O	1.6964840	-2.8901500	-2.4479990
O	4.0173210	-3.6101170	-4.0874030
O	5.6221810	-3.9900940	-1.6622440
O	3.3039700	-3.3291890	-0.0201700
C	3.4851870	-2.2019990	0.5352290
C	6.4552830	-3.0462540	-1.5081800
C	4.4546100	-2.5664420	-4.6547510
C	1.4756190	-1.6547070	-2.6317500
C	7.8804710	-3.4160120	-1.1702940
H	8.0649840	-4.4664610	-1.3951770
H	8.5769670	-2.7794700	-1.7199680
H	8.0440210	-3.2454330	-0.1013120
C	0.0536190	-1.2354200	-2.9209540
H	-0.4415440	-1.0039510	-1.9720820
H	0.0398900	-0.3372870	-3.5400850
H	-0.4916980	-2.0453100	-3.4071500
C	3.2250400	-2.1237600	2.0202510
H	3.2960770	-3.1166810	2.4650880
H	3.9253070	-1.4353470	2.4931390
H	2.2147330	-1.7364270	2.1833260
Rh	3.6320510	-3.5364110	-2.0473500
Rh	4.2925050	-1.1658220	-2.0718520
C	4.6959160	-2.6204010	-6.1449240
H	3.7801870	-2.3073290	-6.6586020
H	5.4944420	-1.9326160	-6.4263880
H	4.9339740	-3.6375480	-6.4577020
C	4.8643620	0.8613470	-2.0708510
C	6.1499050	1.3416370	-2.5447970
C	4.1069720	1.6480140	-1.0561620
C	6.6437390	2.6355380	-2.2394780
C	6.8898400	0.5462430	-3.4562270
O	2.9486190	1.9910170	-1.1364680
O	4.8907320	1.9054910	0.0432680
C	7.8200530	3.1029420	-2.8093680
H	6.0918300	3.2723840	-1.5610290
C	8.0617150	1.0226900	-4.0277500
H	6.5174530	-0.4336420	-3.7120350
C	4.3985600	2.8568420	0.9761170

C	8.5277830	2.3034950	-3.7128980
H	8.1848720	4.0940830	-2.5635070
H	8.6122510	0.4015060	-4.7269570
H	5.2323500	3.5180020	1.2183550
H	3.5706810	3.4235250	0.5481020
C	3.9207990	2.2159220	2.2856100
Cl	2.4077140	1.2926380	2.0595880
Cl	5.1956190	1.1434110	2.9607420
Cl	3.6148630	3.5738750	3.4403210
H	9.4413980	2.6767200	-4.1651740
C	3.6292150	1.9709720	-4.1698350
C	3.7252200	1.0809440	-5.3451000
C	4.6827320	1.5174050	-6.1766890
C	5.2784700	2.8028490	-5.6889700
H	2.6675460	2.2689650	-3.7518150
H	4.0385980	1.2906170	-3.2467800
H	3.1457170	0.1742310	-5.4258690
H	5.0024000	1.0408360	-7.0948300
H	5.1297900	3.6349560	-6.3868000
H	6.3551410	2.7340280	-5.4869260
N	4.5160460	3.0348220	-4.4548520
C	4.5051880	4.2209420	-3.7486520
O	3.7955830	4.4059440	-2.7726810
C	5.4970480	6.4654340	-3.7862020
C	5.9423340	6.4595510	-2.3237770
C	6.5864080	7.0452680	-4.6851080
C	4.1678450	7.1924560	-3.9861890
H	5.1830410	6.0144400	-1.6819080
H	6.8783060	5.9067720	-2.2102550
H	6.1182950	7.4897230	-2.0008550
H	6.2776520	7.0154510	-5.7336040
H	6.7811270	8.0847380	-4.4086340
H	7.5139710	6.4760930	-4.5776730
H	4.2881140	8.2474680	-3.7239380
H	3.8572530	7.1326600	-5.0333530
H	3.3878170	6.7622450	-3.3574400
O	5.3748730	5.0815970	-4.2937570

TS (D/A-carbene): C–H Functionalization Isomer 2 (TCE: down), TS8
B3LYP-D3(BJ) SCF energy in solution (au): -3606.221896
B3LYP-D3(BJ) enthalpy in solution (au): -3606.170568
B3LYP-D3(BJ) free energy in solution (au): -3606.309782
Imaginary frequency (cm^{-1}): -315.88

Symbol	X	Y	Z
O	3.6811330	-1.1832050	-0.0883340
O	2.3627360	-0.7345710	-2.6988090
O	4.8313930	-1.4478710	-4.0651340

O	6.1621300	-1.8300220	-1.4822850
O	1.7257890	-2.9135290	-2.6957170
O	4.1777300	-3.6175830	-4.1400100
O	5.5723910	-4.0213620	-1.5896970
O	3.1146110	-3.3813120	-0.1500160
C	3.2521090	-2.2642970	0.4370570
C	6.3955530	-3.0807290	-1.3744700
C	4.6523310	-2.5637090	-4.6575250
C	1.5123520	-1.6746570	-2.8569360
C	7.7856680	-3.4576550	-0.9191000
H	7.9986960	-4.4993380	-1.1598320
H	8.5238460	-2.7986790	-1.3799710
H	7.8481620	-3.3251050	0.1660540
C	0.1178620	-1.2461430	-3.2474950
H	-0.4171430	-0.9288840	-2.3462280
H	0.1620130	-0.3942720	-3.9284950
H	-0.4239970	-2.0746120	-3.7041540
C	2.8522100	-2.2059540	1.8915260
H	2.7730580	-3.2137510	2.2993170
H	3.5735490	-1.6160040	2.4573580
H	1.8809590	-1.7094010	1.9744710
Rh	3.6227220	-3.5645100	-2.1403920
Rh	4.2827530	-1.1943190	-2.0777320
C	5.0144920	-2.5959760	-6.1235520
H	4.1885340	-2.1623320	-6.6976320
H	5.9038260	-1.9902910	-6.3058210
H	5.1726290	-3.6211720	-6.4588100
C	4.8378360	0.8420980	-2.0267800
C	6.0897630	1.3798990	-2.5310130
C	4.1521550	1.6008270	-0.9448580
C	6.4473800	2.7346780	-2.3139750
C	6.9150070	0.6014480	-3.3782960
O	3.0240870	2.0438730	-0.9635200
O	4.9876440	1.7197660	0.1397670
C	7.5617650	3.2911370	-2.9250610
H	5.8310370	3.3538100	-1.6743580
C	8.0364220	1.1585650	-3.9784110
H	6.6522880	-0.4297670	-3.5568500
C	4.6223740	2.6416190	1.1545820
C	8.3546700	2.5055300	-3.7683260
H	7.8036390	4.3356100	-2.7618070
H	8.6608950	0.5495590	-4.6242010
H	5.5318890	3.1810560	1.4242100
H	3.8594390	3.3344740	0.7981290
C	4.0893600	1.9676120	2.4266830
Cl	2.4658380	1.2652010	2.1752930
Cl	5.2273260	0.6981840	2.9905140

Cl	3.9784770	3.2671150	3.6806250
H	9.2226050	2.9401880	-4.2544020
C	3.4817670	1.9179920	-4.0855630
C	3.5674120	0.9690640	-5.2149740
C	4.4982760	1.3805650	-6.0884080
C	5.0786800	2.6981590	-5.6777820
H	2.5291720	2.1967020	-3.6392760
H	3.9647290	1.2872940	-3.1466510
H	3.0086440	0.0463040	-5.2295790
H	4.8146300	0.8595860	-6.9830060
H	4.9154400	3.4942950	-6.4132160
H	6.1583280	2.6549440	-5.4818240
N	4.3237060	2.9902970	-4.4543240
C	4.3175750	4.2725030	-3.9356580
O	5.0300530	5.1538190	-4.3894390
C	3.2427190	5.6417170	-2.2048820
C	2.7931100	6.7258250	-3.1843500
C	2.1207880	5.2938060	-1.2297400
C	4.5264460	6.0190150	-1.4666920
H	3.5944080	7.0048820	-3.8674460
H	1.9343730	6.3755850	-3.7645880
H	2.4839220	7.6097650	-2.6189800
H	2.3935030	4.4278020	-0.6243340
H	1.9233930	6.1467680	-0.5751050
H	1.2027550	5.0521310	-1.7726890
H	4.3627050	6.9395560	-0.8993670
H	4.8089190	5.2346990	-0.7584310
H	5.3464400	6.1819560	-2.1671010
O	3.4534680	4.3635970	-2.9205560

TS (D/A-carbene): Cyclopropanation (TCE: Down), TS9

B3LYP-D3(BJ) SCF energy in solution (au): -3606.212893
B3LYP-D3(BJ) enthalpy in solution (au): -3606.161803
B3LYP-D3(BJ) free energy in solution (au): -3606.300222
Imaginary frequency (cm^{-1}): -146.69

Symbol	X	Y	Z
O	-1.1903030	0.9081280	-0.2024680
O	-2.2031210	1.0895950	-2.9342230
O	0.4943850	0.6187290	-3.9912770
O	1.5158270	0.5054340	-1.2222390
O	-2.5464940	-1.1507940	-2.9577210
O	0.1520590	-1.6177310	-4.0108560
O	1.1969880	-1.7406260	-1.2834100
O	-1.5023680	-1.3403370	-0.2462750
C	-1.5902500	-0.1962860	0.2972050
C	1.8723700	-0.7014950	-1.0129500
C	0.5693120	-0.5402250	-4.5243480

C	-2.9002850	0.0458650	-3.1755910
C	3.2202980	-0.8872460	-0.3592090
H	3.9342380	-0.1685120	-0.7659990
H	3.1209630	-0.6912300	0.7134370
H	3.5785900	-1.9069480	-0.5008330
C	-4.2742830	0.2853890	-3.7517930
H	-4.2176650	1.0237000	-4.5546850
H	-4.7040860	-0.6454300	-4.1211010
H	-4.9194710	0.6950260	-2.9681360
C	-2.2416240	-0.1466710	1.6566250
H	-2.4184310	-1.1578350	2.0221210
H	-1.6105280	0.4058190	2.3533400
H	-3.1927710	0.3865950	1.5808710
Rh	-0.6878510	-1.5255120	-2.1189210
Rh	-0.3166440	0.9087350	-2.0932980
C	1.2453110	-0.6037070	-5.8717380
H	1.0063040	-1.5380680	-6.3794260
H	0.9503090	0.2518270	-6.4819000
H	2.3286730	-0.5486920	-5.7210760
C	0.1481500	3.0213970	-1.9821070
C	1.5060480	3.4982200	-2.1943400
C	-0.6607260	3.6493560	-0.8925870
C	1.9700620	4.7317080	-1.6649130
C	2.4231430	2.7192410	-2.9383760
O	-1.8069460	4.0428520	-0.9436710
O	0.0786040	3.6762280	0.2640850
C	3.2838270	5.1432940	-1.8420960
H	1.2916120	5.3662240	-1.1078140
C	3.7361680	3.1383290	-3.1167670
H	2.0953320	1.7823060	-3.3605400
C	-0.3425060	4.5658220	1.2885940
C	4.1727690	4.3466930	-2.5709660
H	3.6142840	6.0879620	-1.4231810
H	4.4246130	2.5162330	-3.6781650
H	5.1988060	4.6704410	-2.7143010
C	0.2361780	5.6484820	-4.2100660
C	-0.8456920	4.9596210	-3.4570710
C	-1.0136600	3.6744380	-3.8720730
C	-0.1232330	3.3928900	-5.0512570
H	-0.1393350	6.5504730	-4.7160370
H	1.0609200	5.9733380	-3.5586610
H	-1.4271890	5.4496430	-2.6913420
H	-1.8263760	3.0188920	-3.5916080
H	-0.7294870	3.2335880	-5.9545950
H	0.4994720	2.5106810	-4.9143840
N	0.6782770	4.6181160	-5.1426960
H	0.5565060	5.0803560	1.6323380

H	-1.0708370	5.2821610	0.9058320
C	1.8502670	4.7482610	-5.8149090
O	2.5380550	5.7643270	-5.7755060
O	2.1197940	3.6223780	-6.5132800
C	3.2458550	3.5700220	-7.4578340
C	3.1236000	2.1641220	-8.0433470
H	2.1556850	2.0342480	-8.5357310
H	3.9141980	1.9958980	-8.7796220
H	3.2174160	1.4118850	-7.2555980
C	4.5718250	3.7315960	-6.7140040
H	5.4004110	3.6111220	-7.4184720
H	4.6392400	4.7138080	-6.2476770
H	4.6660130	2.9643850	-5.9413740
C	3.0558810	4.6299510	-8.5441110
H	3.8265000	4.5093770	-9.3111710
H	2.0776040	4.5116080	-9.0198570
H	3.1287990	5.6337950	-8.1256520
C	-0.9613100	3.8626860	2.5040560
Cl	0.1016590	2.5302420	3.0676470
Cl	-1.0841640	5.1149080	3.8048320
Cl	-2.5974890	3.2393160	2.1510530

***exo*-3•$Rh_2(OAc)_4$**

B3LYP-D3(BJ) SCF energy in solution (au): -1996.493621
B3LYP-D3(BJ) enthalpy in solution (au): -1996.450416
B3LYP-D3(BJ) free energy in solution (au): -1996.571797

Symbol	X	Y	Z
O	-1.6601330	0.2077030	0.7660480
O	-1.4414850	1.5200460	-1.8120280
O	1.4785510	1.1532480	-1.7397460
O	1.2422910	-0.1564620	0.8366310
O	-1.6265090	-0.4665470	-2.8838310
O	1.2607770	-0.8446910	-2.7818530
O	1.0005700	-2.1622980	-0.1894740
O	-1.8902420	-1.7858180	-0.2837280
C	-2.2153440	-0.9275860	0.5964300
C	1.4875470	-1.4004220	0.7059000
C	1.8093960	0.2911360	-2.6195600
C	-1.8994540	0.7667090	-2.7315690
C	2.4094640	-2.0250610	1.7217850
H	3.1469280	-1.2966910	2.0614700
H	1.8143120	-2.3393210	2.5858580
H	2.9006620	-2.9033950	1.3020420
C	-2.8127350	1.4052990	-3.7467990
H	-2.2111540	1.7260930	-4.6040580
H	-3.5502250	0.6837490	-4.1008050
H	-3.3040660	2.2803890	-3.3207990

C	-3.3296970	-1.2904010	1.5444050
H	-3.9878910	-2.0331960	1.0930620
H	-2.8909060	-1.7183250	2.4520610
H	-3.8915880	-0.3985220	1.8255240
Rh	-0.3196860	-1.3720480	-1.5627800
Rh	-0.0893610	0.7490220	-0.4587800
C	2.9147670	0.6807090	-3.5631280
H	3.6898850	1.2317080	-3.0273990
H	3.3356880	-0.2000060	-4.0484490
H	2.4989510	1.3464690	-4.3257050
C	0.3974510	4.0470740	-1.4040690
C	-0.3707000	3.9968270	-0.1470260
O	-1.2457960	4.7751850	0.1754400
O	0.0169880	2.9474420	0.6463620
C	-0.5624430	2.8925110	1.9927100
H	-0.8641530	3.9040720	2.2620260
C	1.6478140	5.5479850	-3.0667320
C	0.4790130	5.3870220	-2.1172480
C	-0.4188860	4.3228190	-2.6585780
C	0.1803840	3.7796330	-3.9367080
H	1.5699580	6.4966720	-3.6132760
H	2.6236820	5.5228970	-2.5738410
H	0.0810250	6.2558500	-1.6071350
H	-1.4948860	4.3739430	-2.5541480
H	-0.4224590	4.0733880	-4.8053020
H	0.2659140	2.6908690	-3.9298320
N	1.5126180	4.4005240	-3.9738420
H	-1.4453700	2.2543080	1.9356630
C	2.4212860	4.2169760	-4.9675330
O	3.4353770	4.8956680	-5.0930640
O	2.0485610	3.1852740	-5.7658940
C	2.7769330	2.8892790	-7.0120470
C	1.9863490	1.7145210	-7.5861420
H	0.9449440	1.9994700	-7.7595270
H	2.4248810	1.4010650	-8.5373690
H	2.0049640	0.8632480	-6.8996560
C	4.2184630	2.4775470	-6.7083950
H	4.7102200	2.1783360	-7.6389640
H	4.7728760	3.3022130	-6.2623860
H	4.2389130	1.6233770	-6.0260750
C	2.7046790	4.1007280	-7.9426980
H	3.1431440	3.8435950	-8.9113730
H	1.6621970	4.3911060	-8.1045500
H	3.2489810	4.9472250	-7.5236700
H	1.2234110	3.3534770	-1.4957950
C	0.4866840	2.3303700	2.9288410
H	1.3602700	2.9875950	2.9718560

H	0.0618770	2.2567470	3.9350940
H	0.8082790	1.3373650	2.6091630

Pre-reaction complex, Int1
B3LYP-D3(BJ) SCF energy in solution (au): -1996.406129
B3LYP-D3(BJ) enthalpy in solution (au): -1996.361172
B3LYP-D3(BJ) free energy in solution (au): -1996.487968

Symbol	X	Y	Z
O	-0.8340050	-0.9857220	2.0880000
O	-1.9023900	-0.4953960	-0.5620410
O	0.7251870	0.4582020	-1.4539720
O	1.8324670	-0.1480050	1.1765070
O	-1.3979620	-2.6130400	-1.1953080
O	1.2155390	-1.6629970	-2.1054790
O	2.3412430	-2.2619060	0.5295820
O	-0.3005630	-3.1067200	1.4760800
C	-0.7833270	-2.2509330	2.2746360
C	2.6067450	-1.1631960	1.0981540
C	1.1918470	-0.4070620	-2.2729570
C	-2.1733070	-1.6154860	-1.1197250
C	3.9502050	-1.0137070	1.7684970
H	4.5949270	-1.8570300	1.5225220
H	4.4163700	-0.0758460	1.4581060
H	3.8041120	-0.9696530	2.8520620
C	-3.5459640	-1.7260430	-1.7317620
H	-3.6812490	-2.7034420	-2.1937060
H	-4.3009260	-1.5718330	-0.9557550
H	-3.6688720	-0.9321540	-2.4722160
C	-1.3773480	-2.7426010	3.5720450
H	-1.1345980	-3.7927940	3.7316710
H	-1.0063680	-2.1360780	4.4014400
H	-2.4644630	-2.6226300	3.5327160
Rh	0.4842120	-2.4883640	-0.3487040
Rh	-0.0696190	-0.1952470	0.3371470
C	1.7934630	0.1326210	-3.5467080
H	1.5794310	-0.5433820	-4.3763270
H	1.4165880	1.1332570	-3.7497450
H	2.8807910	0.1826360	-3.4238980
C	-0.3989050	1.6201050	0.9435510
C	-1.1030360	1.8747290	2.1967570
O	-2.2975020	2.1315170	2.1158160
O	-0.3930760	1.7402460	3.3057120
C	-1.1299840	1.8012650	4.5641370
H	-1.5082130	2.8187500	4.6925360
H	-1.9801160	1.1191730	4.4906680
C	-2.5952000	2.5812610	-1.6457520
C	-3.5734290	1.8836400	-2.5475280

C	-3.0292530	1.5795400	-3.7267950
C	-1.5943770	2.0266960	-3.8059700
H	-2.9352780	3.5719530	-1.3190010
H	-2.3834530	1.9919730	-0.7445460
H	-4.5860970	1.6621260	-2.2328270
H	-3.5180680	1.0683970	-4.5477940
H	-1.4023040	2.7167100	-4.6369410
H	-0.9043450	1.1807420	-3.9189880
N	-1.4157520	2.6913780	-2.5112330
C	-0.3284200	3.3897090	-2.1273620
O	-0.2350470	3.9420660	-1.0259950
O	0.6115140	3.4157140	-3.0970650
H	-0.1035170	2.4976080	0.3539780
C	1.8845690	4.1294380	-2.8891180
C	1.6154620	5.6222100	-2.6906370
H	1.0670680	5.8005780	-1.7663600
H	2.5674870	6.1598510	-2.6482860
H	1.0372000	6.0170030	-3.5315890
C	2.6500440	3.5114710	-1.7170000
H	2.7132790	2.4270470	-1.8340090
H	3.6647310	3.9202400	-1.6925030
H	2.1577950	3.7294390	-0.7698170
C	2.6233620	3.8936430	-4.2063340
H	3.5759160	4.4301650	-4.1955260
H	2.8297770	2.8314340	-4.3577250
H	2.0291740	4.2575140	-5.0491260
C	-0.1680490	1.4018750	5.6616600
H	-0.6824880	1.4353310	6.6263120
H	0.2016080	0.3854370	5.5005590
H	0.6857640	2.0836380	5.7017160

Hydride transfer product, Int2

B3LYP-D3(BJ) SCF energy in solution (au): -1996.454120
B3LYP-D3(BJ) enthalpy in solution (au): -1996.410139
B3LYP-D3(BJ) free energy in solution (au): -1996.531424

Symbol	X	Y	Z
O	-0.0554690	-1.1661610	2.3854630
O	-1.6653380	-0.5806900	-0.0002240
O	0.8059240	0.1115110	-1.4774000
O	2.3789360	-0.4521800	0.9316750
O	-1.4487500	-2.7475520	-0.6563590
O	1.0307800	-2.0495340	-2.1396990
O	2.6075560	-2.6149510	0.2661350
O	0.1548770	-3.3295890	1.7171270
C	-0.0706050	-2.4247530	2.5795760
C	3.0475800	-1.5200990	0.7347620
C	1.0576700	-0.8011270	-2.3394050

C	-2.1169450	-1.6931070	-0.4275460
C	4.5072590	-1.4730900	1.1300610
H	5.0584280	-2.2908310	0.6653140
H	4.9436400	-0.5121290	0.8501110
H	4.5816840	-1.5697490	2.2183340
C	-3.6057540	-1.7645910	-0.6937020
H	-3.7791990	-1.9669040	-1.7550450
H	-4.0336190	-2.5980220	-0.1302090
H	-4.0980130	-0.8356010	-0.4082110
C	-0.4281940	-2.8830350	3.9763320
H	0.0268230	-3.8516160	4.1880920
H	-0.1145060	-2.1430310	4.7138190
H	-1.5161630	-2.9926850	4.0423300
Rh	0.5953820	-2.7852510	-0.2361730
Rh	0.3484100	-0.4301040	0.4907390
C	1.4489750	-0.3286520	-3.7219200
H	1.1241720	-1.0477220	-4.4752790
H	1.0355920	0.6590140	-3.9288850
H	2.5407910	-0.2554200	-3.7695990
C	0.2586510	1.6616820	1.0826490
C	-0.9864560	1.9250170	1.7758900
O	-2.0594090	2.3063730	1.2806220
O	-0.9136130	1.6580820	3.1064540
C	-2.1572420	1.6381490	3.8352850
H	-1.8626040	1.7510940	4.8811190
H	-2.7625240	2.4989020	3.5403800
C	-2.5562860	3.2006190	-0.8617080
C	-3.4790430	2.1689330	-1.2289070
C	-2.8757100	1.3965550	-2.1642140
C	-1.5114850	1.9073600	-2.4449100
H	-2.6966950	4.0351040	-0.1938490
H	-4.4547330	2.0430800	-0.7847150
H	-3.2820220	0.5177480	-2.6458270
H	-1.3365050	2.1675230	-3.4932340
H	-0.7288090	1.2023420	-2.1233270
N	-1.4588450	3.1051590	-1.5995760
C	-0.3397210	3.9819430	-1.4532700
O	-0.4042550	4.9674460	-0.7550390
C	2.0095540	4.1464850	-2.1558030
C	2.5438390	4.1562630	-0.7263430
C	2.8135180	3.1995460	-3.0389980
C	1.8982750	5.5411540	-2.7630310
H	1.9535990	4.8070610	-0.0810640
H	2.5459200	3.1454650	-0.3112510
H	3.5737960	4.5232680	-0.7421670
H	2.3914010	3.1561240	-4.0466320
H	3.8450700	3.5534810	-3.1089490

H	2.8132160	2.1940040	-2.6119760
H	2.9003670	5.9685090	-2.8576800
H	1.4521810	5.4898300	-3.7601190
H	1.2978960	6.1998580	-2.1343010
O	0.6551820	3.5082240	-2.1731520
C	-2.9079510	0.3336350	3.6101840
H	-2.2814910	-0.5171750	3.8848690
H	-3.8195650	0.3169870	4.2167750
H	-3.1826940	0.2299360	2.5583150
H	1.1381700	1.7760430	1.7140520
H	0.3477580	2.1927830	0.1411920

51•52•$Rh_2(OAc)_4$

B3LYP-D3(BJ) SCF energy in solution (au): -1996.512408
B3LYP-D3(BJ) enthalpy in solution (au): -1996.467156
B3LYP-D3(BJ) free energy in solution (au): -1996.595145

Symbol	X	Y	Z
O	0.0681080	-1.8211050	2.4273360
O	-1.5154670	-1.0685600	0.0972650
O	0.9032320	-0.0927090	-1.2344340
O	2.4856940	-0.8599970	1.1212840
O	-1.1875200	-3.1326950	-0.7803530
O	1.1822370	-2.1465340	-2.1470080
O	2.8280950	-2.8836350	0.1616080
O	0.4439070	-3.8714260	1.5340440
C	0.1371150	-3.0918290	2.4919240
C	3.2197400	-1.8458260	0.7816410
C	1.1414720	-0.8787120	-2.2102740
C	-1.9111490	-2.1279450	-0.4901840
C	4.6725000	-1.7814710	1.1820440
H	5.2739460	-2.4267990	0.5414140
H	5.0320720	-0.7522430	1.1370210
H	4.7664390	-2.1294610	2.2161490
C	-3.3641640	-2.1717720	-0.8936040
H	-3.5114630	-1.5051890	-1.7496260
H	-3.6576730	-3.1832910	-1.1733190
H	-3.9882150	-1.8059700	-0.0755050
C	-0.2083800	-3.7275850	3.8155380
H	0.3110410	-4.6800910	3.9264430
H	0.0409070	-3.0553170	4.6374580
H	-1.2868270	-3.9170120	3.8409710
Rh	0.8288840	-3.0731770	-0.3314550
Rh	0.4746690	-0.8908310	0.6311590
C	1.4272570	-0.2374980	-3.5450030
H	1.1774320	-0.9211470	-4.3567920
H	0.8717880	0.6962130	-3.6435710
H	2.4960960	-0.0069150	-3.6011570

C	1.9712350	2.2864660	0.6208550
C	0.6334910	2.1986900	1.2888950
O	0.0369910	1.1508650	1.5445120
O	0.1182610	3.3890420	1.5714630
C	-1.2143790	3.4277340	2.1649060
H	-1.5890150	4.4148850	1.8932400
H	-1.8256100	2.6683150	1.6781630
C	-2.4481630	4.2084450	-1.2011080
C	-3.2331840	3.1550180	-0.8189050
C	-2.4484490	1.9563620	-0.9425280
C	-1.2104940	2.3117260	-1.4060570
H	-2.6314740	5.2683230	-1.2633850
H	-4.2570450	3.2250360	-0.4790270
H	-2.7395900	0.9485290	-0.6861370
H	-0.3356330	1.7169850	-1.6069420
H	1.8086980	2.2273010	-0.4582260
N	-1.2035210	3.6976920	-1.5666770
C	-0.1518900	4.5076520	-2.0129760
O	-0.2504240	5.7183550	-2.0729350
C	2.1285340	4.3935100	-2.9029980
C	2.7445280	5.3295430	-1.8646660
C	3.0342800	3.1940110	-3.1685140
C	1.7818130	5.1071040	-4.2092400
H	2.0857290	6.1710180	-1.6523730
H	2.9471490	4.7904410	-0.9357020
H	3.6937000	5.7137430	-2.2491590
H	2.5768750	2.5190220	-3.8952170
H	3.9894560	3.5407000	-3.5712940
H	3.2299120	2.6376510	-2.2482650
H	2.7056280	5.4456660	-4.6869290
H	1.2761190	4.4190650	-4.8929670
H	1.1412330	5.9706570	-4.0329170
O	0.9051020	3.7619220	-2.3446460
C	-1.1416100	3.2370710	3.6685140
H	-0.4876190	3.9849050	4.1257650
H	-2.1424470	3.3469970	4.0972790
H	-0.7696720	2.2399910	3.9163160
H	2.4565850	3.2330220	0.8575470
H	2.5840080	1.4358660	0.9169760

53 • $Rh_2(OAc)_4$

B3LYP-D3(BJ) SCF energy in solution (au): -1996.457764
B3LYP-D3(BJ) enthalpy in solution (au): -1996.414658
B3LYP-D3(BJ) free energy in solution (au): -1996.533637

Symbol	X	Y	Z
O	-0.1221830	-1.2545750	2.6406900
O	-1.4716740	-0.5017290	0.1133920

O	1.0843250	0.3025880	-1.0038900
O	2.4326750	-0.4387010	1.4964260
O	-1.2004350	-2.6377820	-0.6016030
O	1.3229520	-1.8073540	-1.8117510
O	2.7516840	-2.5214300	0.6502240
O	0.2121840	-3.3598340	1.8602740
C	-0.1208220	-2.5227550	2.7568000
C	3.1369900	-1.4734110	1.2572200
C	1.3498560	-0.5413070	-1.9208760
C	-1.8835510	-1.5827460	-0.4206970
C	4.5560590	-1.4580540	1.7754920
H	5.1752710	-2.1584750	1.2143050
H	4.9701260	-0.4500540	1.7183530
H	4.5491390	-1.7632950	2.8273390
C	-3.3315060	-1.6095970	-0.8497260
H	-3.4516570	-2.2167080	-1.7484330
H	-3.9244360	-2.0653700	-0.0494260
H	-3.6969710	-0.5958530	-1.0159440
C	-0.5974040	-3.0811600	4.0773430
H	-0.0962670	-4.0256720	4.2940410
H	-0.4275540	-2.3634550	4.8806270
H	-1.6736060	-3.2739850	4.0079030
Rh	0.7889270	-2.6576380	-0.0032460
Rh	0.4627560	-0.3913220	0.8494340
C	1.7462500	0.0254210	-3.2618790
H	1.5871920	-0.7096240	-4.0512400
H	1.1790660	0.9354700	-3.4539330
H	2.8095300	0.2864280	-3.2366710
C	0.3648290	1.7406480	1.7036600
C	-0.9924960	1.8122290	1.9909290
O	-1.9841630	2.1581010	1.1913620
O	-1.3969110	1.4079860	3.1901910
C	-2.8107370	1.2186620	3.4673180
H	-2.8522940	1.2142940	4.5574750
H	-3.3669770	2.0805180	3.0939750
C	-1.9110760	3.0165100	-0.0160730
C	-3.2459790	2.7929500	-0.6707780
C	-3.0900630	2.1439360	-1.8247730
C	-1.6371710	1.9153550	-2.1284420
H	-1.6925470	4.0154100	0.3609860
H	-4.1735490	3.0736090	-0.1895970
H	-3.8815410	1.7989810	-2.4792550
H	-1.3319540	2.3246770	-3.0930460
H	-1.3688070	0.8551460	-2.0986590
N	-0.9723670	2.6453830	-1.0366590
C	0.1440030	3.4355250	-1.2201260
O	0.5715780	4.1784990	-0.3447020

C	1.8662420	4.0552100	-2.8453220
C	3.0509260	3.6224670	-1.9825230
C	2.0654520	3.6474940	-4.3039230
C	1.5819460	5.5555200	-2.7552650
H	2.9198550	3.9419540	-0.9488090
H	3.1559010	2.5346710	-2.0048470
H	3.9689090	4.0680840	-2.3768740
H	1.1695530	3.8699010	-4.8902220
H	2.9032500	4.2082900	-4.7265090
H	2.2878720	2.5828030	-4.3928080
H	2.4266410	6.1036680	-3.1826420
H	0.6870080	5.8055810	-3.3331500
H	1.4407090	5.8728890	-1.7235280
O	0.6641230	3.2846260	-2.4456480
C	-3.3262550	-0.0848350	2.8824090
H	-2.7342140	-0.9254720	3.2493910
H	-4.3704100	-0.2256040	3.1795300
H	-3.2606720	-0.0703090	1.7941580
H	1.0064140	1.6185040	2.5689000
H	0.7507180	2.3350620	0.8913020

References From Section 11 Computational Calculations

(1) Frisch, M. J.; Trucks, G. W.; Schlegel, H. B.; Scuseria, G. E.; Robb, M. A.; Cheeseman, J. R.; Scalmani, G.; Barone, V.; Petersson, G. A.; Nakatsuji, H.; et al. Gaussian 16 Rev. C.01. 2016.
(2) Lee, C.; Yang, W.; Parr, R. G. Development of the Colle-Salvetti correlation-energy formula into a functional of the electron density. Phys. Rev. B 1988, 37 (2), 785-789.
(3) Becke, A. D. A new mixing of Hartree–Fock and local density-functional theories. J. Chem. Phys. 1993, 98 (2), 1372-1377.
(4) Becke, A. D. Density-functional thermochemistry. III. The role of exact exchange. J. Chem. Phys. 1993, 98 (7), 5648-5652.
(5) Grimme, S.; Hansen, A.; Brandenburg, J. G.; Bannwarth, C. Dispersion-Corrected Mean-Field Electronic Structure Methods. Chem. Rev. 2016, 116 (9), 5105-5154.
(6) Grimme, S.; Antony, J.; Ehrlich, S.; Krieg, H. A consistent and accurate ab initio parametrization of density functional dispersion correction (DFT-D) for the 94 elements H-Pu. J. Chem. Phys. 2010, 132 (15), 154104.
(7) Johnson, E. R.; Becke, A. D. A post-Hartree-Fock model of intermolecular interactions: Inclusion of higher-order corrections. J. Chem. Phys. 2006, 124 (17), 174104.
(8) Hay, P. J.; Wadt, W. R. Ab initio effective core potentials for molecular calculations. Potentials for K to Au including the outermost core orbitals. J. Chem. Phys. 1985, 82 (1), 299-310.
(9) Roy, L. E.; Hay, P. J.; Martin, R. L. Revised Basis Sets for the LANL Effective Core Potentials. J. Chem. Theory Comput. 2008, 4 (7), 1029-1031.
(10) Hariharan, P. C.; Pople, J. A. The influence of polarization functions on molecular orbital hydrogenation energies. Theoretica chimica acta 1973, 28, 213-222.
(11) Hehre, W. J.; Ditchfield, R.; Pople, J. A. Self—Consistent Molecular Orbital Methods. XII. Further Extensions of Gaussian—Type Basis Sets for Use in Molecular Orbital Studies of Organic Molecules. J. Chem. Phys. 1972, 56 (5), 2257-2261.

(12) Cossi, M.; Rega, N.; Scalmani, G.; Barone, V. Energies, structures, and electronic properties of molecules in solution with the C-PCM solvation model. J. Comput. Chem. 2003, 24 (6), 669-681.
(13) Barone, V.; Cossi, M. Quantum Calculation of Molecular Energies and Energy Gradients in Solution by a Conductor Solvent Model. The Journal of Physical Chemistry A 1998, 102 (11), 1995-2001.

12. References

(1) Green, S. P.; Wheelhouse, K. M.; Payne, A. D.; Hallett, J. P.; Miller, P. W.; Bull, J. A. Thermal Stability and Explosive Hazard Assessment of Diazo Compounds and Diazo Transfer Reagents. *Org. Process Res. Dev.* **2020**, *24* (1), 67-84. DOI: 10.1021/acs.oprd.9b00422.
(2) Lyon, W. L.; Wang, J. Z.; Alcázar, J.; MacMillan, D. W. C. Aminoalkylation of Alkenes Enabled by Triple Radical Sorting. *Journal of the American Chemical Society* **2025**, *147* (3), 2296-2302. DOI: 10.1021/jacs.4c14965.
(3) Nguyen, T.-T. H.; Navarro, A.; Ruble, J. C.; Davies, H. M. L. Stereoselective Synthesis of Either Exo- or Endo-3-Azabicyclo[3.1.0]hexane-6-carboxylates by Dirhodium(II)-Catalyzed Cyclopropanation with Ethyl Diazoacetate under Low Catalyst Loadings. *Organic Letters* **2024**, *26* (14), 2832-2836. DOI: 10.1021/acs.orglett.3c03652.
(4) Fu, L.; Mighion, J. D.; Voight, E. A.; Davies, H. M. L. Synthesis of 2,2,2,-Trichloroethyl Aryl- and Vinyldiazoacetates by Palladium-Catalyzed Cross-Coupling. *Chemistry – A European Journal* **2017**, *23* (14), 3272-3275. DOI: https://doi.org/10.1002/chem.201700101.
(5) Bess, E. N.; Guptill, D. M.; Davies, H. M. L.; Sigman, M. S. Using IR vibrations to quantitatively describe and predict site-selectivity in multivariate Rh-catalyzed C–H functionalization. *Chem. Sci.* **2015**, *6* (5), 3057-3062, 10.1039/C5SC00357A. DOI: 10.1039/C5SC00357A.
(6) Nguyen, T.-T. H.; Bosse, A. T.; Ly, D.; Suarez, C. A.; Fu, J.; Shimabukuro, K.; Musaev, D. G.; Davies, H. M. L. Diaryldiazoketones as Effective Carbene Sources for Highly Selective Rh(II)-Catalyzed Intermolecular C–H Functionalization. *Journal of the American Chemical Society* **2024**, *146* (12), 8447-8455. DOI: 10.1021/jacs.3c14552.
(7) Liu, W.; Babl, T.; Röther, A.; Reiser, O.; Davies, H. M. L. Functionalization of Piperidine Derivatives for the Site-Selective and Stereoselective Synthesis of Positional Analogues of Methylphenidate. *Chemistry – A European Journal* **2020**, *26* (19), 4236-4241. DOI: https://doi.org/10.1002/chem.201905773.
(8) Fischer, S.; Nguyen, T.-T. H.; Ratzenboeck, A.; Davies, H. M. L.; Reiser, O. Stereoselective Synthesis of Highly Functionalized Cyclohexenes via Strong-Acid-Mediated Endocyclic C–C Bond Cleavage of Monocyclopropanated Cyclopentadienes. *Organic Letters* **2023**, *25* (24), 4411-4415. DOI: 10.1021/acs.orglett.3c00935.
(9) Poh, J.-S.; Makai, S.; von Keutz, T.; Tran, D. N.; Battilocchio, C.; Pasau, P.; Ley, S. V. Rapid Asymmetric Synthesis of Disubstituted Allenes by Coupling of Flow-Generated Diazo Compounds and Propargylated Amines. *Angewandte Chemie International Edition* **2017**, *56* (7), 1864-1868. DOI: https://doi.org/10.1002/anie.201611067.

(10) Davies, H. M. L.; Bruzinski, P. R.; Lake, D. H.; Kong, N.; Fall, M. J. Asymmetric Cyclopropanations by Rhodium(II) N-(Arylsulfonyl)prolinate Catalyzed Decomposition of Vinyldiazomethanes in the Presence of Alkenes. Practical Enantioselective Synthesis of the Four Stereoisomers of 2-Phenylcyclopropan-1-amino Acid. *Journal of the American Chemical Society* **1996**, *118* (29), 6897-6907. DOI: 10.1021/ja9604931.
(11) Fu, J. T.; Ren, Z.; Bacsa, J.; Musaev, D. G.; Davies, H. M. L. Desymmetrization of cyclohexanes by site- and stereoselective C-H functionalization. *Nature* **2018**, *564* (7736), 395-+, Article. DOI: 10.1038/s41586-018-0799-2.
(12) Tsutsui, H.; Abe, T.; Nakamura, S.; Anada, M.; Hashimoto, S. Practical synthesis of dirhodium(II) Tetrakis N-phthaloyl-(S)-tert-leucinate. *Chem. Pharm. Bull.* **2005**, *53* (10), 1366-1368, Article. DOI: 10.1248/cpb.53.1366.
(13) Reddy, R. P.; Davies, H. M. L. Dirhodium Tetracarboxylates Derived from Adamantylglycine as Chiral Catalysts for Enantioselective C−H Aminations. *Organic Letters* **2006**, *8* (22), 5013-5016. DOI: 10.1021/ol061742l.
(14) Nasrallah, A.; Boquet, V.; Hecker, A.; Retailleau, P.; Darses, B.; Dauban, P. Catalytic Enantioselective Intermolecular Benzylic C(sp3)−H Amination. *Angewandte Chemie International Edition* **2019**, *58* (24), 8192-8196. DOI: https://doi.org/10.1002/anie.201902882.
(15) Reddy, R. P.; Davies, H. M. L. Dirhodium tetracarboxylates derived from adamantylglycine as chiral catalysts for enantioselective C-h aminations. *Org. Lett.* **2006**, *8* (22), 5013-5016. DOI: 10.1021/ol061742l.
(16) Liu, W.; Ren, Z.; Bosse, A. T.; Liao, K.; Goldstein, E. L.; Bacsa, J.; Musaev, D. G.; Stoltz, B. M.; Davies, H. M. L. Catalyst-Controlled Selective Functionalization of Unactivated C–H Bonds in the Presence of Electronically Activated C–H Bonds. *Journal of the American Chemical Society* **2018**, *140* (38), 12247-12255. DOI: 10.1021/jacs.8b07534.
(17) Qin, C.; Davies, H. M. L. Role of Sterically Demanding Chiral Dirhodium Catalysts in Site-Selective C–H Functionalization of Activated Primary C–H Bonds. *Journal of the American Chemical Society* **2014**, *136* (27), 9792-9796. DOI: 10.1021/ja504797x.
(18) Lindsay, V. N. G.; Lin, W.; Charette, A. B. Experimental Evidence for the All-Up Reactive Conformation of Chiral Rhodium(II) Carboxylate Catalysts: Enantioselective Synthesis of cis-Cyclopropane α-Amino Acids. *Journal of the American Chemical Society* **2009**, *131* (45), 16383-16385. DOI: 10.1021/ja9044955.
(19) Davies, H. M. L.; Ren, P. Conformational analysis and stereochemical assignments of products derived from C・H activation at secondary sites. *Tetrahedron Letters* **2001**, *42* (18), 3149-3151. DOI: https://doi.org/10.1016/S0040-4039(01)00385-9.
(20) Hansen, J.; Autschbach, J.; Davies, H. M. L. Computational Study on the Selectivity of Donor/Acceptor-Substituted Rhodium Carbenoids. *The Journal of Organic Chemistry* **2009**, *74* (17), 6555-6563. DOI: 10.1021/jo9009968.
(21) Gwak, S.; Lee, J. H.; Kwon, H.-J.; Han, H. A Study on the Diazo-Transfer Reaction Using o-Nitrobenzenesulfonyl Azide. *Synlett* **2023**, *35* (12), 1429-1435. DOI: 10.1055/a-2184-4836.
(22) Tortoreto, C.; Rackl, D.; Davies, H. M. L. Metal-Free C–H Functionalization of Alkanes by Aryldiazoacetates. *Organic Letters* **2017**, *19* (4), 770-773. DOI: 10.1021/acs.orglett.6b03681.

(23) Zhang, B.; Hollerbach, M. R.; Blakey, S. B.; Davies, H. M. L. C–H Functionalization Approach for the Synthesis of Chiral C2-Symmetric 1,5-Cyclooctadiene Ligands. *Organic Letters* **2019**, *21* (24), 9864-9868. DOI: 10.1021/acs.orglett.9b03764.
(24) Alcaide, B.; Almendros, P.; Aragoncillo, C.; Gómez-Campillos, G.; Quirós, M. T.; Soriano, E. Tunable Metal-Catalyzed Heterocyclization Reactions of Allenic Amino Alcohols: An Experimental and Theoretical Study. *The Journal of Organic Chemistry* **2016**, *81* (17), 7362-7372. DOI: 10.1021/acs.joc.6b00934.
(25) Soai, K.; Ookawa, A. Mixed solvents containing methanol as useful reaction media for unique chemoselective reductions within lithium borohydride. *The Journal of Organic Chemistry* **1986**, *51* (21), 4000-4005. DOI: 10.1021/jo00371a017.
(26) Sapkota, K.; Huang, F. Selective Protection of Secondary Alcohols by Using Formic Acid as a Mild and Efficient Deprotection Reagent for Primary tert-Butyldimethylsilyl Ethers. *Synlett* **2019**, *30* (16), 1895-1898. DOI: 10.1055/s-0037-1611757.
(27) Kumar, T. P.; Chandrasekhar, S. Asymmetric Syntheses of All Stereoisomers of 3-Hydroxyproline; A Constituent of Several Bioactive Compounds. *Synthesis* **2012**, *44* (18), 2889-2894. DOI: 10.1055/s-0032-1316734.
(28) Etling, C.; Tedesco, G.; Di Marco, A.; Kalesse, M. Asymmetric Total Synthesis of Illisimonin A. *Journal of the American Chemical Society* **2023**, *145* (12), 7021-7029. DOI: 10.1021/jacs.3c01262.
(29) Senaweera, S.; Cartwright, K. C.; Tunge, J. A. Decarboxylative Acetoxylation of Aliphatic Carboxylic Acids. *The Journal of Organic Chemistry* **2019**, *84* (19), 12553-12561. DOI: 10.1021/acs.joc.9b02092.
(30) Reed, N. L.; Herman, M. I.; Miltchev, V. P.; Yoon, T. P. Photocatalytic Oxyamination of Alkenes: Copper(II) Salts as Terminal Oxidants in Photoredox Catalysis. *Organic Letters* **2018**, *20* (22), 7345-7350. DOI: 10.1021/acs.orglett.8b03345.
(31) Gilbert, J. C.; Selliah, R. D. Enantioselective synthesis of an ent-trichothecene. *Tetrahedron* **1994**, *50* (6), 1651-1664. DOI: https://doi.org/10.1016/S0040-4020(01)80841-3.
(32) Ku, A. F.; Cuny, G. D. Synthetic Studies of 7-Oxygenated Aporphine Alkaloids: Preparation of (−)-Oliveroline, (−)-Nornuciferidine, and Derivatives. *Organic Letters* **2015**, *17* (5), 1134-1137. DOI: 10.1021/acs.orglett.5b00007.
(33) Zhang, W.; Robins, M. J. Removal of silyl protecting groups from hydroxyl functions with ammonium fluoride in methanol. *Tetrahedron Letters* **1992**, *33* (9), 1177-1180. DOI: https://doi.org/10.1016/S0040-4039(00)91889-6.
(34) Hu, Q.-Y.; Rege, P. D.; Corey, E. J. Simple, Catalytic Enantioselective Syntheses of Estrone and Desogestrel. *Journal of the American Chemical Society* **2004**, *126* (19), 5984-5986. DOI: 10.1021/ja048808x.
(35) Ma, Z.; Hu, H.; Xiong, W.; Zhai, H. Formal syntheses of (−)- and (+)-aphanorphine from (2S,4R)-4-hydroxyproline. *Tetrahedron* **2007**, *63* (32), 7523-7531. DOI: https://doi.org/10.1016/j.tet.2007.05.059.
(36) Choi, H.; Engene, N.; Byrum, T.; Hwang, S.; Oh, D.-C.; Gerwick, W. H. Dragocins A−D, Structurally Intriguing Cytotoxic Metabolites from a Panamanian Marine Cyanobacterium. *Organic Letters* **2019**, *21* (1), 266-270. DOI: 10.1021/acs.orglett.8b03712.
